# Diversity, distribution and conservation of land mammals in Mauritania, North-West Africa

**DOI:** 10.1371/journal.pone.0269870

**Published:** 2022-08-01

**Authors:** José Carlos Brito, Andack Saad Sow, Cândida Gomes Vale, Cristian Pizzigalli, Dieng Hamidou, Duarte Vasconcelos Gonçalves, Fernando Martínez-Freiría, Frederico Santarém, Hugo Rebelo, João Carlos Campos, Juan Manuel Pleguezuelos, Maria Joana Ferreira da Silva, Marisa Naia, Pedro Tarroso, Raquel Godinho, Teresa Luísa Silva, Tiago Macedo, Zbyszek Boratyński, Zeine El Abidine Sidatt, Francisco Álvares

**Affiliations:** 1 CIBIO, Centro de Investigação em Biodiversidade e Recursos Genéticos, *InBIO* Laboratório Associado, Campus de Vairão, Universidade do Porto, Vairão, Portugal; 2 BIOPOLIS Program in Genomics, Biodiversity and Land Planning, CIBIO, Vairão, Portugal; 3 Departamento de Biologia, Faculdade de Ciências, Universidade do Porto, Porto, Portugal; 4 Green Sahel Expertise: Bureau d’Études Spécialise en Environnement, Nouakchott, R.I. Mauritanie; 5 Faculté des Sciences et Techniques, Université des Sciences, de Technologie et de Médecine de Nouakchott, Nouakchott, R.I. Mauritanie; 6 Departamento de Zoología, Facultad de Ciencias, Universidad de Granada, Granada, Spain; 7 Department of Zoology, University of Johannesburg, Johannesburg, South Africa; 8 Parc National du Diawling, Nouakchott, R.I. Mauritanie; Zoological Survey of India, INDIA

## Abstract

Detailed knowledge about biodiversity distribution is critical for monitoring the biological effects of global change processes. Biodiversity knowledge gaps hamper the monitoring of conservation trends and they are especially evident in the desert biome. Mauritania constitutes a remarkable example on how remoteness and regional insecurity affect current knowledge gaps. Mammals remain one of the least studied groups in this country, without a concerted species checklist, the mapping of regions concentrating mammal diversity, or a national assessment of their conservation status. This work assessed the diversity, distribution, and conservation of land mammals in Mauritania. A total of 6,718 published and original observations were assembled in a spatial database and used to update the occurrence status, distribution area, and conservation status. The updated taxonomic list comprises 107 species, including 93 extant, 12 Regionally Extinct, and 2 Extinct in the Wild. Mapping of species distributions allowed locating concentrations of extant mammal species richness in coastal areas, along the Senegal River valley, and in mountain plateaus. Recent regional extinction of large-sized Artiodactyla and Carnivora has been very high (11% extinct species). From the extant mammals, 11% are threatened, including flagship species (e.g., *Addax nasomaculatus* and *Panthera pardus*). Species richness is poorly represented by the current protected areas. Despite the strong advances made, 23% of species categorise as Data Deficient. Persisting systematics and distribution uncertainties require further research. Field surveys in currently unexplored areas (northern and south-eastern regions) are urgently needed to increase knowledge about threatened mammals. The long-term conservation of land mammals in Mauritania is embedded in a complex web of socioeconomic and environmental factors that call for collaborative action and investment in sustainable human development. The current work sets the baseline for the future development of detailed research studies and to address the general challenges faced by mammals and biodiversity in the country.

## Introduction

Detailed knowledge about biodiversity distribution is critical for monitoring the biological effects of global change processes [1]. The current extinction crisis further amplifies the need to understand the direct and indirect drivers of change to promote the conservation and sustainable use of natural resources [2]. However, there are several key shortfalls in current biodiversity knowledge. The so-called Linnean shortfall relates to the differences between the numbers of species that actually exist and those that have been already formally described and catalogued, while the Wallacean shortfall refers to the inadequate knowledge about biodiversity distribution [3]. Addressing these shortfalls and filling current knowledge gaps is deemed urgent to meet global biodiversity conservation targets and achieve sustainability goals [4].

Biodiversity knowledge gaps are especially evident in the desert biome, owing to the usual large dimensions and remoteness that characterises most deserts [5]. The African Sahara and the deserts of Central Asia are particularly understudied, with scarce available biodiversity distribution data in relation to other deserts and biomes [6]. The core areas of these deserts are especially under-sampled due to their restricted accessibility and in some cases to the frequent occurrence of armed conflicts [6, 7].

**Fig 1:**
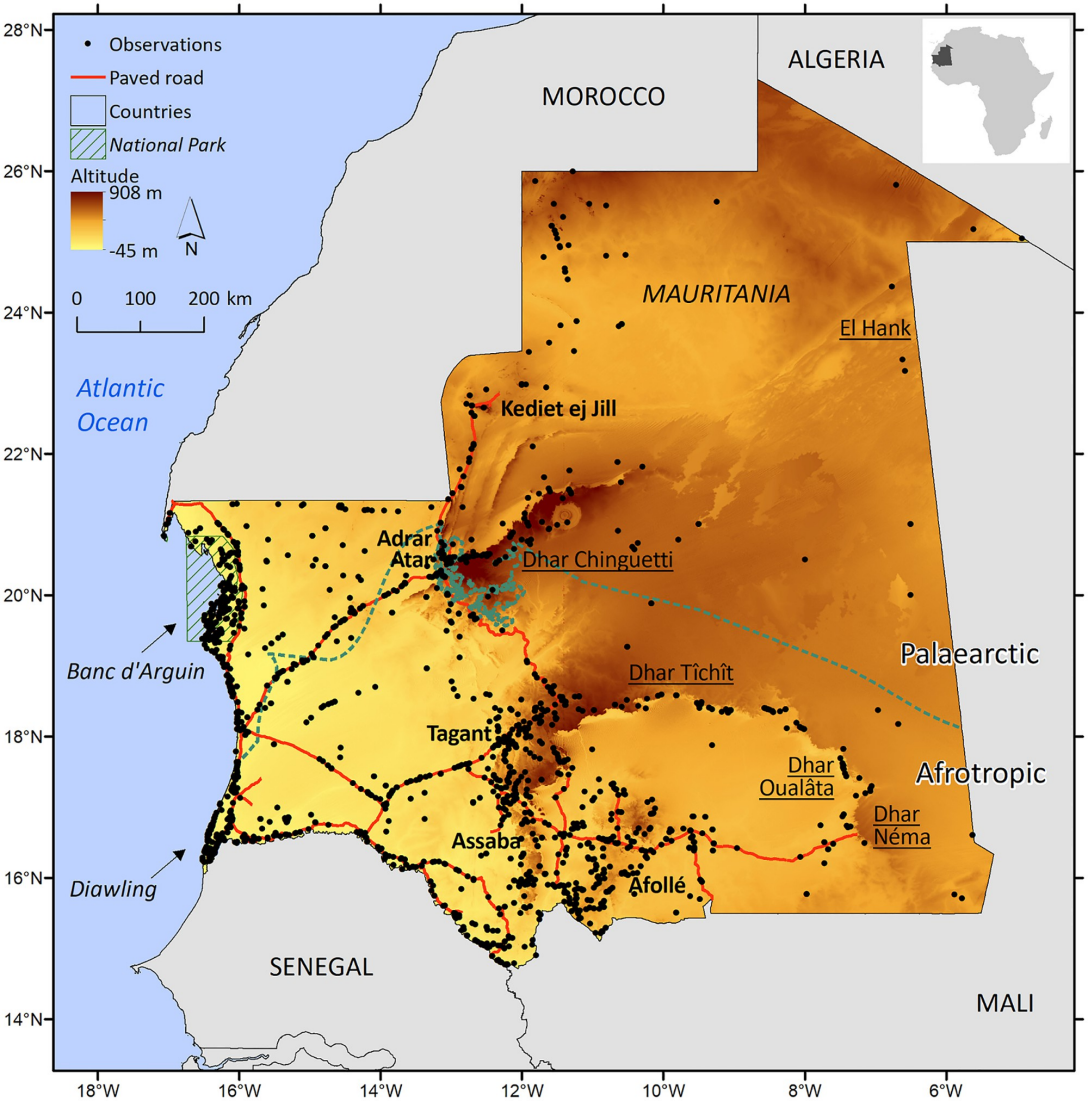
Distribution of observed land mammals in Mauritania. Main mountain plateaus (bold) and escarpments (underlined), national parks (italics), paved roads (red), border between the Palaearctic and Afrotropic biogeographic realms (dashed green line), and location of Mauritania in the African continent (small inset). Altitude from SRTM at 90 m spatial resolution [23].

Mauritania, in North-West Africa, constitutes a remarkable example on how remoteness and regional insecurity affect current biodiversity knowledge gaps (Fig 1). Mauritania, with over a million square kilometres, is essentially a desert country, with nearly 95% of the land surface covered by the Sahara and Sahel ecoregions (S1 Fig), vast expanses of pastoral land and only 0.5% of arable land [8, 9]. There are very few paved roads (S2 Fig), which translates in general remoteness and reduced accessibility to vast inland regions [10, 11]. Regional conflicts in neighbouring countries have affected travel security and land accessibility to Mauritania since the late 1970s, and the country has suffered from an outbreak in terrorism since the mid-2000s [12]. Despite this, the security conditions have ameliorated and according to the global peace index, Mauritania now categorises as Medium Peace (ranking 118 out of 163 countries analysed; [13]). Although socioeconomic conditions have improved over the last decades, Mauritania still categorises as Low Human Development according to the Human Development Index of the United Nations (ranking 157 out of 189 countries; [14]). The perceived levels of public sector corruption are poor and Mauritania scores as low as 29 (maximum 100) according to the corruption perception index (ranking 134 out of 180 countries; [15]). The combination of poor socioeconomic indicators with generalised regional insecurity has hampered field surveys throughout time and translated into reduced knowledge about biodiversity distribution [6]. In the beginning of the 21^st^ century, Mauritania was possibly one of the countries within North Africa with less biodiversity data available. Accordingly, only two national parks, Banc d’Arguin and Diawling (Fig 1), have been designated, which taken together only cover about 1% of the land area of Mauritania [16], a value clearly below the 17% national protected area targets set by the Aichi Biodiversity Targets [17] for more details see [18]). In fact, Mauritania is amongst the 40 most highly underfunded countries for biodiversity conservation [19]. At the same time, the country experienced numerous droughts after the 1960s that induced internal migration to coastal cities and population fixation along the southern main paved road axis, which was built to provide support to vulnerable human communities [20]. After the 1980s, there were strong efforts made in infrastructure development and in the exploitation of natural resources (e.g., mining, oil operations), which additionally increased the challenges to biodiversity conservation. However, given that human population is only about 4.5 million (estimate for 2019), that the density is 4.4 inhabitants per square kilometre, and that more than 50% live in urban areas (S2 Fig), Mauritania is one of the least populated countries in the world [21]. Thus, despite the growing human population trend, increasing infrastructures, and agriculture expansion, Mauritania still harbours large areas of almost undisturbed habitats (S2 Fig). Over 75% of the land area of the country still classifies as Last of the Wild [22].

Under the above described context, the authors of the current study from the research group BIODESERTS (Biodiversity of Deserts and Arid Regions) of CIBIO (Research Center in Biodiversity and Genetic Resources, University of Porto, Portugal; https://cibio.up.pt/en/groups/biodiversity-of-deserts-and-arid-regions-biodeserts) and local colleagues (University of Nouakchott, Diawling National Park) developed yearly field surveys in Mauritania between 2002 and 2021. The surveys were dedicated to collect species distribution data and to fill out biodiversity knowledge gaps. The research efforts were developed using contemporary tools and analytical processes, including surveying (camera-trapping), molecular (DNA sequencing and genotyping) and geomatic (Global Navigation Satellite Systems and Geographical Information Systems) tools. Consequently, there is now a growing body of literature about the distribution of various organisms from multiple taxonomic groups in Mauritania (e.g., [24—27]). Most studies focused on amphibians and reptiles, including their diversity and distribution in the two national parks [28, 29], inferences about their biogeographic patterns and evolutionary histories (e.g., [30, 31]), and barcoding assessments [32]. Still, these studies are also revealing knowledge gaps and exposing severe threats to biodiversity conservation in Mauritania [5, 33, 34]. Mammals in particular remain one of the least studied groups in the country.

Mammals are a diverse group of vertebrates that show specific adaptations to arid environments [35]. Globally, they include a high number of threatened and iconic species that are frequently targeted for conservation actions. Still, there are extensive knowledge gaps about their diversity, distribution, and conservation in Mauritania. A tentative list considered 109 species, comprising 81 land and 28 marine species [36], while a survey on the IUCN Red List database retrieved 118 species, 87 land and 31 marine species [37]. Besides the discrepancies in species numbers, there are uncertainties in the local occurrence of certain species that result indistinct statuses of regional occurrence (e.g., leopard; [37, 38]). Recent updates in the biogeography and systematics of different mammal species or groups [39—44] further call for a comprehensive assessment of their diversity and an update of the species list in the country. Knowledge about mammal distribution in Mauritania has increased with a series of publications from the Atlantic coastal region (e.g., [45, 46]) and the Adrar Atar mountains [47], but available distribution data from inland and remote areas is scarce and displays coarse spatial resolution (e.g., [48, 49]). Ecological niche-based models have been performed for multiple taxonomic groups when high spatial resolution distribution data (e.g. 1x1km) are available (e.g., [50—53]). However, despite the attempts to provide comprehensive data on mammal distribution and to understand local patterns in the distribution of mammal diversity, the available data are still dispersed in partial publications (e.g. [36, 53, 54]) and database collections [55]. Knowledge on the conservation status of mammals in Mauritania is worryingly limited. Large-scale poaching, poisoning, and trafficking have led to the decline and eventual extinction of the elephant and large-sized Artiodactyla and Carnivora representatives [7, 56]. Threats to the survival of mammals and their isolated freshwater habitats in mountains have been preliminarily identified [33, 34]. However, a national list of the conservation status of the mammals of Mauritania is presently lacking. This, together with the mapping of regions representing high concentration of mammal species diversity, is critical information for the establishment of future conservation action on these vertebrates.

This work addresses the current knowledge gaps in the diversity, distribution and conservation of land mammals in Mauritania. The current work aims to answer the following questions: 1) how many extant species of land mammals occur in Mauritania? 2) how many extinctions have occurred since the year 1900? 3) how are mammals spatially distributed? 4) how is mammal species richness distributed? 5) how many threatened mammals occur in Mauritania? and 6) how is mammal richness presently represented in the current network of protected areas of Mauritania? We expect to unveil patterns in mammal distribution and to provide the national conservation status, which will help setting the baseline for future research studies and conservation actions in the country.

## Materials and methods

### Study area

Mauritania (1,043,030 km^2^) is located in the transition zone between the Palaearctic and Afrotropic biogeographic realms, and is covered by eight ecoregions, with the largest extension represented by the South Sahara Desert and the Sahelian Acacia savanna ecoregions in the northern and southern parts of the country, respectively (Fig 1 and S1 Fig). Mauritania comprises a system of four mountain plateaus, Adrar Atar, Tagant, Assaba and Afollé, one mountain massif, Kediet ej Jill, and five escarpments, El Hank, Dhar Chinguetti, Dhar Tîchît, Dhar Oualâta, and Dhar Néma (Fig 1). Wetlands are absent from large sections in northern Mauritania, while in central and mountain regions they are scarce, predominantly isolated, and ephemeral, and the Senegal River in the southwest is the only permanent water source (S3 Fig). There is strong latitudinal gradient in the distribution of annual precipitation and aridity, with northern Mauritania being drier and more arid in comparison to southern regions; northern Mauritania, coastal areas included, are colder in comparison to inland and other coastal regions, and north-eastern Mauritania has higher continentality in comparison to south-western regions (S4 Fig). Mauritania is mostly covered by sand dunes (33.5%; [57]) and 11 main sand seas and three sandy gravel plains can be distinguished (S5 Fig). Other relevant land-cover categories include gravel and sand floodplains (24.2%), compact soil (19.7%), bare rock and rocky soil (8.1%), grasslands (8.8%), and savannah (2.8%) (S6 Fig). The country is divided into 12 administrative units plus the capital Nouakchott (S7 Fig). There are two national parks that are also Ramsar sites (Banc d’Arguin and Diawling), both coastal and mostly targeted at the protection of migratory and wintering Palaearctic birds, and another two Ramsar sites, the coastal Chott Boul and the Lac Gabou in the Tagant plateau (S8 Fig). The Banc d’Arguin National Park is adjoined by the Cap Blanc Reserve, and the Diawling NP is on the northern side of the Senegal River delta and forms part of the Senegal River Delta Transboundary Biosphere Reserve. In 2016, the Awleigatt zoological park (fenced area) has been upgraded to national park category [58].

### Fieldwork

Twenty overland expeditions to Mauritania were performed between 2002 and 2021 by the CIBIO team for collecting species distribution data. Field surveys and animal sample collection were made in accordance with national laws and authorised by the Ministry of Environment and Durable Development of Mauritania (permits 2012-827/MDAPMCEDD/SG, 2015-162/Dir/PND, 2015-100/Dir/PND, 2016-166/D/PND, 2017-063/MEDD/PND/D).

The expeditions were carried out annually from September to January, except in 2009 (March-May), 2015 (August-September), 2017 (April), and 2021 (June), resulting in a total of 660 work/days, and about 85,000 km covered (S9 Fig). Sampling was performed by two up to 11 persons (average: 4.8 persons by expedition). Mammal data were collected using the following methods: 1) opportunistic sampling along roads for roadkill specimens; 2) random walks during the daytime and after sunset searching for presence signs (e.g. faeces, carcasses, vocalizations), and to detect and capture rodents with hand nets in specific habitats; 3) regular trapping for small-mammals, performed between 2011 and 2015 (total 3,527 trap-nights; average 24.32 traps/night) followed by opportunistic trappings; 4) opportunistic camera-trapping on 112 localities, performed between 2011 and 2020 (S10 Fig), with an average of 5 cameras/locality (ranged from 1 to 9 cameras/night); 5) opportunistic observations (e.g., along roads while driving); 6) opportunistic interviews to knowledgeable local people (e.g., shepherds and fisherman), using the pictures from a field guide of African mammals as reference [59] about the occurrence or extinction decade of large-sized mammals in the region (e.g., hippopotamus, elephant, giraffe, lion, leopard, hyenas, non-human primates). Interviews were informal and unstructured, and ensured the anonymity of people interviewed (not asked interviewees names or any other identification information that would link the answers to the specific individual or household) and the replies were kept confidential; and 7) compilation of reliable mammal sightings meetings with the staff of the Diawling National Park. Overall, sampling was biased towards regions around paved roads and main tracks, protected areas, and inland wetlands located in the four main mountains of Mauritania (S9 Fig).

In total, 1,943 mammal observations were collected during expeditions organised by CIBIO team. Of these, 158 observations were collected by camera-trapping and 282 specimens were captured and handled, including trapped small mammals and a few bats captured in nets (S10 Fig). A total of 1,079 biological samples were collected, including tissue, hair, horns, bones, skins, quills and faeces, including 271 roadkill specimens (S10 Fig), and stored in 95% ethanol. Reference digital photographs were also taken when possible. Live specimens were released in the capture site after data collection. A total of 71 roadkill specimens were kept and deposited in the CIBIO tissue collection. All observations were georeferenced with a Global Positioning System (GPS) receiver on the WGS84 datum.

### Laboratory work

Out of the 1,079 samples collected by the CIBIO team, 597 were selected for molecular species identification (S10 Fig). Identifications targeted specific mammal groups, such as ungulates, canids, felids, hares, non-human primates, hedgehogs, jerboas, and gerbils, and was performed by sequencing of mitochondrial and/or nuclear DNA (for details, see [40—44, 60—67]), and in some cases also by genotyping (for details see [41, 44, 64, 65, 67]).

### Bibliographic references

A total of 6,008 published observations were collected from 104 bibliographic references, mostly from the GBIF-Global Biodiversity Information Facility (5,031 observations; [55, 68]) (full list of references available in S1 Text). These included 1,145 observations that were used in previous surveys of mammal diversity in Mauritania and surrounding countries [33, 50—54, 69]. It also included 44 observations confirmed by molecular species identification [39, 70—79]. The geographic coordinates of observations were extracted from publications when available, or georeferenced from the descriptions of localities using 1:200,000 maps (Institut National de l’Information Géographique et Forestière; https://www.ign.fr/) when unavailable.

### Data treatment

Initially, all observations were added to a database and duplicated observations were searched and merged. These included for instance, citations of the same individual/sample in molecular and distributional studies. This filtering process resulted in a total of 6,718 individual observations available for this study. These included 6,008 already published observations (89.4%), and 710 original observations (10.6% of all observations), including 641 samples molecularly identified to species level (9.5% of all observations).

Observations were grouped according to four time periods (S11 Fig): 1) from after the year 2000, comprising 2,322 observations (34.6% of total observations) collected within the scope of the sampling efforts developed for this study; 2) from between the years 1980 and 1999, comprising 923 observations (13.7%) collected after the major landscape impacts resulting from human activities; 3) from between the years 1900 and 1979, comprising 3,382 observations (50.3%) collected before the major landscape impacts resulting from human activities; and 4) from before the year 1900, comprising 91 observations (1.4%) from rock-art and subfossils collected in archaeological sites (dating is uncertain but it may be from at least c.4,000 years ago). Observations collected from interviews were allocated to year/decade when the mammal was reported to occur in the region. Observations of horns and other bone remains found in the wild (specifically of *Addax nasomaculatus*) were estimated as belonging to the period 1980—1999 given their decomposition status.

### Diversity analyses

The taxonomic status of the 6,718 observations collected was reviewed and updated following the Mammal Species of the World [80]. This work was followed as a reference to generate the taxonomic list of land mammals of Mauritania, and disagreements in taxonomy with the IUCN Red List [37] were annotated. Observations of jerboas (*Jaculus* sp.) with no genetic confirmation (N = 164) were considered only to the genus level given its challenging identification based strictly on morphological characters [44, 61, 81]. Additionally, 2,269 observations of small mammals from museums [55] were only identified to the genus level: *Gerbillus* sp. (N = 2,224), *Mastomys* sp. (N = 41), and *Crocidura* sp. (N = 4).

The occurrence status of each species in Mauritania was defined according to the time period of observations and published literature: 1) Extant—species that presently occur in Mauritania; 2) Extinct—species that have gone extinct in Mauritania, either when the extinction was previously reported in the literature, when the species was not observed in the last 20 years from well surveyed areas (e.g. Diawling National Park [82]) where it used to be present, or when observations are available only from before the year 1900; and 3) Extinct in the Wild—species that have gone extinct in Mauritania and that presently occur in the country in enclosed fences as a result of captive programmes in the Awleigatt National Park [58].

### Distribution analyses

The metadata of all observations were added to a georeferenced database linked with a Geographical Information System (Arc GIS 10.5) [83]. The GIS was used to display observations based on the time period (see above) over a 100 km grid cell size (136 cells) on the projected coordinate system Africa Albers Equal Area Conic (S12 Fig). The observed ranges of each species were then confronted with the range polygons from IUCN Red List [37], to identify endemic and nearly-endemic (>75% of the global range) species to Mauritania, and disagreements in range configuration or limits in the country were annotated. The 2,433 observations of *Jaculus*, *Gerbillus*, *Mastomys* and *Crocidura* available only to the genus level were represented in the distribution maps of all species of each genus.

Species richness maps were produced by summing individual species distributions in each 100 km grid cell. Richness maps were produced for total species, for the most specious mammal Orders (Artiodactyla, Carnivora, Chiroptera and Rodentia), and for nationally threatened species (categories Vulnerable, Endangered, and Critically Endangered) plus Near Threatened species [37], following the application of IUCN criteria to the national level of Mauritania (see below). Estimates of species richness excluded all observations from: 1) before the year 1900, to exclude rock-art and subfossil observations; 2) species with occurrence status of Regionally Extinct and Extinct in the Wild; 3) captive populations of species categorised as Extant; 4) individuals identified only up to genus level (*Jaculus* sp., *Gerbillus* sp., *Mastomys* sp., *Crocidura* sp.); 5) introduced species (*Mus musculus*, *Rattus rattus*); and 6) Extant species from localities of reported local extinction, specifically: a) *Ammotragus lervia* from Kediet ej Jill massif; b) *Eudorcas rufifrons*, *Gazella dorcas*, *Hippopotamus amphibius*, and *Papio papio* from the Senegal River delta and Diawling National Park; and d) *Addax nasomaculatus* and *Panthera pardus* from before the year 2000 in well-surveyed localities reporting local extinction. The current network of protected areas of Mauritania was overlapped with the richness maps to identify potential gaps in the representation of mammal richness within the existing protected areas. The Awleigatt National Park was excluded from these analyses due to its *ex-situ* conservation purpose (fenced site).

### Conservation analyses

The conservation status of each species was evaluated according to the Red List Criteria [84] in its application at the national level [85]. Given the wide knowledge gaps about mammals in Mauritania, there was considerable uncertainty in the evaluation of each species against the criteria. For example, it was not possible to estimate population trends nor the area of occupancy using the standardised 2 km grid cell size, given the large size of the country, the vast areas still unsurveyed, and the coarse geographic coordinates of several bibliographic observations. For other parameters, population estimates were only given to selected large-sized mammals based on expert-opinion and literature (e.g., [33, 56, 69, 86—88]). Estimates on Extent of Occurrence (EOO) were carried out in the GIS on the projected coordinate system Africa Albers Equal Area Conic. The EOO was derived from a convex polygon encompassing all observations of each species considered for the calculations of species richness (see above), and with a minimum of three observations. The individual polygons were then clipped by the limits of Mauritania to exclude marine areas and areas in neighbouring countries. Data on population fragmentation was estimated from the individual mapped distributions. Ecological data available for several species (e.g., [51—53, 69]) allowed to infer the number of subpopulations, based on the potential isolation resulting from unsuitable habitat types acting as barrier to dispersal (S5 Fig), as well as decreases in the extent of habitat quality, threats to specific habitats, and exploitation levels of selected species.

The resulting national conservation statuses of Mauritanian mammals followed guidelines for application at the regional level, relying on three premises [85]: 1) if the taxon is endemic to the country or if the national population of a species to be assessed is isolated from conspecific populations outside the country, the criteria must be used without modification; 2) if the population within the country could undergo a rescue effect from populations outside the country, the resulting category of extinction risk must be downgraded; 3) if the population within the country is a demographic sink and the extra-regional source is expected to decrease or cannot perform a rescue effect, the extinction risk of the country population may be upgraded. The approximate proportion of the global population of each species within Mauritania was not calculated because of the imprecise baseline data available and the relatively low levels of mammal endemicity in the country. The final national conservation statuses were confronted with the global conservation statuses available from the Red List [37]. The conservation status categories and respective abbreviations were: RE—Regionally Extinct; EW—Extinct in the Wild; CR—Critically Endangered; EN—Endangered; VU—Vulnerable; NT—Near Threatened; LC—Least Concern; DD—Data Deficient; and NA—Not Applicable. Extinction rates were calculated only considering species with observations after the year 1900, given the lack of earlier literature listing mammals from Mauritania (S1 Text). The lists of occupied habitats and of threats affecting Mauritania land mammals following the IUCN standard classification schemes of habitats [89] and threats [90], respectively, were inferred from the distribution of observations of each species in relation to the distribution of major land-cover categories (S5 Fig) and human activities (S2 Fig).

## Results

### Sampling effort

**Fig 2:**
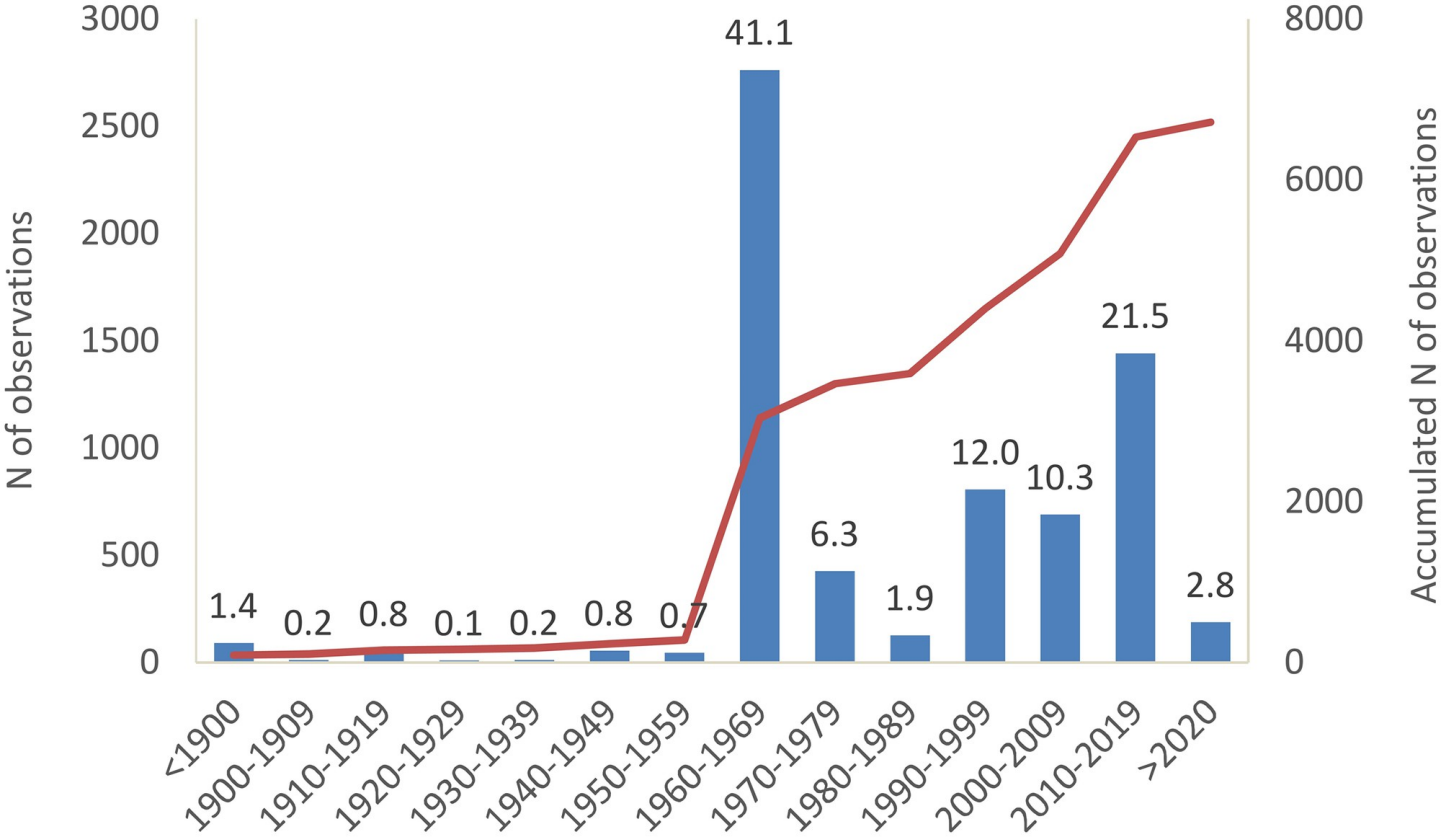
Number of reported observations of land mammals in Mauritania along decadal time periods in the 20^th^ and 21^st^ centuries. Numbers above bars represent the percentage of observations in each time period. Line depicts accumulated number of observations (N). Observations from before the year 1900 are merged.

There was a peak in the number of observations from the period of 1960—1969 (41%; Fig 2), resulting from the expeditions by the Smithsonian Institution African Mammal Project carried out between February 1967 and January 1968 [55]. Still, most observations were collected from after the year 1990 (47%), partially resulting from increased fieldwork efforts by the authors after the year 2000 (S9 Fig). The spatial distribution of the observations showed strong geographic biases in sampling efforts to the south-western coastal areas, the protected areas, the Adrar Atar, and partially the Tagant and Assaba plateaus (S13 Fig). There were vast inland regions unsampled or without a single mammal observation (about 30% of the 100 km grid cells covering the country).

**Fig 3:**
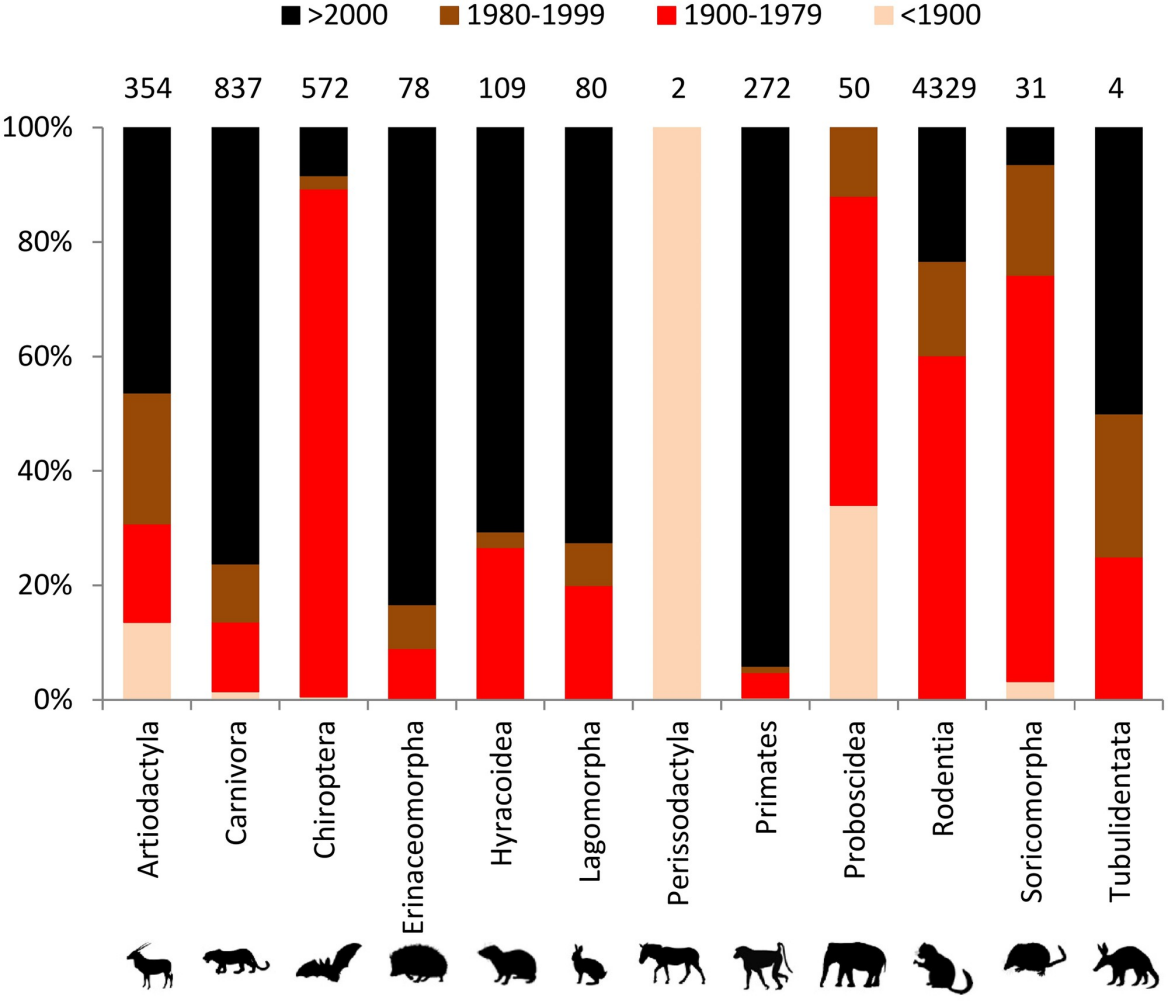
Percentage of observations of land mammals in Mauritania in each time period category by mammal Order. Numbers above bars represent the total number of observations in each mammal Order.

Most of the recent observations (time period >2000) were from mammal Orders Carnivora, Erinaceomorpha, Hyracoidea, Lagomorpha, and Primates (Fig 3). Most observations of Orders Chiroptera, Rodentia, and Soricomorpha were from the time periods 1900—1979 and 1980—1999, and there were no observations of Order Proboscidea in the time period >2000. The observations from Order Perissodactyla were exclusively from the time period <1900. The Order Rodentia accumulates the largest number of observations (64.4%) which is partially consequence of the expeditions of the Smithsonian Institution African Mammal Project.

### Diversity

The taxonomic list of land mammals of Mauritania includes 107 species distributed by 12 Orders and 32 Families (Table 1). The full dataset of 6,718 observations is provided in S2 Text and S3 Text. There were three species (*Taurotragus derbianus*, *Ictonyx libyca*, *Euxerus erythropus*) with disagreements in taxonomy between Mammal Species of the World [79] and IUCN Red List [37]. The 107 land mammals of Mauritania comprise 1) 93 Extant species, including four species of uncertain taxonomic status (*Hipposideros cf*. *caffer*, *Lepus ssp*., *Jaculus cf*. *hirtipes*, *Praomys cf*. *daltoni*), and two introduced species (*Mus musculus*, *Rattus rattus*); 2) 12 Regionally Extinct species of which the vast majority are large-sized Artiodactyla (*Damaliscus lunatus*, *Hippotragus equinus*, *Kobus kob*, *Nanger dama*, *Redunca redunca*, *Taurotragus derbianus*, *Tragelaphus scriptus*, *Lycaon pictus*, *Acinonyx jubatus*, *Panthera leo*, *Ceratotherium simum*, *Loxodonta africana*); and 3) two species Extinct in the Wild with captive individuals in the Awleigatt National Park (*Oryx dammah*, *Giraffa camelopardalis*). The most frequent species in number of observations (N>150) were *Gerbillus gerbillus*, *Felovia vae*, *Canis lupaster*, *Papio papio*, *Nycteris hispida*, and *Euxerus erythropus*.

**Table 1:** Taxonomic list of land mammals of Mauritania.

Taxon	GRL	NRL	N obs	N UTM (%)
**Artiodactyla**				
** Bovidae**				
***Addax nasomaculatus* (de Blainville, 1816)**	CR	CR C2a(ii); D	20	1 (0.7)
***Ammotragus lervia* (Pallas, 1777)**	VU	EN B1a,b(i,iii,v); C2a(i); D	8	2 (1.5)
***Damaliscus lunatus* (Burchell, 1824)** ^a^	LC	RE	3	
***Eudorcas rufifrons* (Gray, 1846)**	VU	EN C2a(i)	22	11 (8.1)
***Gazella dorcas* (Linnaeus, 1758)**	VU	VU C2a(i)	116	22 (16.2)
***Hippotragus equinus* (É. Geoffroy Saint-Hilaire, 1803)** ^a^	LC	RE	4	
***Kobus kob* (Erxleben, 1777)** ^a^	LC	RE	5	
***Nanger dama* (Pallas, 1766)** ^a^	CR	RE	29	
***Oryx dammah* (Cretzschmar, 1826)** ^a^	EW	EW	19	
***Redunca redunca* (Pallas, 1767)** ^a^	LC	RE	4	
***Taurotragus derbianus* (= *Tragelaphus derbianus*) (Gray, 1847)** ^a^	VU	RE	2	
***Tragelaphus scriptus* (Pallas, 1766)** ^a^	LC	RE	9	
**Giraffidae**				
***Giraffa camelopardalis* (Linnaeus, 1758)** ^a^	VU	EW	40	
**Hippopotamidae**				
***Hippopotamus amphibius* Linnaeus, 1758**	VU	CR C2a(i); D	10	3 (2.2)
**Suidae**				
***Phacochoerus africanus* (Gmelin, 1788)**	LC	LC	63	13 (9.6)
**Carnivora**				
** Canidae**				
***Canis lupaster* Hemprich and Ehrenberg, 1832**	LC	LC	251	42 (30.9)
***Lycaon pictus* (Temminck, 1820)** ^a^	EN	RE	1	
***Vulpes pallida* (Cretzschmar, 1826)**	LC	LC	100	23 (16.9)
***Vulpes rueppellii* (Schinz, 1825)**	LC	LC	73	25 (18.4)
***Vulpes zerda* (Zimmermann, 1780)**	LC	LC	98	31 (22.8)
** Felidae**				
***Acinonyx jubatus* (Schreber, 1775)** ^a^	VU	RE	8	
***Caracal caracal* (Schreber, 1776)** ^b^	LC	NT C2a(i)	8	5 (3.7)
***Felis margarita* Loche, 1858**	LC	LC	10	6 (4.4)
***Felis silvestris* Schreber, 1777**	LC	LC	61	26 (19.1)
***Leptailurus serval* (Schreber, 1776)** ^b^	LC	VU B1a,b(i,iii); C2a(i)	7	4 (2.9)
***Panthera leo* (Linnaeus, 1758)** ^a^	VU	RE	16	
***Panthera pardus* (Linnaeus, 1758)**	VU	CR C2a(i); D	22	2 (1.5)
** Herpestidae**				
***Atilax paludinosus* (G.[Baron] Cuvier, 1829)**	LC	DD	4	4 (2.9)
***Herpestes ichneumon* (Linnaeus, 1758)**	LC	LC	5	4 (2.9)
***Herpestes sanguineus* (Rüppell, 1835)**	LC	LC	10	5 (3.7)
***Ichneumia albicauda* (G. Cuvier, 1829)**	LC	LC	10	7 (5.1)
** Hyaenidae**				
***Crocuta crocuta* (Erxleben, 1777)** ^b^	LC	VU C2a(i)	11	7 (5.1)
***Hyaena hyaena* (Linnaeus, 1758)** ^b^	NT	NT C2a(i)	25	17 (12.5)
** Mustelidae**				
***Aonyx capensis* (Schinz, 1821)**	NT	DD	4	4 (2.9)
***Ictonyx libyca* (= *Ictonyx libycus*) (Hemprich & Ehrenberg, 1833)**	LC	LC	16	10 (7.4)
***Ictonyx striatus* (Perry, 1810)**	LC	DD	3	3 (2.2)
***Mellivora capensis* (Schreber, 1776)**	LC	LC	33	18 (13.2)
** Viverridae**				
***Civettictis civetta* (Schreber, 1776)**	LC	LC	15	7 (5.1)
***Genetta genetta* (Linnaeus, 1758)**	LC	LC	46	16 (11.8)
**Chiroptera**				
** Emballonuridae**				
***Taphozous nudiventris* Cretzschmar, 1830** ^a^	LC	DD	2	1 (0.7)
***Taphozous perforatus* É. Geoffroy, 1818**	LC	NT B1a	3	3 (2.2)
** Hipposideridae**				
***Asellia tridens* (É. Geoffroy, 1813)**	LC	LC	52	6 (4.4)
***Hipposideros cf*. *caffer* (Sundevall, 1846)** ^c^	NE	NT B1a	16	4 (2.9)
***Hipposideros tephrus* Cabrera, 1906** ^b^	LC	LC	6	3 (2.2)
** Molossidae**				
***Mops condylurus* (A. Smith, 1833)**	LC	DD	105	2 (1.5)
***Tadarida aegyptiaca* (É. Geoffroy, 1818)**	LC	DD	1	1 (0.7)
** Nycteridae**				
***Nycteris gambiensis* (K. Andersen, 1912)** ^a^	LC	DD	1	
***Nycteris hispida* (Schreber, 1775)** ^b^	LC	LC	179	2 (1.5)
***Nycteris macrotis* Dobson, 1876** ^b^	LC	LC	3	2 (1.5)
***Nycteris thebaica* É. Geoffroy, 1818**	LC	DD	3	1 (0.7)
** Pteropodidae**				
***Eidolon helvum* (Kerr, 1792)**	NT	LC	25	5 (3.7)
** Rhinolophidae**				
***Rhinolophus fumigatus* Rüppell, 1842**	LC	DD	1	1 (0.7)
***Rhinolophus landeri* Martin, 1838**	LC	DD	1	1 (0.7)
** Rhinopomatidae**				
***Rhinopoma cystops* Thomas, 1903**	LC	LC	29	4 (2.9)
***Rhinopoma hardwickii* Gray, 1831**	LC	LC	42	6 (4.4)
***Rhinopoma microphyllum* (Brünnich, 1792)**	LC	LC	50	2 (1.5)
** Vespertilionidae**				
***Eptesicus floweri* (de Winton, 1901)**	LC	DD	2	1 (0.7)
***Nycticeinops schlieffeni* (Peters, 1859)**	LC	LC	46	10 (7.4)
***Pipistrellus rueppellii* (Fischer, 1829)**	LC	LC	4	3 (2.2)
***Scotophilus leucogaster* (Cretzschmar, 1826)**	LC	DD	2	1 (0.7)
**Erinaceomorpha**				
** Erinaceidae**				
***Atelerix albiventris* (Wagner, 1841)**	LC	LC	18	12 (8.8)
***Paraechinus aethiopicus* (Ehrenberg, 1832)**	LC	LC	60	28 (20.6)
**Hyracoidea**				
** Procaviidae**				
***Procavia capensis* (Pallas, 1766)**	LC	LC	109	16 (11.8)
**Lagomorpha**				
** Leporidae**				
***Lepus spp*. Linnaeus, 1758** ^c^	NE	NA	80	42 (30.9)
**Perissodactyla**				
** Rhinocerotidae**				
***Ceratotherium simum* (Burchell, 1817)** ^a^	NT	RE	2	
**Primates**				
** Cercopithecidae**				
***Chlorocebus sabaeus* (Linnaeus, 1766)** ^b^	LC	NT C2a(i)	13	8 (5.9)
***Erythrocebus patas* (Schreber, 1774)**	NT	LC	59	17 (12.5)
***Papio papio* (Desmarest, 1820)**	NT	VU C2a(i)	199	11 (8.1)
** Galagidae**				
***Galago senegalensis* É. Geoffroy Saint-Hilaire, 1796**	LC	DD	1	1 (0.7)
**Proboscidea**				
** Elephantidae**				
***Loxodonta africana* (Blumenbach, 1797)** ^a^	EN	RE	50	
**Rodentia**				
** Ctenodactylidae**				
***Felovia vae* Lataste, 1886** ^d^	LC	LC	276	20 (14.7)
** Dipodidae**				
***Jaculus cf*. *hirtipes* (Lichtenstein, 1823)** ^c^	NE	LC	28	14 (10.3)
***Jaculus jaculus* (Linnaeus, 1758)**	LC	LC	88	24 (17.6)
** Hystricidae**				
***Hystrix cristata* Linnaeus, 1758**	LC	LC	53	23 (16.9)
** Muridae**				
***Acomys airensis* Thomas & Hinton, 1921**	LC	LC	35	13 (9.6)
***Arvicanthis niloticus* (É. Geoffroy, 1803)**	LC	LC	110	14 (10.3)
***Desmodilliscus braueri* Wettstein, 1916**	LC	LC	61	14 (10.3)
***Gerbillus amoenus* (de Winton, 1902)**	LC	LC	141	18 (13.2)
***Gerbillus campestris* (Loche, 1867)**	LC	LC	54	14 (10.3)
***Gerbillus gerbillus* (Olivier, 1801)**	LC	LC	312	34 (25)
***Gerbillus henleyi* (de Winton, 1903)**	LC	LC	5	4 (2.9)
***Gerbillus nancillus* Thomas & Hinton, 1923**	DD	LC	14	7 (5.1)
***Gerbillus nigeriae* Thomas & Hinton, 1920**	LC	LC	60	18 (13.2)
***Gerbillus pyramidum* Geoffroy, 1825**	LC	LC	66	10 (7.4)
***Gerbillus tarabuli* Thomas, 1902**	LC	LC	107	33 (24.3)
***Mastomys erythroleucus* (Temminck, 1853)**	LC	LC	7	5 (3.7)
***Mastomys huberti* (Wroughton, 1909)**	LC	LC	6	3 (2.2)
***Meriones crassus* Sundevall, 1842**	LC	LC	39	7 (5.1)
***Meriones libycus* Lichtenstein, 1823**	LC	DD	1	1 (0.7)
***Mus haussa* (Thomas & Hinton, 1920)**	LC	LC	5	3 (2.2)
***Mus musculus* Linnaeus, 1758**	LC	NA	34	7 (5.1)
***Pachyuromys duprasi* Lataste, 1880**	LC	LC	39	6 (4.4)
***Praomys cf*. *daltoni* (Thomas, 1892)** ^c^^,^^d^	NE	VU B1a,b(iii)	11	3 (2.2)
***Psammomys obesus* Cretzschmar, 1828**	LC	LC	28	8 (5.9)
***Rattus rattus* (Linnaeus, 1758)**	LC	NA	5	3 (2.2)
***Taterillus arenarius* Robbins, 1974**	LC	LC	113	10 (7.4)
***Taterillus gracilis* (Thomas, 1892)**	LC	DD	2	1 (0.7)
***Taterillus pygargus* (F. Cuvier, 1838)**	LC	DD	20	2 (1.5)
***Taterillus tranieri* Dobigny, Granjon, Aniskin, Ba & Volobouev, 2003** ^b^^,^^d^	LC	LC	2	1 (0.7)
** Sciuridae**				
***Euxerus erythropus* (= *Xerus erythropus*) Desmarest, 1817**	LC	LC	178	29 (21.3)
**Soricomorpha**				
** Soricidae**				
***Crocidura cinderella* Thomas, 1911**	LC	DD	1	1 (0.7)
***Crocidura fuscomurina* (Heuglin, 1865)**	LC	DD	1	1 (0.7)
***Crocidura lusitania* Dollman, 1915**	LC	LC	8	6 (4.4)
***Crocidura nanilla* Thomas, 1909**	LC	DD	5	1 (0.7)
***Crocidura olivieri* (Lesson, 1827)**	LC	DD	1	1 (0.7)
***Crocidura viaria* (I. Geoffroy, 1834)**	LC	LC	11	7 (5.1)
**Tubulidentata**				
** Orycteropodidae**				
***Orycteropus afer* (Pallas, 1766)**	LC	DD	4	4 (2.9)

For each species (following the nomenclature in [80] it is presented the Order, Family, the IUCN Red List category at the global level (GRL), the IUCN Red List category and criteria at the national level (NRL), the number of observations (N obs), the number of 100x100 km UTM squares and percentage occupied by each extant species (N UTM (%)). For species with disagreements in taxonomy, the alternative name by IUCN Red List is given in brackets.

^a^Species excluded from estimations of species richness (*)

^b^Species with the final national conservation status downgraded during the Red List assessment (-)

^c^Extant species with uncertain taxonomic status (?)

^d^Endemic or nearly-endemic species (#).

A total of 11 extant species currently unlisted by the IUCN Red List [37] as part of the land mammals of Mauritania were reported in this study: *Hippopotamus amphibius*, *Leptailurus serval*, *Panthera pardus*, *Hipposideros tephrus*, *Mops condylurus*, *Tadarida aegyptiaca*, *Eidolon helvum*, *Paraechinus aethiopicus*, *Gerbillus campestris*, *Mus musculus*, *Rattus rattus*. Another three taxa with uncertain taxonomic status, *Hipposideros cf*. *caffer*, *Jaculus cf*. *hirtipes*, *Praomys cf*. *daltoni*, may likely represent new species for the country. On the contrary, a total of 19 species listed by IUCN as possibly occurring in Mauritania were excluded from the current species list (S4 Text). These included 10 species that were not reported in this study or their taxonomic status is outdated or uncertain. Another nine species have been reported to potentially occur in the country but they were excluded because there are no reliable observations and additional sampling efforts are required before confirming the occurrence status.

### Distribution

Six species were found in more than 30 grid cells (above 20% of the area of the country), including *Canis lupaster*, *Lepus spp*., *Gerbillus tarabuli*, *Vulpes zerda*, *Euxerus erythropus*, and *Paraechinus aethiopicus* (Table 1). All species belonging to the Orders Chiroptera, Soricomorpha and Tubulidentata, about one third of the species of the Order Rodentia, and nearly two thirds of the Order Carnivora were found in 10 or less grid cells.

Mapping of individual species distributions together with notes for particular species and observations are given in the supplementary material (S14 Fig). From the extant species in Mauritania, one is endemic to the country (*Praomys cf*. *daltoni*) and two were nearly endemic (*Felovia vae*, *Taterillus tranieri*), with marginal populations extending into Mali.

There were differences in the observed range of several species in Mauritania in comparison to the IUCN range polygons [37]. Considering the 93 extant species, range differences occurred in 52 species that can be grouped in three main patterns: 1) 11 species that presently are not considered to occur in Mauritania for which this study provides observations in the country. These are *Hippopotamus amphibius*, *Leptailurus serval*, and *Eidolon helvum* along the Senegal River valley, *Panthera pardus* in the Banc d’Arguin National Park and southern Assaba plateau, *Hipposideros tephrus* and *Paraechinus aethiopicus* in western Mauritania (the later also in the mountain plateaus), *Mops condylurus* in the Senegal River delta, *Tadarida aegyptiaca* in the Afollé plateau, *Gerbillus campestris* in the Adrar Atar, Tagant, Assaba plateaus, *Mus musculus* scattered in urban areas, *Rattus rattus* in Nouâdhibou and Diawling National Park (although all observations are from before the year 2000); 2) 38 species for which this study suggests larger ranges in the country in comparison to what is currently known. These include 2.1) 17 species with generally northwards increase in the known range mostly along the Senegal River valley or into the mountain plateaus and coastal areas (*Phacochoerus africanus*, *Caracal caracal*, *Atilax paludinosus*, *Herpestes ichneumon*, *Herpestes sanguineus* [but see comment on S14 Fig], *Ichneumia albicauda*, *Civettictis civetta*, *Nycticeinops schlieffeni*, *Pipistrellus rueppellii*, *Atelerix albiventris*, *Chlorocebus sabaeus*, *Hystrix cristata*, *Acomys airensis*, *Arvicanthis niloticus*, *Gerbillus henleyi*, *Crocidura fuscomurina*, *Crocidura lusitania*), 2.2) 10 species with generally southwards increase in the known range (*Gazella dorcas* [but see comment on S14 Fig], *Felis margarita*, *Rhinopoma hardwickii*, *Gerbillus nigeriae*, *Meriones crassus*, *Meriones libycus*, *Pachyuromys duprasi*, *Psammomys obesus*, *Taterillus arenarius*, *Crocidura oliveiri*), 2.3) six species with generally eastwards increase in the known range (*Felis silvestris*, *Nycteris hispida*, *Rhinopoma cystops*, *Gerbillus amoenus*, *Taterillus pygargus*), and 2.4) four species with varied range increases (*Rhinolophus landeri*, *Nycteris macrotis*, *Nycteris thebaica*, *Taphozous perforatus*, *Jaculus jaculus*); and 3) three species for which the current dataset provides a better definition of their range in the country in comparison to what is currently known. These are *Vulpes zerda* (apparently absent from the mid- and upper Senegal River valley and south-eastern Mauritania), *Mellivora capensis* (apparently absent from inland/eastern Mauritania), and *Procavia capensis* (apparently absent from coastal areas and restricted to the rock outcrops of mountains and escarpments). Considering the 14 species now listed as Regionally Extinct or Extinct in the Wild at national level, the mapped distributions provided a better definition of the former range in the country of *Kobus kob* (formerly occurred also in the lower Senegal River valley) and *Oryx dammah* (formerly occurred also in eastern Mauritania), and evidences for the current absence of *Redunca redunca* and *Tragelaphus scriptus* that are depicted in IUCN range polygons as occurring in southern Mauritania [37].

**Fig 4:**
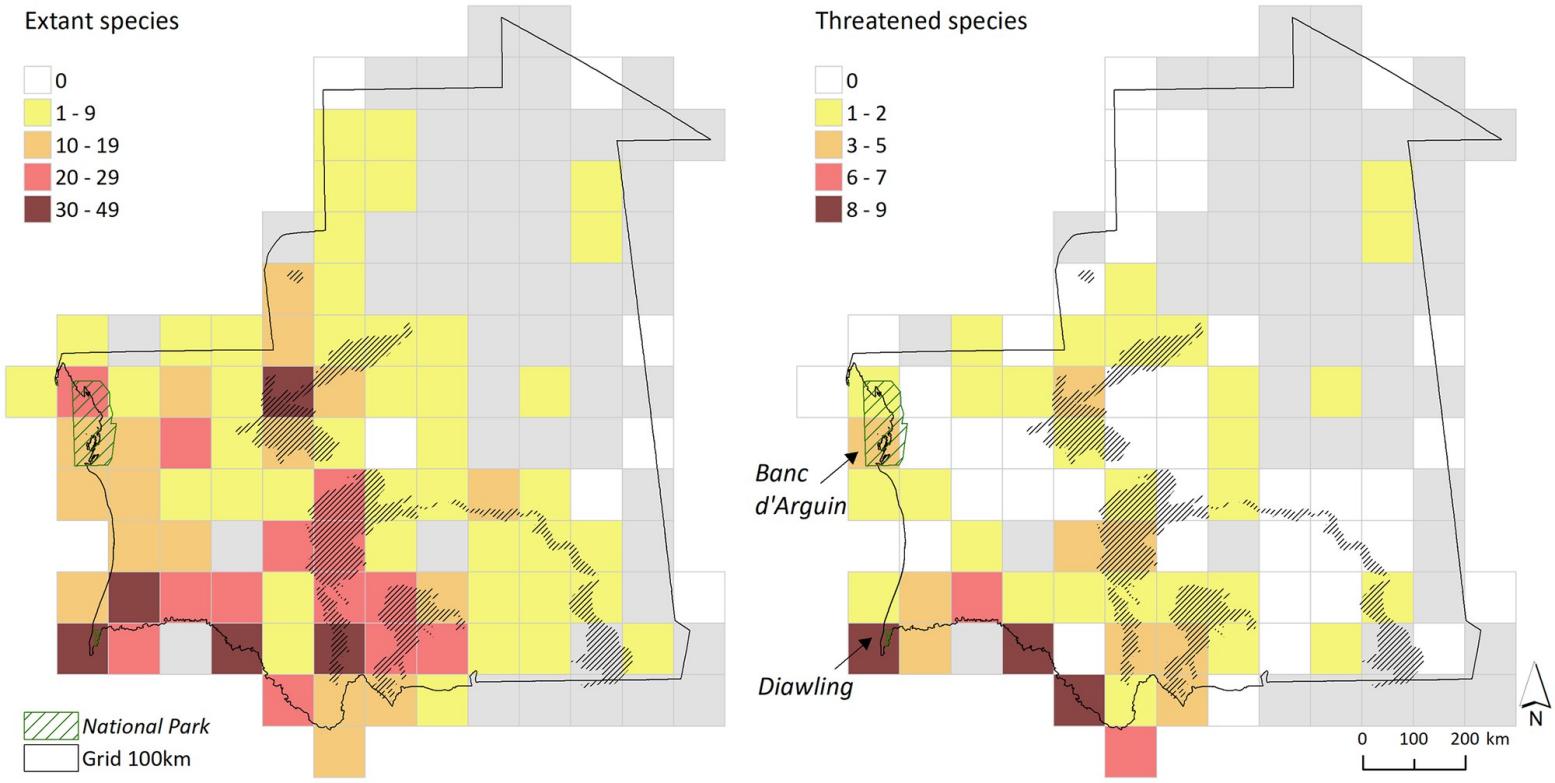
Distribution of mammal species richness in Mauritania based on 100x100 km grid cells. Left: extant species. Right: species listed as Near Threatened, Vulnerable, Endangered, and Critically Endangered at the national level according to the current study. Unsampled grid cells are marked (light grey). Mountain plateaus and escarpments (black dashed) and national parks (green shaded; in italics) are identified.

Species richness was the highest along the Senegal River valley and in the mountain plateaus (Fig 4 left). This pattern holds identical within the most specious mammal Orders of Artiodactyla, Carnivora, Chiroptera, and Rodentia, with the Banc d’Arguin National Park also containing high species richness of Carnivora and Rodentia (S15 Fig).

### Conservation

From the 107 land mammals of Mauritania, the assessment of the extinction risk following IUCN criteria was excluded for three species: *Lepus* spp. due to the taxonomic uncertainties and the two introduced Rodentia. Downgrading of the original national Red List status (NRL) was performed in nine species based on the potential rescuing effect from populations originating in neighbouring countries (for details see S5 Text and individual species in S14 Fig). The most frequent occupied habitats by land mammals in Mauritania are shrublands and rock areas (S6 Text), while the most frequent threats affecting them are related to climate change and severe weather (S7 Text).

**Fig 5:**
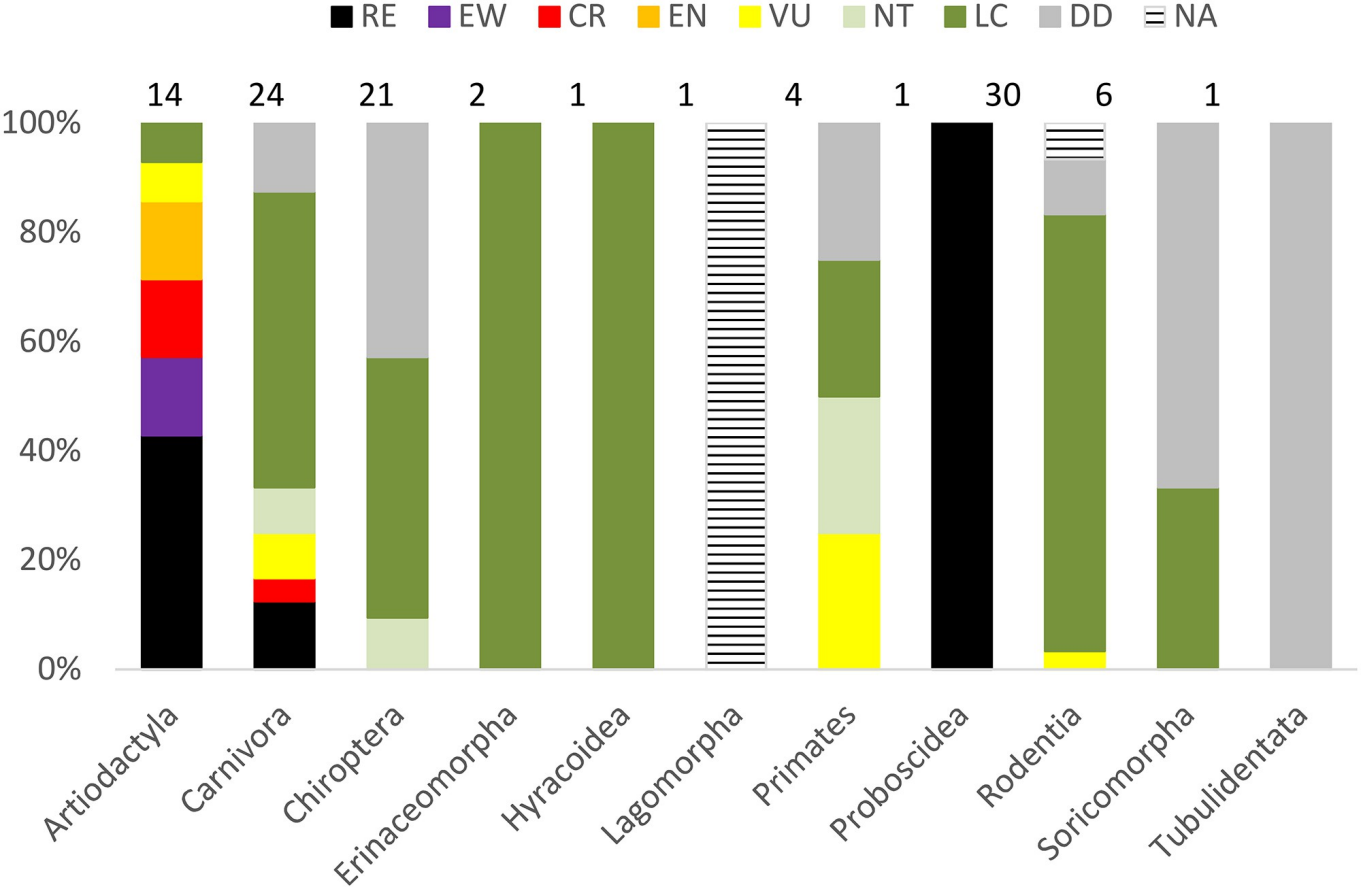
IUCN Red List category at the national level of land mammals of Mauritania. Percentage of species (total N = 107) in each conservation status category [37] by mammal Order. Numbers above bars represent the total number of species in each mammal Order. The species *Ceratotherium simum* and *Taurotragus derbianus* were not considered in calculations.

A total of 14 species were categorised as RE and EW (Table 1), 10 of which were known to occur in Mauritania until the 1980s, two until the 1990s, and two before the year 1900 (S16 Fig). The mammal Orders containing most of these species were the Artiodactyla (60% of the species in the Order), Carnivora (13%), and also Perissodactyla and Proboscidea, with presently no extant species in the country (Fig 5). Excluding *Ceratotherium simum* and *Taurotragus derbianus* because all available observations correspond to subfossil remains dating from before year 1900, the remaining 12 extinct species represent 11.4% of the mammal diversity in the country. The last regions of occurrence for these extinct species were: 1) the upper Senegal River valley in the Province of Guidimaka for *Damaliscus lunatus*, *Hippotragus equinus*, *Kobus kob*, *Redunca redunca*, and *Lycaon pictus*; 2) the Senegal River valley for *Oryx dammah*, *Tragelaphus scriptus*, *Giraffa camelopardalis*, *Panthera leo*, and *Loxodonta africana*; and 3) the mountain plateaus together with the Province of Guidimaka for *Nanger dama* and *Acinonyx jubatus*. The Senegal River valley was the region accumulating the highest number of species lost, but the greatest reduction in relation to the original known richness occurred in the northern and eastern regions (S17 Fig).

From the 90 extant species assessed, Mauritania comprises 10 threatened species (11% of land mammals) with IUCN Red List category at the national level (NRL) of VU (five species), EN (two species), and CR (three species) (Table 1). The mammal Orders containing threatened species according to the NRL were the Artiodactyla (83% of the extant species in the Order categorised as CR, EN or VU), the Primates (25% as VU), the Carnivora (14% as CR or VU), and Rodentia (3% as VU) (Fig 5). Another five species were categorised as NT (5% of land mammals), and 21 species categorised as DD (23% of land mammals). The mammal Orders containing DD species were the Tubulidentata (100% of the extant species in the Order), Soricomorpha (67%), Chiroptera (43%), Primates (25%), Carnivora (14%), and Rodentia (10%).

Considering the set of 105 species (excluding *Ceratotherium simum* and *Taurotragus derbianus* because all observations available are before the year 1900), the NRL listed 53 species (50%) in a distinct extinction risk category in comparison to the global level (GRL) (Table 1). Most of the discrepancies occurred in species categorised by the GRL as LC being categorised by the NRL as DD, NT or RE, and also from species globally categorised as VU to RE at national level (S18 Fig). The number of threatened species categorised according to the GRL decreased from 13 species (12% of 93 extant species) down to 10 species (11%) according to the NRL. However, the number of DD species categorised according the GRL increased from one (1% of extant) up to 21 species (23%) by the NRL.

The highest concentration of threatened or Near-Threatened species was found along specific sections of the Senegal River valley and with lower intensity in the mountain plateaus (Fig 4 right). There were huge gaps in the representation of mammal species richness in the current network of protected areas of Mauritania, either considering total species richness or richness of threatened mammals. Except for the Diawling National Park where mammals are protected, the mountain plateaus together with the mid-upper Senegal River valley are important protection gaps.

## Discussion

The current work provides a comprehensive data compilation about land mammals of Mauritania. The updated species list includes 93 extant, 12 Regionally Extinct, and 2 Extinct in the Wild species. For the first time, it was assembled the individual distributions of 107 mammal species and mapped their spatial distribution and richness. The conservation status at the national level shows that 15% of the extant land mammals of Mauritania are threatened or Near-Threatened, and that mammal species richness is poorly represented in the current network of protected areas of Mauritania. Despite the recent and considerable advances in the knowledge about mammals in the region, there are still several uncertainties and persisting knowledge gaps, as expressed by the 23% of land mammals categorised as Data Deficient. The knowledge advances, the persisting gaps, and the directions in future research, are discussed in the following sections.

### Diversity

The updated list of land mammals of Mauritania considers 93 extant species, an increase by 6 up to 12 species comparing to the previous assessments [36, 37]. Amongst the new species detected, there are two globally Vulnerable species (*Hippopotamus amphibius*, *Panthera pardus*) and one Near Threatened (*Eidolon helvum*), as well as new marginal populations for *Leptailurus serval* and *Panthera pardus* (for details on these species and on the following cases, see individual distribution maps and explanatory texts in S14 Fig). More than 50% of the new species for the country are from the Orders Chiroptera and Rodentia, which indicates that additional surveys and molecular research in these taxonomic groups are still needed. There are systematic uncertainties remaining and poor distribution knowledge in various species, especially in *Hipposideros cf*. *caffer*, *Lepus spp*., *Jaculus cf*. *hirtipes* and *Praomys cf*. *daltoni*. In the latter two cases, there is comprehensible genetic data sustaining that lineages found in Mauritania are largely differentiated from the rest of the species complex and might represent unrecognised species (*Jaculus*: [40, 44, 60, 61, 81]; *Praomys*: [43]). Yet, in the case of *Jaculus cf*. *hirtipes* it is warranted formal species recognition by the Mammals of the World and IUCN Red List [37, 80], and in the case of *Praomys cf*. *daltoni* it is needed the formal species description. In the case of *Hipposideros* and *Lepus*, there are still several doubts about the systematics of these species [67, 91, 92] that need to be clarified before precise identification of species occurring in Mauritania. The future assessment of the taxonomic status of these species should help improve the current knowledge gaps on species diversity.

Current knowledge gaps are translated in a relatively high percentage of mammals categorised as DD (23% of extant species), in comparison to mammals at the global level (14%; [93]), especially in species within the Orders Chiroptera and Soricomorpha. A future comprehensive barcoding assessment of mammal diversity in the country should be prioritised, similar to the one recently made for amphibians [32]. This barcoding initiative could profit from the many sample vouchers available in museum collections outside the country. For instance, there are over 3,500 sample vouchers of small mammals from Mauritania (including 2,000 of *Gerbillus* sp.) in the collections of the Natural History Museums of Paris and the Smithsonian Institute [55] collected in the 1960s and 1990s (Fig 2). Together with additional field collected samples, these can provide the basis for thorough barcoding assessments.

### Distribution

The assembly of the distribution data on land mammals of Mauritania developed herein provided the basis for mapping the distributions of 107 species, including extant and extinct species. However, the available distribution data are biased towards coastal and mountain regions (S13 Fig), while more than 30% of the area of the country remains completely unsampled (Fig 4). Vast inland sections along the eastern border with Algeria and Mali do not contain a single mammal observation. The geographical sampling biases affect many aspects of the current work, for instance in the spatial identification of concentrations of species richness or in the estimation of parameters for applying the IUCN Red List criteria (e.g., extent of occurrence and area of occupancy). To circumvent sampling biases, ecological modelling may be used to complete sampling gaps and estimate potential ranges for species with representative sampling sizes [33, 50—53, 69]. The database of observations produced in this work (S2 Text and S3 Text) provides framework data for the future application of ecological modelling tools. Nonetheless, field surveys are still required before ecological models can be applied, especially concerning: 1) species with low number of observations, particularly species from Orders Carnivora, Chiroptera, Rodentia, and Soricomorpha; and 2) species possibly occurring in eastern and south-eastern Mauritania, which still require additional observations from those regions to ensure that the full range of environmental conditions where these species occur is represented in the ecological models [52].

The inland areas close to the border with Mali contain Critically Endangered mammals that require urgent field surveys to confirm their occurrence and range. The extended and remote Ouarâne sand dunes (S5 Fig) of eastern Mauritania may have been a refuge for a relict population of *Addax nasomaculatus* (details on observation in S14 Fig). Likewise, the neighbouring Erg Ijâfene, Iguîdi and Chech may still provide suitable conditions for this persisting population. However, the single most recent observation of this species is from the year 2007, and there is no updated information about its status. Another example is the case of *Panthera pardus*, where the available information (details on S14 Fig) reports occurrence in the extreme southern Afollé plateau (close to the Malian border) and in the Banc d’Arguin National Park, calls for dedicated field surveys in these regions. Despite the logistic constraints of sampling remote areas, the national and global conservation statuses of these flagship species demand for concerted action.

Large numbers of mammal species were found in coastal areas, along the Senegal River valley, and mountain plateaus (Fig 4), and these areas also hold the one endemic (*Praomys cf*. *daltoni*) and two nearly endemic (*Felovia vae*, *Taterillus tranieri*) mammals to Mauritania. These areas are located in the Sahel (S1 Fig) and experience colder temperatures and more humid conditions throughout the year in comparison to inland regions (S4 Fig). The Senegal River provides a permanent water source (S3 Fig), and the mountains contain isolated rock-pools (locally known as *guelta*), generally small sized and seasonal, that also represent water sources [33]. In drylands, rainfall and water availability are critical factors in vegetation dynamics and thus affect local productivity levels [20, 35]. This tends to amplify in the Sahel and areas experiencing humid conditions or in proximity to water sources provide the best possible conditions for establishing mammal communities with diversified trophic webs. Still, the sampling biases towards some of these areas certainly affected the spatial identification of areas of high species richness. Field surveys in currently unexplored areas, such as the northern and south-eastern regions, may reveal other locally relevant patterns of mammal species richness.

### Conservation

The assembly of the distribution data on land mammals of Mauritania here developed provided the basis for mapping the distributions of 14 Regionally Extinct and Extinct in the Wild species along temporal periods (S14 Fig). The spatial and temporal patterns of range contraction that emerge from this study locate the Senegal River valley (especially the Province of Guidimaka) and the mountain plateaus as the last strongholds for species that have disappeared from other locations. These areas still concentrate large numbers of extant mammals, suggesting their enhanced role for the conservation of mammal diversity in Mauritania. The factors for the decline of extinct species are well documented and are mostly related with overhunting for multiple purposes, including meat consumption, human-wildlife conflicts, and poaching for the international traffic (e.g. [7, 56]). The magnitude of these activities tends to increase in periods of conflict or drought, and the Sahel is an especially vulnerable area within the African context [94]. This confers strong vulnerability to the current areas of refugia for threatened mammals in Mauritania (Fig 4) to fluctuations in social security and climate change, especially under projected scenarios of growing human population [21]. For instance, the formerly remote areas of the Dakhlet-Nouâdhibou and Inchiri provinces have become much more accessible after the paved road linking Nouâdhibou with the capital Nouakchott was completed in 2005, which now supports a network of exploratory tracks for established and prospecting gold mining operations (S2 Fig; [authors, pers. observ.]). Besides unsustainable, these operations likely affect the remaining populations of large-sized mammals, either by disturbance or poaching. As such, ensuring the mechanisms for sustainable human development and anticipating the impacts of climate change by designing adaption measures, is fundamental to safeguard the long-term conservation of the remaining mammal diversity in Mauritania.

A total of 10 land mammals of Mauritania (11.1% of the 90 extant species evaluated against IUCN criteria) were classified as threatened, which is a low percentage in comparison to the 22.2% of the global mammal fauna evaluated so far (5,954 species). However, a total of 14 species (11.4% of the 105 species assumed to be present in Mauritania in the year 1900) were classified as RE or EW in recent times, which is a much higher number in comparison to the 1.4% of the global mammal fauna [93], and depict the fast pace at which extinctions are recently occurring for mammal species in the country. Downgrading of the national extinction risk category with respect to the global one was performed in nine out of the 90 extant species evaluated due to the potential rescuing effect from populations occurring in neighbouring countries [37], but the extinction risk may be upgraded in the future if such population connectivity is interrupted. The current work also demonstrated the importance of applying the IUCN Red list criteria to the national level, as most changes occurred from species globally categorised as LC to nationally categorised as DD, NT or RE, and also from globally VU to nationally RE. Also, the extinction risk at the national level increased in several species already globally categorised as threatened (*Ammotragus lervia*, *Eudorcas rufifrons*, *Hippopotamus amphibius*, *Panthera pardus*), or in species globally considered as LC (*Caracal caracal*, *Leptailurus serval*, *Crocuta crocuta*, *Papio papio*). Still, the present assessment of the national conservation status should be interpreted as preliminary. Although it was based on the best available data and on the knowledge accumulated over nearly 20 years of field surveys, there are still several uncertainties associated with the data needed to apply the IUCN Red List criteria [37]. These include knowledge gaps in most species about spatial and temporal dynamics in population size, fragmentation and trends, which can undergo changes related to short (seasonal) and long term (climate change) oscillations [95]. Additional field surveys together with long-term monitoring data on population demographic trends are essential for estimating patterns in this arid region.

There were 10 mammals classified as threatened of extinction at the national level. These species should be targeted for population assessment and habitat protection under future monitoring schemes. The priority areas harbouring these species for surveys are: 1) the mountain plateaus and escarpments for *Ammotragus lervia*, *Papio papio*, *Praomys cf*. *daltoni*, and *Panthera pardus* (for the later also the Banc d’Arguin National Park); 2) the Senegal River delta, valley and major tributaries for *Hippopotamus amphibius* and *Leptailurus serval*; 3) the northern and eastern Mauritania for *Addax nasomaculatus* and *Gazella dorcas*; and 4) the southern Mauritania for *Eudorcas rufifrons* and *Crocuta crocuta*. Taken together, the sampling of these areas represents almost all of Mauritania, and thus may be a challenging task. However, dedicated surveys to each of these areas in the future would allow covering many of the knowledge gaps addressed so far.

The Artiodactyla is the mammal Order containing most species considered extinct (nine out of the 14 Regionally Extinct or Extinct in the Wild) and threatened (five out of the 10 threatened) in Mauritania. Despite the well-documented extensive human pressure over wild ungulates [56, 96], few actions have been promoted to improve the conservation status of the globally threatened species. To this respect, the gazetting of the Awleigatt National Park is a remarkable initiative that can be used to promote *ex-situ* conservation of native wild ungulates. The site has the potential to be used as transferring or breeding facility in the scope of future population reinforcement programmes of *Addax nasomaculatus*, *Ammotragus lervia*, *Eudorcas rufifrons*, *Gazella dorcas*, and *Nanger dama*. So far, the park hosts populations of two Extinct in the Wild species in Mauritania, *Oryx dammah* and *Giraffa camelopardalis* [58]. However, the geographic origin of the founder population should be determined to understand the levels of genetic diversity and taxonomic units that are available to be considered for reintroduction into the wild [97]. The park also currently hosts another 13 exotic ungulates from various geographic origins that are kept under captive breeding programmes. Safety measures need to be thoroughly applied to prevent accidental escaping from the breeding facilities. Overall, the Awleigatt National Park displays great potential to take a leading role in regional large-mammal conservation while current efforts need to focus on supporting future reintroduction programmes strictly of native ungulates.

Mammal species richness is presently poorly represented in the current network of protected areas of Mauritania. Although the Banc d’Arguin and Diawling cover important levels of species richness, there are major representation gaps along the Senegal River valley and in the mountain plateaus (Fig 4). This poor representation is expected given the overall low performance of Mauritania in meeting protected area targets [16], which has been related also with poor performance in conserving large-sized mammals in West African countries [56]. Protected areas together with local community engagement in conservation are key tools in securing the survival of Sahara-Sahel megafauna, while sustainably developing the economy, as well as regional peace and stability [7]. As such, to ensure the representation of mammal species richness in protected areas, it could be considered a system of micro-reserves based on: 1) the rehabilitation and formal protection of the former Classified Forest designated along the Senegal River Valley. These high-standing and dense forests result from the seasonal flooding of the river valley and are important nesting sites for birds and breeding habitats for amphibians [27, 32]. They would have the potential to be used as stepping-stone habitats along the main course of the Senegal River by aquatic and semi-aquatic mammals, as well as forest dwelling species; and 2) the formal protection of mountain rock-pools and the associated drainage systems that ensure seasonal connectivity [98], and gene flow between isolated populations of water-dependent taxa, as demonstrated in crocodiles [99]. Water availability in the mountain rock-pools has also been identified as critical environmental factor associated with the occurrence of many rock-specialist mammals and other fauna [33, 51—53, 69]. Still, the allocation of conservation resources should be optimised by the future design of conservation planning scenarios that target both the representation (species richness) and the persistence (genetic diversity and population connectivity) of mammals. To do so, the current knowledge gaps in distribution need to be reduced and the barcoding assessments need to be completed.

## Conclusion

This work provides the first description of the patterns in the diversity, distribution, and conservation of land mammals of Mauritania, and sets a baseline for the future development of management actions and detailed research studies in the country. These studies are in need to fill out persisting knowledge gaps. Together with the fact that most mammals are nocturnal, elusive or cryptic, or any combination of those, low accessibility and remoteness place logistical challenges to accomplish field surveys. However, the global and national conservation statuses of the several threatened mammals calls for collaborative action, especially given the observed fast pace of regional extinction of Mauritanian mammals in the last few decades, as shown by this study. In fact, these logistic challenges are minor aspects comparing to the challenges placed to the long-term mammal conservation. The landscapes where mammal diversity currently dwells are being challenged by climate change, land degradation and lack of social, urban and territory planning in a growing human population context [21]. Mauritania is also amongst the top-five countries unable to retain top talents, and brain drain deprives the country from the human resources required to drive and implement the structural changes needed for reverting biodiversity loss while promoting sustainable human development [100]. To improve human development and ensure the protection of natural resources, conservation actions should be community-based, by integrating the experiences and ambitions of local people in national management decisions. Potential future reintroduction programmes need to be supported by organised local human communities to maximise its probability of success. To this respect, the promotion of sustainable livelihoods should be prompt as an alternative to the current (and historical) agro-pastoralism model, where land-use tends to promote overgrazing, water abstraction and habitat loss, and is extremely vulnerable to climate oscillations [20, 33]. Diversifying job opportunities via sustainable development is a potential option where ecotourism may play a leading role [101]. Mauritania displays multiple natural values that may support ecotourism operations: 1) large sections of the country still contain important richness of Sahara-Sahel flagship mammals [102]; 2) the mountain rock-pools and floodplains have been ranked as highly adequate to support distinct types of ecotourism activities [103]; and 3) the coastal areas, mountain plateaus, and the Senegal River valley are amongst the best localities displaying cultural ecosystem services in the Sahara-Sahel [104] that are priority for conservation [105]. The long-term conservation of land mammals in Mauritania is thus embedded in a complex web of constraining socioeconomic and climatic factors. The advances in knowledge provided by the current work set the baseline to address the general challenges faced by mammals and biodiversity in the country, as well as in the western Sahara-Sahel.

## Supporting information

S1 Fig Ecoregions. Distribution of terrestrial ecoregions in Mauritania following [1] and border between the Palaearctic and Afrotropic biogeographic realms (dashed green line).
(DOCX)

S2 Fig Human activities. Distribution of populated places [1], mining for exploitation of natural resources [updated from 2], paved (red line) and unpaved (grey line) roads [updated from 3], railways (black line) [updated from 2], human footprint [4], human influence index [5], global accessibility to cities [6], and Last of the Wild [5].
(DOCX)

S3 Fig Hydrographic network. Hydrographic network (including the Senegal River) [1], location of major seasonal wetlands and mountain rock pools (*Guelta*) [2], and digital elevation model of Mauritania [3].
(DOCX)

S4 Fig Climate. Distribution of annual mean temperature [1], annual precipitation [1], aridity index [2], and continentality index (average temperature of warmest month—average temperature of coldest month) [3] in Mauritania.
(DOCX)

S5 Fig Land-cover. Distribution of major land-cover categories [1] in Mauritania. The main sand dune areas (underlined) and sandy gravel plains (italics) are identified.
(DOCX)

S6 Fig Habitats. Representative pictures of the major land-cover categories found in Mauritania [1] and of the habitats frequently mentioned throughout the texts. Codes of the localities of pictures are mapped.
(DOCX)

S7 Fig Administrative units. Administrative regions (coloured areas; shadowed text) and capital of provinces [1] in Mauritania.
(DOCX)

S8 Fig Protected areas. Location of National Parks and Ramsar sites designated in Mauritania [1]. The Banc d’Arguin National Park is adjoined by the Cap Blanc Reserve, which protects the largest breeding colony of Mediterranean Monk Seal within its global range. A zoological park (Awleigatt) has been upgraded to national park category in 2016 [2] but it is not yet listed in the World Database of Protected Areas [1].
(DOCX)

S9 Fig Sampling routes. Sampling routes taken by CIBIO team between 2002 and 2021, distribution of mammal observations collected during surveys and by collaborators, and national parks of Mauritania [1].
(DOCX)

S10 Fig Examples of types of observations. Distribution of selected examples of types of field observations (black dots) of land mammals in Mauritania in relation to all compiled observations (grey dots): Camera trapping—observations collected by camera-trapping; Molecular species id.—observations with species identification confirmed by barcoding; Road-kills—observations of roadkill mammal specimens; and Captured—observations of live captured mammal specimens.
(DOCX)

S11 Fig Time periods of observations. Distribution of observations (black dots) in four time periods in relation to all observations (grey dots) of land mammals in Mauritania: >2000 —observations from after the year 2000; 1980—1999 —observations from between the years 1980 and 1999; 1900—1979 —observations from between the years 1900 and 1979; and <1900 —observations from before the year 1900.
(DOCX)

S12 Fig Reference grid UTM 100 km. Alphanumeric code of each 100 km grid cell size on the projected coordinate system Africa Albers Equal Area Conic. External units on X- and Y-axes refer to the Universal Transverse Mercator (UTM) coordinate system.
(DOCX)

S13 Fig Distribution of number of observations. Number of observations of land mammals in Mauritania in each 100 km UTM grid cell. Mountain plateaus and escarpments (black dashed) and national parks (green shaded; in italics) are identified.
(DOCX)

S14 Fig Species distribution maps. Status and distribution maps of land mammals in Mauritania. Comparisons between the distribution maps and the range polygons available from IUCN [1] are given below figure captions. The extinction risk category at the global [1] and national level (this study) are presented and commented when pertinent. References provided in legends are available in S1 Text.
(DOCX)

S15 Fig Distribution of species richness by mammal order. Distribution of species richness of land mammals in Mauritania at 100x100 km UTM scale in the most specious mammal orders: Artiodactyla, Carnivora, Chiroptera, and Rodentia. Grid cells without a single mammal observation are marked (light grey). Mountain plateaus and escarpments (black dashed) and national parks (in italics) are identified.
(DOCX)

S16 Fig Time of last observation of non-extant species. Time of last known observation of Regionally Extinct (RE) and Extinct in the Wild (EW) mammal species in Mauritania. For the EW species with captive individuals (*), the time of release on captivity is marked as +. The observations collected for *Ceratotherium simum* and *Taurotragus derbianus* were strictly from before the year 1900.
(DOCX)

S17 Fig Temporal change in the distribution of species richness. Top: Distribution of mammal species richness in Mauritania based on 100x100 km grid cells considering all species assumed to be present in the country by the year 1900. Bottom left: Number of species lost in each grid cell in comparison to the distribution of extant species richness (mapped in Fig 4). Bottom right: Percentage of species lost in each grid cell. Unsampled grid cells are marked (light grey). Mountain plateaus and escarpments (black dashed) and national parks (green shaded; in italics) are identified.
(DOCX)

S18 Fig Global and national conservation status. Percentage of species listed in each category of extinction risk [1] at the global (GRL) and the national (NRL; Mauritania) levels. The source and destination of the main changes in conservation status are marked by arrows.
(DOCX)

S1 Text Bibliographic references. List of bibliographic references from where mammal observations were extracted.
(DOCX)

S2 Text Dataset of observations. Dataset of observations of land mammals in Mauritania (excluding observations available at GBIF) including the code of the observation (Code), Order, Family and Species name (Species), the Latitude and Longitude in decimal degrees (WGS84 projection), the code of the UTM 100 km grid cell size of the observation (UTM100), and when available the reference to molecular or observational data (Ref). Codes of UTM 100 km cells are available in S12 Fig. References are available in S1 Text.
(DOCX)

S3 Text Dataset of observations. Dataset of observations of land mammals in Mauritania available at GBIF, including the code of the observation (Code), Order, Family and Species name (Species), the Latitude and Longitude in decimal degrees (WGS84 projection), the code of the UTM 100 km grid cell size of the observation (UTM100; codes are available in S12 Fig).
(DOCX)

S4 Text Taxonomic list of land mammals with reported occurrence in Mauritania not considered. For each species (following the nomenclature of Mammal Species of the World; [1]) it is presented and discussed the details on the reported observation or range. For species with disagreements in taxonomy, the alternative name given by IUCN Red List is given between brackets.
(DOCX)

S5 Text National Red List assessment. Details on the national conservation status assessment of land mammals species in Mauritania following the recommendations of IUCN [1, 2], including the global Red List status (GRL; [3]), the original national Red List status (NRL Original) and criteria, the final national Red List status (NRL Final) after considering if each species is endemic or nearly-endemic to Mauritania (Endemism), if the Mauritanian populations are a sink in relation to neighbouring populations (Sink), if Mauritanian populations can be rescued by neighbouring populations (Rescue) which implied a downgrading of the NRL Original (Graded). The extent of occurrence (EOO) and comments on calculation are also provided.
(DOCX)

S6 Text Habitats. List of occupied habitats by land mammals in Mauritania following the IUCN standard habitat classification scheme [1].
(DOCX)

S7 Text Threats. List of threats affecting land mammals categorised as threatened (CR, EN, VU) and Near-Threatened (NT) under the national Red List assessment following the IUCN standard threats classification scheme [1].
(DOCX)
