# Supplementary material for: Diversity, distribution and conservation of land mammals in Mauritania, North-West Africa
Source: PLoS One. 2022 Aug 1;17(8):e0269870. doi: 10.1371/journal.pone.0269870 (PMC9342785; doi:10.1371/journal.pone.0269870)
Supplement: S1 Fig — Distribution of terrestrial ecoregions in Mauritania following [1] and border between the Palaearctic and Afrotropic biogeographic realms (dashed green line). (DOCX) [file pone.0269870.s001.docx]

**S1 Figure. Terrestrial Ecoregions**. Distribution of terrestrial ecoregions in Mauritania following [1] and border between the Palaearctic and Afrotropic biogeographic realms (dashed green line).


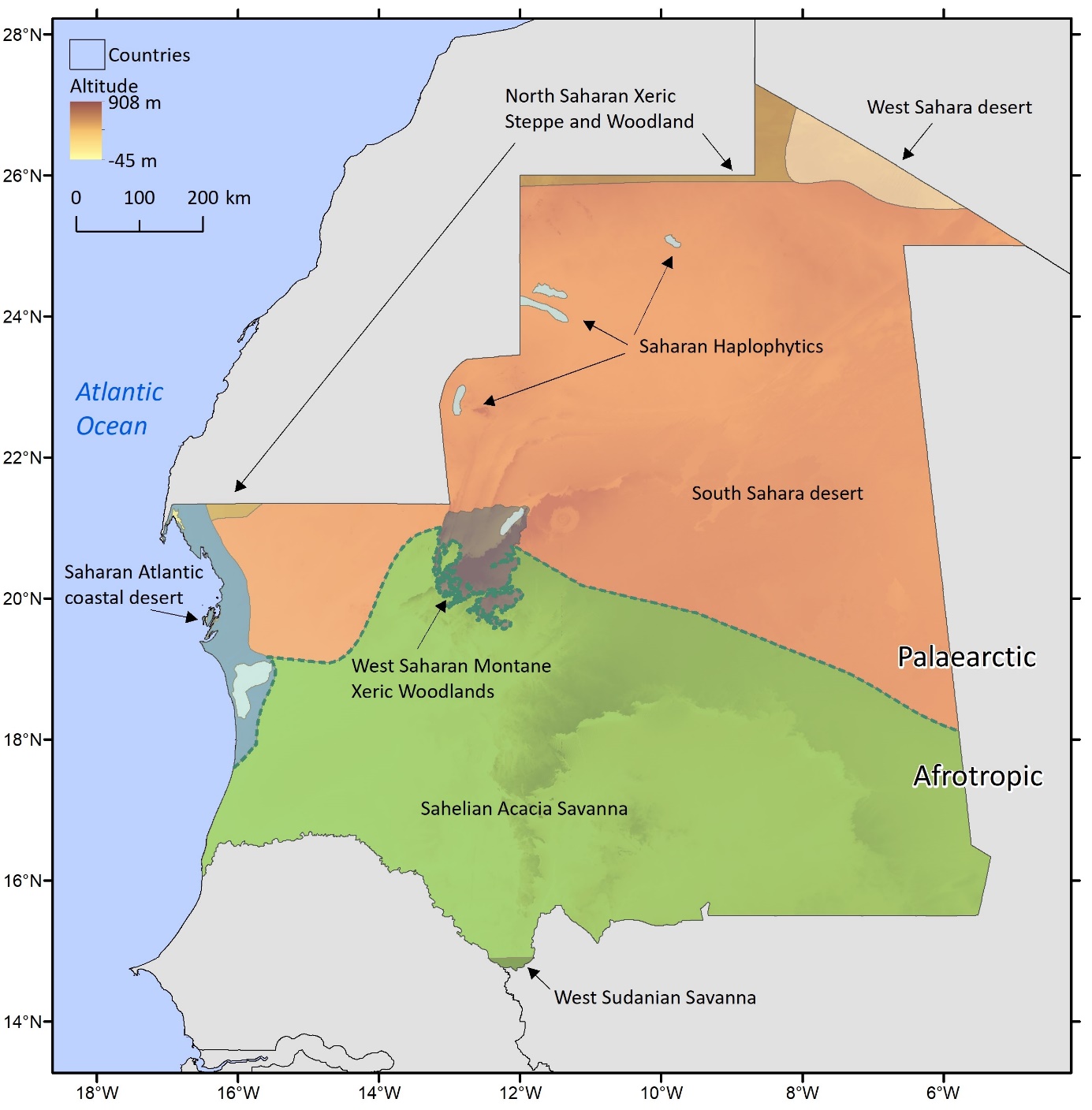


[1] Dinerstein E, Olson D, Joshi A, Vynne C, Burgess ND, Wikramanayake E, et al. An ecoregion-based approach to protecting half the terrestrial realm. BioScience. 2017; 67: 534–545. Available from: https://ecoregions2017.appspot.com/.
