# Supplementary material for: Diversity, distribution and conservation of land mammals in Mauritania, North-West Africa
Source: PLoS One. 2022 Aug 1;17(8):e0269870. doi: 10.1371/journal.pone.0269870 (PMC9342785; doi:10.1371/journal.pone.0269870)
Supplement: S2 Fig — Distribution of populated places [1], mining for exploitation of natural resources [updated from 2], paved (red line) and unpaved (grey line) roads [updated from 3], railways (black line) [updated from 2], human footprint [4], human influence index [5], global accessibility to cities [6], and Last of the Wild [5]. (DOCX) [file pone.0269870.s002.docx]

**S2 Figure. Human activities**. Distribution of populated places [1], mining for exploitation of natural resources [updated from 2], paved (red line) and unpaved (grey line) roads [updated from 3], railways (black line) [updated from 2], human footprint [4], human influence index [5], global accessibility to cities [6], and Last of the Wild [5].


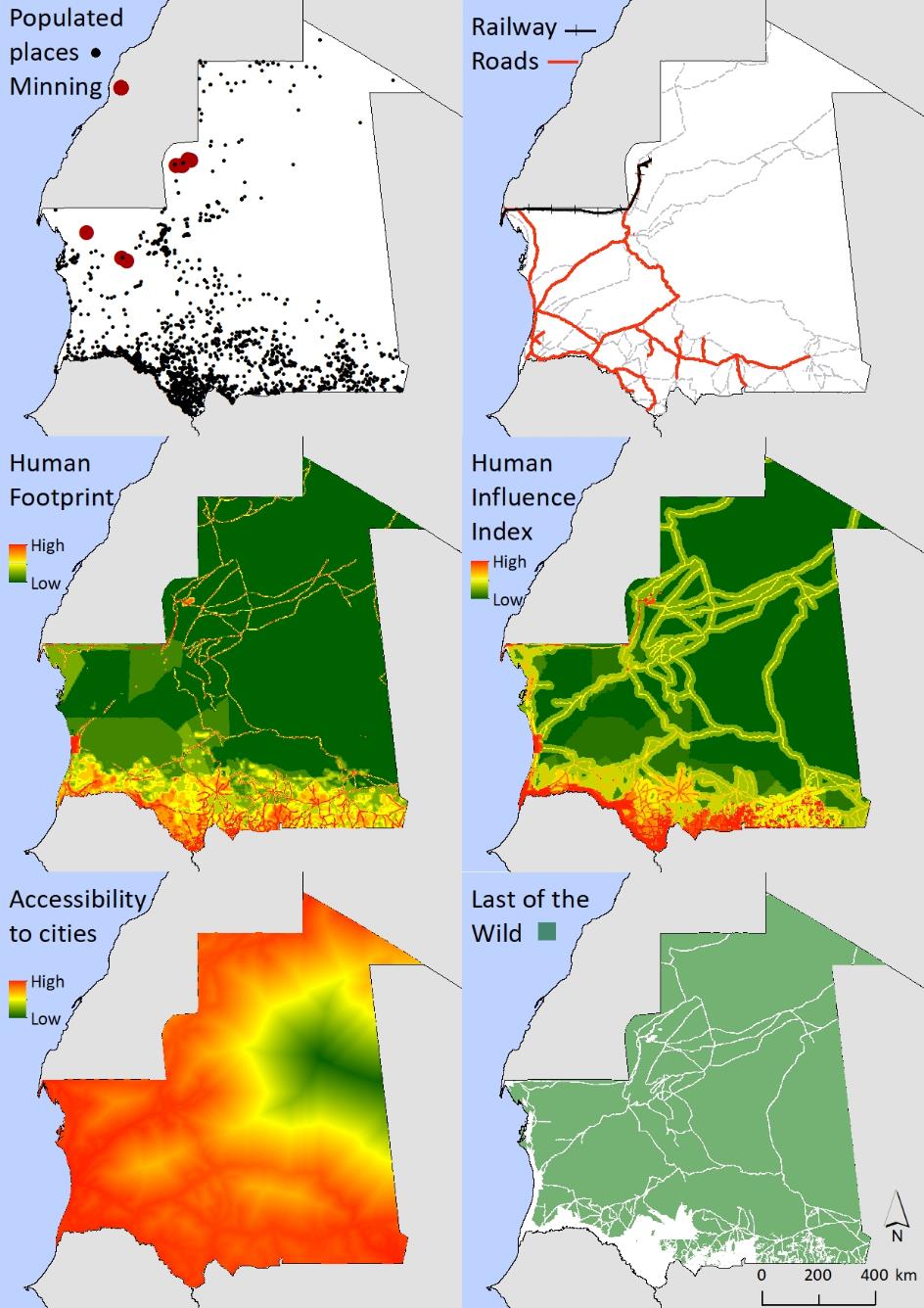


[1] NGA. National Geospatial-Intelligence Agency GEOnet Names Server (GNS). 2016. Available from: http://geonames.nga.mil/gns/html

[2] NIMA. Vector Map (VMap) Level 0. National Imagery and Mapping Agency's (NIMA). 1997. Available from: http://earth-info.nga.mil/publications/vmap0.html

[3] CIESIN-ITOS. Global Roads Open Access Data Set, Version 1 (gROADSv1). Center for International Earth Science Information Network - CIESIN - Columbia University, and Information Technology Outreach Services - ITOS - University of Georgia. 2013. Available from: Palisades, NY: NASA Socioeconomic Data and Applications Center (SEDAC). DOI: 10.7927/H4VD6WCT

[4] Venter O, Sanderson EW, Magrach A, Allan JR, Beher J, Jones KR, et al. Last of the Wild Project, Version 3 (LWP-3): 2009 Human Footprint, 2018 Release. 2018. Available from: Palisades, NY: NASA Socioeconomic Data and Applications Center (SEDAC). DOI: 10.7927/H46T0JQ4

[5] WCS-CIESIN. Last of the Wild Project, Version 2, 2005 (LWP-2): Global Human Footprint Dataset (Geographic). Wildlife Conservation Society (WCS), and Center for International Earth Science Information Network (CIESIN), Columbia University. 2005. Available from: NASA Socioeconomic Data and Applications Center (SEDAC), Palisades. DOI: 10.7927/H4M61H5F

[6] Weiss DJ, Nelson A, Gibson HS, Temperley W, Peedell S, Lieber A, et al. A global map of travel time to cities to assess inequalities in accessibility in 2015. Nature. 2018; 553: 333-336. Available from: https://malariaatlas.org/researchproject/accessibility_to_cities/
