# Supplementary material for: Diversity, distribution and conservation of land mammals in Mauritania, North-West Africa
Source: PLoS One. 2022 Aug 1;17(8):e0269870. doi: 10.1371/journal.pone.0269870 (PMC9342785; doi:10.1371/journal.pone.0269870)
Supplement: S3 Fig — Hydrographic network (including the Senegal River) [1], location of major seasonal wetlands and mountain rock pools (Guelta) [2], and digital elevation model of Mauritania [3]. (DOCX) [file pone.0269870.s003.docx]

**S3 Figure. Hydrographic network.** Hydrographic network (including the Senegal River) [1], location of major seasonal wetlands and mountain rock pools (*Guelta*) [2], and digital elevation model of Mauritania [3].


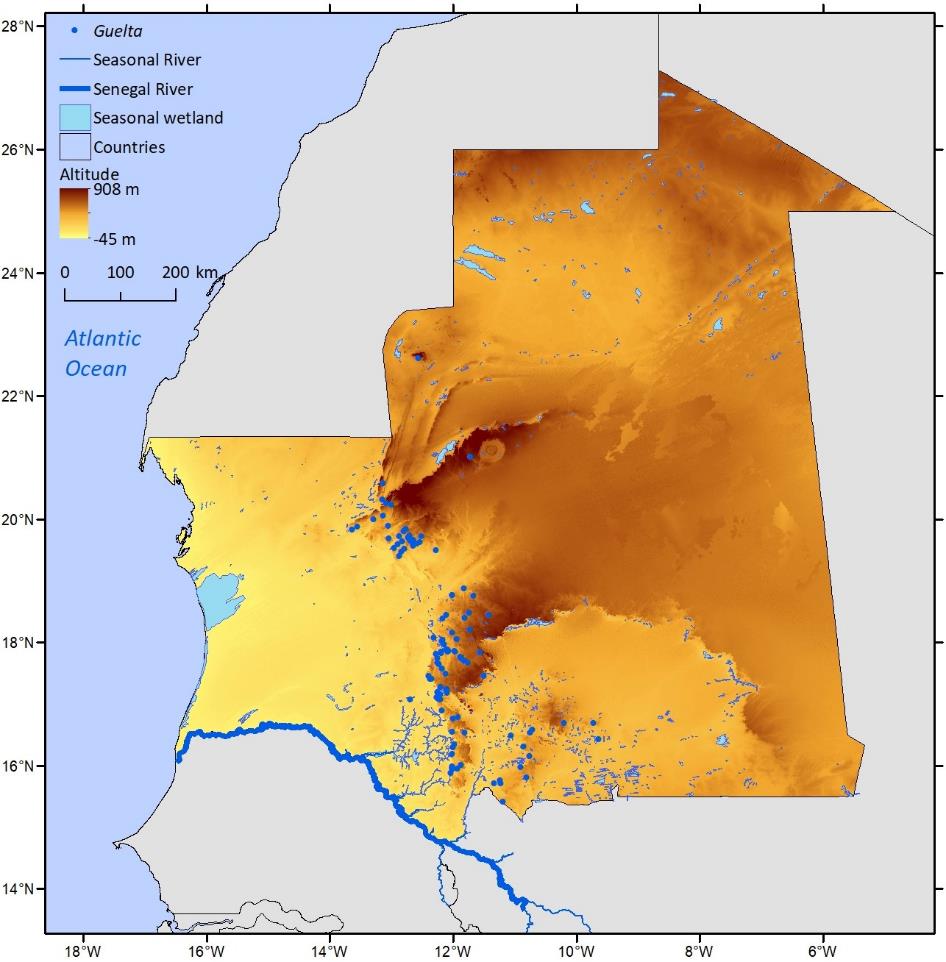


[1] Lehner B, Verdin K, Jarvis A. New global hydrography derived from spaceborne elevation data. EOS Transac. 2008; 89: 93-94. Available from: https://www.hydrosheds.org/

[2] Vale CG, Pimm SL, Brito JC. Overlooked mountain rock pools in deserts are critical local hotspots of biodiversity. PLoS One. 2015; 10: e0118367.

[3] Jarvis, A., Reuter, H.I., Nelson, A., Guevara, E. Hole-filled SRTM for the globe Version 4. CGIAR-CSI SRTM 90m Database. 2008. Available from: http://srtm.csi.cgiar.org.
