# Supplementary material for: Diversity, distribution and conservation of land mammals in Mauritania, North-West Africa
Source: PLoS One. 2022 Aug 1;17(8):e0269870. doi: 10.1371/journal.pone.0269870 (PMC9342785; doi:10.1371/journal.pone.0269870)
Supplement: S4 Fig — Distribution of annual mean temperature [1], annual precipitation [1], aridity index [2], and continentality index (average temperature of warmest month—average temperature of coldest month) [3] in Mauritania. (DOCX) [file pone.0269870.s004.docx]

**S4 Figure**. **Climate.** Distribution of annual mean temperature [1], annual precipitation [1], aridity index [2], and continentality index (average temperature of warmest month - average temperature of coldest month) [3] in Mauritania.


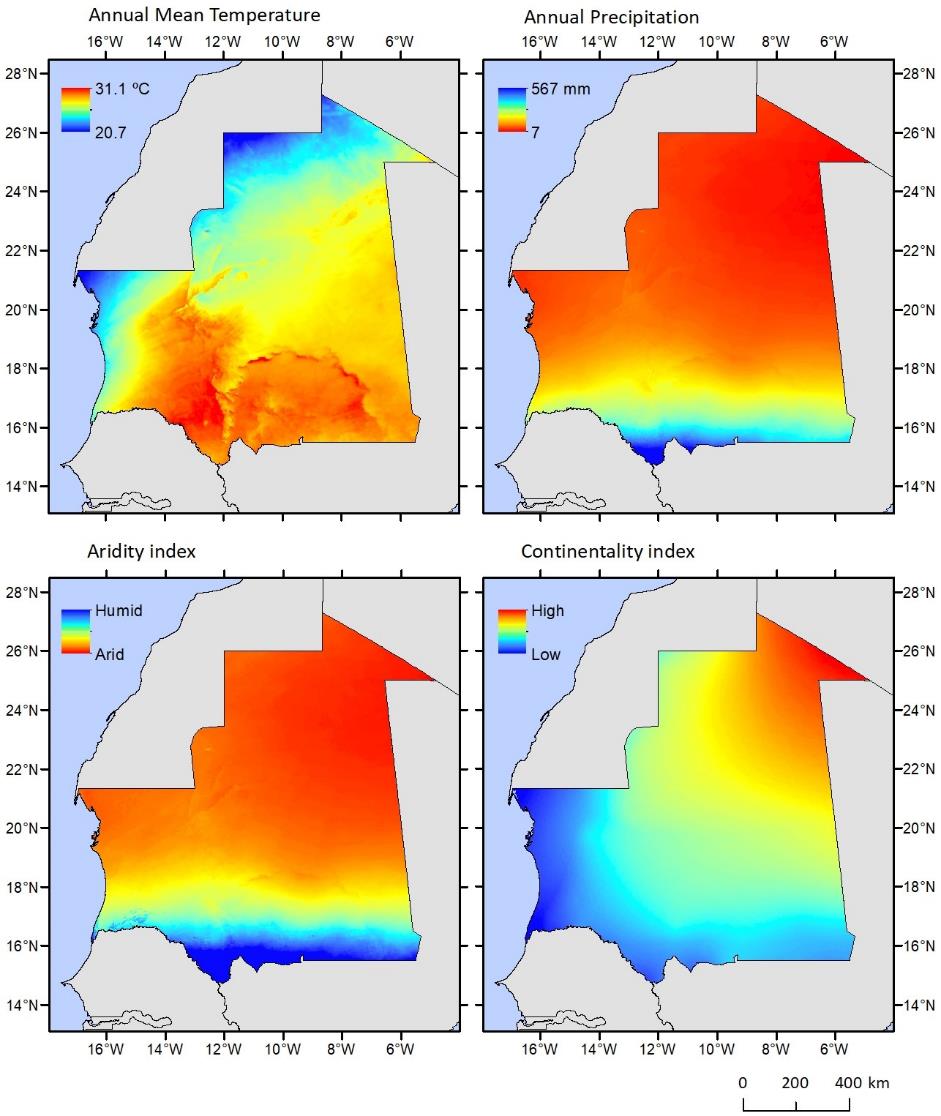


[1] Fick, S.E., Hijmans, R.J. WorldClim 2: new 1km spatial resolution climate surfaces for global land areas. International Journal of Climatology 37: 4302-4315. 2017. Available from: https://www.worldclim.org/.

[2] Trabucco, A., Zomer, R.J. Global Aridity Index and Potential Evapo-Transpiration (ET0) Climate Database v2. CGIAR Consortium for Spatial Information (CGIAR-CSI). 2018. Available from: https://cgiarcsi.community.

[3] Title, P.O., Bemmels, J.B. ENVIREM: ENVIronmental Rasters for Ecological Modeling version 1.0 [Data set]. University of Michigan - Deep Blue. 2018. Available from: https://doi.org/10.7302/Z2BR8Q40.
