# Supplementary material for: Diversity, distribution and conservation of land mammals in Mauritania, North-West Africa
Source: PLoS One. 2022 Aug 1;17(8):e0269870. doi: 10.1371/journal.pone.0269870 (PMC9342785; doi:10.1371/journal.pone.0269870)
Supplement: S5 Fig — Distribution of major land-cover categories [1] in Mauritania. The main sand dune areas (underlined) and sandy gravel plains (italics) are identified. (DOCX) [file pone.0269870.s005.docx]

**S5 Figure. Land-cover.** Distribution of major land-cover categories [1] in Mauritania. The main sand dune areas (underlined) and sandy gravel plains (italics) are identified.


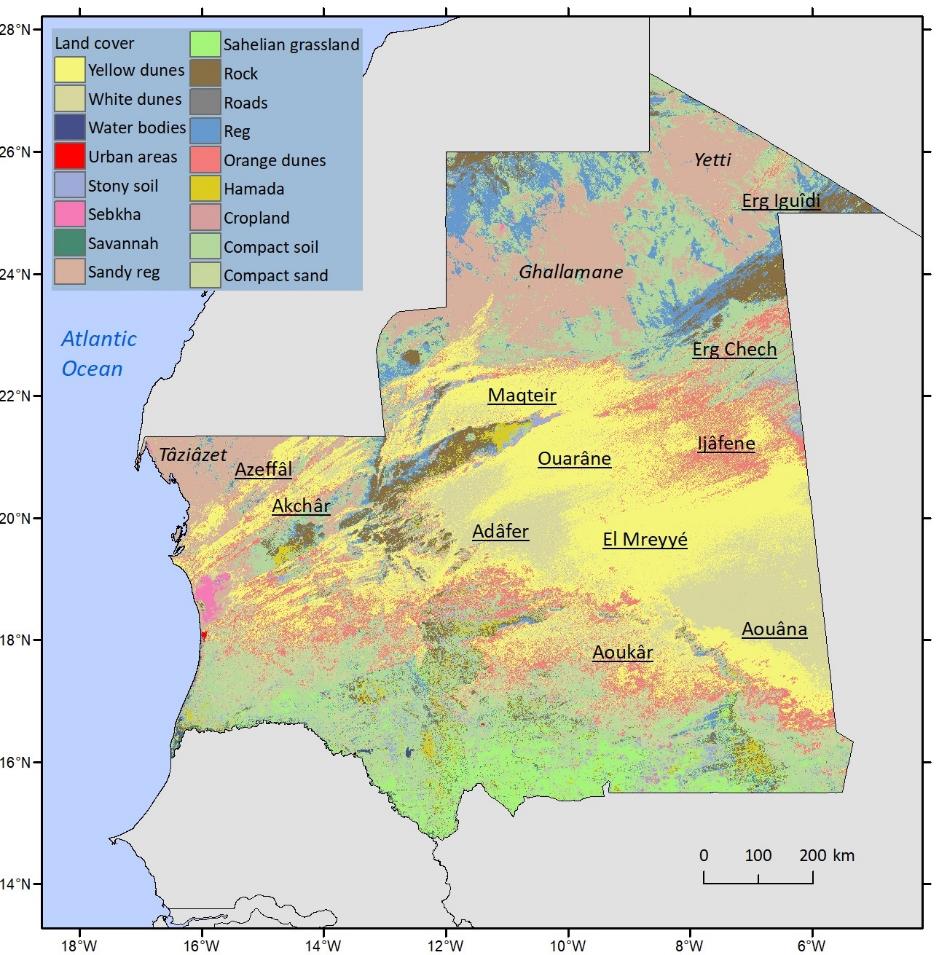


[1] Campos, J.C., Brito, J.C. Mapping underrepresented land cover heterogeneity in arid regions: the Sahara-Sahel example. ISPRS Journal of Photogrammetry and Remote Sensing. 2018; 146: 211-220. Available from: https://drive.google.com/open?id=1cr4q554d5mTWu4SLvfTEQ5EXdgFzc78w
