# Supplementary material for: Diversity, distribution and conservation of land mammals in Mauritania, North-West Africa
Source: PLoS One. 2022 Aug 1;17(8):e0269870. doi: 10.1371/journal.pone.0269870 (PMC9342785; doi:10.1371/journal.pone.0269870)
Supplement: S6 Fig — Representative pictures of the major land-cover categories found in Mauritania [1] and of the habitats frequently mentioned throughout the texts. Codes of the localities of pictures are mapped. (DOCX) [file pone.0269870.s006.docx]

**S6 Figure. Habitats.** Representative pictures of the major land-cover categories found in Mauritania [1] and of the habitats frequently mentioned throughout the texts. Codes of the localities of pictures are mapped.


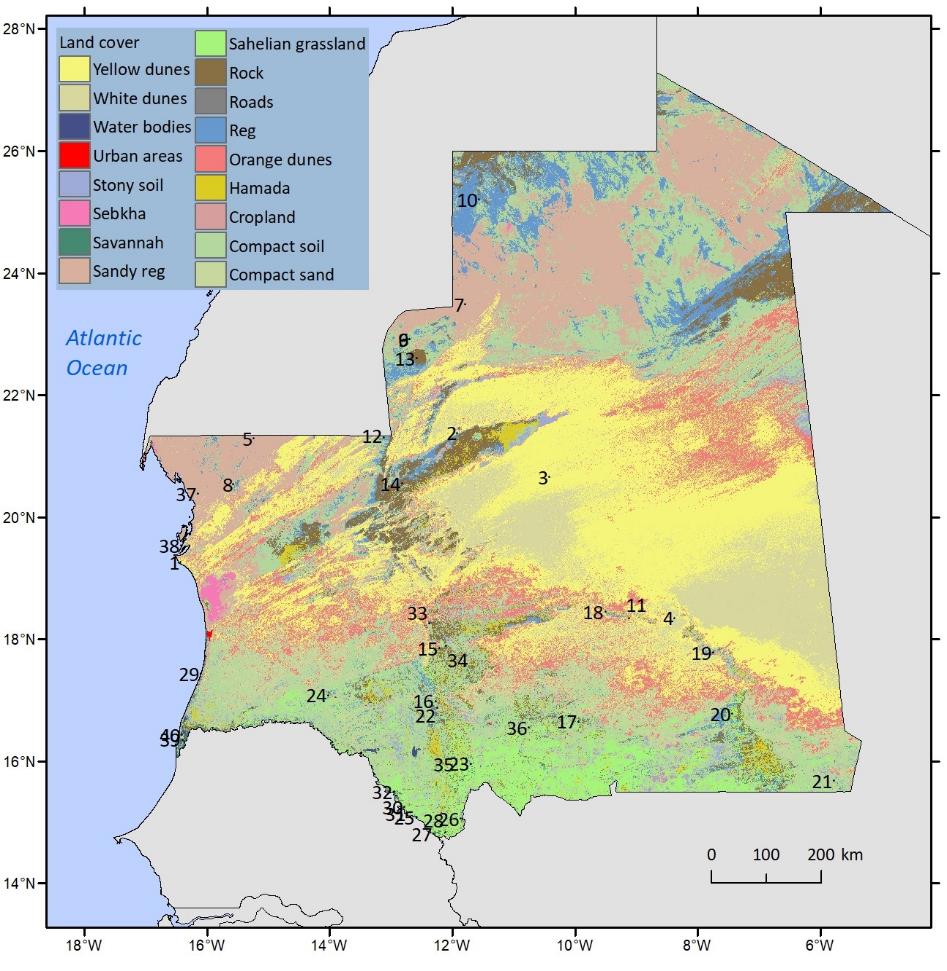


[1] Campos, J.C., Brito, J.C. Mapping underrepresented land cover heterogeneity in arid regions: the Sahara-Sahel example. ISPRS Journal of Photogrammetry and Remote Sensing. 2018; 146: 211-220. Available from: https://drive.google.com/open?id=1cr4q554d5mTWu4SLvfTEQ5EXdgFzc78w


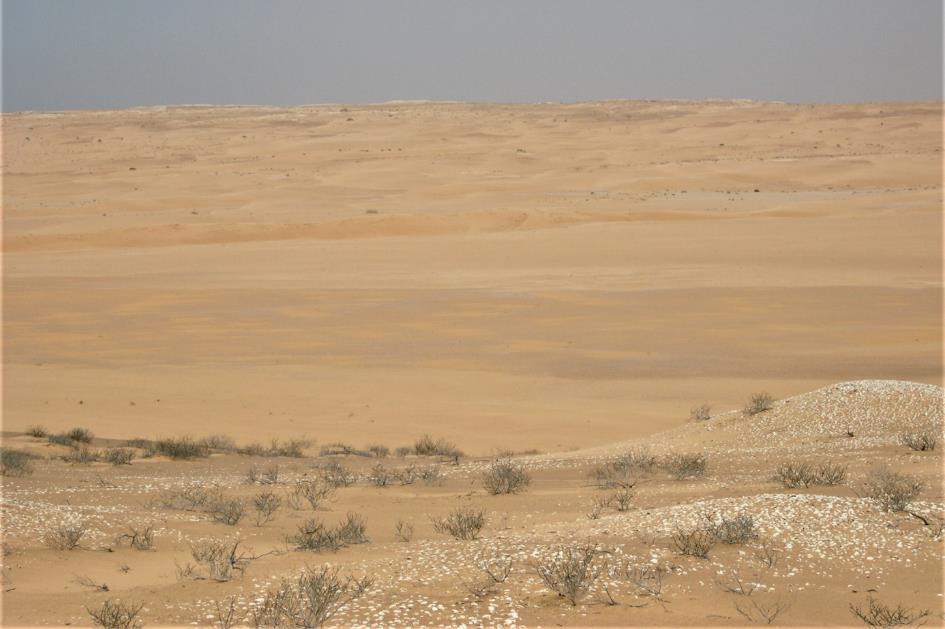


01 - Sand dunes (Erg Akchâr in coastal area about 15 km south of Nouâmghâr, Province of Inchiri; November 2004)


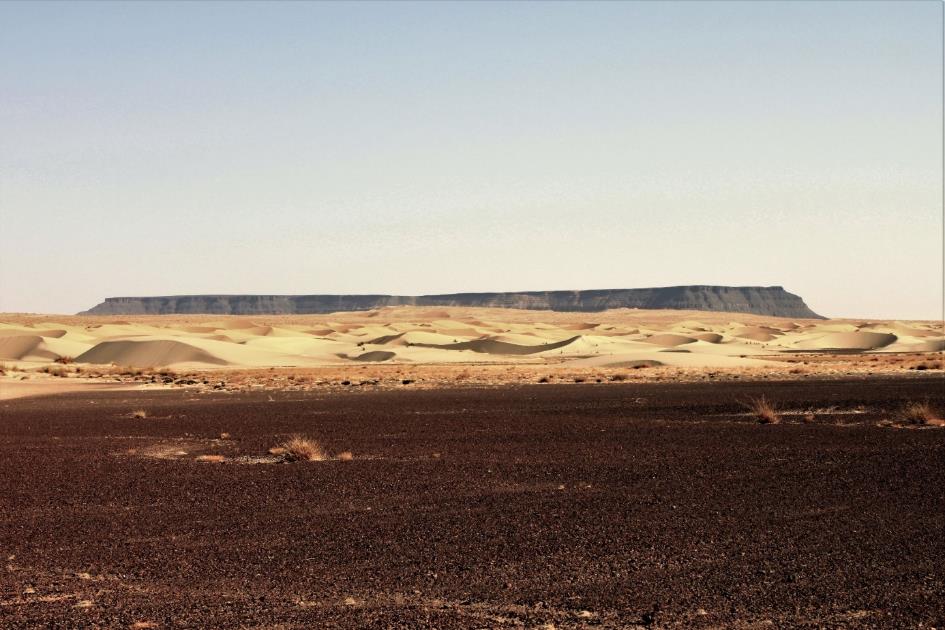


02 - Sand dunes in background (extreme southern limit of Erg Maqteïr, Province of Tiris-Zemmour; October 2011)


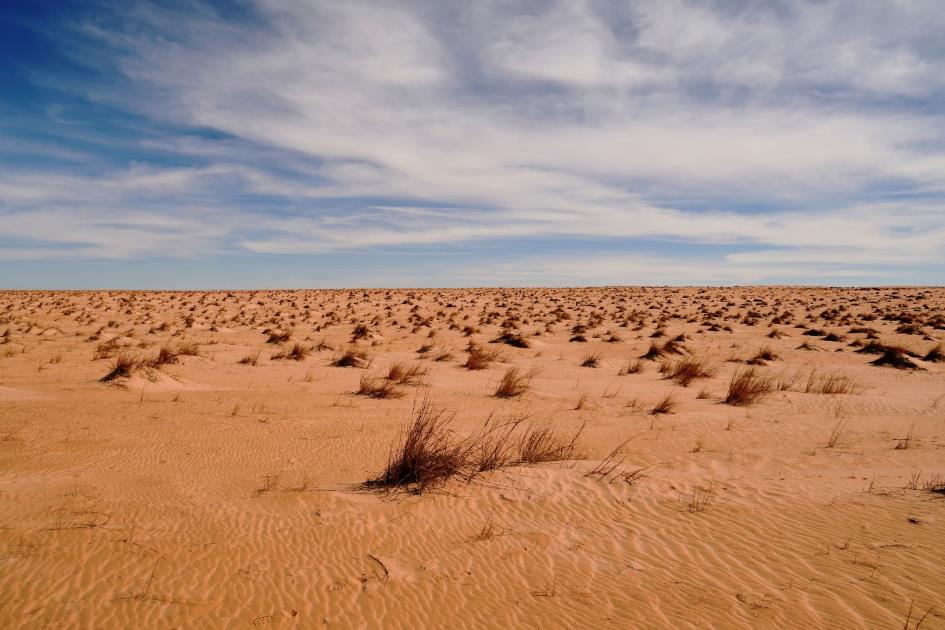


03 - Sand dunes (Erg Ouarâne about 130 km south-east of Ouadâne, Province of Adrar; November 2019)


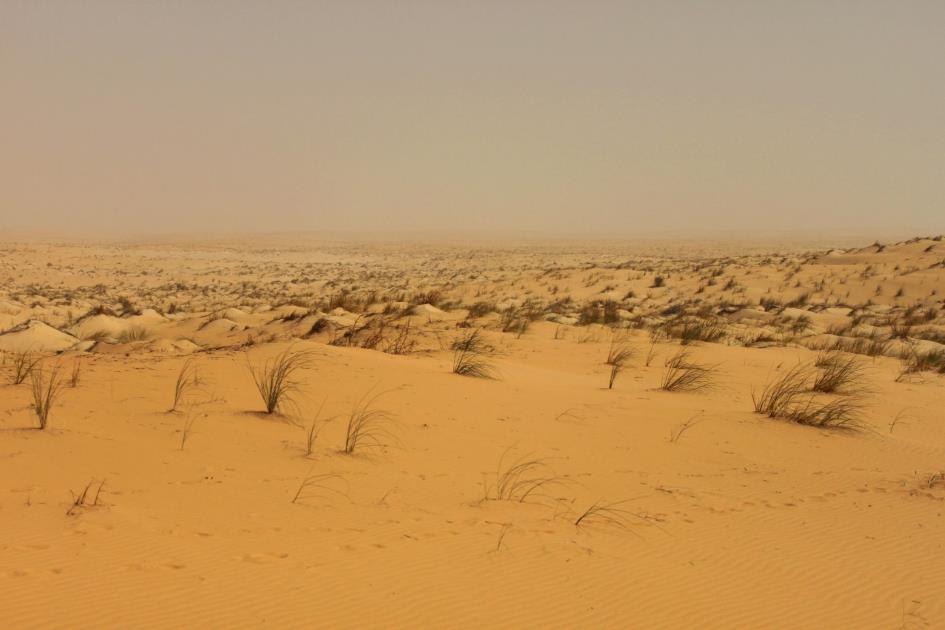


04 - Sand dunes (Aklé Aoukâr about 150 km east of Tîchît, Province of Hodh Ech Chargui; January 2014)


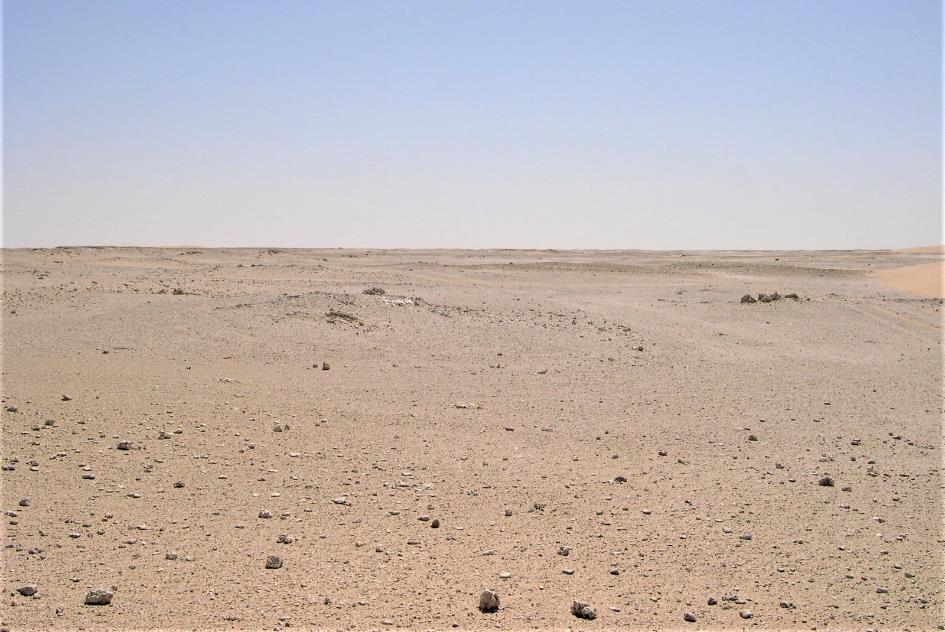


05 - Gravel and sand floodplains (About 100 km east of Nouâdhibou, Province of Dakhlet-Nouâdhibou; November 2003)


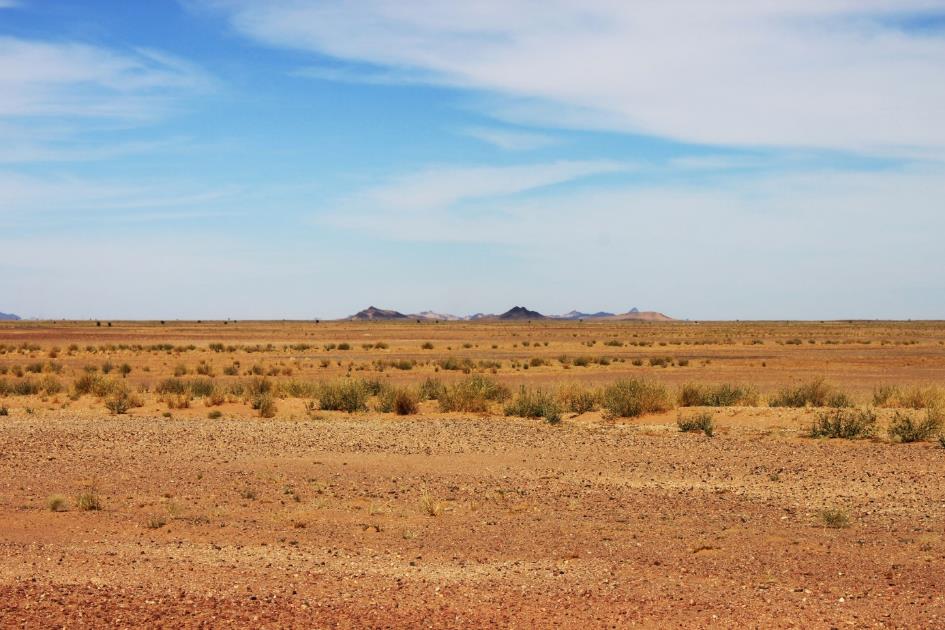


06 - Gravel and sand floodplains (About 30 km north of F’dérik, Province of Tiris-Zemmour; April 2017)


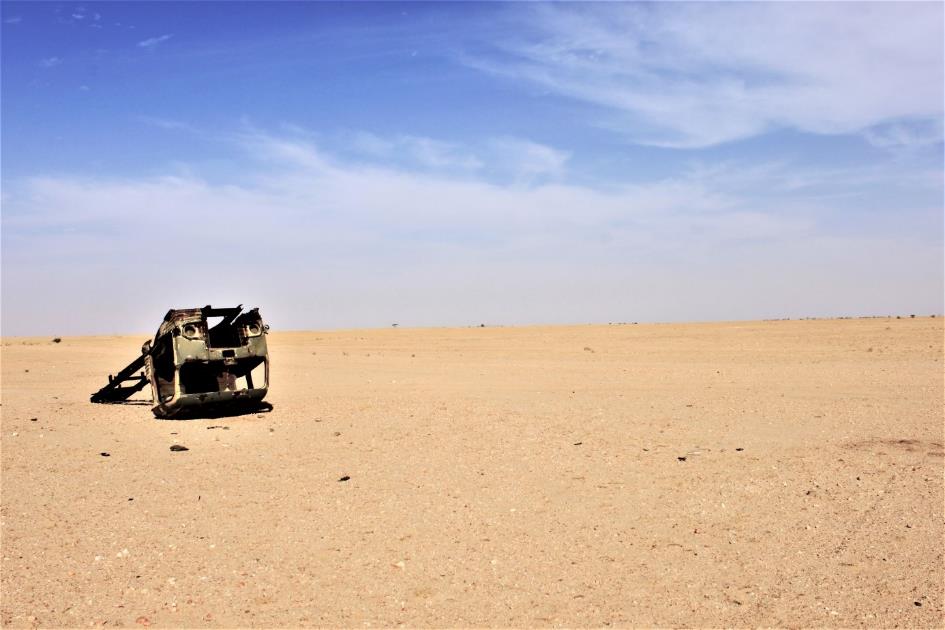


07 - Gravel and sand floodplains (About 130 km north-east of F’dérik, Province of Tiris-Zemmour; April 2017)


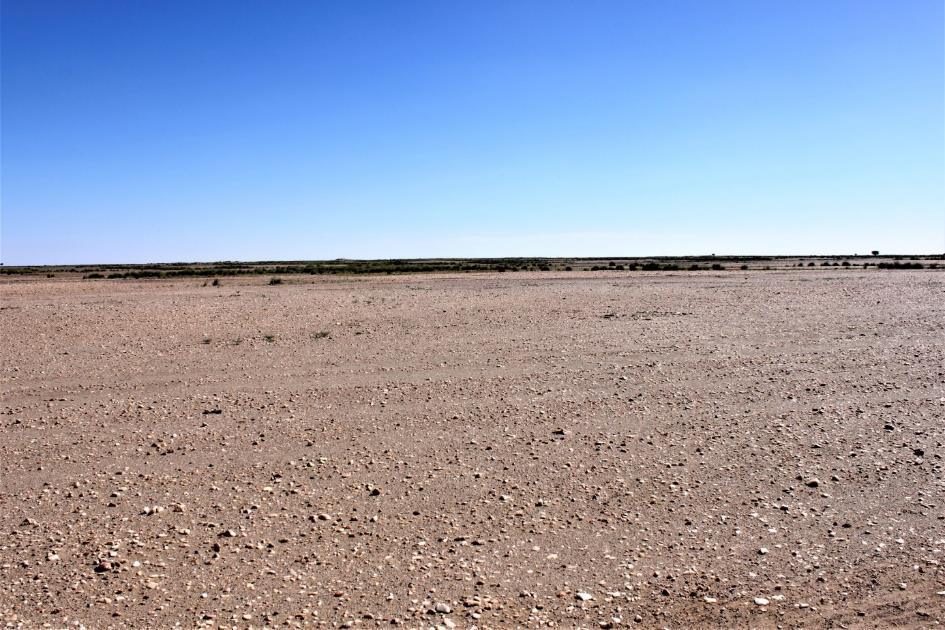


08 - Gravel and sand floodplains (Near Tasiast, Province of Inchiri; December 2010)


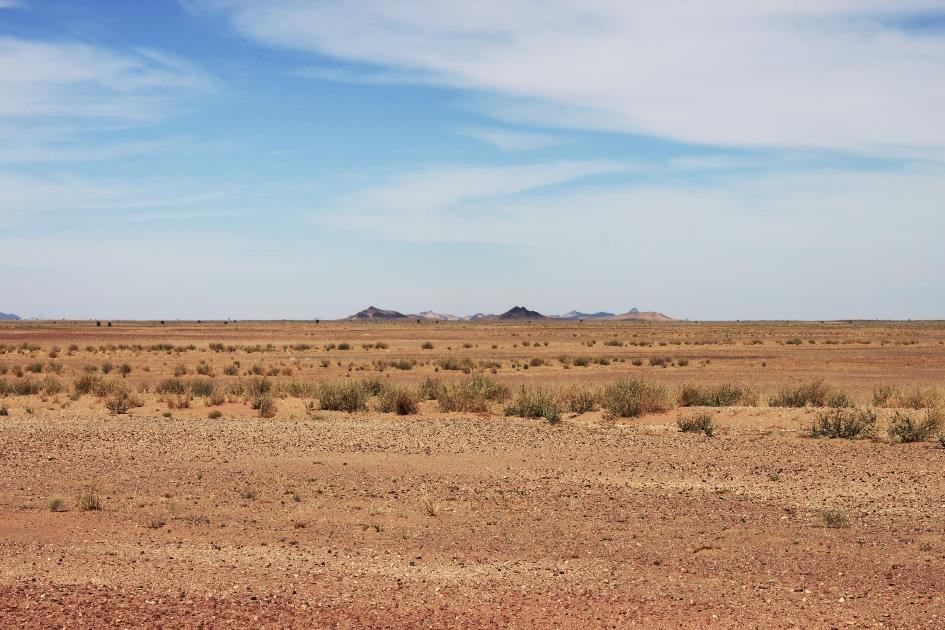


09 - Compact soil (About 25 km north of Fdérik with Gleib el Ouatât in the background, Province of Tiris-Zemmour; April 2017)


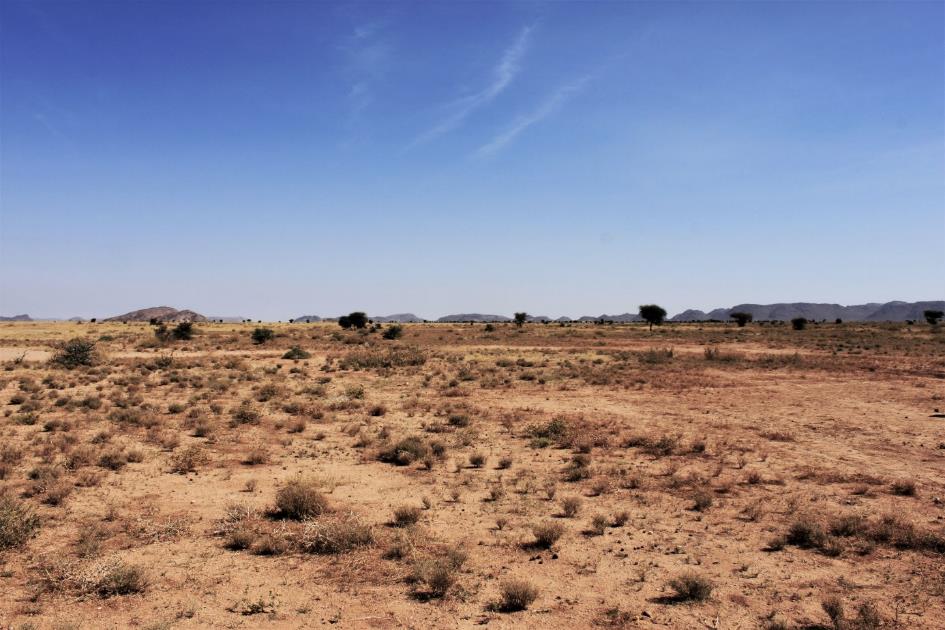


10 - Compact soil (Bir Moghrein, Province of Tiris-Zemmour; April 2017)


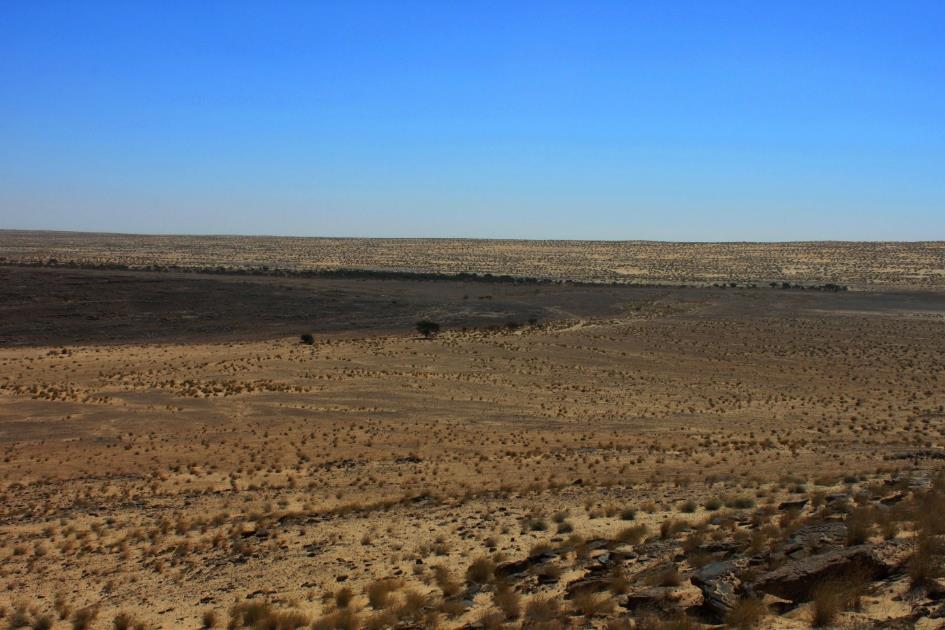


11 - Compact soil (El Rhimiya, about 45 km east of Tîchît, Province of Tagant; February 2014)


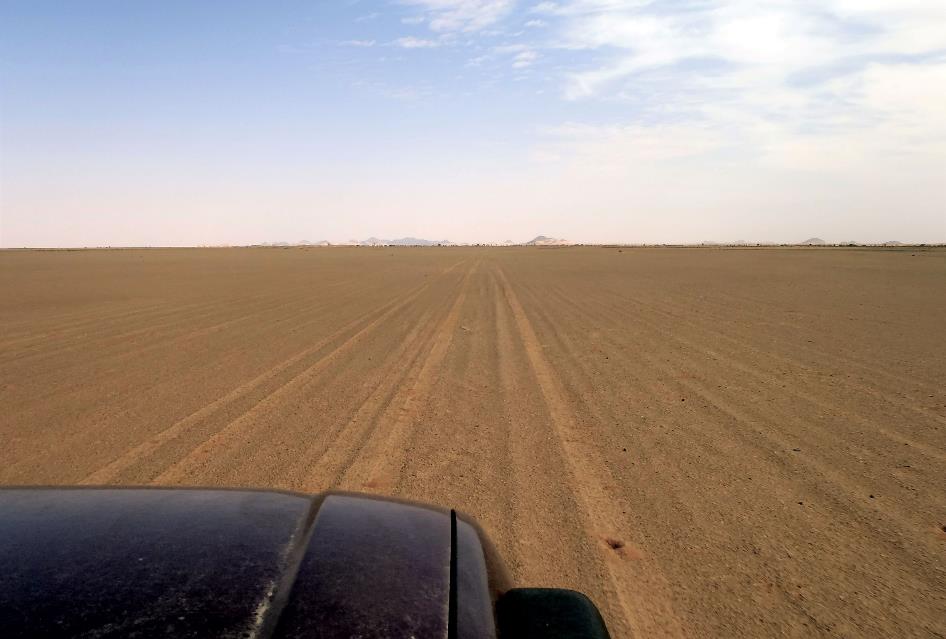


12 - Compact soil (Near Choum, Province of Adrar; November 2011)


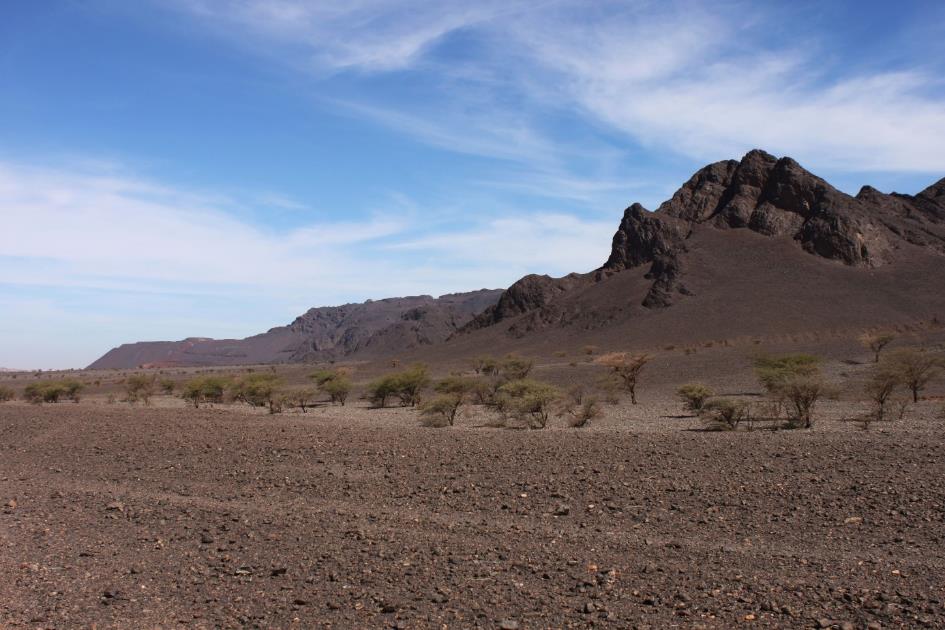


13 - Bare rock and rocky soil (Kediet ej Jill massif, southern slope, Province of Tiris-Zemmour; April 2017)


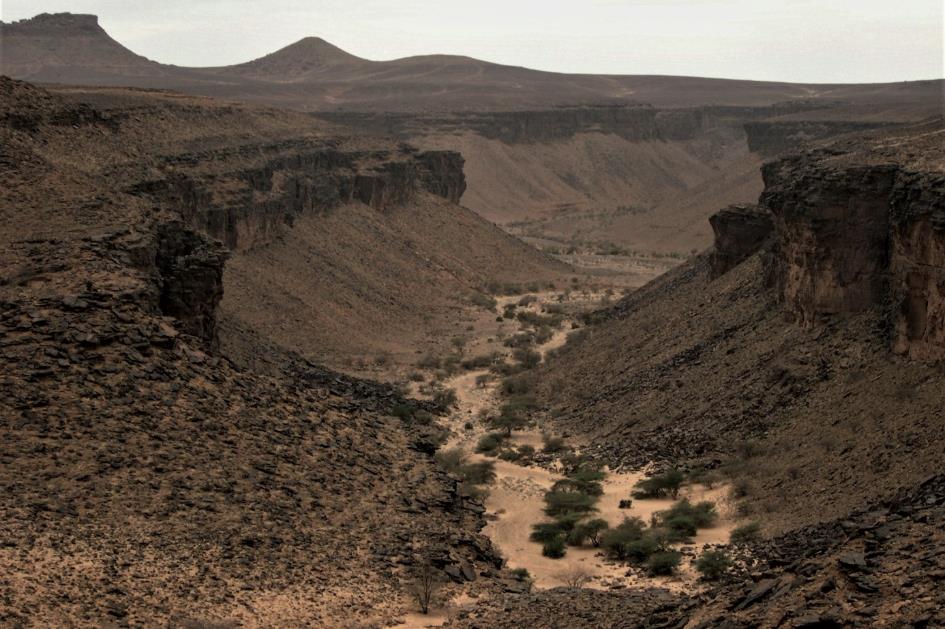


14 - Bare rock and rocky soil (Adrar Atar plateau, Oued Amogjâr Pass, Province of Adrar; November 2004)


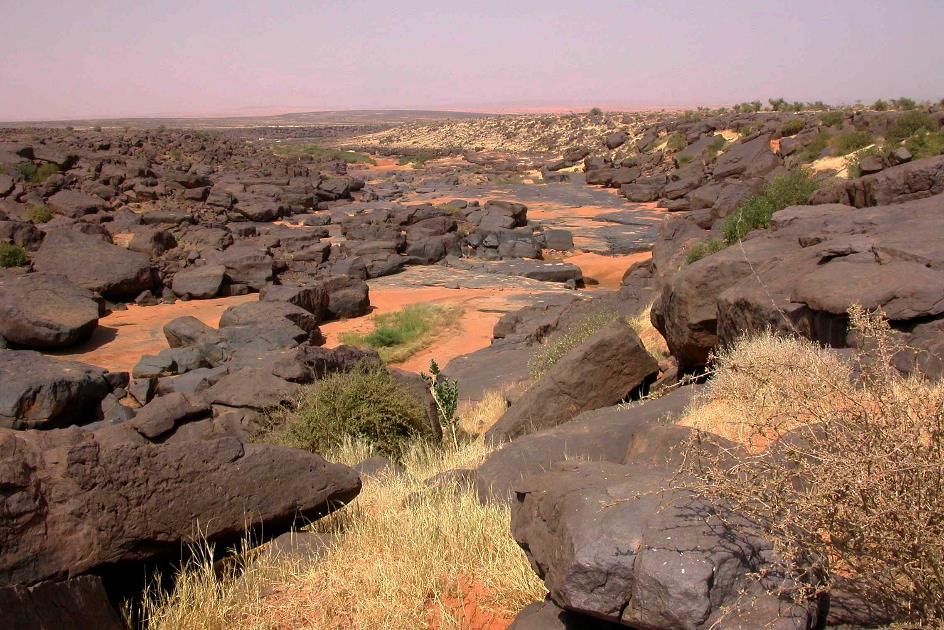


15 - Bare rock and rocky soil (Tagant plateau, Oued Bourâgga, Province of Tagant; November 2008)


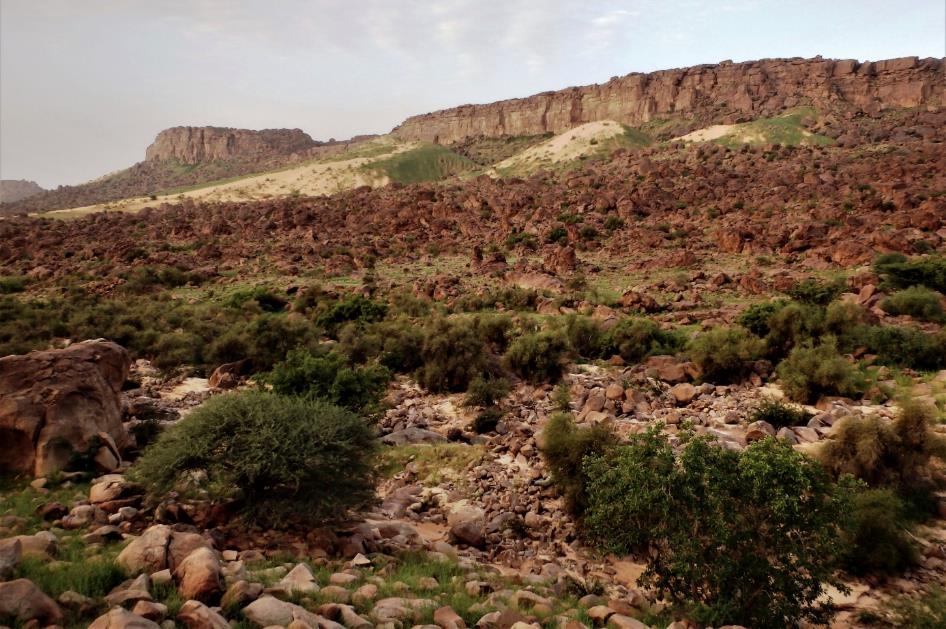


16 - Bare rock and rocky soil (Assaba plateau, near Greinat el Atrous, Province of Assaba; September 2015)


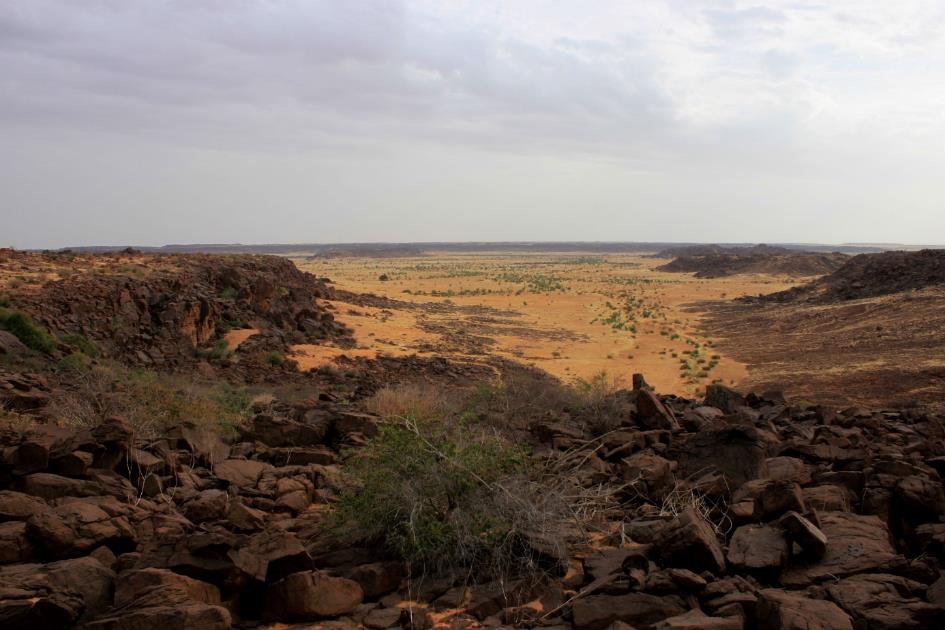


17 - Bare rock and rocky soil (Afollé plateau, near Dmouch Telli, Province of Hodh El Gharbi; November 2014)


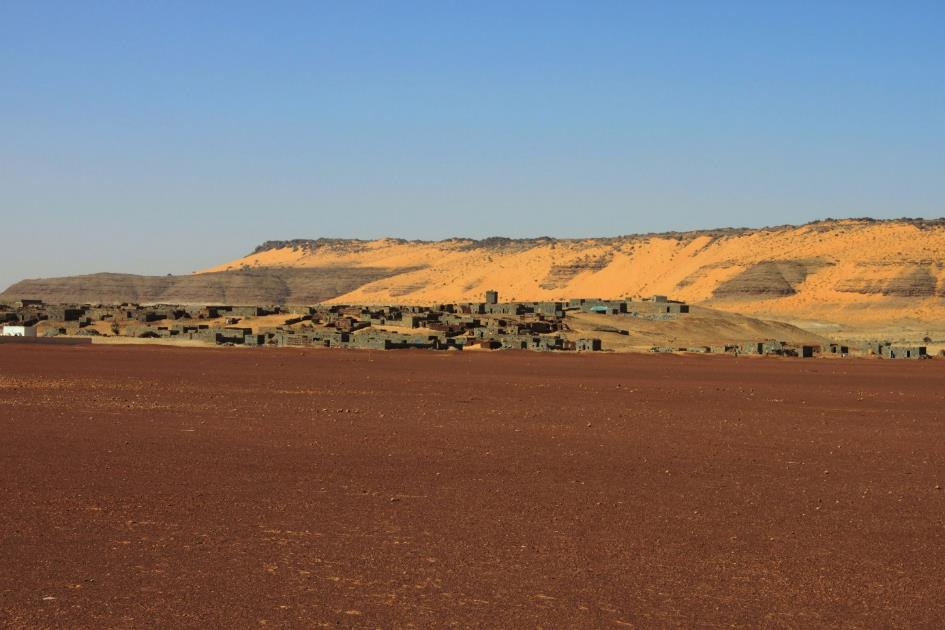


18 - Bare rock and rocky soil in the background (Dhar Tîchît and the village of Tîchît, Province of Tagant; February 2014)


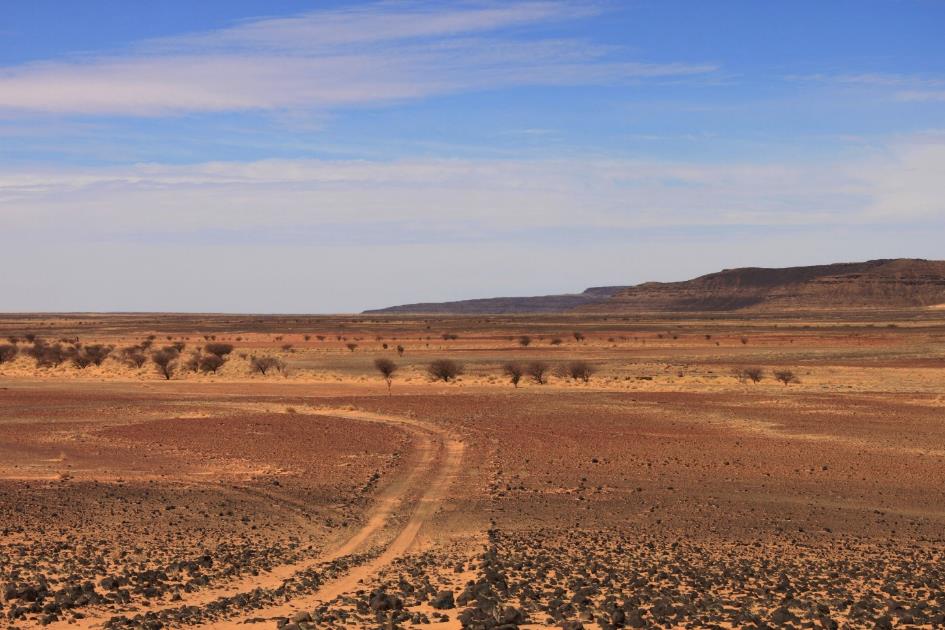


19 - Bare rock and rocky soil (Dhar Oualâta at a distance, near Sgué wells, Province of Hodh Ech Chargui; January 2014)


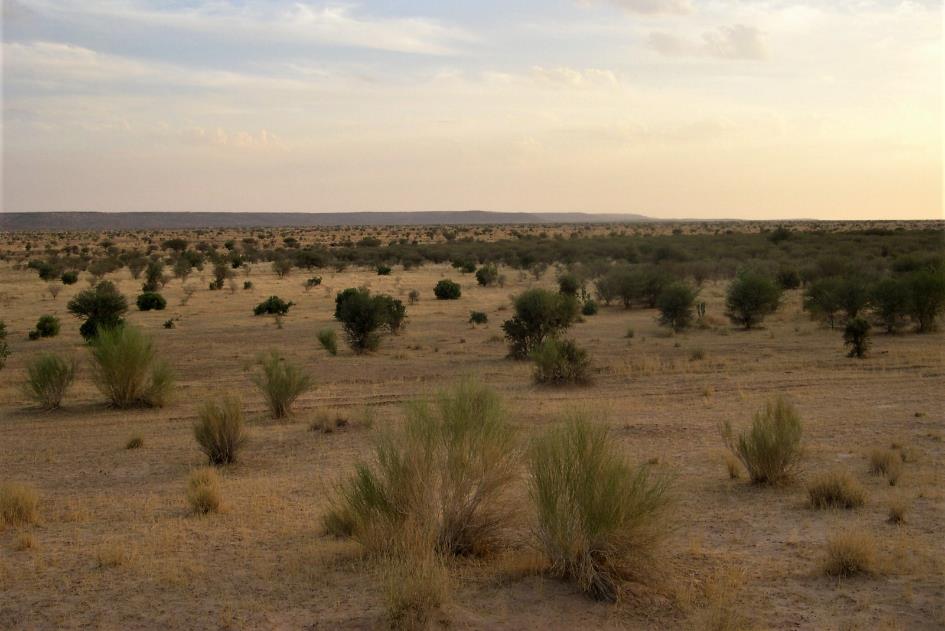


20 - Bare rock and rocky soil in the background (Dhar Néma at a distance, about 5 km north-west of Néma, Province of Hodh Ech Chargui; November 2003)


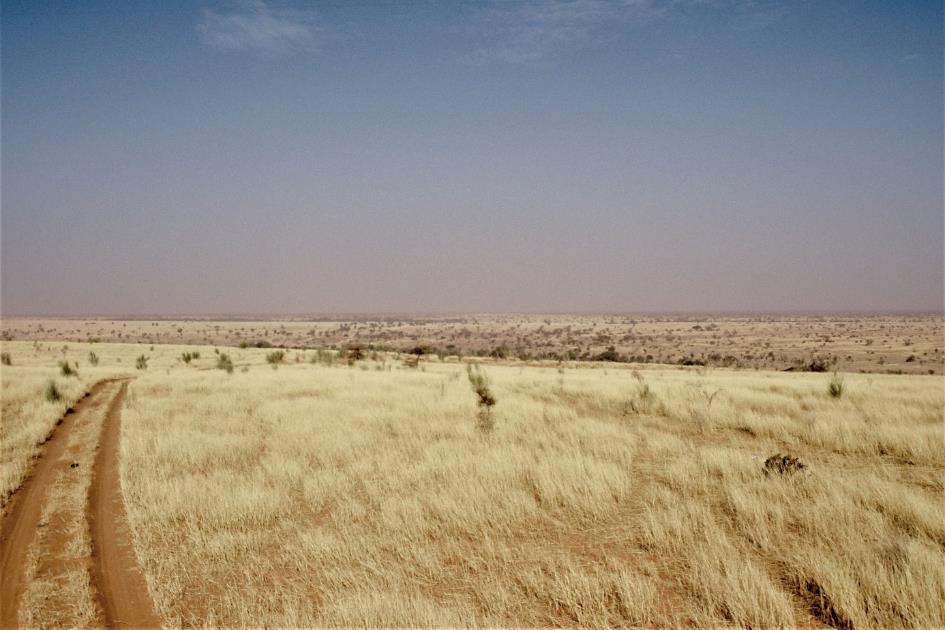


21 - Grasslands (About 10 km south-east of Bassikounou, Province of Hodh Ech Chargui; March 2007)


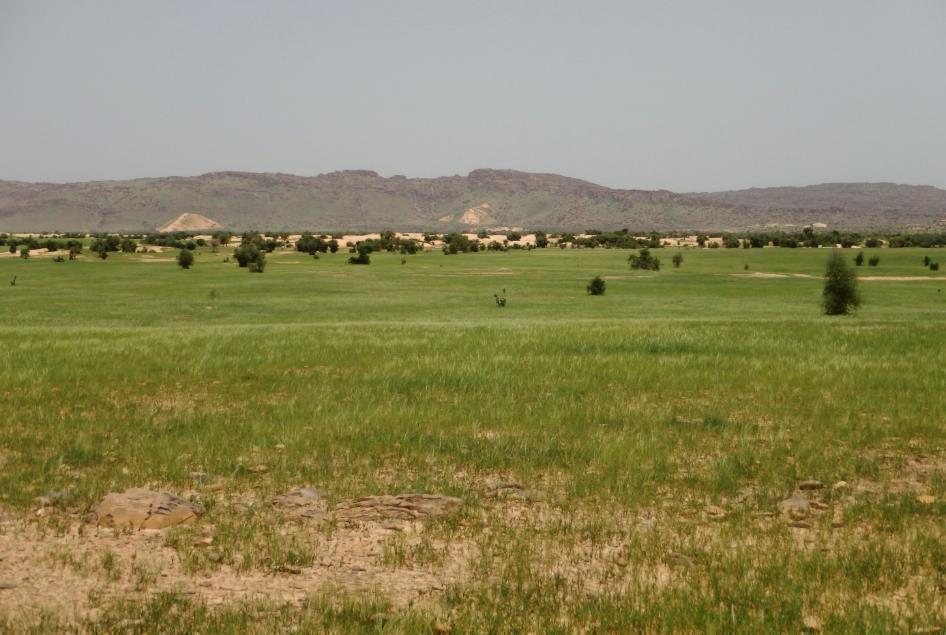


22 - Grasslands (About 50 km south of El Ghaira, Province of Assaba; September 2015)


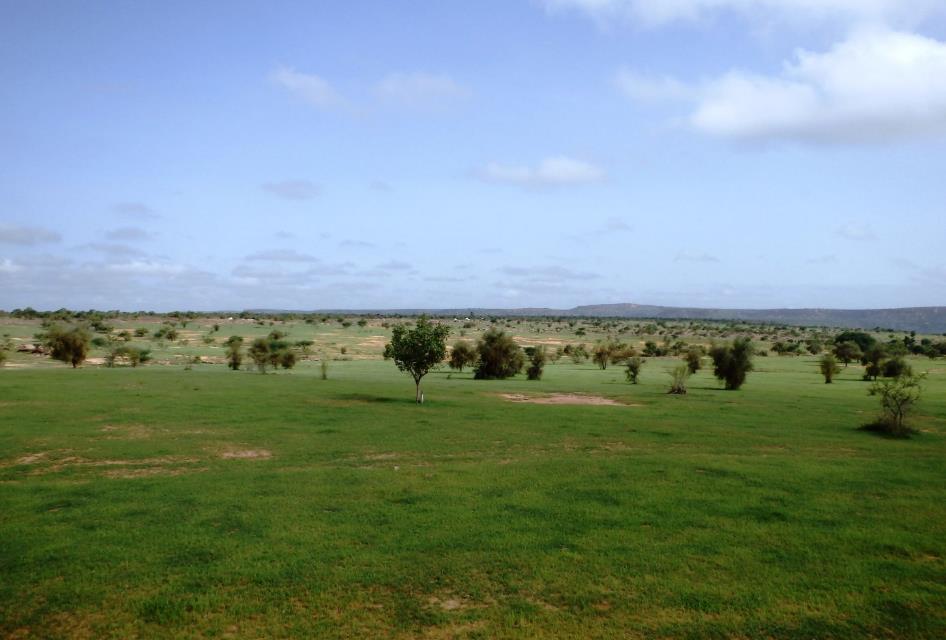


23 - Grasslands (About 5 km east of Soufa Pass, Province of Guidimaka; September 2015)


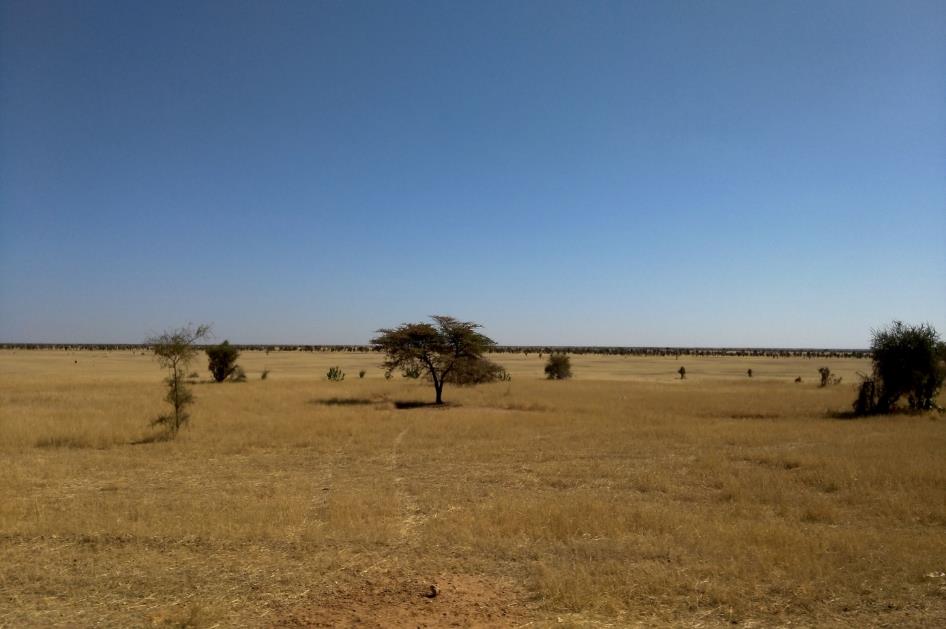


24 - Grasslands (Near Aleg, Province of Brakna; January 2016)


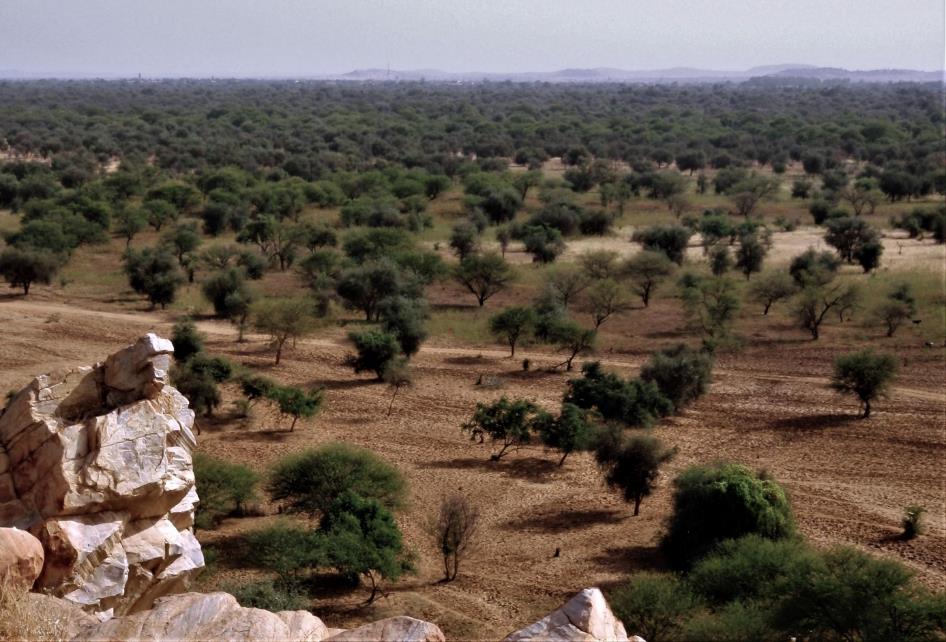


25 - Savannah (Moudéri, Province of Guidimaka; November 2012)


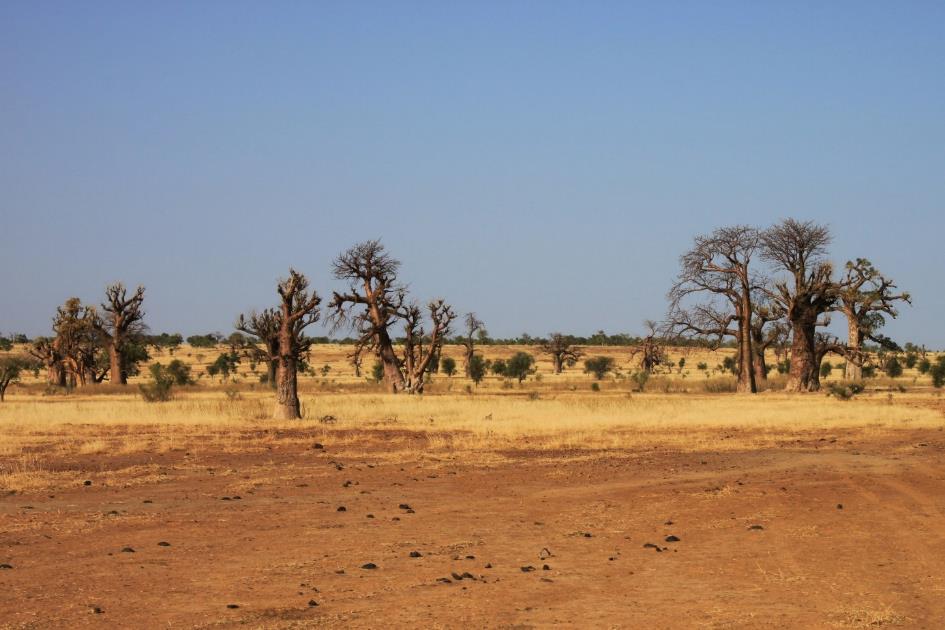


26 - Savannah (Baediam, Province of Guidimaka; November 2014)


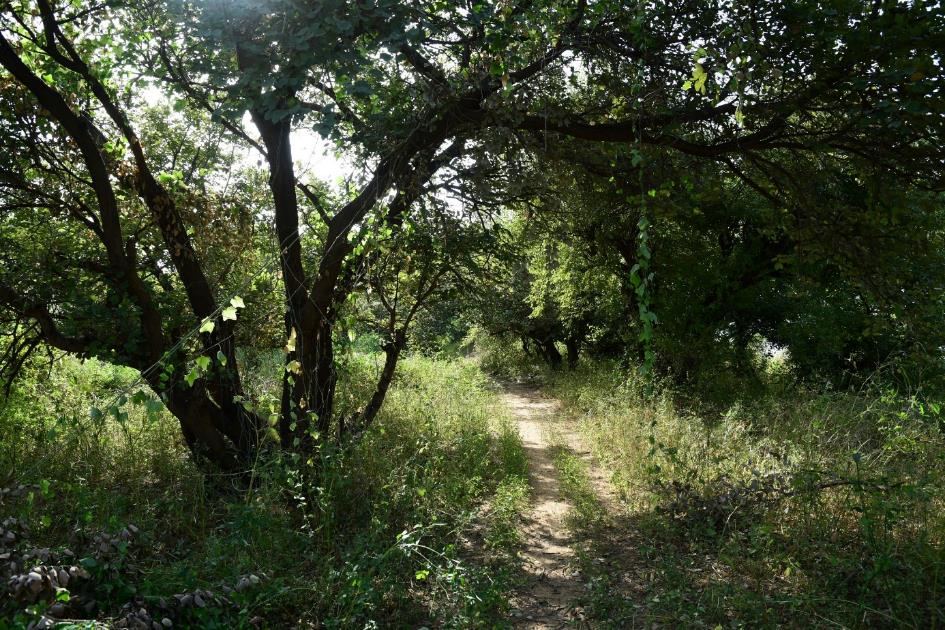


27 - Savannah (About 5 km south-east of Moulessimou, Province of Guidimaka; November 2020)


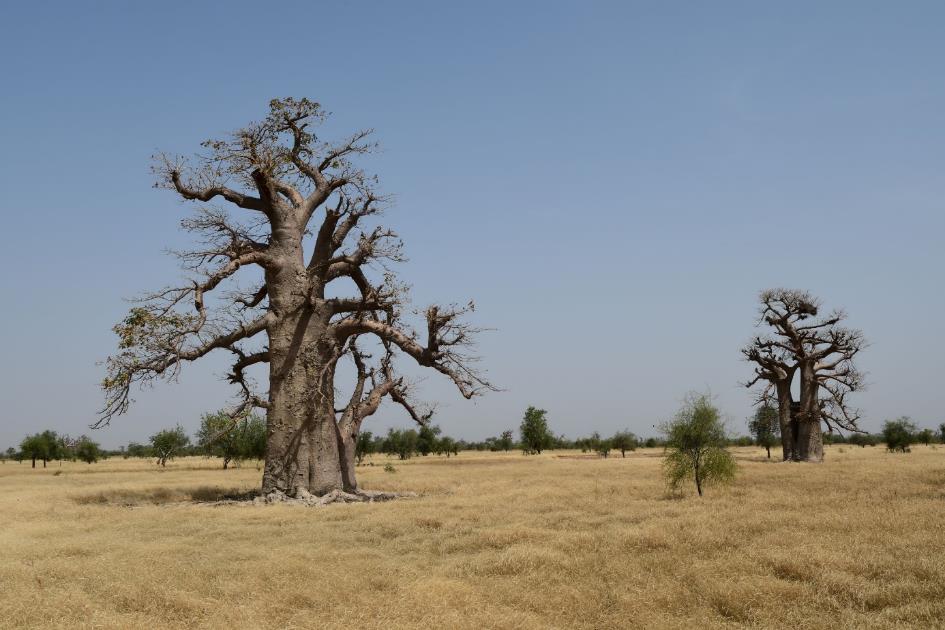


28 - Savannah (About 10 km north of Khabou-Guidimaka, Province of Guidimaka; November 2020)


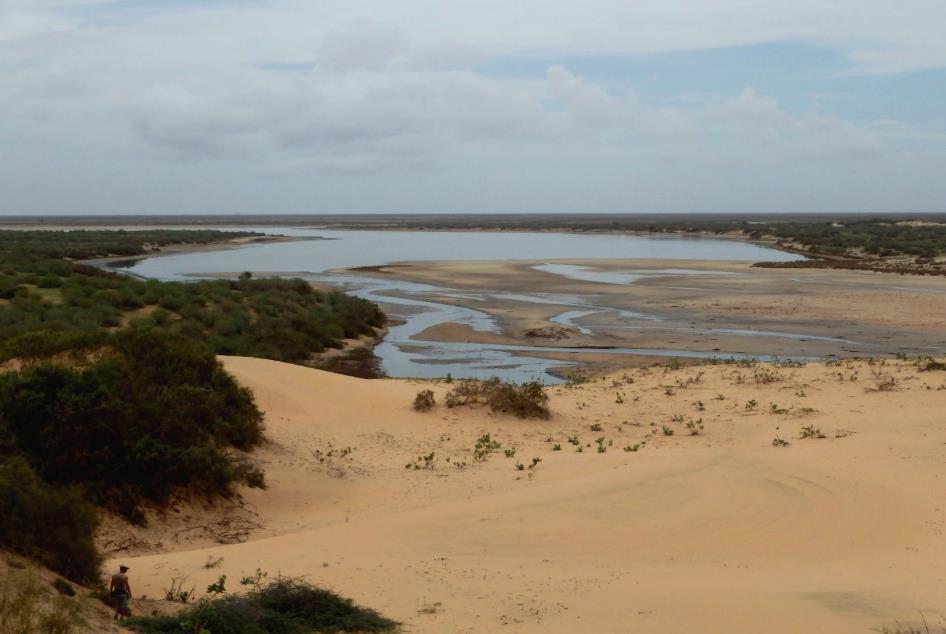


29 - Coastal area (Chott Boul, Province of Trarza; August 2015)


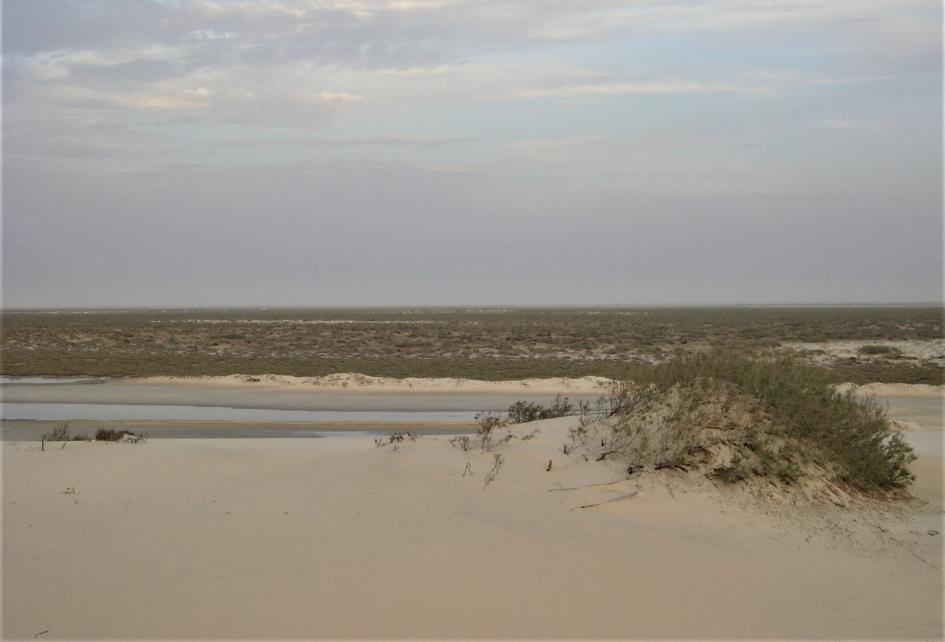


30 - Coastal area (Tâmzakt, Province of Trarza; November 2004)


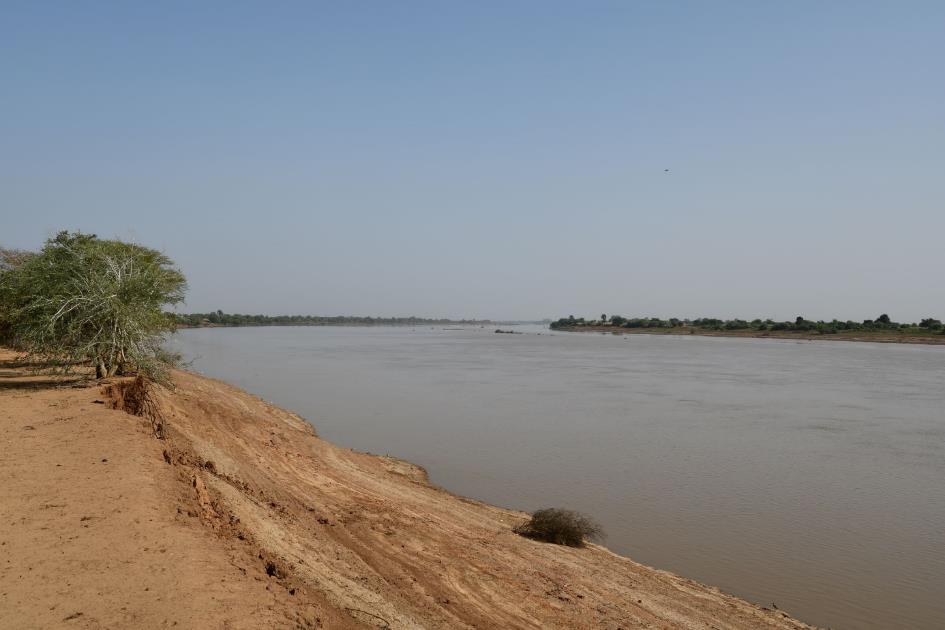


31 - Senegal River valley (North-west of Wompou, Province of Gorgol; November 2020)


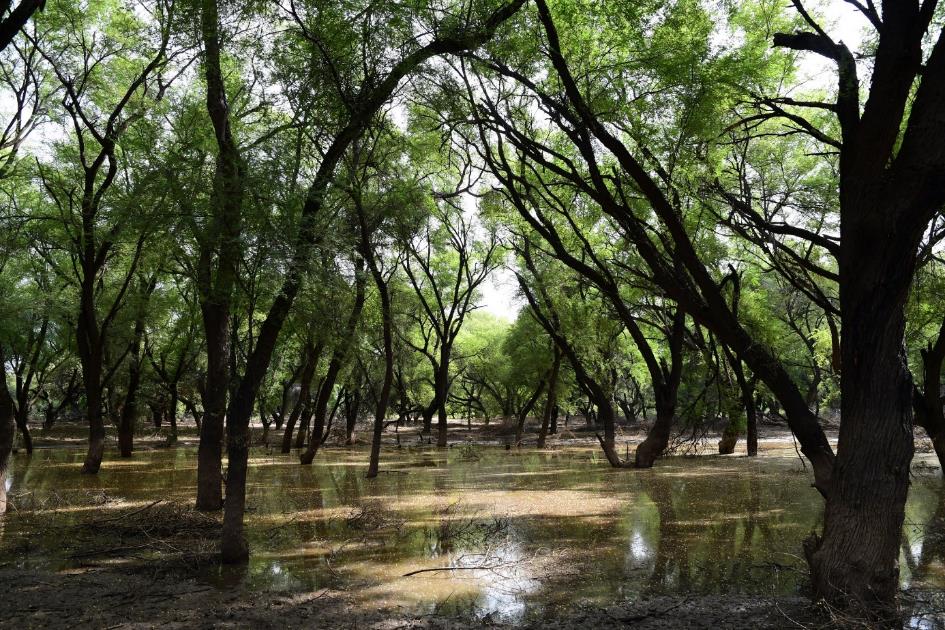


32 - Senegal River valley (Ngouye Classified Forest, Province of Gorgol; November 2020)


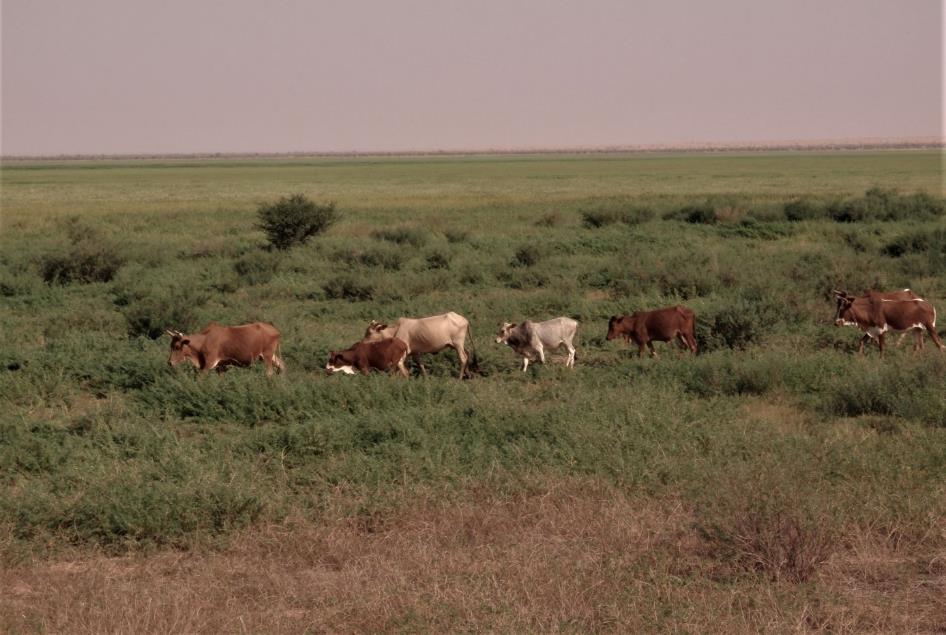


33 - Lac Gabou (Tagant plateau, Province of Tagant; November 2008)


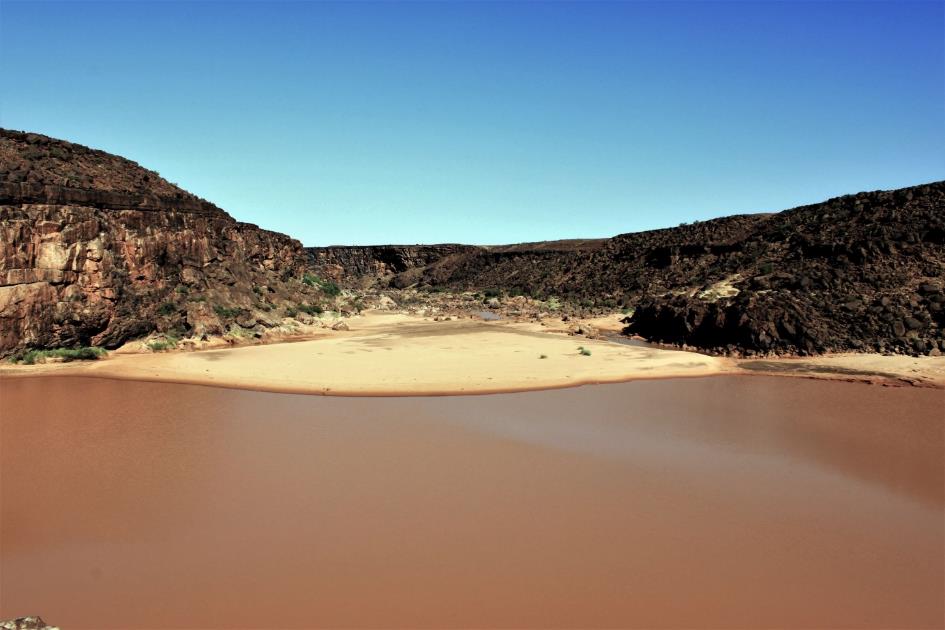


34 - *Guelta* Tartêga (Tagant plateau, Province of Tagant; November 2011)


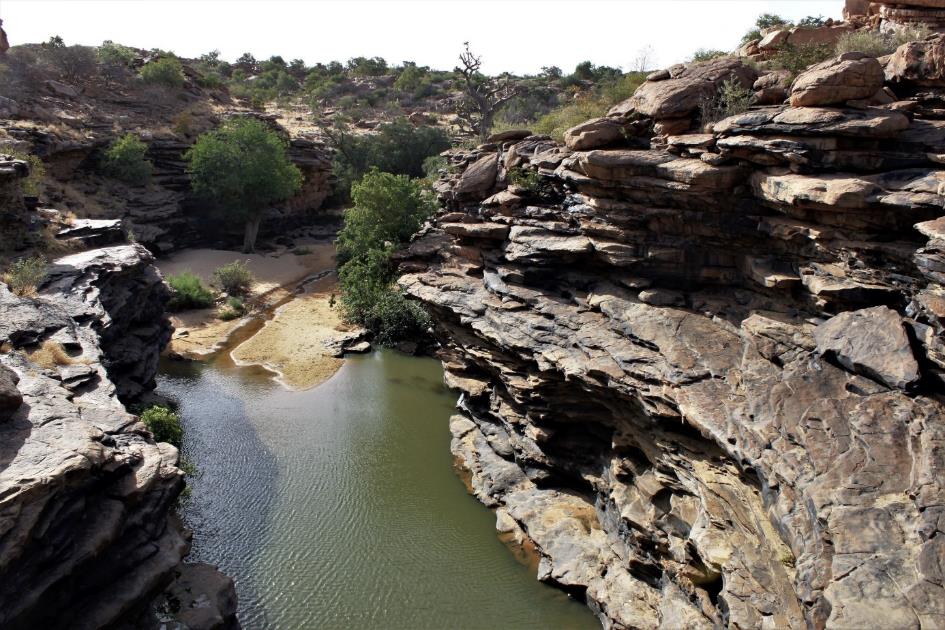


35 - *Guelta* El Barda (Assaba plateau, Province of Guidimaka; November 2012)


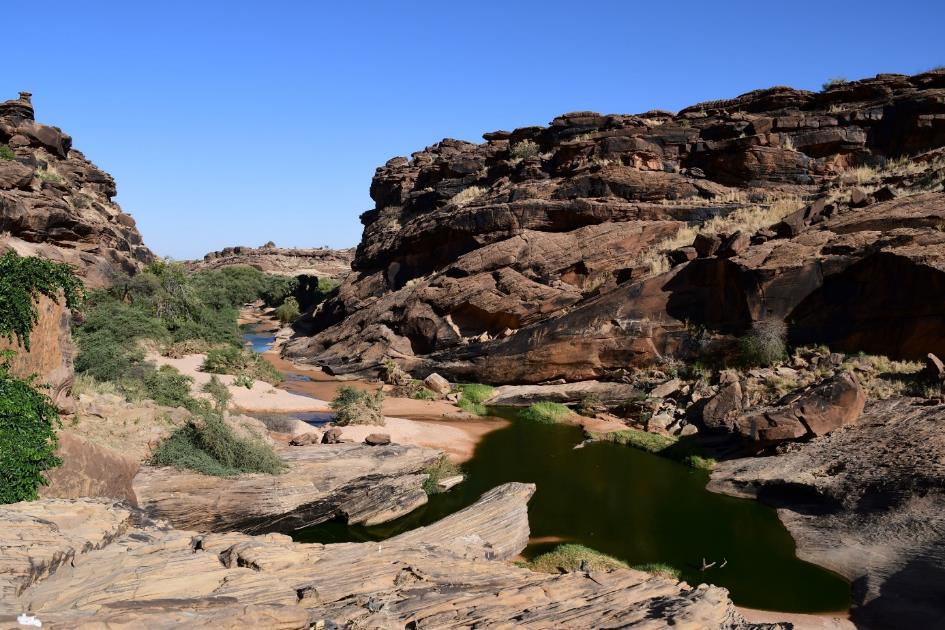


36 - *Guelta* Metraoucha (Afollé plateau, Province of Assaba; November 2020)


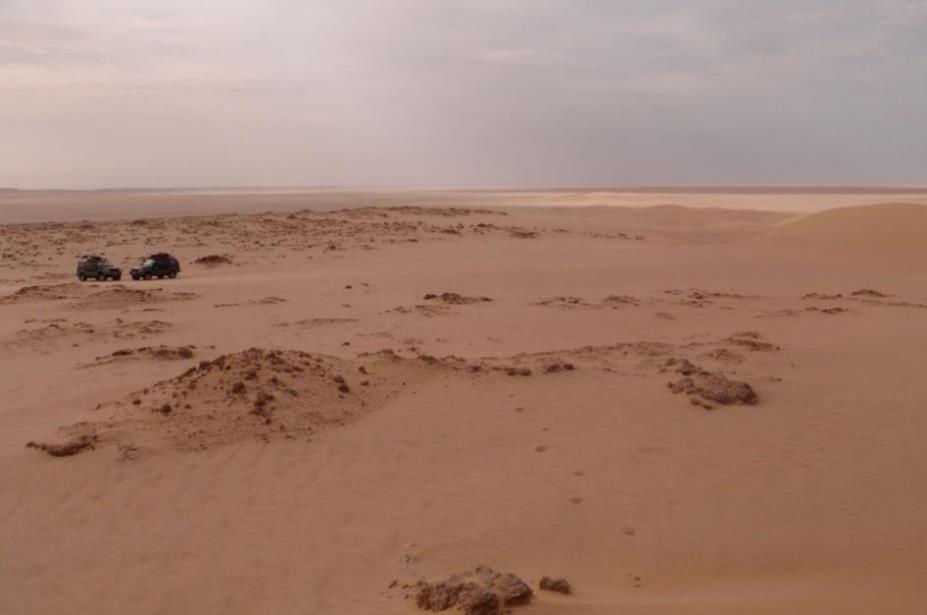


37 - Banc d’Arguin National Park (N-Tedâtene, Province of Dakhlet-Nouâdhibou; November 2008)


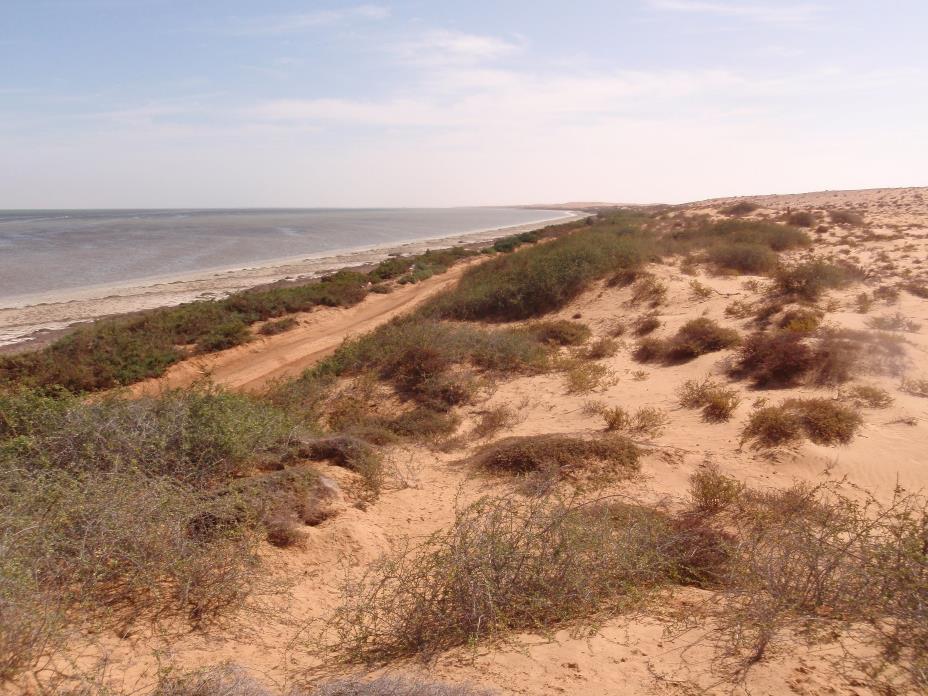


38 - Banc d’Arguin National Park (Teichott, Province of Dakhlet-Nouâdhibou; May 2009)


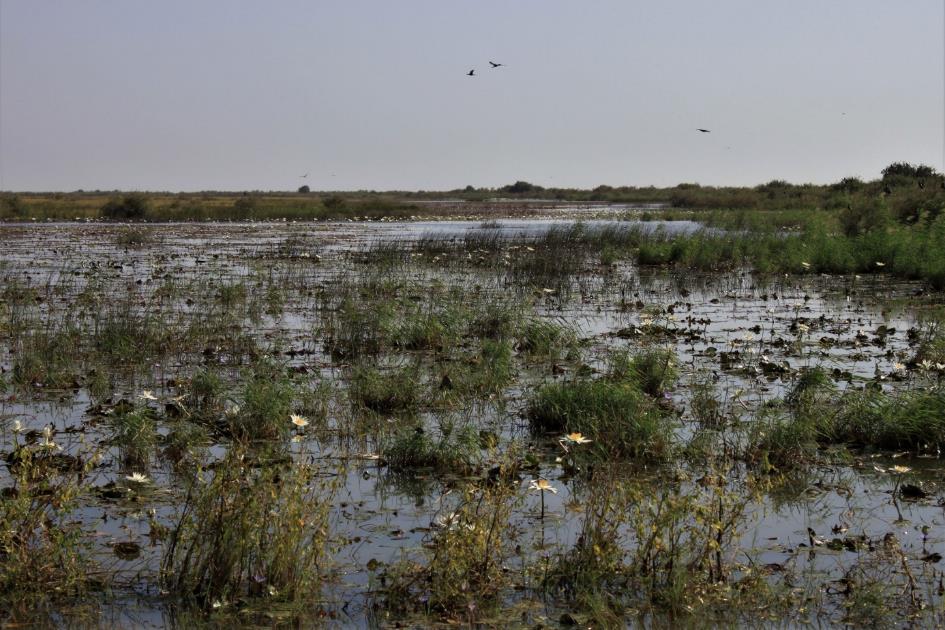


39 - Diawling National Park (Bell, Province of Trarza; November 2014)


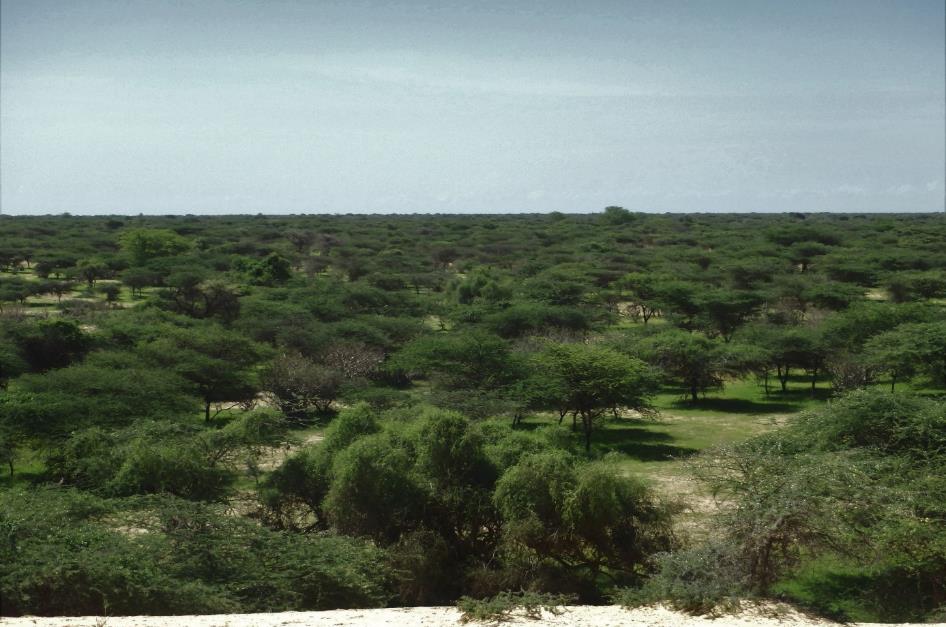


40 - Diawling National Park (Toundou Ziré, Province of Trarza; August 2015)
