# Supplementary material for: Diversity, distribution and conservation of land mammals in Mauritania, North-West Africa
Source: PLoS One. 2022 Aug 1;17(8):e0269870. doi: 10.1371/journal.pone.0269870 (PMC9342785; doi:10.1371/journal.pone.0269870)
Supplement: S7 Fig — Administrative regions (coloured areas; shadowed text) and capital of provinces [1] in Mauritania. (DOCX) [file pone.0269870.s007.docx]

**S7 Figure. Administrative units.** Administrative regions (coloured areas; shadowed text) and capital of provinces [1] in Mauritania.


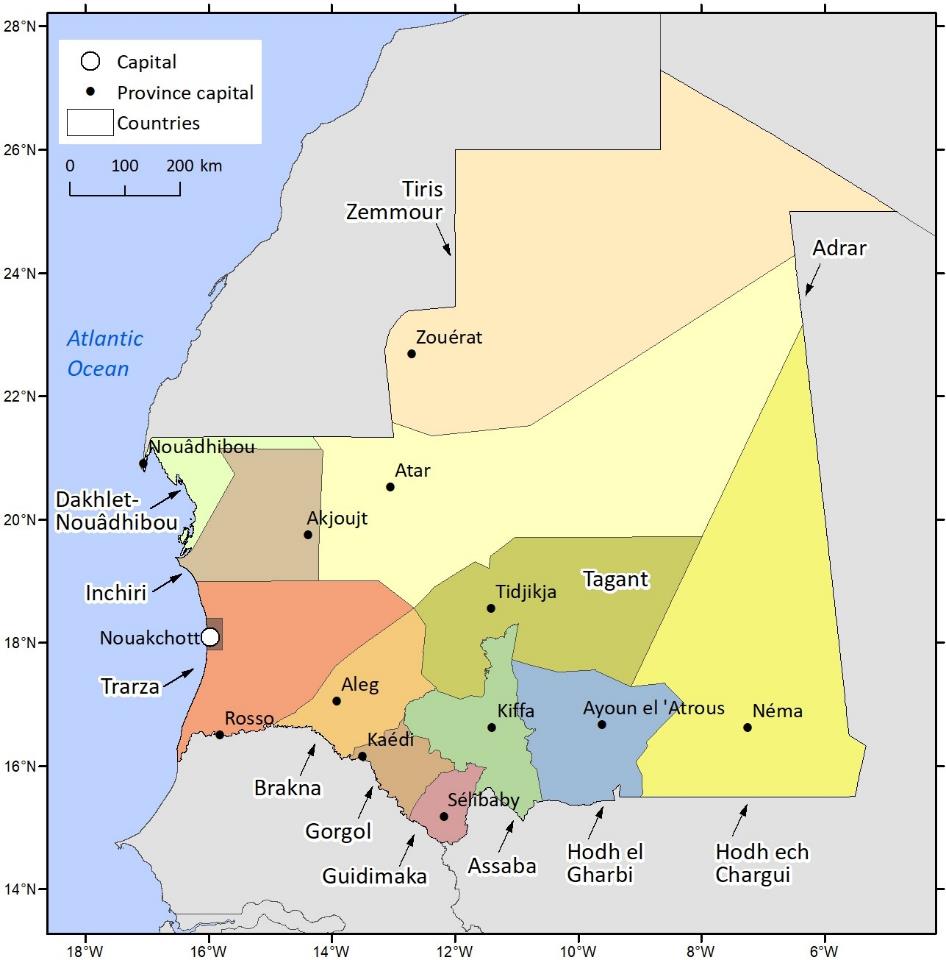


[1] Hijmans, R.J. Global Administrative Areas. V3.6. 2018. Available from: https://gadm.org/.
