# Supplementary material for: Diversity, distribution and conservation of land mammals in Mauritania, North-West Africa
Source: PLoS One. 2022 Aug 1;17(8):e0269870. doi: 10.1371/journal.pone.0269870 (PMC9342785; doi:10.1371/journal.pone.0269870)
Supplement: S8 Fig — Location of National Parks and Ramsar sites designated in Mauritania [1]. The Banc d’Arguin National Park is adjoined by the Cap Blanc Reserve, which protects the largest breeding colony of Mediterranean Monk Seal within its global range. A zoological park (Awleigatt) has been upgraded to national park category in 2016 [2] but it is not yet listed in the World Database of Protected Areas [1]. (DOCX) [file pone.0269870.s008.docx]

**S8 Figure. Protected Areas.** Location of National Parks and Ramsar sites designated in Mauritania [1]. The Banc d’Arguin National Park is adjoined by the Cap Blanc Reserve, which protects the largest breeding colony of Mediterranean Monk Seal within its global range. A zoological park (Awleigatt) has been upgraded to national park category in 2016 [2] but it is not yet listed in the World Database of Protected Areas [1].


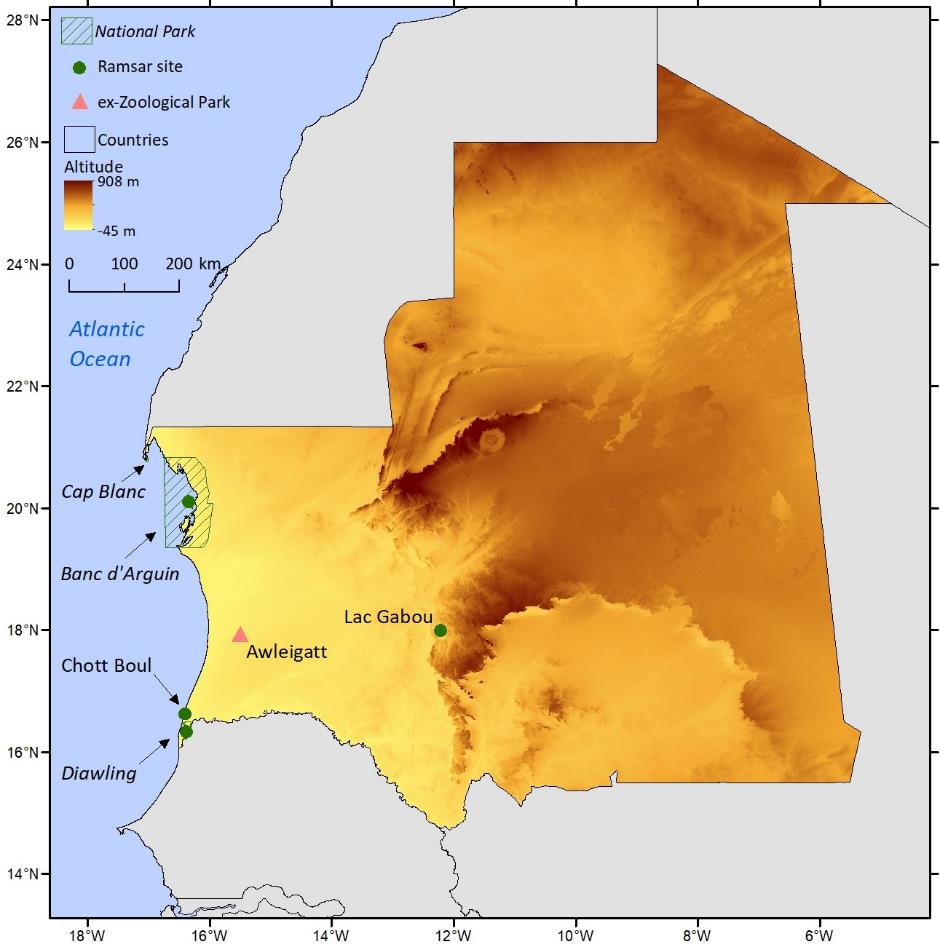


[1] UNEP-WCMC. Protected Area Profile for Mauritania from the World Database of Protected Areas, October 2021. [cited 2021 October 15]. Available from: www.protectedplanet.net.

[2] Thiam AB. Parc National d'Awleigatt. Manuel de la faune herbivore et de la flore ligneuse du Parc National de l'Awleigatt. Report. 23pp. Nouakchott: Ministère de l'Environnement et du Developpement Durable, République Islamique de la Mauritanie; 2020.
