# Supplementary material for: Diversity, distribution and conservation of land mammals in Mauritania, North-West Africa
Source: PLoS One. 2022 Aug 1;17(8):e0269870. doi: 10.1371/journal.pone.0269870 (PMC9342785; doi:10.1371/journal.pone.0269870)
Supplement: S9 Fig — Sampling routes taken by CIBIO team between 2002 and 2021, distribution of mammal observations collected during surveys and by collaborators, and national parks of Mauritania [1]. (DOCX) [file pone.0269870.s009.docx]

**S9 Figure. Sampling routes.** Sampling routes taken by CIBIO team between 2002 and 2021, distribution of mammal observations collected during surveys and by collaborators, and national parks of Mauritania [1].


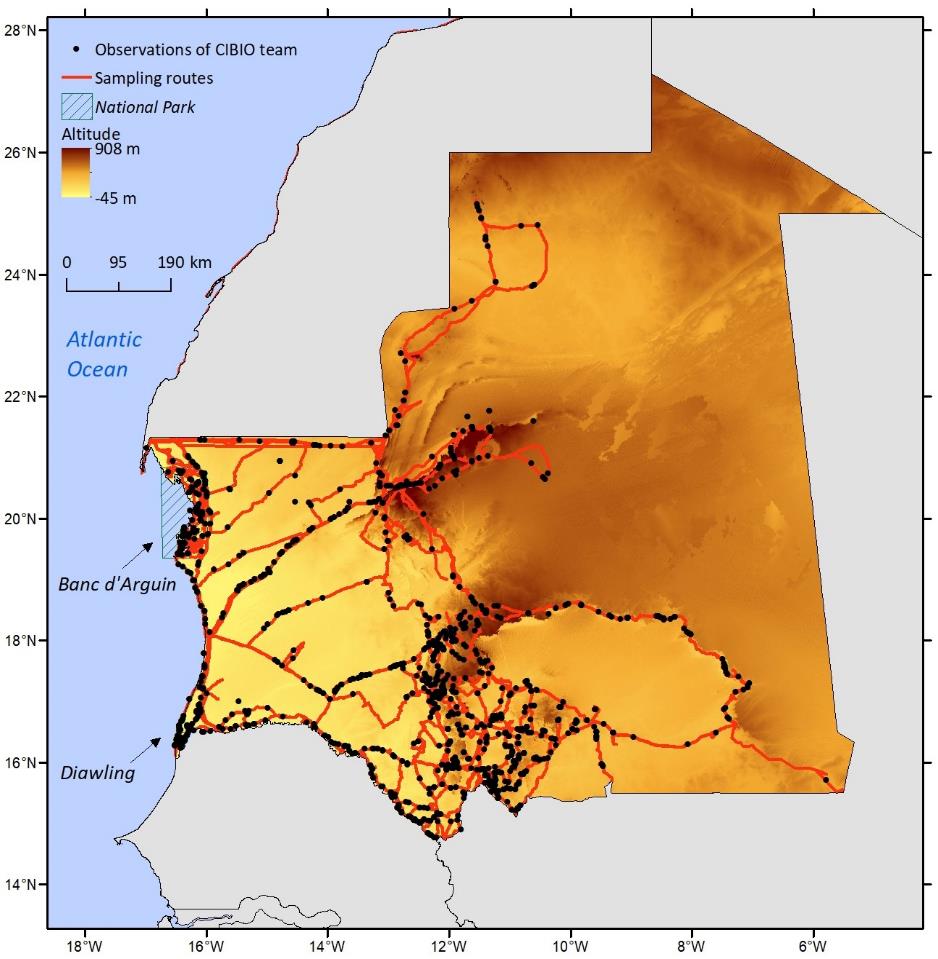


[1] UNEP-WCMC. Protected Area Profile for Mauritania from the World Database of Protected Areas, October 2021. [cited 2021 October 15]. Available from: www.protectedplanet.net.
