# Supplementary material for: Diversity, distribution and conservation of land mammals in Mauritania, North-West Africa
Source: PLoS One. 2022 Aug 1;17(8):e0269870. doi: 10.1371/journal.pone.0269870 (PMC9342785; doi:10.1371/journal.pone.0269870)

**S10 Figure. Examples of types of field observations.** Distribution of selected examples of types of field observations (black dots) of land mammals in Mauritania in relation to all compiled observations (grey dots): Camera trapping – observations collected by camera-trapping; Molecular species id. – observations with species identification confirmed by barcoding; Road-kills – observations of roadkill mammal specimens; and Captured – observations of live captured mammal specimens.


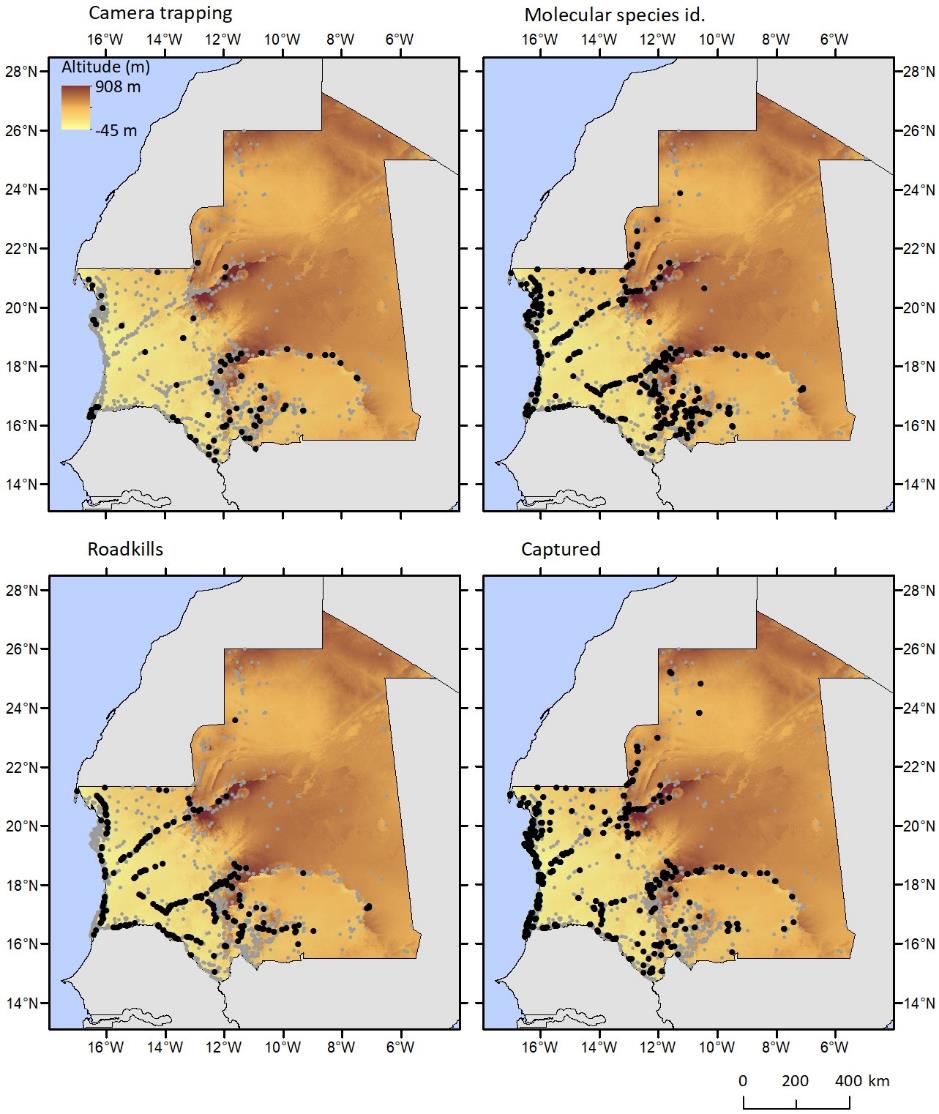

Supplement: S10 Fig — Distribution of selected examples of types of field observations (black dots) of land mammals in Mauritania in relation to all compiled observations (grey dots): Camera trapping—observations collected by camera-trapping; Molecular species id.—observations with species identification confirmed by barcoding; Road-kills—observations of roadkill mammal specimens; and Captured—observations of live captured mammal specimens. (DOCX) [file pone.0269870.s010.docx]
