# Supplementary material for: Diversity, distribution and conservation of land mammals in Mauritania, North-West Africa
Source: PLoS One. 2022 Aug 1;17(8):e0269870. doi: 10.1371/journal.pone.0269870 (PMC9342785; doi:10.1371/journal.pone.0269870)

**S12 Figure. Time periods of observations.** Distribution of observations (black dots) in four time periods in relation to all observations (grey dots) of land mammals in Mauritania: >2000 – observations from after the year 2000; 1980-1999 – observations from between the years 1980 and 1999; 1900-1979 – observations from between the years 1900 and 1979; and <1900 – observations from before the year 1900.


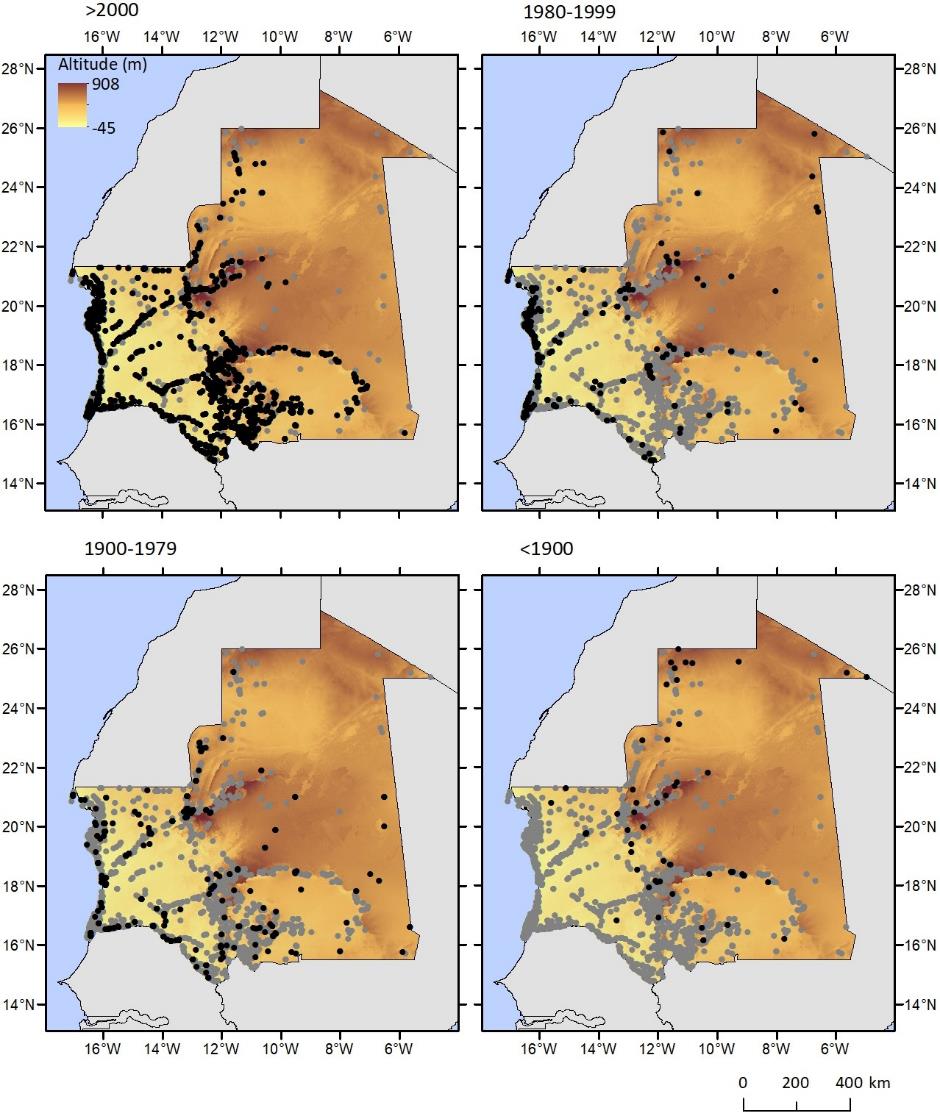

Supplement: S11 Fig — Distribution of observations (black dots) in four time periods in relation to all observations (grey dots) of land mammals in Mauritania: >2000 —observations from after the year 2000; 1980—1999 —observations from between the years 1980 and 1999; 1900—1979 —observations from between the years 1900 and 1979; and <1900 —observations from before the year 1900. (DOCX) [file pone.0269870.s011.docx]
