# Supplementary material for: Diversity, distribution and conservation of land mammals in Mauritania, North-West Africa
Source: PLoS One. 2022 Aug 1;17(8):e0269870. doi: 10.1371/journal.pone.0269870 (PMC9342785; doi:10.1371/journal.pone.0269870)

**S13 Figure. Reference grid UTM 100 km.** Alphanumeric code of each 100 km grid cell size on the projected coordinate system Africa Albers Equal Area Conic. External units on X- and Y-axes refer to the Universal Transverse Mercator (UTM) coordinate system.


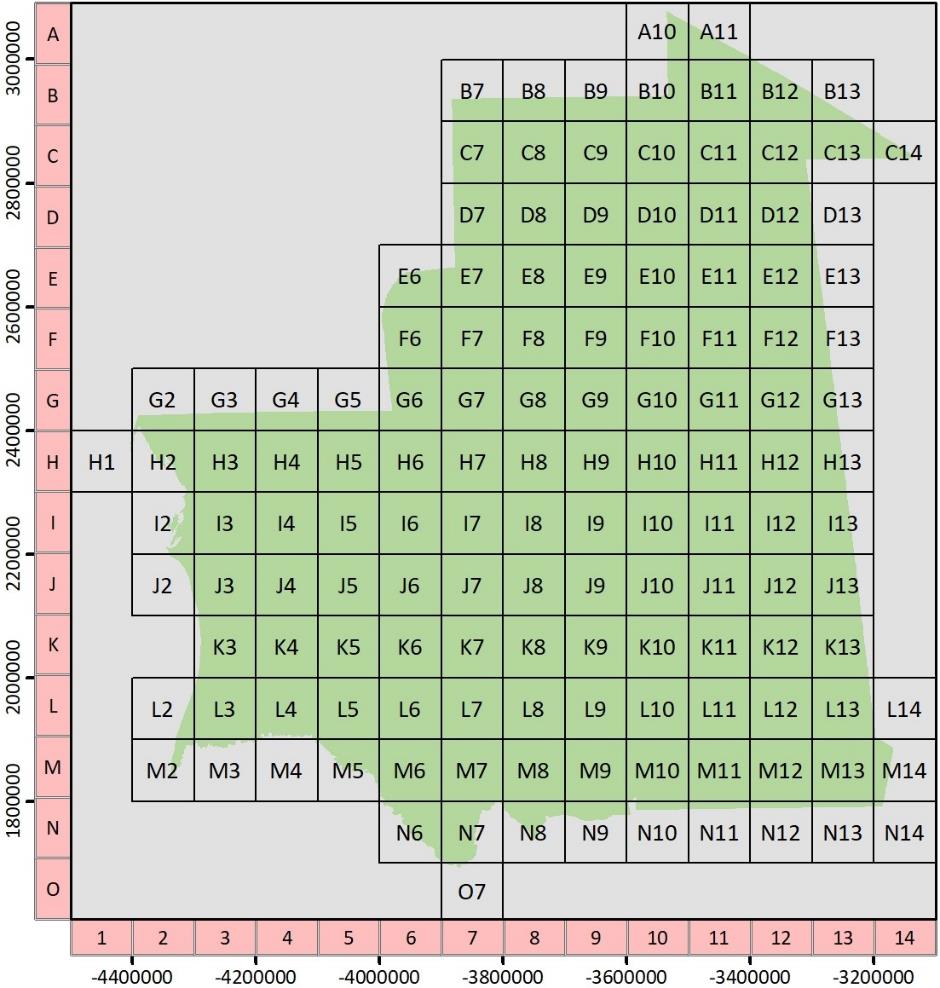

Supplement: S12 Fig — Alphanumeric code of each 100 km grid cell size on the projected coordinate system Africa Albers Equal Area Conic. External units on X- and Y-axes refer to the Universal Transverse Mercator (UTM) coordinate system. (DOCX) [file pone.0269870.s012.docx]
