# Supplementary material for: Diversity, distribution and conservation of land mammals in Mauritania, North-West Africa
Source: PLoS One. 2022 Aug 1;17(8):e0269870. doi: 10.1371/journal.pone.0269870 (PMC9342785; doi:10.1371/journal.pone.0269870)

**S14 Figure. Distribution of number of observations.** Number of observations of land mammals in Mauritania in each 100 km UTM grid cell**.** Mountain plateaus and escarpments (black dashed) and national parks (green shaded; in italics) are identified.


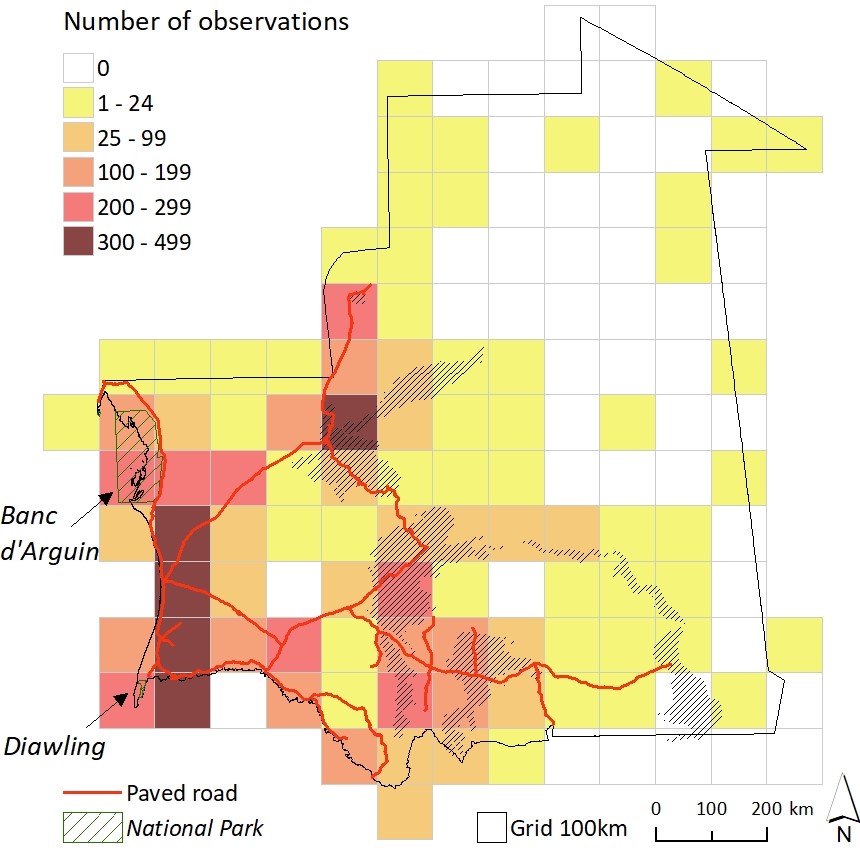

Supplement: S13 Fig — Number of observations of land mammals in Mauritania in each 100 km UTM grid cell. Mountain plateaus and escarpments (black dashed) and national parks (green shaded; in italics) are identified. (DOCX) [file pone.0269870.s013.docx]
