# Supplementary material for: Diversity, distribution and conservation of land mammals in Mauritania, North-West Africa
Source: PLoS One. 2022 Aug 1;17(8):e0269870. doi: 10.1371/journal.pone.0269870 (PMC9342785; doi:10.1371/journal.pone.0269870)
Supplement: S14 Fig — Status and distribution maps of land mammals in Mauritania. Comparisons between the distribution maps and the range polygons available from IUCN [1] are given below figure captions. The extinction risk category at the global [1] and national level (this study) are presented and commented when pertinent. References provided in legends are available in S1 Text. (DOCX) [file pone.0269870.s014.docx]

**S18 Figure. Species distribution maps.** Status and distribution maps of land mammals in Mauritania. Comparisons between the distribution maps and the range polygons available from IUCN [1] are given below figure captions. The extinction risk category at the global [1] and national level (this study) are presented and commented when pertinent. References provided in legends are available in S11 Text.

[1] IUCN. The IUCN Red List of Threatened Species. Version 2021-2. 2021. [cited 2021 October 12]. Available from: https://www.iucnredlist.org.


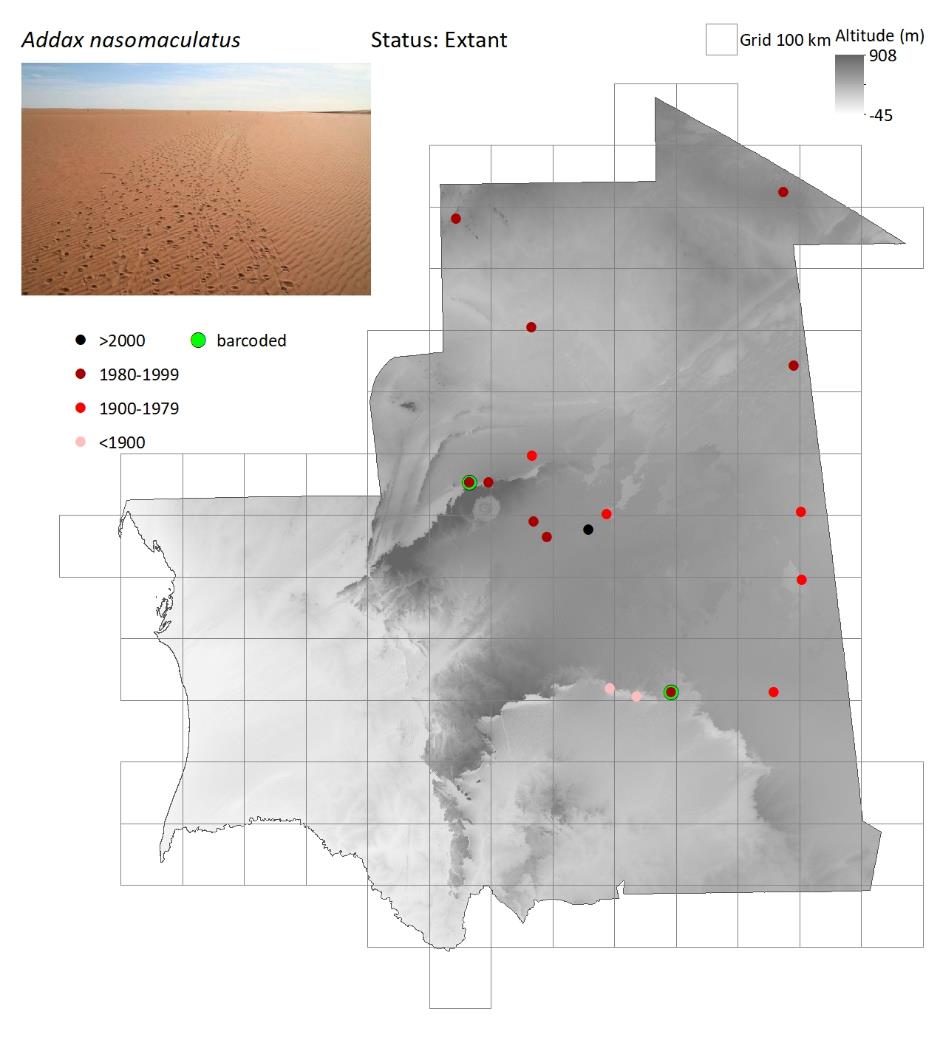


001 – Status and distribution of *Addax nasomaculatus* in Mauritania. Picture of footprints in Ouarâne, Province of Adrar (1^st^ April 2007). The picture was taken after observing a group of 15 individuals (Vernet 2008). No observations were made after that date. The barcoded observations correspond to horns kept by former hunter (north) or found during field surveys (south-west).

Comparison with IUCN range polygons: No change. Mapped distribution is similar to reported range in Mauritania.

Conservation status: Global IUCN- CR; National final- CR C2a(ii); D; National original- CR.

Comment on the assessment: Downgrading excluded due to the absence of neighbouring populations. All mature individuals were considered to occur in one single subpopulation.


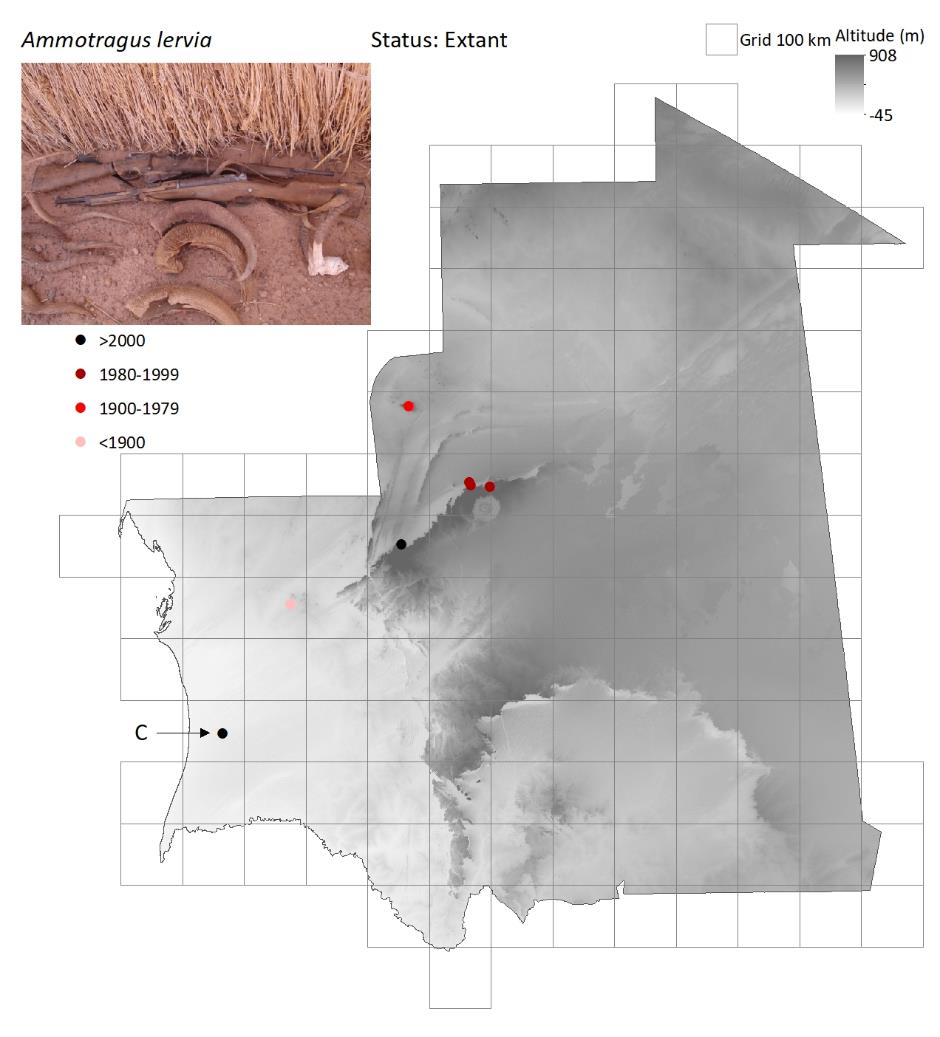


002 – Status and distribution of *Ammotragus lervia* in Mauritania. Picture of horns in Tarf Tazazmout, Province of Tiris Zemmour (March 2009). Interviews to local people confirmed the presence in the area in the 1980s. The firearms belonged to a former hunter who also captured at the time *Addax nasomaculatus* (left side horns) and *Gazella dorcas* (right side skull and horns). The species has captive individuals in the PN Awleigatt, Province of Trarza (Thiam 2020), where a captive population was established (signalled as C in map).

Comparison with IUCN range polygons: No change. Mapped distribution is similar to reported range in Mauritania.

Conservation status: Global IUCN- VU; National final- EN B1a,b(i,iii,v); C2a(i); D; National original- EN.

Comment on the assessment: Downgrading excluded due to isolation of Mauritanian population from neighbouring populations in Morocco. Inferred continuous population decline and decline in area, extent, and habitat quality due to hunting, disturbance, overgrazing, and frequent drought.


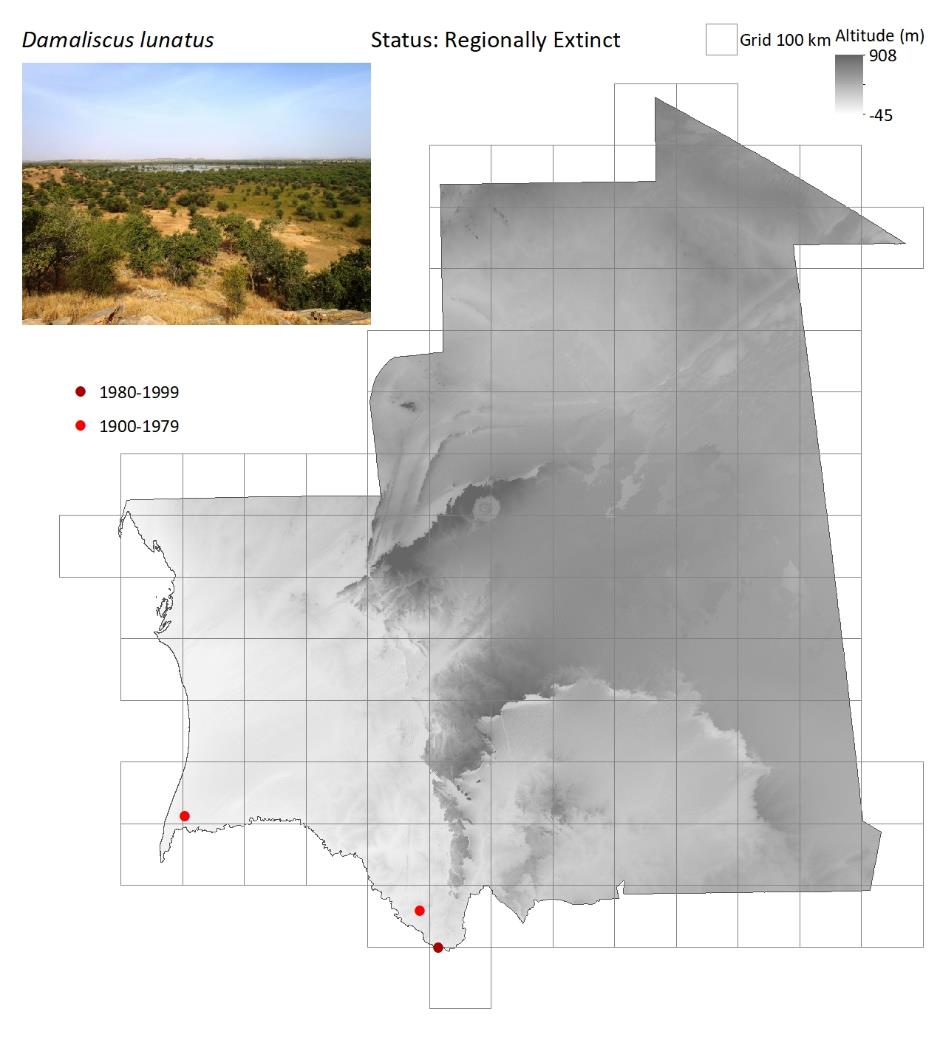


003 - Status and distribution of *Damaliscus lunatus* in Mauritania. Picture of the habitat in the Senegal River floodplain, Province of Guidimaka (November 2012), where the species was known to occur in the 1980s (Mauritanie 2000 SARL 2001). No observations were made after that period.

Comparison with IUCN range polygons: Not possible. The reported range of the species does not include areas with extinct populations.

Conservation status: Global IUCN- LC; National final- RE; National original- RE.


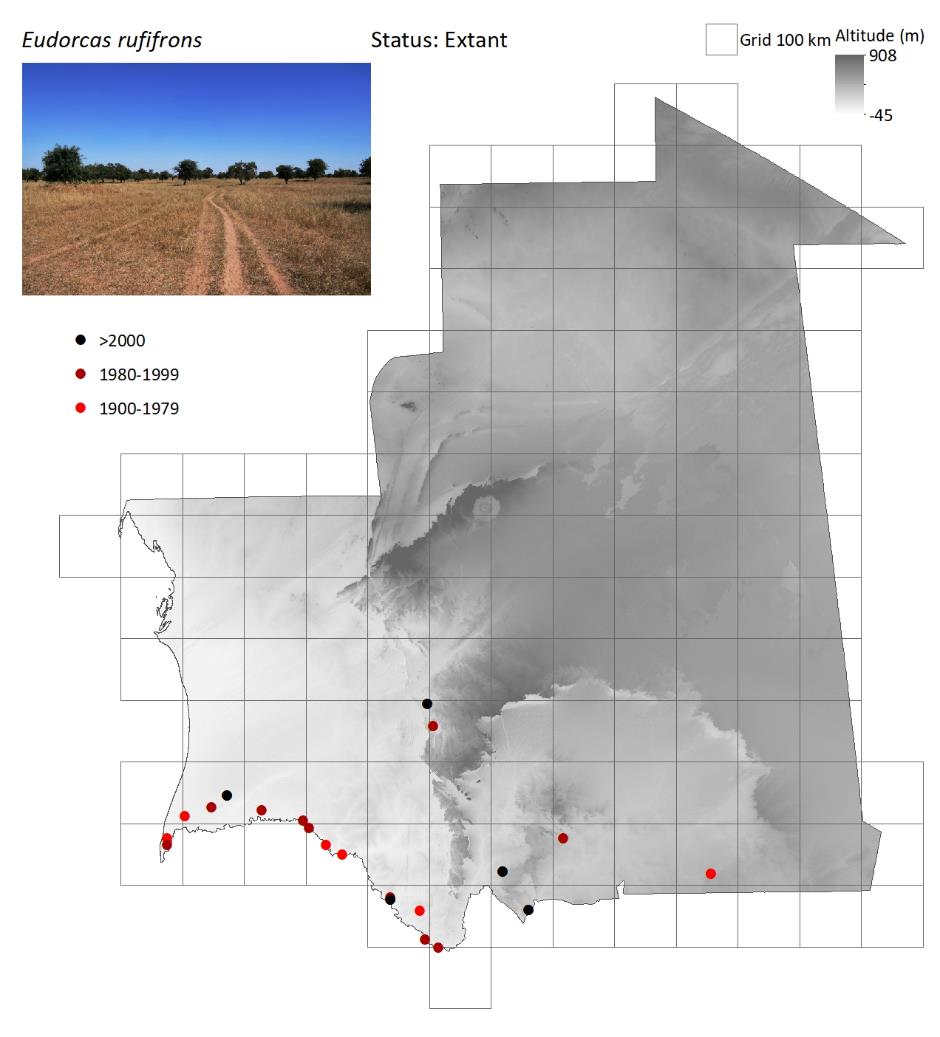


004 - Status and distribution of *Eudorcas rufifrons* in Mauritania. Picture of the habitat near Mare de Toya, Province of Assaba (November 2020), where interviews to local people reported the current occurrence of the species.

Comparison with IUCN range polygons: No change. Mapped distribution is similar to reported range in Mauritania.

Conservation status: Global IUCN- VU; National final- EN C2a(i); National original- EN.

Comment on the assessment: Downgrading excluded due lack of sufficient evidence proving rescue effect from neighbouring populations in Mali and Senegal. Inferred continuous population decline due to hunting, disturbance, overgrazing, and frequent drought.


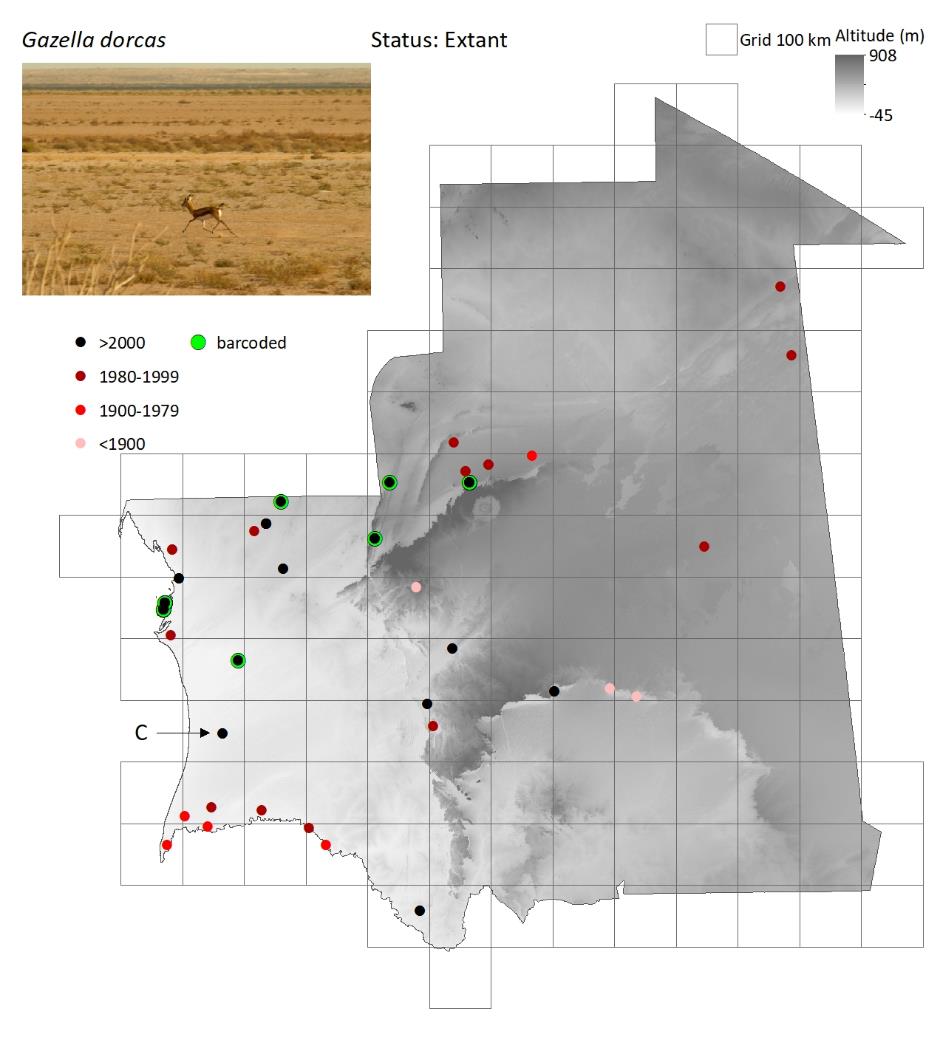


005 – Status and distribution of *Gazella dorcas* in Mauritania. Picture in Tidra Island, Province of Dakhlet-Nouâdhibou (November 2011). The species has captive individuals in the PN Awleigatt, Province of Trarza (Thiam 2020), where a captive population was established (signalled as C in map).

Comparison with IUCN range polygons: Range expansion. The observations along the Senegal River valley apparently expand southwards the reported range of the species in Mauritania. However, there are no vouchers or photographs from this area, and possibly may represent misidentifications with *Eudorcas rufifrons*. Additional sampling is needed to understand the current range of the species.

Conservation status: Global IUCN- VU; National final- VU C2a(i); National original- VU.

Comment on the assessment: Downgrading excluded due lack of sufficient evidence proving rescue effect from neighbouring populations in Algeria, Morocco and Mali. Inferred continuous population decline due to hunting, disturbance, overgrazing, and frequent drought.


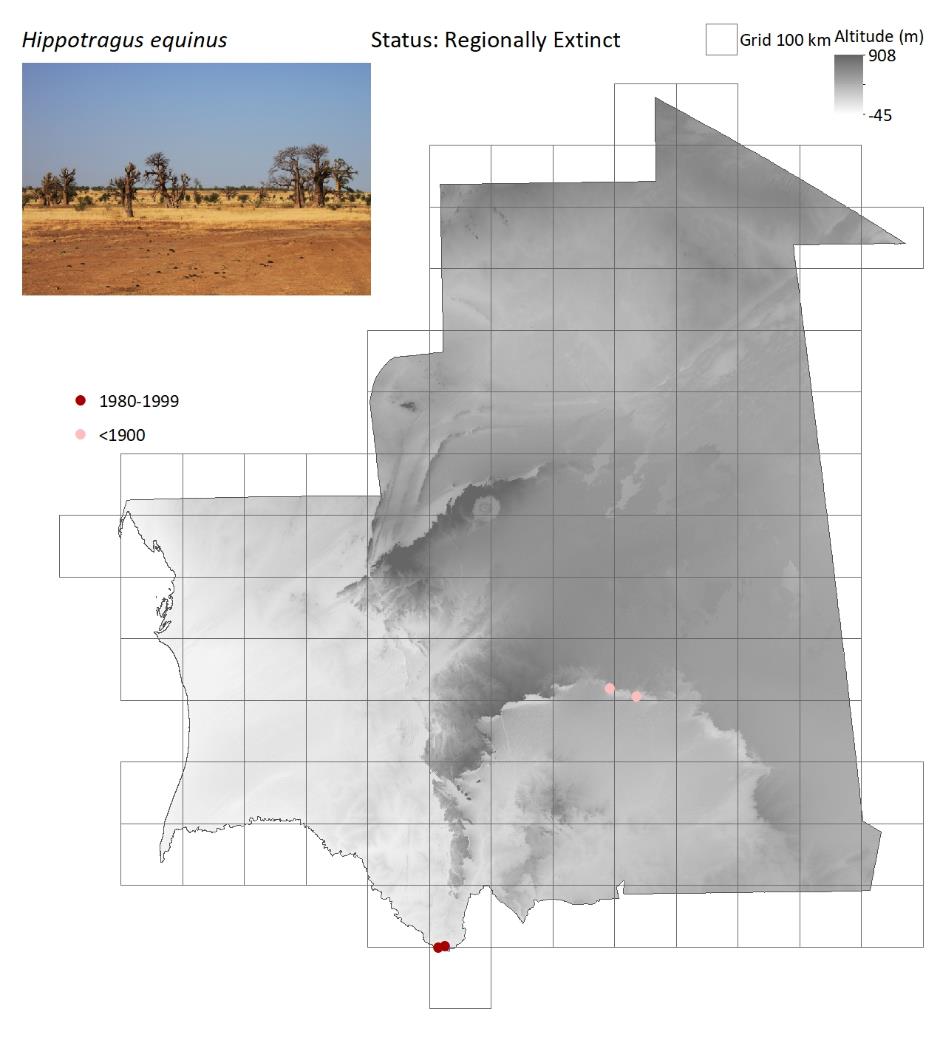


006 – Status and distribution of *Hippotragus equinus* in Mauritania. Picture of the habitat in Baédiam, Province of Guidimaka (November 2014), where the species was known to occur in the 1980s (East 1999; Mauritanie 2000 SARL 2001). No observations were made after that period.

Comparison with IUCN range polygons: Not possible. The reported range of the species does not cover Mauritania and does not include areas with extinct populations.

Conservation status: Global IUCN- LC; National final- RE; National original- RE.


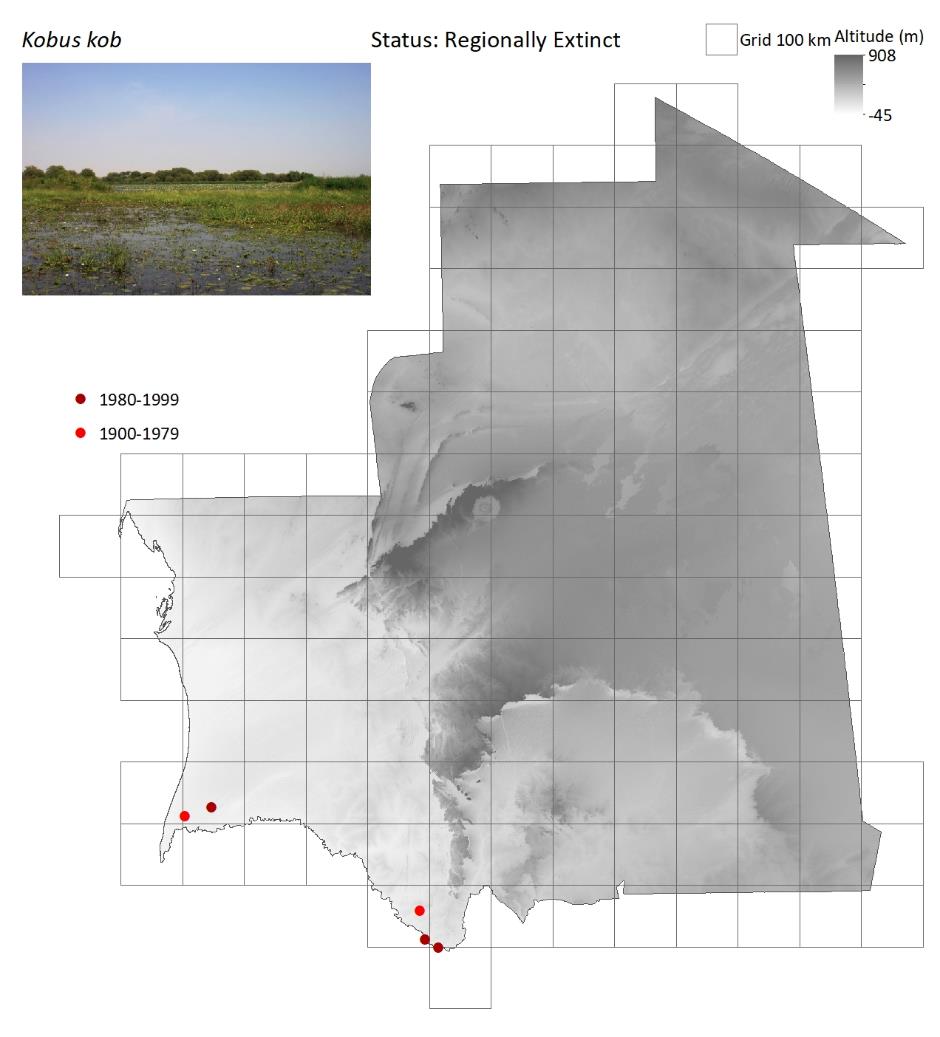


007 – Status and distribution of *Kobus kob* in Mauritania. Picture of the habitat in the Senegal River floodplain near Gouraye, Province of Guidimaka (November 2014), where the species was known to occur in the 1980s (East 1999; Mauritanie 2000 SARL 2001). No observations were made after that period.

Comparison with IUCN range polygons: Better definition of former range. The observations in the lower Senegal River valley expand westwards the reported former range of the species in Mauritania.

Conservation status: Global IUCN- LC; National final- RE; National original- RE.


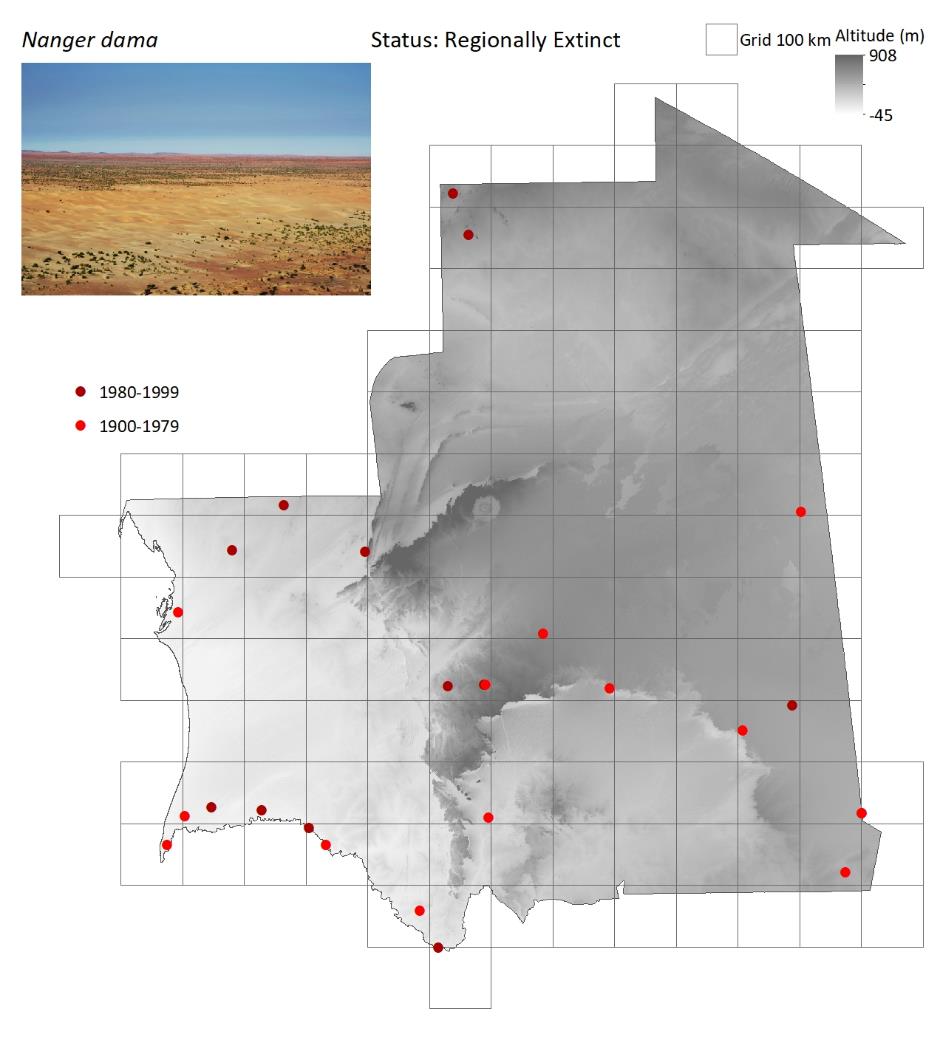


008 - Status and distribution of *Nanger dama* in Mauritania. Picture of the habitat in El Khat River system, Province of Tagant (November 2019), where the species was known to occur in the 1980s (Dia 2004; Gueye and Dia 2004). No observations were made after that period.

Comparison with IUCN range polygons: Not possible. The reported former range of the species does not cover Mauritania, neither as extinct populations.

Conservation status: Global IUCN- CR; National final- RE; National original- RE.


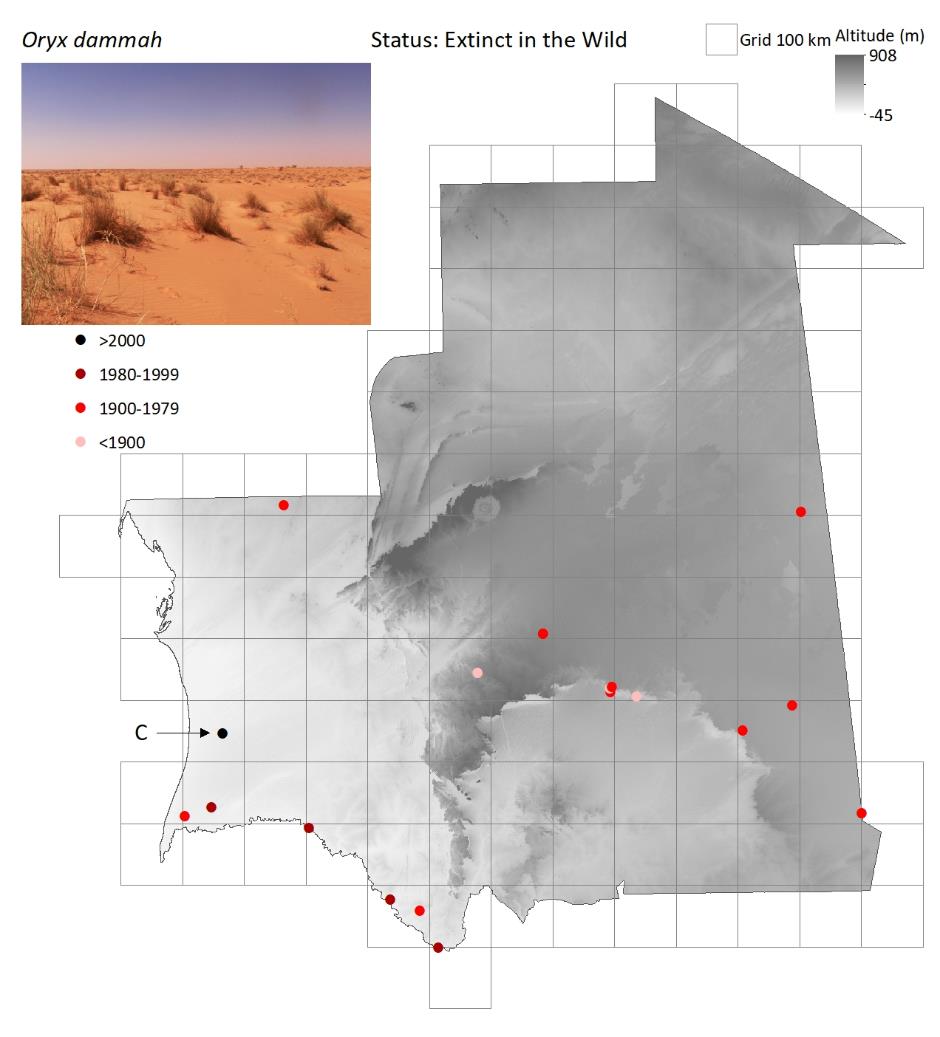


009 - Status and distribution of *Oryx dammah* in Mauritania. Picture of the habitat in the Azeffâl dunes, Province of Dakhlet-Nouâdhibou (March 2009), where the species was known to occur in the 1960s (Beudels-Jamar et al 2006). The species has captive individuals in the PN Awleigatt, Province of Trarza (Thiam 2020), where a captive population was established (signalled as C in map).

Comparison with IUCN range polygons: Better definition of former range. The observations in eastern Mauritania expand north-eastwards the reported former range of the species in Mauritania.

Conservation status: Global IUCN- EW; National final- EW; National original- EW.


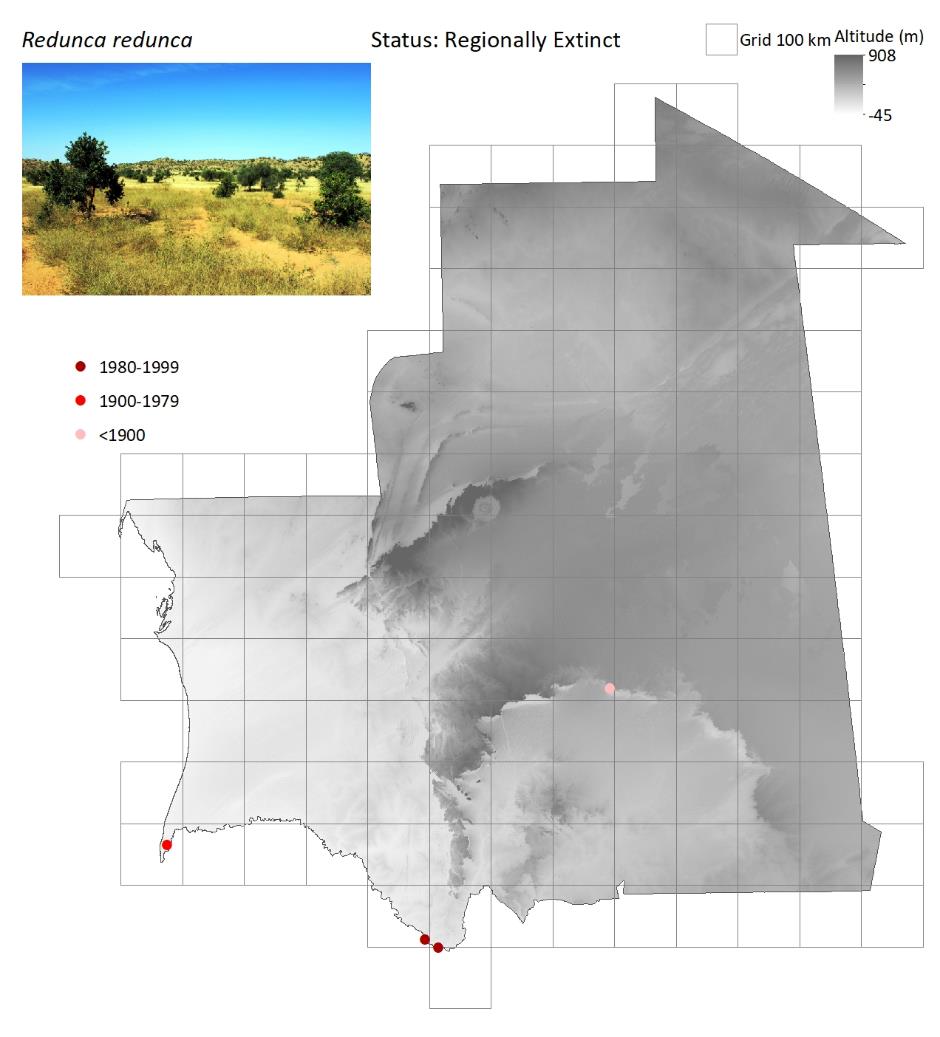


010 – Status and distribution of *Redunca redunca* in Mauritania. Picture of the habitat south of Sélibaby, Province of Guidimaka (November 2011), where the species was known to occur in the 1980s (East 1999; Mauritanie 2000 SARL 2001). No observations were made after that period.

Comparison with IUCN range polygons: Range contraction. The reported current range of the species covers the Province of Guidimaka but the occurrence status in Mauritania is now considered Extinct.

Conservation status: Global IUCN- LC; National final- RE; National original- RE.


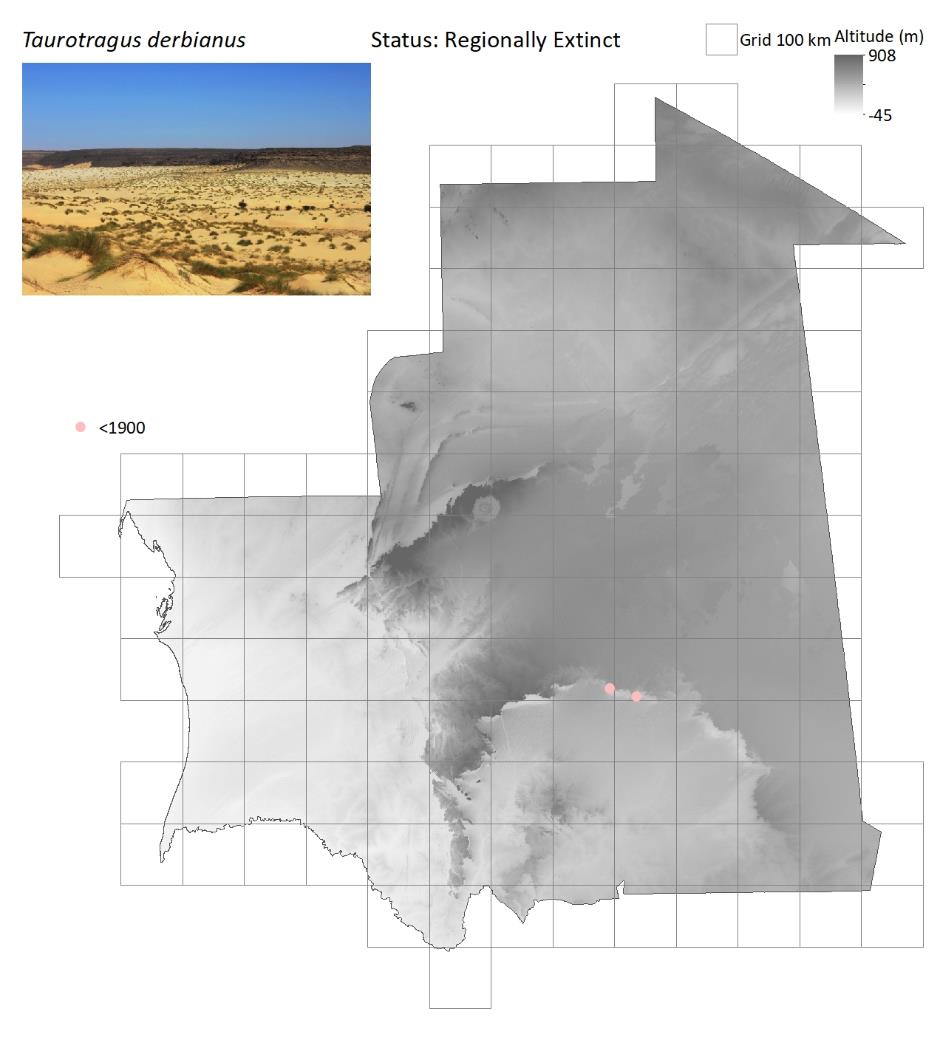


011 – Status and distribution of *Taurotragus derbianus* (=*Tragelaphus derbianus*) in Mauritania. Picture of the habitat in Dhar Tichitt, Province of Tagant (February 2014), where subfossil remains of the species were recovered (Holl 1985, 2009).

Comparison with IUCN range polygons: Not possible. The reported range of the species does not include areas with extinct populations.

Conservation status: Global IUCN- VU; National final- RE; National original- RE.


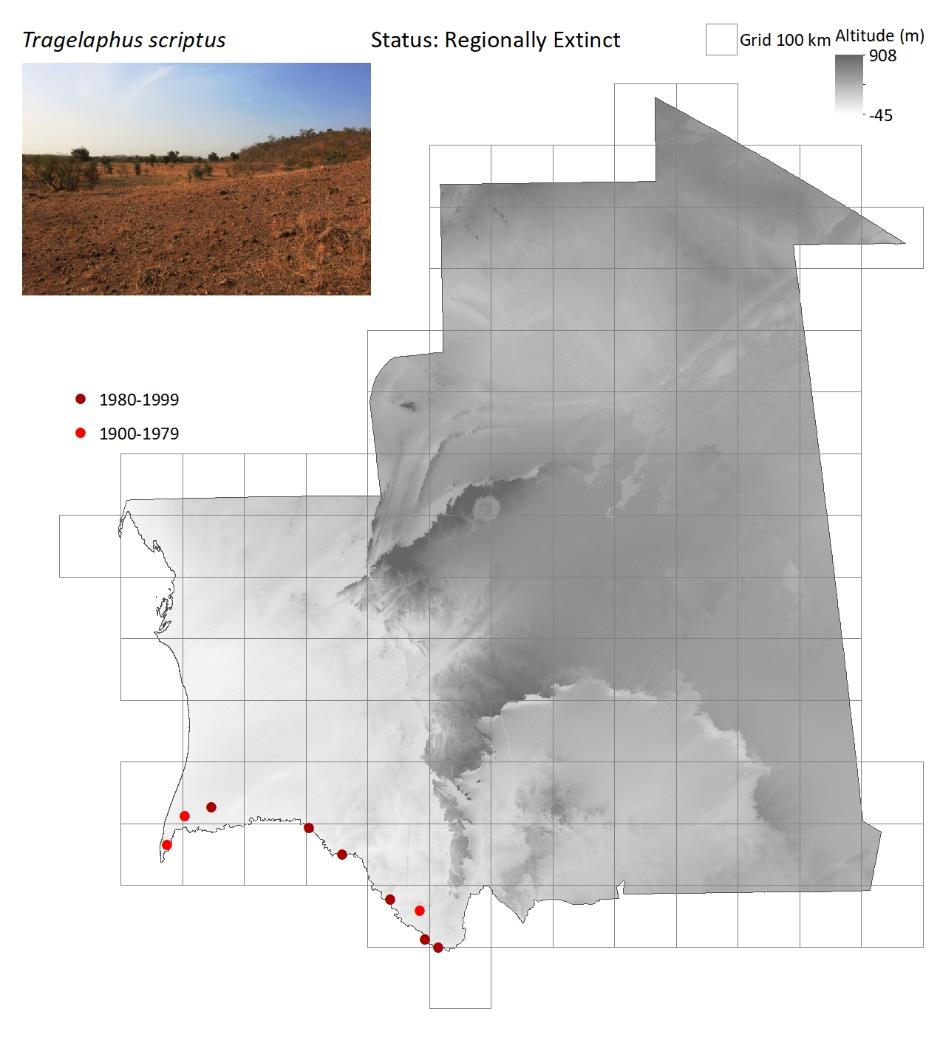


012 – Status and distribution of *Tragelaphus scriptus* in Mauritania. Picture of the habitat south of Sélibaby, Province of Guidimaka (January 2014), where the species was known to occur in the 1980s (East 1999; Mauritanie 2000 SARL 2001). No observations were made after that period.

Comparison with IUCN range polygons: Range contraction. The reported current range of the species covers the Province of Guidimaka but the occurrence status in Mauritania is now considered Extinct.

Conservation status: Global IUCN- LC; National final- RE; National original- RE.


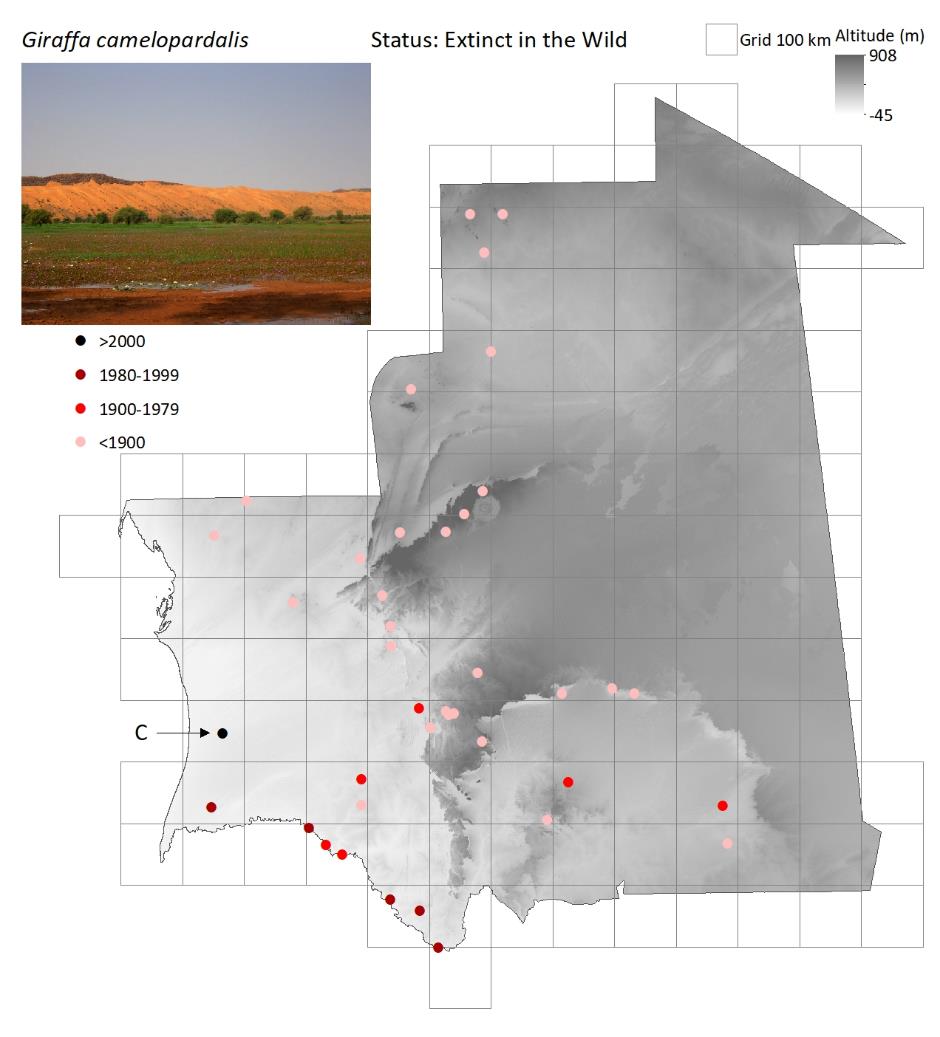


013 - Status and distribution of *Giraffa camelopardalis* in Mauritania. Picture of the habitat in the Gabou basin, Province of Tagant (November 2003), where the species was known to occur until the 1970s (Ciofolo 1995). The species has captive individuals in the PN Awleigatt, Province of Trarza (Thiam 2020), where a captive population was established (signalled as C in map).

Comparison with IUCN range polygons: Not possible. The reported range of the species does not include areas with extinct populations.

Conservation status: Global IUCN- VU; National final- EW; National original- EW.


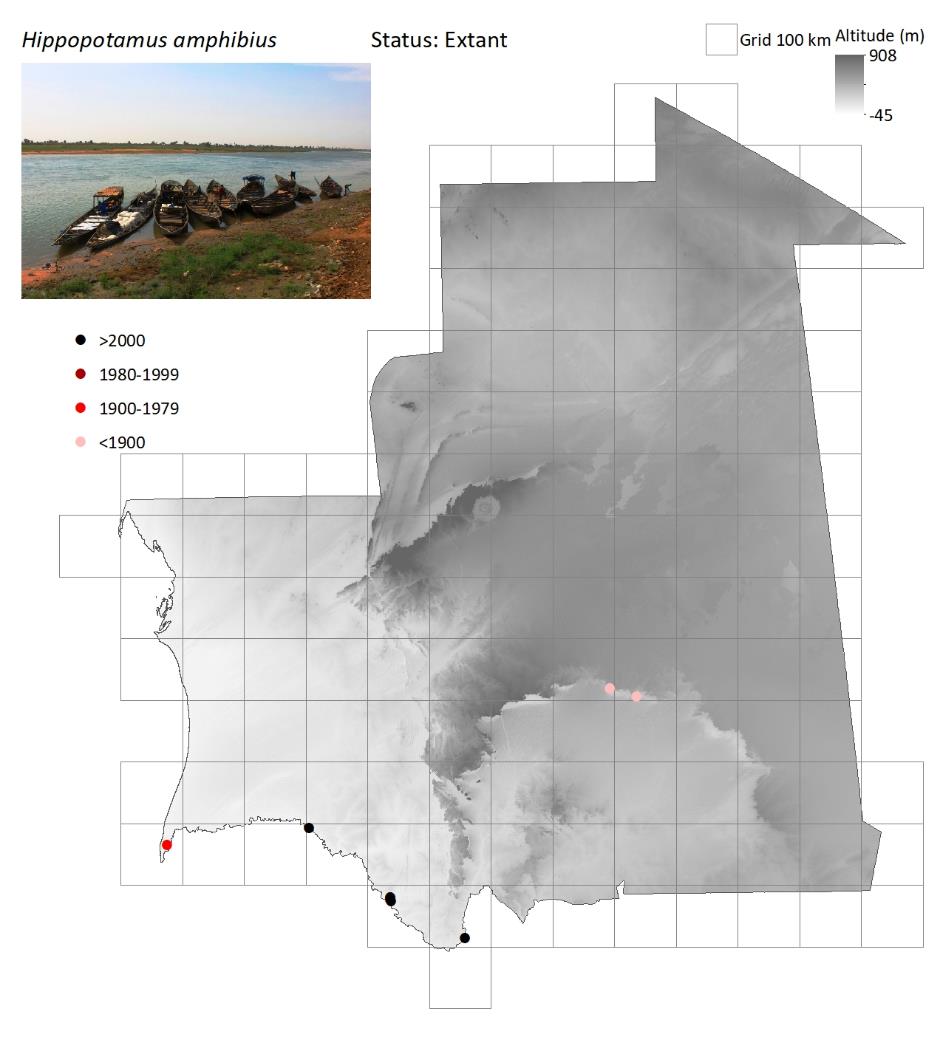


014 - Status and distribution of *Hippopotamus amphibius* in Mauritania. Picture of the habitat in the Senegal River in Khabou-Guidimaka, Province of Guidimaka (January 2014), where interviews to local people reported the presence of the species. The species was known to occur in Diawling National Park at least until the 1960s (Ba et al. 2002), but no observations were made after that period.

Comparison with IUCN range polygons: New species for Mauritania. The observations along the Senegal River valley and tributaries confirm the occurrence of the species in Mauritania and expand the reported range, as the closest known populations are from the Gambia River valley in Senegal.

Conservation status: Global IUCN- VU; National final- CR C2a(i); D; National original- CR.

Comment on the assessment: Downgrading excluded due lack of sufficient evidence proving rescue effect from neighbouring populations in Mali and Senegal. Inferred continuous population decline due to hunting and habitat disturbance.


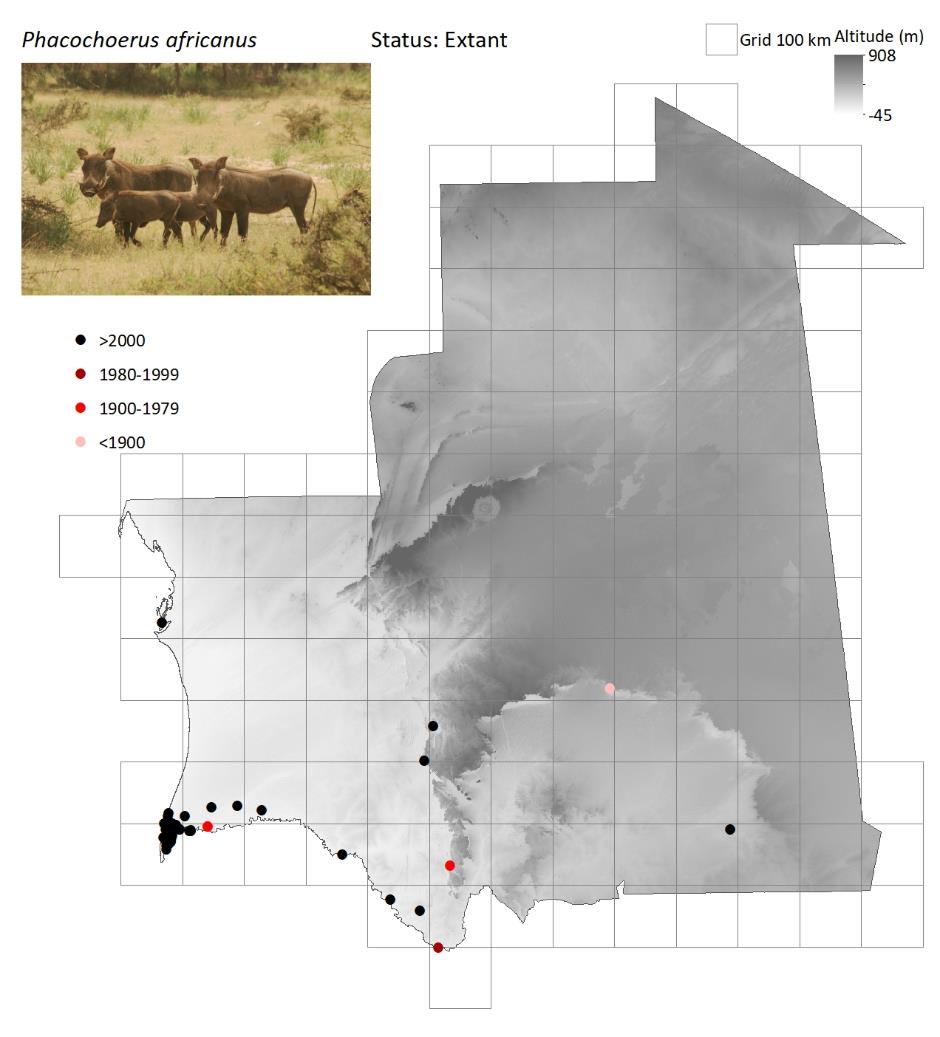


015 - Status and distribution of *Phacochoerus africanus* in Mauritania. Picture in the Diawling National Park, Province of Trarza (October 2010).

Comparison with IUCN range polygons: Range expansion. The observations in the Banc d’Arguin National Park, the Tagant mountain, and in inland Hodh Ech Chargui Province expand northwards and eastwards the reported range of the species in Mauritania.

Conservation status: Global IUCN- LC; National final- LC; National original- LC.


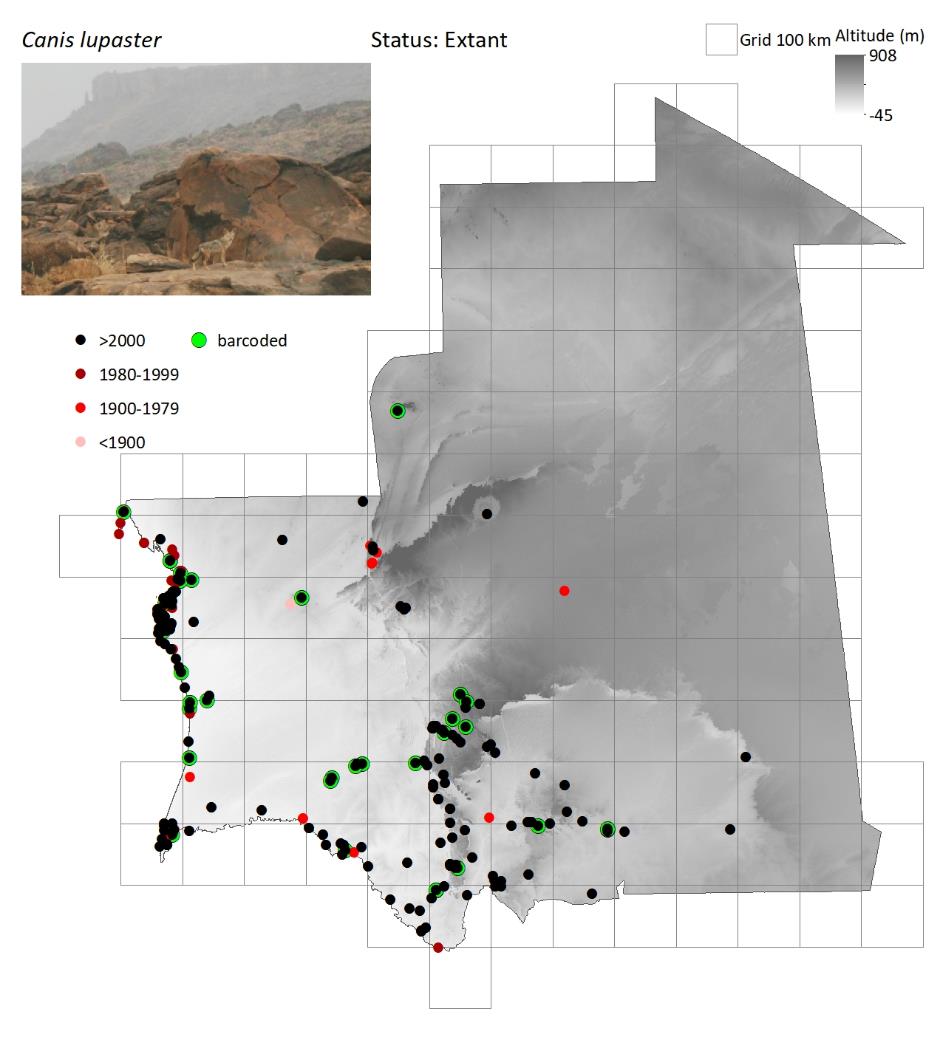


016 - Status and distribution of *Canis lupaster* in Mauritania. Picture in Agnana, Province of Assaba (October 2008).

Comparison with IUCN range polygons: No change. Mapped distribution is similar to reported range in Mauritania.

Conservation status: Global IUCN- LC; National final- LC; National original- LC.


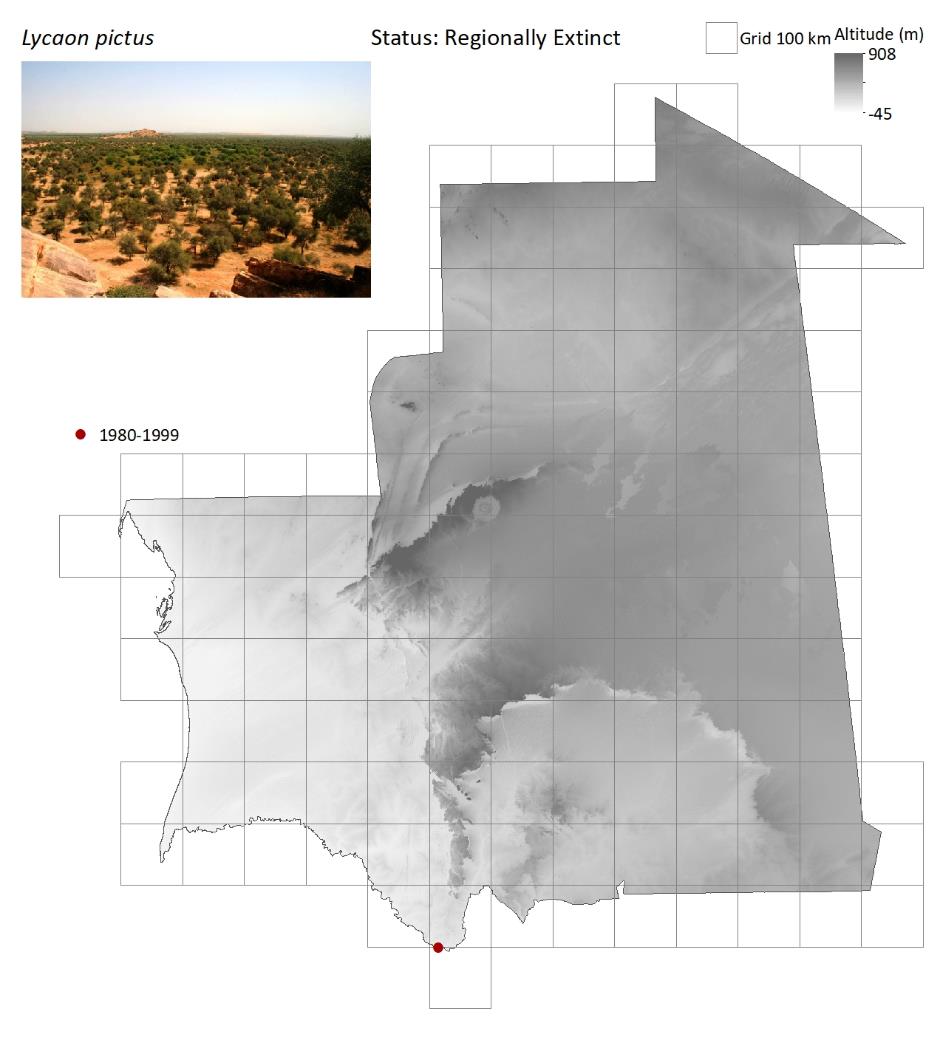


017 - Status and distribution of *Lycaon pictus* in Mauritania. Picture of the habitat in the Senegal River floodplain at Moudéri, Province of Guidimaka (November 2012), where the species was known to occur until the 1990s (Mauritanie 2000 SARL 2001). No observations were made after that period.

Comparison with IUCN range polygons: Not possible. The reported range of the species does not include areas with extinct populations.

Conservation status: Global IUCN- EN; National final- RE; National original- RE.


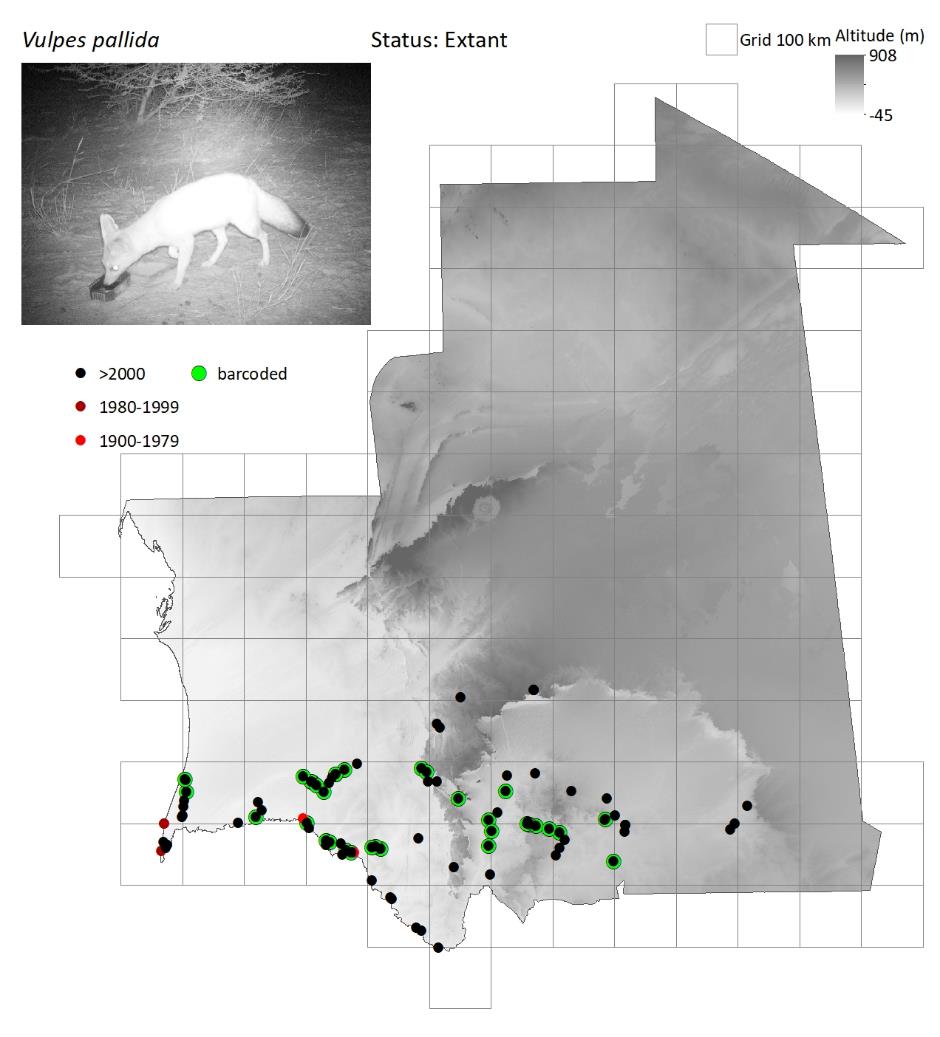


018 - Status and distribution of *Vulpes pallida* in Mauritania. Picture by camera-trapping in Hofret Tadreisa, Province of Hodh El Gharbi (January 2014).

Comparison with IUCN range polygons: No change. Mapped distribution is similar to reported range in Mauritania.

Conservation status: Global IUCN- LC; National final- LC; National original- LC.


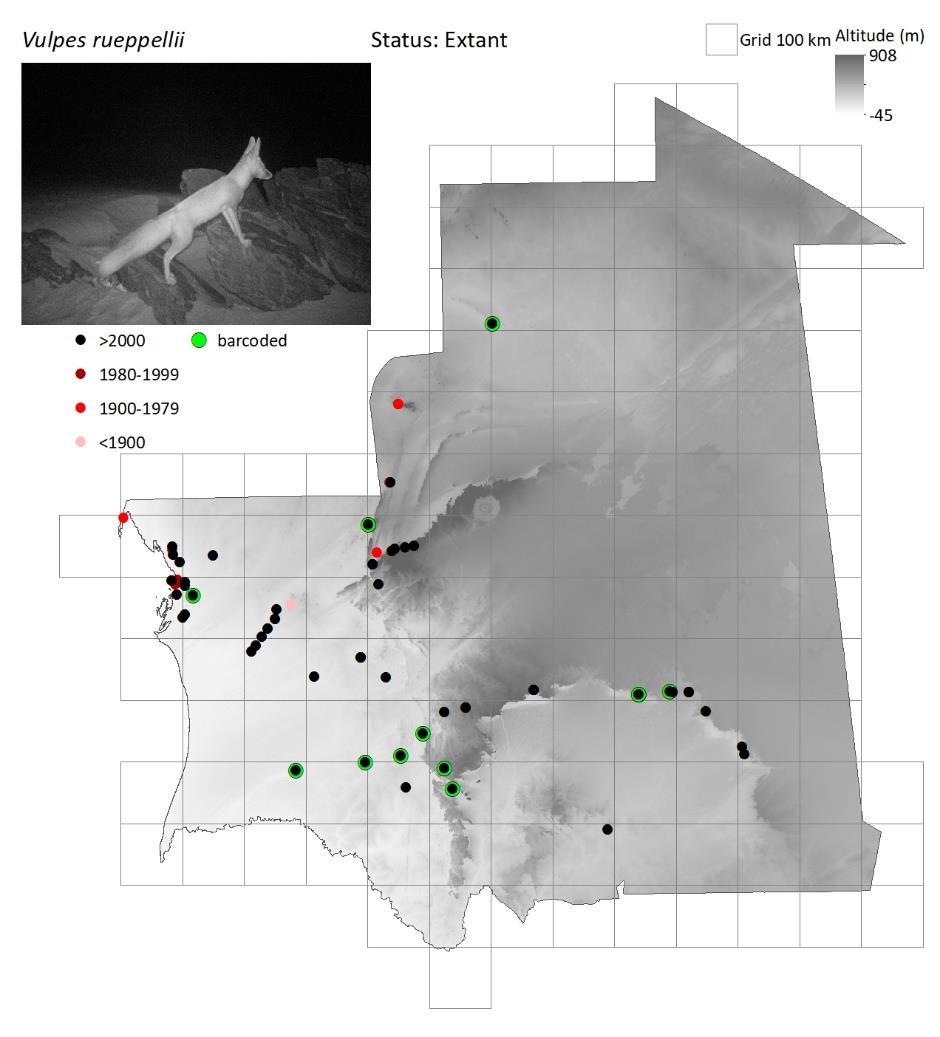


019 - Status and distribution of *Vulpes rueppellii* in Mauritania. Picture by camera-trapping in Bou Naga, Province of Trarza (November 2014).

Comparison with IUCN range polygons: No change. Mapped distribution is similar to reported range in Mauritania.

Conservation status: Global IUCN- LC; National final- LC; National original- LC.


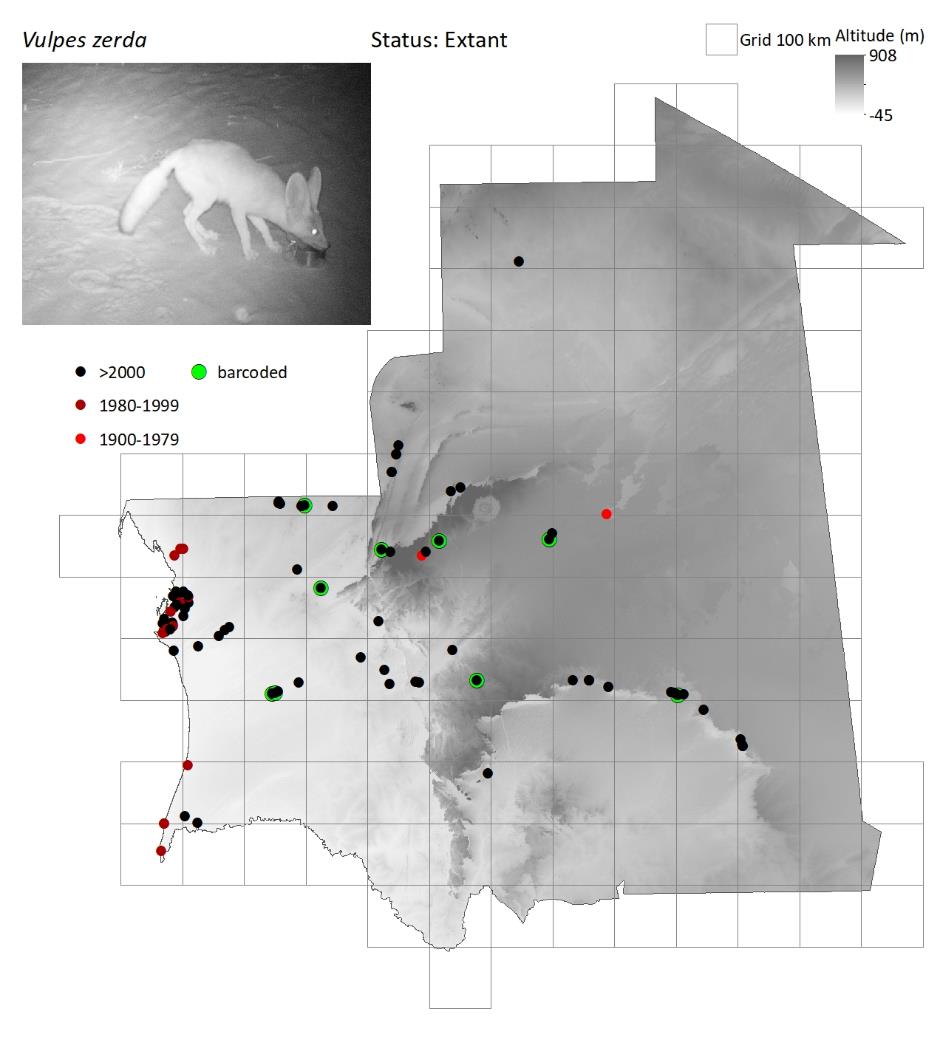


020 - Status and distribution of *Vulpes zerda* in Mauritania. Picture by camera-trapping in Zig, Province of Tagant (February 2014).

Comparison with IUCN range polygons: Better definition of range. The lack of observations along the mid- and upper section of the Senegal River valley and from south-eastern Mauritania suggests that the range of the species in Mauritania is smaller than what is presently known.

Conservation status: Global IUCN- LC; National final- LC; National original- LC.


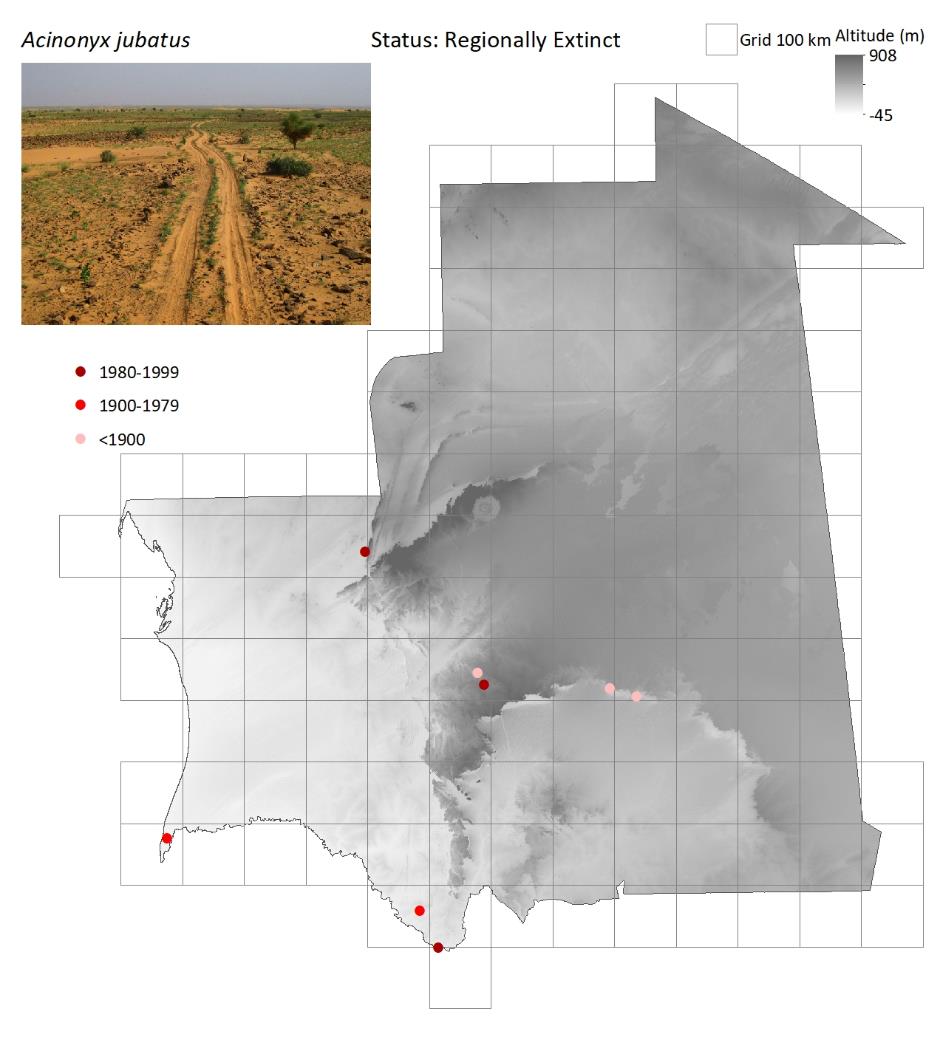


021 - Status and distribution of *Acinonyx jubatus* in Mauritania. Picture of the habitat in the Tagant plateau north of Tidjikja, Province of Tagant (November 2003), where the species was known to occur in the 1970s (Trotignon 1975; Le Berre 1990). No observations were made after that period.

Comparison with IUCN range polygons: Not possible. The reported range of the species does not include areas with extinct populations.

Conservation status: Global IUCN- VU; National final- RE; National original- RE.


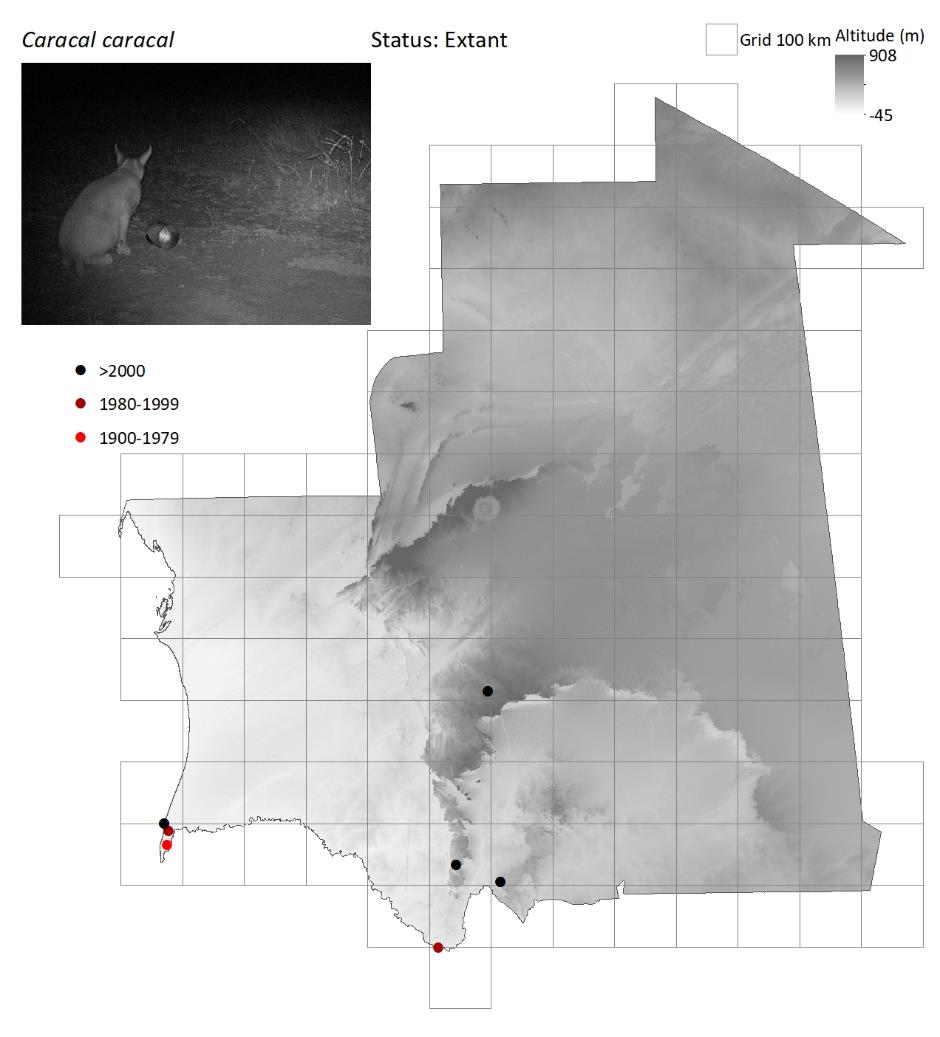


022 - Status and distribution of *Caracal caracal* in Mauritania. Picture bycamera-trapping in *Guelta* El Barda, Province of Assaba (November 2012).

Comparison with IUCN range polygons: Range expansion. The observation in the Tagant plateau expands northwards the reported range of the species in Mauritania.

Conservation status: Global IUCN- LC; National final- NT C2a(i); National original- VU.

Comment on the assessment: Downgraded due to the occurrence of neighbouring populations in Mali and Senegal that may likely provide rescue effect. Inferred continuous population decline due to hunting.


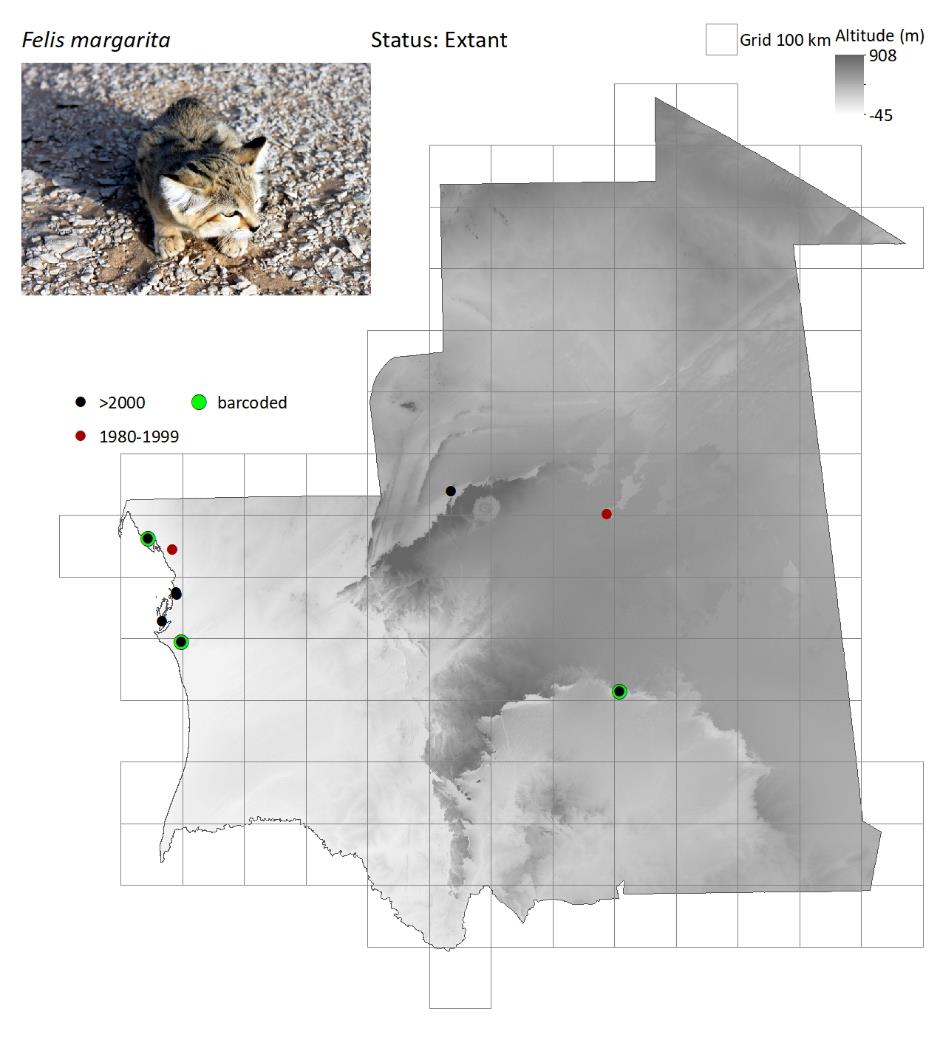


023 - Status and distribution of *Felis margarita* in Mauritania. Picture in the Banc d’Arguin National Park, Province of Dakhlet-Nouâdhibou (December 2011).

Comparison with IUCN range polygons: Range expansion. The observations collected in the Provinces of Adrar and Tagant expand eastwards and southwards, respectively, the reported range of the species in Mauritania.

Conservation status: Global IUCN- LC; National final- LC; National original- LC.


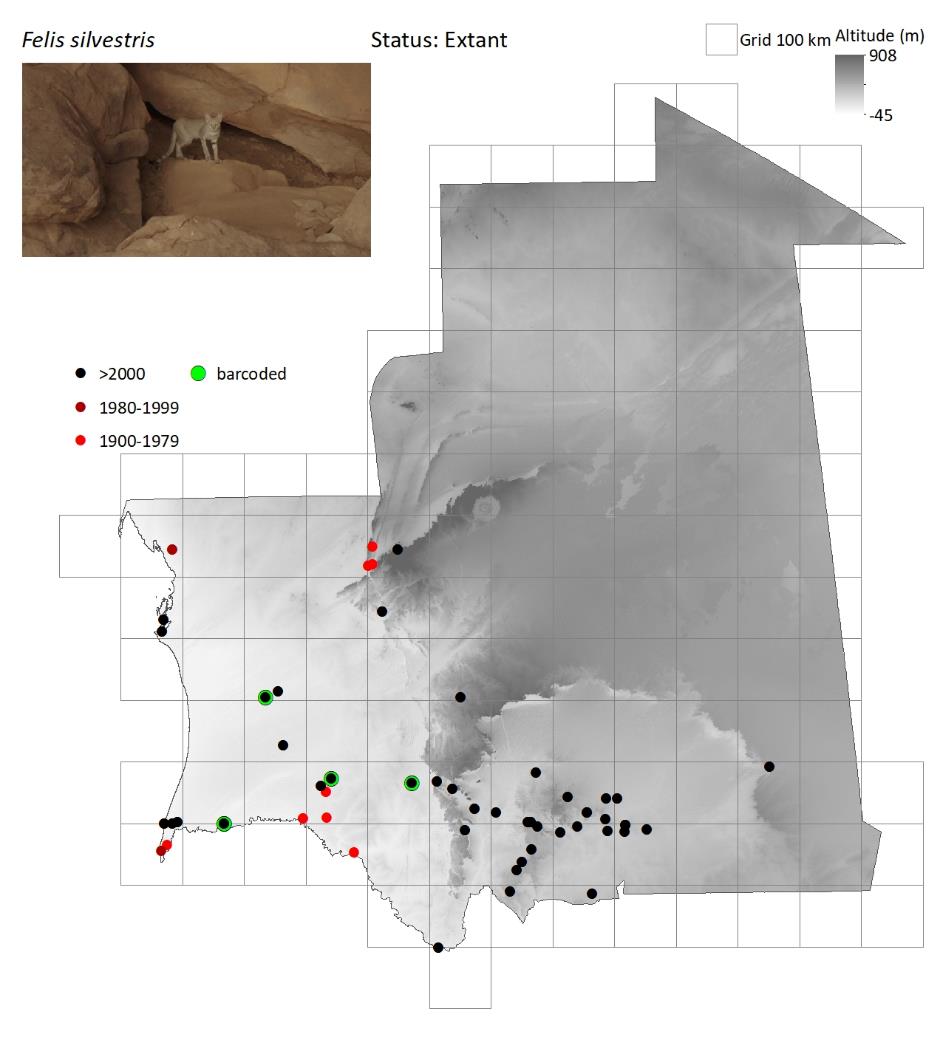


024 - Status and distribution of *Felis silvestris* in Mauritania. Picture near *Guelta* Metraoucha, Province of Hodh El Gharbi (June 2021).

Comparison with IUCN range polygons: Range expansion. The observations collected in the Adrar Atar plateau expand eastwards the reported range of the species in Mauritania.

Conservation status: Global IUCN- LC; National final- LC; National original- LC.


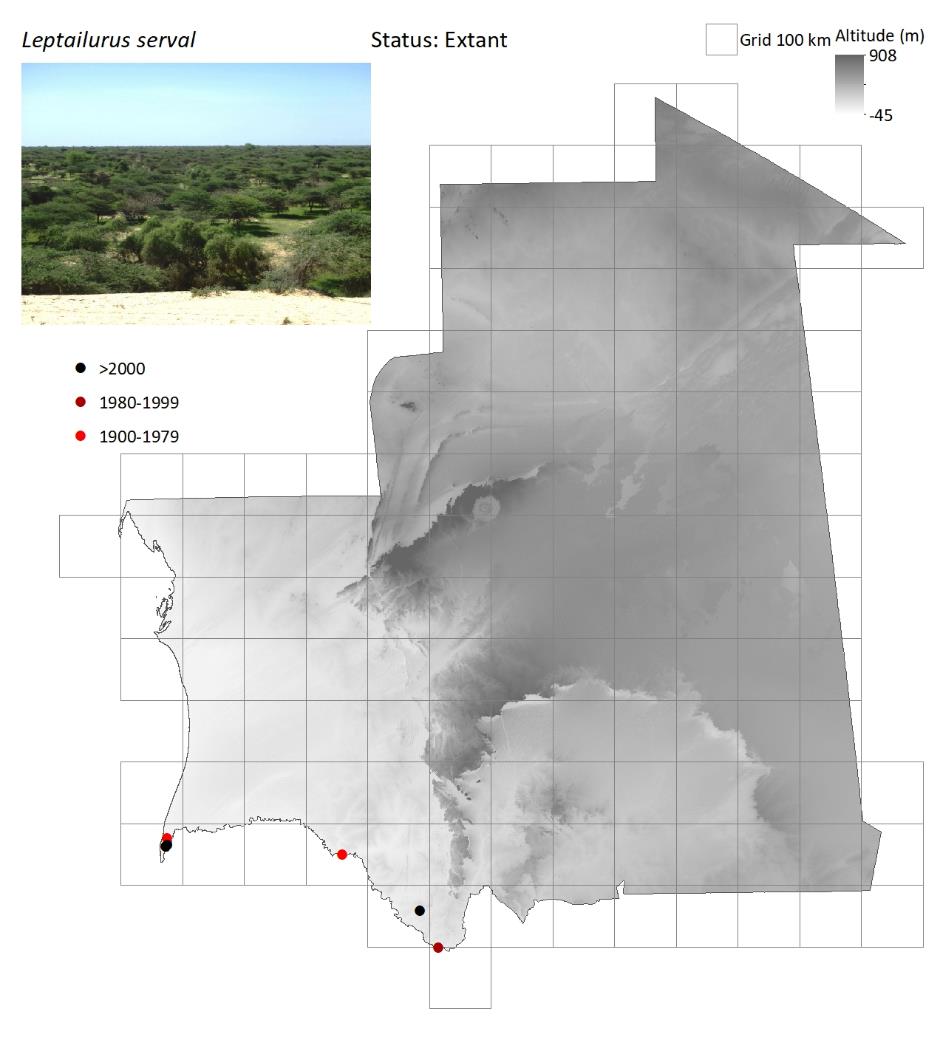


025 - Status and distribution of *Leptailurus serval* in Mauritania. Picture of the habitat in the Diawling National Park, Province of Trarza (August 2015), where an individual was observed by the authors.

Comparison with IUCN range polygons: New species for Mauritania. The observations along the Senegal River valley confirm the occurrence of the species in Mauritania and expand the reported range, as the closest known populations are from the Ferlo region in Senegal.

Conservation status: Global IUCN- LC; National final- VU B1a,b(i,iii); C2a(i); National original- EN.

Comment on the assessment: Downgraded due to the occurrence of neighbouring populations in Mali and Senegal that may likely provide rescue effect. Inferred continuous population decline and decline in area and habitat quality due to persecution, disturbance, logging and wood harvesting, and frequent drought.


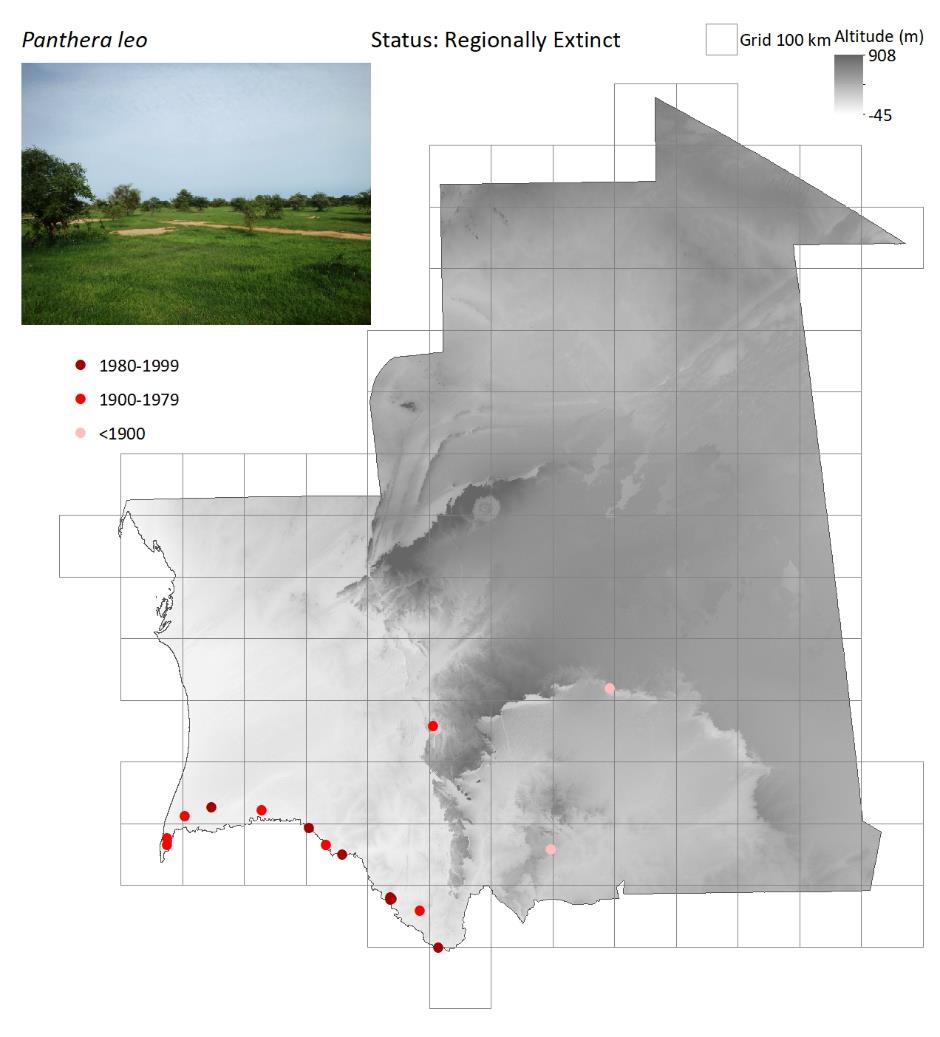


026 - Status and distribution of *Panthera leo* in Mauritania. Picture of the habitat in Arr, Province of Guidimaka (August 2015), where the species was known to occur until the 1990s (Dia 2004; Gueye and Dia 2004). No observations were made after that period.

Comparison with IUCN range polygons: Not possible. The reported range of the species does not include areas with extinct populations.

Conservation status: Global IUCN- VU; National final- RE; National original- RE.


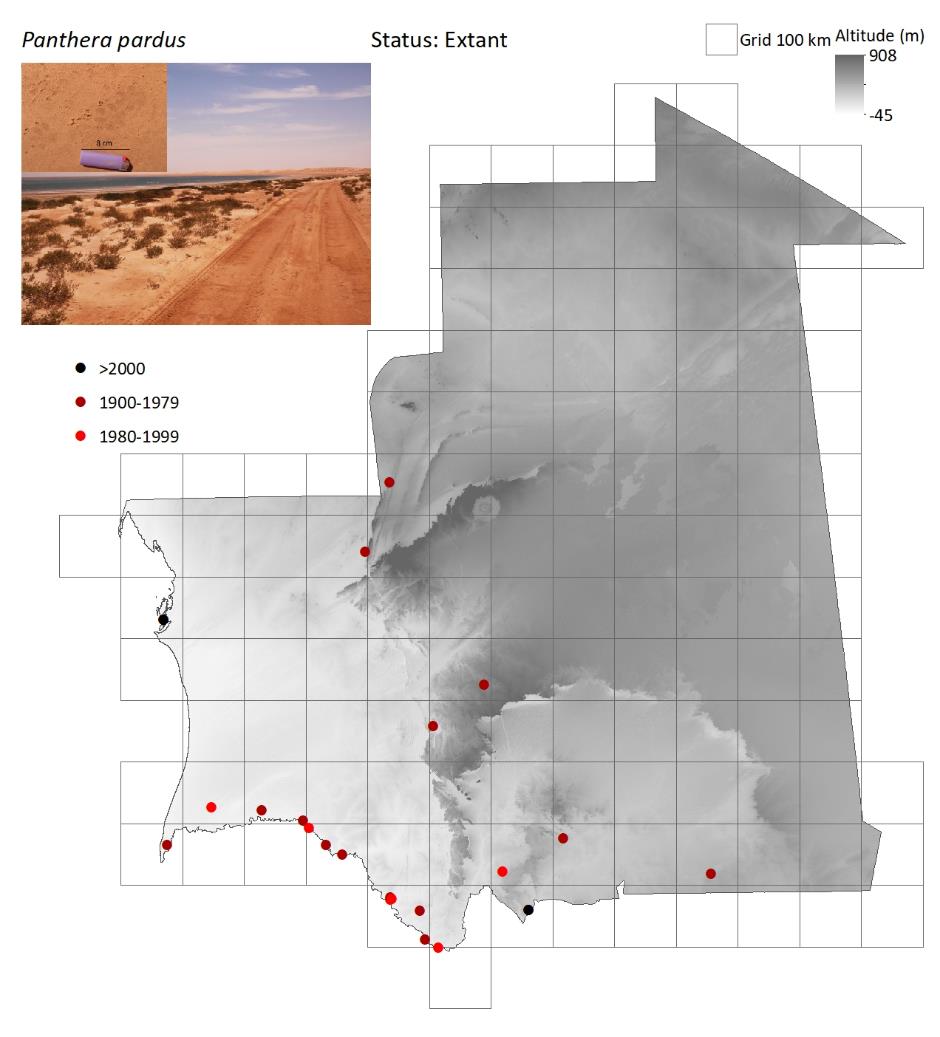


027 - Status and distribution of *Panthera pardus* in Mauritania. Picture of the habitat north of Teichott, Province of Dakhlet-Nouâdhibou (17^th^ May 2009), where an individual was briefly observed by the authours (Jacobson et al 2016). There is an inset picture with the footprints of the observed individual. No observations were made after that date. Another observation (2020) was collected from an interview at Mare de Toya, Province of Assaba, where local people reported occurrence.

Comparison with IUCN range polygons: New species for Mauritania. The observations confirm the current occurrence of the species in Mauritania and expand the reported range, as the closest known populations are from the Niokolo-Koba National Park in Senegal. Still, the limited available observations demand for additional sampling to understand the current range of the species.

Conservation status: Global IUCN- VU; National final- CR C2a(i); D; National original- CR.

Comment on the assessment: Downgrading excluded due lack of sufficient evidence proving rescue effect from neighbouring populations in Mali and Senegal**.** Inferred continuous population decline due to hunting and habitat disturbance.


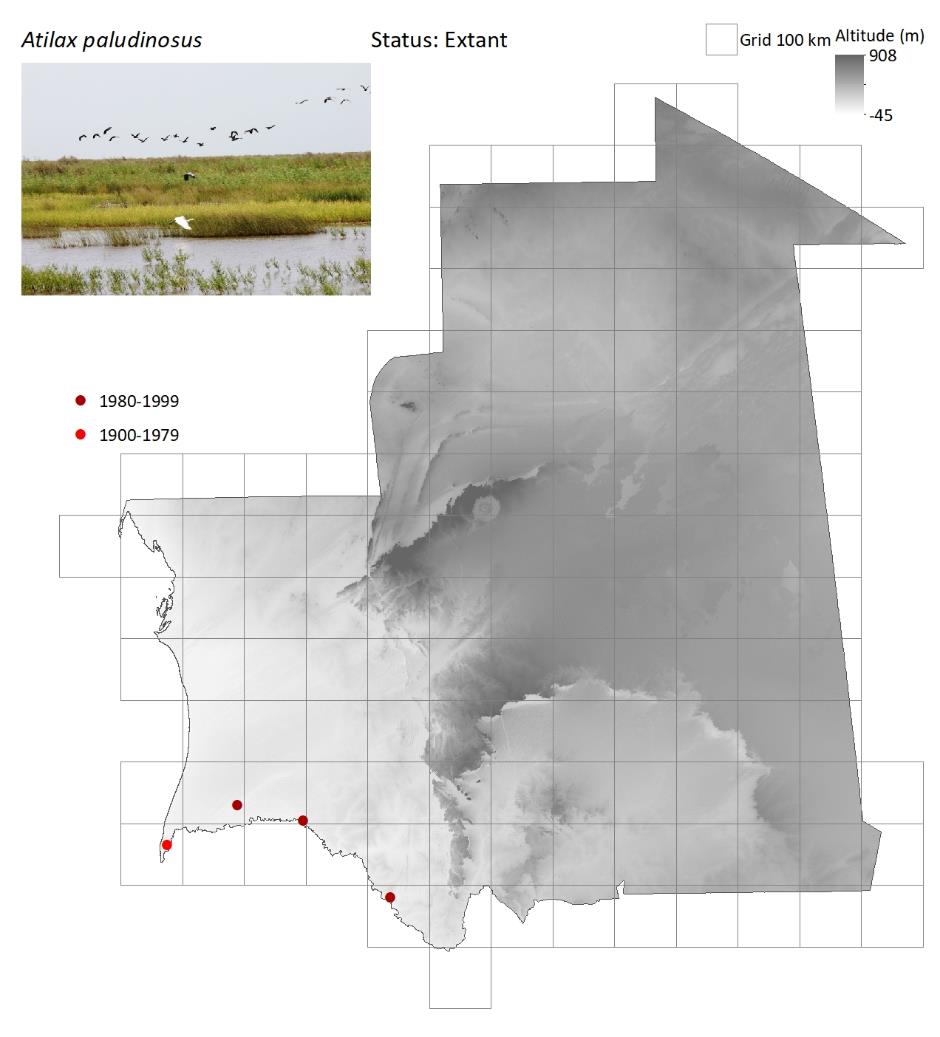


028 - Status and distribution of *Atilax paludinosus* in Mauritania. Picture of the habitat near Keur Macene, Province of Trarza (November 2012), where the species was reported to occur in the 1980s (National Research Council 1981; Hughes et al 1992). No observations were made after that date.

Comparison with IUCN range polygons: Range expansion. The observations along the Senegal River valley expand westwards the reported range of the species in Mauritania. Still, observations are from before the year 2000, and additional sampling is needed to understand the current range of the species.

Conservation status: Global IUCN- LC; National final- DD; National original- DD.

Comment on the assessment: There are only four observations available from before the year 2000.


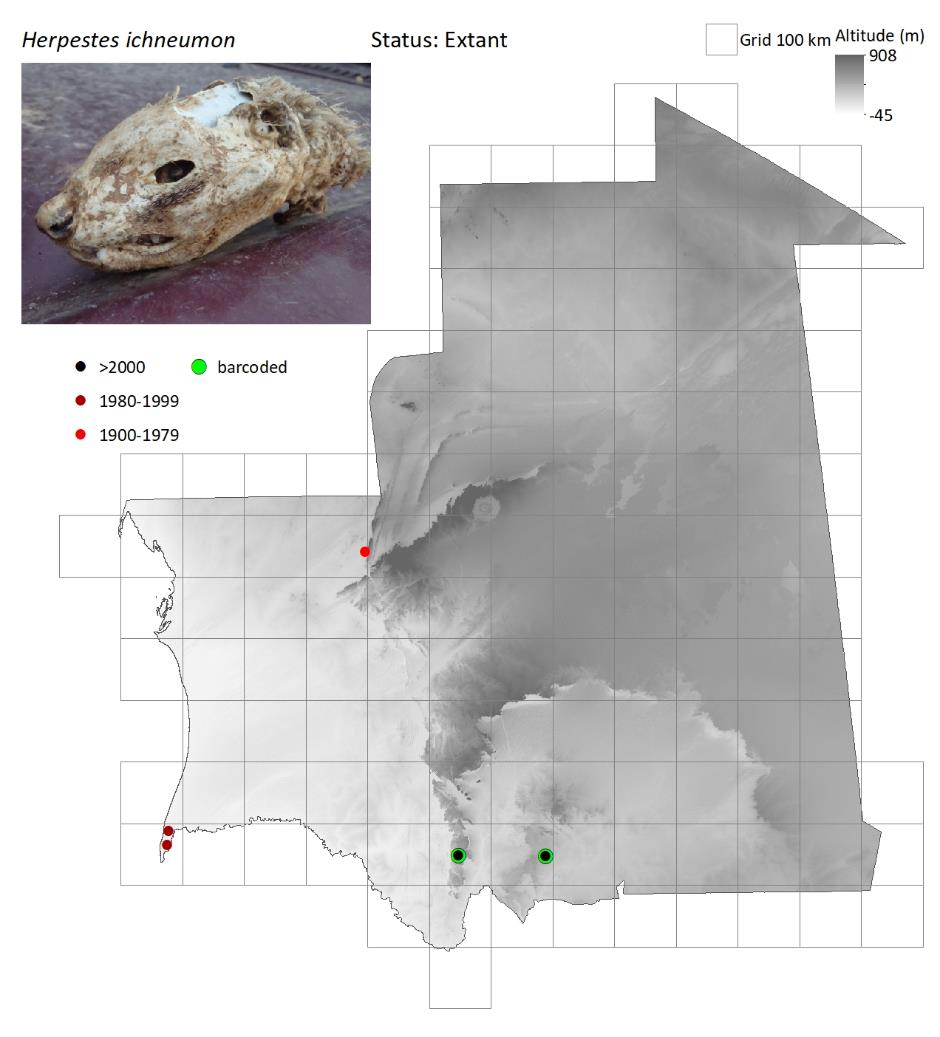


029 - Status and distribution of *Herpestes ichneumon* in Mauritania. Picture of a skull confirmed by barcoding found in Djafarat, Province of Assaba (November 2014).

Comparison with IUCN range polygons: Range expansion. The observations in the Assaba and Afollé plateaus expand northwards the reported range of the species in Mauritania.

Conservation status: Global IUCN- LC; National final- LC; National original- LC.


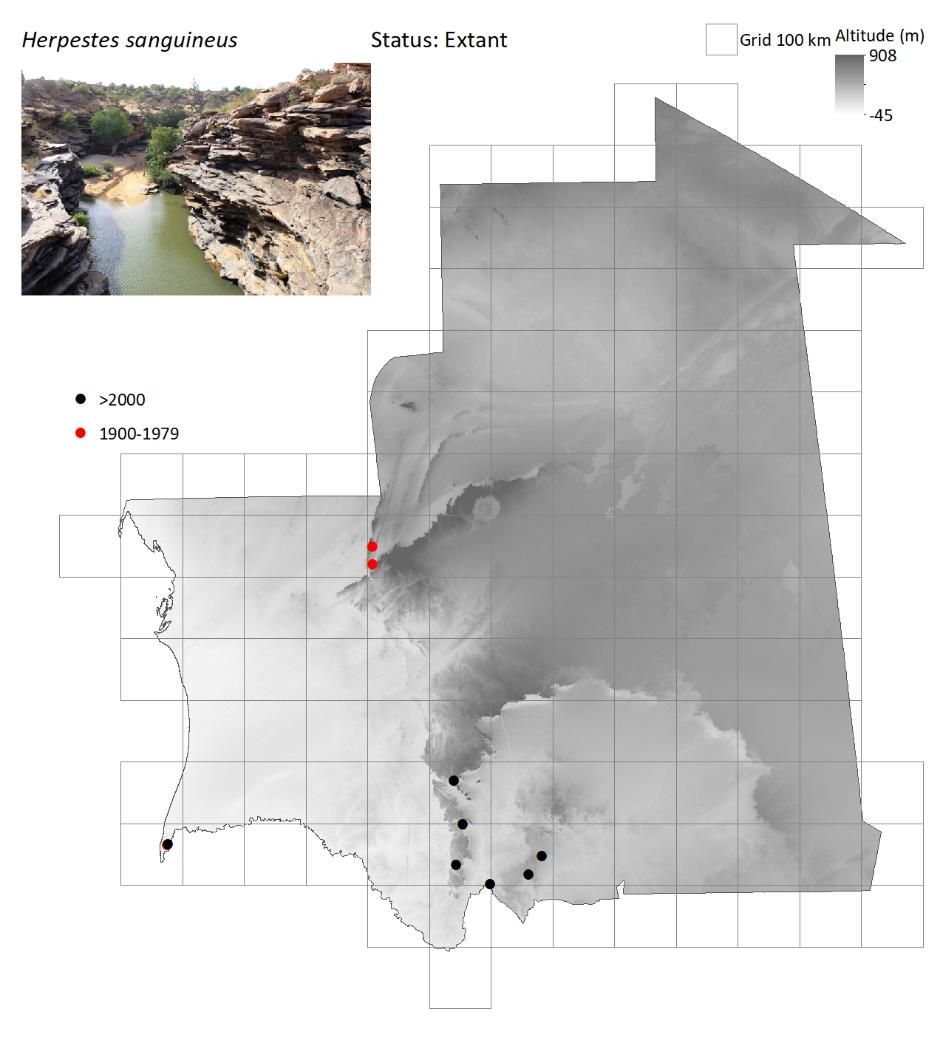


030 - Status and distribution of *Herpestes sanguineus* in Mauritania. Picture of the habitat at *Guelta* El Barda, Province of Assaba (November 2012), where the species was observed by the authors.

Comparison with IUCN range polygons: Range expansion. The observations in Diawling National Park and in the Adrar Atar expand westwards and northwards the reported range of the species in Mauritania. However, the observations in the Adrar Atar were collected in the 1950s (Dekeyser and Villiers 1956), and they may represent misidentifications with *Herpestes ichneumon.* Additional sampling is needed to understand the current range of the species.

Conservation status: Global IUCN- LC; National final- LC; National original- LC.


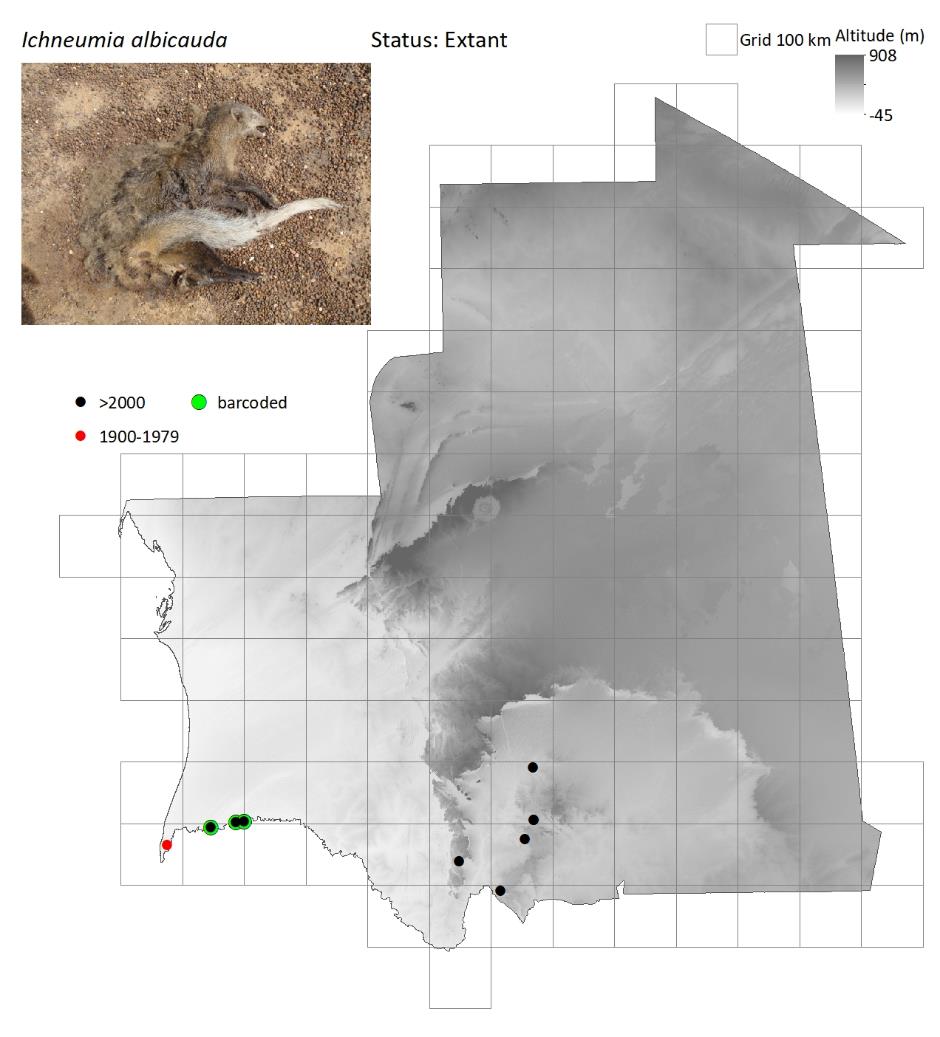


031 - Status and distribution of *Ichneumia albicauda* in Mauritania. Picture of a roadkill specimen confirmed by barcoding found near Garak, Province of Trarza (November 2012).

Comparison with IUCN range polygons: Range expansion. The observations in the Afollé plateau expand northwards the reported range of the species in Mauritania.

Conservation status: Global IUCN- LC; National final- LC; National original- LC.


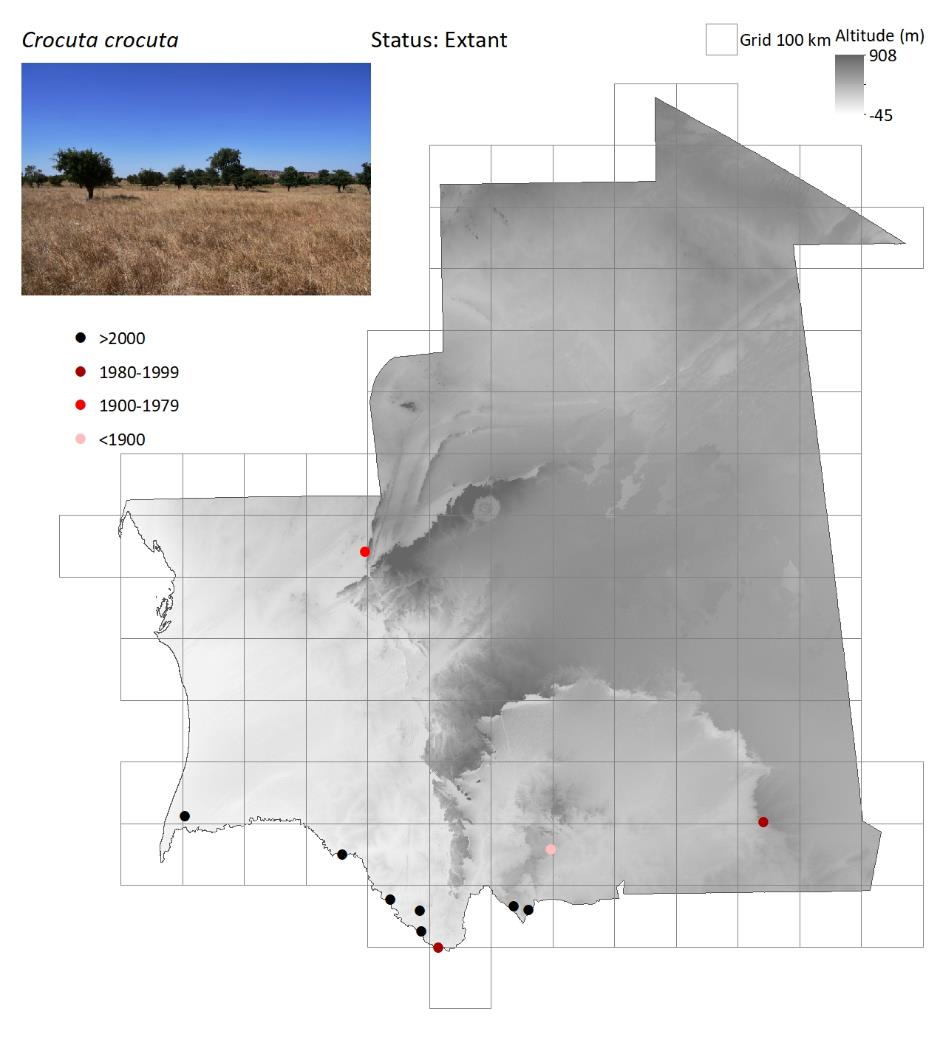


032 - Status and distribution of *Crocuta crocuta* in Mauritania. Picture of the habitat in Chkata, Province of Assaba (November 2020), where interviews to local people reported the occurrence of the species.

Comparison with IUCN range polygons: No change. Mapped distribution is similar to reported range in Mauritania.

Conservation status: Global IUCN- LC; National final- VU C2a(i); National original- EN.

Comment on the assessment: Downgraded due to the occurrence of neighbouring populations in Mali and Senegal that may likely provide rescue effect. Inferred continuous population decline due to killing by poisoning.


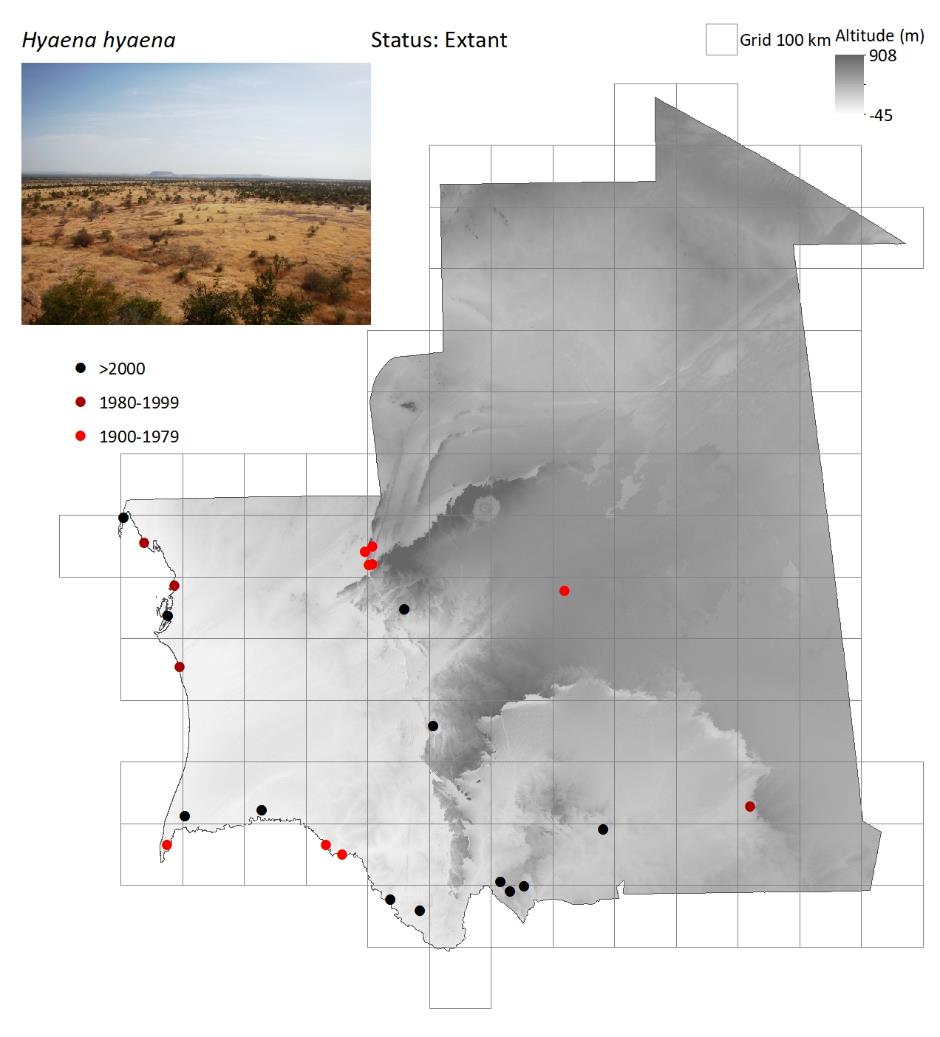


033 - Status and distribution of *Hyaena hyaena* in Mauritania. Picture from November 2014 of the habitat in Maoudass, Province of Assaba, where interviews to local people reported the occurrence of the species.

Comparison with IUCN range polygons: No change. Mapped distribution is similar to reported range in Mauritania.

Conservation status: Global IUCN- NT; National final- NT C2a(i); National original- VU.

Comment on the assessment: Downgraded due to the occurrence of neighbouring populations in Algeria, Mali, Morocco and Senegal that may likely provide rescue effect. Inferred continuous population decline due to killing by poisoning.


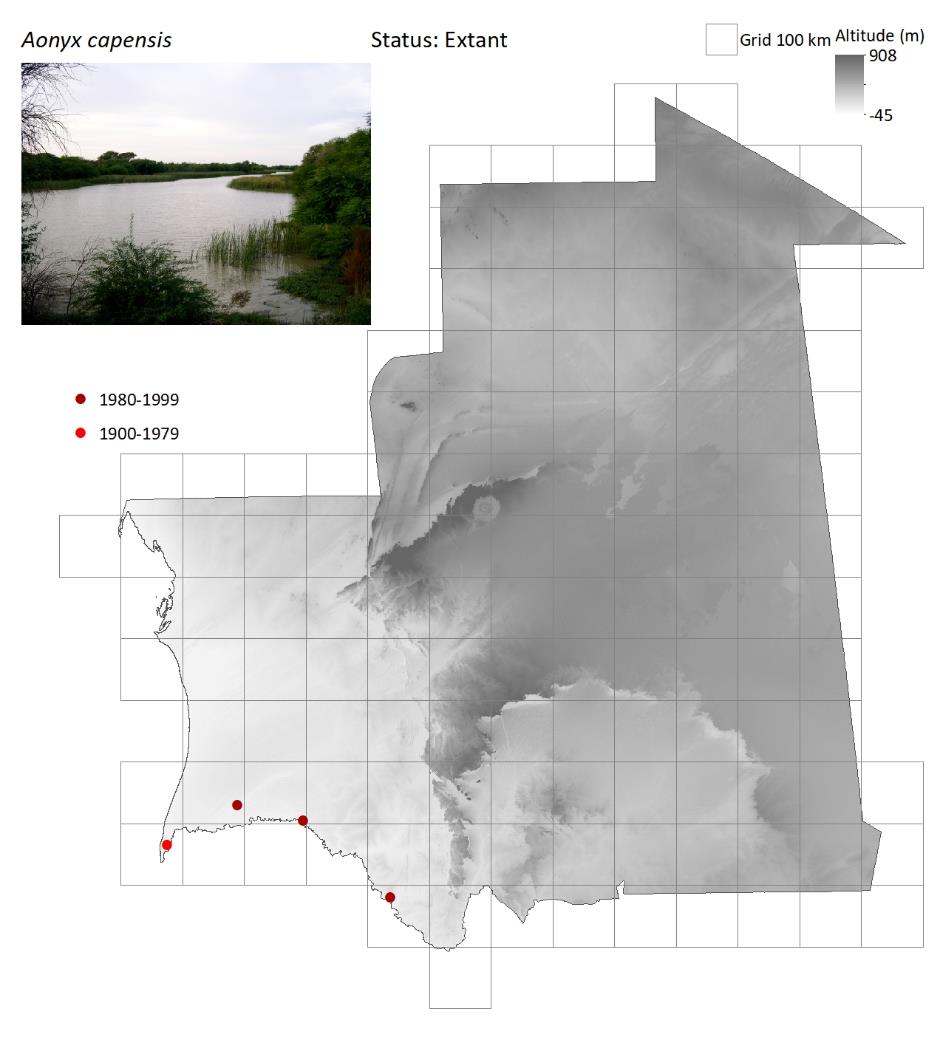


034 - Status and distribution of *Aonyx capensis* in Mauritania. Picture of the habitat in R’Kiz lake, Province of Trarza (December 2007), where the species has been reported (Hughes et al 1992).

Comparison with IUCN range polygons: No change. Mapped distribution is similar to reported range in Mauritania.

Conservation status: Global IUCN- LC; National final- DD; National original- DD.

Comment on the assessment: There are only four observations available from before the year 2000.


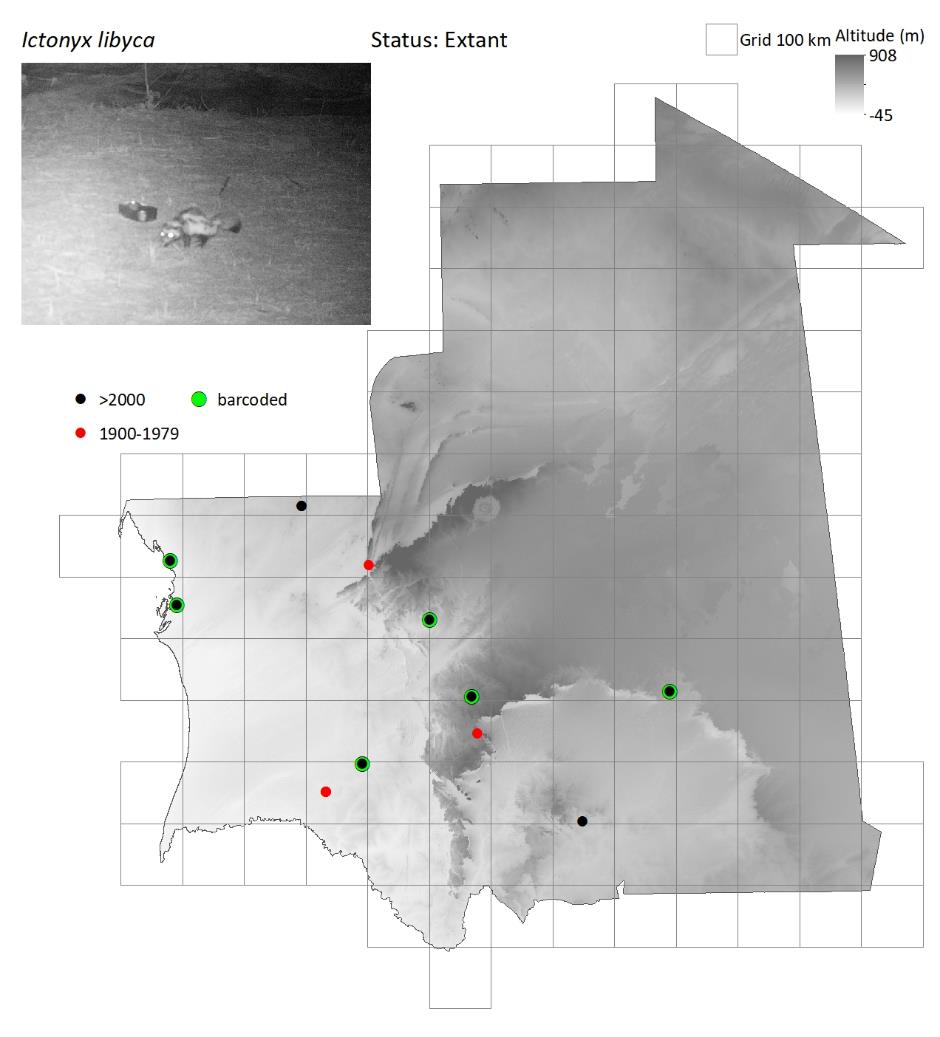


035 - Status and distribution of *Ictonyx libyca* (=*Ictonyx libycus*) in Mauritania. Picture by camera-trapping in tâmoûrt El Khcheb, Province of Trarza (November 2014).

Comparison with IUCN range polygons: No change. Mapped distribution is similar to reported range in Mauritania.

Conservation status: Global IUCN- LC; National final- LC; National original- LC.


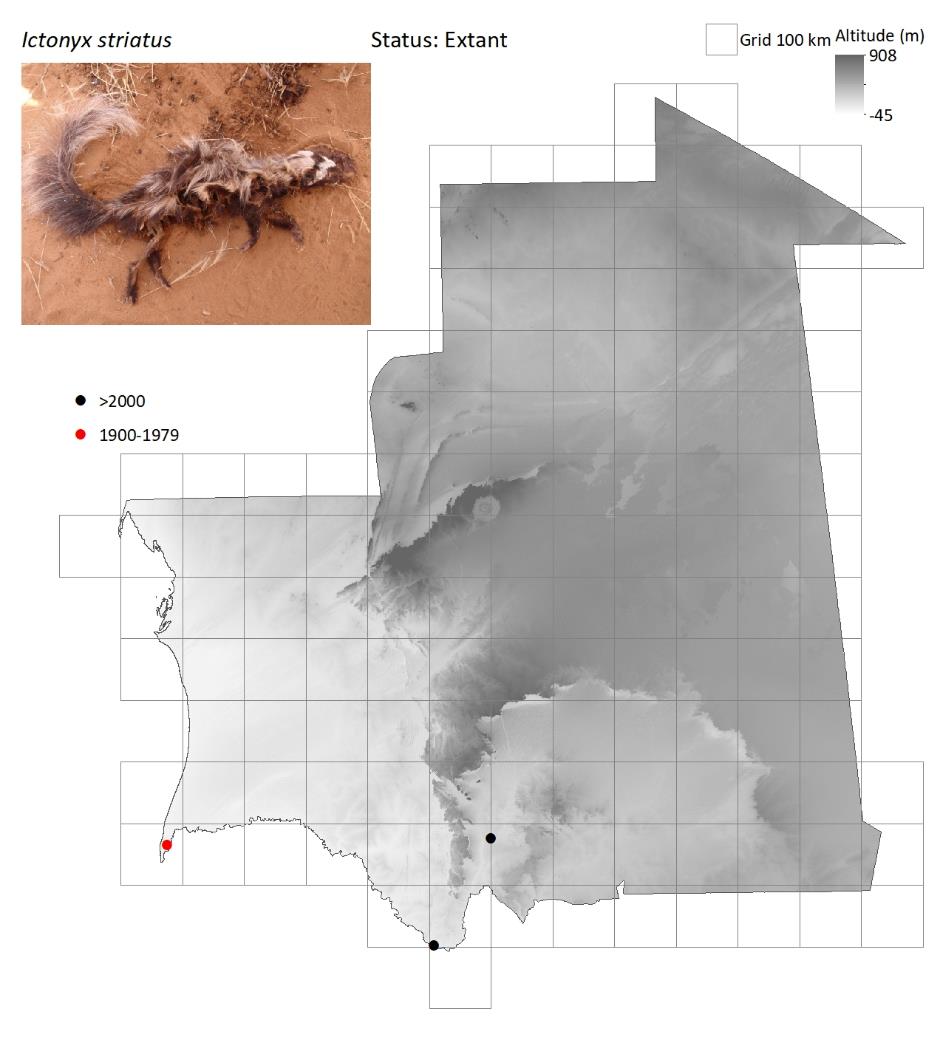


036 - Status and distribution of *Ictonyx striatus* in Mauritania. Picture of a roadkill specimen found near Kiffa, Province of Assaba (November 2008).

Comparison with IUCN range polygons: No change. Mapped distribution is similar to reported range in Mauritania.

Conservation status: Global IUCN- LC; National final- DD; National original- DD.

Comment on the assessment: There are only three observations available, two of them from after the year 2000.


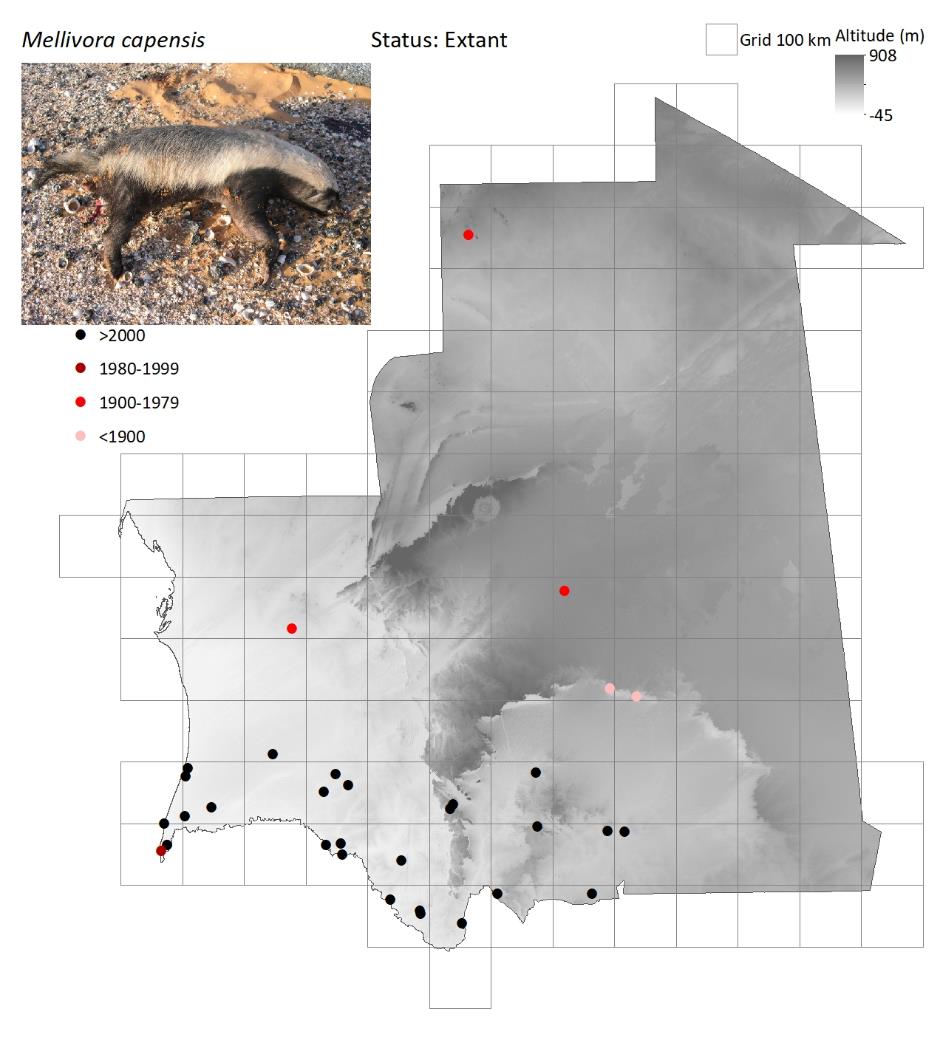


037 - Status and distribution of *Mellivora capensis* in Mauritania. Picture of a roadkill specimen found near Boutilimit, Province of Trarza (November 2003).

Comparison with IUCN range polygons: Better definition of range. The lack of observations along eastern regions of the country suggests that the range of the species in Mauritania may be smaller than what is presently known.

Conservation status: Global IUCN- LC; National final- LC; National original- LC.


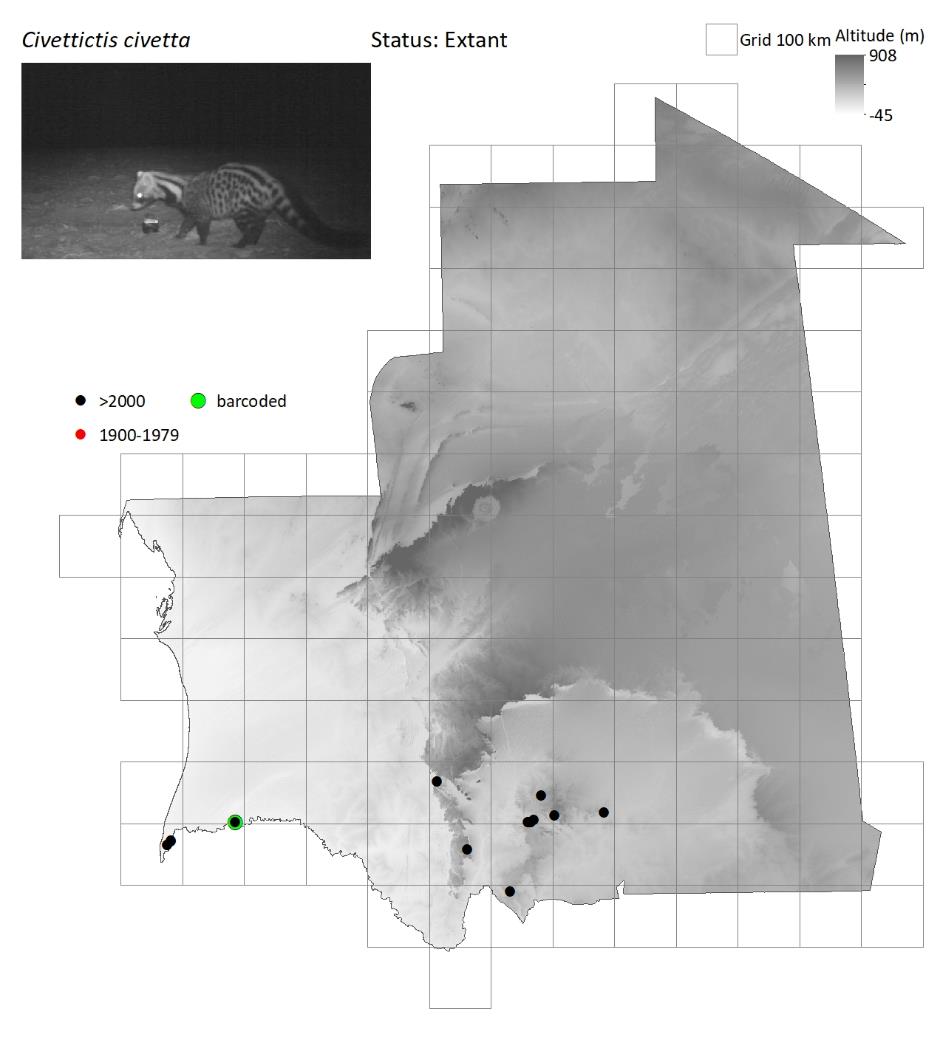


038 - Status and distribution of *Civettictis civetta* in Mauritania. Picture by camera-trapping near *Guelta* Oumm el Mhâr, Province of Hodh El Gharbi (November 2011).

Comparison with IUCN range polygons: Range expansion. The observations along the Senegal River valley and in northern Assaba plateau expand northwards the reported range of the species in Mauritania.

Conservation status: Global IUCN- LC; National final- LC; National original- LC.


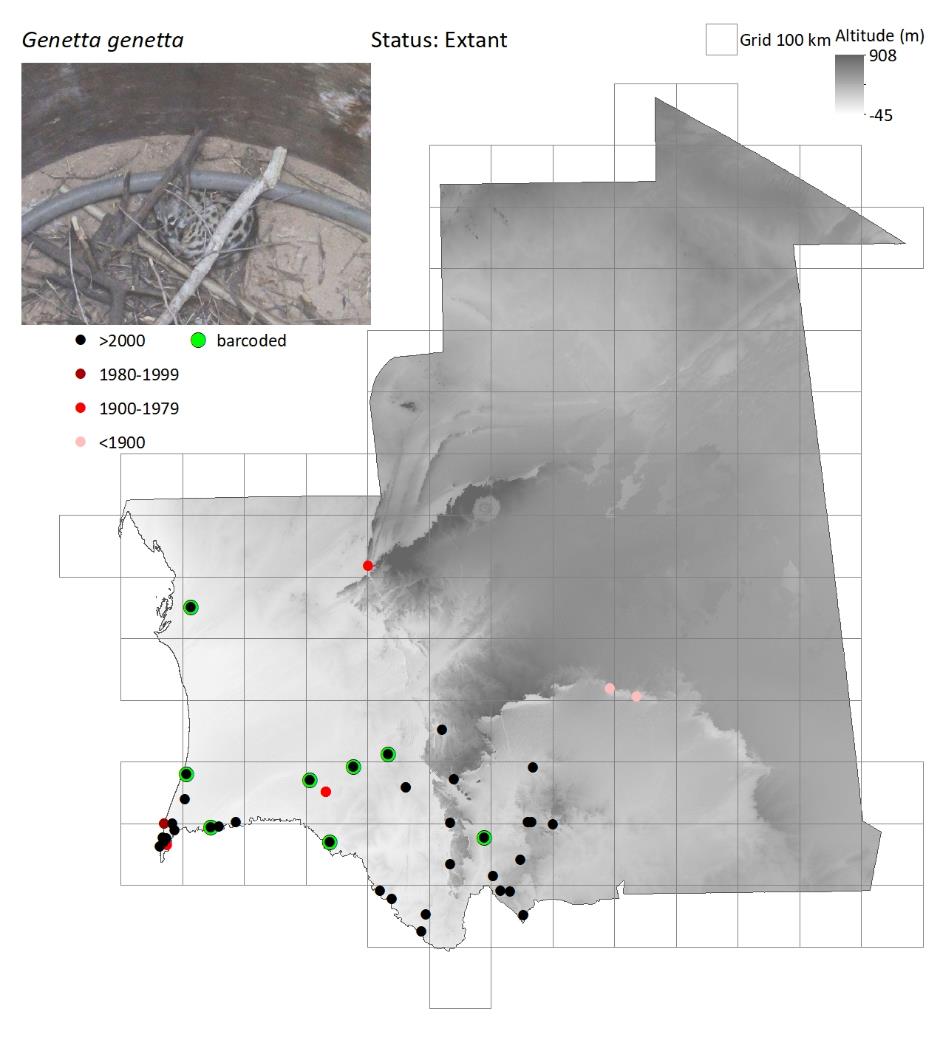


039 - Status and distribution of *Genetta genetta* in Mauritania. Picture of specimen found trapped inside a dry well in the Diawling National Park, Province of Trarza (February 2016). The specimen was captured and released outside the well (<https://www.youtube.com/watch?v=I7dr1PvRRQ8>).

Comparison with IUCN range polygons: No change. Mapped distribution is similar to reported range in Mauritania.

Conservation status: Global IUCN- LC; National final- LC; National original- LC.


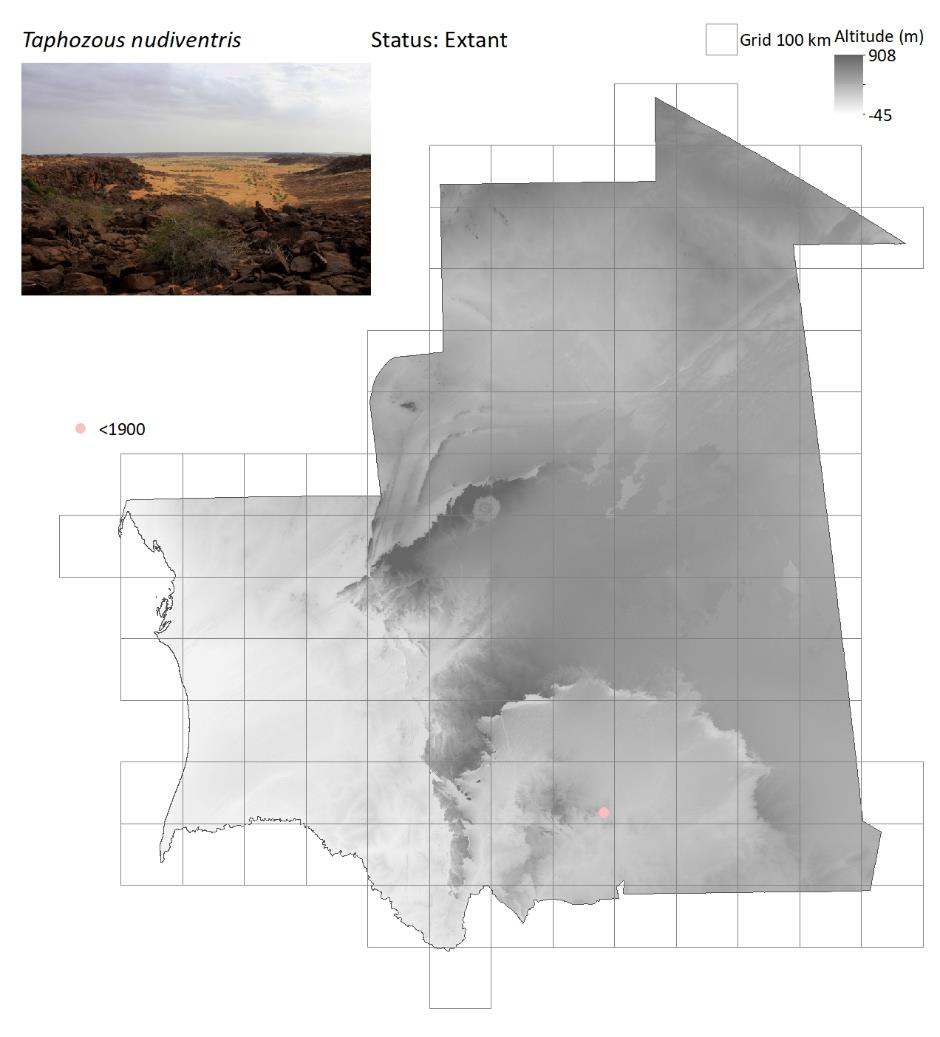


040 - Status and distribution of *Taphozous nudiventris* in Mauritania. Picture of the habitat near Ayoun el Atouss, Province of Hodh El Gharbi (November 2014), where the species has been reported before the 1990s (GBIF.org 2021).

Comparison with IUCN range polygons: Uncertainty. The observations near Ayoun el Atrouss apparently expand eastwards the reported range of the species in Mauritania. These observations correspond to two vouchers (Natural History Museum of Genève: MAM-1885.080 -081; GBIF.org 2021). However, the species is referred as occurring in “Mauritania” without a precise locality by the African Chiroptera Report (ACR 2020) and the range polygon from the IUCN Red List (IUCN 2021) depicts species occurrence in Mauritania but only in the western region (Province of Trarza). Additional sampling is needed to understand the current status and range of the species.

Conservation status: Global IUCN- LC; National final- DD; National original- DD.

Comment on the assessment: There are only two observations available from before the year 1900 and only one of them has precise geographic coordinates.


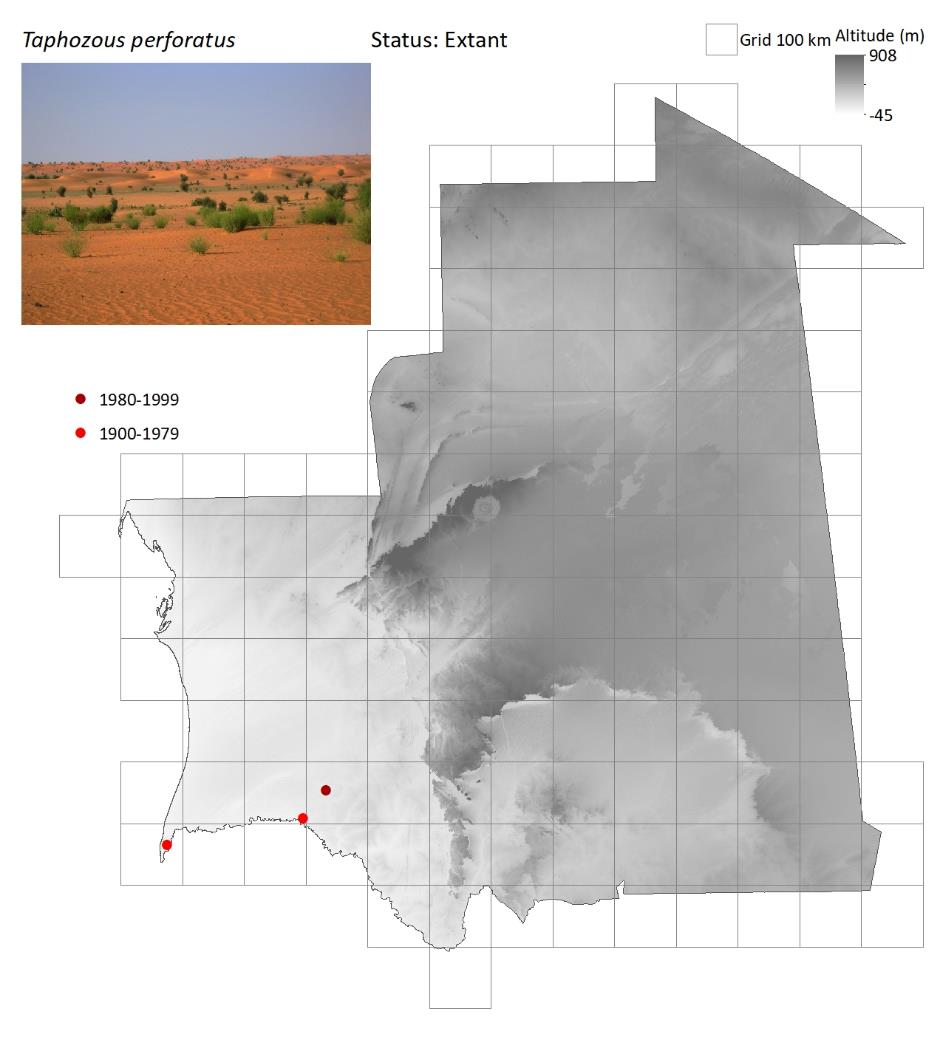


041 - Status and distribution of *Taphozous perforatus* in Mauritania. Picture of the habitat near Aleg, Province of Brakna (November 2003), where the species has been reported (ACR 2020).

Comparison with IUCN range polygons: Range expansion. The observations Senegal River delta expand westwards the reported range of the species in Mauritania.

Conservation status: Global IUCN- LC; National final- NT B1a; National original- NT.

Comment on the assessment: Downgrading excluded due lack of evidence proving rescue effect from neighbouring populations in Senegal. Reduced extent of occurrence but lack of evidence on continuous decline in extent or habitat quality.


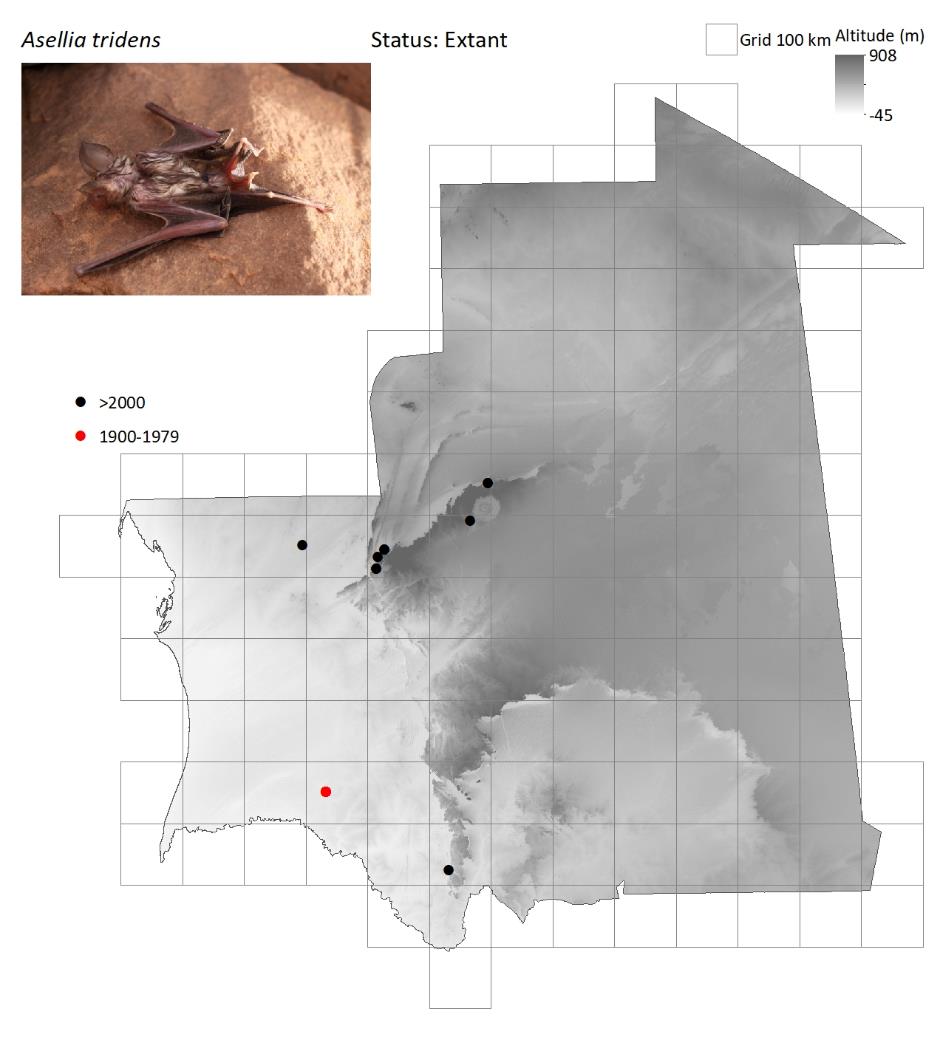


042 - Status and distribution of *Asellia tridens* in Mauritania. Picture of specimen found dead in El Beyyed, Province of Tiris Zemmour (August 2012).

Comparison with IUCN range polygons: No change. Mapped distribution is similar to reported range in Mauritania.

Conservation status: Global IUCN- LC; National final- LC; National original- LC.


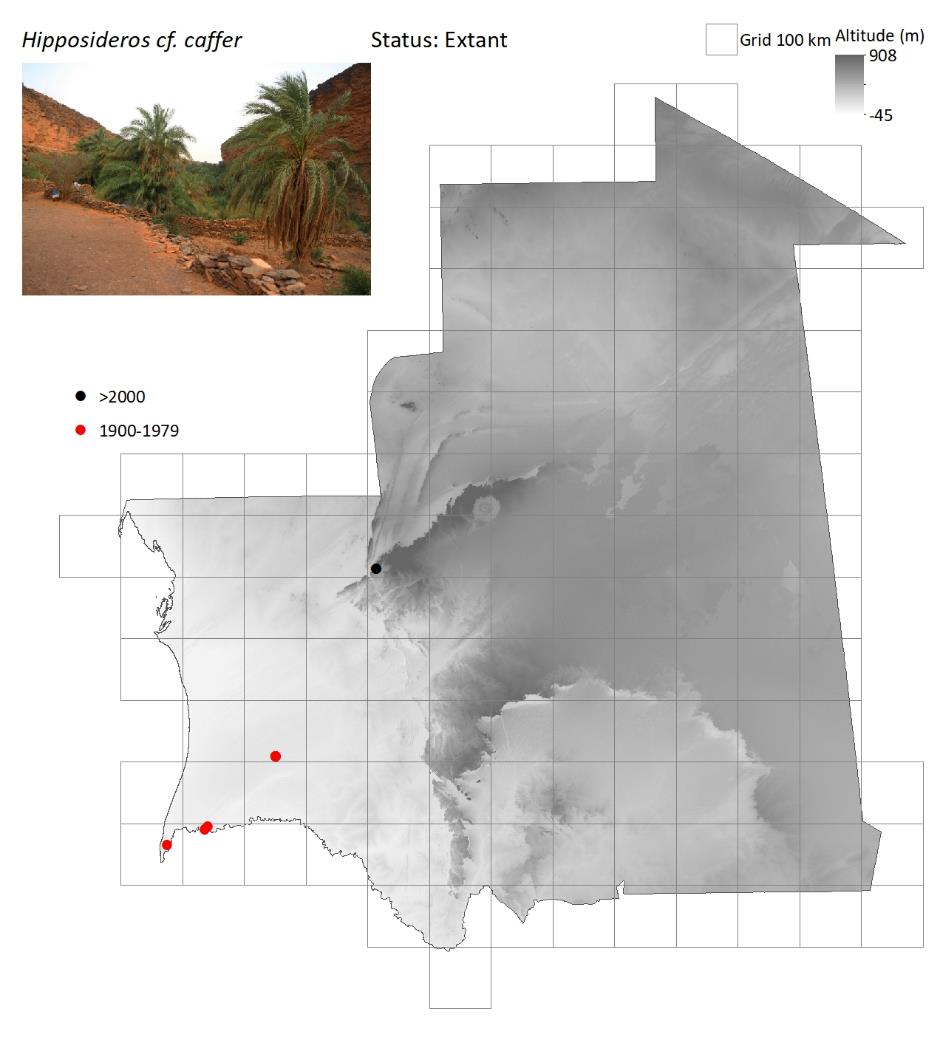


043 - Status and distribution of *Hipposideros cf. caffer* in Mauritania. Picture of the habitat in Terjît oasis, Province of Adrar (September 2008), where the species has been recently reported (Padial and Ibáñez 2005; ACR 2020).

Comparison with IUCN range polygons: Uncertainty. According to the Mammal Species of the World (Wilson and Reeder 2005) and the IUCN Red List the species is known from Southern Africa (IUCN 2021), while the African Chiroptera Report lists several vouchers collected in Mauritania (ACR 2020; GBIF.org 2021), and there are several published observations (Padial and Ibáñez 2005; Qumseyeh and Schütter 1981) attributed to the species. Molecular barcoding and an assessment of the systematics and taxonomy are needed to clear the taxonomic status of the species.

Conservation status: Global IUCN- NE; National final- NT B1a; National original- NT.

Comment on the assessment: Downgrading excluded due lack of sufficient evidence proving rescue effect from neighbouring populations in Senegal. Reduced extent of occurrence but lack of evidence on continuous decline in range extent or habitat quality.


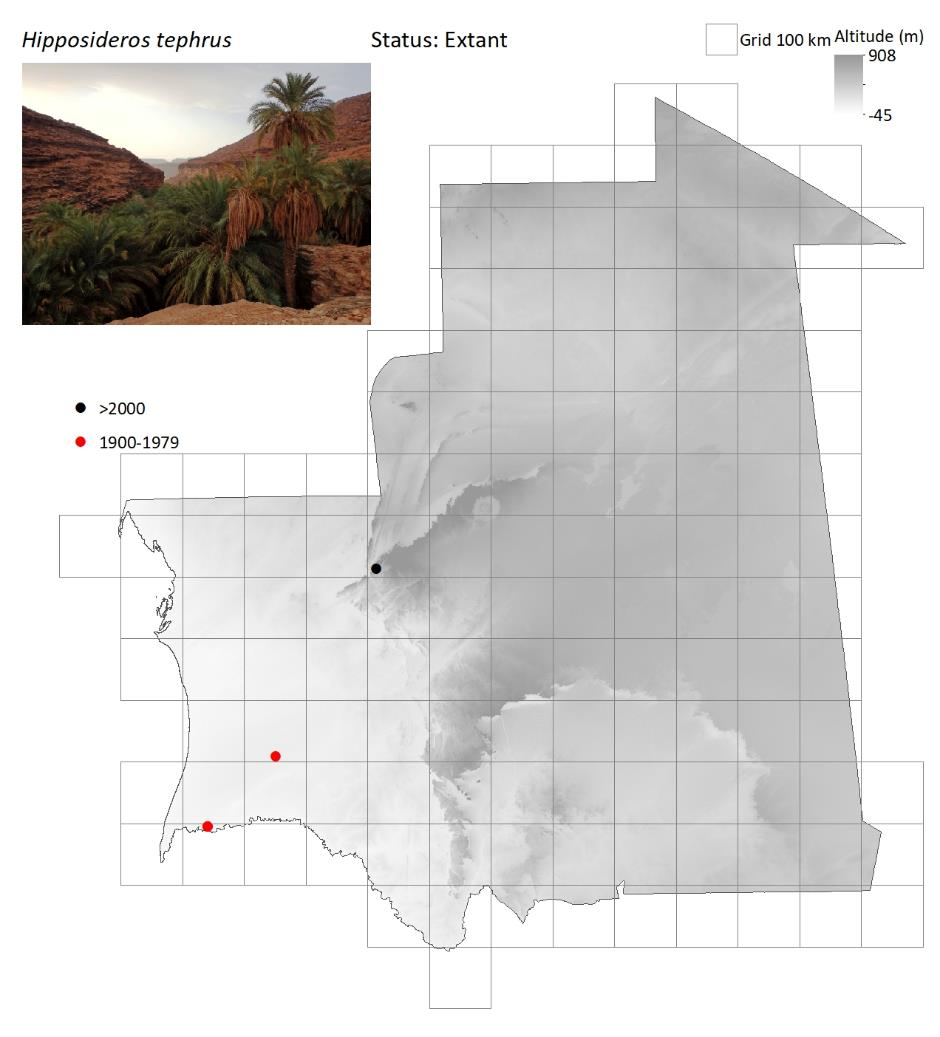


044 - Status and distribution of *Hipposideros tephrus* in Mauritania. Picture of the habitat in Terjît oasis, Province of Adrar (September 2015), where the species has been reported (Allegrini et al 2011; ACR 2020).

Comparison with IUCN range polygons: New species for Mauritania. The observations confirm the occurrence of the species in Mauritania and expand the reported range, as the closest known populations are from the Gambia River valley in Senegal and The Gambia.

Conservation status: Global IUCN- LC; National final- LC; National original- NT B1a.

Comment on the assessment: Downgraded due to the occurrence of neighbouring populations in Morocco and Senegal that may likely provide rescue effect. Reduced extent of occurrence but lack of evidence on continuous decline in range extent or habitat quality.


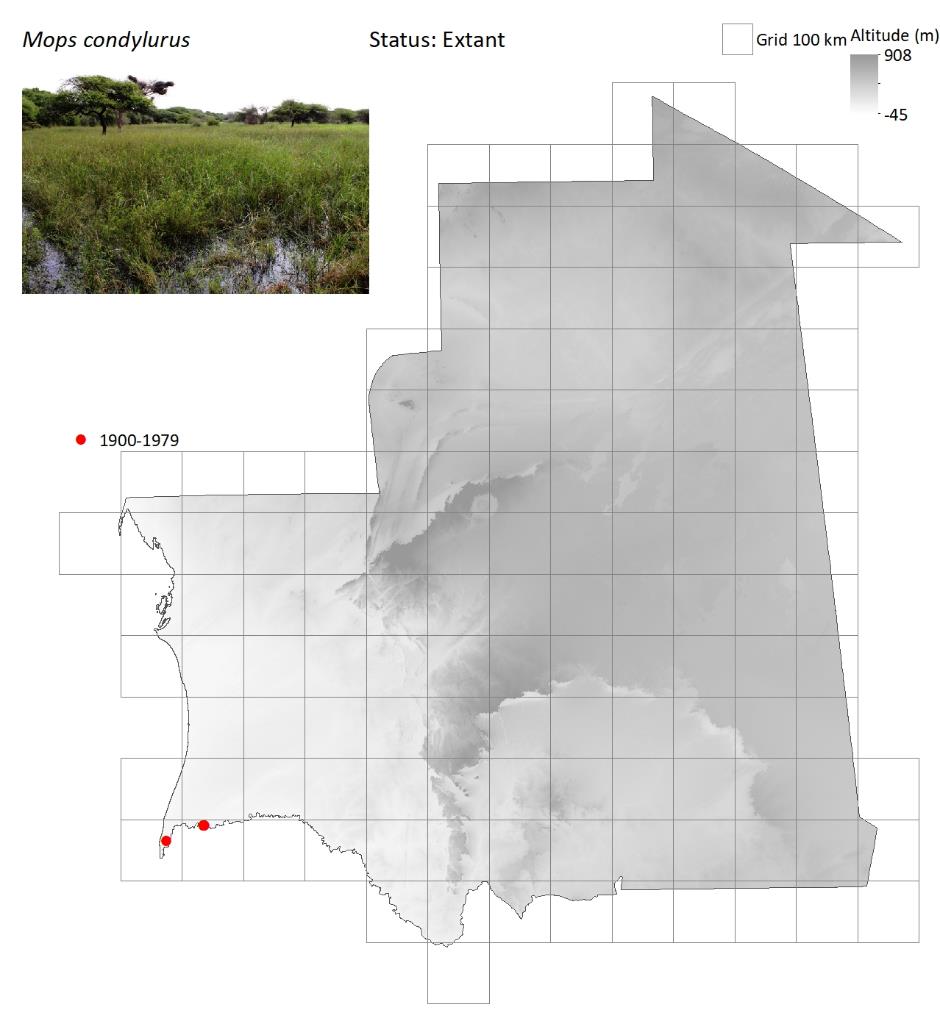


045 - Status and distribution of *Mops condylurus* in Mauritania. Picture of the habitat in the Senegal River delta, Province of Trarza (October 2010), where the species has been reported (Qumseyeh and Schütter 1981; ACR 2020).

Comparison with IUCN range polygons: New species for Mauritania. The observations in the Senegal River delta confirm the occurrence of the species in Mauritania and expand northwards the reported range, as the closest known populations are from the southern Senegal and Mali.

Conservation status: Global IUCN- LC; National final- DD; National original- DD.

Comment on the assessment: There are only two observations available from before the year 2000.


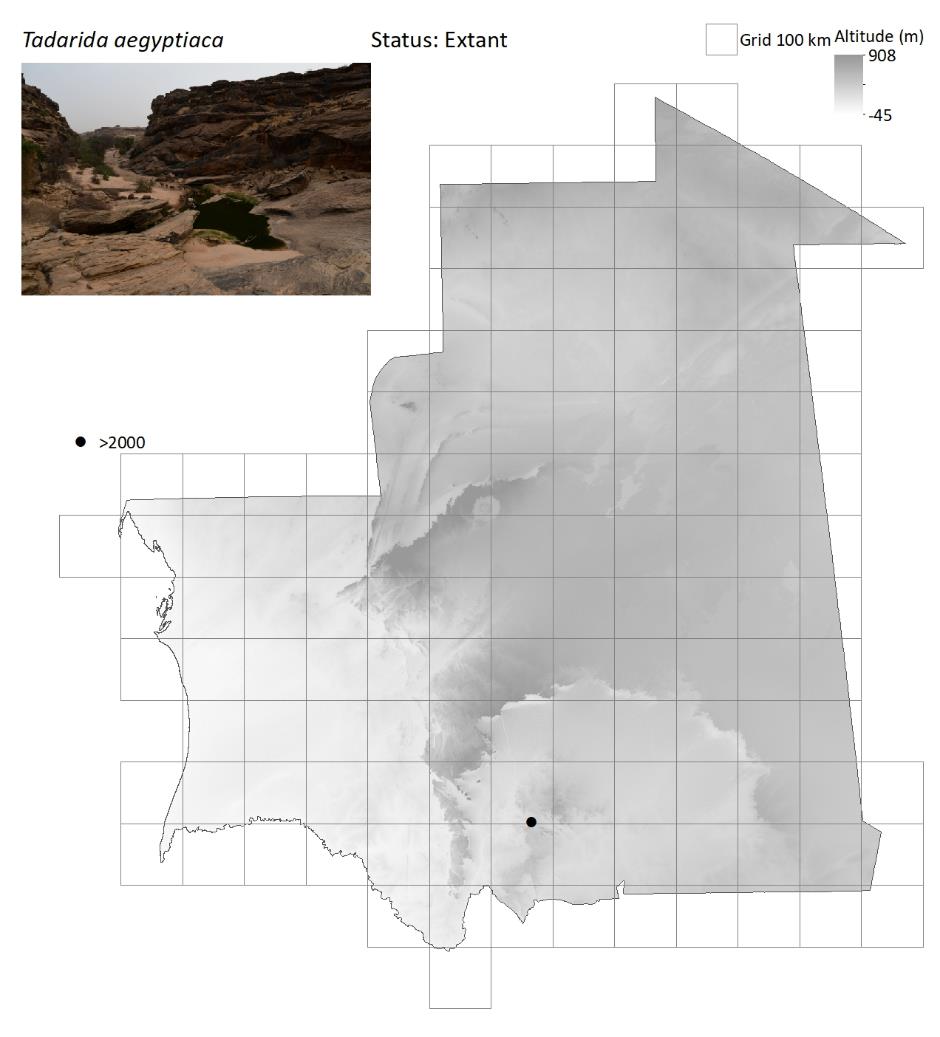


046 - Status and distribution of *Tadarida aegyptiaca* in Mauritania. Picture of the habitat in *Guelta* Metraoucha, Province of Hodh El Gharbi (June 2021), where the species has been reported (Nickel 2003).

Comparison with IUCN range polygons: New species for Mauritania. The observation in the Afollé plateau confirms the occurrence of the species in Mauritania and expand westwards the reported range, as the closest known populations are from the Hoggar Mountains in Algeria.

Conservation status: Global IUCN- LC; National final- DD; National original- DD.

Comment on the assessment: There is only one observation available.


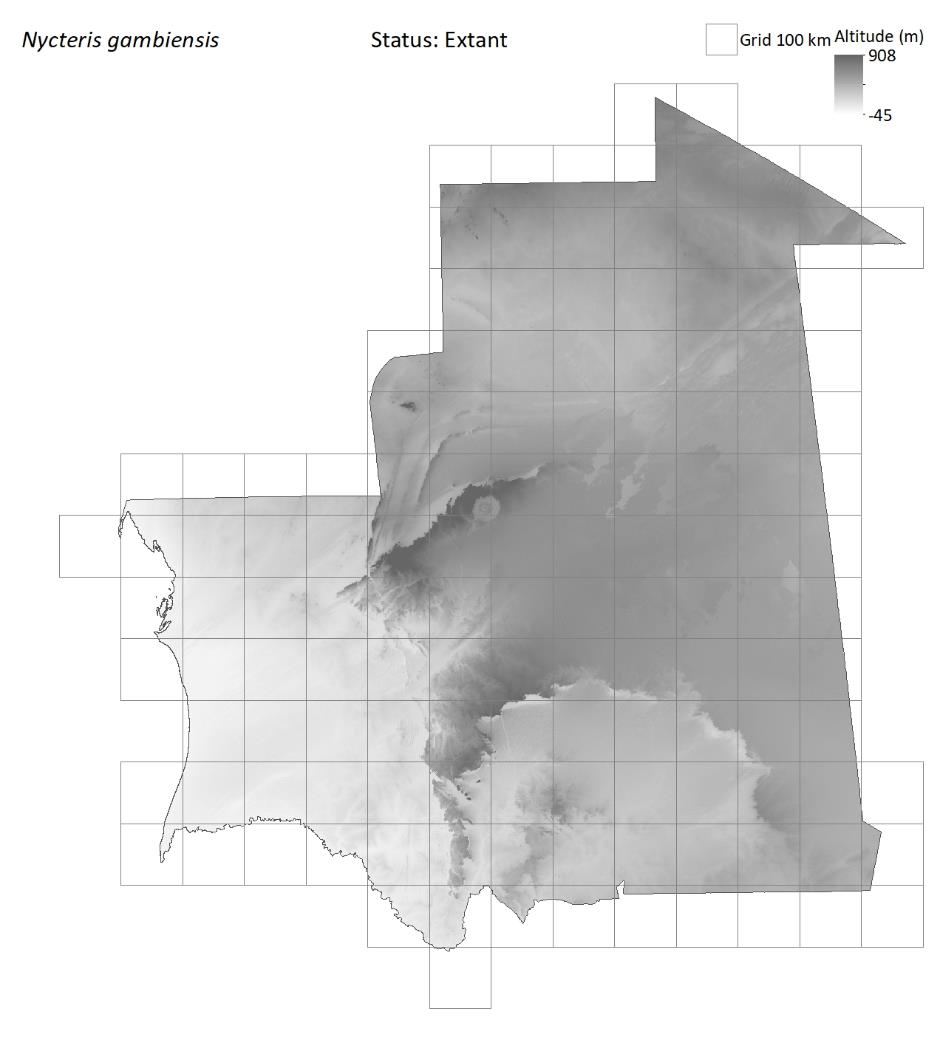


047 - Status and distribution of *Nycteris gambiensis* in Mauritania. The species has been reported from Mauritania without a precise locality (ACR 2020). For this reason, the distribution is not mapped.

Comparison with IUCN range polygons: Uncertainty. The species is reported to occur in the Province of Guidimaka (IUCN 2021) and the African Chiroptera Report (ACR 2020) reports presence in Mauritania without a single location. Additional sampling is needed to understand the current status and range of the species.

Conservation status: Global IUCN- LC; National final- DD; National original- DD.

Comment on the assessment: No single observation that can be mapped is available.


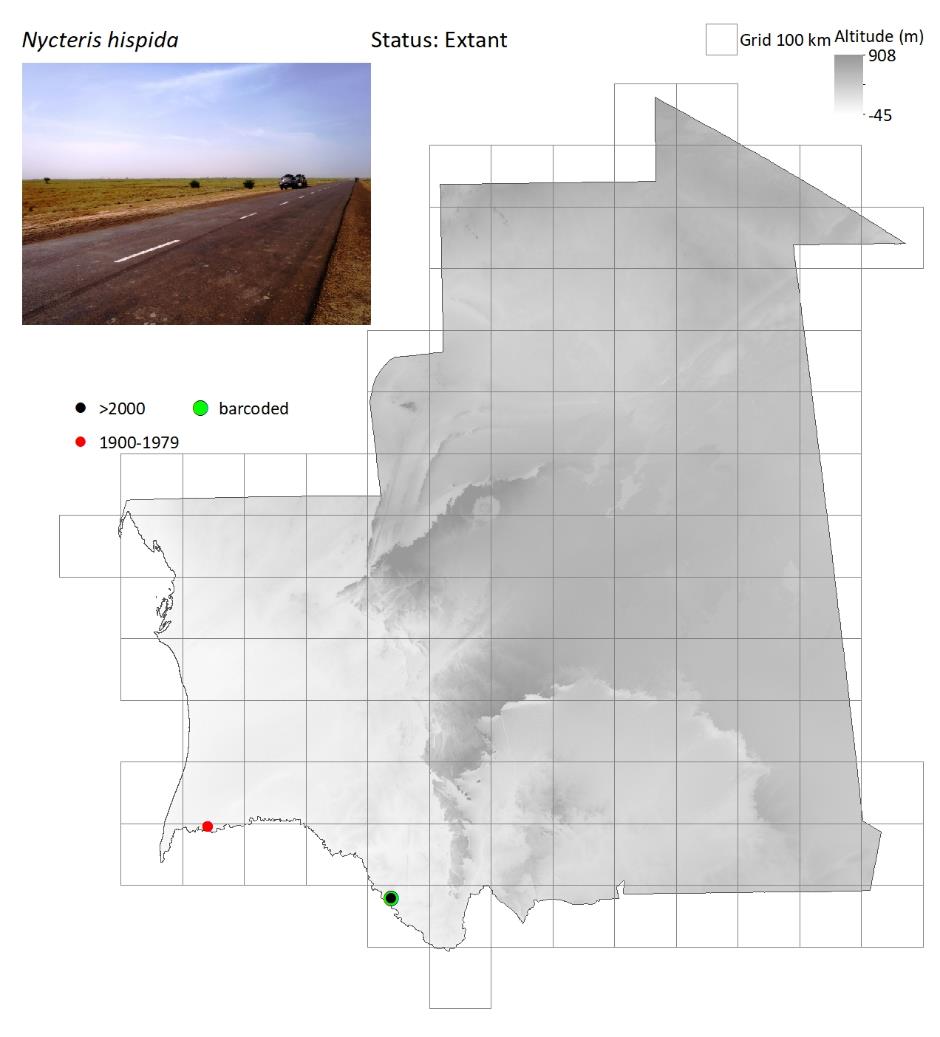


048 - Status and distribution of *Nycteris hispida* in Mauritania. Picture of the habitat near Garak, Province of Trarza (November 2012), where the species has been reported (Qumseyeh and Schütter 1981; ACR 2020).

Comparison with IUCN range polygons: Range expansion. The observation in the upper Senegal River valley expands eastwards the reported range in Mauritania.

Conservation status: Global IUCN- LC; National final- LC; National original- NT B1a.

Comment on the assessment: Downgraded due to the occurrence of neighbouring populations in Senegal that may likely provide rescue effect. Reduced extent of occurrence but lack of evidence on continuous decline in range extent or habitat quality.


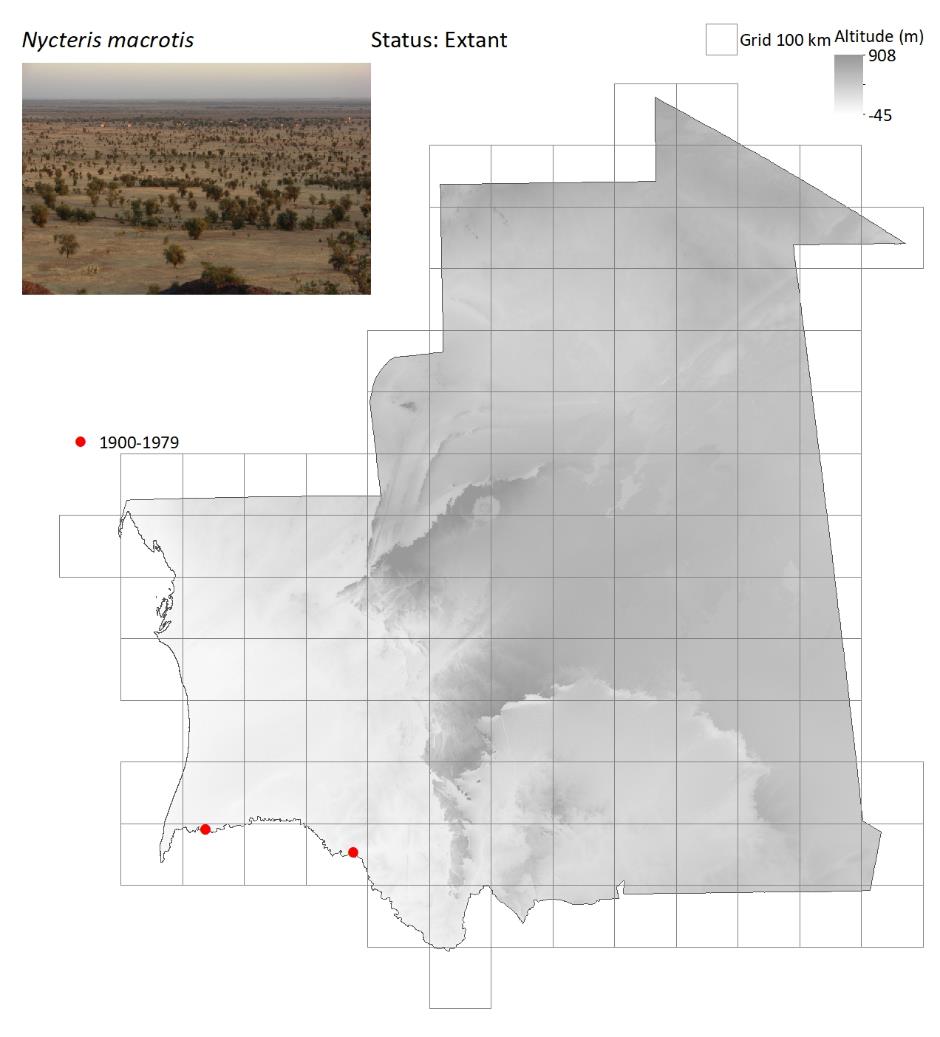


049 - Status and distribution of *Nycteris macrotis* in Mauritania. Picture of the habitat near Kaédi, Province of Gorgol (December 2007), where the species has been reported (ACR 2020).

Comparison with IUCN range polygons: No change. Mapped distribution is similar to reported range in Mauritania.

Conservation status: Global IUCN- LC; National final- LC; National original- NT B1a.

Comment on the assessment: Downgraded due to the occurrence of neighbouring populations in Senegal that may likely provide rescue effect. Reduced extent of occurrence but lack of evidence on continuous decline in range extent or habitat quality.


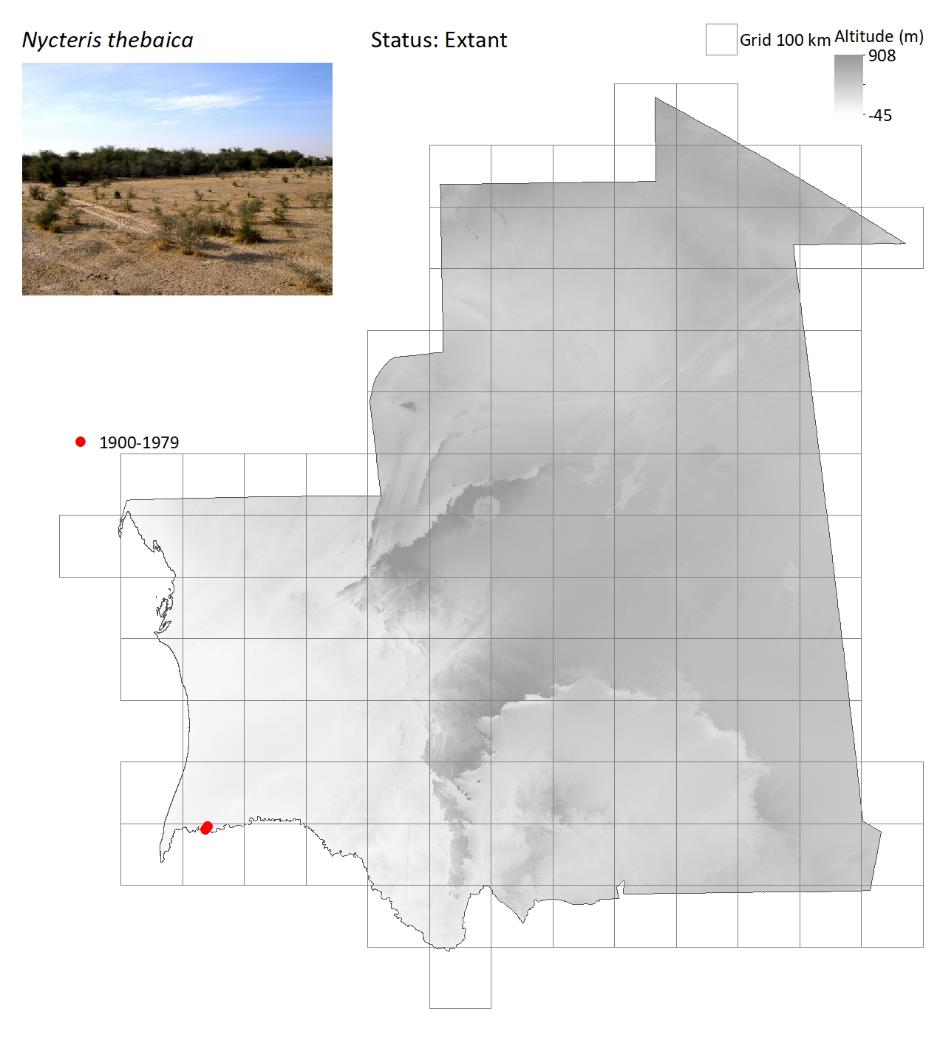


050 - Status and distribution of *Nycteris thebaica* in Mauritania. Picture of the habitat near Garak, Province of Trarza (December 2007), where the species has been reported (GBIF.org 2021).

Comparison with IUCN range polygons: Range expansion. The observation in the Senegal River valley expands westwards the reported range in Mauritania.

Conservation status: Global IUCN- LC; National final- DD; National original- DD.

Comment on the assessment: There are only two observations available from before the year 2000.


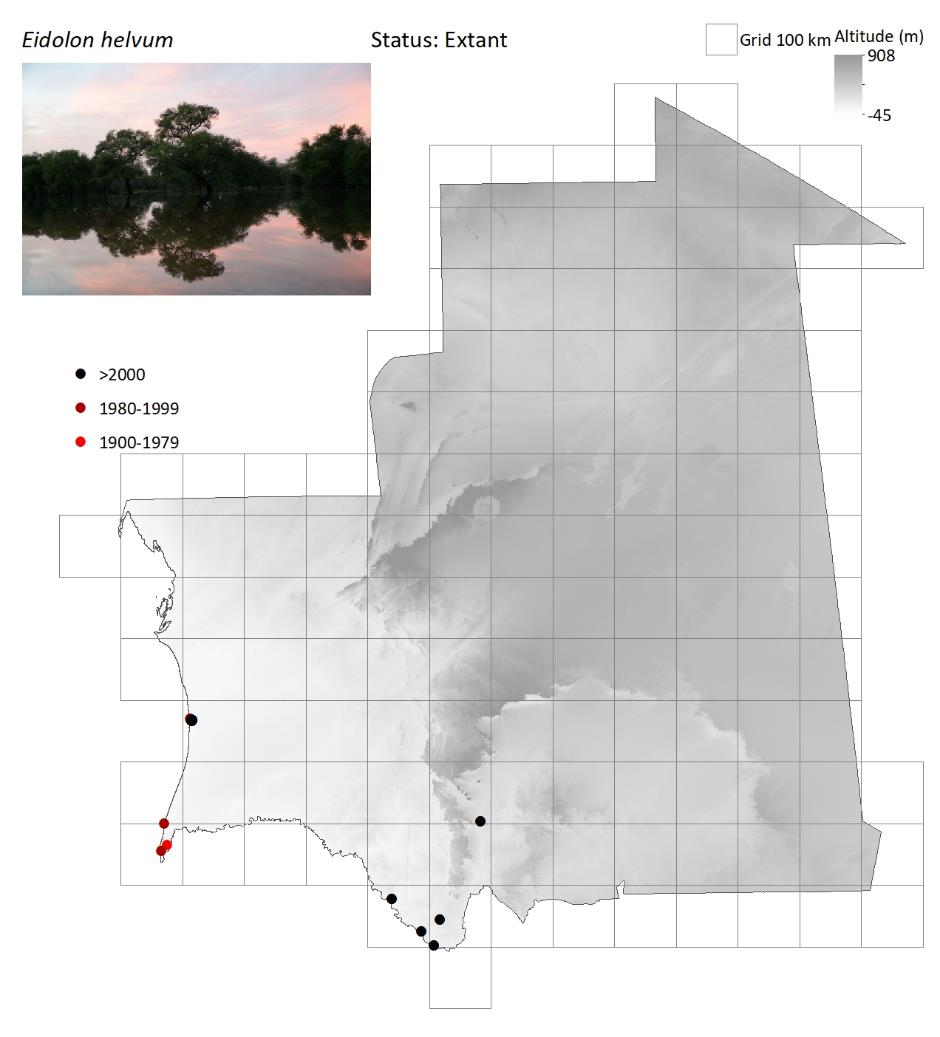


051 - Status and distribution of *Eidolon helvum* in Mauritania. Picture of the habitat in *tamoûrt* Kreiket, Province of Assaba (November 2020), where the species was observed by the authors.

Comparison with IUCN range polygons: New species for Mauritania. The observations in the Senegal River valley, Nouakchott, and eastern Assaba plateau confirm the occurrence of the species in Mauritania and expand the reported range, as the closest known populations are from coastal Senegal.

Conservation status: Global IUCN- NT; National final- LC; National original- LC.

Comment on the assessment: The species may actually be expanding in Mauritania, benefiting from increasing agricultural activities in southern regions. This species was previously much more localised and in recent years it has been increasingly easier to observe, especially in the Province of Guidimaka.


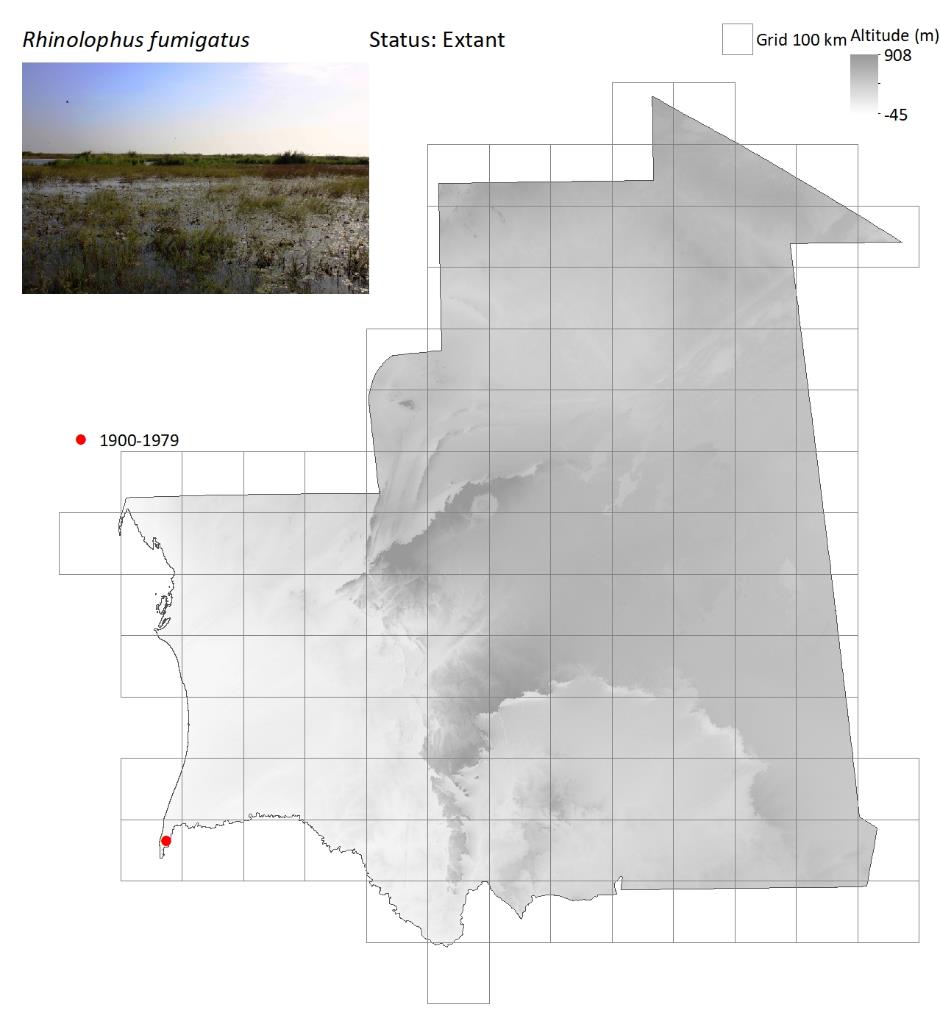


052 - Status and distribution of *Rhinolophus fumigatus* in Mauritania. Picture of the habitat in the Senegal River delta, Province of Trarza (November 2004), where the species has been reported (National Research Council 1981).

Comparison with IUCN range polygons: Uncertainty. The observation in the Senegal River delta apparently confirms the occurrence of the species in Mauritania and expands the reported range, as the closest known populations are from north-eastern Senegal and south-western Mali (IUCN 2021). However, the observation was reported without a voucher or a photograph (National Research Council 1981), and it may represent a misidentification. A voucher reported from “Mauritania Sahara” is deposited in the Natural History Museum of Paris (MO-1911-1555; ACR 2020). Additional sampling is needed to understand the current status and range of the species in Mauritania.

Conservation status: Global IUCN- LC; National final- DD; National original- DD.

Comment on the assessment: There is only one observation available from before the year 2000.


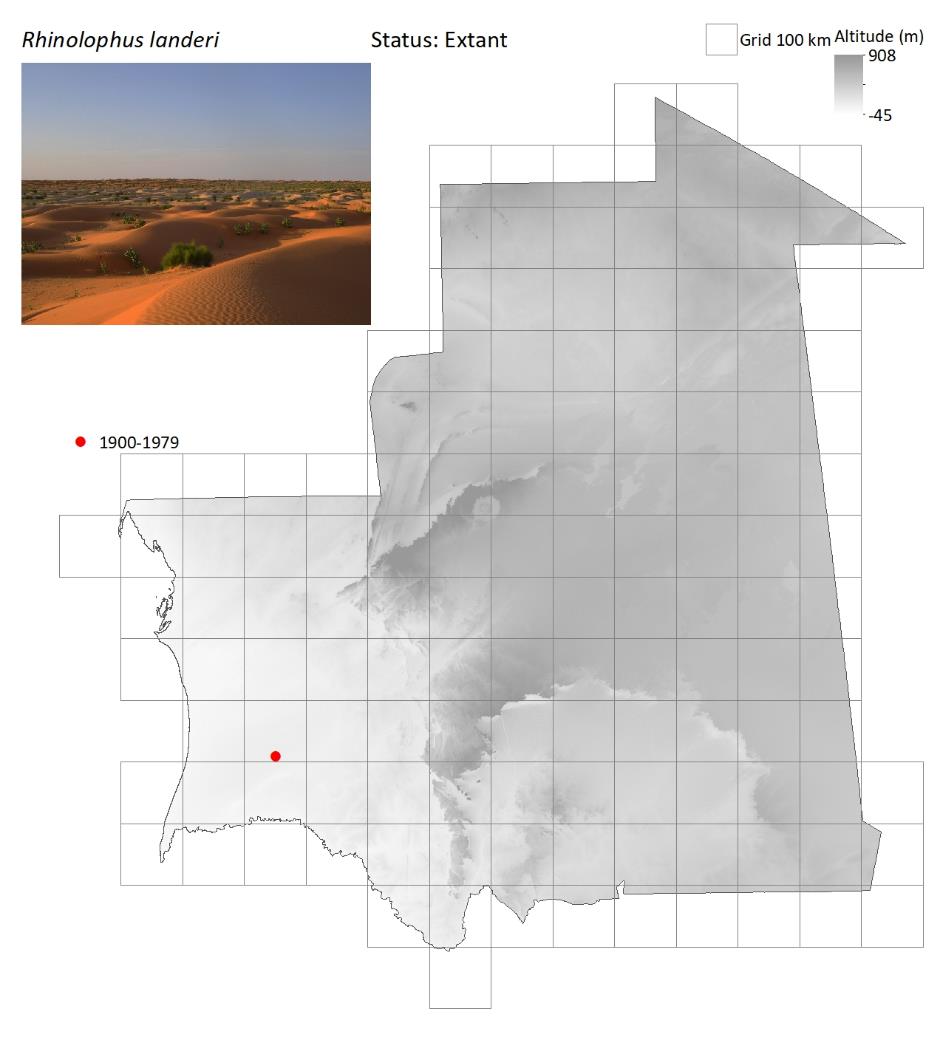


053 - Status and distribution of *Rhinolophus landeri* in Mauritania. Picture of the habitat near Boutilimit, Province of Trarza (November 2003), where the species has been reported (ACR 2020).

Comparison with IUCN range polygons: Range expansion. The single observation in Trarza Province expands northwards the reported range of this species in Mauritania, as it was only known from Guidimaka. Still, the observation is from before the year 2000, and additional sampling is needed to understand the current range of the species in Mauritania.

Conservation status: Global IUCN- LC; National final- DD; National original- DD.

Comment on the assessment: There is only one observation available from before the year 2000.


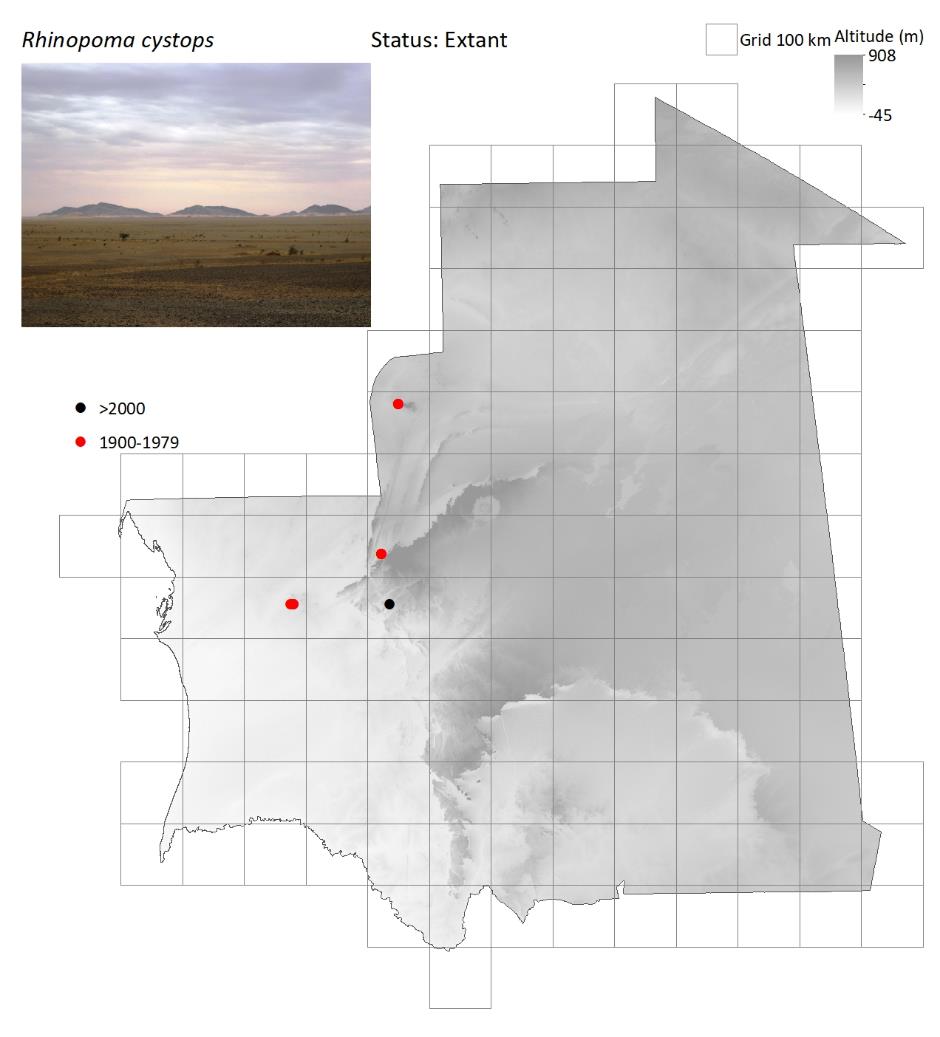


054 - Status and distribution of *Rhinopoma cystops* in Mauritania. Picture of the habitat near Akjoujt, Province of Inchiri (November 2004), where the species has been reported (Poulet 1970; Qumseyeh and Schütter 1981; ACR 2020).

Comparison with IUCN range polygons: Range expansion. The observation in the Adrar Atar plateau expands eastwards the reported range of the species in Mauritania.

Conservation status: Global IUCN- LC; National final- LC; National original- LC.


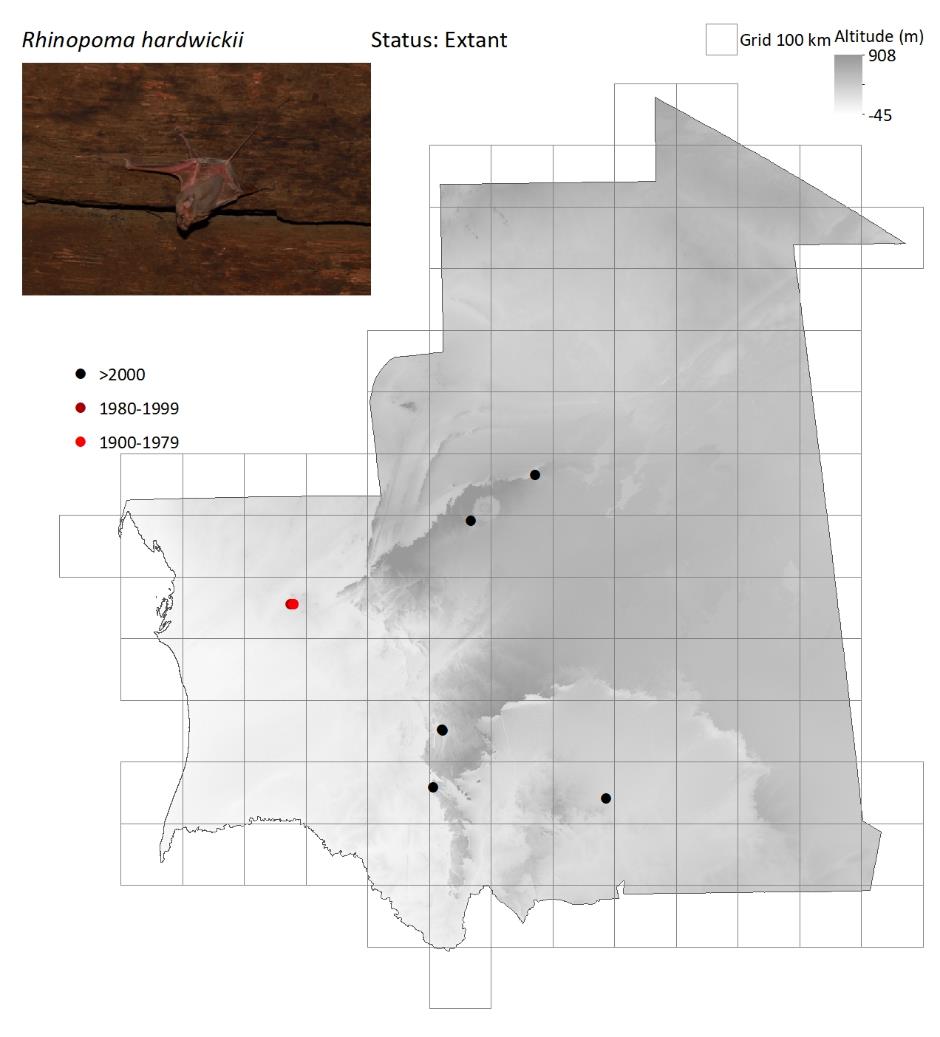


055 - Status and distribution of *Rhinopoma hardwickii* in Mauritania. Picture in Ouadâne, Province of Adrar (July 2012).

Comparison with IUCN range polygons: Range expansion. The observations in the Tagant, Assaba and Afollé plateaus expand south-westward the reported range of the species in Mauritania.

Conservation status: Global IUCN- LC; National final- LC; National original- LC.


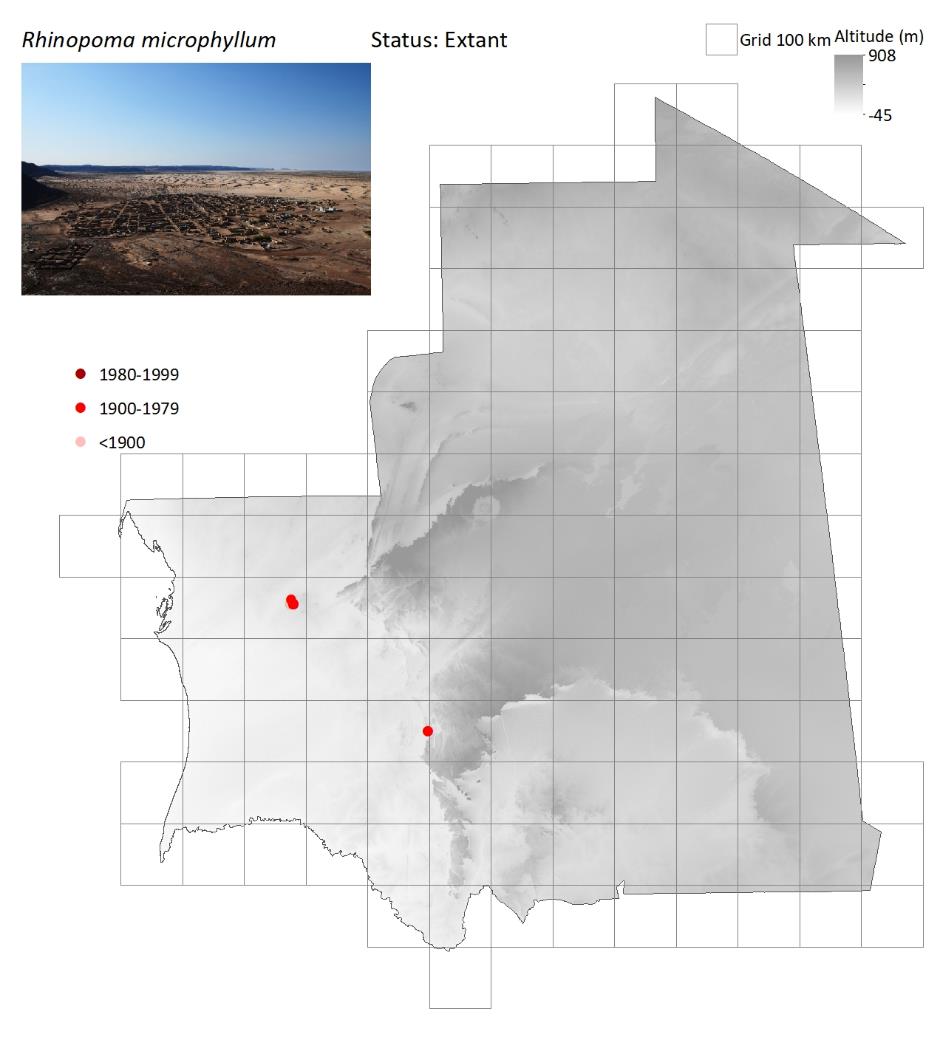


056 - Status and distribution of *Rhinopoma microphyllum* in Mauritania. Picture of Moudjéria, Province of Tagant (November 2019), where the species has been reported (ACR 2020).

Comparison with IUCN range polygons: No change. Mapped distribution is similar to reported range in Mauritania.

Conservation status: Global IUCN- LC; National final- LC; National original- LC.


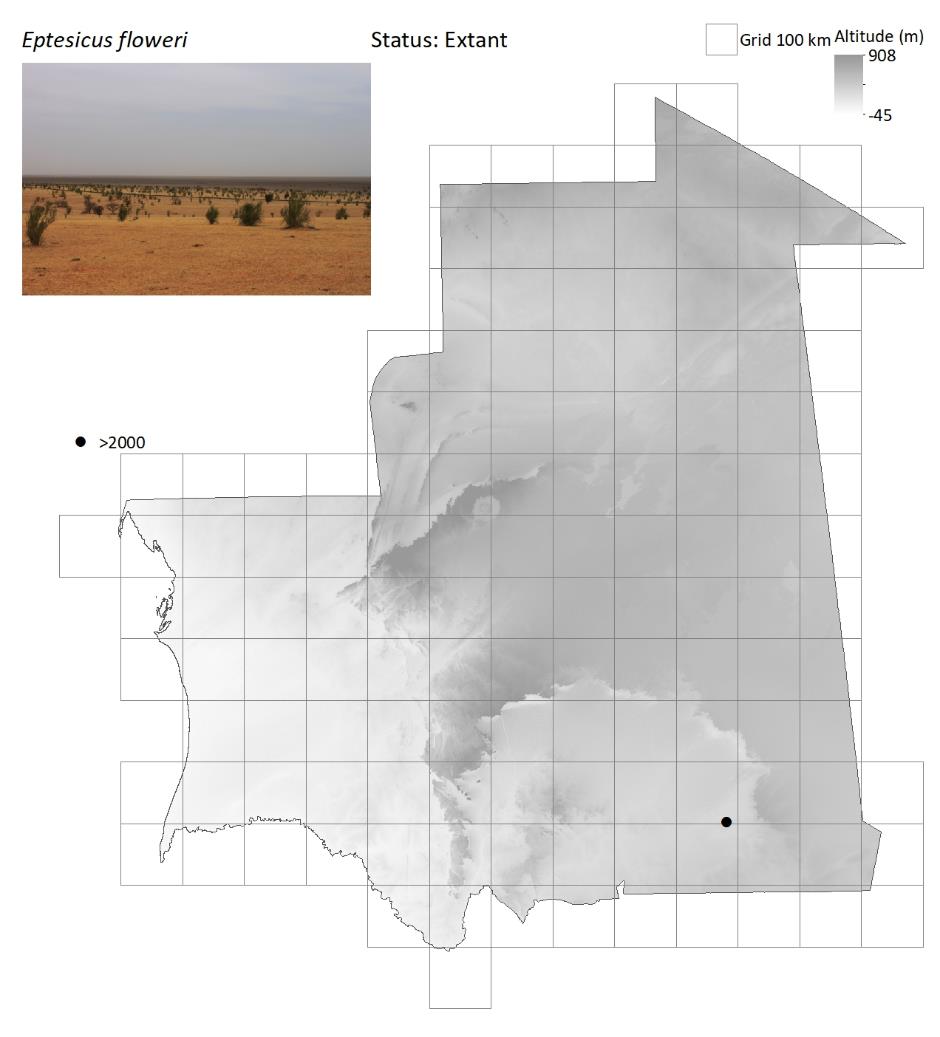


057 - Status and distribution of *Eptesicus floweri* in Mauritania. Picture of the habitat in Lake Mahmoûdé, Province of Hodh Ech Chargui (January 2014), where the species has been reported (Padial and Ibáñez 2005; ACR 2020).

Comparison with IUCN range polygons: No change. Mapped distribution is similar to reported range in Mauritania.

Conservation status: Global IUCN- LC; National final- DD; National original- DD.

Comment on the assessment: There is only one observation available.


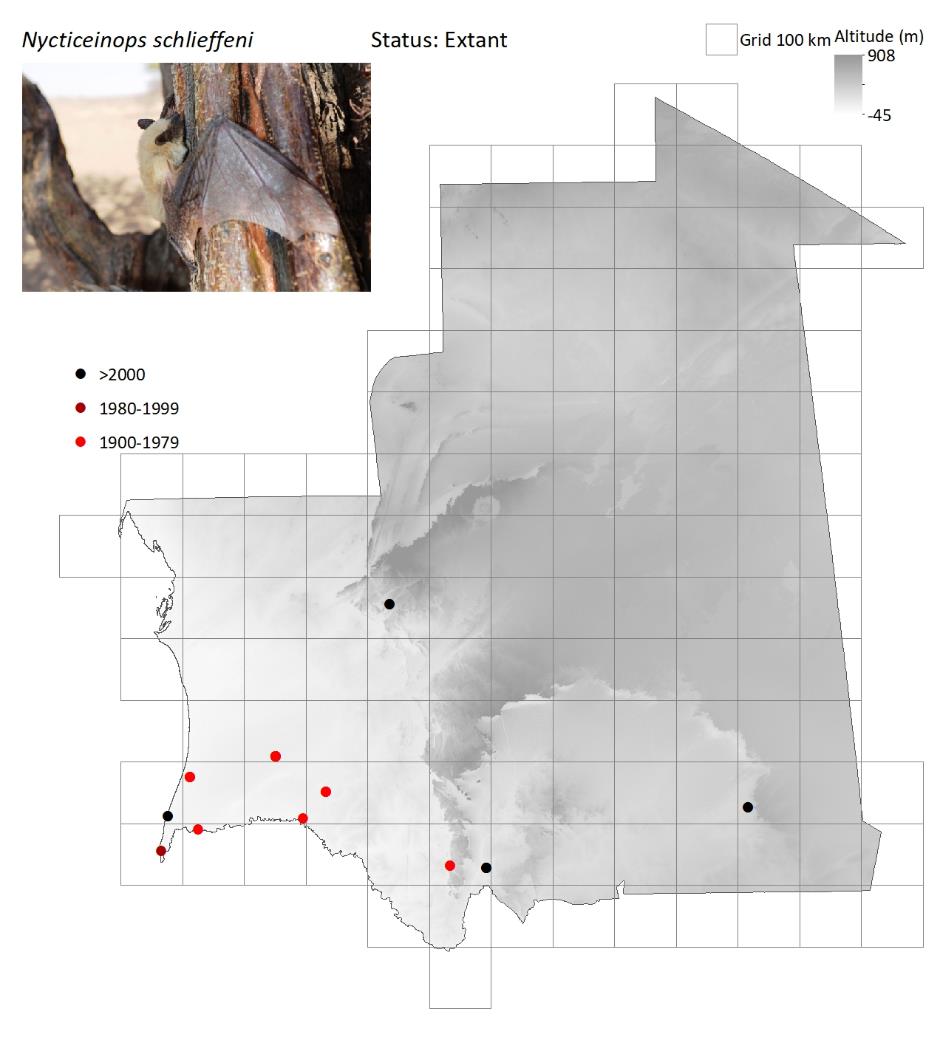


058 - Status and distribution of *Nycticeinops schlieffeni* in Mauritania. Picture near Néma, Province of Hodh Ech Chargui (January 2014).

Comparison with IUCN range polygons: Range expansion. Most observations, but especially the ones from the Adrar Atar plateau and Dhar Néma, expand northwards and eastwards the reported range of the species in Mauritania.

Conservation status: Global IUCN- LC; National final- LC; National original- LC.


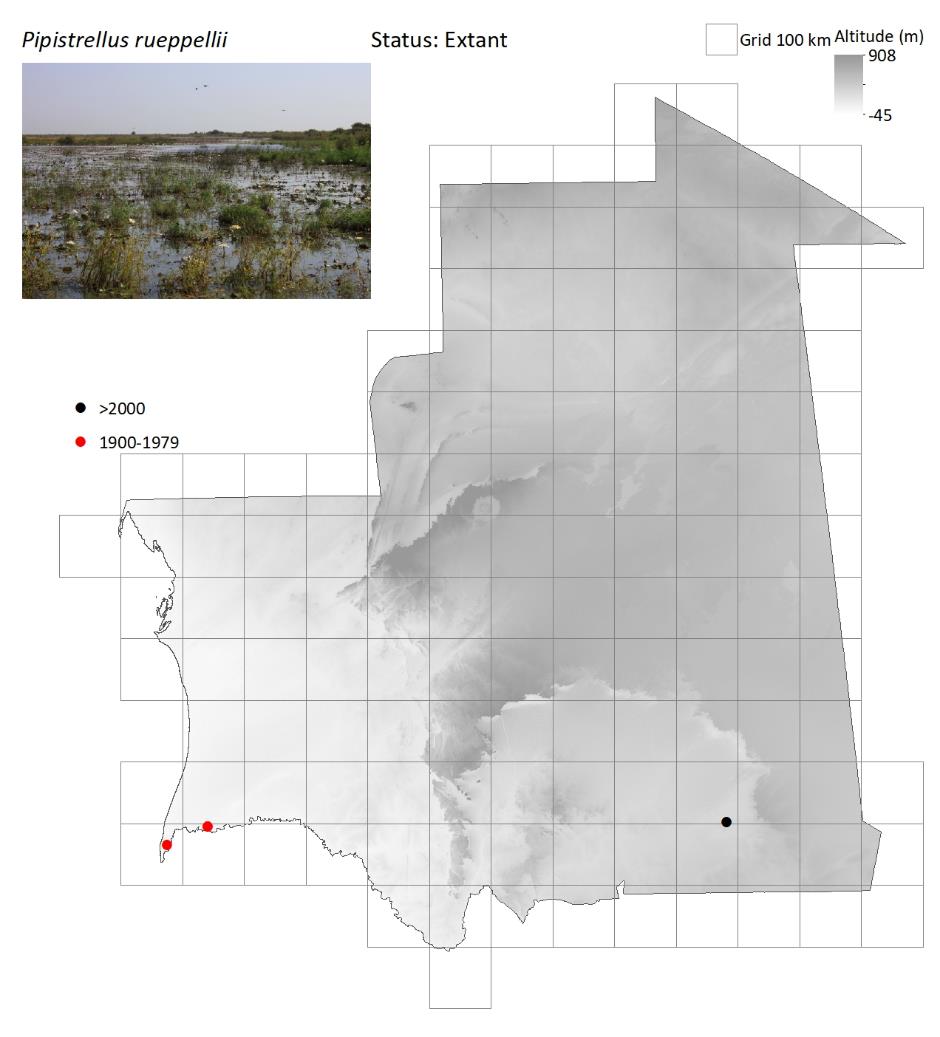


059 - Status and distribution of *Pipistrellus rueppellii* in Mauritania. Picture of the habitat in Senegal River delta, Province of Trarza (November 2014), where the species has been reported (National Research Council 1981).

Comparison with IUCN range polygons: Range expansion. All observations, but especially the one from Lake Mahmoûdé, expand eastward the reported range of the species in Mauritania.

Conservation status: Global IUCN- LC; National final- LC; National original- LC.


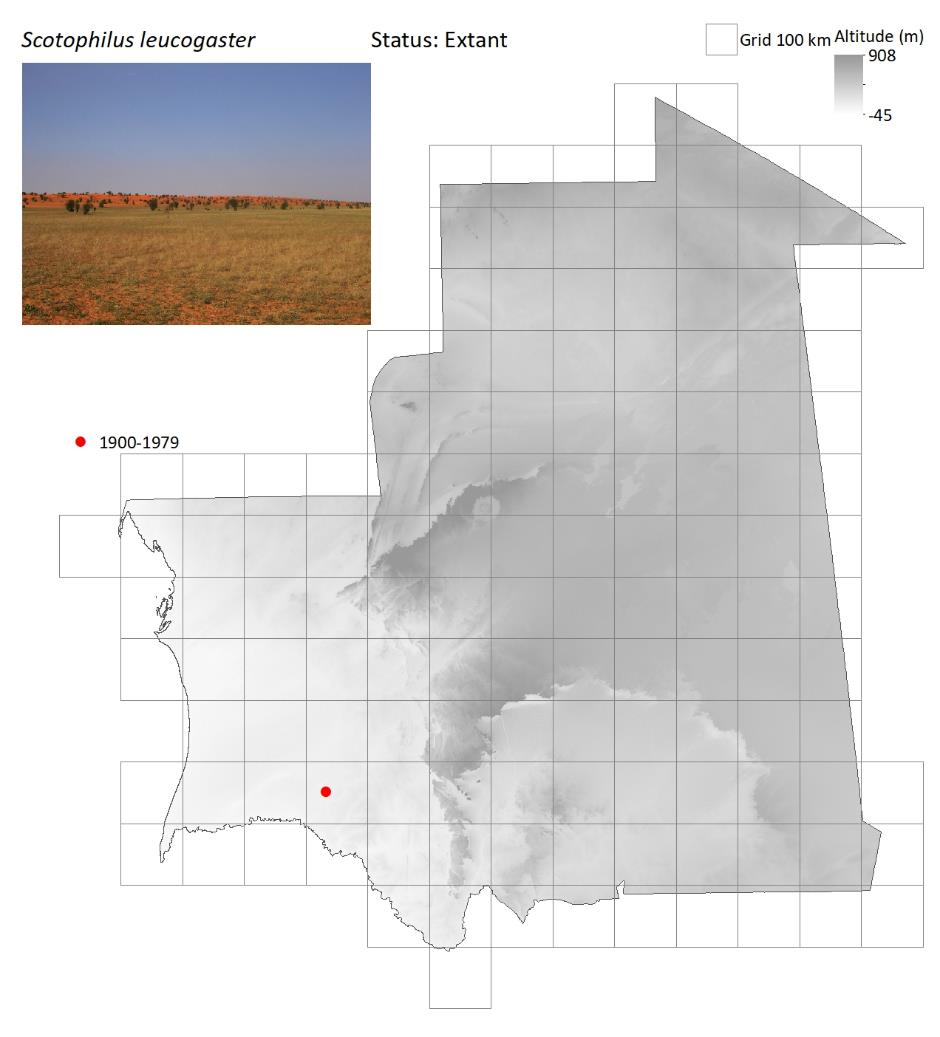


060 - Status and distribution of *Scotophilus leucogaster* in Mauritania. Picture of the habitat near Aleg, Province of Brakna (November 2003), where the species has been reported (Qumseyeh and Schütter 1981; ACR 2020).

Comparison with IUCN range polygons: No change. Mapped distribution is similar to reported range in Mauritania.

Conservation status: Global IUCN- LC; National final- DD; National original- DD.

Comment on the assessment: There is only one observation available from before the year 2000.


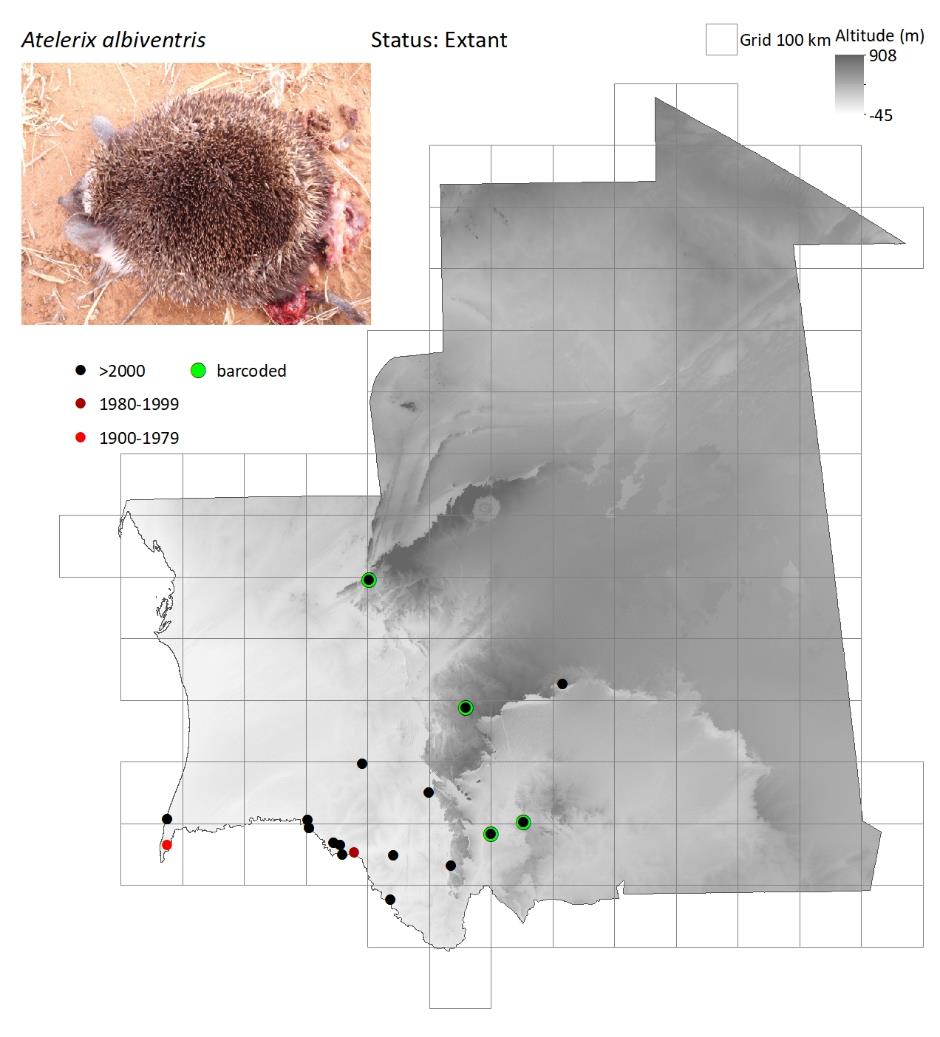


061 - Status and distribution of *Atelerix albiventris* in Mauritania. Picture of a roadkill specimen found in tâmoûrt El 'Asba, Province of Assaba (November 2008).

Comparison with IUCN range polygons: Range expansion. The observations in the Adrar Atar, Tagant, Assaba and Afollé plateaus expand northwards the reported range of the species in Mauritania.

Conservation status: Global IUCN- LC; National final- LC; National original- LC.


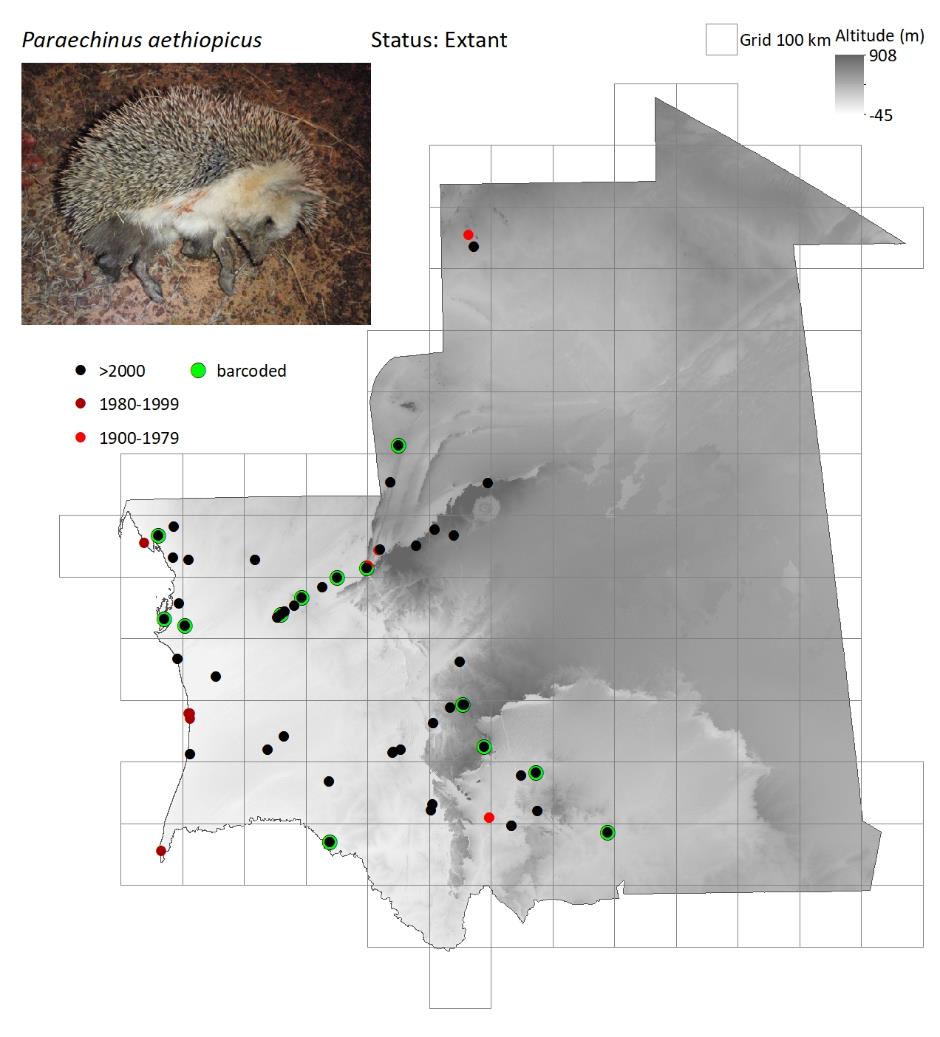


062 - Status and distribution of *Paraechinus aethiopicus* in Mauritania. Picture of a roadkill specimen found in Nega Pass, Province of Tagant (November 2011).

Comparison with IUCN range polygons: New species for Mauritania. The observations in western Mauritania and in the four mountain plateaus confirm the occurrence of the species in Mauritania and expand the reported range, as the closest known populations are from Central Morocco.

Conservation status: Global IUCN- LC; National final- LC; National original- LC.


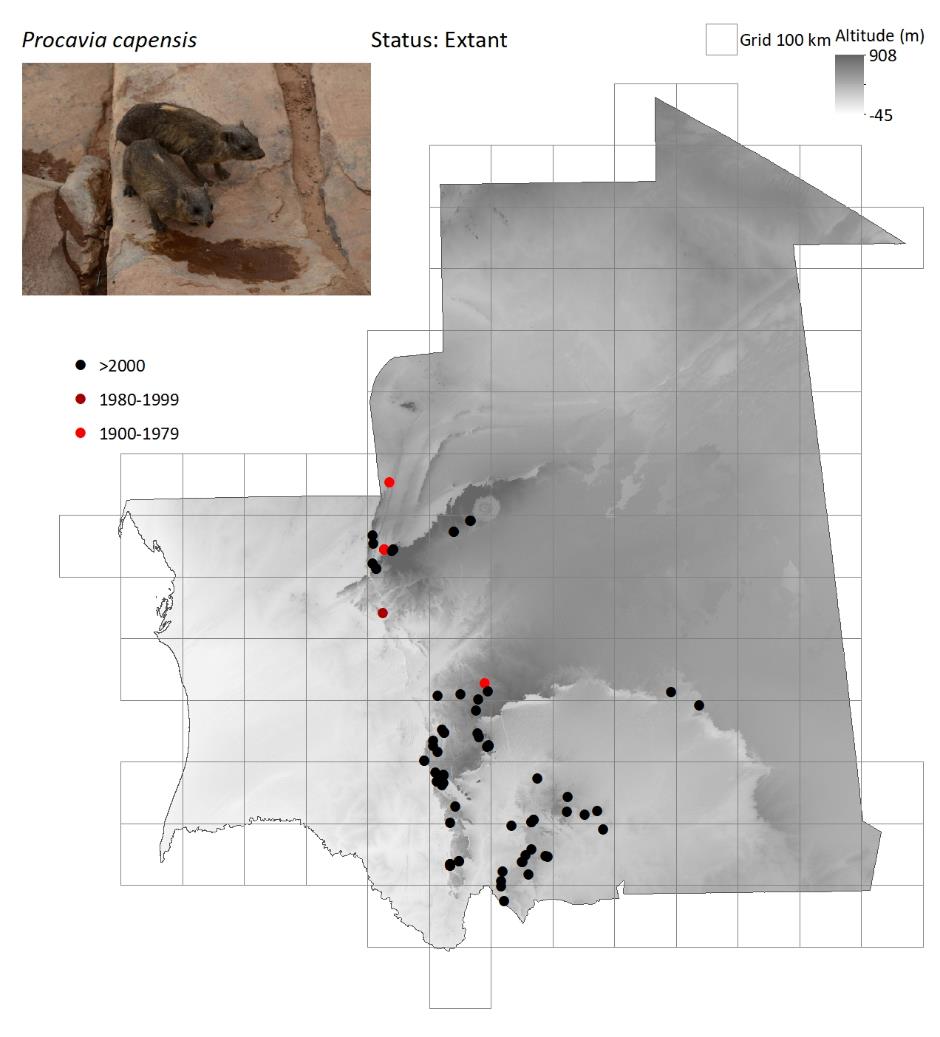


063 - Status and distribution of *Procavia capensis* in Mauritania. Picture in Ouadâne, Province of Adrar (November 2019).

Comparison with IUCN range polygons: Better definition of range. The species is apparently restricted to the rock outcrops of the mountain plateaus and escarpments and is absent from coastal flat areas. This indicates that the range of the species in Mauritania is smaller than what is presently reported.

Conservation status: Global IUCN- LC; National final- LC; National original- LC.


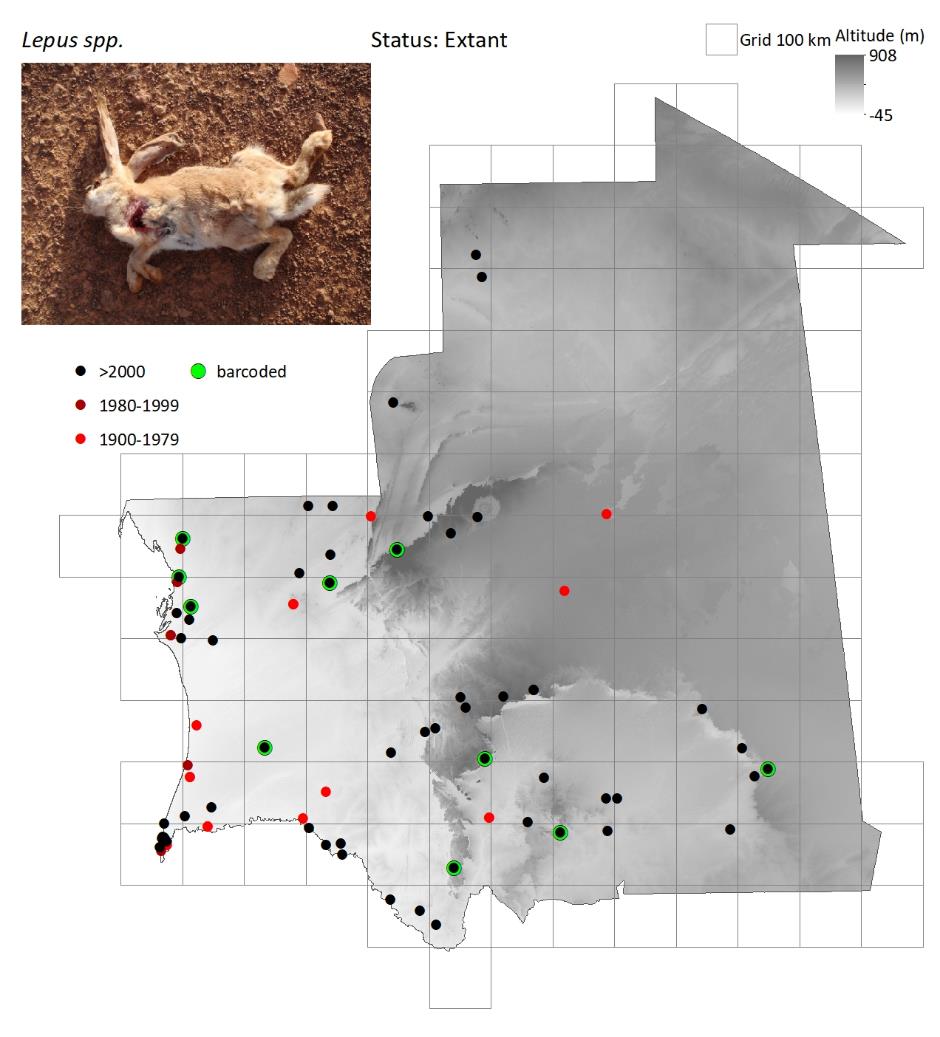


064 - Status and distribution of *Lepus spp.* in Mauritania. Picture of a roadkill specimen found in the crossroad to the Amogjâr pass, Province of Adrar (October 2011).

Comparison with IUCN range polygons: Uncertainty. The current taxonomic status of the species precludes range comparisons. Both *Lepus capensis* and *L. microtis* are reported from Mauritania (IUCN 2021). Given that the taxonomic status of both species is pending a thorough revision (Lado et al 2019), it was opted to represent all available observations as *Lepus spp*. Molecular barcoding and an assessment of the systematics and taxonomy of the genus are needed to clear the taxonomic status of the species.

Conservation status: Global IUCN- NE; National final- NA; National original- NA.

Comment on the assessment: The current taxonomic uncertainty precludes the assessment.


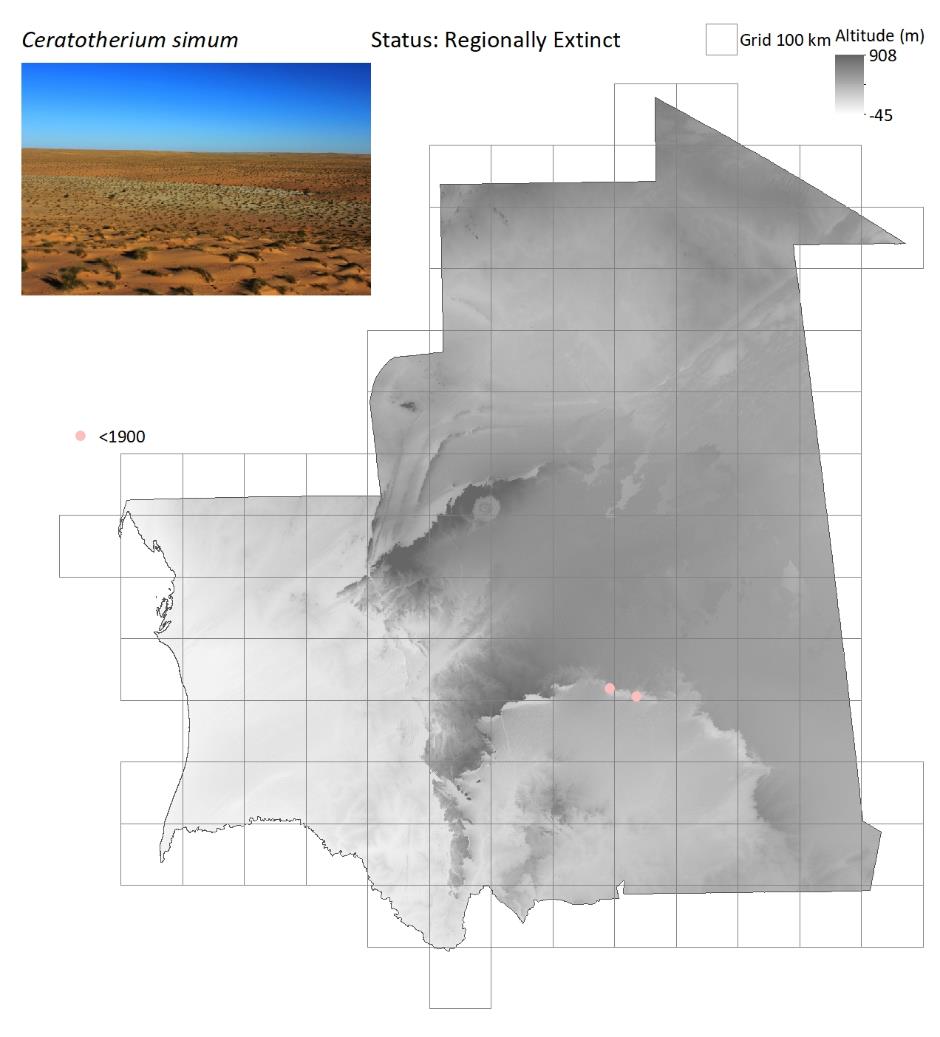


065 - Status and distribution of *Ceratotherium simum* in Mauritania. Picture of the habitat in Zig on the foothills of the Dhar Tichitt, Province of Tagant (February 2014), where subfossils of the species were recovered (Holl 1985, 2009).

Comparison with IUCN range polygons: Not possible. The reported range of the species does not include areas with extinct populations.

Conservation status: Global IUCN- NT; National final- RE; National original- RE.


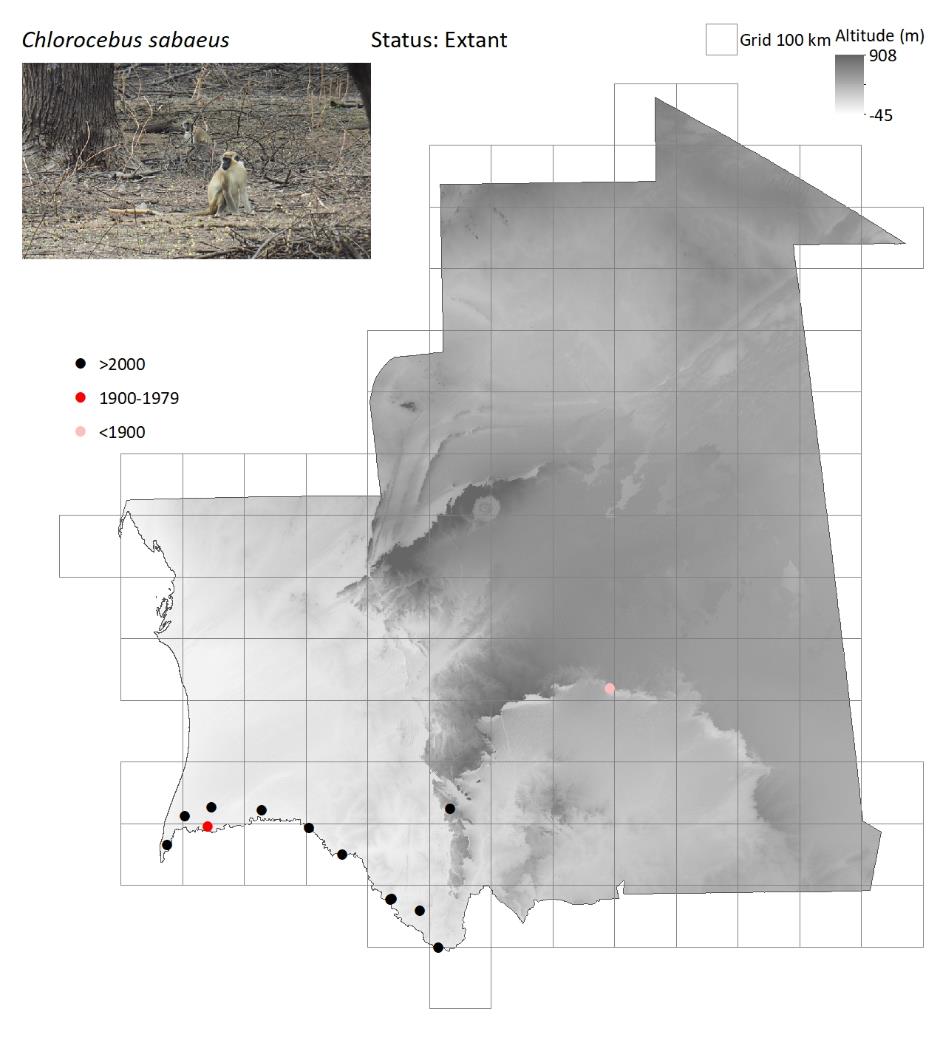


066 - Status and distribution of *Chlorocebus sabaeus* in Mauritania. Picture in Ngouye, Province of Gorgol (November 2020).

Comparison with IUCN range polygons: Range expansion. The observations in the lower Senegal River valley expand westwards the reported range of this species in Mauritania. The observation in the Assaba plateau expands northwards the reported range, although it was obtained from an interview to local people. Additional sampling is needed to understand the current range of the species in Mauritania.

Conservation status: Global IUCN- LC; National final- NT C2a(i); National original- VU.

Comment on the assessment: Downgraded due to the occurrence of neighbouring populations in Mali and Senegal that may likely provide rescue effect. Inferred continuous population decline due to persecution.


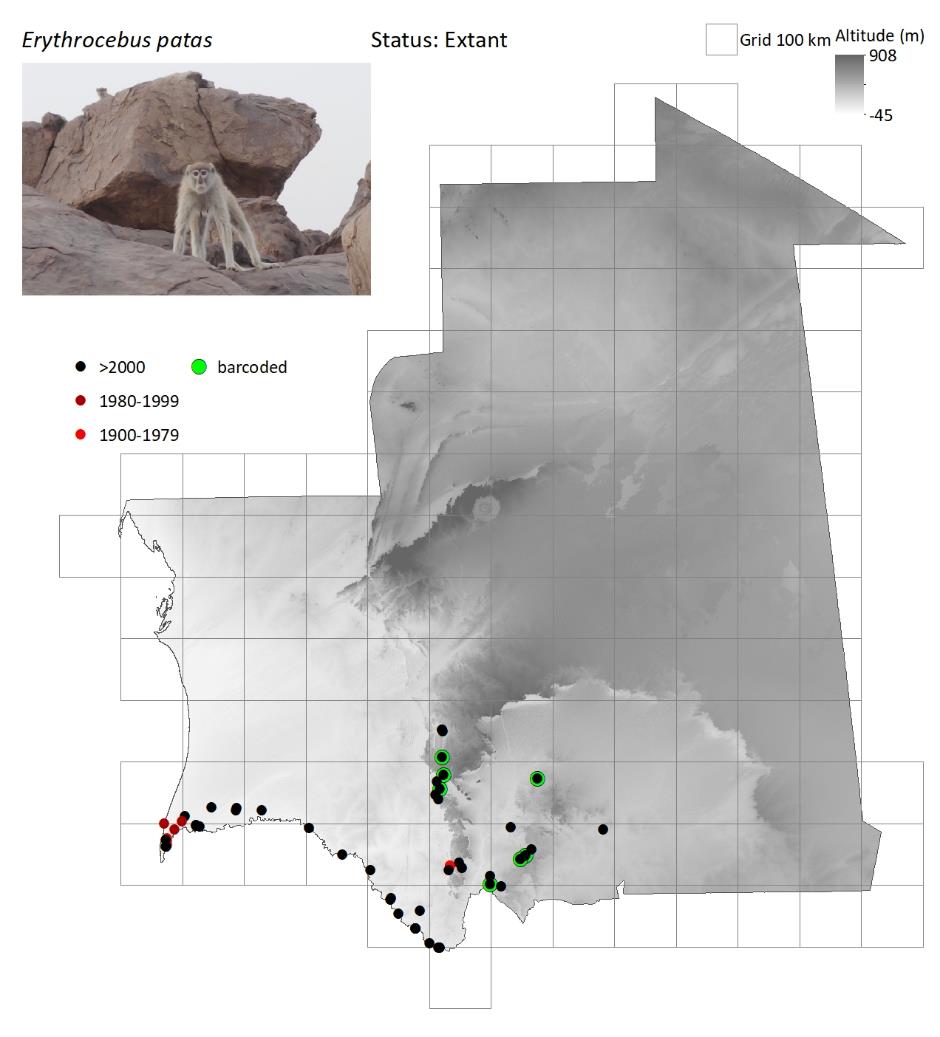


067 - Status and distribution of *Erythrocebus patas* in Mauritania. Picture in *Guelta* Matmâta, Province of Tagant (June 2021).

Comparison with IUCN range polygons: No change. Mapped distribution is similar to reported range in Mauritania.

Conservation status: Global IUCN- NT; National final- LC; National original- LC.

Comment on the assessment: Contrarily from other sub-Saharan populations of non-human primates, the species is apparently not affected by killing for bushmeat.


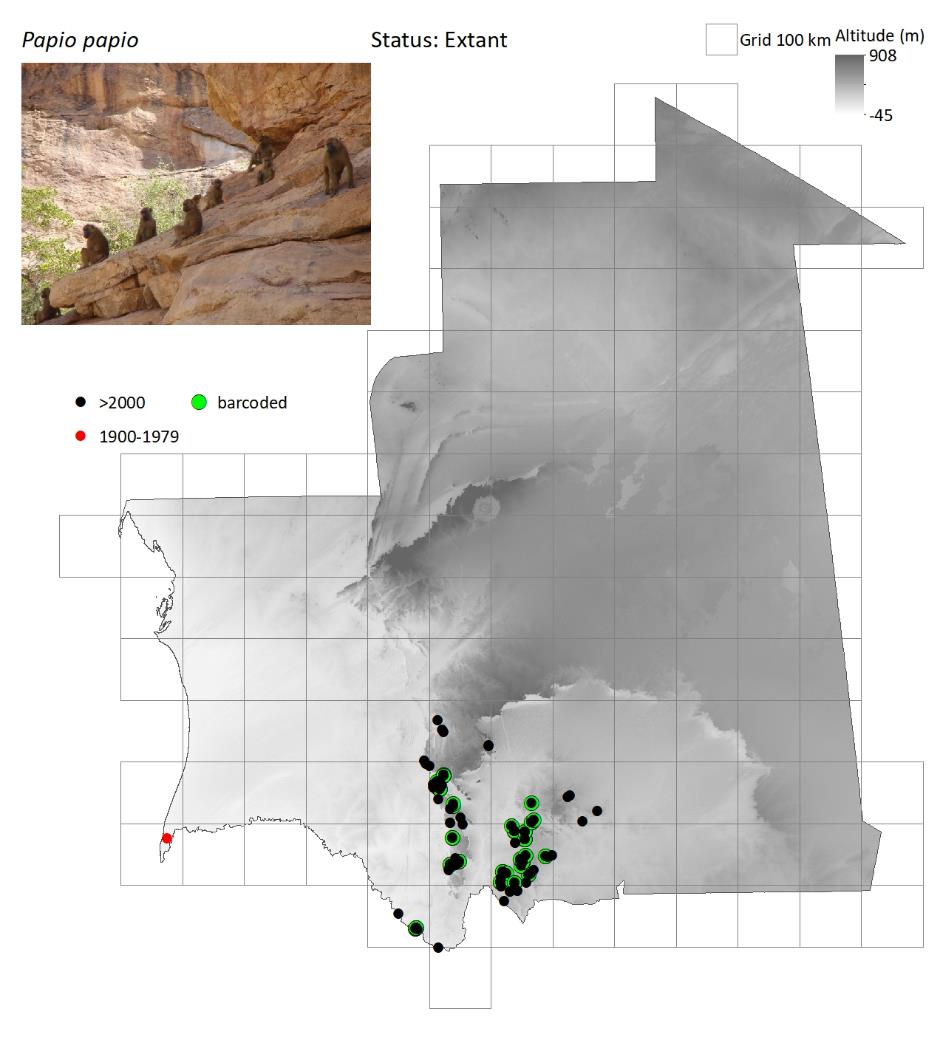


068 - Status and distribution of *Papio papio* in Mauritania. Picture of a group observed at Guenétir, Province of Assaba (December 2007). The species was known to occur in the Senegal River delta at least until the 1920s (Spatz 1930). No observations were made after that period.

Comparison with IUCN range polygons: No change. Mapped distribution is similar to reported range in Mauritania.

Conservation status: Global IUCN- NT; National final- VU C2a(i); National original- VU.

Comment on the assessment: Downgrading excluded due lack of evidence proving rescue effect from neighbouring populations in Mali and Senegal. Inferred continuous population decline due to persecution.


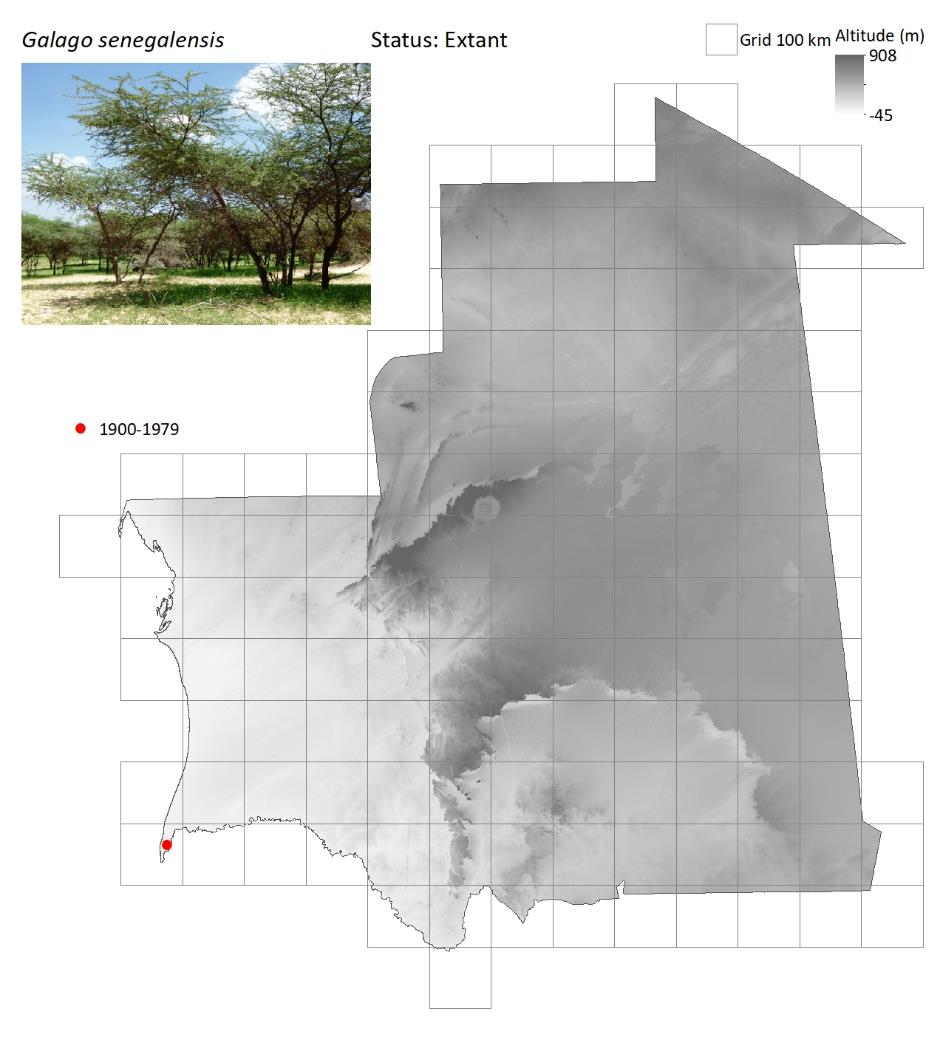


069 - Status and distribution of *Galago senegalensis* in Mauritania. Picture of the habitat in the Diawling National Park, Province of Trarza (August 2015), where the species has been reported to occur (National Research Council 1981).

Comparison with IUCN range polygons: No change. Mapped distribution is similar to reported range in Mauritania.

Conservation status: Global IUCN- LC; National final- DD; National original- DD.

Comment on the assessment: There is only one observation available from before the year 2000.


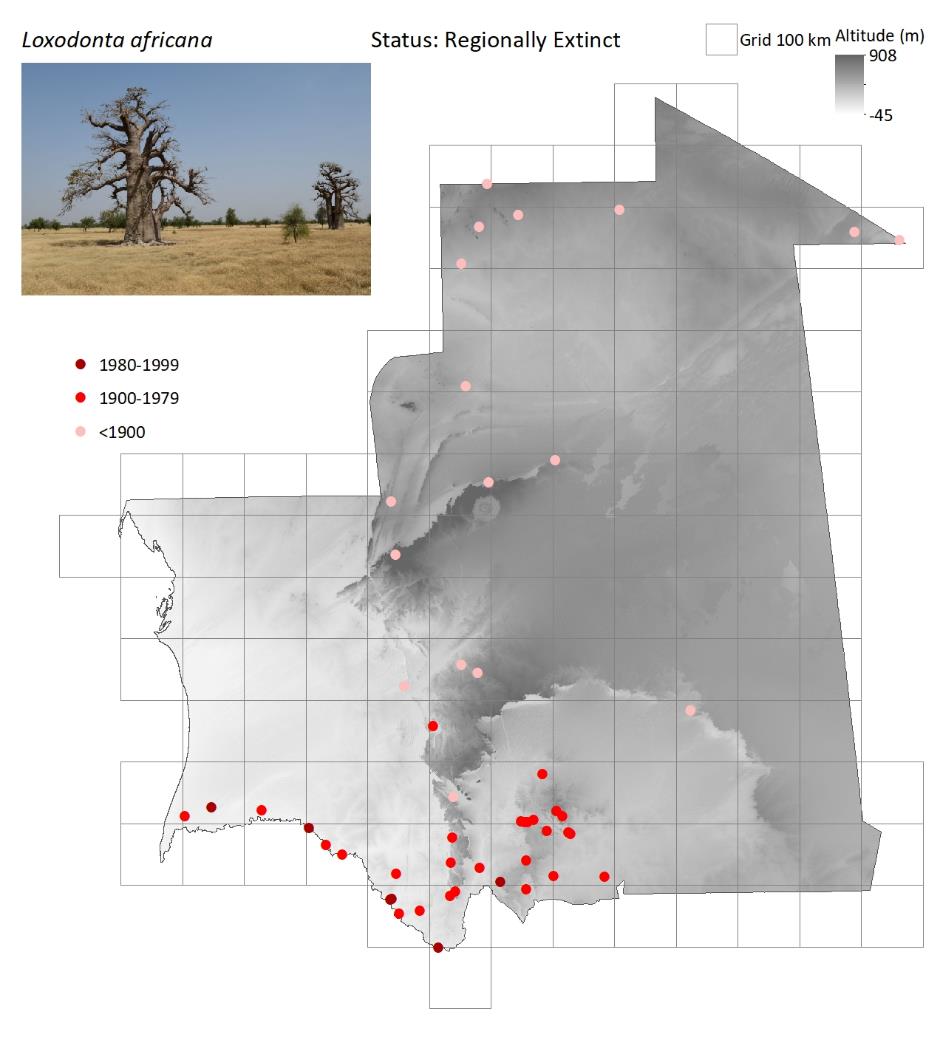


070 - Status and distribution of *Loxodonta africana* in Mauritania. Picture of the habitat near Sélibaby, Province Guidimaka (November 2020), where the species was known to occur until the 1970s (Dia 2004; Gueye and Dia 2004). No observations were made after that period.

Comparison with IUCN range polygons: Not possible. The reported range of the species does not include areas with extinct populations.

Conservation status: Global IUCN- EN; National final- RE; National original- RE.


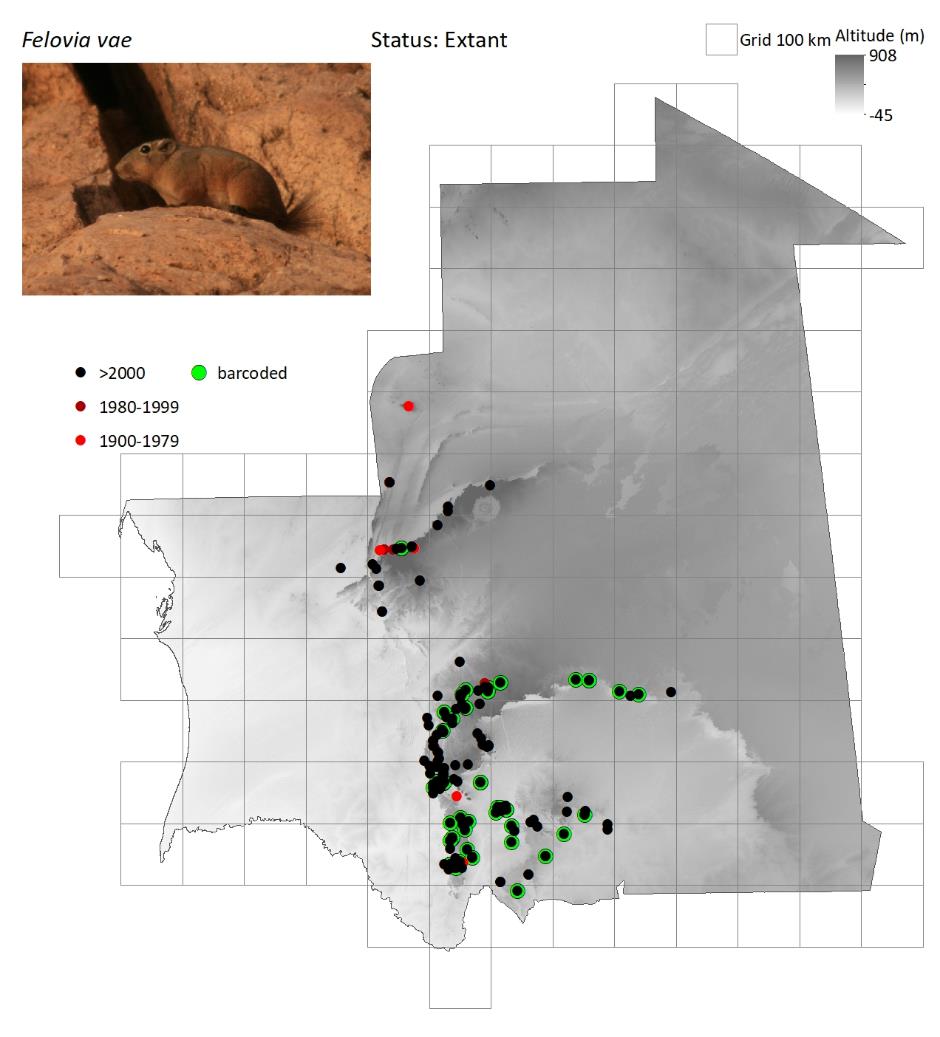


071 - Status and distribution of *Felovia vae* in Mauritania. Picture in *Guelta* Goumbel, Province of Guidimaka (November 2010).

Comparison with IUCN range polygons: No change. Mapped distribution is similar to reported range in Mauritania.

Conservation status: Global IUCN- LC; National final- LC; National original- LC.

Comment on the assessment: The species is nearly-endemic to Mauritania.


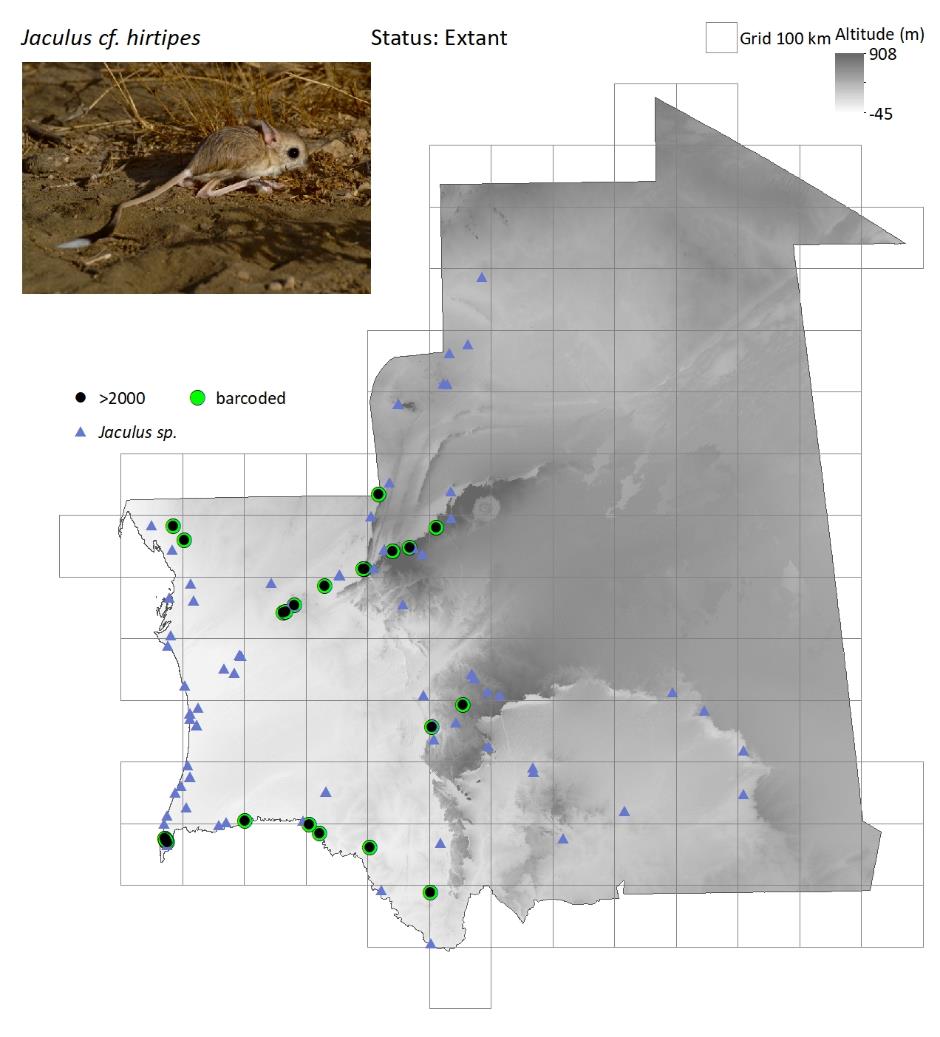


072 - Status and distribution of *Jaculus cf. hirtipes* in Mauritania. Picture in Toundou Hagui, Province of Trarza (January 2020). Non-barcoded observations of genus *Jaculus* are represented by triangles.

Comparison with IUCN range polygons: Uncertainty. The current taxonomic status of the species precludes range comparisons, as the species is presently not recognised by the Mammal Species of the World (Wilson and Reeder 2005) nor the IUCN Red List (IUCN 2021).

Conservation status: Global IUCN- NE; National final- LC; National original- LC.

Comment on the assessment: The taxonomic status of the species is pending a revaluation from the Mammal Species of the World (Wilson and Reeder 2005) and the IUCN Red List (IUCN 2021), as there is sufficient molecular evidence to consider it as full species (Shenbrot et al 2016; Boratyński et al 2017; Moutinho et al 2020).


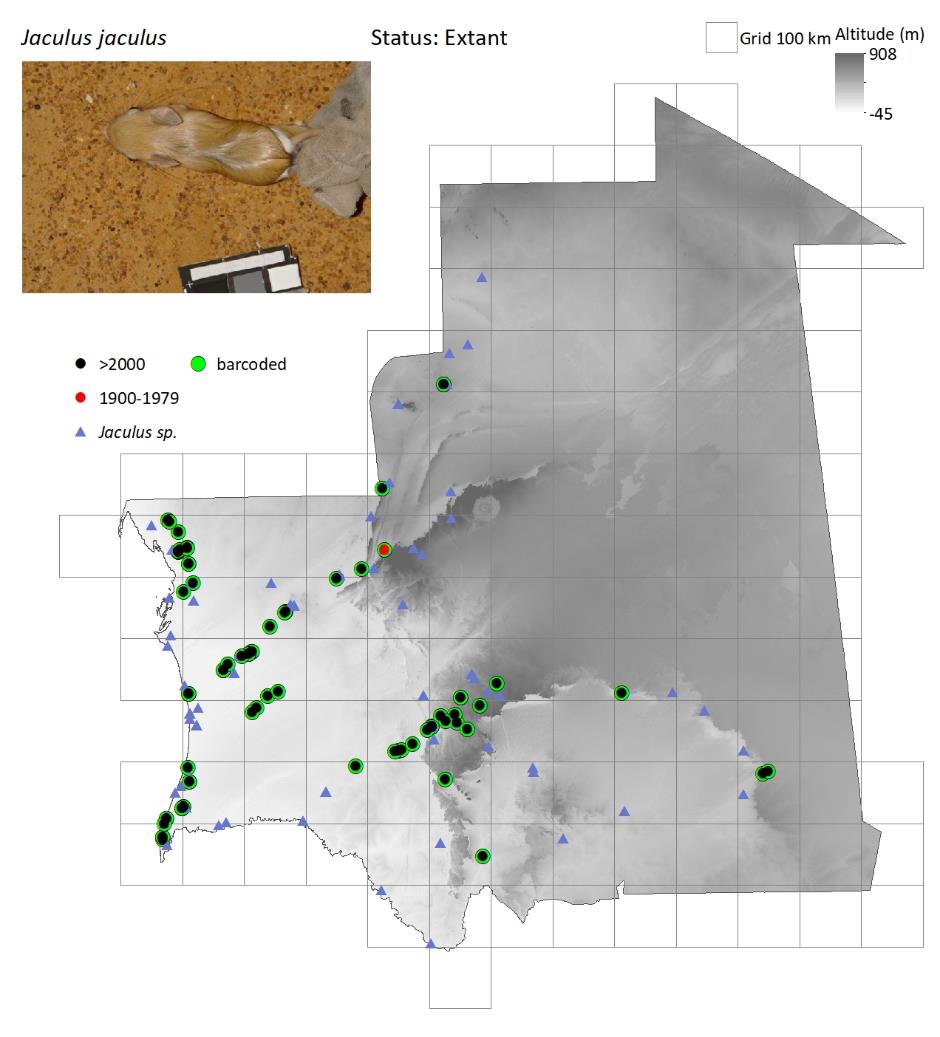


073 - Status and distribution of *Jaculus jaculus* in Mauritania. Picture near Nouakchott, Province of Trarza (January 2020). Non-barcoded observations of genus *Jaculus* are represented by triangles.

Comparison with IUCN range polygons: Range expansion. The barcoded observations expand the reported range of the species in Mauritania.

Conservation status: Global IUCN- LC; National final- LC; National original- LC.


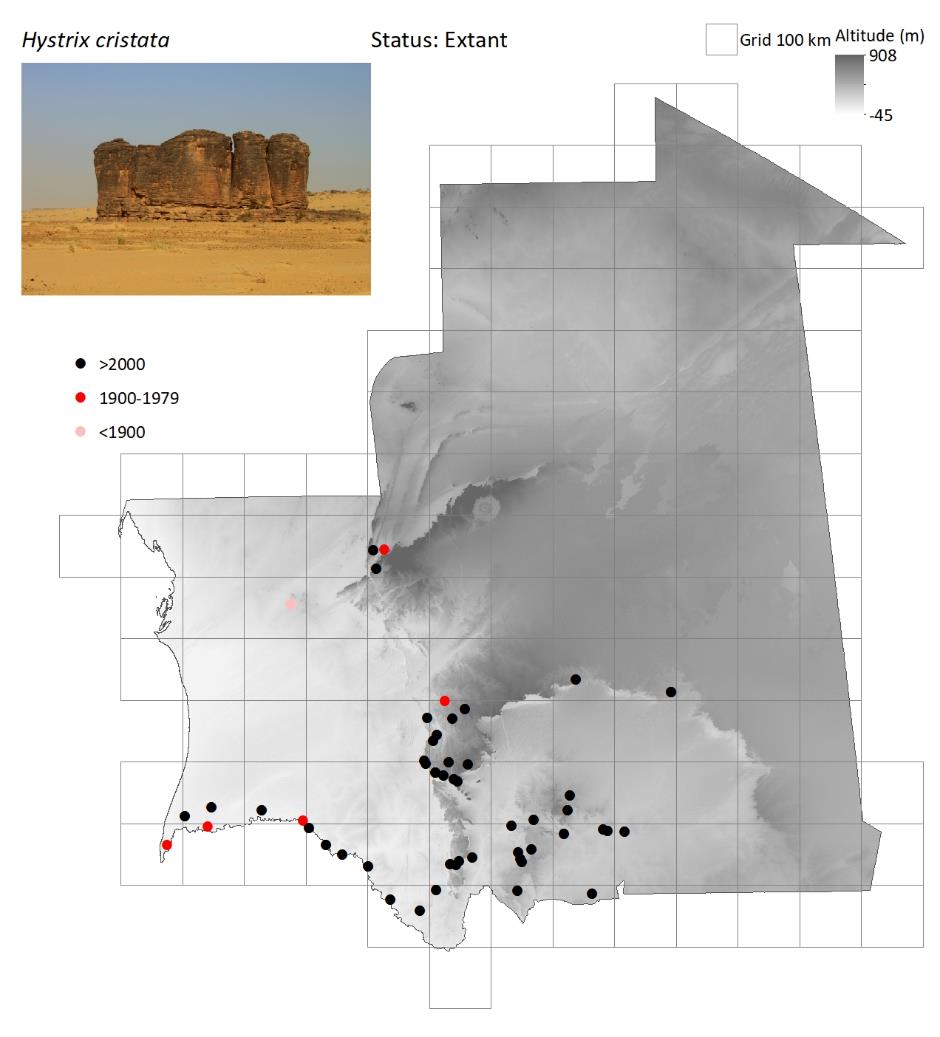


074 - Status and distribution of *Hystrix cristata* in Mauritania. Picture of the habitat in Guelb Massai, Province of Tagant (February 2014), where quills of the species were found by the authors.

Comparison with IUCN range polygons: Range expansion. The observations along the Senegal River valley and mountain plateaus and escarpments expand northwards the reported range of the species in Mauritania.

Conservation status: Global IUCN- LC; National final- LC; National original- LC.


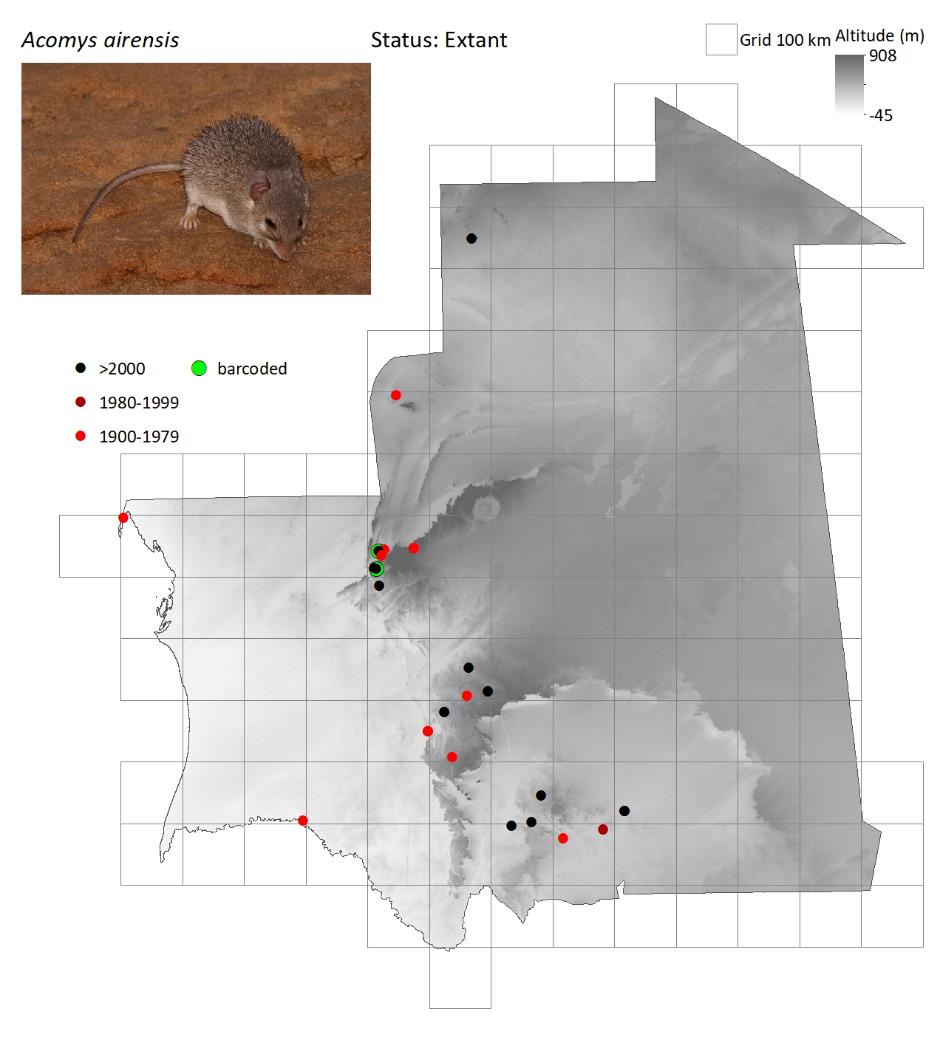


075 - Status and distribution of *Acomys airensis* in Mauritania. Picture in Ayoûn en Na'aj, Province of Assaba (January 2014).

Comparison with IUCN range polygons: Range expansion. The observations in the Adrar Atar plateau and Bir Moghrein expand northwards the reported range of the species in Mauritania.

Conservation status: Global IUCN- LC; National final- LC; National original- LC.


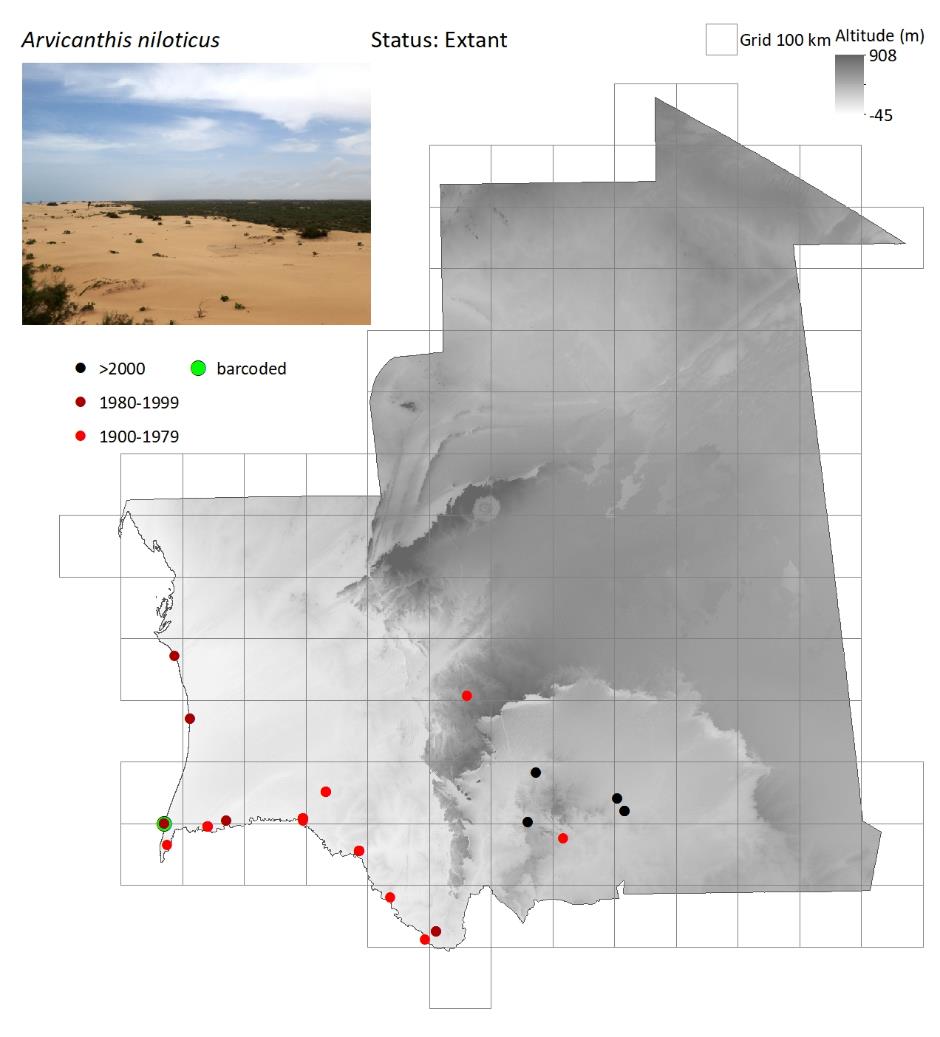


076 - Status and distribution of *Arvicanthis niloticus* in Mauritania. Picture of the habitat in Chott Boul, Province of Trarza (August 2015), where the species has been reported (Granjon et al 1997, 2002; Dobigny et al 2013).

Comparison with IUCN range polygons: Range expansion. The observation in the Tagant plateau expands northwards the reported range of the species in Mauritania. Still, the observation is from before the year 1980, and additional sampling is needed to understand the current range of the species.

Conservation status: Global IUCN- LC; National final- LC; National original- LC.


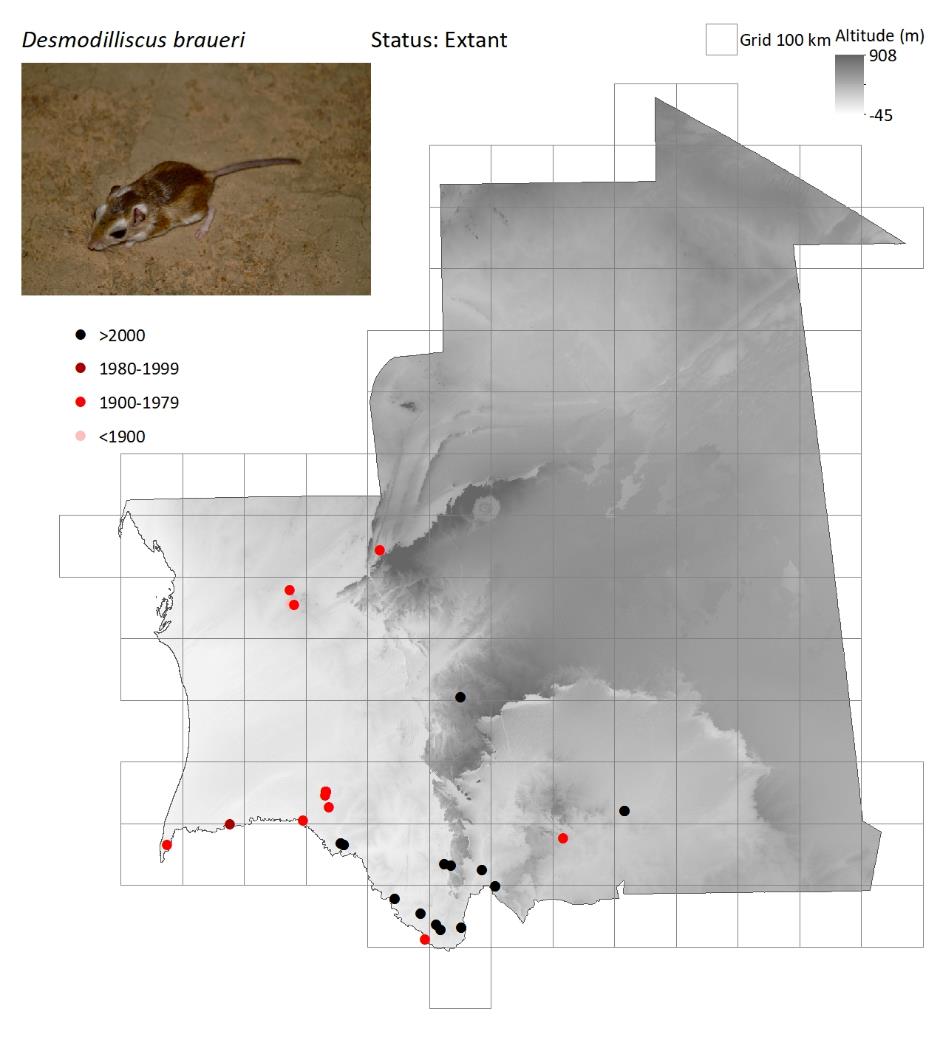


077 - Status and distribution of *Desmodilliscus braueri* in Mauritania. Picture near Maghama, Province of Gorgol (August 2015).

Comparison with IUCN range polygons: No change. Mapped distribution is similar to reported range in Mauritania.

Conservation status: Global IUCN- LC; National final- LC; National original- LC.


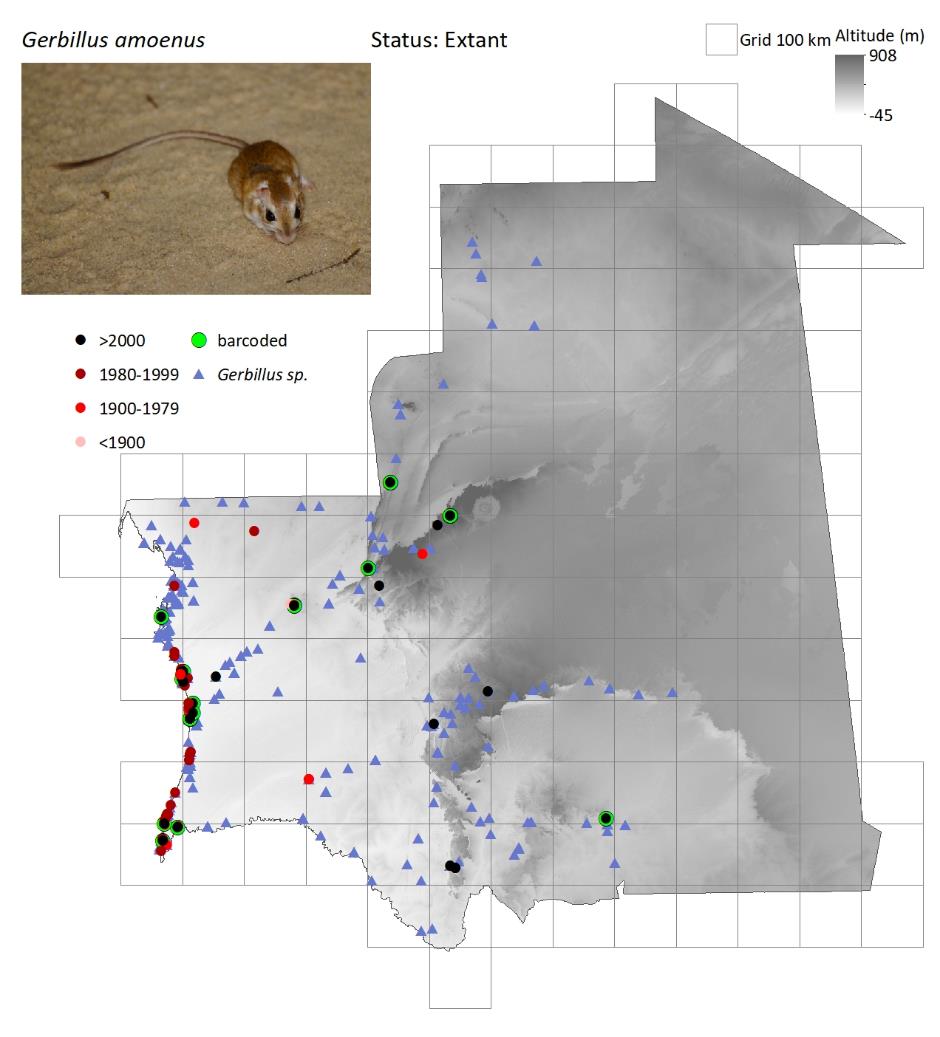


078 - Status and distribution of *Gerbillus amoenus* in Mauritania. Picture in Chott Boul, Province of Trarza (September 2015). Observations identified only up to genus level are represented by triangles.

Comparison with IUCN range polygons: Range expansion. The barcoded observations in the Adrar Atar and eastern Afollé plateaus expands eastwards the reported range of the species in Mauritania.

Conservation status: Global IUCN- LC; National final- LC; National original- LC.


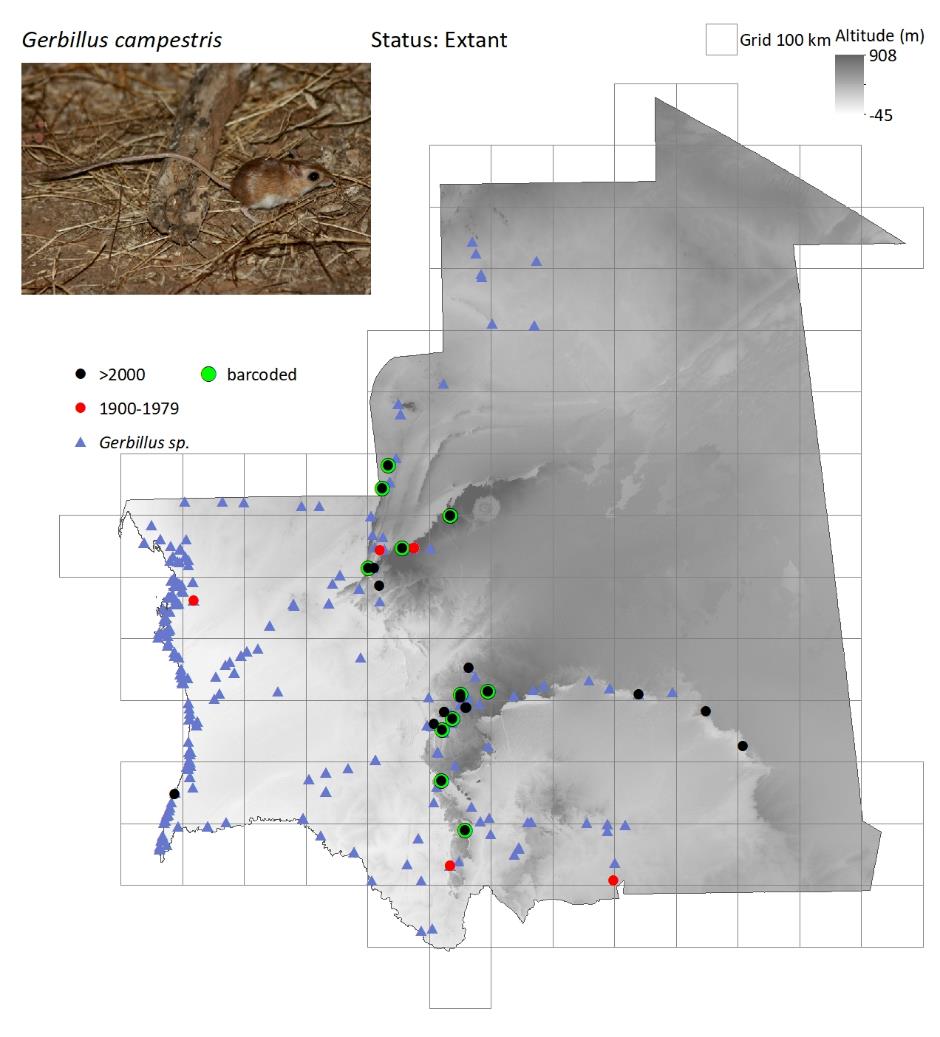


079 - Status and distribution of *Gerbillus campestris* in Mauritania. Picture near El Grâne, Province of Assaba (November 2012). Observations identified only up to genus level are represented by triangles.

Comparison with IUCN range polygons: New species for Mauritania. The barcoded observations in the Adrar Atar, Tagant and Afollé plateaus confirm the wide occurrence of the species in Mauritania and expand the reported range, as the closest known populations are from central Morocco.

Conservation status: Global IUCN- LC; National final- LC; National original- LC.


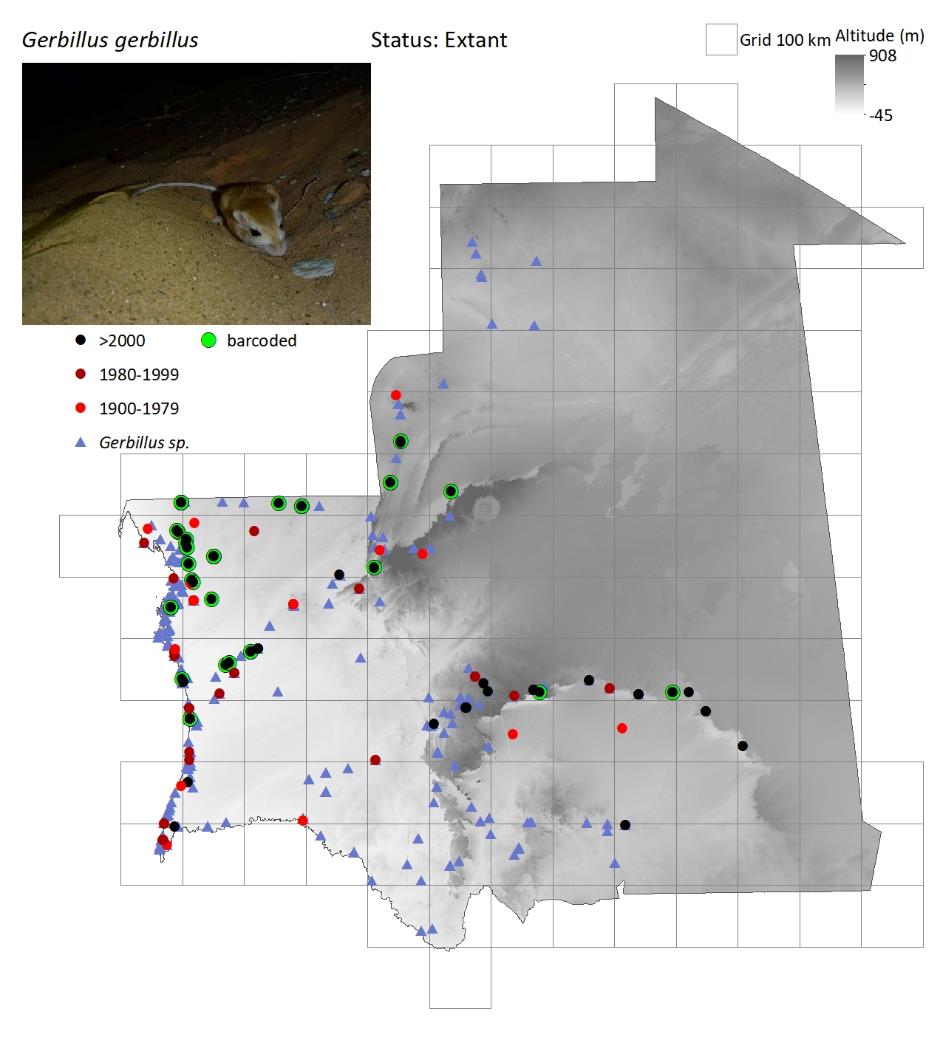


080 - Status and distribution of *Gerbillus gerbillus* in Mauritania. Picture in Nseirat, Province of Hodh Ech Chargui (January 2014). Observations identified only up to genus level are represented by triangles.

Comparison with IUCN range polygons: No change. Mapped distribution is similar to reported range in Mauritania.

Conservation status: Global IUCN- LC; National final- LC; National original- LC.


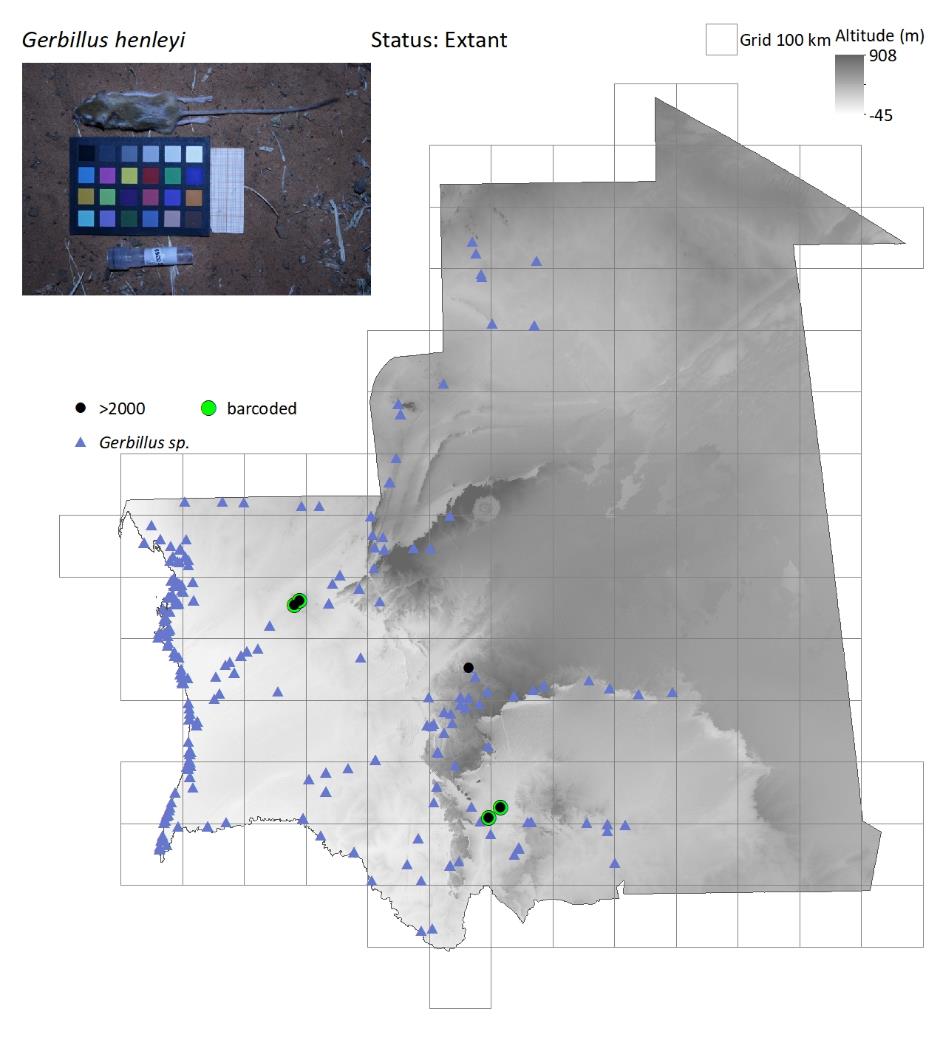


081 - Status and distribution of *Gerbillus henleyi* in Mauritania. Picture in Dâr Oulâd Tâleb, Province of Assaba (November 2012). Observations identified only up to genus level are represented by triangles.

Comparison with IUCN range polygons: Range expansion. The barcoded observations in Akjoujt expand northwards the reported range of the species in Mauritania.

Conservation status: Global IUCN- LC; National final- LC; National original- LC.


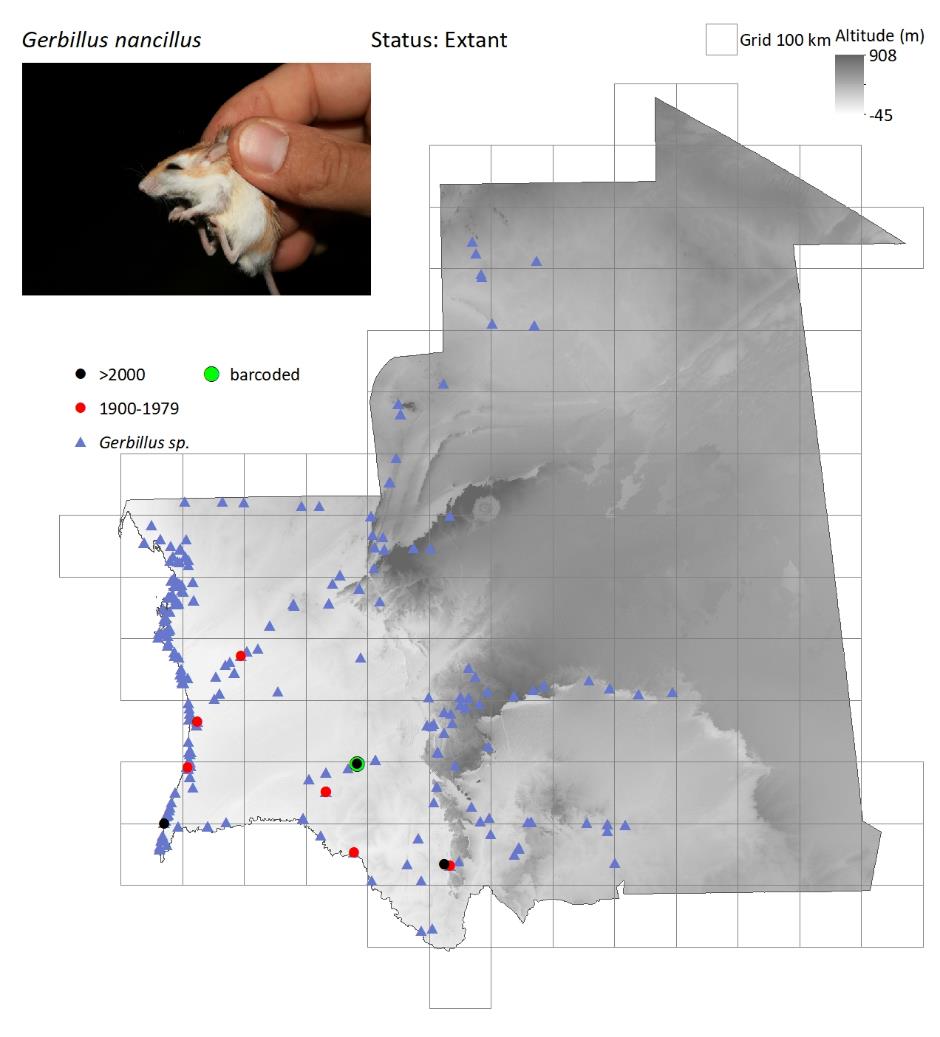


082 - Status and distribution of *Gerbillus nancillus* in Mauritania. Picture in about 20 km north-east of Chogâr, Province of Brakna (November 2010). Observations identified only up to genus level are represented by triangles.

Comparison with IUCN range polygons: No change. Mapped distribution is similar to reported range in Mauritania.

Conservation status: Global IUCN- DD; National final- LC; National original- LC.


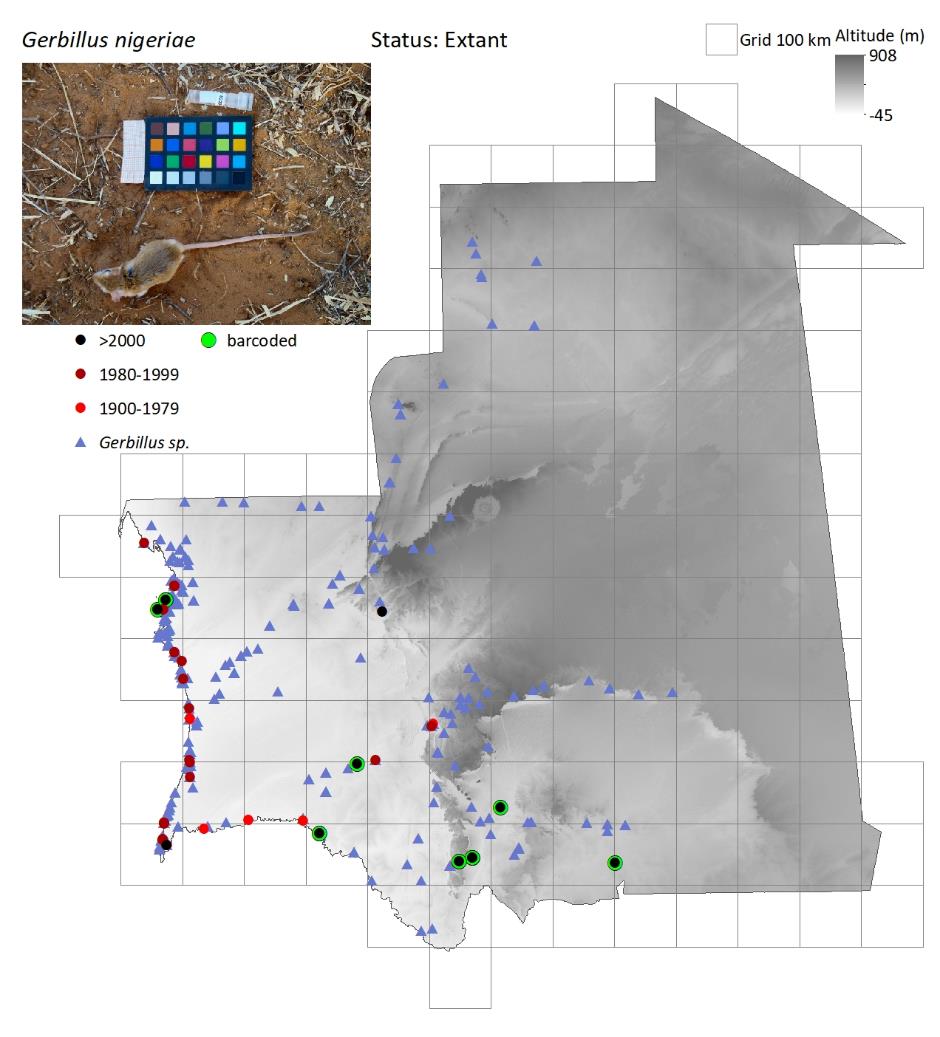


083 - Status and distribution of *Gerbillus nigeriae* in Mauritania. Picture in Oued El Ouadhâne, Province of Assaba (November 2012). Observations identified only up to genus level are represented by triangles.

Comparison with IUCN range polygons: Range expansion. The barcoded observations in south and eastern Mauritania expand eastwards the reported range of the species in Mauritania.

Conservation status: Global IUCN- LC; National final- LC; National original- LC.

Comment on the assessment: The species is endemic to Mauritania.


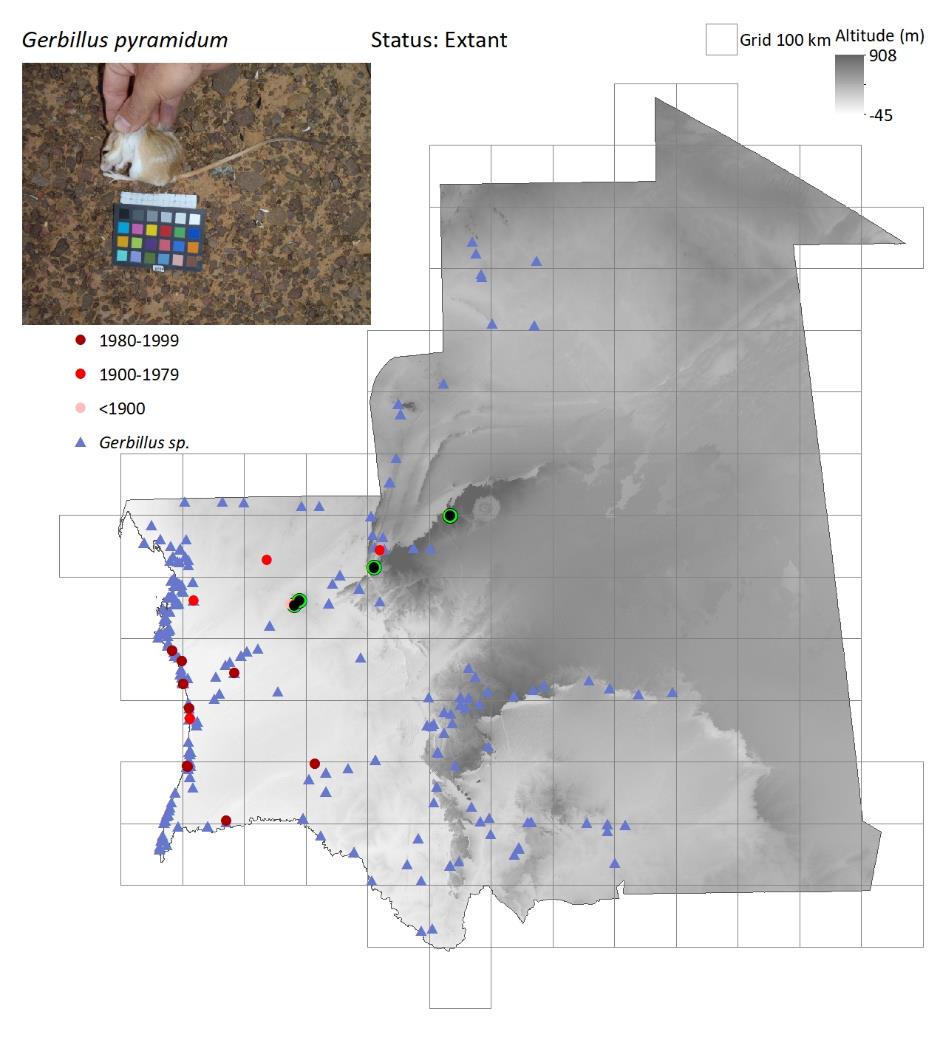


084 - Status and distribution of *Gerbillus pyramidum* in Mauritania. Picture in Kkneg el Gouadim, Province of Adrar (November 2011). Observations identified only up to genus level are represented by triangles.

Comparison with IUCN range polygons: No change. Mapped distribution is similar to reported range in Mauritania.

Conservation status: Global IUCN- LC; National final- LC; National original- LC.


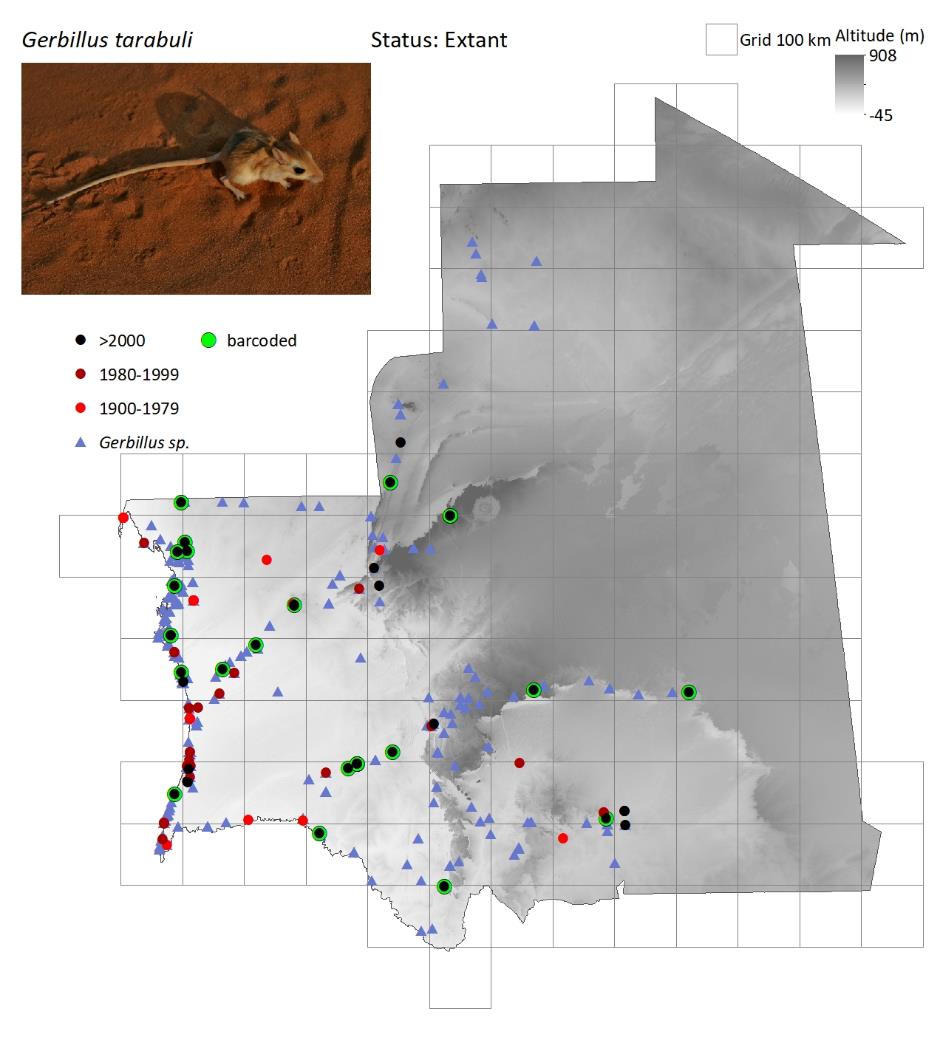


085 - Status and distribution of *Gerbillus tarabuli* in Mauritania. Picture about 20 km north-east of Chogâr, Province of Brakna (November 2010). Observations identified only up to genus level are represented by triangles.

Comparison with IUCN range polygons: No change. Mapped distribution is similar to reported range in Mauritania.

Conservation status: Global IUCN- LC; National final- LC; National original- LC.


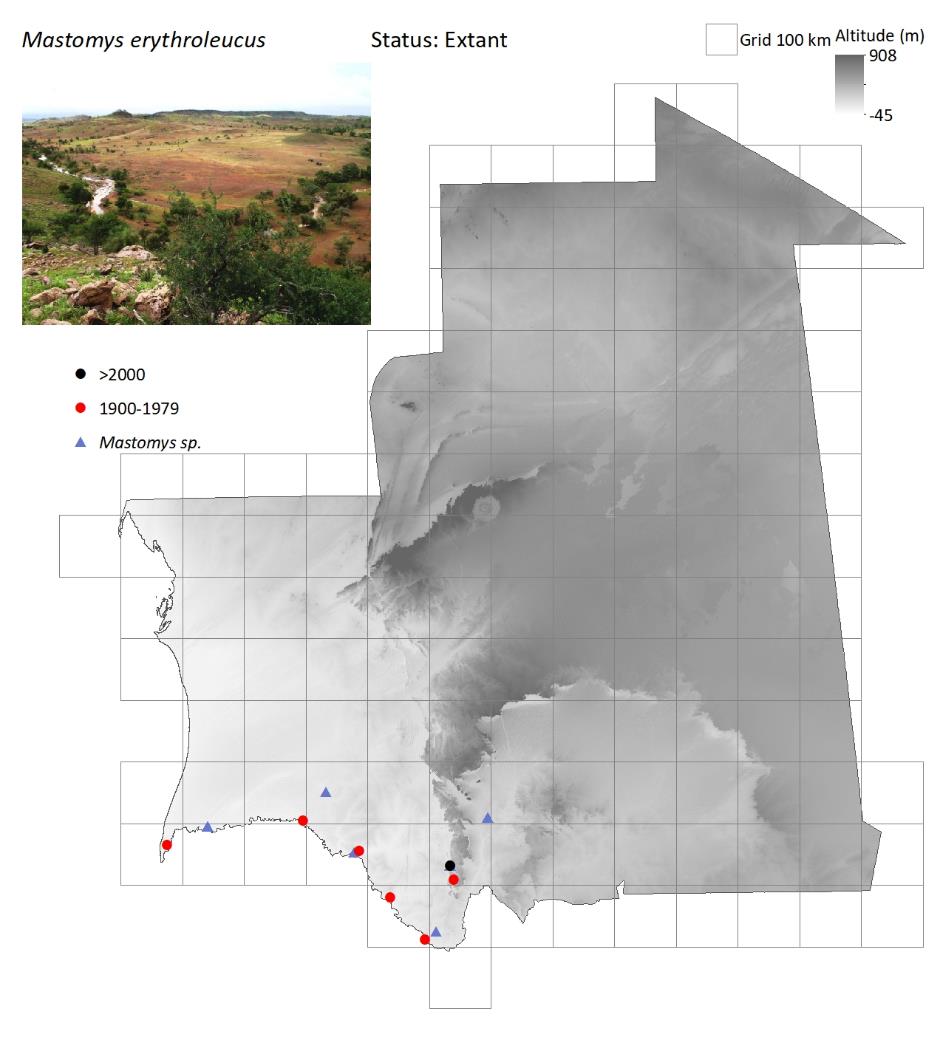


086 - Status and distribution of *Mastomys erythroleucus* in Mauritania. Picture of the habitat in Soufa, Province of Guidimaka (August 2015), where the species has been reported (Diatta et al 2015). Observations identified only up to genus level are represented by triangles.

Comparison with IUCN range polygons: No change. Mapped distribution is similar to reported range in Mauritania.

Conservation status: Global IUCN- LC; National final- LC; National original- LC.


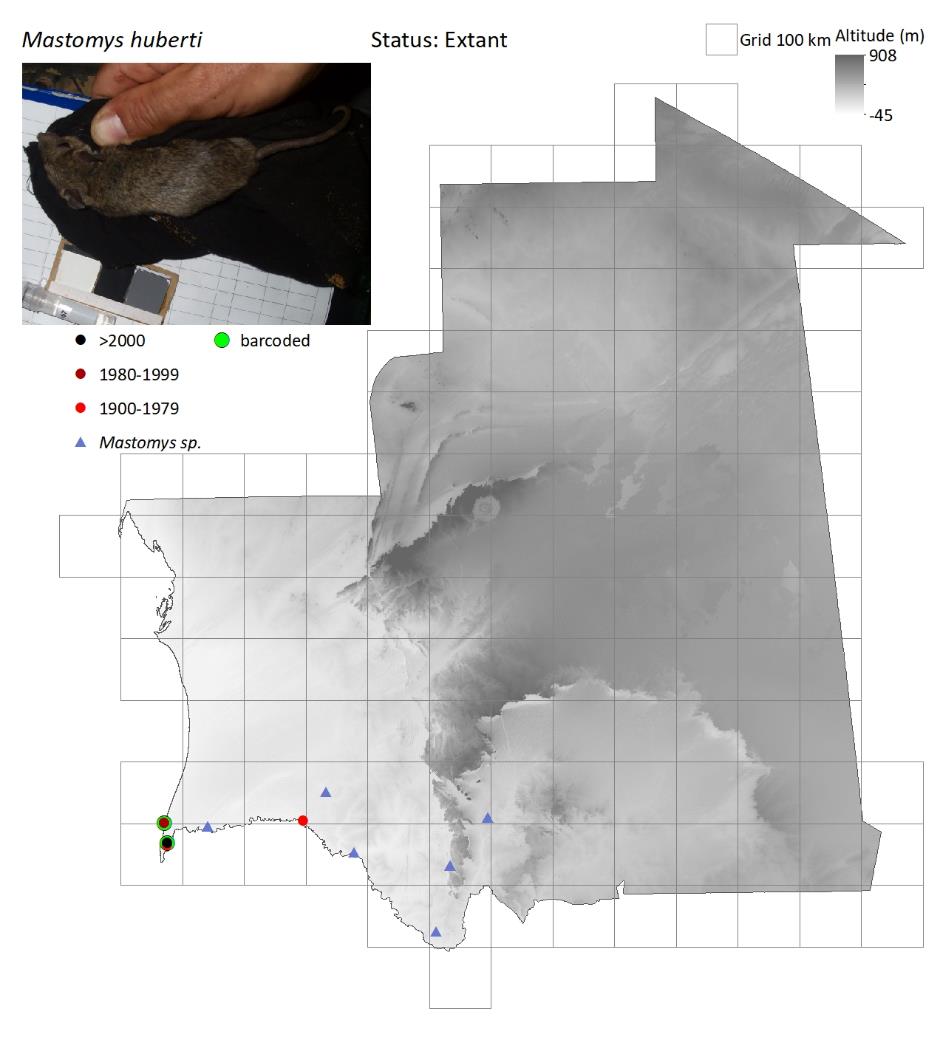


087 - Status and distribution of *Mastomys huberti* in Mauritania. Picture in Bell, Province of Trarza (January 2020). Observations identified only up to genus level are represented by triangles.

Comparison with IUCN range polygons: No change. Mapped distribution is similar to reported range in Mauritania.

Conservation status: Global IUCN- LC; National final- LC; National original- LC.


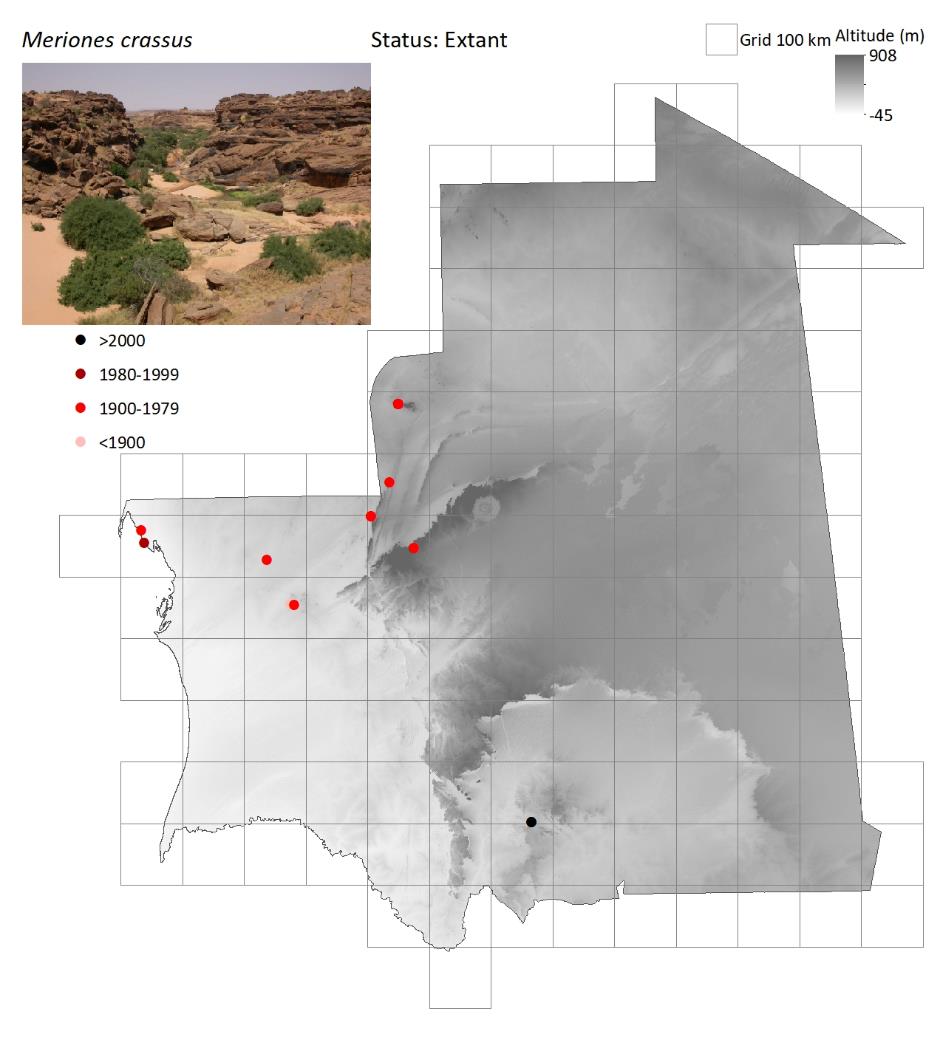


088 - Status and distribution of *Meriones crassus* in Mauritania. Picture of the habitat in *Guelta* Metraoucha, Province of Hodh El Gharbi (October 2008), where the species has been reported (Nickel 2003).

Comparison with IUCN range polygons: Range expansion. All observations, particularly the recent one in the central Afollé plateau, expand southwards the reported range of the species in Mauritania.

Conservation status: Global IUCN- LC; National final- LC; National original- LC.


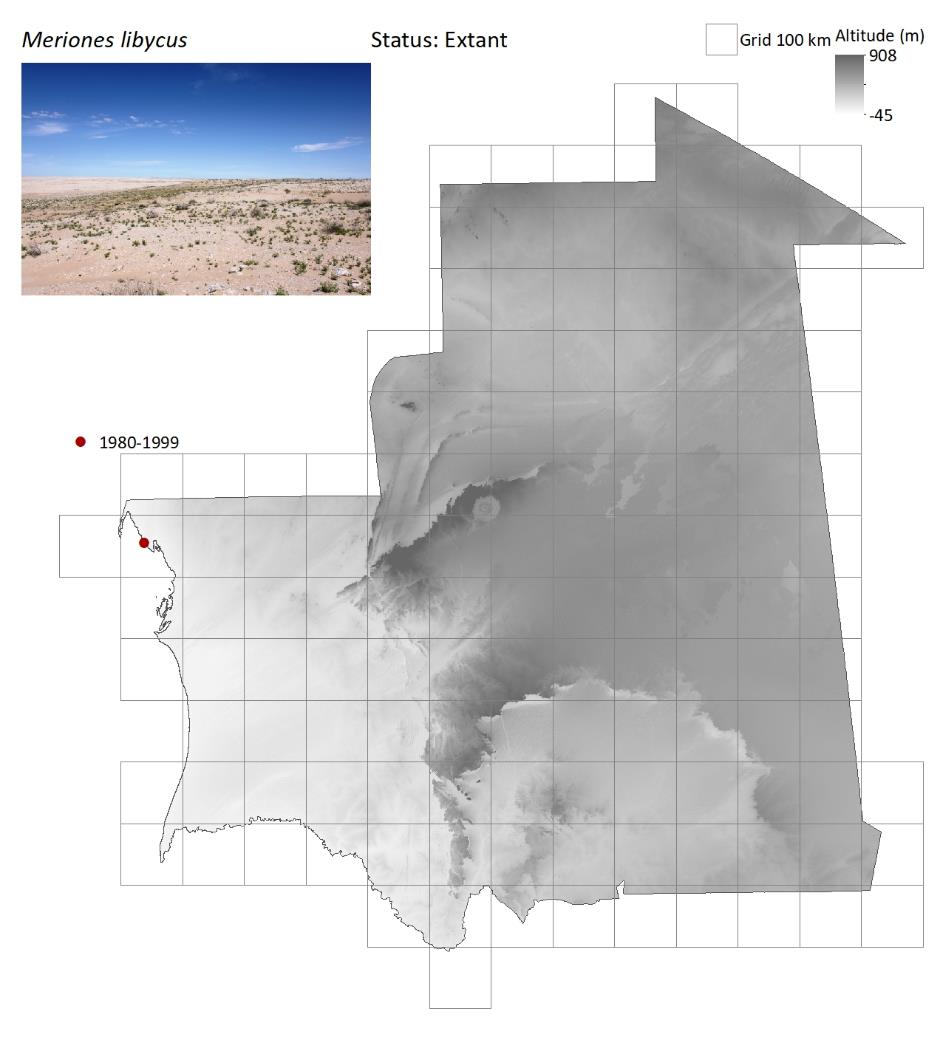


089 - Status and distribution of *Meriones libycus* in Mauritania. Picture near Cap Sainte-Anne, Province of Dakhlet-Nouâdhibou (November 2010), where the species has been reported (Granjon et al 1997).

Comparison with IUCN range polygons: Range expansion. The observation in the Cap Sainte-Anne slightly expands south-westwards the reported range of the species in Mauritania.

Conservation status: Global IUCN- LC; National final- DD; National original- DD.

Comment on the assessment: There is only one observation available from before the year 2000.


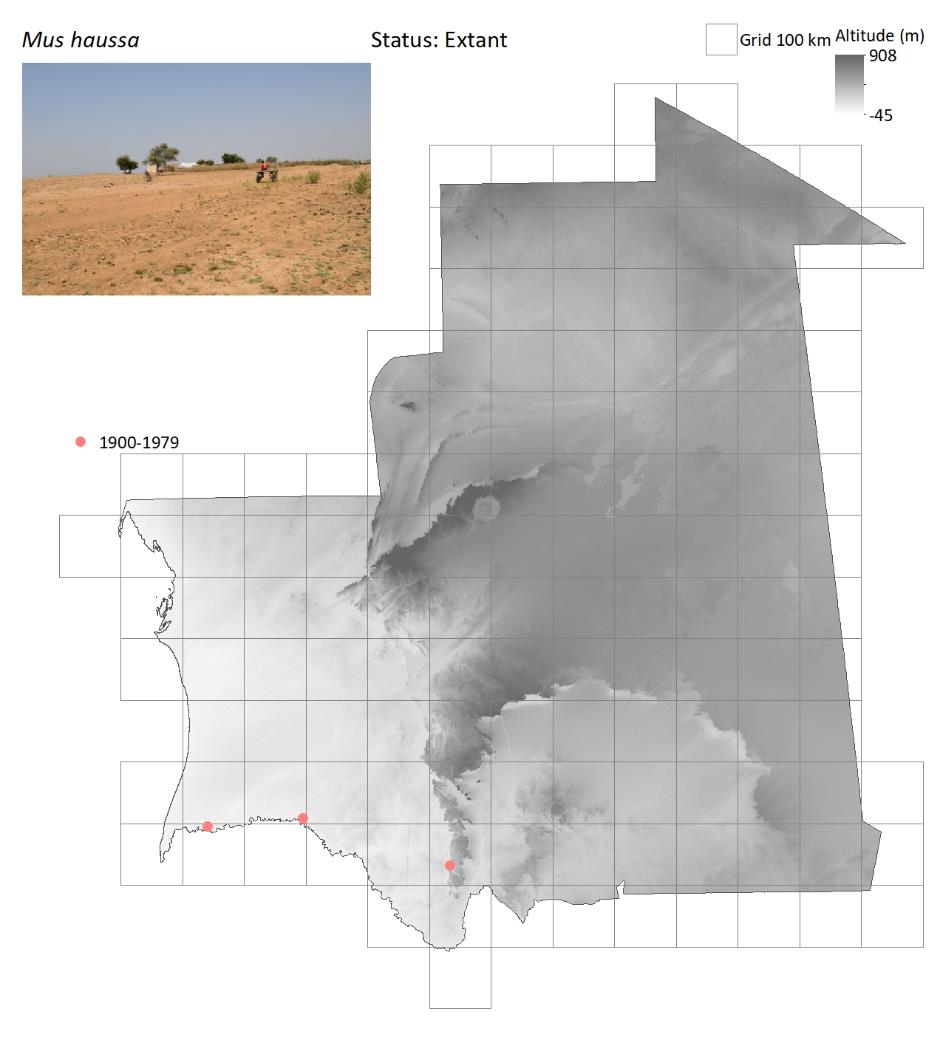


090 - Status and distribution of *Mus haussa* in Mauritania. Picture of the habitat near Boghé, Province of Brakna (November 2012), where the species has been reported (GBIF.org 2021).

Comparison with IUCN range polygons: No change. Mapped distribution is similar to reported range in Mauritania.

Conservation status: Global IUCN- LC; National final- LC; National original- LC.


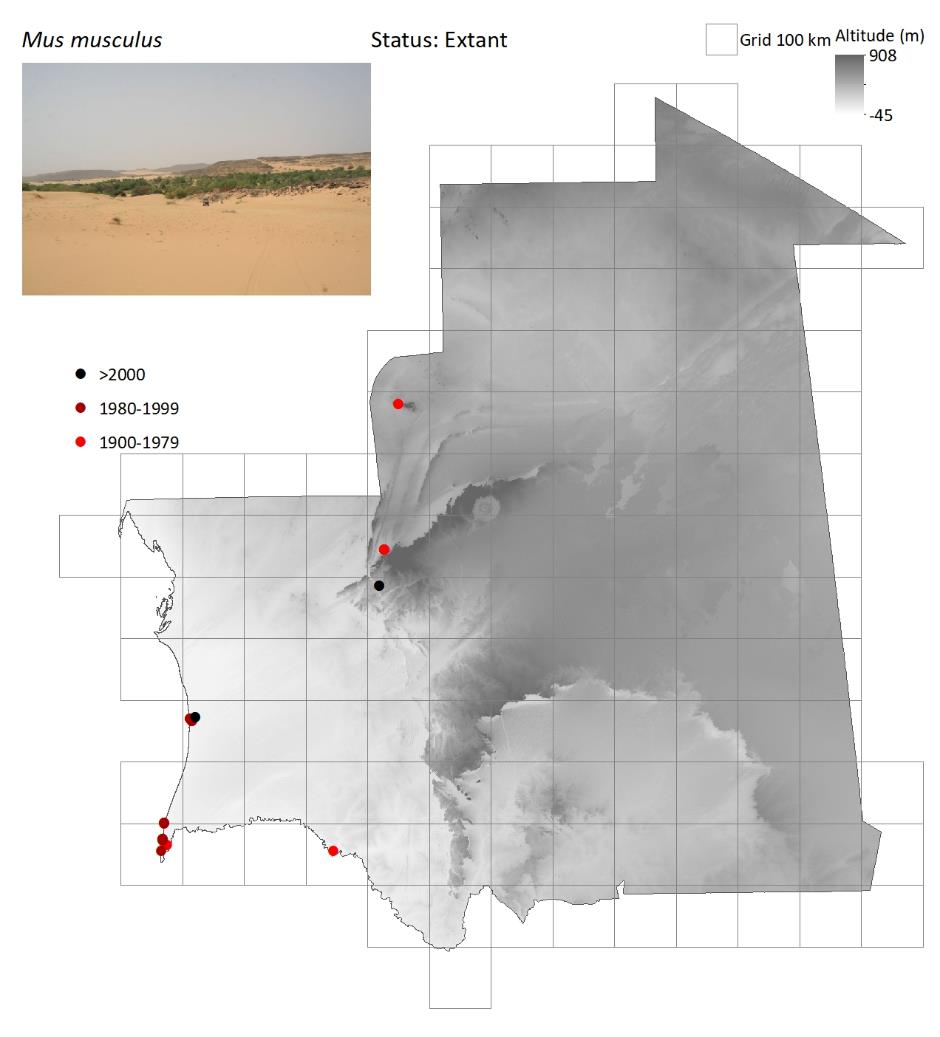


091 - Status and distribution of *Mus musculus* in Mauritania. Picture of the habitat in Oujeft, Province of Adrar (October 2008), where the species has been reported (Diatta et al 2015).

Comparison with IUCN range polygons: New non-native species for Mauritania. The observations collected confirm the occurrence of the species in Mauritania and expand the reported range, as the closest known populations are from central Morocco.

Conservation status: Global IUCN- LC; National final- NA; National original- NA.

Comment on the assessment: The species is non-native and introduced to Mauritania which precludes the assessment.


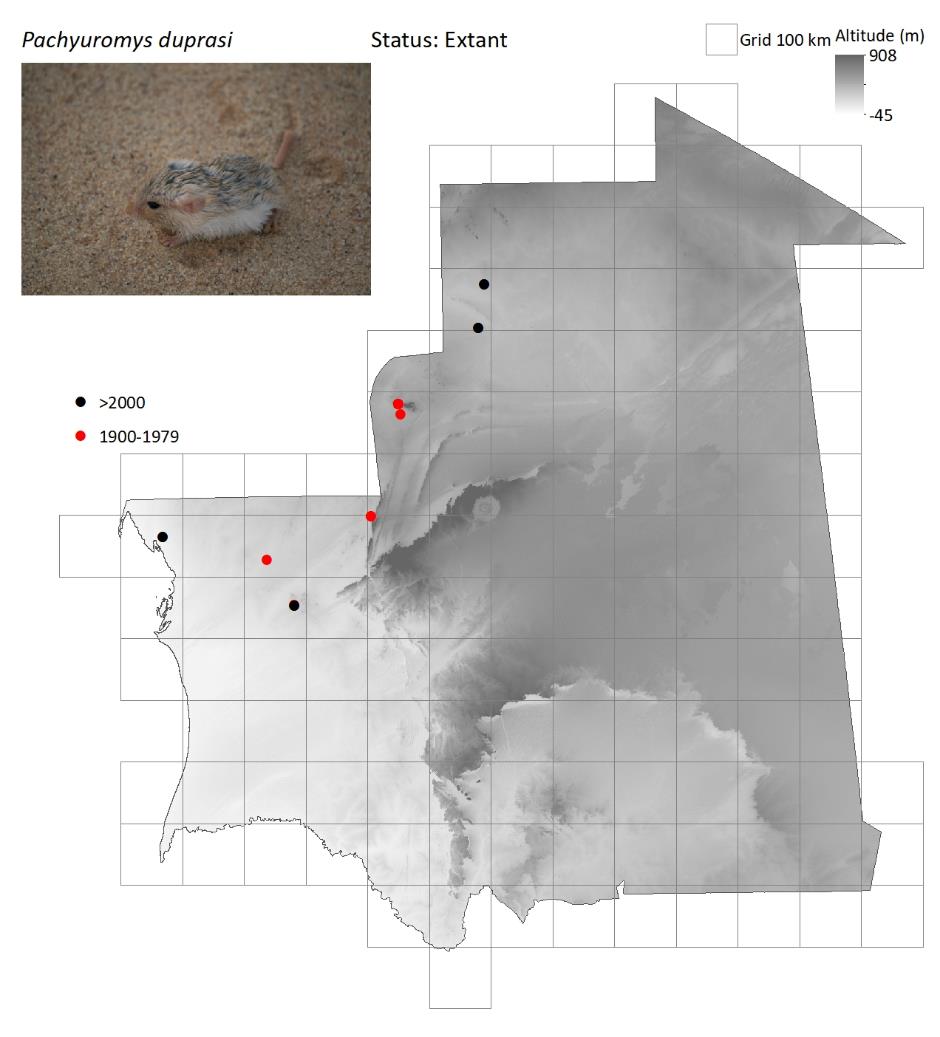


092 - Status and distribution of *Pachyuromys duprasi* in Mauritania. Picture in Amgheououas es Sâhli, Province of Dakhlet-Nouâdhibou (November 2010).

Comparison with IUCN range polygons: Range expansion. The observations in northern Mauritania and Akjoujt expand north-eastwards the reported range of the species in Mauritania.

Conservation status: Global IUCN- LC; National final- LC; National original- LC.


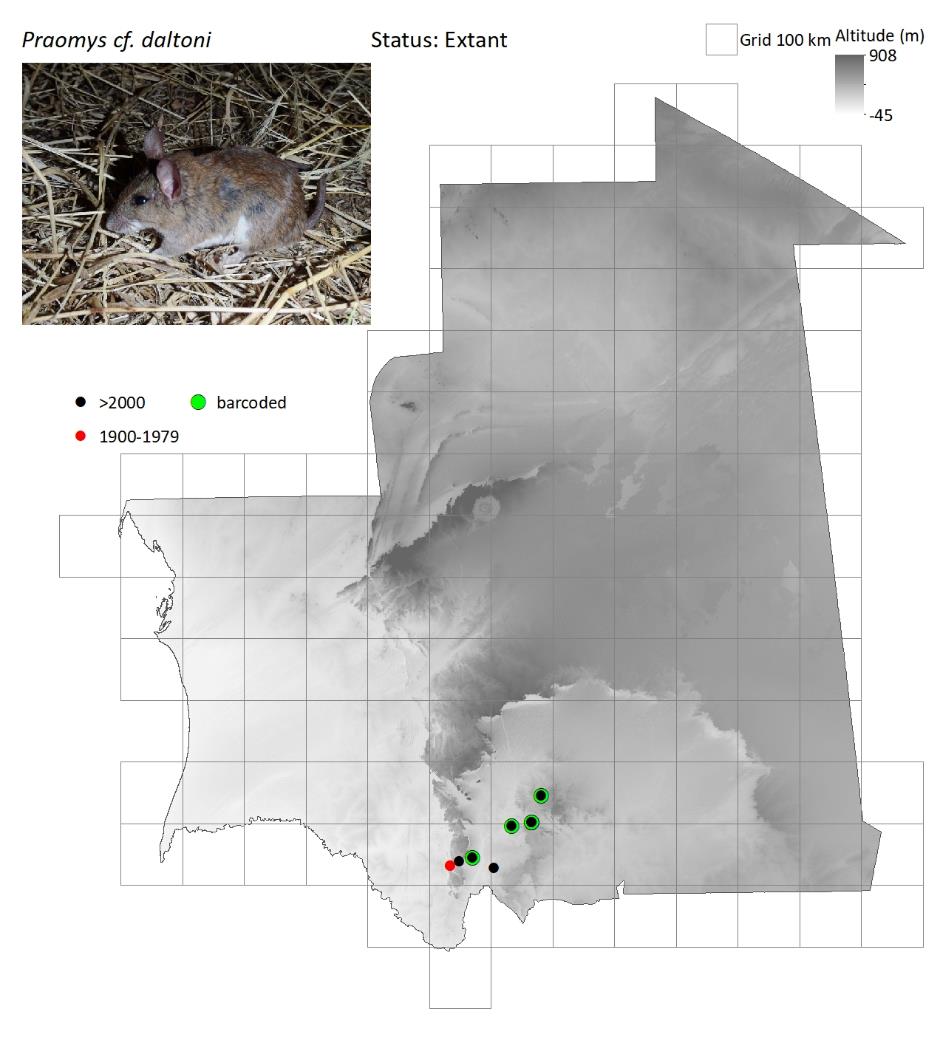


093 - Status and distribution of *Praomys cf. daltoni* in Mauritania. Picture in Oued El Ouadhâne, Province of Assaba (November 2012).

Comparison with IUCN range polygons: Uncertainty. The observations in the Assaba and Afollé plateaus expand northwards the reported range of the species in Mauritania (IUCN 2021). However, the taxonomic status of Mauritanian populations deserved further investigation as they might represent an unrecognized species (Mikula et al 2020).

Conservation status: Global IUCN- NE; National final- VU B1a,b(iii); National original- VU.

Comment on the assessment: New endemic species to Mauritania still to be described for which there is recent molecular evidence supporting species status (Mikula et al 2020). Downgrading due to the potential rescue effect from populations in Mali and Senegal was not considered given the genetic isolation of Mauritanian populations (Mikula et al 2020). Inferred continuous decline in habitat quality due to overgrazing and frequent drought.


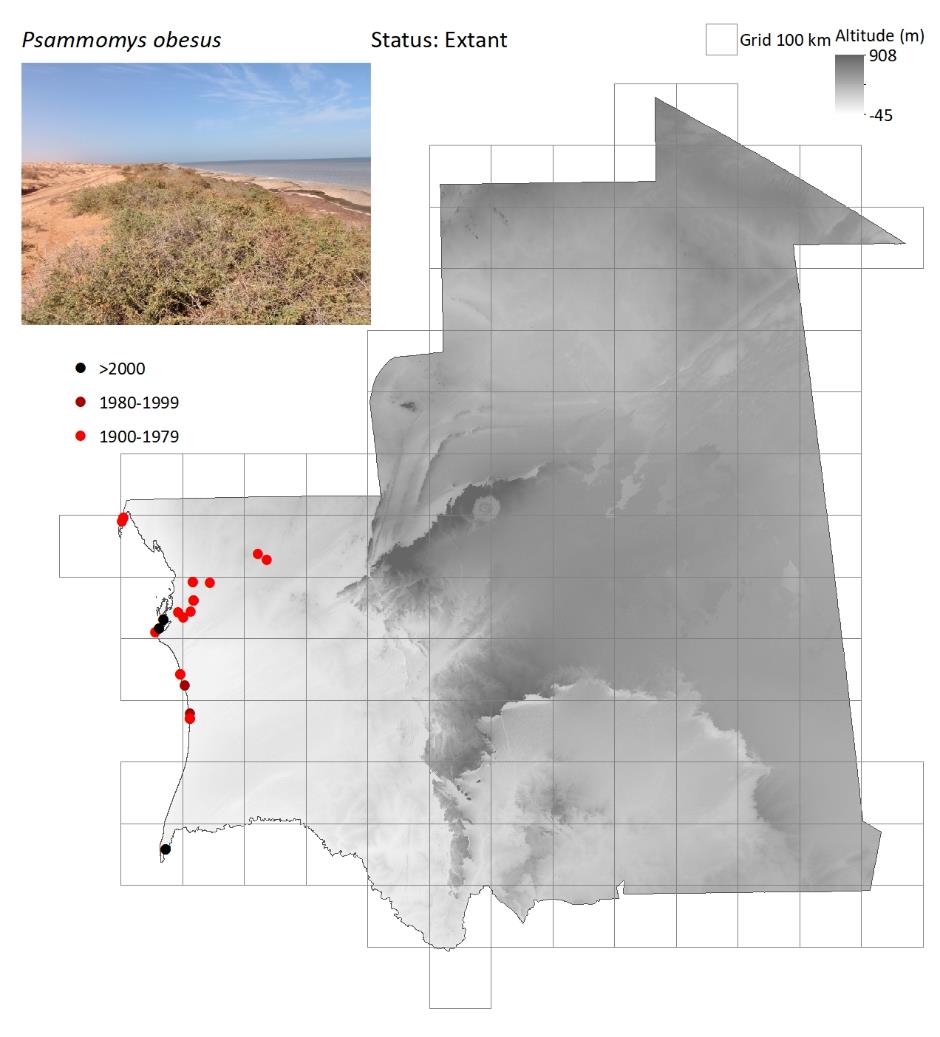


094 - Status and distribution of *Psammomys obesus* in Mauritania. Picture of the habitat near Teichott, Province of Dakhlet-Nouâdhibou (May 2009), where the species was observed.

Comparison with IUCN range polygons: Range expansion. The observation in the Diawling National Park expands southwards the reported range of the species in Mauritania.

Conservation status: Global IUCN- LC; National final- LC; National original- LC.


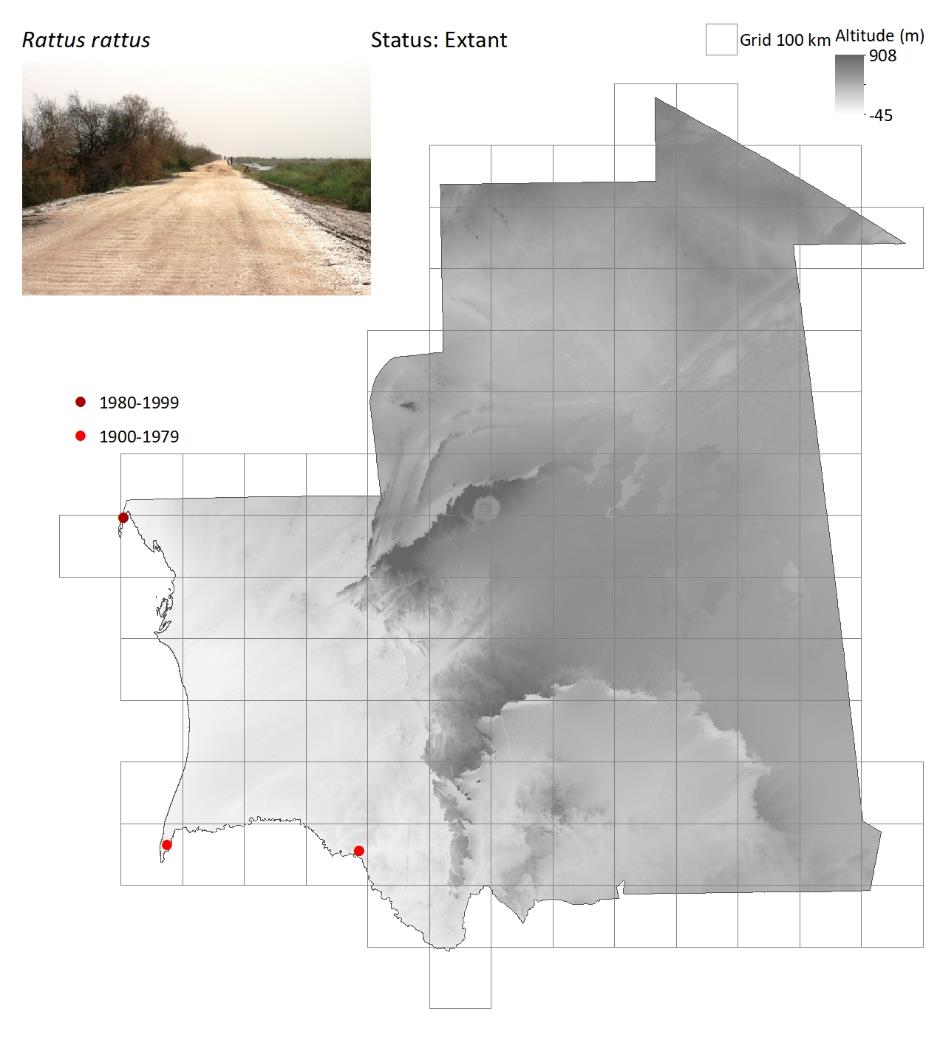


095 - Status and distribution of *Rattus rattus* in Mauritania. Picture of the habitat in the Senegal River delta, Province of Trarza (October 2010), where the species has been reported (National Research Council 1981).

Comparison with IUCN range polygons: New non-native species for Mauritania. The observations confirm the occurrence of the species in Mauritania and expand the reported range, as the closest known populations are from central Morocco and southern Senegal. Still, available observations are from before the year 2000, and additional sampling is needed to understand the current range of the species.

Conservation status: Global IUCN- LC; National final- NA; National original- NA.

Comment on the assessment: The species is non-native and introduced to Mauritania which precludes the assessment.


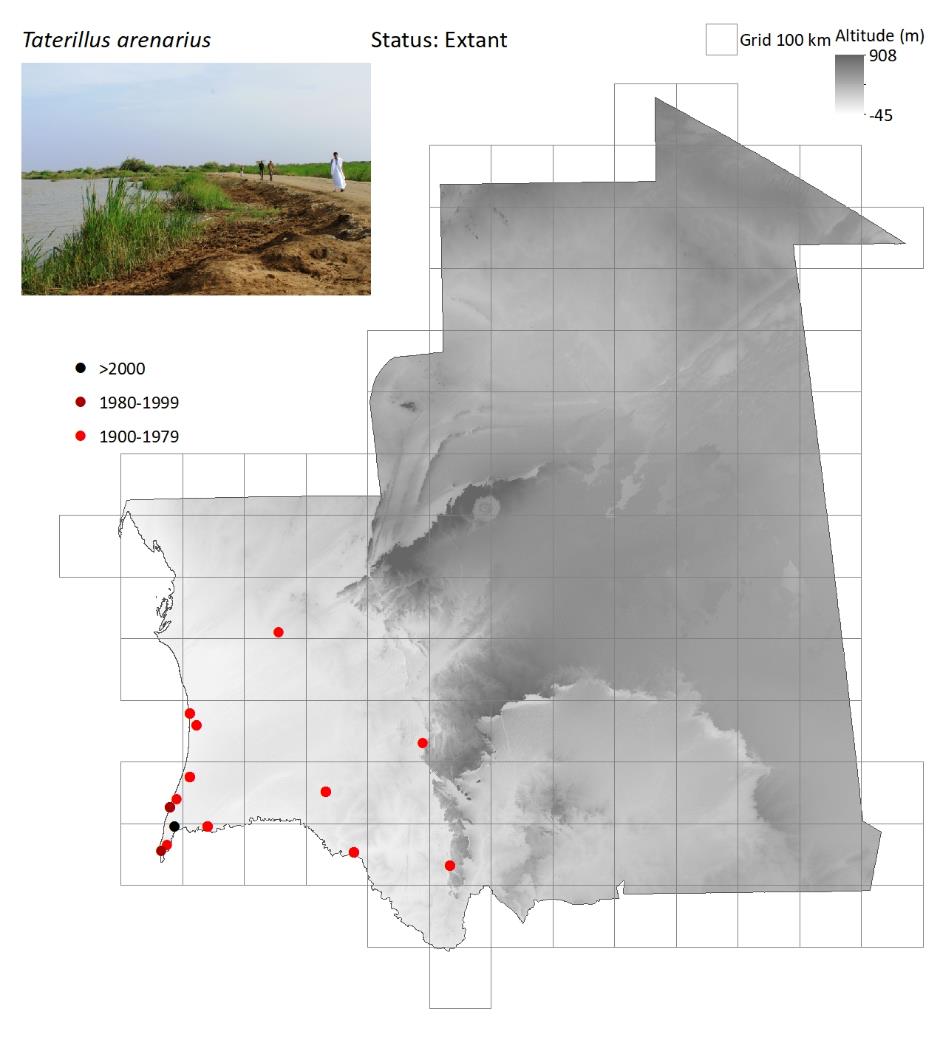


096 - Status and distribution of *Taterillus arenarius* in Mauritania. Picture of the habitat near Keur Massene, Province of Trarza (November 2012), where the species has been reported (Diatta et al 2015).

Comparison with IUCN range polygons: Range expansion. The observations in the Senegal River Delta expand southwards the reported range of the species in Mauritania.

Conservation status: Global IUCN- LC; National final- LC; National original- LC.


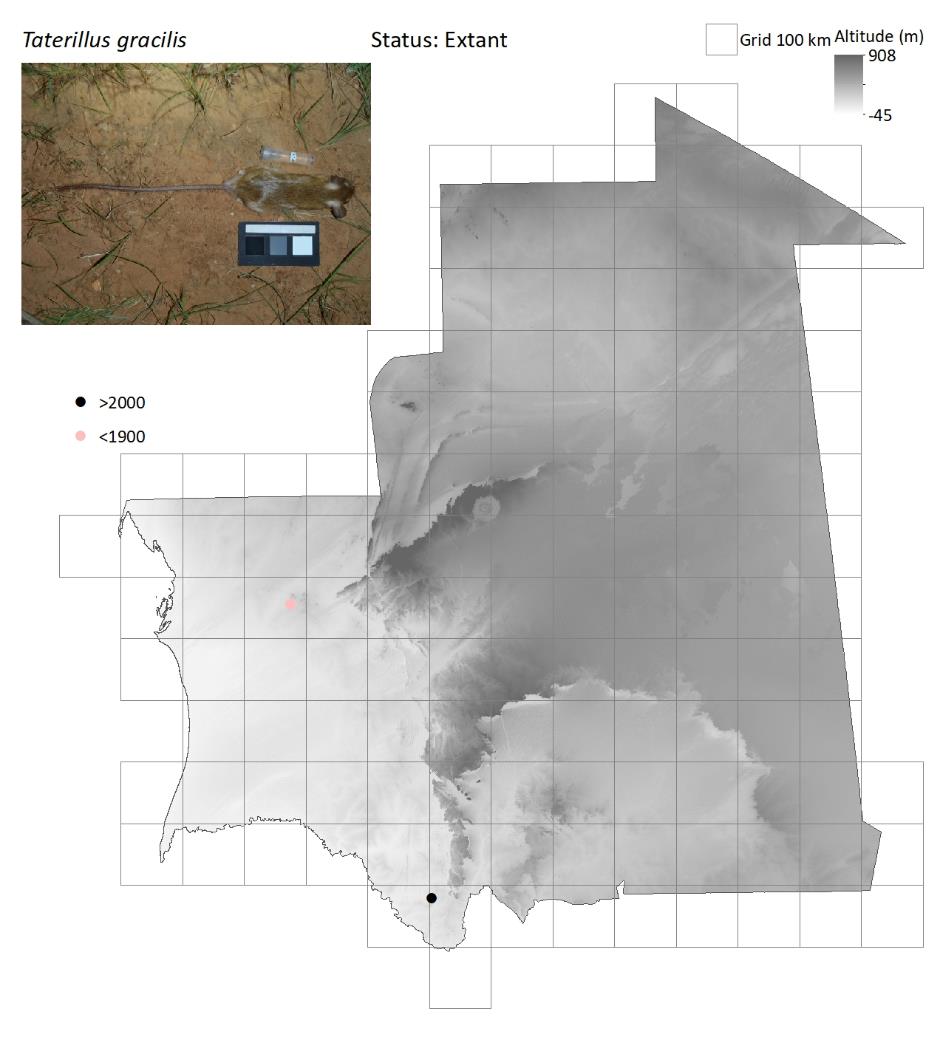


097 - Status and distribution of *Taterillus gracilis* in Mauritania. Picture in Artémou, Province of Guidimaka (August 2015).

Comparison with IUCN range polygons: Range expansion. The recent observation in Artémou slightly expands northwards the reported range of the species in Mauritania.

Conservation status: Global IUCN- LC; National final- DD; National original- DD.

Comment on the assessment: There are only two observations available.


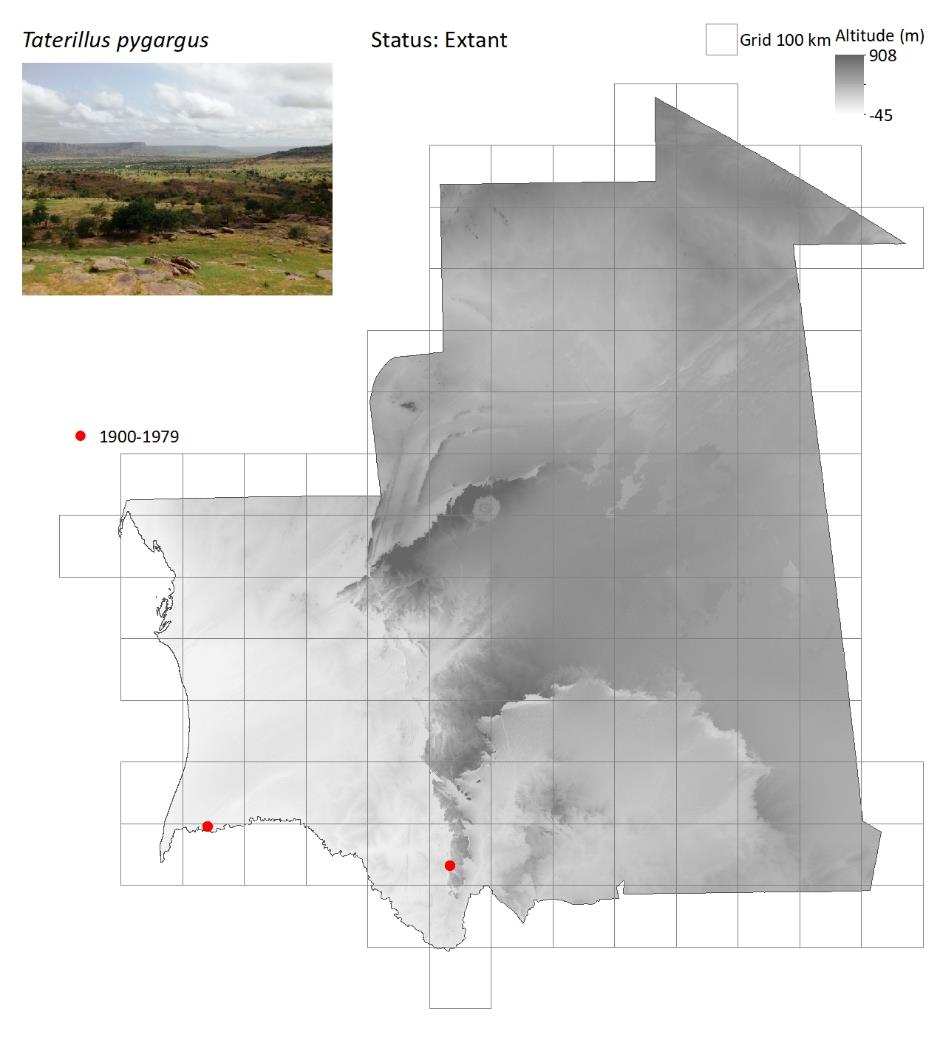


098 - Status and distribution of *Taterillus pygargus* in Mauritania. Picture of the habitat near Pass de Soufa, Province of Guidimaka (September 2015), where the species has been reported (GBIF.org 2021).

Comparison with IUCN range polygons: Range expansion. The observation in Pass de Soufa (Province of Guidimaka) expands eastward the reported range of the species in Mauritania.

Conservation status: Global IUCN- LC; National final- DD; National original- DD.

Comment on the assessment: There are only two distinct observations available from before the year 2000.


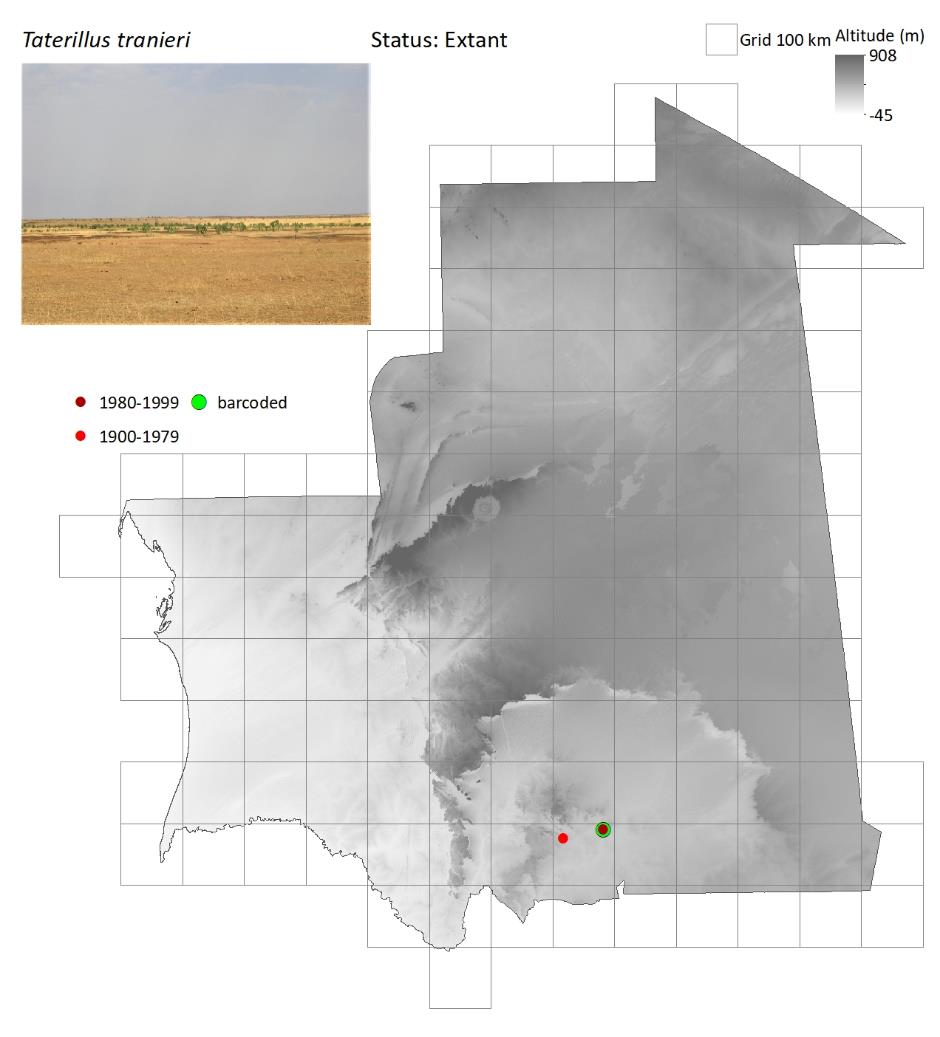


099 - Status and distribution of *Taterillus tranieri* in Mauritania. Picture of the habitat near Tintâne, Province of Hodh El Gharbi (November 2003), where the species has been reported (Granjon et al 2009).

Comparison with IUCN range polygons: No change. Mapped distribution is similar to reported range in Mauritania.

Conservation status: Global IUCN- LC; National final- LC; National original- NT D2.

Comment on the assessment: Downgraded due to the occurrence of neighbouring populations in Mali that may likely provide rescue effect. The species is nearly-endemic to Mauritania.


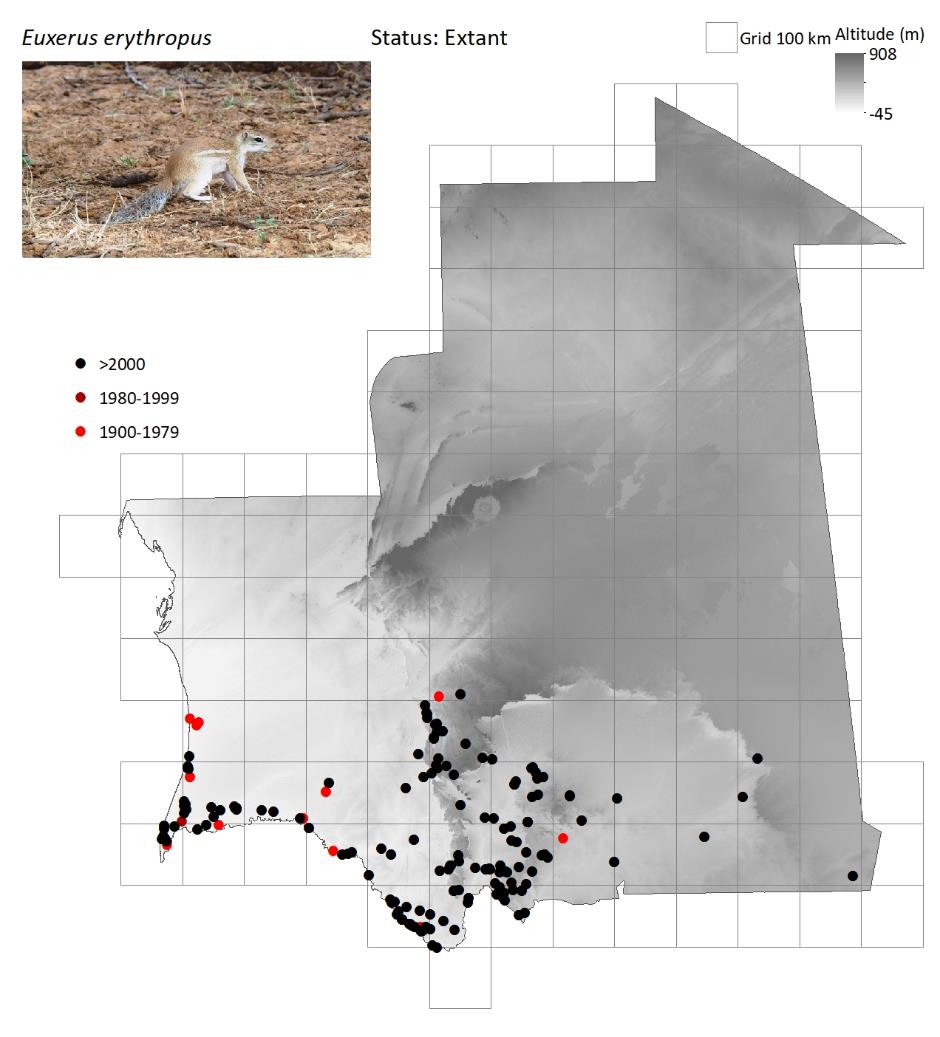


100 - Status and distribution of *Euxerus erythropus* (=*Xerus erythropus*) in Mauritania. Picture near Boumedeit, Province of Tagant (November 2020).

Comparison with IUCN range polygons: No change. Mapped distribution is similar to reported range in Mauritania.

Conservation status: Global IUCN- LC; National final- LC; National original- LC.

101 - Status and distribution of *Crocidura cinderella* in Mauritania. Picture of the habitat about 10 km north of Nouakchott, Province of Trarza (November 2004), where the species has been reported (GBIF.org 2021). Observations identified only up to genus level are represented by triangles.

Comparison with IUCN range polygons: No change. Mapped distribution is similar to reported range in Mauritania.

Conservation status: Global IUCN- LC; National final- DD; National original- DD.

Comment on the assessment: There is only one observation available from before the year 2000.

102 - Status and distribution of *Crocidura fuscomurina* in Mauritania. Picture of the habitat in Chott Boul, Province of Trarza (August 2015), where the species has been reported (Granjon et al 1997, 2002). Observations identified only up to genus level are represented by triangles.

Comparison with IUCN range polygons: Range expansion. The observation in Chott Boul slightly expands northwards the reported range of the species in coastal Mauritania. Still, the observation is from before the year 2000, and additional sampling is needed to understand the current range of the species in Mauritania.

Conservation status: Global IUCN- LC; National final- DD; National original- DD.

Comment on the assessment: There is only one observation available from before the year 2000.

103 - Status and distribution of *Crocidura lusitania* in Mauritania. Picture of the habitat north of Nouakchott, Province of Nouakchott (November 2004), where the species has been reported (Granjon et al 1997). Observations identified only up to genus level are represented by triangles.

Comparison with IUCN range polygons: No change. Mapped distribution is similar to reported range in Mauritania.

Conservation status: Global IUCN- LC; National final- LC; National original- LC.

104 - Status and distribution of *Crocidura nanilla* in Mauritania. Picture of the habitat near Garak, Province of Trarza (November 2010), where the species has been reported (GBIF.org 2021). Observations identified only up to genus level are represented by triangles.

Comparison with IUCN range polygons: No change. Mapped distribution is similar to reported range in Mauritania.

Conservation status: Global IUCN- LC; National final- DD; National original- DD.

Comment on the assessment: There is only one distinct observation available from before the year 2000.

105 - Status and distribution of *Crocidura olivieri* in Mauritania. Picture near Sélibaby, Province of Guidimaka (November 2010). Observations identified only up to genus level are represented by triangles.

Comparison with IUCN range polygons: Range expansion. The observation in Sélibaby expands eastwards the reported range of the species in Mauritania. There is a voucher deposited (MNHN MO-1913-666) with location in “Mauritania”, which is consequently not mapped. Additional sampling is needed to understand the current range of the species.

Conservation status: Global IUCN- LC; National final- DD; National original- DD.

Comment on the assessment: There is only one confirmed observation available.

106 - Status and distribution of *Crocidura viaria* in Mauritania. Picture of the habitat near Bir Moghrein, Province of Tiris Zemmour (April 2017), where the species has been reported (Heim de Balsac 1948; Le Berre 1990). Observations identified only up to genus level are represented by triangles.

Comparison with IUCN range polygons: No change. Mapped distribution is similar to reported range in Mauritania.

Conservation status: Global IUCN- LC; National final- LC; National original- LC.

107 - Status and distribution of *Orycteropus afer* in Mauritania. Picture of the habitat near Sélibaby, Province of Guidimaka (November 2011), where the species has been reported (Dia 2004; Gueye and Dia 2004).

Comparison with IUCN range polygons: No change. Mapped distribution is similar to reported range in Mauritania.

Conservation status: Global IUCN- LC; National final- DD; National original- DD.

Comment on the assessment: There are only four observations available.
