# Supplementary material for: Diversity, distribution and conservation of land mammals in Mauritania, North-West Africa
Source: PLoS One. 2022 Aug 1;17(8):e0269870. doi: 10.1371/journal.pone.0269870 (PMC9342785; doi:10.1371/journal.pone.0269870)

**S19 Figure. Distribution of species richness by mammal Order.** Distribution of species richness of land mammals in Mauritania at 100x100 km UTM scale in the most specious mammal orders: Artiodactyla, Carnivora, Chiroptera, and Rodentia. Grid cells without a single mammal observation are marked (light grey). Mountain plateaus and escarpments (black dashed) and national parks (in italics) are identified.


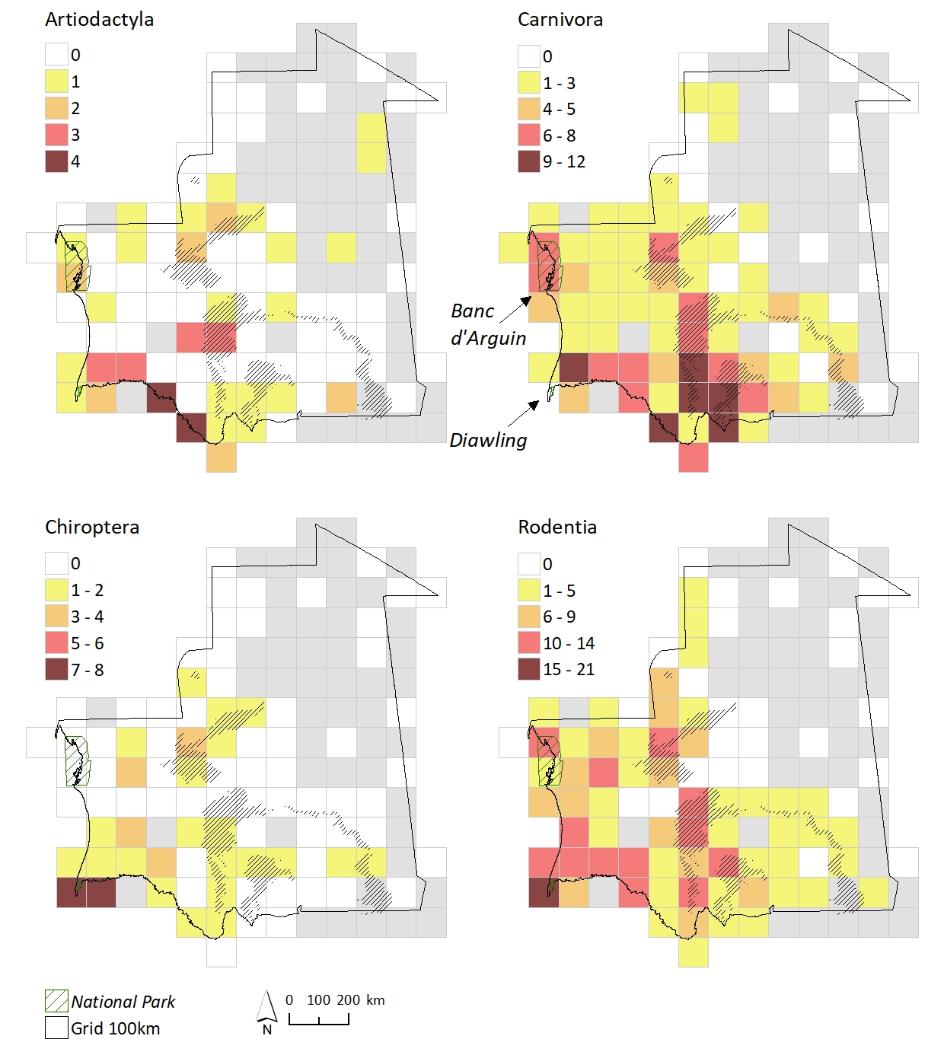

Supplement: S15 Fig — Distribution of species richness of land mammals in Mauritania at 100x100 km UTM scale in the most specious mammal orders: Artiodactyla, Carnivora, Chiroptera, and Rodentia. Grid cells without a single mammal observation are marked (light grey). Mountain plateaus and escarpments (black dashed) and national parks (in italics) are identified. (DOCX) [file pone.0269870.s015.docx]
