# Supplementary material for: Diversity, distribution and conservation of land mammals in Mauritania, North-West Africa
Source: PLoS One. 2022 Aug 1;17(8):e0269870. doi: 10.1371/journal.pone.0269870 (PMC9342785; doi:10.1371/journal.pone.0269870)

**S23 Figure – Time of last observation of non-extant species.** Time of last known observation of Regionally Extinct (RE) and Extinct in the Wild (EW) mammal species in Mauritania. For the EW species with captive individuals (*), the time of release on captivity is marked as +. The observations collected for *Ceratotherium simum* and *Taurotragus derbianus* were strictly from before the year 1900.


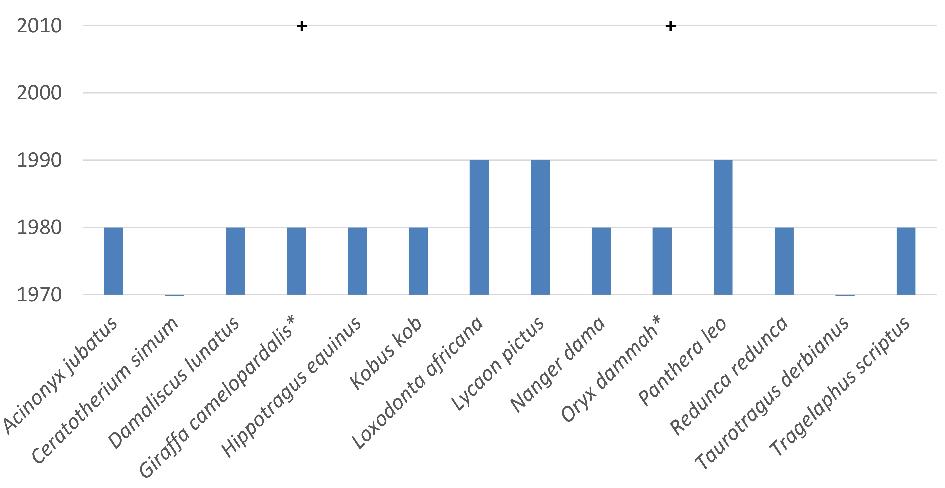

Supplement: S16 Fig — Time of last known observation of Regionally Extinct (RE) and Extinct in the Wild (EW) mammal species in Mauritania. For the EW species with captive individuals (*), the time of release on captivity is marked as +. The observations collected for Ceratotherium simum and Taurotragus derbianus were strictly from before the year 1900. (DOCX) [file pone.0269870.s016.docx]
