# Supplementary material for: Diversity, distribution and conservation of land mammals in Mauritania, North-West Africa
Source: PLoS One. 2022 Aug 1;17(8):e0269870. doi: 10.1371/journal.pone.0269870 (PMC9342785; doi:10.1371/journal.pone.0269870)

**S24 Figure – Temporal change in the distribution of species richness.** Top: Distribution of mammal species richness in Mauritania based on 100x100 km grid cells considering all species assumed to be present in the country by the year 1900. Bottom left: Number of species lost in each grid cell in comparison to the distribution of extant species richness (mapped in Fig. 4). Bottom right: Percentage of species lost in each grid cell. Unsampled grid cells are marked (light grey). Mountain plateaus and escarpments (black dashed) and national parks (green shaded; in italics) are identified.


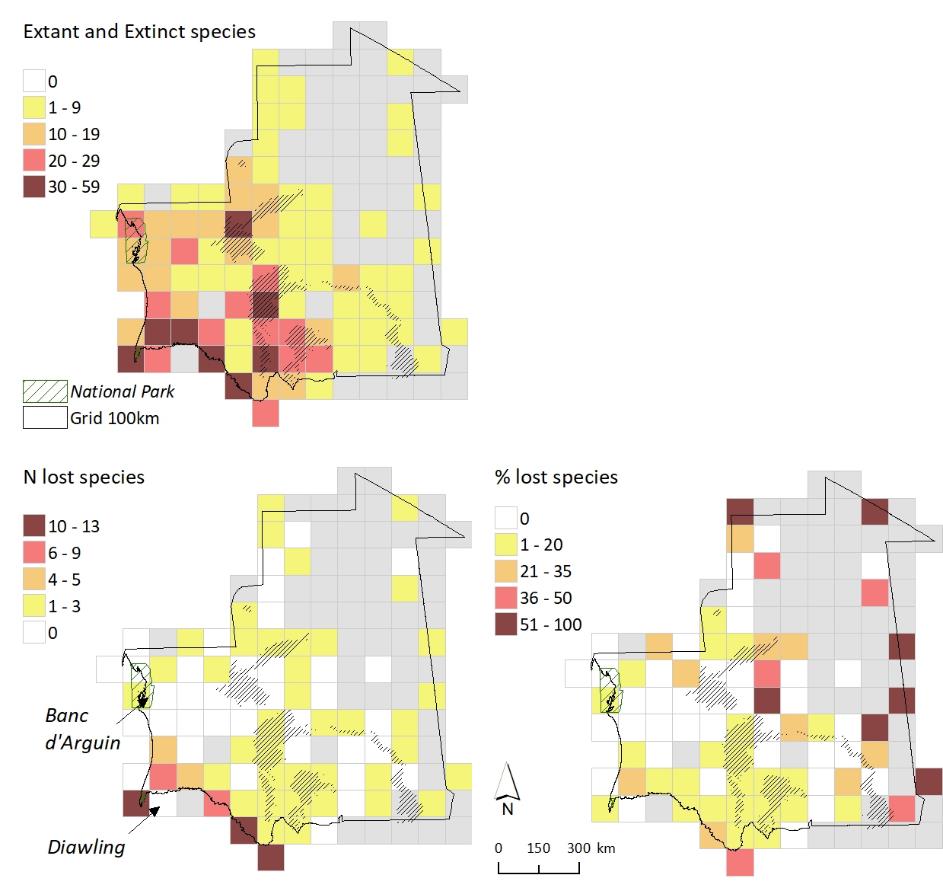

Supplement: S17 Fig — Top: Distribution of mammal species richness in Mauritania based on 100x100 km grid cells considering all species assumed to be present in the country by the year 1900. Bottom left: Number of species lost in each grid cell in comparison to the distribution of extant species richness (mapped in Fig 4). Bottom right: Percentage of species lost in each grid cell. Unsampled grid cells are marked (light grey). Mountain plateaus and escarpments (black dashed) and national parks (green shaded; in italics) are identified. (DOCX) [file pone.0269870.s017.docx]
