# Supplementary material for: Diversity, distribution and conservation of land mammals in Mauritania, North-West Africa
Source: PLoS One. 2022 Aug 1;17(8):e0269870. doi: 10.1371/journal.pone.0269870 (PMC9342785; doi:10.1371/journal.pone.0269870)
Supplement: S18 Fig — Percentage of species listed in each category of extinction risk [1] at the global (GRL) and the national (NRL; Mauritania) levels. The source and destination of the main changes in conservation status are marked by arrows. (DOCX) [file pone.0269870.s018.docx]

**S25 Figure –Global and national conservation status.** Percentage of species listed in each category of extinction risk [1] at the global (GRL) and the national (NRL; Mauritania) levels. The source and destination of the main changes in conservation status are marked by arrows.


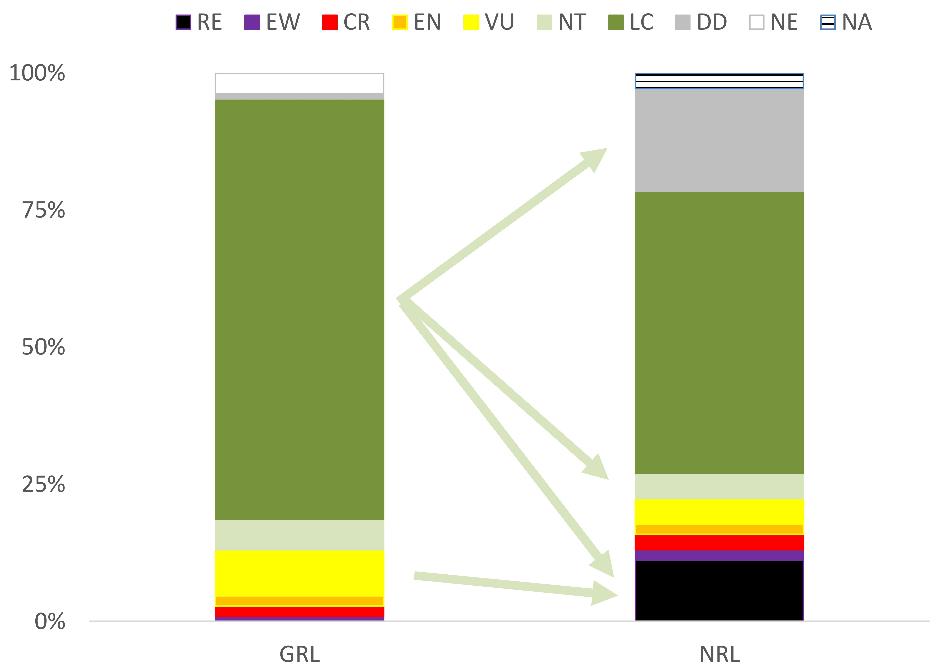


[1] IUCN. The IUCN Red List of Threatened Species. Version 2021-2. 2021. [cited 2021 October 12]. Available from: https://www.iucnredlist.org.
