# Supplementary material for: Diversity, distribution and conservation of land mammals in Mauritania, North-West Africa
Source: PLoS One. 2022 Aug 1;17(8):e0269870. doi: 10.1371/journal.pone.0269870 (PMC9342785; doi:10.1371/journal.pone.0269870)
Supplement: S1 Text — List of bibliographic references from where mammal observations were extracted. (DOCX) [file pone.0269870.s019.docx]

**S11 Text. Bibliographic references.** List of bibliographic references from where mammal observations were extracted.

ACR (2020). *African Chiroptera Report 2020*. V. Van Cakenberghe and E.C.J. Seamark (Eds). AfricanBats NPC, Pretoria.

Allegrini, B., Durand, G., Durand, E., and Peyre, O. (2011). On some bats recorded in the Adrar region, Mauritania. African Bat Conservation News 26: 2-4.

Aulagnier, S., Cuzin, F., and Thevenot, M. (2017). Mammifères Sauvages du Maroc. Peuplement, Répartition, Écologie. Société Française pour l'Étude et la Protection des Mammifères, Paris.

Bâ, K., Mathiot, C., Diallo, M., Nabeth, P., Lochouarn, L., Kâne, Y., Abdalahi, M.O., and Granjon, L. (2001). Preliminary study on some rodents of southern Mauritania as reservoir of human pathogenic viruses. In: Denys, C., Granjon, L., and Poulet, A. (Eds.). *African small mammals*. IRD, Paris, pp. 101-107.

Ba, A., Fall, O., and Hamerlynck, O. (2002). Le Parc National du Diawling: expérience de co-gestion pour la restauration des plaines inondables. In: Gawler, M. (Ed.). *Strategies for Wise Use of Wetlands: Best Practices in Participatory Management*. Wetlands International IUCN, WWF Publication No. 56, Wageningen, The Netherlands, pp. 19-25.

Beudels-Jamar, R. C., Devillers, P., Lafontaine, R.-M., Devillers-Terschuren, J., and Beudels, M.-O. (2006). *Les Antilopes Sahélo-Sahariennes. Statut et Perspectives*. Rapport sur l'état de conservation des six Antilopes Sahélo-Sahariennes. CMS Technical Series Publication N° 11. UNEP/CMS Secretariat, Bonn.

Boratyński, Z., Brito, J.C., and Mappes, T. (2012). The origin of two cryptic species of African desert jerboas (Dipodidae: *Jaculus*). Biological Journal of the Linnean Society 105: 435-445.

Boratyński, Z., Campos, J.C., Gonçalves, D.V., Granjon, L., Martínez-Freiría, F., Sow, A.S., Velo-Antón, G., and Brito, J.C. (2013). The Sudano-Sahelian Dalton's Mouse, *Praomys daltoni*, in Mauritania, Eastern Assaba mountains. Go-South Bulletin 10: 17-20.

Boratyński, Z., Brito, J.C., Campos, J.C., Karala, M., Koskela, E., and Mappes, T. (2014). Large spatial scale of the phenotype-environment color matching in two cryptic species of African desert jerboas (Dipodidae: *Jaculus*). PLoS One 9: e94342.

Boratyński, Z., Brito, J.C., Campos, J.C., Cunha, J.L., Granjon, L., Mappes, T., Ndiaye, A., Rzebik-Kowalska, B., and Serén, N. (2017). Repeated evolution of camouflage in speciose desert rodents. Scientific Reports 7: 3522.

Brito, J.C., Acosta, A.L., Álvares, F., and Cuzin, F. (2009). Biogeography and conservation of taxa from remote regions: An application of ecological-niche based models and GIS to North-African canids. Biological Conservation 142: 3020-3029.

Brito, J.C., Álvares, F., Martínez-Freiría, F., Sierra, P., Sillero, N., and Tarroso, P. (2010). Data on the distribution of mammals from Mauritania, West Africa. Mammalia 74: 449-455.

Campbell, A., Coulson, D., Challis, S., and Keenan, J. (2006). Some Mauritanian rock art sites. Sahara 17: 143-148.

Chevret, P. and Dobigny G. (2005). Systematics and evolution of the subfamily Gerbillinae (Mammalia, Rodentia, Muridae). Molecular Phylogenetics and Evolution 35: 674–688.

Chudeau, R. (1920). Remarques sur quelques mammifères du Sahara et du nord du Soudan. Association française pour l'Avancement des Sciences 44: 307-312.

Ciofolo, I. (1995) West Africa's last giraffes: the conflict between development and conservation. Journal of Tropical Ecology 11: 577-588.

Convention on Migratory Species (1998). *Conservation Measures for Sahelo-Saharan Antelopes. Action Plan and Status Reports*. CMS Technical Series Publication No. 4, Bonn.

Cosson, J.‐F., Tranier, M., and Colas, F. (1996). On the occurrence and possible migratory behaviour of the fruit bat *Eidolon helvum* in Mauritania, Africa. Journal of African Zoology 110: 369-371.

Dekeyser, P.L. and Villiers, A. (1956). Contribution à l'étude du peuplement de la Mauretanie. Notations écologiques et biogeographiques sur la faune de I'Adrar. Mémoires de l'Institut Français de l'Afrique Noire 44: 1-222.

Denys, C., Granjon, L., and Poulet, A. (2001). *African Small Mammals*. IRD Éditions, Paris.

Dia, A.T. (2004). *Inventaire de la biodiversité des sites de l'UNP/MAU: Rapport provisoire*. Projet RAF/98/G31. Conservation de la Diversité Biologique par la Réhabilitation Participative des Sols Dégradés dans les Zones Arides et Semi-Arides Transfrontalières de la Mauritanie et du Sénégal, Nouakchott.

Diarra, M. (1994). Information Sheet on Ramsar Wetlands. Parc National du Diawling. Wetlands International. http://www.wetlands.org/reports/ris/1MR002en.pdf.

Diatta, G., Duplantier, J.-M., Granjon, L., Bâ, K., Chauvancy, G., Ndiayed, M., and Trape, J.-F. (2015). Borrelia infection in small mammals in West Africa and its relationship with tick occurrence inside burrows. Acta Tropica 152: 131-140.

Dobigny, G., Aniskin, V., Granjon, L., Cornette, R., and Volobouev, V. (2005). Recent radiation in West African *Taterillus* (Rodentia, Gerbillinae): the concerted role of chromosome and climatic changes. Heredity 95: 358-368.

Dobigny, G., Tatard, C., Gauthier, P., Bâ, K., Duplantier, J.-P., Granjon, L., and Kergoat, G.J. (2013). Mitochondrial and nuclear genes-based phylogeography of *Arvicanthis niloticus* (Murinae) and Sub-Saharan open habitats Pleistocene history. PLoS ONE 8: e77815.

Duchemin, G. (1949). Les elephants de Mauritanie. Notes Africaines 44: 127-129.

East, R. (1999). *African Antelope Database 1998*. IUCN, Gland, Switzerland and Cambridge, UK.

Faleh, A.B., Granjon, L., Tatard, C., Boratyński, Z., Cosson, J.-F., and Said, K. (2012). Phylogeography of two cryptic species of African desert jerboas (Dipodidae: *Jaculus*). Biological Journal of the Linnean Society 107: 27-38.

Favotti, J. (1960). Découverte de peintures rupestres anciennes a Tenses (Adrar Mauritanien). Notes Africaines 88: 103-106.

Flanagan, D.E. (2006). I. Comparação de metodologias para estimar a abundância de carnívoros em meio desértico. II. Distribuição e ritmos de actividade do chacal dourado e do feneco no Parc National du Banc d'Arguin, Mauritânia. Internship Report. Faculdade de Ciências da Universidade de Lisboa, Portugal.

Garbit, L. (1935). Note géographique sur le Massif de la Kédiat Idjil. Bulletin du Comité d'Études Historiques et Scientifiques de l'Afrique Occidentale Française 28: 393-402.

GBIF.org (2021). GBIF Occurrence Download. [cited 2021 November 05]. Available from: https://doi.org/10.15468/dl.5n5czb

Gowthorpe, P. (1993). Une visite au Parc National du Banc d'Arguin: itinéraires et présentation des principales composantes naturelles. Parc National du Banc d'Arguin, Nouakchott.

Grandidier, G. (1932). Les éléphants de Mauritanie. La Terre et la Vie 3: 130-134.

Granjon, L., Cosson, J.-F., Cuisin, J., Tranier, M., and Colas, F. (1997). Les mammifères du littoral mauritanien. 2. Biogéographie et écologie. In: Colas, F. (Ed.). *Environnement et Littoral Mauritanien: actes du colloque, 12-13 juin 1995, Nouakchott, Mauritanie*. CIRAD, CNERV, ISS, CIRAD-EMVT, Montpellier, pp. 73-81.

Granjon, L., Bruderer, C., Cosson, J.-F., Dia, A.T., and Colas, F. (2002). The small mammal community of a coastal site of south-west Mauritania. African Journal of Ecology 40: 10-17.

Granjon, L. and Duplantier, J.M. (2009). *Les rongeurs de l'Afrique sahélo-soudanienne*. IRD Éditions, Marseille.

Gueye, S. and Dia, A.T. (2004). *Inventaire de la biodiversité des sites des UNP/MAU et UNP/SEN. Document de synthese*. Projet RAF/98/G31. Conservation de la Diversité Biologique par la Réhabilitation Participative des Sols Dégradés dans les Zones Arides et Semi-Arides Transfrontalières de la Mauritanie et du Sénégal. Nouakchott.

Heim de Balsac, H. (1948). Etat actuel de nos connaissances concernant la faune des mammifères du Maroc. Mémoires de la Société des Sciences Naturelles du Maroc 1920-1945: 289-303.

Holl, A.F.C. (1985). Subsistence patterns of the Dhar Tichitt Neolithic, Mauritania. The African Archaeological Review 3: 151-162.

Holl, A.F.C. (2002). Time, space, and image making: rock art from the Dhar Tichitt (Mauritania). The African Archaeological Review 19: 75-118.

Holl, A.F.C. (2009). Coping with uncertainty: Neolithic life in the Dhar Tichitt-Walata, Mauritania, (ca. 4000–2300 BP). Comptes Rendus Geoscience 341: 703-712.

Howard-Mccombe, J., Banfield, L., Kitchener, A.C., Al Qahtani, H., Toosy, A., Qarqas, M., Craig, M., Abramov, A., Veron, G., Brito, J.C., Azizi, S., Breton, B., Silwa, A., Witzenberger, K., Hochkirch, A., and Senn, H. (2019). A mitochondrial phylogeny of the sand cat (*Felis margarita* Loche, 1858). Journal of Mammalian Evolution 27: 525-534.

Hughes, R.H., Hughes, J.S., and Bernacsek, G. (1992). Mauritania. In: Bernacsek, G.M., Hughes, J.S., Hughes, R.H. (Eds.). *A Directory of African Wetlands*. IUCN, UNEP, WCMC, Gland.

Jacobson, A.P., Gerngross, P., Lemeris Jr., J.R., Schoonover, R.F., Anco, C., Breitenmoser-Wuersten, C., Durant, S.M., Farhadinia, M.S., Henschel, P., Kamler, J.F., Laguardia, A., Rostro-García, S., Stein, A.B., and Dollar, L. (2016). Leopard (Panthera pardus) status, distribution, and the research efforts across its range. PeerJ 4: e1974.

Jullien, R. and Petter, F. (1970). La faune du gisement d'Akjoujt (Mauritanie). Bulletin du Muséum National d'Histoire Naturelle, Paris 41: 1290-1291.

Kirsch-Jung, K.P. and Khtour, D.O. (2007). *Conservation et utilisation des zones humides dans le Hodh El Gharbi mauritanien*. République Islamique de Mauritanie, Secrétariat d'Etat auprès du Premier Ministre chargé de l'Environnement and Coopération Technique Allemande (GTZ), Nouakchott. Available at: <http://www2.gtz.de/dokumente/bib/07-0608.pdf>.

Klein, J.M., Poulet, A.R., and Simonkovich, E. (1975). Observations écologiques dans une zone enzootique de peste en Mauritanie 1. Les rongeurs, et en particulier *Gerbilus gerbillus* Olivier, 1801 (Rodentia, Gerbillinae). Cahiers ORSTOM, série Entomologie Médicale et Parasitologie 13: 13-28.

Koepfli, K.-P., Pollinger, J., Godinho, R., Robinson, J., Lea, A., Hendricks, S., Schweizer, R.M., Thalmann, O., Silva, P., Fan, Z., Yurchenko, A.A., Dobrynin, P., Makunin, A., Cahill, J.A., Shapiro, B., Álvares, F., Brito, J.C., Geffen, E., Leonard, J.A., Helgen, K.M., Johnson, W.E., O’Brien, S.J., Van Valkenburgh, B., and Wayne, R.K. (2015). Genome-wide evidence reveals that African and Eurasian Golden Jackals are distinct species. Current Biology 25: 2158-2165.

Kopp, G.H., Ferreira da Silva, M.J., Fischer, J., Brito, J.C., Regnaut, S., Roos, C., and Zinner, D. (2014). The influence of social systems on patterns of mitochondrial DNA variation in Baboons. International Journal of Primatology 35: 210-225.

Lado, S., Alves, P.C., Islam, M.Z., Brito, J.C., and Melo-Ferreira, J. (2019). The evolutionary history of the Cape hare (*Lepus capensis* sensu lato): insights for systematics and biogeography. Heredity 123: 634-646.

Lafont, P. and Measson, L. (2001). Compte rendu d'un voyage dans le delta du fleuve Senegal. Partie II - Suite et fin: le sud mauritanien. Available at: <http://www.lpo.fr/voyages/doc/MauritanieSud2001Louvel.pdf>.

Lamarche, B. (1980). L'addax *Addax nasomaculatus* (Blainville): I. Biologie. Report to WWF-IUCN, Gand.

Lamarche, B. and Hamerlink, O. (1998). Les ongulés sahélo-sahariens du Mali et de la Mauritanie: statut et répartition, passée et présent. In: UNEP/CMS (ed.). *Proceedings of the Seminar on the Conservation and Antelopes*. CMS Technical Series Publication No. 3. UNEP/CMS, Bonn.

Le Berre, M. (1990). *Faune du Sahara 2 - Mammiferes*. LeChevalier - R. Chabaud.

Leite, J.V., Álvares, F., Velo-Antón, G., Brito, J.C., and Godinho, R. (2015). Differentiation of North African foxes and population genetic dynamics in the desert - insights into the evolutionary history of two sister taxa, *Vulpes rueppellii* and *V. vulpes*. Organisms Diversity and Evolution 15: 731-745.

Ly, O.K. and Zein, S.A.O.M. (2009). Évaluation économique d'une zone humide: le cas du Diawling, Mauritanie. IUCN, Gland, Suisse.

Mahé, E. (1985). Contribution à l'étude scientifique de la région du Banc d'Arguin. Univ. Sciences et Techniques du Languedoc, Montpellier.

Mauritanie 2000 SARL (2001). *Projet Guidimaka: Recuile Socio-Economique*. Rapport final. MDRE/GTZ/ARGE ECO-IRAM, Nouakchott.

Messerli, B. and Winiger, M. (1992). Climate, environmental change, and resources of the African Mountains from the Mediterranean to the Equator. Mountain Research and Development 12: 315-336.

Mikula, O., Nicolas, V., Boratyński, Z., Denys, C., Dobigny, G., Fichet-Calvet, E., Gagaré, S., Hutterer, R., Nimo-Paintsil, S.C., Olayemi, A., Bryja, J. (2020). Commensalism outweighs phylogeographical structure in its effect on phenotype of a Sudanian savanna rodent. Biological Journal of the Linnean Society, 129:931-949.

Mills, M.G.L. and Hofer, H. (1998). *Hyaenas. Status, Survey and Conservation Action Plan*. IUCN, Gland, Switzerland and Cambridge, UK.

Monod, T. (1961). Majâbat al-Koubrâ (supplément). Bulletin de l'Institut Fondamental d'Afrique Noire 23: 591-637.

Monod, T. (1964). Majabât al-Koubrâ (2e supplément). Bulletin de l'Institut Fondamental d'Afrique Noire 26: 1393-1402.

Monod, T. (1968). Rapport sur une mission effectuée dans l'Adrar de Mauritanie (21 Novembre au 17 Décembre 1967). Bulletin de l'Institut Fondamental d'Afrique Noire 30: 1737-1747.

Moreno, E. (2009). Restauración de ungulados Sahlo-Saharianos en la Meseta de Tagant.Conservación y Desarrollo en la Meseta de Tagant (Mauritania). Universidad Complutense de Madrid. Available at: <http://www.tagant.org>.

Mouline, K., Granjon, L., Galan, M., Tatard, C., Abdoullaye, D., Atteyene, S.A., Duplantier, J.-P., and Cosson,J.-F. (2008). Phylogeography of a Sahelian rodent species *Mastomys huberti*: a Plio-Pleistocene story of emergence and colonization of humid habitats. Molecular Ecology 17: 1036-1053.

Moutinho, A.F., Serén, N., Paupério, J., Silva, T.L., Martínez-Freiría, F., Sotelo, G., Faria, R., Mappes, T., Alves, P.C., Brito, J.C., and Boratyński, Z. (2020). Evolutionary history of two cryptic species of northern African jerboas. BMC Evolutionary Biology 20: 26.

Munier, P. (1952). L'Assaba. Essai Monographique. Études Mauritaniennes 3: 1-71.

National Research Council (1981). *Environmental Degradation in Mauritania*. National Academy Press, Washington.

Ndiaye, A., Bâ, K., Aniskin, V., Benazzou, T., Chevret, P., Konecný, A., Sembène, M., Tatard, C., Kergoat, G.J., and Granjon, L. (2012). Evolutionary systematics and biogeography of endemic gerbils (Rodentia, Muridae) from Morocco: an integrative approach. Zoologica Scripta 41: 11-28.

Ndiaye, A., Shanas, U., Chevret, P., and Granjon, L. (2013). Molecular variation and chromosomal stability within *Gerbillus nanus* (Rodentia, Gerbillinae): taxonomic and biogeographic implications. Mammalia 77: 105-111.

Ndiaye, A., Hima, K., Dobigny, G., Sow, A., Dalecky, A., Bâ, K., Thiam, M., and Granjon, L. (2014). Integrative taxonomy of a poorly known Sahelian rodent, *Gerbillus nancillus* (Muridae, Gerbillinae). Zoologischer Anzeiger 253: 430-439.

Ndiaye, A., Chevret, P., Dobigny, G., and Granjon, L. (2016). Evolutionary systematics and biogeography of the arid habitat-adapted rodent genus *Gerbillus* (Rodentia, Muridae): a mostly Plio-Pleistocene African history. Journal of Zoological Systematics and Evolutionary Research 54: 299-317.

Newby, J.E. (1984). Large mammals. In: Cloudsley-Thompson, J.L. (Ed.). *Key Environments: Sahara Desert*. Pergamon Press, Oxford, pp. 277-290.

Nickel, H. (2003). *Ökologische untersuchungen zur wirbeltierfauna im südöstlichen Mauretanien*. Zwei fallstudien unter berücksichtigung der Krokodile. Deutsche Gesellschaft für Technische Zusammenarbeit, Eschborn.

Nicolas, V., Granjon, L., Duplantier, J.-P., Cruaud, C., and Dobigny, G. (2009). Phylogeography of spiny mice (genus *Acomys*, Rodentia: Muridae) from the south-western margin of the Sahara with taxonomic implications. Biological Journal of the Linnean Society 98: 29-46.

Padial, J.M. and Ibáñez, C. (2005). New records and comments for the Mauritanian mammal fauna. Mammalia 69: 239-244.

Padial, J.M. and Tellería, J.L. (2009). Observations of the *Felou gundi* (Felovia vae Lataste 1886) in central Mauritania (Rodentia: Ctenodactylidae). Mammalia 73: 153-154.

Padial, J.M., Castroviejo-Fischer, S., Quintana, A.Z., Ávilla, E., Pérez-Marín, J., and Castroviejo, J. (2002). Notas de distribución de reptiles para el NE de África. Boletín de la Asociación Herpetológica Española 13: 2-5.

Peled, E., Shanas, U., Granjon, L., and Ben-Shlomo, R. (2016). Connectivity in fragmented landscape: generalist and specialist gerbils show unexpected gene flow patterns. Journal of Arid Environments 125: 88-97.

Poulet, A.R. (1970). Les Rhinopomatidae de Mauritanie. Mammalia 34: 237-243.

Poulet, A.R. (1974). Rongeurs et insectivores dans des pelotes d'Effraie en Mauritanie. Mammalia 38: 145-146.

Qumseyeh, M.B. and Schütter, D.A. (1981). Bat records from Mauritania, Africa (Mammalia: Chiroptera). Annals of Carnegie Museum 50: 345-351.

Ribas, A., Diagne, C., Tatard, C., Diallo, M., Poonlaphdecha, S., and Brouat, C. (2017). Whipworm diversity in West African rodents: a molecular approach and the description of *Trichuris duplantieri* n. sp. (Nematoda: Trichuridae). Parasitology Research 116: 1265-1271.

Senones, M. and Puigaudeau, O.D. (1939). Peintures rupestres du Tagant (Mauritanie). Journal de la Société des Africanistes 9: 43-70.

Sevenet, L. (1943). Étude sur le "Djouf" (Sahara Occidental). Bulletin de l'Institut Fondamental d'Afrique Noire 5: 1-26.

Sheppard, T. (1975). The joint services West East Sahara expedition. The Geographical Journal 142: 201-215.

Silva, T.L., Godinho, R., Castro, D., Abáigar, T., Brito, J.C., and Alves, P.C. (2015). Genetic identification of endangered North African ungulates using noninvasive sampling. Molecular Ecology Resources 15: 652-661.

Simões, M.L. (2001). O chacal-dourado (Canis aureus Linnaeus, 1758) no Parc National du Banc d'Arguin, Mauritânia. Internship Report. Faculdade de Ciências da Universidade de Lisboa, Portugal.

Spatz, P. (1930). Reisen aus den jahren 1928 und 1929 nach dem Senegal und Mauretanien. Zeitschrift für Säugetierkunde 5: 13.

Tellería, J.L. (2009). Biodiversidad y conservación en la Meseta de Tagant. Universidad Complutense de Madrid. Available at: <http:www.tagant.org>.

Thiam, A.B. (2020). Parc National d'Awleigatt. Manuel de la faune herbivore et de la flore ligneuse du Parc National de l'Awleigatt. Report. 23pp. Ministère de l'Environnement et du Developpement Durable, Nouakchott, République Islamique de la Mauritanie.

Thomassey, P. (1951). Notes sur la géographie et l'habitat de la région de Koumbi Saleh. Bulletin de l'Institut Fondamental d'Afrique Noire 13: 476-486.

Trotignon, J. (1975). Le status et la conservation de l'addax et de l'oryx et de la faune associée en Mauritanie. Report to IUCN, Morges.

Vale, C.G., Álvares, F., and Brito, J.C. (2012). Distribution, suitable areas and conservation status of the *Felou gundi* (Felovia vae Lataste 1886). Mammalia 76: 201-207.

Vale, C.G., Tarroso, P., and Brito, J.C. (2014). Predicting species distribution at range margins: testing the effects of study area extent and resolution, and threshold selection in the Sahara-Sahel transition zone. Diversity and Distributions 20: 20-33.

Vale, C.G., Pimm, S.L., and Brito, J.C. (2015). Overlooked mountain rock pools in deserts are critical local hotspots of biodiversity. PLoS One 10: e0118367.

Vale, C.G., Ferreira da Silva, M.J., Campos, J.C., Torres, J., and Brito, J.C. (2015). Applying species distribution modelling to the conservation of an ecologically plastic species (*Papio papio*) across biogeographic regions in West Africa. Journal for Nature Conservation 27: 26-36.

Vale, C.G., Campos, J.C., Silva, T.L., Gonçalves, D.V., Sow, A.S., Martínez-Freiría, F., Boratyński, Z., and Brito, J.C. (2016). Biogeography and conservation of mammals from the West Sahara-Sahel: an application of ecological niche-based models and GIS. Hystrix-Italian Journal of Mammology, doi:10.4404/hystrix-27.1-11659.

Valverde, J.A. (1957). *Aves del Sahara Español* (Estudio Ecológico del Desierto). Instituto de Estudios Africanos, CSIC, Madrid.

Velo-Antón, G., Boratyński, Z., Ferreira, C.M., Lima, V.O., Alves, P.C., and Brito, J.C. (2019). Intra-specific genetic diversity and distribution of North African hedgehogs (Mammalia: Erinaceidae). Biological Journal of the Linnean Society 127: 156-163.

Vernet, R. (2008). *Addax nasomaculatus*. [cited 2009 January 12]. Available from: http://www.geres-asso.org/Nouvelles_observations.html#MAMMIFERES

Verschuren, J. (1984). République Islamique de Mauritanie. Parc National du Banc d’Arguin. Plan directeur préliminaire. UICN/WWF, Gland.
