# Supplementary material for: Diversity, distribution and conservation of land mammals in Mauritania, North-West Africa
Source: PLoS One. 2022 Aug 1;17(8):e0269870. doi: 10.1371/journal.pone.0269870 (PMC9342785; doi:10.1371/journal.pone.0269870)
Supplement: S2 Text — Dataset of observations of land mammals in Mauritania (excluding observations available at GBIF) including the code of the observation (Code), Order, Family and Species name (Species), the Latitude and Longitude in decimal degrees (WGS84 projection), the code of the UTM 100 km grid cell size of the observation (UTM100), and when available the reference to molecular or observational data (Ref). Codes of UTM 100 km cells are available in S12 Fig. References are available in S1 Text. (DOCX) [file pone.0269870.s020.docx]

**S15 Text. Dataset of observations.** Dataset of observations of land mammals in Mauritania (excluding observations available at GBIF) including the code of the observation (Code), Order, Family and Species name (Species), the Latitude and Longitude in decimal degrees (WGS84 projection), the code of the UTM 100 km grid cell size of the observation (UTM100), and when available the reference to molecular or observational data (Ref). Codes of UTM 100 km cells are available in S13 Figure. References are available in S11 Text.

| Code | Order | Family | Species | Latitude | Longitude | UTM100 | Ref |
| --- | --- | --- | --- | --- | --- | --- | --- |
| BIB_0011 | Artiodactyla | Bovidae | Addax nasomaculatus | 20.783330 | -9.790647 | H9 | Vernet 2008 |
| BIB_0010 | Artiodactyla | Bovidae | Addax nasomaculatus | 21.880000 | -10.650000 | G8 | Monod 1968 |
| BIB_0009 | Artiodactyla | Bovidae | Addax nasomaculatus | 21.000000 | -9.500000 | G9 | Monod 1964 |
| BIB_0008 | Artiodactyla | Bovidae | Addax nasomaculatus | 25.800000 | -6.700000 | B12 | Beudels-Jamar et al 2006 |
| BIB_0007 | Artiodactyla | Bovidae | Addax nasomaculatus | 23.170000 | -6.580000 | E12 | Beudels-Jamar et al 2006 |
| BIB_0006 | Artiodactyla | Bovidae | Addax nasomaculatus | 20.000000 | -6.500000 | I13 | Sheppard 1975; Convention on Migratory Species 1998; East 1999 |
| BIB_0005 | Artiodactyla | Bovidae | Addax nasomaculatus | 18.333333 | -9.083333 | J10 | Holl 1985 |
| BIB_0004 | Artiodactyla | Bovidae | Addax nasomaculatus | 18.450000 | -9.500000 | J9 | Holl 2009 |
| BIB_0003 | Artiodactyla | Bovidae | Addax nasomaculatus | 25.220000 | -11.577000 | C7 | Le Berre 1990 |
| BIB_0002 | Artiodactyla | Bovidae | Addax nasomaculatus | 18.370000 | -6.960000 | J12 | Monod 1961 |
| BIB_0001 | Artiodactyla | Bovidae | Addax nasomaculatus | 21.000000 | -6.500000 | G13 | Sevenet 1943 |
| 15882 | Artiodactyla | Bovidae | Addax nasomaculatus | 20.906198 | -10.642113 | H8 |  |
| 15873 | Artiodactyla | Bovidae | Addax nasomaculatus | 20.679500 | -10.441688 | H8 |  |
| 13756 | Artiodactyla | Bovidae | Addax nasomaculatus | 23.800140 | -10.632000 | D8 |  |
| 9745 | Artiodactyla | Bovidae | Addax nasomaculatus | 18.390480 | -8.554273 | J10 |  |
| 5897 | Artiodactyla | Bovidae | Addax nasomaculatus | 21.500223 | -11.619505 | G7 |  |
| 5896 | Artiodactyla | Bovidae | Addax nasomaculatus | 21.500223 | -11.619505 | G7 |  |
| 5895 | Artiodactyla | Bovidae | Addax nasomaculatus | 21.500223 | -11.619505 | G7 |  |
| 3565 | Artiodactyla | Bovidae | Addax nasomaculatus | 21.493688 | -11.331722 | G7 |  |
| 2889 | Artiodactyla | Bovidae | Addax nasomaculatus | 21.500223 | -11.619505 | G7 |  |
| BIB_0014 | Artiodactyla | Bovidae | Ammotragus lervia | 22.650000 | -12.540000 | F6 | Garbit 1935 |
| BIB_0013 | Artiodactyla | Bovidae | Ammotragus lervia | 19.751600 | -14.428100 | I4 | Jullien and Petter 1970 |
| BIB_0012 | Artiodactyla | Bovidae | Ammotragus lervia | 17.888000 | -15.509000 | K3 | Thiam 2020 |
| 13692 | Artiodactyla | Bovidae | Ammotragus lervia | 20.608300 | -12.688700 | H6 |  |
| 5890 | Artiodactyla | Bovidae | Ammotragus lervia | 21.500223 | -11.619505 | G7 |  |
| 5887 | Artiodactyla | Bovidae | Ammotragus lervia | 21.500223 | -11.619505 | G7 |  |
| 3574 | Artiodactyla | Bovidae | Ammotragus lervia | 21.430742 | -11.302216 | G7 |  |
| 2891 | Artiodactyla | Bovidae | Ammotragus lervia | 21.459242 | -11.600289 | G7 |  |
| BIB_0017 | Artiodactyla | Bovidae | Damaliscus lunatus | 14.775834 | -12.213947 | O7 | Mauritanie 2000 SARL 2001 |
| BIB_0016 | Artiodactyla | Bovidae | Damaliscus lunatus | 16.710000 | -16.120000 | L3 | Gueye and Dia 2004 |
| BIB_0015 | Artiodactyla | Bovidae | Damaliscus lunatus | 15.300000 | -12.500000 | N6 | Dia 2004; Gueye and Dia 2004 |
| BIB_0036 | Artiodactyla | Bovidae | Eudorcas rufifrons | 16.260000 | -13.940000 | M5 | Dia 2004 |
| BIB_0035 | Artiodactyla | Bovidae | Eudorcas rufifrons | 18.270000 | -12.330000 | K6 | Moreno 2009 |
| BIB_0034 | Artiodactyla | Bovidae | Eudorcas rufifrons | 14.775834 | -12.213947 | O7 | Mauritanie 2000 SARL 2001 |
| BIB_0033 | Artiodactyla | Bovidae | Eudorcas rufifrons | 15.470000 | -12.950000 | N6 | Dia 2004 |
| BIB_0032 | Artiodactyla | Bovidae | Eudorcas rufifrons | 16.710000 | -16.120000 | L3 | Gueye and Dia 2004 |
| BIB_0031 | Artiodactyla | Bovidae | Eudorcas rufifrons | 16.620000 | -14.290000 | L4 | East 1999 |
| BIB_0030 | Artiodactyla | Bovidae | Eudorcas rufifrons | 16.300000 | -10.250000 | M9 | East 1999 |
| BIB_0029 | Artiodactyla | Bovidae | Eudorcas rufifrons | 15.767000 | -7.975000 | M11 | East 1999 |
| BIB_0028 | Artiodactyla | Bovidae | Eudorcas rufifrons | 15.500000 | -12.956000 | N6 | East 1999 |
| BIB_0027 | Artiodactyla | Bovidae | Eudorcas rufifrons | 14.890000 | -12.420000 | N6 | East 1999 |
| BIB_0026 | Artiodactyla | Bovidae | Eudorcas rufifrons | 16.780000 | -14.920000 | L4 | Dia 2004 |
| BIB_0025 | Artiodactyla | Bovidae | Eudorcas rufifrons | 15.767000 | -7.975000 | M11 | Thomassey 1951 |
| BIB_0024 | Artiodactyla | Bovidae | Eudorcas rufifrons | 17.950000 | -12.250000 | K7 | Tellería 2009 |
| BIB_0023 | Artiodactyla | Bovidae | Eudorcas rufifrons | 16.830000 | -15.700000 | L3 | Dia 2004; Gueye and Dia 2004 |
| BIB_0022 | Artiodactyla | Bovidae | Eudorcas rufifrons | 16.120000 | -13.690000 | M5 | Dia 2004 |
| BIB_0021 | Artiodactyla | Bovidae | Eudorcas rufifrons | 16.400000 | -16.400000 | M2 | Spatz 1930 |
| BIB_0020 | Artiodactyla | Bovidae | Eudorcas rufifrons | 16.303340 | -16.401357 | M2 | National Research Council 1981; East 1999; Ba et al 2002 |
| BIB_0019 | Artiodactyla | Bovidae | Eudorcas rufifrons | 16.510000 | -14.190000 | M5 | Dia 2004 |
| BIB_0018 | Artiodactyla | Bovidae | Eudorcas rufifrons | 15.300000 | -12.500000 | N6 | Gueye and Dia 2004 |
| 18751 | Artiodactyla | Bovidae | Eudorcas rufifrons | 15.288200 | -10.812240 | N8 |  |
| 15324 | Artiodactyla | Bovidae | Eudorcas rufifrons | 16.993167 | -15.458500 | L3 | Silva et al 2015 |
| 12463 | Artiodactyla | Bovidae | Eudorcas rufifrons | 15.845405 | -11.205873 | M8 |  |
| BIB_0059 | Artiodactyla | Bovidae | Gazella dorcas | 16.260000 | -13.940000 | M5 | Dia 2004; Gueye and Dia 2004 |
| BIB_0058 | Artiodactyla | Bovidae | Gazella dorcas | 18.270000 | -12.330000 | K6 | Moreno 2009 |
| BIB_0057 | Artiodactyla | Bovidae | Gazella dorcas | 16.303340 | -16.401357 | M2 | National Research Council 1981 |
| BIB_0056 | Artiodactyla | Bovidae | Gazella dorcas | 16.710000 | -16.120000 | L3 | Gueye and Dia 2004 |
| BIB_0055 | Artiodactyla | Bovidae | Gazella dorcas | 22.100000 | -11.850000 | F7 | Lamarche and Hamerlink 1998 |
| BIB_0054 | Artiodactyla | Bovidae | Gazella dorcas | 21.880000 | -10.650000 | G8 | Monod 1968 |
| BIB_0053 | Artiodactyla | Bovidae | Gazella dorcas | 20.500000 | -8.000000 | H11 | East 1999 |
| BIB_0052 | Artiodactyla | Bovidae | Gazella dorcas | 16.780000 | -14.920000 | L4 | Dia 2004 |
| BIB_0051 | Artiodactyla | Bovidae | Gazella dorcas | 24.364000 | -6.765000 | D12 | East 1999 |
| BIB_0050 | Artiodactyla | Bovidae | Gazella dorcas | 19.973059 | -12.477612 | I6 | Favotti 1960 |
| BIB_0049 | Artiodactyla | Bovidae | Gazella dorcas | 17.950000 | -12.250000 | K7 | Tellería 2009 |
| BIB_0048 | Artiodactyla | Bovidae | Gazella dorcas | 16.830000 | -15.700000 | L3 | Dia 2004; Gueye and Dia 2004 |
| BIB_0047 | Artiodactyla | Bovidae | Gazella dorcas | 18.333333 | -9.083333 | J10 | Holl 1985 |
| BIB_0046 | Artiodactyla | Bovidae | Gazella dorcas | 18.450000 | -9.500000 | J9 | Holl 2009 |
| BIB_0045 | Artiodactyla | Bovidae | Gazella dorcas | 16.510000 | -14.190000 | M5 | Dia 2004 |
| BIB_0044 | Artiodactyla | Bovidae | Gazella dorcas | 20.585660 | -16.236789 | H2 | Verschuren 1984 |
| BIB_0043 | Artiodactyla | Bovidae | Gazella dorcas | 20.585660 | -16.236789 | H2 | Gowthorpe 1993 |
| BIB_0042 | Artiodactyla | Bovidae | Gazella dorcas | 20.831667 | -14.961024 | H4 | Granjon et al 1997 |
| BIB_0041 | Artiodactyla | Bovidae | Gazella dorcas | 17.888000 | -15.509000 | K3 | Thiam 2020 |
| BIB_0040 | Artiodactyla | Bovidae | Gazella dorcas | 15.300000 | -12.500000 | N6 | Dia 2004 |
| BIB_0039 | Artiodactyla | Bovidae | Gazella dorcas | 23.328000 | -6.613000 | E12 | East 1999 |
| BIB_0037 | Artiodactyla | Bovidae | Gazella dorcas | 19.333333 | -16.283333 | I2 | Granjon et al 1997 |
| 15434 | Artiodactyla | Bovidae | Gazella dorcas | 21.500223 | -11.619505 | G7 |  |
| 15433 | Artiodactyla | Bovidae | Gazella dorcas | 20.700000 | -13.100000 | H6 |  |
| 15217 | Artiodactyla | Bovidae | Gazella dorcas | 18.951333 | -15.254667 | J3 | Silva et al 2015 |
| 14147 | Artiodactyla | Bovidae | Gazella dorcas | 20.944650 | -14.778967 | H4 |  |
| 14146 | Artiodactyla | Bovidae | Gazella dorcas | 20.944650 | -14.778967 | H4 |  |
| 14145 | Artiodactyla | Bovidae | Gazella dorcas | 20.944650 | -14.778967 | H4 |  |
| 14144 | Artiodactyla | Bovidae | Gazella dorcas | 20.944650 | -14.778967 | H4 |  |
| 14143 | Artiodactyla | Bovidae | Gazella dorcas | 20.944650 | -14.778967 | H4 |  |
| 14141 | Artiodactyla | Bovidae | Gazella dorcas | 20.944650 | -14.778967 | H4 |  |
| 13691 | Artiodactyla | Bovidae | Gazella dorcas | 20.273300 | -14.529500 | H4 |  |
| 13690 | Artiodactyla | Bovidae | Gazella dorcas | 18.428100 | -10.354100 | J9 |  |
| 6575 | Artiodactyla | Bovidae | Gazella dorcas | 19.705000 | -16.390000 | I2 |  |
| 6264 | Artiodactyla | Bovidae | Gazella dorcas | 19.705000 | -16.390000 | I2 |  |
| 5992 | Artiodactyla | Bovidae | Gazella dorcas | 19.806500 | -16.370000 | I2 |  |
| 5991 | Artiodactyla | Bovidae | Gazella dorcas | 19.806500 | -16.370000 | I2 |  |
| 5990 | Artiodactyla | Bovidae | Gazella dorcas | 19.806500 | -16.370000 | I2 |  |
| 5989 | Artiodactyla | Bovidae | Gazella dorcas | 19.806500 | -16.370000 | I2 |  |
| 5988 | Artiodactyla | Bovidae | Gazella dorcas | 19.705000 | -16.390000 | I2 |  |
| 5987 | Artiodactyla | Bovidae | Gazella dorcas | 19.705000 | -16.390000 | I2 |  |
| 5978 | Artiodactyla | Bovidae | Gazella dorcas | 19.705000 | -16.390000 | I2 |  |
| 5976 | Artiodactyla | Bovidae | Gazella dorcas | 19.806500 | -16.370000 | I2 |  |
| 5965 | Artiodactyla | Bovidae | Gazella dorcas | 19.806500 | -16.370000 | I2 |  |
| 5961 | Artiodactyla | Bovidae | Gazella dorcas | 19.806500 | -16.370000 | I2 |  |
| 5960 | Artiodactyla | Bovidae | Gazella dorcas | 19.806500 | -16.370000 | I2 |  |
| 5958 | Artiodactyla | Bovidae | Gazella dorcas | 19.806500 | -16.370000 | I2 |  |
| 5957 | Artiodactyla | Bovidae | Gazella dorcas | 19.806500 | -16.370000 | I2 |  |
| 5956 | Artiodactyla | Bovidae | Gazella dorcas | 19.806500 | -16.370000 | I2 |  |
| 5955 | Artiodactyla | Bovidae | Gazella dorcas | 19.806500 | -16.370000 | I2 |  |
| 5954 | Artiodactyla | Bovidae | Gazella dorcas | 19.806500 | -16.370000 | I2 |  |
| 5948 | Artiodactyla | Bovidae | Gazella dorcas | 19.806500 | -16.370000 | I2 |  |
| 5944 | Artiodactyla | Bovidae | Gazella dorcas | 19.806500 | -16.370000 | I2 |  |
| 5941 | Artiodactyla | Bovidae | Gazella dorcas | 19.806500 | -16.370000 | I2 |  |
| 5930 | Artiodactyla | Bovidae | Gazella dorcas | 19.806500 | -16.370000 | I2 |  |
| 5899 | Artiodactyla | Bovidae | Gazella dorcas | 21.500223 | -11.619505 | G7 |  |
| 5898 | Artiodactyla | Bovidae | Gazella dorcas | 21.500223 | -11.619505 | G7 |  |
| 5892 | Artiodactyla | Bovidae | Gazella dorcas | 21.500223 | -11.619505 | G7 |  |
| 5891 | Artiodactyla | Bovidae | Gazella dorcas | 21.500223 | -11.619505 | G7 |  |
| 5889 | Artiodactyla | Bovidae | Gazella dorcas | 21.500223 | -11.619505 | G7 |  |
| 5888 | Artiodactyla | Bovidae | Gazella dorcas | 21.500223 | -11.619505 | G7 |  |
| 5886 | Artiodactyla | Bovidae | Gazella dorcas | 21.500223 | -11.619505 | G7 |  |
| 5775 | Artiodactyla | Bovidae | Gazella dorcas | 21.526448 | -12.864365 | G6 |  |
| 3570 | Artiodactyla | Bovidae | Gazella dorcas | 21.663533 | -11.683610 | G7 |  |
| 1452 | Artiodactyla | Bovidae | Gazella dorcas | 19.705000 | -16.390000 | I2 |  |
| BIB_0063 | Artiodactyla | Bovidae | Hippotragus equinus | 14.775834 | -12.213947 | O7 | Mauritanie 2000 SARL 2001 |
| BIB_0062 | Artiodactyla | Bovidae | Hippotragus equinus | 14.790000 | -12.110000 | N7 | East 1999 |
| BIB_0061 | Artiodactyla | Bovidae | Hippotragus equinus | 18.333333 | -9.083333 | J10 | Holl 1985 |
| BIB_0060 | Artiodactyla | Bovidae | Hippotragus equinus | 18.450000 | -9.500000 | J9 | Holl 2009 |
| BIB_0068 | Artiodactyla | Bovidae | Kobus kob | 14.890000 | -12.420000 | N6 | East 1999 |
| BIB_0067 | Artiodactyla | Bovidae | Kobus kob | 14.775834 | -12.213947 | O7 | Mauritanie 2000 SARL 2001 |
| BIB_0066 | Artiodactyla | Bovidae | Kobus kob | 16.710000 | -16.120000 | L3 | Gueye and Dia 2004 |
| BIB_0065 | Artiodactyla | Bovidae | Kobus kob | 16.830000 | -15.700000 | L3 | Dia 2004; Gueye and Dia 2004 |
| BIB_0064 | Artiodactyla | Bovidae | Kobus kob | 15.300000 | -12.500000 | N6 | Dia 2004; Gueye and Dia 2004 |
| BIB_0097 | Artiodactyla | Bovidae | Nanger dama | 16.260000 | -13.940000 | M5 | Dia 2004; Gueye and Dia 2004 |
| BIB_0096 | Artiodactyla | Bovidae | Nanger dama | 18.540000 | -11.440000 | J7 | Newby 1984; Le Berre 1990 |
| BIB_0095 | Artiodactyla | Bovidae | Nanger dama | 18.533444 | -11.425170 | J7 | Moreno 2009 |
| BIB_0094 | Artiodactyla | Bovidae | Nanger dama | 18.520000 | -12.010000 | J7 | Denys et al 2001 |
| BIB_0093 | Artiodactyla | Bovidae | Nanger dama | 20.560000 | -15.310000 | H3 | Beudels-Jamar et al 2006 |
| BIB_0092 | Artiodactyla | Bovidae | Nanger dama | 14.775834 | -12.213947 | O7 | Mauritanie 2000 SARL 2001 |
| BIB_0091 | Artiodactyla | Bovidae | Nanger dama | 16.303340 | -16.401357 | M2 | National Research Council 1981 |
| BIB_0090 | Artiodactyla | Bovidae | Nanger dama | 16.710000 | -16.120000 | L3 | Gueye and Dia 2004 |
| BIB_0089 | Artiodactyla | Bovidae | Nanger dama | 16.780000 | -14.920000 | L4 | Dia 2004 |
| BIB_0088 | Artiodactyla | Bovidae | Nanger dama | 16.615280 | -11.402313 | L7 | Moreno 2009 |
| BIB_0087 | Artiodactyla | Bovidae | Nanger dama | 16.600000 | -5.620000 | L14 | Sevenet 1943 |
| BIB_0086 | Artiodactyla | Bovidae | Nanger dama | 16.600000 | -5.620000 | L14 | Beudels-Jamar et al 2006 |
| BIB_0085 | Artiodactyla | Bovidae | Nanger dama | 19.751600 | -14.428100 | I4 | Jullien and Petter 1970 |
| BIB_0084 | Artiodactyla | Bovidae | Nanger dama | 16.830000 | -15.700000 | L3 | Dia 2004; Gueye and Dia 2004 |
| BIB_0083 | Artiodactyla | Bovidae | Nanger dama | 18.333333 | -9.083333 | J10 | Holl 1985 |
| BIB_0082 | Artiodactyla | Bovidae | Nanger dama | 18.450000 | -9.500000 | J9 | Holl 2009 |
| BIB_0081 | Artiodactyla | Bovidae | Nanger dama | 18.450000 | -9.500000 | J9 | Beudels-Jamar et al 2006 |
| BIB_0080 | Artiodactyla | Bovidae | Nanger dama | 17.820000 | -7.450000 | K12 | Beudels-Jamar et al 2006 |
| BIB_0079 | Artiodactyla | Bovidae | Nanger dama | 16.510000 | -14.190000 | M5 | Dia 2004 |
| BIB_0078 | Artiodactyla | Bovidae | Nanger dama | 25.857000 | -11.810000 | B7 | Aulagnier et al 2017 |
| BIB_0077 | Artiodactyla | Bovidae | Nanger dama | 25.220000 | -11.577000 | C7 | Beudels-Jamar et al 2006 |
| BIB_0076 | Artiodactyla | Bovidae | Nanger dama | 15.760000 | -5.880000 | M13 | Beudels-Jamar et al 2006 |
| BIB_0075 | Artiodactyla | Bovidae | Nanger dama | 19.666667 | -16.166667 | I2 | Convention on Migratory Species 1998 |
| BIB_0074 | Artiodactyla | Bovidae | Nanger dama | 21.210000 | -14.500000 | G4 | Beudels-Jamar et al 2006 |
| BIB_0073 | Artiodactyla | Bovidae | Nanger dama | 15.300000 | -12.500000 | N6 | Dia 2004 |
| BIB_0072 | Artiodactyla | Bovidae | Nanger dama | 18.170000 | -6.670000 | K12 | Beudels-Jamar et al 2006 |
| BIB_0071 | Artiodactyla | Bovidae | Nanger dama | 21.000000 | -6.500000 | G13 | Sevenet 1943 |
| BIB_0070 | Artiodactyla | Bovidae | Nanger dama | 20.500000 | -13.250000 | H5 | Newby 1984; Le Berre 1990 |
| BIB_0069 | Artiodactyla | Bovidae | Nanger dama | 19.263695 | -10.511706 | I8 | Sevenet 1943 |
| BIB_0116 | Artiodactyla | Bovidae | Oryx dammah | 18.400764 | -9.491781 | J9 | Moreno 2009 |
| BIB_0115 | Artiodactyla | Bovidae | Oryx dammah | 14.775834 | -12.213947 | O7 | Mauritanie 2000 SARL 2001 |
| BIB_0114 | Artiodactyla | Bovidae | Oryx dammah | 15.470000 | -12.950000 | N6 | Gueye and Dia 2004 |
| BIB_0113 | Artiodactyla | Bovidae | Oryx dammah | 16.710000 | -16.120000 | L3 | Gueye and Dia 2004 |
| BIB_0112 | Artiodactyla | Bovidae | Oryx dammah | 16.600000 | -5.620000 | L14 | Sevenet 1943 |
| BIB_0111 | Artiodactyla | Bovidae | Oryx dammah | 18.710000 | -11.550000 | J7 | Campbell et al 2006 |
| BIB_0110 | Artiodactyla | Bovidae | Oryx dammah | 16.830000 | -15.700000 | L3 | Gueye and Dia 2004 |
| BIB_0109 | Artiodactyla | Bovidae | Oryx dammah | 18.333333 | -9.083333 | J10 | Holl 1985 |
| BIB_0108 | Artiodactyla | Bovidae | Oryx dammah | 18.480000 | -9.460000 | J9 | Beudels-Jamar et al 2006 |
| BIB_0107 | Artiodactyla | Bovidae | Oryx dammah | 18.450000 | -9.500000 | J9 | Holl 2009 |
| BIB_0106 | Artiodactyla | Bovidae | Oryx dammah | 17.820000 | -7.450000 | K12 | Beudels-Jamar et al 2006 |
| BIB_0105 | Artiodactyla | Bovidae | Oryx dammah | 16.510000 | -14.190000 | M5 | Gueye and Dia 2004 |
| BIB_0104 | Artiodactyla | Bovidae | Oryx dammah | 21.210000 | -14.500000 | G4 | Beudels-Jamar et al 2006; Aulagnier et al 2017 |
| BIB_0103 | Artiodactyla | Bovidae | Oryx dammah | 17.888000 | -15.509000 | K3 | Thiam 2020 |
| BIB_0102 | Artiodactyla | Bovidae | Oryx dammah | 15.300000 | -12.500000 | N6 | Dia 2004; Gueye and Dia 2004 |
| BIB_0101 | Artiodactyla | Bovidae | Oryx dammah | 18.170000 | -6.670000 | K12 | Beudels-Jamar et al 2006 |
| BIB_0100 | Artiodactyla | Bovidae | Oryx dammah | 21.000000 | -6.500000 | G13 | Sevenet 1943 |
| BIB_0099 | Artiodactyla | Bovidae | Oryx dammah | 19.263695 | -10.511706 | I8 | Sevenet 1943 |
| BIB_0098 | Artiodactyla | Bovidae | Oryx dammah | 19.263695 | -10.511706 | I8 | Beudels-Jamar et al 2006 |
| BIB_0120 | Artiodactyla | Bovidae | Redunca redunca | 14.890000 | -12.420000 | N6 | East 1999 |
| BIB_0119 | Artiodactyla | Bovidae | Redunca redunca | 14.775834 | -12.213947 | O7 | Mauritanie 2000 SARL 2001 |
| BIB_0118 | Artiodactyla | Bovidae | Redunca redunca | 16.303340 | -16.401357 | M2 | National Research Council 1981 |
| BIB_0117 | Artiodactyla | Bovidae | Redunca redunca | 18.450000 | -9.500000 | J9 | Holl 2009 |
| BIB_0122 | Artiodactyla | Bovidae | Taurotragus derbianus | 18.333333 | -9.083333 | J10 | Holl 1985 |
| BIB_0121 | Artiodactyla | Bovidae | Taurotragus derbianus | 18.450000 | -9.500000 | J9 | Holl 2009 |
| BIB_0131 | Artiodactyla | Bovidae | Tragelaphus scriptus | 14.890000 | -12.420000 | N6 | East 1999 |
| BIB_0130 | Artiodactyla | Bovidae | Tragelaphus scriptus | 14.775834 | -12.213947 | O7 | Mauritanie 2000 SARL 2001 |
| BIB_0129 | Artiodactyla | Bovidae | Tragelaphus scriptus | 16.303340 | -16.401357 | M2 | National Research Council 1981 |
| BIB_0128 | Artiodactyla | Bovidae | Tragelaphus scriptus | 15.470000 | -12.950000 | N6 | Gueye and Dia 2004 |
| BIB_0127 | Artiodactyla | Bovidae | Tragelaphus scriptus | 16.710000 | -16.120000 | L3 | Gueye and Dia 2004 |
| BIB_0126 | Artiodactyla | Bovidae | Tragelaphus scriptus | 16.830000 | -15.700000 | L3 | Dia 2004; Gueye and Dia 2004 |
| BIB_0125 | Artiodactyla | Bovidae | Tragelaphus scriptus | 16.120000 | -13.690000 | M5 | Gueye and Dia 2004 |
| BIB_0124 | Artiodactyla | Bovidae | Tragelaphus scriptus | 16.510000 | -14.190000 | M5 | Gueye and Dia 2004 |
| BIB_0123 | Artiodactyla | Bovidae | Tragelaphus scriptus | 15.300000 | -12.500000 | N6 | Dia 2004; Gueye and Dia 2004 |
| BIB_0171 | Artiodactyla | Giraffidae | Giraffa camelopardalis | 23.446321 | -11.260584 | E7 | Ciofolo 1995 |
| BIB_0170 | Artiodactyla | Giraffidae | Giraffa camelopardalis | 22.906306 | -12.505314 | E6 | Messerli and Winiger 1992 |
| BIB_0169 | Artiodactyla | Giraffidae | Giraffa camelopardalis | 16.260000 | -13.940000 | M5 | Gueye and Dia 2004 |
| BIB_0168 | Artiodactyla | Giraffidae | Giraffa camelopardalis | 16.575135 | -10.505997 | L8 | Messerli and Winiger 1992 |
| BIB_0167 | Artiodactyla | Giraffidae | Giraffa camelopardalis | 19.115934 | -12.880186 | J6 | Messerli and Winiger 1992 |
| BIB_0166 | Artiodactyla | Giraffidae | Giraffa camelopardalis | 14.775834 | -12.213947 | O7 | Mauritanie 2000 SARL 2001 |
| BIB_0165 | Artiodactyla | Giraffidae | Giraffa camelopardalis | 19.865678 | -13.005143 | I6 | Messerli and Winiger 1992 |
| BIB_0164 | Artiodactyla | Giraffidae | Giraffa camelopardalis | 19.407501 | -12.880186 | I6 | Messerli and Winiger 1992 |
| BIB_0163 | Artiodactyla | Giraffidae | Giraffa camelopardalis | 20.782032 | -15.587595 | H3 | Messerli and Winiger 1992 |
| BIB_0162 | Artiodactyla | Giraffidae | Giraffa camelopardalis | 15.470000 | -12.950000 | N6 | Gueye and Dia 2004 |
| BIB_0161 | Artiodactyla | Giraffidae | Giraffa camelopardalis | 16.738462 | -7.768822 | L11 | Ciofolo 1995 |
| BIB_0160 | Artiodactyla | Giraffidae | Giraffa camelopardalis | 18.157928 | -12.047137 | K7 | Messerli and Winiger 1992 |
| BIB_0159 | Artiodactyla | Giraffidae | Giraffa camelopardalis | 16.830351 | -13.374019 | L5 | Ciofolo 1995 |
| BIB_0158 | Artiodactyla | Giraffidae | Giraffa camelopardalis | 17.197905 | -13.374019 | L5 | Ciofolo 1995 |
| BIB_0157 | Artiodactyla | Giraffidae | Giraffa camelopardalis | 16.200263 | -7.715283 | M11 | Messerli and Winiger 1992 |
| BIB_0156 | Artiodactyla | Giraffidae | Giraffa camelopardalis | 21.281861 | -15.087766 | G4 | Messerli and Winiger 1992 |
| BIB_0155 | Artiodactyla | Giraffidae | Giraffa camelopardalis | 18.710000 | -11.550000 | J7 | Campbell et al 2006 |
| BIB_0154 | Artiodactyla | Giraffidae | Giraffa camelopardalis | 17.709000 | -11.491000 | K7 | Senones and Puigaudeau 1939 |
| BIB_0153 | Artiodactyla | Giraffidae | Giraffa camelopardalis | 17.928000 | -12.293000 | K7 | Senones and Puigaudeau 1939 |
| BIB_0152 | Artiodactyla | Giraffidae | Giraffa camelopardalis | 21.365166 | -11.422350 | G7 | Messerli and Winiger 1992 |
| BIB_0151 | Artiodactyla | Giraffidae | Giraffa camelopardalis | 21.031946 | -11.713918 | G7 | Messerli and Winiger 1992 |
| BIB_0150 | Artiodactyla | Giraffidae | Giraffa camelopardalis | 18.392455 | -10.249811 | J9 | Ciofolo 1995 |
| BIB_0149 | Artiodactyla | Giraffidae | Giraffa camelopardalis | 18.208678 | -12.455135 | K6 | Ciofolo 1995 |
| BIB_0148 | Artiodactyla | Giraffidae | Giraffa camelopardalis | 16.830000 | -15.700000 | L3 | Gueye and Dia 2004 |
| BIB_0147 | Artiodactyla | Giraffidae | Giraffa camelopardalis | 16.120000 | -13.690000 | M5 | Dia 2004; Gueye and Dia 2004 |
| BIB_0146 | Artiodactyla | Giraffidae | Giraffa camelopardalis | 18.455364 | -9.463309 | J9 | Holl 2002 |
| BIB_0145 | Artiodactyla | Giraffidae | Giraffa camelopardalis | 20.782032 | -12.005485 | H7 | Messerli and Winiger 1992 |
| BIB_0144 | Artiodactyla | Giraffidae | Giraffa camelopardalis | 16.510000 | -14.190000 | M5 | Gueye and Dia 2004 |
| BIB_0143 | Artiodactyla | Giraffidae | Giraffa camelopardalis | 18.379055 | -9.116656 | J10 | Holl 2002 |
| BIB_0142 | Artiodactyla | Giraffidae | Giraffa camelopardalis | 24.947276 | -11.339046 | C7 | Messerli and Winiger 1992 |
| BIB_0141 | Artiodactyla | Giraffidae | Giraffa camelopardalis | 25.530410 | -11.047478 | C8 | Messerli and Winiger 1992 |
| BIB_0140 | Artiodactyla | Giraffidae | Giraffa camelopardalis | 25.530410 | -11.547308 | C7 | Messerli and Winiger 1992 |
| BIB_0139 | Artiodactyla | Giraffidae | Giraffa camelopardalis | 17.106016 | -10.157923 | L9 | Ciofolo 1995 |
| BIB_0138 | Artiodactyla | Giraffidae | Giraffa camelopardalis | 17.888000 | -15.509000 | K3 | Thiam 2020 |
| BIB_0137 | Artiodactyla | Giraffidae | Giraffa camelopardalis | 20.407160 | -13.338363 | H5 | Messerli and Winiger 1992 |
| BIB_0136 | Artiodactyla | Giraffidae | Giraffa camelopardalis | 20.782032 | -12.713576 | H6 | Messerli and Winiger 1992 |
| BIB_0135 | Artiodactyla | Giraffidae | Giraffa camelopardalis | 15.300000 | -12.500000 | N6 | Dia 2004; Gueye and Dia 2004 |
| BIB_0134 | Artiodactyla | Giraffidae | Giraffa camelopardalis | 19.770782 | -14.384793 | I4 | Ciofolo 1995 |
| BIB_0133 | Artiodactyla | Giraffidae | Giraffa camelopardalis | 18.126000 | -11.916000 | K7 | Senones and Puigaudeau 1939 |
| BIB_0132 | Artiodactyla | Giraffidae | Giraffa camelopardalis | 18.100000 | -12.000000 | K7 | Ciofolo 1995 |
| BIB_0177 | Artiodactyla | Hippopotamidae | Hippopotamus amphibius | 15.470000 | -12.950000 | N6 | Mauritanie 2000 SARL 2001 |
| BIB_0176 | Artiodactyla | Hippopotamidae | Hippopotamus amphibius | 15.470000 | -12.950000 | N6 | Dia 2004 |
| BIB_0175 | Artiodactyla | Hippopotamidae | Hippopotamus amphibius | 16.303340 | -16.401357 | M2 | Ba et al 2002 |
| BIB_0174 | Artiodactyla | Hippopotamidae | Hippopotamus amphibius | 18.333333 | -9.083333 | J10 | Holl 1985 |
| BIB_0173 | Artiodactyla | Hippopotamidae | Hippopotamus amphibius | 18.450000 | -9.500000 | J9 | Holl 2009 |
| BIB_0172 | Artiodactyla | Hippopotamidae | Hippopotamus amphibius | 16.510000 | -14.190000 | M5 | Dia 2004; Gueye and Dia 2004 |
| 19647 | Artiodactyla | Hippopotamidae | Hippopotamus amphibius | 14.901400 | -11.798900 | N7 |  |
| 18585 | Artiodactyla | Hippopotamidae | Hippopotamus amphibius | 15.498203 | -12.954795 | N6 |  |
| 7470 | Artiodactyla | Hippopotamidae | Hippopotamus amphibius | 15.444237 | -12.946745 | N6 |  |
| 4653 | Artiodactyla | Hippopotamidae | Hippopotamus amphibius | 15.500420 | -12.956430 | N6 |  |
| BIB_0214 | Artiodactyla | Suidae | Phacochoerus africanus | 14.775834 | -12.213947 | O7 | Mauritanie 2000 SARL 2001 |
| BIB_0213 | Artiodactyla | Suidae | Phacochoerus africanus | 16.520424 | -16.279112 | M2 | Diarra 1994 |
| BIB_0210 | Artiodactyla | Suidae | Phacochoerus africanus | 15.470000 | -12.950000 | N6 | Gueye and Dia 2004 |
| BIB_0208 | Artiodactyla | Suidae | Phacochoerus africanus | 16.710000 | -16.120000 | L3 | Dia 2004; Gueye and Dia 2004 |
| BIB_0207 | Artiodactyla | Suidae | Phacochoerus africanus | 16.398708 | -7.665378 | M11 | Nickel 2003 |
| BIB_0206 | Artiodactyla | Suidae | Phacochoerus africanus | 16.780000 | -14.920000 | L4 | Dia 2004; Gueye and Dia 2004 |
| BIB_0203 | Artiodactyla | Suidae | Phacochoerus africanus | 17.950000 | -12.250000 | K7 | Tellería 2009 |
| BIB_0202 | Artiodactyla | Suidae | Phacochoerus africanus | 16.830000 | -15.700000 | L3 | Dia 2004; Gueye and Dia 2004 |
| BIB_0201 | Artiodactyla | Suidae | Phacochoerus africanus | 16.120000 | -13.690000 | M5 | Dia 2004; Gueye and Dia 2004 |
| BIB_0183 | Artiodactyla | Suidae | Phacochoerus africanus | 16.400000 | -16.400000 | M2 | Spatz 1930 |
| BIB_0182 | Artiodactyla | Suidae | Phacochoerus africanus | 16.303340 | -16.401357 | M2 | National Research Council 1981; Ly and Zein 2009 |
| BIB_0180 | Artiodactyla | Suidae | Phacochoerus africanus | 18.450000 | -9.500000 | J9 | Holl 2009 |
| BIB_0179 | Artiodactyla | Suidae | Phacochoerus africanus | 16.607000 | -16.439323 | M2 | Lafont and Measson 2001 |
| BIB_0178 | Artiodactyla | Suidae | Phacochoerus africanus | 15.300000 | -12.500000 | N6 | Dia 2004 |
| 19916 | Artiodactyla | Suidae | Phacochoerus africanus | 16.313810 | -16.397398 | M2 |  |
| 19915 | Artiodactyla | Suidae | Phacochoerus africanus | 16.242438 | -16.419047 | M2 |  |
| 19914 | Artiodactyla | Suidae | Phacochoerus africanus | 16.348385 | -16.399160 | M2 |  |
| 19650 | Artiodactyla | Suidae | Phacochoerus africanus | 19.521100 | -16.418300 | I2 |  |
| 18569 | Artiodactyla | Suidae | Phacochoerus africanus | 16.367770 | -16.419638 | M2 |  |
| 16181 | Artiodactyla | Suidae | Phacochoerus africanus | 16.413341 | -16.332027 | M2 |  |
| 16175 | Artiodactyla | Suidae | Phacochoerus africanus | 16.436300 | -16.319882 | M2 |  |
| 13887 | Artiodactyla | Suidae | Phacochoerus africanus | 16.527492 | -16.421770 | M2 |  |
| 12887 | Artiodactyla | Suidae | Phacochoerus africanus | 16.607937 | -16.313605 | M2 |  |
| 12886 | Artiodactyla | Suidae | Phacochoerus africanus | 16.583798 | -16.307665 | M2 |  |
| 12885 | Artiodactyla | Suidae | Phacochoerus africanus | 16.564938 | -16.279850 | M2 |  |
| BIB_0313 | Carnivora | Canidae | Canis lupaster | 16.260000 | -13.940000 | M5 | Dia 2004 |
| BIB_0312 | Carnivora | Canidae | Canis lupaster | 19.661631 | -16.489712 | I2 | Simões 2001 |
| BIB_0309 | Carnivora | Canidae | Canis lupaster | 19.643171 | -16.416162 | I2 | Dia 2004; Gueye and Dia 2004 |
| BIB_0308 | Carnivora | Canidae | Canis lupaster | 19.848636 | -16.351575 | I2 | Simões 2001 |
| BIB_0307 | Carnivora | Canidae | Canis lupaster | 19.749057 | -16.408212 | I2 | Mahé 1985 |
| BIB_0306 | Carnivora | Canidae | Canis lupaster | 19.633333 | -16.425000 | I2 | Gowthorpe 1993 |
| BIB_0305 | Carnivora | Canidae | Canis lupaster | 19.938184 | -16.250071 | I2 | Mahé 1985 |
| BIB_0304 | Carnivora | Canidae | Canis lupaster | 14.775834 | -12.213947 | O7 | Mauritanie 2000 SARL 2001 |
| BIB_0303 | Carnivora | Canidae | Canis lupaster | 16.303340 | -16.401357 | M2 | National Research Council 1981 |
| BIB_0302 | Carnivora | Canidae | Canis lupaster | 16.520424 | -16.279112 | M2 | Diarra 1994 |
| BIB_0300 | Carnivora | Canidae | Canis lupaster | 16.384695 | -9.300555 | M10 | Kirsch-Jung and Khtour 2007 |
| BIB_0299 | Carnivora | Canidae | Canis lupaster | 17.244936 | -10.670560 | L8 | Kirsch-Jung and Khtour 2007 |
| BIB_0298 | Carnivora | Canidae | Canis lupaster | 15.504833 | -9.816833 | N9 | Kirsch-Jung and Khtour 2007 |
| BIB_0297 | Carnivora | Canidae | Canis lupaster | 20.495493 | -16.210280 | H2 | Mahé 1985 |
| BIB_0296 | Carnivora | Canidae | Canis lupaster | 19.364583 | -16.465478 | I2 | Mahé 1985 |
| BIB_0295 | Carnivora | Canidae | Canis lupaster | 18.189955 | -16.007304 | K3 | Granjon et al 1997 |
| BIB_0294 | Carnivora | Canidae | Canis lupaster | 21.159517 | -16.974929 | G2 | Mahé 1985 |
| BIB_0293 | Carnivora | Canidae | Canis lupaster | 15.470000 | -12.950000 | N6 | Dia 2004 |
| BIB_0292 | Carnivora | Canidae | Canis lupaster | 19.870735 | -16.390955 | I2 | Simões 2001 |
| BIB_0291 | Carnivora | Canidae | Canis lupaster | 20.591667 | -13.175000 | H6 | Dekeyser and Villiers 1956 |
| BIB_0290 | Carnivora | Canidae | Canis lupaster | 19.553701 | -16.387410 | I2 | Mahé 1985 |
| BIB_0289 | Carnivora | Canidae | Canis lupaster | 19.880000 | -10.170000 | I9 | Monod 1961 |
| BIB_0288 | Carnivora | Canidae | Canis lupaster | 16.398708 | -7.665378 | M11 | Nickel 2003 |
| BIB_0287 | Carnivora | Canidae | Canis lupaster | 16.780000 | -14.920000 | L4 | Dia 2004 |
| BIB_0286 | Carnivora | Canidae | Canis lupaster | 19.709032 | -16.486888 | I2 | Simões 2001 |
| BIB_0285 | Carnivora | Canidae | Canis lupaster | 19.712360 | -16.482097 | I2 | Mahé 1985 |
| BIB_0283 | Carnivora | Canidae | Canis lupaster | 20.490000 | -13.076667 | H6 | Dekeyser and Villiers 1956 |
| BIB_0264 | Carnivora | Canidae | Canis lupaster | 20.343333 | -13.145000 | H6 | Dekeyser and Villiers 1956 |
| BIB_0263 | Carnivora | Canidae | Canis lupaster | 20.330000 | -13.156667 | H6 | Dekeyser and Villiers 1956 |
| BIB_0262 | Carnivora | Canidae | Canis lupaster | 19.751600 | -14.428100 | I4 | Jullien and Petter 1970 |
| BIB_0261 | Carnivora | Canidae | Canis lupaster | 16.399252 | -9.559874 | M9 | Kirsch-Jung and Khtour 2007 |
| BIB_0260 | Carnivora | Canidae | Canis lupaster | 17.950000 | -12.250000 | K7 | Tellería 2009 |
| BIB_0259 | Carnivora | Canidae | Canis lupaster | 17.069652 | -10.223949 | L9 | Kirsch-Jung and Khtour 2007 |
| BIB_0258 | Carnivora | Canidae | Canis lupaster | 16.830000 | -15.700000 | L3 | Dia 2004; Gueye and Dia 2004 |
| BIB_0257 | Carnivora | Canidae | Canis lupaster | 20.264750 | -16.109693 | H2 | Mahé 1985 |
| BIB_0256 | Carnivora | Canidae | Canis lupaster | 16.120000 | -13.690000 | M5 | Dia 2004 |
| BIB_0255 | Carnivora | Canidae | Canis lupaster | 16.400000 | -16.400000 | M2 | Spatz 1930 |
| BIB_0254 | Carnivora | Canidae | Canis lupaster | 16.303340 | -16.401357 | M2 | Ly and Zein 2009 |
| BIB_0252 | Carnivora | Canidae | Canis lupaster | 16.607000 | -16.439323 | M2 | Lafont and Measson 2001 |
| BIB_0251 | Carnivora | Canidae | Canis lupaster | 16.607000 | -16.439323 | M2 | Granjon et al 1997 |
| BIB_0250 | Carnivora | Canidae | Canis lupaster | 19.635880 | -16.490018 | I2 | Simões 2001 |
| BIB_0249 | Carnivora | Canidae | Canis lupaster | 19.734382 | -16.258397 | I2 | Mahé 1985 |
| BIB_0248 | Carnivora | Canidae | Canis lupaster | 20.127154 | -16.261000 | I2 | Mahé 1985 |
| BIB_0247 | Carnivora | Canidae | Canis lupaster | 20.687000 | -16.674000 | H2 | Granjon et al 1997 |
| BIB_0246 | Carnivora | Canidae | Canis lupaster | 20.687000 | -16.674000 | H2 | Mahé 1985 |
| BIB_0245 | Carnivora | Canidae | Canis lupaster | 20.830961 | -17.056299 | H1 | Mahé 1985 |
| BIB_0244 | Carnivora | Canidae | Canis lupaster | 16.510000 | -14.190000 | M5 | Dia 2004; Gueye and Dia 2004 |
| BIB_0241 | Carnivora | Canidae | Canis lupaster | 20.585660 | -16.236789 | H2 | Hughes et al 1992 |
| BIB_0240 | Carnivora | Canidae | Canis lupaster | 19.970200 | -16.231150 | I2 | Flanagan 2006 |
| BIB_0239 | Carnivora | Canidae | Canis lupaster | 19.966550 | -16.199900 | I2 | Flanagan 2006 |
| BIB_0238 | Carnivora | Canidae | Canis lupaster | 19.952050 | -16.248750 | I2 | Flanagan 2006 |
| BIB_0237 | Carnivora | Canidae | Canis lupaster | 19.907383 | -16.253517 | I2 | Flanagan 2006 |
| BIB_0236 | Carnivora | Canidae | Canis lupaster | 19.898817 | -16.308083 | I2 | Flanagan 2006 |
| BIB_0235 | Carnivora | Canidae | Canis lupaster | 19.823083 | -16.256417 | I2 | Flanagan 2006 |
| BIB_0234 | Carnivora | Canidae | Canis lupaster | 19.770017 | -16.263500 | I2 | Flanagan 2006 |
| BIB_0233 | Carnivora | Canidae | Canis lupaster | 19.565467 | -16.461800 | I2 | Simões 2001 |
| BIB_0232 | Carnivora | Canidae | Canis lupaster | 19.537483 | -16.413667 | I2 | Flanagan 2006 |
| BIB_0231 | Carnivora | Canidae | Canis lupaster | 19.532083 | -16.417783 | I2 | Flanagan 2006 |
| BIB_0230 | Carnivora | Canidae | Canis lupaster | 19.529167 | -16.419017 | I2 | Flanagan 2006 |
| BIB_0229 | Carnivora | Canidae | Canis lupaster | 19.527983 | -16.376217 | I2 | Flanagan 2006 |
| BIB_0228 | Carnivora | Canidae | Canis lupaster | 19.524850 | -16.378167 | I2 | Flanagan 2006 |
| BIB_0227 | Carnivora | Canidae | Canis lupaster | 19.499717 | -16.274467 | I2 | Flanagan 2006 |
| BIB_0226 | Carnivora | Canidae | Canis lupaster | 19.479417 | -16.282733 | I2 | Flanagan 2006 |
| BIB_0225 | Carnivora | Canidae | Canis lupaster | 19.471517 | -16.283017 | I2 | Flanagan 2006 |
| BIB_0224 | Carnivora | Canidae | Canis lupaster | 19.459050 | -16.331617 | I2 | Flanagan 2006 |
| BIB_0223 | Carnivora | Canidae | Canis lupaster | 19.402850 | -16.394400 | I2 | Flanagan 2006 |
| BIB_0222 | Carnivora | Canidae | Canis lupaster | 19.392950 | -16.354450 | I2 | Flanagan 2006 |
| BIB_0221 | Carnivora | Canidae | Canis lupaster | 19.376650 | -16.443767 | I2 | Flanagan 2006 |
| BIB_0220 | Carnivora | Canidae | Canis lupaster | 19.374733 | -16.450867 | I2 | Flanagan 2006 |
| BIB_0219 | Carnivora | Canidae | Canis lupaster | 19.369333 | -16.476850 | I2 | Flanagan 2006 |
| BIB_0218 | Carnivora | Canidae | Canis lupaster | 20.993050 | -17.031624 | H1 | Mahé 1985 |
| BIB_0217 | Carnivora | Canidae | Canis lupaster | 15.300000 | -12.500000 | N6 | Dia 2004; Gueye and Dia 2004 |
| BIB_0216 | Carnivora | Canidae | Canis lupaster | 19.125000 | -16.259999 | J2 | Granjon et al 1997 |
| BIB_0215 | Carnivora | Canidae | Canis lupaster | 20.101608 | -16.170559 | I2 | Mahé 1985 |
| 19780 | Carnivora | Canidae | Canis lupaster | 17.891670 | -12.124542 | K7 |  |
| 19745 | Carnivora | Canidae | Canis lupaster | 16.543913 | -10.751155 | L8 |  |
| 19743 | Carnivora | Canidae | Canis lupaster | 16.543913 | -10.751155 | L8 |  |
| 19737 | Carnivora | Canidae | Canis lupaster | 16.756482 | -11.997233 | L7 |  |
| 19677 | Carnivora | Canidae | Canis lupaster | 16.543913 | -10.751155 | L8 |  |
| 19676 | Carnivora | Canidae | Canis lupaster | 16.540090 | -10.801490 | L8 |  |
| 19017 | Carnivora | Canidae | Canis lupaster | 17.669865 | -11.352030 | K8 |  |
| 18974 | Carnivora | Canidae | Canis lupaster | 16.514157 | -16.444982 | M2 |  |
| 18937 | Carnivora | Canidae | Canis lupaster | 17.101413 | -12.256712 | L7 |  |
| 18880 | Carnivora | Canidae | Canis lupaster | 17.240833 | -12.101667 | L7 |  |
| 18825 | Carnivora | Canidae | Canis lupaster | 17.634802 | -11.414415 | K7 |  |
| 18812 | Carnivora | Canidae | Canis lupaster | 16.538033 | -10.741550 | L8 |  |
| 18810 | Carnivora | Canidae | Canis lupaster | 16.888725 | -12.184868 | L7 |  |
| 18717 | Carnivora | Canidae | Canis lupaster | 15.628413 | -11.233462 | N8 |  |
| 18705 | Carnivora | Canidae | Canis lupaster | 15.778737 | -11.356423 | M8 |  |
| 18609 | Carnivora | Canidae | Canis lupaster | 15.010340 | -12.481578 | N6 |  |
| 18583 | Carnivora | Canidae | Canis lupaster | 16.409008 | -13.983112 | M5 |  |
| 18577 | Carnivora | Canidae | Canis lupaster | 16.573233 | -16.441032 | M2 |  |
| 18573 | Carnivora | Canidae | Canis lupaster | 16.279653 | -16.515098 | M2 |  |
| 18572 | Carnivora | Canidae | Canis lupaster | 16.303340 | -16.401357 | M2 |  |
| 16616 | Carnivora | Canidae | Canis lupaster | 16.300000 | -16.400000 | M2 |  |
| 16152 | Carnivora | Canidae | Canis lupaster | 16.385838 | -16.481308 | M2 |  |
| 16132 | Carnivora | Canidae | Canis lupaster | 16.474840 | -16.302815 | M2 |  |
| 16129 | Carnivora | Canidae | Canis lupaster | 15.983935 | -12.684633 | M6 |  |
| 16125 | Carnivora | Canidae | Canis lupaster | 15.711938 | -11.325832 | M8 |  |
| 16088 | Carnivora | Canidae | Canis lupaster | 15.707348 | -11.226087 | M8 |  |
| 16035 | Carnivora | Canidae | Canis lupaster | 15.627513 | -11.320600 | N8 |  |
| 15974 | Carnivora | Canidae | Canis lupaster | 15.944687 | -11.929082 | M7 |  |
| 15973 | Carnivora | Canidae | Canis lupaster | 15.907810 | -11.906105 | M7 |  |
| 15961 | Carnivora | Canidae | Canis lupaster | 16.270153 | -12.160453 | M7 |  |
| 15950 | Carnivora | Canidae | Canis lupaster | 17.916793 | -12.253300 | K7 |  |
| 15949 | Carnivora | Canidae | Canis lupaster | 17.916980 | -12.244008 | K7 |  |
| 15948 | Carnivora | Canidae | Canis lupaster | 17.916980 | -12.244008 | K7 |  |
| 15942 | Carnivora | Canidae | Canis lupaster | 17.881083 | -12.094000 | K7 |  |
| 15912 | Carnivora | Canidae | Canis lupaster | 19.698833 | -12.723827 | I6 |  |
| 15889 | Carnivora | Canidae | Canis lupaster | 21.024758 | -11.360903 | G7 |  |
| 13881 | Carnivora | Canidae | Canis lupaster | 16.311300 | -16.397415 | M2 |  |
| 13864 | Carnivora | Canidae | Canis lupaster | 16.547455 | -12.009590 | L7 |  |
| 13856 | Carnivora | Canidae | Canis lupaster | 17.950323 | -12.197333 | K7 |  |
| 13833 | Carnivora | Canidae | Canis lupaster | 18.206040 | -11.730977 | K7 |  |
| 13807 | Carnivora | Canidae | Canis lupaster | 19.666810 | -12.639187 | I6 |  |
| 13800 | Carnivora | Canidae | Canis lupaster | 19.654210 | -12.664588 | I6 |  |
| 12902 | Carnivora | Canidae | Canis lupaster | 17.436277 | -12.394925 | L6 |  |
| 12901 | Carnivora | Canidae | Canis lupaster | 17.436277 | -12.394925 | L6 |  |
| 12888 | Carnivora | Canidae | Canis lupaster | 16.607937 | -16.313605 | M2 |  |
| BIB_0314 | Carnivora | Canidae | Lycaon pictus | 14.775834 | -12.213947 | O7 | Mauritanie 2000 SARL 2001 |
| BIB_0331 | Carnivora | Canidae | Vulpes pallida | 16.260000 | -13.940000 | M5 | Dia 2004 |
| BIB_0330 | Carnivora | Canidae | Vulpes pallida | 14.775834 | -12.213947 | O7 | Mauritanie 2000 SARL 2001 |
| BIB_0329 | Carnivora | Canidae | Vulpes pallida | 16.303340 | -16.401357 | M2 | National Research Council 1981 |
| BIB_0328 | Carnivora | Canidae | Vulpes pallida | 16.384695 | -9.300555 | M10 | Kirsch-Jung and Khtour 2007 |
| BIB_0327 | Carnivora | Canidae | Vulpes pallida | 17.244936 | -10.670560 | L8 | Kirsch-Jung and Khtour 2007 |
| BIB_0326 | Carnivora | Canidae | Vulpes pallida | 16.398708 | -7.665378 | M11 | Nickel 2003 |
| BIB_0325 | Carnivora | Canidae | Vulpes pallida | 16.780000 | -14.920000 | L4 | Dia 2004; Gueye and Dia 2004 |
| BIB_0322 | Carnivora | Canidae | Vulpes pallida | 16.120000 | -13.690000 | M5 | Gueye and Dia 2004 |
| BIB_0321 | Carnivora | Canidae | Vulpes pallida | 16.222172 | -16.490439 | M2 | Granjon et al 1997 |
| BIB_0320 | Carnivora | Canidae | Vulpes pallida | 16.607000 | -16.439323 | M2 | Lafont and Measson 2001 |
| BIB_0319 | Carnivora | Canidae | Vulpes pallida | 16.607000 | -16.439323 | M2 | Granjon et al 1997 |
| BIB_0318 | Carnivora | Canidae | Vulpes pallida | 16.540090 | -10.801490 | L8 | Nickel 2003 |
| BIB_0317 | Carnivora | Canidae | Vulpes pallida | 16.558743 | -9.606609 | L9 | Kirsch-Jung and Khtour 2007 |
| BIB_0316 | Carnivora | Canidae | Vulpes pallida | 16.510000 | -14.190000 | M5 | Dia 2004 |
| 19921 | Carnivora | Canidae | Vulpes pallida | 16.842883 | -16.137393 | L3 |  |
| 19920 | Carnivora | Canidae | Vulpes pallida | 16.701259 | -16.167486 | L2 |  |
| 19919 | Carnivora | Canidae | Vulpes pallida | 16.258737 | -16.421502 | M2 |  |
| 19918 | Carnivora | Canidae | Vulpes pallida | 17.224916 | -16.096360 | L3 |  |
| 19675 | Carnivora | Canidae | Vulpes pallida | 16.540090 | -10.801490 | L8 |  |
| 18977 | Carnivora | Canidae | Vulpes pallida | 15.476092 | -12.933600 | N6 |  |
| 18778 | Carnivora | Canidae | Vulpes pallida | 16.540090 | -10.801490 | L8 |  |
| 18756 | Carnivora | Canidae | Vulpes pallida | 16.276767 | -10.228702 | M9 |  |
| 18755 | Carnivora | Canidae | Vulpes pallida | 16.162802 | -10.317173 | M9 |  |
| 18754 | Carnivora | Canidae | Vulpes pallida | 16.064058 | -10.369157 | M9 |  |
| 18673 | Carnivora | Canidae | Vulpes pallida | 15.919212 | -11.963062 | M7 |  |
| 16127 | Carnivora | Canidae | Vulpes pallida | 15.806692 | -11.394198 | M7 |  |
| 13876 | Carnivora | Canidae | Vulpes pallida | 16.715407 | -16.156510 | L2 |  |
| 13857 | Carnivora | Canidae | Vulpes pallida | 17.154127 | -12.335823 | L6 |  |
| 12904 | Carnivora | Canidae | Vulpes pallida | 16.347448 | -16.462917 | M2 |  |
| 7922 | Carnivora | Canidae | Vulpes pallida | 16.155158 | -13.544458 | M5 |  |
| 7918 | Carnivora | Canidae | Vulpes pallida | 16.155158 | -13.544458 | M5 |  |
| 7914 | Carnivora | Canidae | Vulpes pallida | 16.155158 | -13.544458 | M5 |  |
| BIB_0351 | Carnivora | Canidae | Vulpes rueppellii | 20.136667 | -16.168333 | I2 | Gowthorpe 1993 |
| BIB_0350 | Carnivora | Canidae | Vulpes rueppellii | 21.068914 | -16.992276 | H2 | Aulagnier et al 2017 |
| BIB_0349 | Carnivora | Canidae | Vulpes rueppellii | 20.070100 | -16.200159 | I2 | Mahé 1985 |
| BIB_0348 | Carnivora | Canidae | Vulpes rueppellii | 20.101608 | -16.170559 | I2 | Gowthorpe 1993 |
| BIB_0347 | Carnivora | Canidae | Vulpes rueppellii | 20.490000 | -13.076667 | H6 | Dekeyser and Villiers 1956; Le Berre 1990 |
| BIB_0346 | Carnivora | Canidae | Vulpes rueppellii | 19.751600 | -14.428100 | I4 | Jullien and Petter 1970 |
| BIB_0337 | Carnivora | Canidae | Vulpes rueppellii | 20.127838 | -16.259999 | I2 | Mahé 1985 |
| BIB_0336 | Carnivora | Canidae | Vulpes rueppellii | 20.585660 | -16.236789 | H2 | Hughes et al 1992 |
| BIB_0335 | Carnivora | Canidae | Vulpes rueppellii | 19.921450 | -16.187650 | I2 | Flanagan 2006 |
| BIB_0334 | Carnivora | Canidae | Vulpes rueppellii | 19.632933 | -16.058800 | I3 | Flanagan 2006 |
| BIB_0333 | Carnivora | Canidae | Vulpes rueppellii | 19.588167 | -16.096883 | I3 | Flanagan 2006 |
| 19922 | Carnivora | Canidae | Vulpes rueppellii | 19.076373 | -15.038093 | J4 |  |
| 15831 | Carnivora | Canidae | Vulpes rueppellii | 20.907042 | -13.199035 | H6 |  |
| 15442 | Carnivora | Canidae | Vulpes rueppellii | 20.557098 | -12.632996 | H6 |  |
| 13667 | Carnivora | Canidae | Vulpes rueppellii | 23.871750 | -11.226817 | D8 |  |
| BIB_0382 | Carnivora | Canidae | Vulpes zerda | 17.439796 | -16.062595 | L3 | Granjon et al 1997 |
| BIB_0380 | Carnivora | Canidae | Vulpes zerda | 20.597688 | -16.070054 | H3 | Mahé 1985 |
| BIB_0379 | Carnivora | Canidae | Vulpes zerda | 20.595355 | -16.110624 | H2 | Mahé 1985 |
| BIB_0378 | Carnivora | Canidae | Vulpes zerda | 20.500097 | -16.205409 | H2 | Mahé 1985 |
| BIB_0377 | Carnivora | Canidae | Vulpes zerda | 21.000000 | -9.500000 | G9 | Monod 1964 |
| BIB_0376 | Carnivora | Canidae | Vulpes zerda | 16.710000 | -16.120000 | L3 | Dia 2004; Gueye and Dia 2004 |
| BIB_0375 | Carnivora | Canidae | Vulpes zerda | 16.222172 | -16.490439 | M2 | Granjon et al 1997 |
| BIB_0374 | Carnivora | Canidae | Vulpes zerda | 16.607000 | -16.439323 | M2 | Lafont and Measson 2001 |
| BIB_0373 | Carnivora | Canidae | Vulpes zerda | 16.607000 | -16.439323 | M2 | Granjon et al 1997 |
| BIB_0372 | Carnivora | Canidae | Vulpes zerda | 20.438333 | -12.370000 | H6 | Dekeyser and Villiers 1956 |
| BIB_0370 | Carnivora | Canidae | Vulpes zerda | 19.969233 | -16.199783 | I2 | Flanagan 2006 |
| BIB_0369 | Carnivora | Canidae | Vulpes zerda | 19.941050 | -16.151967 | I2 | Flanagan 2006 |
| BIB_0368 | Carnivora | Canidae | Vulpes zerda | 19.911250 | -16.163150 | I2 | Flanagan 2006 |
| BIB_0367 | Carnivora | Canidae | Vulpes zerda | 19.901433 | -16.246967 | I2 | Flanagan 2006 |
| BIB_0366 | Carnivora | Canidae | Vulpes zerda | 19.807200 | -16.021100 | I3 | Flanagan 2006 |
| BIB_0365 | Carnivora | Canidae | Vulpes zerda | 19.805733 | -16.000833 | I3 | Flanagan 2006 |
| BIB_0364 | Carnivora | Canidae | Vulpes zerda | 19.743967 | -16.222300 | I2 | Padial et al 2002 |
| BIB_0363 | Carnivora | Canidae | Vulpes zerda | 19.612600 | -16.090400 | I3 | Flanagan 2006 |
| BIB_0362 | Carnivora | Canidae | Vulpes zerda | 19.515933 | -16.258767 | I2 | Flanagan 2006 |
| BIB_0361 | Carnivora | Canidae | Vulpes zerda | 19.504950 | -16.409683 | I2 | Flanagan 2006 |
| BIB_0360 | Carnivora | Canidae | Vulpes zerda | 19.484600 | -16.247750 | I2 | Flanagan 2006 |
| BIB_0359 | Carnivora | Canidae | Vulpes zerda | 19.430833 | -16.331617 | I2 | Flanagan 2006 |
| BIB_0358 | Carnivora | Canidae | Vulpes zerda | 19.387883 | -16.365133 | I2 | Flanagan 2006 |
| BIB_0357 | Carnivora | Canidae | Vulpes zerda | 19.886893 | -15.999371 | I3 | Mahé 1985 |
| BIB_0356 | Carnivora | Canidae | Vulpes zerda | 19.799043 | -16.140820 | I2 | Mahé 1985 |
| BIB_0355 | Carnivora | Canidae | Vulpes zerda | 19.670634 | -16.275883 | I2 | Mahé 1985 |
| BIB_0354 | Carnivora | Canidae | Vulpes zerda | 19.455606 | -16.245346 | I2 | Mahé 1985 |
| BIB_0353 | Carnivora | Canidae | Vulpes zerda | 19.414531 | -16.351599 | I2 | Mahé 1985 |
| BIB_0352 | Carnivora | Canidae | Vulpes zerda | 19.367019 | -16.407314 | I2 | Mahé 1985 |
| 18849 | Carnivora | Canidae | Vulpes zerda | 17.253462 | -11.408055 | L7 |  |
| 15934 | Carnivora | Canidae | Vulpes zerda | 18.600288 | -11.558017 | J7 |  |
| 15871 | Carnivora | Canidae | Vulpes zerda | 20.644302 | -10.407993 | H8 |  |
| 15870 | Carnivora | Canidae | Vulpes zerda | 20.733510 | -10.358735 | H8 |  |
| 15842 | Carnivora | Canidae | Vulpes zerda | 20.652362 | -12.108133 | H7 |  |
| 15833 | Carnivora | Canidae | Vulpes zerda | 20.490177 | -12.318732 | H6 |  |
| 15832 | Carnivora | Canidae | Vulpes zerda | 20.527647 | -13.002570 | H6 |  |
| 13719 | Carnivora | Canidae | Vulpes zerda | 24.795877 | -10.808218 | C8 |  |
| 9685 | Carnivora | Canidae | Vulpes zerda | 17.589240 | -7.445895 | K12 |  |
| BIB_0390 | Carnivora | Felidae | Acinonyx jubatus | 18.540000 | -11.440000 | J7 | Trotignon 1975; Le Berre 1990 |
| BIB_0389 | Carnivora | Felidae | Acinonyx jubatus | 14.775834 | -12.213947 | O7 | Mauritanie 2000 SARL 2001 |
| BIB_0388 | Carnivora | Felidae | Acinonyx jubatus | 18.710000 | -11.550000 | J7 | Campbell et al 2006 |
| BIB_0387 | Carnivora | Felidae | Acinonyx jubatus | 16.400000 | -16.400000 | M2 | Spatz 1930 |
| BIB_0386 | Carnivora | Felidae | Acinonyx jubatus | 18.333333 | -9.083333 | J10 | Holl 1985 |
| BIB_0385 | Carnivora | Felidae | Acinonyx jubatus | 18.450000 | -9.500000 | J9 | Holl 2009 |
| BIB_0384 | Carnivora | Felidae | Acinonyx jubatus | 15.300000 | -12.500000 | N6 | Dia 2004 |
| BIB_0383 | Carnivora | Felidae | Acinonyx jubatus | 20.500000 | -13.250000 | H5 | Dekeyser and Villiers 1956; Le Berre 1990 |
| BIB_0395 | Carnivora | Felidae | Caracal caracal | 16.498611 | -16.378056 | M2 | Hughes et al 1992 |
| BIB_0394 | Carnivora | Felidae | Caracal caracal | 14.775834 | -12.213947 | O7 | Mauritanie 2000 SARL 2001 |
| BIB_0393 | Carnivora | Felidae | Caracal caracal | 16.303340 | -16.401357 | M2 | National Research Council 1981; Hughes et al 1992 |
| BIB_0392 | Carnivora | Felidae | Caracal caracal | 16.607000 | -16.439323 | M2 | Lafont and Measson 2001 |
| BIB_0391 | Carnivora | Felidae | Caracal caracal | 16.607000 | -16.439323 | M2 | Granjon et al 1997 |
| 11090 | Carnivora | Felidae | Caracal caracal | 15.694747 | -11.237802 | M8 |  |
| 9991 | Carnivora | Felidae | Caracal caracal | 18.442815 | -11.387390 | J7 |  |
| BIB_0401 | Carnivora | Felidae | Felis margarita | 21.000000 | -9.500000 | G9 | Lamarche 1980; Le Berre 1990 |
| BIB_0400 | Carnivora | Felidae | Felis margarita | 20.585660 | -16.236789 | H2 | Hughes et al 1992 |
| BIB_0399 | Carnivora | Felidae | Felis margarita | 20.585660 | -16.236789 | H2 | Gowthorpe 1993 |
| BIB_0398 | Carnivora | Felidae | Felis margarita | 19.959883 | -16.194767 | I2 | Flanagan 2006 |
| BIB_0397 | Carnivora | Felidae | Felis margarita | 19.924200 | -16.188667 | I2 | Flanagan 2006 |
| BIB_0396 | Carnivora | Felidae | Felis margarita | 19.536717 | -16.414700 | I2 | Flanagan 2006 |
| 5881 | Carnivora | Felidae | Felis margarita | 21.372827 | -11.910393 | G7 |  |
| BIB_0426 | Carnivora | Felidae | Felis silvestris lybica | 16.858848 | -9.408776 | L10 | Kirsch-Jung and Khtour 2007 |
| BIB_0425 | Carnivora | Felidae | Felis silvestris lybica | 14.775834 | -12.213947 | O7 | Mauritanie 2000 SARL 2001 |
| BIB_0424 | Carnivora | Felidae | Felis silvestris lybica | 16.303340 | -16.401357 | M2 | National Research Council 1981; Ly and Zein 2009 |
| BIB_0423 | Carnivora | Felidae | Felis silvestris lybica | 16.384695 | -9.300555 | M10 | Kirsch-Jung and Khtour 2007 |
| BIB_0422 | Carnivora | Felidae | Felis silvestris lybica | 17.249855 | -10.667613 | L8 | Kirsch-Jung and Khtour 2007 |
| BIB_0421 | Carnivora | Felidae | Felis silvestris lybica | 15.504833 | -9.816833 | N9 | Kirsch-Jung and Khtour 2007 |
| BIB_0420 | Carnivora | Felidae | Felis silvestris lybica | 20.580946 | -13.136361 | H6 | Dekeyser and Villiers 1956; Le Berre 1990 |
| BIB_0419 | Carnivora | Felidae | Felis silvestris lybica | 20.297290 | -13.209960 | H6 | Dekeyser and Villiers 1956; Le Berre 1990 |
| BIB_0417 | Carnivora | Felidae | Felis silvestris lybica | 20.323193 | -13.142101 | H6 | Dekeyser and Villiers 1956; Le Berre 1990 |
| BIB_0416 | Carnivora | Felidae | Felis silvestris lybica | 16.403148 | -9.559860 | M9 | Kirsch-Jung and Khtour 2007 |
| BIB_0415 | Carnivora | Felidae | Felis silvestris lybica | 16.222172 | -16.490439 | M2 | Granjon et al 1997 |
| BIB_0414 | Carnivora | Felidae | Felis silvestris lybica | 16.607000 | -16.439323 | M2 | Lafont and Measson 2001 |
| BIB_0413 | Carnivora | Felidae | Felis silvestris lybica | 16.607000 | -16.439323 | M2 | Granjon et al 1997 |
| BIB_0412 | Carnivora | Felidae | Felis silvestris lybica | 16.540090 | -10.801490 | L8 | Nickel 2003 |
| BIB_0411 | Carnivora | Felidae | Felis silvestris lybica | 16.574737 | -9.593983 | L9 | Kirsch-Jung and Khtour 2007 |
| BIB_0408 | Carnivora | Felidae | Felis silvestris lybica | 20.585660 | -16.236789 | H2 | Hughes et al 1992 |
| BIB_0407 | Carnivora | Felidae | Felis silvestris lybica | 20.585660 | -16.236789 | H2 | Gowthorpe 1993 |
| BIB_0406 | Carnivora | Felidae | Felis silvestris lybica | 19.385300 | -16.427083 | I2 | Flanagan 2006 |
| BIB_0405 | Carnivora | Felidae | Felis silvestris lybica | 17.110283 | -14.001633 | L5 | Lafont and Measson 2001 |
| 20003 | Carnivora | Felidae | Felis silvestris lybica | 20.533300 | -12.750700 | H6 |  |
| 19751 | Carnivora | Felidae | Felis silvestris lybica | 16.543913 | -10.751155 | L8 |  |
| 19714 | Carnivora | Felidae | Felis silvestris lybica | 16.543913 | -10.751155 | L8 |  |
| 12906 | Carnivora | Felidae | Felis silvestris lybica | 16.607937 | -16.313605 | M2 |  |
| 12899 | Carnivora | Felidae | Felis silvestris lybica | 17.152482 | -12.199115 | L7 |  |
| 12893 | Carnivora | Felidae | Felis silvestris lybica | 16.153808 | -10.752278 | M8 |  |
| 12892 | Carnivora | Felidae | Felis silvestris lybica | 15.970850 | -10.901560 | M8 |  |
| 12797 | Carnivora | Felidae | Felis silvestris lybica | 16.588238 | -15.507783 | M3 |  |
| 12796 | Carnivora | Felidae | Felis silvestris lybica | 16.588238 | -15.507783 | M3 |  |
| 12491 | Carnivora | Felidae | Felis silvestris lybica | 15.857488 | -10.981663 | M8 |  |
| 12054 | Carnivora | Felidae | Felis silvestris lybica | 19.624178 | -13.006232 | I6 |  |
| BIB_0431 | Carnivora | Felidae | Leptailurus serval | 14.775834 | -12.213947 | O7 | Mauritanie 2000 SARL 2001 |
| BIB_0430 | Carnivora | Felidae | Leptailurus serval | 16.303340 | -16.401357 | M2 | National Research Council 1981 |
| BIB_0429 | Carnivora | Felidae | Leptailurus serval | 16.120000 | -13.690000 | M5 | Dia 2004; Gueye and Dia 2004 |
| BIB_0428 | Carnivora | Felidae | Leptailurus serval | 16.400000 | -16.400000 | M2 | Spatz 1930 |
| BIB_0427 | Carnivora | Felidae | Leptailurus serval | 15.300000 | -12.500000 | N6 | Dia 2004 |
| 11641 | Carnivora | Felidae | Leptailurus serval | 16.283965 | -16.422123 | M2 |  |
| 8027 | Carnivora | Felidae | Leptailurus serval | 16.303340 | -16.401357 | M2 |  |
| BIB_0445 | Carnivora | Felidae | Panthera leo | 16.260000 | -13.940000 | M5 | Gueye and Dia 2004 |
| BIB_0444 | Carnivora | Felidae | Panthera leo | 14.775834 | -12.213947 | O7 | Mauritanie 2000 SARL 2001 |
| BIB_0443 | Carnivora | Felidae | Panthera leo | 16.149000 | -10.455900 | M8 | Campbell et al 2006 |
| BIB_0442 | Carnivora | Felidae | Panthera leo | 15.470000 | -12.950000 | N6 | Gueye and Dia 2004 |
| BIB_0441 | Carnivora | Felidae | Panthera leo | 16.710000 | -16.120000 | L3 | Gueye and Dia 2004 |
| BIB_0440 | Carnivora | Felidae | Panthera leo | 16.780000 | -14.920000 | L4 | Dia 2004; Gueye and Dia 2004 |
| BIB_0439 | Carnivora | Felidae | Panthera leo | 17.950000 | -12.250000 | K7 | Tellería 2009 |
| BIB_0438 | Carnivora | Felidae | Panthera leo | 16.830000 | -15.700000 | L3 | Gueye and Dia 2004 |
| BIB_0437 | Carnivora | Felidae | Panthera leo | 16.120000 | -13.690000 | M5 | Gueye and Dia 2004 |
| BIB_0436 | Carnivora | Felidae | Panthera leo | 16.400000 | -16.400000 | M2 | Spatz 1930 |
| BIB_0435 | Carnivora | Felidae | Panthera leo | 16.303340 | -16.401357 | M2 | Ba et al 2002 |
| BIB_0434 | Carnivora | Felidae | Panthera leo | 18.450000 | -9.500000 | J9 | Holl 2009 |
| BIB_0433 | Carnivora | Felidae | Panthera leo | 16.510000 | -14.190000 | M5 | Gueye and Dia 2004 |
| BIB_0432 | Carnivora | Felidae | Panthera leo | 15.300000 | -12.500000 | N6 | Dia 2004; Gueye and Dia 2004 |
| 18591 | Carnivora | Felidae | Panthera leo | 15.476092 | -12.933600 | N6 |  |
| 4652 | Carnivora | Felidae | Panthera leo | 15.500420 | -12.956430 | N6 |  |
| BIB_0462 | Carnivora | Felidae | Panthera pardus | 16.260000 | -13.940000 | M5 | Gueye and Dia 2004 |
| BIB_0461 | Carnivora | Felidae | Panthera pardus | 18.540000 | -11.440000 | J7 | Chudeau 1920; Le Berre 1990 |
| BIB_0460 | Carnivora | Felidae | Panthera pardus | 14.775834 | -12.213947 | O7 | Mauritanie 2000 SARL 2001 |
| BIB_0459 | Carnivora | Felidae | Panthera pardus | 15.470000 | -12.950000 | N6 | Gueye and Dia 2004 |
| BIB_0458 | Carnivora | Felidae | Panthera pardus | 16.620000 | -14.290000 | L4 | Jacobson et al 2016 |
| BIB_0457 | Carnivora | Felidae | Panthera pardus | 16.303340 | -16.401357 | M2 | Jacobson et al 2016 |
| BIB_0456 | Carnivora | Felidae | Panthera pardus | 16.300000 | -10.250000 | M9 | Jacobson et al 2016 |
| BIB_0455 | Carnivora | Felidae | Panthera pardus | 15.767000 | -7.975000 | M11 | Jacobson et al 2016 |
| BIB_0454 | Carnivora | Felidae | Panthera pardus | 15.500000 | -12.956000 | N6 | Jacobson et al 2016 |
| BIB_0453 | Carnivora | Felidae | Panthera pardus | 14.890000 | -12.420000 | N6 | Jacobson et al 2016 |
| BIB_0452 | Carnivora | Felidae | Panthera pardus | 16.780000 | -14.920000 | L4 | Gueye and Dia 2004 |
| BIB_0451 | Carnivora | Felidae | Panthera pardus | 17.950000 | -12.250000 | K7 | Tellería 2009 |
| BIB_0450 | Carnivora | Felidae | Panthera pardus | 16.830000 | -15.700000 | L3 | Gueye and Dia 2004 |
| BIB_0449 | Carnivora | Felidae | Panthera pardus | 16.120000 | -13.690000 | M5 | Dia 2004; Gueye and Dia 2004 |
| BIB_0448 | Carnivora | Felidae | Panthera pardus | 16.510000 | -14.190000 | M5 | Gueye and Dia 2004 |
| BIB_0447 | Carnivora | Felidae | Panthera pardus | 15.300000 | -12.500000 | N6 | Gueye and Dia 2004 |
| BIB_0446 | Carnivora | Felidae | Panthera pardus | 20.500000 | -13.250000 | H5 | Dekeyser and Villiers 1956; Le Berre 1990 |
| 20004 | Carnivora | Felidae | Panthera pardus | 21.526448 | -12.864365 | G6 |  |
| 18750 | Carnivora | Felidae | Panthera pardus | 15.288200 | -10.812240 | N8 |  |
| 18592 | Carnivora | Felidae | Panthera pardus | 15.476092 | -12.933600 | N6 |  |
| 12464 | Carnivora | Felidae | Panthera pardus | 15.845405 | -11.205873 | M8 |  |
| 3547 | Carnivora | Felidae | Panthera pardus | 19.558323 | -16.401927 | I2 | Jacobson et al 2016 |
| BIB_0466 | Carnivora | Herpestidae | Atilax paludinosus | 16.620000 | -14.290000 | L4 | Hughes et al 1992 |
| BIB_0465 | Carnivora | Herpestidae | Atilax paludinosus | 16.303340 | -16.401357 | M2 | National Research Council 1981; Hughes et al 1992 |
| BIB_0464 | Carnivora | Herpestidae | Atilax paludinosus | 15.500000 | -12.956000 | N6 | Hughes et al 1992 |
| BIB_0463 | Carnivora | Herpestidae | Atilax paludinosus | 16.850556 | -15.303889 | L3 | Hughes et al 1992 |
| BIB_0469 | Carnivora | Herpestidae | Herpestes ichneumon | 16.498611 | -16.378056 | M2 | Hughes et al 1992 |
| BIB_0468 | Carnivora | Herpestidae | Herpestes ichneumon | 16.303340 | -16.401357 | M2 | National Research Council 1981; Hughes et al 1992 |
| BIB_0467 | Carnivora | Herpestidae | Herpestes ichneumon | 20.500000 | -13.250000 | H5 | Heim de Balsac 1948; Le Berre 1990 |
| 15991 | Carnivora | Herpestidae | Herpestes ichneumon | 16.082252 | -11.891343 | M7 |  |
| 11249 | Carnivora | Herpestidae | Herpestes ichneumon | 16.054682 | -10.537382 | M8 |  |
| BIB_0472 | Carnivora | Herpestidae | Herpestes sanguineus | 16.303340 | -16.401357 | M2 | National Research Council 1981 |
| BIB_0471 | Carnivora | Herpestidae | Herpestes sanguineus | 20.580946 | -13.136361 | H6 | Dekeyser and Villiers 1956 |
| BIB_0470 | Carnivora | Herpestidae | Herpestes sanguineus | 20.323193 | -13.142101 | H6 | Dekeyser and Villiers 1956 |
| 16030 | Carnivora | Herpestidae | Herpestes sanguineus | 15.661542 | -11.397000 | M7 |  |
| 13880 | Carnivora | Herpestidae | Herpestes sanguineus | 16.309605 | -16.393887 | M2 |  |
| 11272 | Carnivora | Herpestidae | Herpestes sanguineus | 16.053925 | -10.590483 | M8 |  |
| 11227 | Carnivora | Herpestidae | Herpestes sanguineus | 15.798355 | -10.805377 | M8 |  |
| BIB_0473 | Carnivora | Herpestidae | Ichneumia albicauda | 16.303340 | -16.401357 | M2 | National Research Council 1981 |
| 19000 | Carnivora | Herpestidae | Ichneumia albicauda | 15.562402 | -11.244473 | N8 |  |
| 15995 | Carnivora | Herpestidae | Ichneumia albicauda | 16.002553 | -11.871748 | M7 |  |
| 12907 | Carnivora | Herpestidae | Ichneumia albicauda | 16.301168 | -10.853177 | M8 |  |
| 12897 | Carnivora | Herpestidae | Ichneumia albicauda | 17.329165 | -10.708572 | L8 |  |
| 10773 | Carnivora | Herpestidae | Ichneumia albicauda | 16.624455 | -15.206538 | L3 |  |
| 10770 | Carnivora | Herpestidae | Ichneumia albicauda | 16.608507 | -15.329743 | L3 |  |
| 7419 | Carnivora | Herpestidae | Ichneumia albicauda | 16.548878 | -15.720755 | M3 |  |
| BIB_0481 | Carnivora | Hyaenidae | Crocuta crocuta | 14.775834 | -12.213947 | O7 | Mauritanie 2000 SARL 2001 |
| BIB_0480 | Carnivora | Hyaenidae | Crocuta crocuta | 16.149000 | -10.455900 | M8 | Campbell et al 2006 |
| BIB_0479 | Carnivora | Hyaenidae | Crocuta crocuta | 15.470000 | -12.950000 | N6 | Dia 2004; Gueye and Dia 2004 |
| BIB_0478 | Carnivora | Hyaenidae | Crocuta crocuta | 16.490000 | -7.150000 | L12 | Mills and Hofer 1998 |
| BIB_0477 | Carnivora | Hyaenidae | Crocuta crocuta | 16.710000 | -16.120000 | L3 | Dia 2004 |
| BIB_0476 | Carnivora | Hyaenidae | Crocuta crocuta | 16.120000 | -13.690000 | M5 | Dia 2004; Gueye and Dia 2004 |
| BIB_0475 | Carnivora | Hyaenidae | Crocuta crocuta | 15.300000 | -12.500000 | N6 | Gueye and Dia 2004 |
| BIB_0474 | Carnivora | Hyaenidae | Crocuta crocuta | 20.500000 | -13.250000 | H5 | Chudeau 1920; Le Berre 1990 |
| 18749 | Carnivora | Hyaenidae | Crocuta crocuta | 15.288200 | -10.812240 | N8 |  |
| 18732 | Carnivora | Hyaenidae | Crocuta crocuta | 15.347000 | -11.034000 | N8 |  |
| 18608 | Carnivora | Hyaenidae | Crocuta crocuta | 15.010340 | -12.481578 | N6 |  |
| BIB_0502 | Carnivora | Hyaenidae | Hyaena hyaena | 16.260000 | -13.940000 | M5 | Dia 2004 |
| BIB_0501 | Carnivora | Hyaenidae | Hyaena hyaena | 16.303340 | -16.401357 | M2 | National Research Council 1981 |
| BIB_0500 | Carnivora | Hyaenidae | Hyaena hyaena | 18.870000 | -16.160000 | J2 | Gowthorpe 1993 |
| BIB_0499 | Carnivora | Hyaenidae | Hyaena hyaena | 21.068914 | -16.992276 | H2 | Aulagnier et al 2017 |
| BIB_0498 | Carnivora | Hyaenidae | Hyaena hyaena | 15.470000 | -12.950000 | N6 | Dia 2004; Gueye and Dia 2004 |
| BIB_0497 | Carnivora | Hyaenidae | Hyaena hyaena | 16.720000 | -7.350000 | L12 | Mills and Hofer 1998 |
| BIB_0496 | Carnivora | Hyaenidae | Hyaena hyaena | 20.580946 | -13.136361 | H6 | Dekeyser and Villiers 1956 |
| BIB_0495 | Carnivora | Hyaenidae | Hyaena hyaena | 16.710000 | -16.120000 | L3 | Dia 2004 |
| BIB_0494 | Carnivora | Hyaenidae | Hyaena hyaena | 19.880000 | -10.170000 | I9 | Monod 1961 |
| BIB_0493 | Carnivora | Hyaenidae | Hyaena hyaena | 16.780000 | -14.920000 | L4 | Dia 2004; Gueye and Dia 2004 |
| BIB_0492 | Carnivora | Hyaenidae | Hyaena hyaena | 20.303600 | -13.197283 | H6 | Dekeyser and Villiers 1956 |
| BIB_0491 | Carnivora | Hyaenidae | Hyaena hyaena | 20.323193 | -13.142101 | H6 | Dekeyser and Villiers 1956 |
| BIB_0490 | Carnivora | Hyaenidae | Hyaena hyaena | 17.950000 | -12.250000 | K7 | Tellería 2009 |
| BIB_0489 | Carnivora | Hyaenidae | Hyaena hyaena | 20.050000 | -16.217000 | I2 | Granjon et al 1997 |
| BIB_0488 | Carnivora | Hyaenidae | Hyaena hyaena | 16.120000 | -13.690000 | M5 | Dia 2004; Gueye and Dia 2004 |
| BIB_0487 | Carnivora | Hyaenidae | Hyaena hyaena | 16.425376 | -9.630707 | M9 | Kirsch-Jung and Khtour 2007 |
| BIB_0486 | Carnivora | Hyaenidae | Hyaena hyaena | 20.687000 | -16.674000 | H2 | Granjon et al 1997 |
| BIB_0485 | Carnivora | Hyaenidae | Hyaena hyaena | 19.613400 | -16.326667 | I2 | Flanagan 2006 |
| BIB_0484 | Carnivora | Hyaenidae | Hyaena hyaena | 15.300000 | -12.500000 | N6 | Dia 2004; Gueye and Dia 2004 |
| BIB_0483 | Carnivora | Hyaenidae | Hyaena hyaena | 20.500000 | -13.250000 | H5 | Mills and Hofer 1998 |
| BIB_0482 | Carnivora | Hyaenidae | Hyaena hyaena | 20.500000 | -13.250000 | H5 | Chudeau 1920; Le Berre 1990 |
| 13801 | Carnivora | Hyaenidae | Hyaena hyaena | 19.654210 | -12.664588 | I6 |  |
| 11166 | Carnivora | Hyaenidae | Hyaena hyaena | 15.627462 | -10.878050 | N8 |  |
| 11146 | Carnivora | Hyaenidae | Hyaena hyaena | 15.551130 | -11.087958 | N8 |  |
| 11089 | Carnivora | Hyaenidae | Hyaena hyaena | 15.694747 | -11.237802 | M8 |  |
| BIB_0506 | Carnivora | Mustelidae | Aonyx capensis | 16.620000 | -14.290000 | L4 | Hughes et al 1992 |
| BIB_0505 | Carnivora | Mustelidae | Aonyx capensis | 16.303340 | -16.401357 | M2 | National Research Council 1981; Hughes et al 1992 |
| BIB_0504 | Carnivora | Mustelidae | Aonyx capensis | 15.500000 | -12.956000 | N6 | Hughes et al 1992 |
| BIB_0503 | Carnivora | Mustelidae | Aonyx capensis | 16.850556 | -15.303889 | L3 | Hughes et al 1992 |
| BIB_0513 | Carnivora | Mustelidae | Ictonyx libyca | 20.303600 | -13.197283 | H6 | Dekeyser and Villiers 1956; Le Berre 1990 |
| 15926 | Carnivora | Mustelidae | Ictonyx libyca | 19.496963 | -12.277607 | I7 |  |
| 11435 | Carnivora | Mustelidae | Ictonyx libyca | 16.543543 | -9.956927 | L9 |  |
| 9841 | Carnivora | Mustelidae | Ictonyx libyca | 18.367125 | -11.636903 | J7 |  |
| 9755 | Carnivora | Mustelidae | Ictonyx libyca | 18.401312 | -8.573102 | J10 |  |
| 6406 | Carnivora | Mustelidae | Ictonyx libyca | 20.420480 | -16.276895 | H2 |  |
| 6003 | Carnivora | Mustelidae | Ictonyx libyca | 17.424440 | -13.357260 | L5 |  |
| 5760 | Carnivora | Mustelidae | Ictonyx libyca | 21.197600 | -14.222085 | G4 |  |
| 5759 | Carnivora | Mustelidae | Ictonyx libyca | 21.197600 | -14.222085 | G4 |  |
| 2738 | Carnivora | Mustelidae | Ictonyx libyca | 19.773410 | -16.189340 | I2 |  |
| BIB_0514 | Carnivora | Mustelidae | Ictonyx striatus | 16.303340 | -16.401357 | M2 | National Research Council 1981 |
| 18983 | Carnivora | Mustelidae | Ictonyx striatus | 14.799752 | -12.290222 | N7 |  |
| BIB_0534 | Carnivora | Mustelidae | Mellivora capensis | 16.260000 | -13.940000 | M5 | Dia 2004 |
| BIB_0533 | Carnivora | Mustelidae | Mellivora capensis | 16.303340 | -16.401357 | M2 | National Research Council 1981 |
| BIB_0532 | Carnivora | Mustelidae | Mellivora capensis | 16.384695 | -9.300555 | M10 | Kirsch-Jung and Khtour 2007 |
| BIB_0531 | Carnivora | Mustelidae | Mellivora capensis | 17.249855 | -10.667613 | L8 | Kirsch-Jung and Khtour 2007 |
| BIB_0530 | Carnivora | Mustelidae | Mellivora capensis | 15.504833 | -9.816833 | N9 | Kirsch-Jung and Khtour 2007 |
| BIB_0528 | Carnivora | Mustelidae | Mellivora capensis | 15.470000 | -12.950000 | N6 | Dia 2004 |
| BIB_0527 | Carnivora | Mustelidae | Mellivora capensis | 16.710000 | -16.120000 | L3 | Dia 2004; Gueye and Dia 2004 |
| BIB_0526 | Carnivora | Mustelidae | Mellivora capensis | 19.880000 | -10.170000 | I9 | Monod 1961 |
| BIB_0525 | Carnivora | Mustelidae | Mellivora capensis | 19.392494 | -14.411847 | I4 | Valverde 1957 |
| BIB_0524 | Carnivora | Mustelidae | Mellivora capensis | 16.403148 | -9.559860 | M9 | Kirsch-Jung and Khtour 2007 |
| BIB_0523 | Carnivora | Mustelidae | Mellivora capensis | 16.830000 | -15.700000 | L3 | Dia 2004; Gueye and Dia 2004 |
| BIB_0522 | Carnivora | Mustelidae | Mellivora capensis | 16.120000 | -13.690000 | M5 | Dia 2004 |
| BIB_0521 | Carnivora | Mustelidae | Mellivora capensis | 16.303340 | -16.401357 | M2 | Ly and Zein 2009 |
| BIB_0520 | Carnivora | Mustelidae | Mellivora capensis | 18.333333 | -9.083333 | J10 | Holl 1985 |
| BIB_0519 | Carnivora | Mustelidae | Mellivora capensis | 18.450000 | -9.500000 | J9 | Holl 2009 |
| BIB_0518 | Carnivora | Mustelidae | Mellivora capensis | 16.222172 | -16.490439 | M2 | Granjon et al 1997 |
| BIB_0517 | Carnivora | Mustelidae | Mellivora capensis | 16.607000 | -16.439323 | M2 | Lafont and Measson 2001 |
| BIB_0516 | Carnivora | Mustelidae | Mellivora capensis | 25.220000 | -11.577000 | C7 | Chudeau 1920; Le Berre 1990 |
| BIB_0515 | Carnivora | Mustelidae | Mellivora capensis | 15.300000 | -12.500000 | N6 | Dia 2004; Gueye and Dia 2004 |
| 16039 | Carnivora | Mustelidae | Mellivora capensis | 15.527658 | -11.290263 | N8 |  |
| BIB_0538 | Carnivora | Viverridae | Civettictis civetta | 16.631944 | -10.386944 | L9 | Padial and Ibáñez 2005 |
| BIB_0537 | Carnivora | Viverridae | Civettictis civetta | 16.303340 | -16.401357 | M2 | National Research Council 1981 |
| BIB_0536 | Carnivora | Viverridae | Civettictis civetta | 16.540090 | -10.801490 | L8 | Nickel 2003 |
| BIB_0535 | Carnivora | Viverridae | Civettictis civetta | 16.661389 | -9.623056 | L9 | Padial and Ibáñez 2005 |
| 19010 | Carnivora | Viverridae | Civettictis civetta | 16.544140 | -10.750440 | L8 |  |
| 19007 | Carnivora | Viverridae | Civettictis civetta | 16.544140 | -10.750440 | L8 |  |
| 12900 | Carnivora | Viverridae | Civettictis civetta | 17.152482 | -12.199115 | L7 |  |
| 12898 | Carnivora | Viverridae | Civettictis civetta | 16.923777 | -10.593842 | L8 |  |
| 12800 | Carnivora | Viverridae | Civettictis civetta | 16.347458 | -16.343328 | M2 |  |
| 12799 | Carnivora | Viverridae | Civettictis civetta | 16.360420 | -16.341897 | M2 |  |
| 11427 | Carnivora | Viverridae | Civettictis civetta | 15.551130 | -11.087958 | N8 |  |
| 10769 | Carnivora | Viverridae | Civettictis civetta | 16.610713 | -15.343240 | L3 |  |
| 8026 | Carnivora | Viverridae | Civettictis civetta | 16.303340 | -16.401357 | M2 |  |
| 7919 | Carnivora | Viverridae | Civettictis civetta | 16.163073 | -11.748173 | M7 |  |
| BIB_0550 | Carnivora | Viverridae | Genetta genetta | 16.303340 | -16.401357 | M2 | National Research Council 1981 |
| BIB_0549 | Carnivora | Viverridae | Genetta genetta | 16.538033 | -10.741550 | L8 | Nickel 2003 |
| BIB_0548 | Carnivora | Viverridae | Genetta genetta | 20.297290 | -13.209960 | H6 | Dekeyser and Villiers 1956; Le Berre 1990 |
| BIB_0547 | Carnivora | Viverridae | Genetta genetta | 18.333333 | -9.083333 | J10 | Holl 1985 |
| BIB_0546 | Carnivora | Viverridae | Genetta genetta | 18.450000 | -9.500000 | J9 | Holl 2009 |
| BIB_0545 | Carnivora | Viverridae | Genetta genetta | 16.607000 | -16.439323 | M2 | Lafont and Measson 2001 |
| BIB_0544 | Carnivora | Viverridae | Genetta genetta | 16.607000 | -16.439323 | M2 | Granjon et al 1997 |
| BIB_0543 | Carnivora | Viverridae | Genetta genetta | 16.540090 | -10.801490 | L8 | Nickel 2003 |
| 19644 | Carnivora | Viverridae | Genetta genetta | 16.279653 | -16.515098 | M2 |  |
| 19005 | Carnivora | Viverridae | Genetta genetta | 15.211420 | -10.886612 | N8 |  |
| 19002 | Carnivora | Viverridae | Genetta genetta | 15.562402 | -11.244473 | N8 |  |
| 18993 | Carnivora | Viverridae | Genetta genetta | 15.778737 | -11.356423 | M8 |  |
| 18986 | Carnivora | Viverridae | Genetta genetta | 15.957078 | -12.009859 | M7 |  |
| 18979 | Carnivora | Viverridae | Genetta genetta | 15.010340 | -12.481578 | N6 |  |
| 18976 | Carnivora | Viverridae | Genetta genetta | 15.476092 | -12.933600 | N6 |  |
| 18973 | Carnivora | Viverridae | Genetta genetta | 16.408688 | -16.470562 | M2 |  |
| 15940 | Carnivora | Viverridae | Genetta genetta | 17.887298 | -12.110844 | K7 |  |
| 12905 | Carnivora | Viverridae | Genetta genetta | 16.607937 | -16.313605 | M2 |  |
| 12903 | Carnivora | Viverridae | Genetta genetta | 16.347448 | -16.462917 | M2 |  |
| 12895 | Carnivora | Viverridae | Genetta genetta | 17.329165 | -10.708572 | L8 |  |
| 12894 | Carnivora | Viverridae | Genetta genetta | 16.008648 | -10.929053 | M8 |  |
| 12819 | Carnivora | Viverridae | Genetta genetta | 16.401183 | -16.415468 | M2 |  |
| 12798 | Carnivora | Viverridae | Genetta genetta | 16.514258 | -16.282980 | M2 |  |
| 6002 | Carnivora | Viverridae | Genetta genetta | 17.377328 | -13.495870 | L5 |  |
| BIB_0552 | Chiroptera | Emballonuridae | Taphozous nudiventris | 16.661020 | -9.616550 | L9 | GBIF.org 2021 |
| BIB_0551 | Chiroptera | Emballonuridae | Taphozous nudiventris | 16.661020 | -9.616550 | L9 | GBIF.org 2021 |
| BIB_0555 | Chiroptera | Emballonuridae | Taphozous perforatus | 16.303340 | -16.401357 | M2 | National Research Council 1981 |
| BIB_0553 | Chiroptera | Emballonuridae | Taphozous perforatus | 17.048056 | -13.920833 | L5 | ACR 2020 |
| BIB_0598 | Chiroptera | Hipposideridae | Asellia tridens | 20.427222 | -13.065000 | H6 | ACR 2020 |
| 7063 | Chiroptera | Hipposideridae | Asellia tridens | 21.487200 | -11.336028 | G7 |  |
| 7062 | Chiroptera | Hipposideridae | Asellia tridens | 21.487200 | -11.336028 | G7 |  |
| BIB_0618 | Chiroptera | Hipposideridae | Hipposideros cf. caffer | 16.303340 | -16.401357 | M2 | National Research Council 1981 |
| BIB_0617 | Chiroptera | Hipposideridae | Hipposideros cf. caffer | 16.516667 | -15.816667 | M3 | Qumseyeh and Schütter 1981 |
| BIB_0626 | Chiroptera | Hipposideridae | Hipposideros tephrus | 20.252804 | -13.088188 | H6 | Allegrini et al 2011; ACR 2020 |
| BIB_0621 | Chiroptera | Hipposideridae | Hipposideros tephrus | 17.546667 | -14.694444 | K4 | ACR 2020 |
| BIB_0731 | Chiroptera | Molossidae | Mops condylurus | 16.303340 | -16.401357 | M2 | National Research Council 1981 |
| BIB_0627 | Chiroptera | Molossidae | Mops condylurus | 16.512550 | -15.804920 | M3 | GBIF.org 2021 |
| BIB_0732 | Chiroptera | Molossidae | Tadarida aegyptiaca | 16.538033 | -10.741550 | L8 | Nickel 2003 |
| 6222 | Chiroptera | Nycteridae | Nycteris hispida | 15.490148 | -12.940880 | N6 |  |
| 6221 | Chiroptera | Nycteridae | Nycteris hispida | 15.490148 | -12.940880 | N6 |  |
| BIB_0912 | Chiroptera | Nycteridae | Nycteris macrotis | 16.512550 | -15.804920 | M3 | GBIF.org 2021 |
| BIB_0911 | Chiroptera | Nycteridae | Nycteris macrotis | 16.512550 | -15.804920 | M3 | GBIF.org 2021 |
| BIB_0910 | Chiroptera | Nycteridae | Nycteris macrotis | 16.148611 | -13.516111 | M5 | ACR 2020 |
| BIB_0915 | Chiroptera | Nycteridae | Nycteris thebaica | 16.512550 | -15.804920 | M3 | GBIF.org 2021 |
| BIB_0914 | Chiroptera | Nycteridae | Nycteris thebaica | 16.550000 | -15.766667 | M3 | GBIF.org 2021 |
| BIB_0913 | Chiroptera | Nycteridae | Nycteris thebaica | 16.550000 | -15.766667 | M3 | GBIF.org 2021 |
| BIB_0935 | Chiroptera | Pteropodidae | Eidolon helvum | 16.303340 | -16.401357 | M2 | National Research Council 1981 |
| BIB_0919 | Chiroptera | Pteropodidae | Eidolon helvum | 18.115564 | -16.015721 | K3 | Granjon et al 1997 |
| BIB_0918 | Chiroptera | Pteropodidae | Eidolon helvum | 18.083000 | -15.983000 | K3 | Cosson et al 1996; ACR 2020 |
| BIB_0917 | Chiroptera | Pteropodidae | Eidolon helvum | 16.222172 | -16.490439 | M2 | Granjon et al 1997 |
| BIB_0916 | Chiroptera | Pteropodidae | Eidolon helvum | 16.607000 | -16.439323 | M2 | Granjon et al 1997 |
| 18869 | Chiroptera | Pteropodidae | Eidolon helvum | 16.567450 | -11.533010 | L7 |  |
| 18654 | Chiroptera | Pteropodidae | Eidolon helvum | 15.170145 | -12.190143 | N7 |  |
| 18640 | Chiroptera | Pteropodidae | Eidolon helvum | 14.799752 | -12.290222 | N7 |  |
| 18607 | Chiroptera | Pteropodidae | Eidolon helvum | 15.010340 | -12.481578 | N6 |  |
| 18594 | Chiroptera | Pteropodidae | Eidolon helvum | 15.476092 | -12.933600 | N6 |  |
| BIB_0936 | Chiroptera | Rhinolophidae | Rhinolophus fumigatus | 16.303340 | -16.401357 | M2 | National Research Council 1981 |
| BIB_0937 | Chiroptera | Rhinolophidae | Rhinolophus landeri | 17.546667 | -14.694444 | K4 | ACR 2020 |
| BIB_0966 | Chiroptera | Rhinopomatidae | Rhinopoma cystops | 19.729931 | -12.891111 | I6 | Allegrini et al 2011 |
| BIB_0965 | Chiroptera | Rhinopomatidae | Rhinopoma cystops | 19.751600 | -14.428100 | I4 | ACR 2020 |
| BIB_0964 | Chiroptera | Rhinopomatidae | Rhinopoma cystops | 22.678530 | -12.707130 | F6 | GBIF.org 2021 |
| BIB_0963 | Chiroptera | Rhinopomatidae | Rhinopoma cystops | 22.678530 | -12.707130 | F6 | GBIF.org 2021 |
| BIB_0962 | Chiroptera | Rhinopomatidae | Rhinopoma cystops | 22.678530 | -12.707130 | F6 | GBIF.org 2021 |
| BIB_0961 | Chiroptera | Rhinopomatidae | Rhinopoma cystops | 22.678530 | -12.707130 | F6 | GBIF.org 2021 |
| BIB_0960 | Chiroptera | Rhinopomatidae | Rhinopoma cystops | 22.678530 | -12.707130 | F6 | GBIF.org 2021 |
| BIB_0959 | Chiroptera | Rhinopomatidae | Rhinopoma cystops | 22.678530 | -12.707130 | F6 | GBIF.org 2021 |
| BIB_0958 | Chiroptera | Rhinopomatidae | Rhinopoma cystops | 22.678530 | -12.707130 | F6 | GBIF.org 2021 |
| BIB_0957 | Chiroptera | Rhinopomatidae | Rhinopoma cystops | 22.678530 | -12.707130 | F6 | GBIF.org 2021 |
| BIB_0956 | Chiroptera | Rhinopomatidae | Rhinopoma cystops | 22.678530 | -12.707130 | F6 | GBIF.org 2021 |
| BIB_0955 | Chiroptera | Rhinopomatidae | Rhinopoma cystops | 22.678530 | -12.707130 | F6 | GBIF.org 2021 |
| BIB_0954 | Chiroptera | Rhinopomatidae | Rhinopoma cystops | 20.465160 | -13.002200 | H6 | GBIF.org 2021 |
| BIB_0953 | Chiroptera | Rhinopomatidae | Rhinopoma cystops | 20.465160 | -13.002200 | H6 | GBIF.org 2021 |
| BIB_0952 | Chiroptera | Rhinopomatidae | Rhinopoma cystops | 20.465160 | -13.002200 | H6 | GBIF.org 2021 |
| BIB_0951 | Chiroptera | Rhinopomatidae | Rhinopoma cystops | 20.465160 | -13.002200 | H6 | GBIF.org 2021 |
| BIB_0950 | Chiroptera | Rhinopomatidae | Rhinopoma cystops | 20.465160 | -13.002200 | H6 | GBIF.org 2021 |
| BIB_0949 | Chiroptera | Rhinopomatidae | Rhinopoma cystops | 20.465160 | -13.002200 | H6 | GBIF.org 2021 |
| BIB_0948 | Chiroptera | Rhinopomatidae | Rhinopoma cystops | 20.465160 | -13.002200 | H6 | GBIF.org 2021 |
| BIB_0947 | Chiroptera | Rhinopomatidae | Rhinopoma cystops | 20.465160 | -13.002200 | H6 | GBIF.org 2021 |
| BIB_0946 | Chiroptera | Rhinopomatidae | Rhinopoma cystops | 20.465160 | -13.002200 | H6 | GBIF.org 2021 |
| BIB_0945 | Chiroptera | Rhinopomatidae | Rhinopoma cystops | 20.465160 | -13.002200 | H6 | GBIF.org 2021 |
| BIB_0944 | Chiroptera | Rhinopomatidae | Rhinopoma cystops | 19.750000 | -14.383333 | I4 | Qumseyeh and Schütter 1981; ACR 2020 |
| BIB_0943 | Chiroptera | Rhinopomatidae | Rhinopoma cystops | 19.750000 | -14.383333 | I4 | GBIF.org 2021 |
| BIB_0942 | Chiroptera | Rhinopomatidae | Rhinopoma cystops | 19.750000 | -14.383333 | I4 | Poulet 1970; ACR 2020; GBIF.org 2021 |
| BIB_0941 | Chiroptera | Rhinopomatidae | Rhinopoma cystops | 19.750000 | -14.383333 | I4 | Poulet 1970; ACR 2020; GBIF.org 2021 |
| BIB_0940 | Chiroptera | Rhinopomatidae | Rhinopoma cystops | 19.750000 | -14.383333 | I4 | Poulet 1970; ACR 2020; GBIF.org 2021 |
| BIB_0939 | Chiroptera | Rhinopomatidae | Rhinopoma cystops | 19.750000 | -14.383333 | I4 | Poulet 1970; ACR 2020; GBIF.org 2021 |
| BIB_0938 | Chiroptera | Rhinopomatidae | Rhinopoma cystops | 19.750000 | -14.383333 | I4 | Poulet 1970; ACR 2020; GBIF.org 2021 |
| 11944 | Chiroptera | Rhinopomatidae | Rhinopoma hardwickii | 17.067572 | -12.260290 | L7 |  |
| 7090 | Chiroptera | Rhinopomatidae | Rhinopoma hardwickii | 21.593960 | -10.601700 | G8 |  |
| 7071 | Chiroptera | Rhinopomatidae | Rhinopoma hardwickii | 20.933333 | -11.616667 | H7 |  |
| BIB_1050 | Chiroptera | Rhinopomatidae | Rhinopoma microphyllum | 17.873611 | -12.331667 | K6 | ACR 2020 |
| BIB_1048 | Chiroptera | Rhinopomatidae | Rhinopoma microphyllum | 19.751600 | -14.428100 | I4 | Jullien and Petter 1970 |
| BIB_1047 | Chiroptera | Rhinopomatidae | Rhinopoma microphyllum | 19.816667 | -14.416667 | I4 | ACR 2020 |
| BIB_1002 | Chiroptera | Rhinopomatidae | Rhinopoma microphyllum | 19.750000 | -14.383333 | I4 | Poulet 1970; ACR 2020 |
| BIB_1090 | Chiroptera | Vespertilionidae | Nycticeinops schlieffeni | 16.510278 | -15.914167 | M3 | ACR 2020 |
| BIB_1087 | Chiroptera | Vespertilionidae | Nycticeinops schlieffeni | 19.733478 | -12.889167 | I6 | Allegrini et al 2011 |
| 11664 | Chiroptera | Vespertilionidae | Nycticeinops schlieffeni | 16.713367 | -16.381220 | L2 |  |
| 9657 | Chiroptera | Vespertilionidae | Nycticeinops schlieffeni | 16.710073 | -7.383727 | L12 |  |
| 9288 | Chiroptera | Vespertilionidae | Nycticeinops schlieffeni | 15.892698 | -11.449035 | M7 |  |
| 9287 | Chiroptera | Vespertilionidae | Nycticeinops schlieffeni | 15.892698 | -11.449035 | M7 |  |
| BIB_1099 | Chiroptera | Vespertilionidae | Pipistrellus rueppellii | 16.303340 | -16.401357 | M2 | National Research Council 1981 |
| BIB_1107 | Erinaceomorpha | Erinaceidae | Atelerix albiventris | 15.933333 | -12.000000 | M7 | Diatta et al 2015 |
| BIB_1106 | Erinaceomorpha | Erinaceidae | Atelerix albiventris | 16.303340 | -16.401357 | M2 | National Research Council 1981 |
| BIB_1105 | Erinaceomorpha | Erinaceidae | Atelerix albiventris | 15.470000 | -12.950000 | N6 | Dia 2004 |
| BIB_1103 | Erinaceomorpha | Erinaceidae | Atelerix albiventris | 16.120000 | -13.690000 | M5 | Dia 2004 |
| BIB_1102 | Erinaceomorpha | Erinaceidae | Atelerix albiventris | 16.510000 | -14.190000 | M5 | Dia 2004 |
| 16130 | Erinaceomorpha | Erinaceidae | Atelerix albiventris | 16.095012 | -12.891092 | M6 |  |
| 12794 | Erinaceomorpha | Erinaceidae | Atelerix albiventris | 16.631098 | -14.219627 | L5 |  |
| BIB_1120 | Erinaceomorpha | Erinaceidae | Paraechinus aethiopicus | 18.716667 | -15.600000 | J3 | Diatta et al 2015 |
| BIB_1119 | Erinaceomorpha | Erinaceidae | Paraechinus aethiopicus | 18.189955 | -16.007304 | K3 | Granjon et al 1997 |
| BIB_1113 | Erinaceomorpha | Erinaceidae | Paraechinus aethiopicus | 20.297290 | -13.209960 | H6 | Dekeyser and Villiers 1956; Le Berre 1990 |
| BIB_1112 | Erinaceomorpha | Erinaceidae | Paraechinus aethiopicus | 16.222172 | -16.490439 | M2 | Granjon et al 1997 |
| BIB_1111 | Erinaceomorpha | Erinaceidae | Paraechinus aethiopicus | 20.687000 | -16.674000 | H2 | Granjon et al 1997 |
| BIB_1108 | Erinaceomorpha | Erinaceidae | Paraechinus aethiopicus | 19.733333 | -14.366667 | I4 | Diatta et al 2015 |
| 18823 | Erinaceomorpha | Erinaceidae | Paraechinus aethiopicus | 17.214982 | -10.893852 | L8 |  |
| 18821 | Erinaceomorpha | Erinaceidae | Paraechinus aethiopicus | 16.696333 | -10.648162 | L8 |  |
| 15960 | Erinaceomorpha | Erinaceidae | Paraechinus aethiopicus | 16.738730 | -12.302762 | L7 |  |
| 15907 | Erinaceomorpha | Erinaceidae | Paraechinus aethiopicus | 20.817940 | -12.172360 | H7 |  |
| 13859 | Erinaceomorpha | Erinaceidae | Paraechinus aethiopicus | 16.815238 | -12.277170 | L7 |  |
| 13707 | Erinaceomorpha | Erinaceidae | Paraechinus aethiopicus | 25.041477 | -11.501800 | C7 |  |
| BIB_1127 | Hyracoidea | Procaviidae | Procavia capensis | 16.538033 | -10.741550 | L8 | Nickel 2003 |
| BIB_1126 | Hyracoidea | Procaviidae | Procavia capensis | 16.425376 | -9.630707 | M9 | Kirsch-Jung and Khtour 2007 |
| BIB_1125 | Hyracoidea | Procaviidae | Procavia capensis | 21.525000 | -12.860600 | G6 | Heim de Balsac 1948; Le Berre 1990 |
| 19681 | Hyracoidea | Procaviidae | Procavia capensis | 16.538033 | -10.741550 | L8 |  |
| 19642 | Hyracoidea | Procaviidae | Procavia capensis | 15.957078 | -12.009859 | M7 |  |
| 18893 | Hyracoidea | Procaviidae | Procavia capensis | 17.232793 | -12.100005 | L7 |  |
| 18844 | Hyracoidea | Procaviidae | Procavia capensis | 17.657525 | -11.391248 | K7 |  |
| 18817 | Hyracoidea | Procaviidae | Procavia capensis | 16.538033 | -10.741550 | L8 |  |
| 16087 | Hyracoidea | Procaviidae | Procavia capensis | 15.707348 | -11.226087 | M8 |  |
| 16085 | Hyracoidea | Procaviidae | Procavia capensis | 15.628413 | -11.233462 | N8 |  |
| 16055 | Hyracoidea | Procaviidae | Procavia capensis | 15.415229 | -11.190639 | N8 |  |
| 15997 | Hyracoidea | Procaviidae | Procavia capensis | 16.002553 | -11.871748 | M7 |  |
| 15849 | Hyracoidea | Procaviidae | Procavia capensis | 20.778198 | -11.871185 | H7 |  |
| 12747 | Hyracoidea | Procaviidae | Procavia capensis | 17.170608 | -10.637773 | L8 |  |
| 12662 | Hyracoidea | Procaviidae | Procavia capensis | 16.579150 | -10.704550 | L8 |  |
| 12579 | Hyracoidea | Procaviidae | Procavia capensis | 16.153808 | -10.752278 | M8 |  |
| 12567 | Hyracoidea | Procaviidae | Procavia capensis | 16.068188 | -10.839677 | M8 |  |
| 12548 | Hyracoidea | Procaviidae | Procavia capensis | 15.970850 | -10.901560 | M8 |  |
| 12537 | Hyracoidea | Procaviidae | Procavia capensis | 15.972653 | -10.896275 | M8 |  |
| 12453 | Hyracoidea | Procaviidae | Procavia capensis | 15.845405 | -11.205873 | M8 |  |
| BIB_1191 | Lagomorpha | Leporidae | Lepus spp. | 16.260000 | -13.940000 | M5 | Dia 2004 |
| BIB_1188 | Lagomorpha | Leporidae | Lepus spp. | 17.439796 | -16.062595 | L3 | Granjon et al 1997 |
| BIB_1187 | Lagomorpha | Leporidae | Lepus spp. | 16.858848 | -9.408776 | L10 | Kirsch-Jung and Khtour 2007 |
| BIB_1186 | Lagomorpha | Leporidae | Lepus spp. | 16.303340 | -16.401357 | M2 | National Research Council 1981 |
| BIB_1185 | Lagomorpha | Leporidae | Lepus spp. | 20.595355 | -16.110624 | H2 | Gowthorpe 1993 |
| BIB_1184 | Lagomorpha | Leporidae | Lepus spp. | 21.000000 | -9.500000 | G9 | Monod 1964 |
| BIB_1181 | Lagomorpha | Leporidae | Lepus spp. | 15.470000 | -12.950000 | N6 | Dia 2004 |
| BIB_1180 | Lagomorpha | Leporidae | Lepus spp. | 20.101608 | -16.170559 | I2 | Gowthorpe 1993 |
| BIB_1179 | Lagomorpha | Leporidae | Lepus spp. | 16.710000 | -16.120000 | L3 | Dia 2004 |
| BIB_1178 | Lagomorpha | Leporidae | Lepus spp. | 19.880000 | -10.170000 | I9 | Monod 1961 |
| BIB_1177 | Lagomorpha | Leporidae | Lepus spp. | 16.398708 | -7.665378 | M11 | Nickel 2003 |
| BIB_1174 | Lagomorpha | Leporidae | Lepus spp. | 16.403148 | -9.559860 | M9 | Kirsch-Jung and Khtour 2007 |
| BIB_1171 | Lagomorpha | Leporidae | Lepus spp. | 16.830000 | -15.700000 | L3 | Dia 2004 |
| BIB_1170 | Lagomorpha | Leporidae | Lepus spp. | 16.120000 | -13.690000 | M5 | Dia 2004 |
| BIB_1169 | Lagomorpha | Leporidae | Lepus spp. | 16.222172 | -16.490439 | M2 | Granjon et al 1997 |
| BIB_1168 | Lagomorpha | Leporidae | Lepus spp. | 16.607000 | -16.439323 | M2 | Granjon et al 1997; Denys et al 2001 |
| BIB_1167 | Lagomorpha | Leporidae | Lepus spp. | 16.540090 | -10.801490 | L8 | Nickel 2003 |
| BIB_1166 | Lagomorpha | Leporidae | Lepus spp. | 16.510000 | -14.190000 | M5 | Dia 2004 |
| BIB_1164 | Lagomorpha | Leporidae | Lepus spp. | 15.300000 | -12.500000 | N6 | Dia 2004 |
| BIB_1158 | Lagomorpha | Leporidae | Lepus spp. | 19.333333 | -16.283333 | I2 | Granjon et al 1997 |
| BIB_1157 | Lagomorpha | Leporidae | Lepus spp. | 19.333333 | -16.283333 | I2 | Gowthorpe 1993 |
| 19904 | Lagomorpha | Leporidae | Lepus spp. | 16.377407 | -16.421867 | M2 |  |
| 19903 | Lagomorpha | Leporidae | Lepus spp. | 16.406685 | -16.443882 | M2 |  |
| 19902 | Lagomorpha | Leporidae | Lepus spp. | 16.386052 | -16.462360 | M2 |  |
| 19901 | Lagomorpha | Leporidae | Lepus spp. | 16.392240 | -16.477313 | M2 |  |
| 19900 | Lagomorpha | Leporidae | Lepus spp. | 16.358050 | -16.411267 | M2 |  |
| 19899 | Lagomorpha | Leporidae | Lepus spp. | 16.419198 | -16.468585 | M2 |  |
| 16143 | Lagomorpha | Leporidae | Lepus spp. | 16.270695 | -16.516517 | M2 |  |
| 15952 | Lagomorpha | Leporidae | Lepus spp. | 17.866665 | -12.377850 | K6 |  |
| 15845 | Lagomorpha | Leporidae | Lepus spp. | 20.759313 | -11.918537 | H7 |  |
| 13710 | Lagomorpha | Leporidae | Lepus spp. | 24.916660 | -11.462185 | C7 |  |
| 13683 | Lagomorpha | Leporidae | Lepus spp. | 24.573660 | -11.386578 | D7 |  |
| 12792 | Lagomorpha | Leporidae | Lepus spp. | 17.570117 | -12.903832 | K6 |  |
| 12259 | Lagomorpha | Leporidae | Lepus spp. | 19.283665 | -16.123750 | J2 |  |
| BIB_1193 | Perissodactyla | Rhinocerotidae | Ceratotherium simum | 18.333333 | -9.083333 | J10 | Holl 1985 |
| BIB_1192 | Perissodactyla | Rhinocerotidae | Ceratotherium simum | 18.450000 | -9.500000 | J9 | Holl 2009 |
| BIB_1204 | Primates | Cercopithecidae | Chlorocebus sabaeus | 14.775834 | -12.213947 | O7 | Mauritanie 2000 SARL 2001 |
| BIB_1203 | Primates | Cercopithecidae | Chlorocebus sabaeus | 15.470000 | -12.950000 | N6 | Dia 2004; Gueye and Dia 2004 |
| BIB_1202 | Primates | Cercopithecidae | Chlorocebus sabaeus | 16.710000 | -16.120000 | L3 | Gueye and Dia 2004 |
| BIB_1201 | Primates | Cercopithecidae | Chlorocebus sabaeus | 16.780000 | -14.920000 | L4 | Dia 2004; Gueye and Dia 2004 |
| BIB_1199 | Primates | Cercopithecidae | Chlorocebus sabaeus | 16.830000 | -15.700000 | L3 | Dia 2004; Gueye and Dia 2004 |
| BIB_1198 | Primates | Cercopithecidae | Chlorocebus sabaeus | 16.120000 | -13.690000 | M5 | Gueye and Dia 2004 |
| BIB_1197 | Primates | Cercopithecidae | Chlorocebus sabaeus | 16.303340 | -16.401357 | M2 | National Research Council 1981; Ly and Zein 2009 |
| BIB_1196 | Primates | Cercopithecidae | Chlorocebus sabaeus | 18.450000 | -9.500000 | J9 | Holl 2009 |
| BIB_1195 | Primates | Cercopithecidae | Chlorocebus sabaeus | 16.510000 | -14.190000 | M5 | Dia 2004; Gueye and Dia 2004 |
| BIB_1194 | Primates | Cercopithecidae | Chlorocebus sabaeus | 15.300000 | -12.500000 | N6 | Dia 2004; Gueye and Dia 2004 |
| 18588 | Primates | Cercopithecidae | Chlorocebus sabaeus | 15.476092 | -12.933600 | N6 |  |
| BIB_1225 | Primates | Cercopithecidae | Erythrocebus patas | 14.775834 | -12.213947 | O7 | Mauritanie 2000 SARL 2001 |
| BIB_1224 | Primates | Cercopithecidae | Erythrocebus patas | 16.303340 | -16.401357 | M2 | National Research Council 1981 |
| BIB_1222 | Primates | Cercopithecidae | Erythrocebus patas | 16.520424 | -16.279112 | M2 | Diarra 1994 |
| BIB_1215 | Primates | Cercopithecidae | Erythrocebus patas | 15.470000 | -12.950000 | N6 | Dia 2004; Gueye and Dia 2004 |
| BIB_1214 | Primates | Cercopithecidae | Erythrocebus patas | 16.710000 | -16.120000 | L3 | Dia 2004; Gueye and Dia 2004 |
| BIB_1213 | Primates | Cercopithecidae | Erythrocebus patas | 16.780000 | -14.920000 | L4 | Dia 2004; Gueye and Dia 2004 |
| BIB_1212 | Primates | Cercopithecidae | Erythrocebus patas | 16.635783 | -16.161282 | L2 | Granjon et al 1997 |
| BIB_1211 | Primates | Cercopithecidae | Erythrocebus patas | 16.830000 | -15.700000 | L3 | Dia 2004; Gueye and Dia 2004 |
| BIB_1210 | Primates | Cercopithecidae | Erythrocebus patas | 16.120000 | -13.690000 | M5 | Gueye and Dia 2004 |
| BIB_1209 | Primates | Cercopithecidae | Erythrocebus patas | 16.400000 | -16.400000 | M2 | Spatz 1930 |
| BIB_1208 | Primates | Cercopithecidae | Erythrocebus patas | 16.607000 | -16.439323 | M2 | Granjon et al 1997 |
| BIB_1207 | Primates | Cercopithecidae | Erythrocebus patas | 16.425376 | -9.630707 | M9 | Kirsch-Jung and Khtour 2007 |
| BIB_1206 | Primates | Cercopithecidae | Erythrocebus patas | 16.510000 | -14.190000 | M5 | Dia 2004; Gueye and Dia 2004 |
| BIB_1205 | Primates | Cercopithecidae | Erythrocebus patas | 15.300000 | -12.500000 | N6 | Dia 2004; Gueye and Dia 2004 |
| 19773 | Primates | Cercopithecidae | Erythrocebus patas | 17.887298 | -12.110844 | K7 |  |
| 19752 | Primates | Cercopithecidae | Erythrocebus patas | 17.887298 | -12.110844 | K7 |  |
| 19652 | Primates | Cercopithecidae | Erythrocebus patas | 17.152482 | -12.199115 | L7 |  |
| 18923 | Primates | Cercopithecidae | Erythrocebus patas | 16.954852 | -12.229285 | L7 |  |
| 18709 | Primates | Cercopithecidae | Erythrocebus patas | 15.784802 | -11.400002 | M7 |  |
| 18651 | Primates | Cercopithecidae | Erythrocebus patas | 14.768328 | -12.200763 | O7 |  |
| 16168 | Primates | Cercopithecidae | Erythrocebus patas | 16.377930 | -16.421630 | M2 |  |
| 16073 | Primates | Cercopithecidae | Erythrocebus patas | 15.628413 | -11.233462 | N8 |  |
| 16029 | Primates | Cercopithecidae | Erythrocebus patas | 15.661542 | -11.397000 | M7 |  |
| 12748 | Primates | Cercopithecidae | Erythrocebus patas | 17.170608 | -10.637773 | L8 |  |
| 12587 | Primates | Cercopithecidae | Erythrocebus patas | 16.153808 | -10.752278 | M8 |  |
| 12571 | Primates | Cercopithecidae | Erythrocebus patas | 16.068188 | -10.839677 | M8 |  |
| 12559 | Primates | Cercopithecidae | Erythrocebus patas | 16.053727 | -10.852842 | M8 |  |
| 12522 | Primates | Cercopithecidae | Erythrocebus patas | 16.019812 | -10.923370 | M8 |  |
| 12513 | Primates | Cercopithecidae | Erythrocebus patas | 16.019812 | -10.923370 | M8 |  |
| 12412 | Primates | Cercopithecidae | Erythrocebus patas | 17.042720 | -12.160860 | L7 |  |
| 12369 | Primates | Cercopithecidae | Erythrocebus patas | 17.240833 | -12.101667 | L7 |  |
| 12328 | Primates | Cercopithecidae | Erythrocebus patas | 17.492952 | -12.116253 | K7 |  |
| 12282 | Primates | Cercopithecidae | Erythrocebus patas | 17.875568 | -12.091653 | K7 |  |
| BIB_1232 | Primates | Cercopithecidae | Papio papio | 14.775834 | -12.213947 | O7 | Mauritanie 2000 SARL 2001 |
| BIB_1228 | Primates | Cercopithecidae | Papio papio | 16.538033 | -10.741550 | L8 | Nickel 2003 |
| BIB_1227 | Primates | Cercopithecidae | Papio papio | 16.911465 | -10.140778 | L9 | Kirsch-Jung and Khtour 2007 |
| BIB_1226 | Primates | Cercopithecidae | Papio papio | 16.400000 | -16.400000 | M2 | Spatz 1930 |
| 19766 | Primates | Cercopithecidae | Papio papio | 17.881083 | -12.094000 | K7 |  |
| 19679 | Primates | Cercopithecidae | Papio papio | 16.538033 | -10.741550 | L8 |  |
| 19651 | Primates | Cercopithecidae | Papio papio | 17.152482 | -12.199115 | L7 |  |
| 19641 | Primates | Cercopithecidae | Papio papio | 15.957078 | -12.009859 | M7 |  |
| 19019 | Primates | Cercopithecidae | Papio papio | 15.957078 | -12.009859 | M7 |  |
| 18938 | Primates | Cercopithecidae | Papio papio | 17.101413 | -12.256712 | L7 |  |
| 18843 | Primates | Cercopithecidae | Papio papio | 17.657525 | -11.391248 | K7 |  |
| 16101 | Primates | Cercopithecidae | Papio papio | 15.757282 | -11.231708 | M8 |  |
| 16078 | Primates | Cercopithecidae | Papio papio | 15.628413 | -11.233462 | N8 |  |
| 16052 | Primates | Cercopithecidae | Papio papio | 15.415229 | -11.190639 | N8 |  |
| 15994 | Primates | Cercopithecidae | Papio papio | 16.002553 | -11.871748 | M7 |  |
| 15986 | Primates | Cercopithecidae | Papio papio | 16.040542 | -11.928843 | M7 |  |
| 12781 | Primates | Cercopithecidae | Papio papio | 17.152482 | -12.199115 | L7 |  |
| 12708 | Primates | Cercopithecidae | Papio papio | 16.817493 | -10.740085 | L8 |  |
| 12673 | Primates | Cercopithecidae | Papio papio | 16.579150 | -10.704550 | L8 |  |
| 12635 | Primates | Cercopithecidae | Papio papio | 16.538033 | -10.741550 | L8 |  |
| 12634 | Primates | Cercopithecidae | Papio papio | 16.538033 | -10.741550 | L8 |  |
| 12633 | Primates | Cercopithecidae | Papio papio | 16.538033 | -10.741550 | L8 |  |
| 12632 | Primates | Cercopithecidae | Papio papio | 16.538033 | -10.741550 | L8 |  |
| 12631 | Primates | Cercopithecidae | Papio papio | 16.538033 | -10.741550 | L8 |  |
| 12621 | Primates | Cercopithecidae | Papio papio | 16.412065 | -10.849448 | M8 |  |
| 12613 | Primates | Cercopithecidae | Papio papio | 16.412065 | -10.849448 | M8 |  |
| 12612 | Primates | Cercopithecidae | Papio papio | 16.412065 | -10.849448 | M8 |  |
| 12611 | Primates | Cercopithecidae | Papio papio | 16.412065 | -10.849448 | M8 |  |
| 12610 | Primates | Cercopithecidae | Papio papio | 16.412065 | -10.849448 | M8 |  |
| 12598 | Primates | Cercopithecidae | Papio papio | 16.301168 | -10.853177 | M8 |  |
| 12597 | Primates | Cercopithecidae | Papio papio | 16.301168 | -10.853177 | M8 |  |
| 12596 | Primates | Cercopithecidae | Papio papio | 16.301168 | -10.853177 | M8 |  |
| 12595 | Primates | Cercopithecidae | Papio papio | 16.301168 | -10.853177 | M8 |  |
| 12594 | Primates | Cercopithecidae | Papio papio | 16.301168 | -10.853177 | M8 |  |
| 12573 | Primates | Cercopithecidae | Papio papio | 16.068188 | -10.839677 | M8 |  |
| 12572 | Primates | Cercopithecidae | Papio papio | 16.068188 | -10.839677 | M8 |  |
| 12542 | Primates | Cercopithecidae | Papio papio | 15.972653 | -10.896275 | M8 |  |
| 12541 | Primates | Cercopithecidae | Papio papio | 15.972653 | -10.896275 | M8 |  |
| 12540 | Primates | Cercopithecidae | Papio papio | 15.972653 | -10.896275 | M8 |  |
| 12539 | Primates | Cercopithecidae | Papio papio | 15.972653 | -10.896275 | M8 |  |
| 12538 | Primates | Cercopithecidae | Papio papio | 15.972653 | -10.896275 | M8 |  |
| 12515 | Primates | Cercopithecidae | Papio papio | 16.019812 | -10.923370 | M8 |  |
| 12514 | Primates | Cercopithecidae | Papio papio | 16.019812 | -10.923370 | M8 |  |
| 12512 | Primates | Cercopithecidae | Papio papio | 16.019812 | -10.923370 | M8 |  |
| 12511 | Primates | Cercopithecidae | Papio papio | 16.019812 | -10.923370 | M8 |  |
| 12504 | Primates | Cercopithecidae | Papio papio | 16.008648 | -10.929053 | M8 |  |
| 12497 | Primates | Cercopithecidae | Papio papio | 15.923907 | -10.910007 | M8 |  |
| 12486 | Primates | Cercopithecidae | Papio papio | 15.676287 | -11.026053 | M8 |  |
| 12485 | Primates | Cercopithecidae | Papio papio | 15.676287 | -11.026053 | M8 |  |
| 12484 | Primates | Cercopithecidae | Papio papio | 15.676287 | -11.026053 | M8 |  |
| 12483 | Primates | Cercopithecidae | Papio papio | 15.676287 | -11.026053 | M8 |  |
| 12482 | Primates | Cercopithecidae | Papio papio | 15.676287 | -11.026053 | M8 |  |
| 12481 | Primates | Cercopithecidae | Papio papio | 15.676287 | -11.026053 | M8 |  |
| 12480 | Primates | Cercopithecidae | Papio papio | 15.676287 | -11.026053 | M8 |  |
| 12462 | Primates | Cercopithecidae | Papio papio | 15.822447 | -11.145082 | M8 |  |
| 12455 | Primates | Cercopithecidae | Papio papio | 15.845405 | -11.205873 | M8 |  |
| 12454 | Primates | Cercopithecidae | Papio papio | 15.845405 | -11.205873 | M8 |  |
| 12451 | Primates | Cercopithecidae | Papio papio | 15.845405 | -11.205873 | M8 |  |
| 12450 | Primates | Cercopithecidae | Papio papio | 15.845405 | -11.205873 | M8 |  |
| 12449 | Primates | Cercopithecidae | Papio papio | 15.845405 | -11.205873 | M8 |  |
| 12413 | Primates | Cercopithecidae | Papio papio | 17.042720 | -12.160860 | L7 |  |
| 12411 | Primates | Cercopithecidae | Papio papio | 17.042720 | -12.160860 | L7 |  |
| 12410 | Primates | Cercopithecidae | Papio papio | 17.042720 | -12.160860 | L7 |  |
| 12409 | Primates | Cercopithecidae | Papio papio | 17.042720 | -12.160860 | L7 |  |
| 12408 | Primates | Cercopithecidae | Papio papio | 17.042720 | -12.160860 | L7 |  |
| 12383 | Primates | Cercopithecidae | Papio papio | 17.232793 | -12.100005 | L7 |  |
| 12382 | Primates | Cercopithecidae | Papio papio | 17.232793 | -12.100005 | L7 |  |
| 12380 | Primates | Cercopithecidae | Papio papio | 17.240833 | -12.101667 | L7 |  |
| 12379 | Primates | Cercopithecidae | Papio papio | 17.240833 | -12.101667 | L7 |  |
| 12371 | Primates | Cercopithecidae | Papio papio | 17.240833 | -12.101667 | L7 |  |
| 12370 | Primates | Cercopithecidae | Papio papio | 17.240833 | -12.101667 | L7 |  |
| 12368 | Primates | Cercopithecidae | Papio papio | 17.240833 | -12.101667 | L7 |  |
| 12367 | Primates | Cercopithecidae | Papio papio | 17.240833 | -12.101667 | L7 |  |
| 12366 | Primates | Cercopithecidae | Papio papio | 17.240833 | -12.101667 | L7 |  |
| 12365 | Primates | Cercopithecidae | Papio papio | 17.240833 | -12.101667 | L7 |  |
| 12364 | Primates | Cercopithecidae | Papio papio | 17.240833 | -12.101667 | L7 |  |
| 12363 | Primates | Cercopithecidae | Papio papio | 17.240833 | -12.101667 | L7 |  |
| 12362 | Primates | Cercopithecidae | Papio papio | 17.240833 | -12.101667 | L7 |  |
| 12361 | Primates | Cercopithecidae | Papio papio | 17.240833 | -12.101667 | L7 |  |
| 12360 | Primates | Cercopithecidae | Papio papio | 17.240833 | -12.101667 | L7 |  |
| BIB_1234 | Primates | Galagidae | Galago senegalensis | 16.303340 | -16.401357 | M2 | National Research Council 1981 |
| BIB_1281 | Proboscidea | Elephantidae | Loxodonta africana | 22.934587 | -11.653543 | E7 | Messerli and Winiger 1992 |
| BIB_1280 | Proboscidea | Elephantidae | Loxodonta africana | 15.740000 | -9.620000 | M9 | Duchemin 1949 |
| BIB_1279 | Proboscidea | Elephantidae | Loxodonta africana | 16.260000 | -13.940000 | M5 | Gueye and Dia 2004 |
| BIB_1278 | Proboscidea | Elephantidae | Loxodonta africana | 15.263000 | -12.818000 | N6 | Duchemin 1949 |
| BIB_1277 | Proboscidea | Elephantidae | Loxodonta africana | 16.387240 | -10.175642 | M9 | Duchemin 1949 |
| BIB_1276 | Proboscidea | Elephantidae | Loxodonta africana | 17.234600 | -10.559400 | L8 | Grandidier 1932 |
| BIB_1275 | Proboscidea | Elephantidae | Loxodonta africana | 18.531291 | -12.682983 | J6 | Messerli and Winiger 1992 |
| BIB_1274 | Proboscidea | Elephantidae | Loxodonta africana | 15.570000 | -11.940000 | N7 | Duchemin 1949 |
| BIB_1273 | Proboscidea | Elephantidae | Loxodonta africana | 15.977785 | -12.008811 | M7 | Moreno 2009 |
| BIB_1272 | Proboscidea | Elephantidae | Loxodonta africana | 14.775834 | -12.213947 | O7 | Mauritanie 2000 SARL 2001 |
| BIB_1271 | Proboscidea | Elephantidae | Loxodonta africana | 16.547000 | -10.849000 | L8 | Duchemin 1949 |
| BIB_1270 | Proboscidea | Elephantidae | Loxodonta africana | 18.828247 | -11.789290 | J7 | Messerli and Winiger 1992 |
| BIB_1269 | Proboscidea | Elephantidae | Loxodonta africana | 16.579150 | -10.704550 | L8 | Duchemin 1949 |
| BIB_1268 | Proboscidea | Elephantidae | Loxodonta africana | 15.770000 | -10.410000 | M9 | Duchemin 1949 |
| BIB_1267 | Proboscidea | Elephantidae | Loxodonta africana | 15.470000 | -12.950000 | N6 | Dia 2004; Gueye and Dia 2004 |
| BIB_1266 | Proboscidea | Elephantidae | Loxodonta africana | 16.710000 | -16.120000 | L3 | Gueye and Dia 2004 |
| BIB_1265 | Proboscidea | Elephantidae | Loxodonta africana | 15.836446 | -12.855671 | M6 | Duchemin 1949 |
| BIB_1264 | Proboscidea | Elephantidae | Loxodonta africana | 16.780000 | -14.920000 | L4 | Gueye and Dia 2004 |
| BIB_1263 | Proboscidea | Elephantidae | Loxodonta africana | 15.900000 | -11.560000 | M7 | Munier 1952 |
| BIB_1262 | Proboscidea | Elephantidae | Loxodonta africana | 18.710000 | -11.550000 | J7 | Campbell et al 2006 |
| BIB_1261 | Proboscidea | Elephantidae | Loxodonta africana | 16.916670 | -11.953799 | L7 | Messerli and Winiger 1992 |
| BIB_1260 | Proboscidea | Elephantidae | Loxodonta africana | 16.338801 | -11.978097 | M7 | Munier 1952 |
| BIB_1259 | Proboscidea | Elephantidae | Loxodonta africana | 17.950000 | -12.250000 | K7 | Tellería 2009 |
| BIB_1258 | Proboscidea | Elephantidae | Loxodonta africana | 18.114614 | -8.255796 | K11 | Messerli and Winiger 1992 |
| BIB_1257 | Proboscidea | Elephantidae | Loxodonta africana | 16.830000 | -15.700000 | L3 | Gueye and Dia 2004 |
| BIB_1256 | Proboscidea | Elephantidae | Loxodonta africana | 21.814676 | -10.296076 | G9 | Messerli and Winiger 1992 |
| BIB_1255 | Proboscidea | Elephantidae | Loxodonta africana | 16.617433 | -10.264207 | L9 | Duchemin 1949 |
| BIB_1254 | Proboscidea | Elephantidae | Loxodonta africana | 16.700000 | -10.350000 | L9 | Grandidier 1932 |
| BIB_1253 | Proboscidea | Elephantidae | Loxodonta africana | 16.120000 | -13.690000 | M5 | Dia 2004; Gueye and Dia 2004 |
| BIB_1252 | Proboscidea | Elephantidae | Loxodonta africana | 15.990000 | -10.830000 | M8 | Duchemin 1949 |
| BIB_1251 | Proboscidea | Elephantidae | Loxodonta africana | 25.041860 | -4.922384 | C14 | Messerli and Winiger 1992 |
| BIB_1250 | Proboscidea | Elephantidae | Loxodonta africana | 25.174408 | -5.612812 | C13 | Messerli and Winiger 1992 |
| BIB_1249 | Proboscidea | Elephantidae | Loxodonta africana | 16.540090 | -10.801490 | L8 | Duchemin 1949 |
| BIB_1248 | Proboscidea | Elephantidae | Loxodonta africana | 16.416000 | -10.506900 | M8 | Duchemin 1949 |
| BIB_1247 | Proboscidea | Elephantidae | Loxodonta africana | 16.510000 | -14.190000 | M5 | Gueye and Dia 2004 |
| BIB_1246 | Proboscidea | Elephantidae | Loxodonta africana | 24.781437 | -11.693376 | C7 | Messerli and Winiger 1992 |
| BIB_1245 | Proboscidea | Elephantidae | Loxodonta africana | 25.510621 | -10.807939 | C8 | Messerli and Winiger 1992 |
| BIB_1244 | Proboscidea | Elephantidae | Loxodonta africana | 25.344092 | -11.415986 | C7 | Messerli and Winiger 1992 |
| BIB_1243 | Proboscidea | Elephantidae | Loxodonta africana | 25.988889 | -11.280240 | B7 | Messerli and Winiger 1992 |
| BIB_1242 | Proboscidea | Elephantidae | Loxodonta africana | 16.560000 | -10.910000 | L8 | Duchemin 1949 |
| BIB_1241 | Proboscidea | Elephantidae | Loxodonta africana | 16.360000 | -10.140000 | M9 | Duchemin 1949 |
| BIB_1240 | Proboscidea | Elephantidae | Loxodonta africana | 21.239687 | -12.839236 | G6 | Messerli and Winiger 1992 |
| BIB_1239 | Proboscidea | Elephantidae | Loxodonta africana | 15.300000 | -12.500000 | N6 | Dia 2004; Gueye and Dia 2004 |
| BIB_1238 | Proboscidea | Elephantidae | Loxodonta africana | 20.458419 | -12.787152 | H6 | Messerli and Winiger 1992 |
| BIB_1237 | Proboscidea | Elephantidae | Loxodonta africana | 15.500000 | -12.020000 | N7 | Duchemin 1949 |
| BIB_1236 | Proboscidea | Elephantidae | Loxodonta africana | 15.580000 | -10.840000 | N8 | Duchemin 1949 |
| BIB_1235 | Proboscidea | Elephantidae | Loxodonta africana | 25.562705 | -9.245402 | C10 | Messerli and Winiger 1992 |
| 18593 | Proboscidea | Elephantidae | Loxodonta africana | 15.476092 | -12.933600 | N6 |  |
| 11088 | Proboscidea | Elephantidae | Loxodonta africana | 15.694747 | -11.237802 | M8 |  |
| 3567 | Proboscidea | Elephantidae | Loxodonta africana | 21.493688 | -11.331722 | G7 |  |
| BIB_1335 | Rodentia | Ctenodactylidae | Felovia vae | 17.821264 | -12.185762 | K7 | Padial and Tellería 2009 |
| BIB_1318 | Rodentia | Ctenodactylidae | Felovia vae | 20.000000 | -13.050000 | I6 | Diatta et al 2015 |
| BIB_1317 | Rodentia | Ctenodactylidae | Felovia vae | 16.538033 | -10.741550 | L8 | Nickel 2003 |
| BIB_1315 | Rodentia | Ctenodactylidae | Felovia vae | 17.750000 | -11.500000 | K7 | Padial and Tellería 2009 |
| BIB_1314 | Rodentia | Ctenodactylidae | Felovia vae | 22.650000 | -12.540000 | F6 | Heim de Balsac 1948; Le Berre 1990 |
| BIB_1313 | Rodentia | Ctenodactylidae | Felovia vae | 18.200000 | -11.733333 | K7 | Padial and Tellería 2009 |
| BIB_1312 | Rodentia | Ctenodactylidae | Felovia vae | 18.450000 | -11.533333 | J7 | Padial and Tellería 2009 |
| BIB_1311 | Rodentia | Ctenodactylidae | Felovia vae | 15.971176 | -11.813263 | M7 | Granjon et al 2009 |
| BIB_1310 | Rodentia | Ctenodactylidae | Felovia vae | 17.834850 | -11.557833 | K7 | Padial and Tellería 2009 |
| BIB_1307 | Rodentia | Ctenodactylidae | Felovia vae | 17.566667 | -12.166667 | K7 | Padial and Tellería 2009 |
| BIB_1289 | Rodentia | Ctenodactylidae | Felovia vae | 21.525000 | -12.860600 | G6 | Heim de Balsac 1948; Le Berre 1990 |
| BIB_1285 | Rodentia | Ctenodactylidae | Felovia vae | 16.927979 | -11.895995 | L7 | Granjon et al 2009 |
| BIB_1283 | Rodentia | Ctenodactylidae | Felovia vae | 20.527377 | -13.019975 | H6 | Granjon et al 2009 |
| BIB_1282 | Rodentia | Ctenodactylidae | Felovia vae | 18.466667 | -11.733333 | J7 | Padial and Tellería 2009 |
| 19800 | Rodentia | Ctenodactylidae | Felovia vae | 18.150508 | -12.065716 | K7 |  |
| 19747 | Rodentia | Ctenodactylidae | Felovia vae | 16.543913 | -10.751155 | L8 |  |
| 19746 | Rodentia | Ctenodactylidae | Felovia vae | 16.543913 | -10.751155 | L8 |  |
| 19693 | Rodentia | Ctenodactylidae | Felovia vae | 16.543913 | -10.751155 | L8 |  |
| 19680 | Rodentia | Ctenodactylidae | Felovia vae | 16.538033 | -10.741550 | L8 |  |
| 18988 | Rodentia | Ctenodactylidae | Felovia vae | 16.002553 | -11.871748 | M7 |  |
| 18847 | Rodentia | Ctenodactylidae | Felovia vae | 17.657525 | -11.391248 | K7 |  |
| 18816 | Rodentia | Ctenodactylidae | Felovia vae | 16.538033 | -10.741550 | L8 |  |
| 18697 | Rodentia | Ctenodactylidae | Felovia vae | 16.002553 | -11.871748 | M7 |  |
| 18689 | Rodentia | Ctenodactylidae | Felovia vae | 15.944687 | -11.929082 | M7 |  |
| 15996 | Rodentia | Ctenodactylidae | Felovia vae | 16.002553 | -11.871748 | M7 |  |
| 15987 | Rodentia | Ctenodactylidae | Felovia vae | 16.040542 | -11.928843 | M7 |  |
| 15985 | Rodentia | Ctenodactylidae | Felovia vae | 15.944687 | -11.929082 | M7 |  |
| 13845 | Rodentia | Ctenodactylidae | Felovia vae | 18.075420 | -12.036527 | K7 |  |
| 13840 | Rodentia | Ctenodactylidae | Felovia vae | 18.186208 | -11.889620 | K7 |  |
| 13839 | Rodentia | Ctenodactylidae | Felovia vae | 18.266550 | -11.796350 | K7 |  |
| 13828 | Rodentia | Ctenodactylidae | Felovia vae | 18.505612 | -11.434330 | J7 |  |
| 19856 | Rodentia | Dipodidae | Jaculus cf. hirtipes | 16.347967 | -16.399085 | M2 |  |
| 19855 | Rodentia | Dipodidae | Jaculus cf. hirtipes | 16.368080 | -16.420075 | M2 |  |
| 19854 | Rodentia | Dipodidae | Jaculus cf. hirtipes | 16.369265 | -16.420316 | M2 |  |
| 19853 | Rodentia | Dipodidae | Jaculus cf. hirtipes | 16.385057 | -16.423265 | M2 |  |
| 19852 | Rodentia | Dipodidae | Jaculus cf. hirtipes | 16.399468 | -16.434140 | M2 |  |
| 19851 | Rodentia | Dipodidae | Jaculus cf. hirtipes | 16.358685 | -16.412122 | M2 |  |
| 19850 | Rodentia | Dipodidae | Jaculus cf. hirtipes | 16.399468 | -16.434140 | M2 |  |
| 19849 | Rodentia | Dipodidae | Jaculus cf. hirtipes | 16.373815 | -16.421493 | M2 |  |
| 15935 | Rodentia | Dipodidae | Jaculus cf. hirtipes | 18.255663 | -11.780890 | K7 |  |
| 15906 | Rodentia | Dipodidae | Jaculus cf. hirtipes | 20.847822 | -12.150492 | H7 |  |
| 19893 | Rodentia | Dipodidae | Jaculus jaculus | 19.076182 | -15.044215 | J4 |  |
| 19892 | Rodentia | Dipodidae | Jaculus jaculus | 19.075372 | -15.043983 | J4 |  |
| 19891 | Rodentia | Dipodidae | Jaculus jaculus | 19.075655 | -15.036148 | J4 |  |
| 19890 | Rodentia | Dipodidae | Jaculus jaculus | 19.044415 | -15.091852 | J4 |  |
| 19889 | Rodentia | Dipodidae | Jaculus jaculus | 19.044968 | -15.092365 | J4 |  |
| 19888 | Rodentia | Dipodidae | Jaculus jaculus | 19.044445 | -15.091390 | J4 |  |
| 19887 | Rodentia | Dipodidae | Jaculus jaculus | 19.045007 | -15.092515 | J4 |  |
| 19886 | Rodentia | Dipodidae | Jaculus jaculus | 19.043818 | -15.093622 | J4 |  |
| 19885 | Rodentia | Dipodidae | Jaculus jaculus | 19.041940 | -15.092880 | J4 |  |
| 19884 | Rodentia | Dipodidae | Jaculus jaculus | 19.041812 | -15.092960 | J4 |  |
| 19883 | Rodentia | Dipodidae | Jaculus jaculus | 19.041773 | -15.094307 | J4 |  |
| 19882 | Rodentia | Dipodidae | Jaculus jaculus | 19.041025 | -15.093987 | J4 |  |
| 19881 | Rodentia | Dipodidae | Jaculus jaculus | 18.814063 | -15.481520 | J3 |  |
| 19880 | Rodentia | Dipodidae | Jaculus jaculus | 18.815037 | -15.480432 | J3 |  |
| 19879 | Rodentia | Dipodidae | Jaculus jaculus | 18.816335 | -15.477790 | J3 |  |
| 19878 | Rodentia | Dipodidae | Jaculus jaculus | 17.207400 | -16.042905 | L3 |  |
| 19877 | Rodentia | Dipodidae | Jaculus jaculus | 17.205222 | -16.042935 | L3 |  |
| 19876 | Rodentia | Dipodidae | Jaculus jaculus | 16.843265 | -16.137305 | L3 |  |
| 19875 | Rodentia | Dipodidae | Jaculus jaculus | 16.837957 | -16.149825 | L3 |  |
| 19874 | Rodentia | Dipodidae | Jaculus jaculus | 16.845613 | -16.138797 | L3 |  |
| 19873 | Rodentia | Dipodidae | Jaculus jaculus | 16.827125 | -16.157737 | L2 |  |
| 19872 | Rodentia | Dipodidae | Jaculus jaculus | 16.824018 | -16.160787 | L2 |  |
| 19871 | Rodentia | Dipodidae | Jaculus jaculus | 16.824747 | -16.158827 | L2 |  |
| 19870 | Rodentia | Dipodidae | Jaculus jaculus | 16.386922 | -16.473782 | M2 |  |
| 19869 | Rodentia | Dipodidae | Jaculus jaculus | 16.414433 | -16.465735 | M2 |  |
| 19868 | Rodentia | Dipodidae | Jaculus jaculus | 16.413418 | -16.465935 | M2 |  |
| 19867 | Rodentia | Dipodidae | Jaculus jaculus | 20.564303 | -16.150050 | H2 |  |
| 19866 | Rodentia | Dipodidae | Jaculus jaculus | 20.556745 | -16.152083 | H2 |  |
| 19865 | Rodentia | Dipodidae | Jaculus jaculus | 20.548420 | -16.153613 | H2 |  |
| 19864 | Rodentia | Dipodidae | Jaculus jaculus | 20.557157 | -16.151753 | H2 |  |
| 19863 | Rodentia | Dipodidae | Jaculus jaculus | 20.579422 | -16.129780 | H2 |  |
| 15954 | Rodentia | Dipodidae | Jaculus jaculus | 17.603628 | -12.789012 | K6 |  |
| 15953 | Rodentia | Dipodidae | Jaculus jaculus | 17.615095 | -12.745202 | K6 |  |
| BIB_1480 | Rodentia | Dipodidae | Jaculus sp. | 22.974539 | -11.941135 | E7 | Granjon et al 2009 |
| BIB_1471 | Rodentia | Dipodidae | Jaculus sp. | 16.568819 | -15.596202 | M3 | Granjon et al 2009 |
| BIB_1467 | Rodentia | Dipodidae | Jaculus sp. | 18.189955 | -16.007304 | K3 | Granjon et al 1997 |
| BIB_1433 | Rodentia | Dipodidae | Jaculus sp. | 18.115564 | -16.015721 | K3 | Granjon et al 2009 |
| BIB_1432 | Rodentia | Dipodidae | Jaculus sp. | 17.983333 | -12.233333 | K7 | Diatta et al 2015 |
| BIB_1428 | Rodentia | Dipodidae | Jaculus sp. | 20.068708 | -14.720671 | I4 | Granjon et al 2009 |
| BIB_1423 | Rodentia | Dipodidae | Jaculus sp. | 16.300000 | -10.250000 | M9 | Granjon et al 2009 |
| BIB_1422 | Rodentia | Dipodidae | Jaculus sp. | 19.751600 | -14.428100 | I4 | Jullien and Petter 1970 |
| BIB_1421 | Rodentia | Dipodidae | Jaculus sp. | 22.983333 | -12.000000 | E7 | Diatta et al 2015 |
| BIB_1389 | Rodentia | Dipodidae | Jaculus sp. | 16.303340 | -16.401357 | M2 | Granjon et al 2009 |
| BIB_1386 | Rodentia | Dipodidae | Jaculus sp. | 20.460500 | -12.358300 | H6 | Dekeyser and Villiers 1956 |
| BIB_1375 | Rodentia | Dipodidae | Jaculus sp. | 16.620000 | -14.290000 | L4 | Granjon et al 2009 |
| BIB_1374 | Rodentia | Dipodidae | Jaculus sp. | 20.585660 | -16.236789 | H2 | Granjon et al 2009 |
| BIB_1373 | Rodentia | Dipodidae | Jaculus sp. | 20.083333 | -15.966667 | I3 | Klein et al 1975 |
| BIB_1372 | Rodentia | Dipodidae | Jaculus sp. | 16.680556 | -9.301944 | L10 | Ba et al 2001 |
| BIB_1371 | Rodentia | Dipodidae | Jaculus sp. | 16.680556 | -9.301944 | L10 | Denys et al 2001 |
| BIB_1370 | Rodentia | Dipodidae | Jaculus sp. | 20.266667 | -13.116667 | H6 | Diatta et al 2015 |
| BIB_1367 | Rodentia | Dipodidae | Jaculus sp. | 20.536743 | -12.961179 | H6 | Granjon et al 2009 |
| BIB_1358 | Rodentia | Dipodidae | Jaculus sp. | 17.143650 | -16.164047 | L2 | Granjon et al 2009 |
| BIB_1352 | Rodentia | Dipodidae | Jaculus sp. | 19.740000 | -14.370000 | I4 | Poulet 1974 |
| BIB_1351 | Rodentia | Dipodidae | Jaculus sp. | 19.733333 | -14.366667 | I4 | Diatta et al 2015 |
| 19016 | Rodentia | Dipodidae | Jaculus sp. | 17.329165 | -10.708572 | L8 |  |
| 18584 | Rodentia | Dipodidae | Jaculus sp. | 15.604115 | -13.091327 | N6 |  |
| 15967 | Rodentia | Dipodidae | Jaculus sp. | 16.270153 | -12.160453 | M7 |  |
| 15438 | Rodentia | Dipodidae | Jaculus sp. | 20.978747 | -11.905247 | H7 |  |
| 13825 | Rodentia | Dipodidae | Jaculus sp. | 18.690398 | -11.623535 | J7 |  |
| 13824 | Rodentia | Dipodidae | Jaculus sp. | 18.700608 | -11.633887 | J7 |  |
| 13785 | Rodentia | Dipodidae | Jaculus sp. | 19.722428 | -12.687930 | I6 |  |
| 13681 | Rodentia | Dipodidae | Jaculus sp. | 24.573660 | -11.386578 | D7 |  |
| 13663 | Rodentia | Dipodidae | Jaculus sp. | 23.566840 | -11.612132 | E7 |  |
| 13660 | Rodentia | Dipodidae | Jaculus sp. | 23.438058 | -11.900442 | E7 |  |
| BIB_1500 | Rodentia | Hystricidae | Hystrix cristata | 16.260000 | -13.940000 | M5 | Dia 2004; Gueye and Dia 2004 |
| BIB_1498 | Rodentia | Hystricidae | Hystrix cristata | 18.307733 | -12.056339 | K7 | Granjon et al 2009 |
| BIB_1497 | Rodentia | Hystricidae | Hystrix cristata | 16.303340 | -16.401357 | M2 | National Research Council 1981 |
| BIB_1496 | Rodentia | Hystricidae | Hystrix cristata | 16.384695 | -9.300555 | M10 | Kirsch-Jung and Khtour 2007 |
| BIB_1495 | Rodentia | Hystricidae | Hystrix cristata | 15.504833 | -9.816833 | N9 | Kirsch-Jung and Khtour 2007 |
| BIB_1494 | Rodentia | Hystricidae | Hystrix cristata | 15.470000 | -12.950000 | N6 | Dia 2004; Gueye and Dia 2004 |
| BIB_1493 | Rodentia | Hystricidae | Hystrix cristata | 16.710000 | -16.120000 | L3 | Dia 2004; Gueye and Dia 2004 |
| BIB_1492 | Rodentia | Hystricidae | Hystrix cristata | 16.780000 | -14.920000 | L4 | Dia 2004; Gueye and Dia 2004 |
| BIB_1491 | Rodentia | Hystricidae | Hystrix cristata | 19.751600 | -14.428100 | I4 | Jullien and Petter 1970 |
| BIB_1490 | Rodentia | Hystricidae | Hystrix cristata | 16.403148 | -9.559860 | M9 | Kirsch-Jung and Khtour 2007 |
| BIB_1488 | Rodentia | Hystricidae | Hystrix cristata | 16.911465 | -10.140778 | L9 | Kirsch-Jung and Khtour 2007 |
| BIB_1487 | Rodentia | Hystricidae | Hystrix cristata | 16.830000 | -15.700000 | L3 | Dia 2004; Gueye and Dia 2004 |
| BIB_1486 | Rodentia | Hystricidae | Hystrix cristata | 16.120000 | -13.690000 | M5 | Dia 2004; Gueye and Dia 2004 |
| BIB_1485 | Rodentia | Hystricidae | Hystrix cristata | 16.425376 | -9.630707 | M9 | Kirsch-Jung and Khtour 2007 |
| BIB_1484 | Rodentia | Hystricidae | Hystrix cristata | 16.620000 | -14.290000 | L4 | Granjon et al 2009 |
| BIB_1483 | Rodentia | Hystricidae | Hystrix cristata | 16.510000 | -14.190000 | M5 | Dia 2004; Gueye and Dia 2004 |
| BIB_1482 | Rodentia | Hystricidae | Hystrix cristata | 20.536743 | -12.961179 | H6 | Dekeyser and Villiers 1956; Le Berre 1990 |
| BIB_1481 | Rodentia | Hystricidae | Hystrix cristata | 15.300000 | -12.500000 | N6 | Dia 2004; Gueye and Dia 2004 |
| 18950 | Rodentia | Hystricidae | Hystrix cristata | 17.451667 | -12.394850 | K6 |  |
| 15975 | Rodentia | Hystricidae | Hystrix cristata | 15.944687 | -11.929082 | M7 |  |
| 12692 | Rodentia | Hystricidae | Hystrix cristata | 16.579150 | -10.704550 | L8 |  |
| 12583 | Rodentia | Hystricidae | Hystrix cristata | 16.153808 | -10.752278 | M8 |  |
| 12556 | Rodentia | Hystricidae | Hystrix cristata | 15.970850 | -10.901560 | M8 |  |
| 12523 | Rodentia | Hystricidae | Hystrix cristata | 16.019812 | -10.923370 | M8 |  |
| 12385 | Rodentia | Hystricidae | Hystrix cristata | 17.232793 | -12.100005 | L7 |  |
| BIB_1529 | Rodentia | Muridae | Acomys airensis | 22.819879 | -12.734806 | F6 | Granjon et al 2009 |
| BIB_1528 | Rodentia | Muridae | Acomys airensis | 20.252804 | -13.088188 | H6 | Nicolas et al 2009 |
| BIB_1527 | Rodentia | Muridae | Acomys airensis | 18.381180 | -11.716032 | J7 | Granjon et al 2009 |
| BIB_1526 | Rodentia | Muridae | Acomys airensis | 17.492582 | -11.954418 | K7 | Granjon et al 2009 |
| BIB_1525 | Rodentia | Muridae | Acomys airensis | 18.783333 | -11.683333 | J7 | Diatta et al 2015 |
| BIB_1524 | Rodentia | Muridae | Acomys airensis | 20.000000 | -13.050000 | I6 | Diatta et al 2015 |
| BIB_1523 | Rodentia | Muridae | Acomys airensis | 21.068914 | -16.992276 | H2 | Dekeyser and Villiers 1956; Le Berre 1990 |
| BIB_1515 | Rodentia | Muridae | Acomys airensis | 16.538033 | -10.741550 | L8 | Nickel 2003 |
| BIB_1514 | Rodentia | Muridae | Acomys airensis | 16.300000 | -10.250000 | M9 | Granjon et al 2009 |
| BIB_1508 | Rodentia | Muridae | Acomys airensis | 16.620000 | -14.290000 | L4 | Granjon et al 2009 |
| BIB_1506 | Rodentia | Muridae | Acomys airensis | 16.680556 | -9.301944 | L10 | Ba et al 2001 |
| BIB_1505 | Rodentia | Muridae | Acomys airensis | 16.680556 | -9.301944 | L10 | Denys et al 2001 |
| BIB_1503 | Rodentia | Muridae | Acomys airensis | 20.516667 | -13.050000 | H6 | Nicolas et al 2009 |
| BIB_1502 | Rodentia | Muridae | Acomys airensis | 20.266667 | -13.116667 | H6 | Diatta et al 2015 |
| BIB_1501 | Rodentia | Muridae | Acomys airensis | 20.445083 | -13.008487 | H6 | Granjon et al 2009 |
| 13697 | Rodentia | Muridae | Acomys airensis | 25.156463 | -11.534827 | C7 |  |
| 10119 | Rodentia | Muridae | Acomys airensis | 18.150508 | -12.065716 | K7 |  |
| 10118 | Rodentia | Muridae | Acomys airensis | 18.150508 | -12.065716 | K7 |  |
| 10093 | Rodentia | Muridae | Acomys airensis | 18.442821 | -11.387395 | J7 |  |
| 10092 | Rodentia | Muridae | Acomys airensis | 18.442821 | -11.387395 | J7 |  |
| 9359 | Rodentia | Muridae | Acomys airensis | 16.489543 | -11.057988 | M8 |  |
| BIB_1639 | Rodentia | Muridae | Arvicanthis niloticus | 16.858848 | -9.408776 | L10 | Kirsch-Jung and Khtour 2007 |
| BIB_1638 | Rodentia | Muridae | Arvicanthis niloticus | 16.620000 | -14.290000 | L4 | Granjon et al 2009 |
| BIB_1637 | Rodentia | Muridae | Arvicanthis niloticus | 15.500000 | -12.956000 | N6 | Granjon et al 2009 |
| BIB_1636 | Rodentia | Muridae | Arvicanthis niloticus | 14.890000 | -12.420000 | N6 | Granjon et al 2009 |
| BIB_1635 | Rodentia | Muridae | Arvicanthis niloticus | 16.303340 | -16.401357 | M2 | National Research Council 1981; Granjon and Duplantier 2009 |
| BIB_1634 | Rodentia | Muridae | Arvicanthis niloticus | 17.249855 | -10.667613 | L8 | Kirsch-Jung and Khtour 2007 |
| BIB_1631 | Rodentia | Muridae | Arvicanthis niloticus | 18.381180 | -11.716032 | J7 | Granjon et al 2009; Le Berre 1990 |
| BIB_1613 | Rodentia | Muridae | Arvicanthis niloticus | 16.300000 | -10.250000 | M9 | Granjon et al 2009 |
| BIB_1612 | Rodentia | Muridae | Arvicanthis niloticus | 15.000000 | -12.250000 | N7 | Granjon et al 1997 |
| BIB_1574 | Rodentia | Muridae | Arvicanthis niloticus | 16.607000 | -16.439323 | M2 | Granjon et al 1997, 2002; Dobigny et al 2013 |
| BIB_1567 | Rodentia | Muridae | Arvicanthis niloticus | 16.540090 | -10.801490 | L8 | Nickel 2003 |
| BIB_1538 | Rodentia | Muridae | Arvicanthis niloticus | 16.680556 | -9.301944 | L10 | Ba et al 2001 |
| BIB_1537 | Rodentia | Muridae | Arvicanthis niloticus | 16.680556 | -9.301944 | L10 | Denys et al 2001 |
| BIB_1686 | Rodentia | Muridae | Desmodilliscus braueri | 15.933333 | -12.000000 | M7 | Diatta et al 2015 |
| BIB_1685 | Rodentia | Muridae | Desmodilliscus braueri | 16.620000 | -14.290000 | L4 | Granjon et al 2009 |
| BIB_1684 | Rodentia | Muridae | Desmodilliscus braueri | 14.890000 | -12.420000 | N6 | Granjon et al 2009 |
| BIB_1683 | Rodentia | Muridae | Desmodilliscus braueri | 19.954817 | -14.429411 | I4 | Granjon et al 2009 |
| BIB_1682 | Rodentia | Muridae | Desmodilliscus braueri | 16.300000 | -10.250000 | M9 | Granjon et al 2009 |
| BIB_1681 | Rodentia | Muridae | Desmodilliscus braueri | 19.751600 | -14.428100 | I4 | Jullien and Petter 1970 |
| BIB_1679 | Rodentia | Muridae | Desmodilliscus braueri | 16.303340 | -16.401357 | M2 | Granjon et al 2009 |
| BIB_1678 | Rodentia | Muridae | Desmodilliscus braueri | 16.680556 | -9.301944 | L10 | Ba et al 2001 |
| BIB_1677 | Rodentia | Muridae | Desmodilliscus braueri | 16.680556 | -9.301944 | L10 | Denys et al 2001 |
| BIB_1641 | Rodentia | Muridae | Desmodilliscus braueri | 19.740000 | -14.370000 | I4 | Poulet 1974; Le Berre 1990 |
| 16031 | Rodentia | Muridae | Desmodilliscus braueri | 15.627513 | -11.320600 | N8 |  |
| 16018 | Rodentia | Muridae | Desmodilliscus braueri | 15.870257 | -11.529027 | M7 |  |
| BIB_1811 | Rodentia | Muridae | Gerbillus amoenus | 18.599848 | -16.079906 | J3 | Granjon et al 1997 |
| BIB_1802 | Rodentia | Muridae | Gerbillus amoenus | 15.933333 | -12.016667 | M7 | Diatta et al 2015 |
| BIB_1799 | Rodentia | Muridae | Gerbillus amoenus | 18.716667 | -15.600000 | J3 | Diatta et al 2015 |
| BIB_1798 | Rodentia | Muridae | Gerbillus amoenus | 20.000000 | -13.050000 | I6 | Diatta et al 2015 |
| BIB_1797 | Rodentia | Muridae | Gerbillus amoenus | 20.974000 | -15.892000 | H3 | Klein et al 1975 |
| BIB_1796 | Rodentia | Muridae | Gerbillus amoenus | 18.189955 | -16.007304 | K3 | Granjon et al 1997 |
| BIB_1795 | Rodentia | Muridae | Gerbillus amoenus | 18.238000 | -16.027000 | K3 | Klein et al 1975 |
| BIB_1793 | Rodentia | Muridae | Gerbillus amoenus | 18.801810 | -16.104548 | J3 | Peled et al 2016 |
| BIB_1785 | Rodentia | Muridae | Gerbillus amoenus | 18.115564 | -16.015721 | K3 | Ndiaye et al 2013 |
| BIB_1784 | Rodentia | Muridae | Gerbillus amoenus | 18.115564 | -16.015721 | K3 | Ndiaye et al 2012 |
| BIB_1783 | Rodentia | Muridae | Gerbillus amoenus | 18.115564 | -16.015721 | K3 | Chevret and Dobigny 2005 |
| BIB_1782 | Rodentia | Muridae | Gerbillus amoenus | 18.333000 | -15.967000 | K3 | Ndiaye et al 2016 |
| BIB_1781 | Rodentia | Muridae | Gerbillus amoenus | 18.200000 | -15.967000 | K3 | Ndiaye et al 2016 |
| BIB_1780 | Rodentia | Muridae | Gerbillus amoenus | 17.983333 | -12.233333 | K7 | Diatta et al 2015 |
| BIB_1779 | Rodentia | Muridae | Gerbillus amoenus | 18.689493 | -16.127390 | J2 | Peled et al 2016 |
| BIB_1778 | Rodentia | Muridae | Gerbillus amoenus | 18.650000 | -16.100000 | J3 | Diatta et al 2015 |
| BIB_1772 | Rodentia | Muridae | Gerbillus amoenus | 16.553000 | -16.234000 | M2 | Ndiaye et al 2016 |
| BIB_1770 | Rodentia | Muridae | Gerbillus amoenus | 19.751600 | -14.428100 | I4 | Jullien and Petter 1970 |
| BIB_1766 | Rodentia | Muridae | Gerbillus amoenus | 16.303340 | -16.401357 | M2 | Granjon et al 2009 |
| BIB_1755 | Rodentia | Muridae | Gerbillus amoenus | 16.364000 | -16.468000 | M2 | Ndiaye et al 2016 |
| BIB_1754 | Rodentia | Muridae | Gerbillus amoenus | 16.222172 | -16.490439 | M2 | Granjon et al 1997 |
| BIB_1753 | Rodentia | Muridae | Gerbillus amoenus | 19.600000 | -16.432000 | I2 | Ndiaye et al 2016 |
| BIB_1752 | Rodentia | Muridae | Gerbillus amoenus | 16.607000 | -16.439323 | M2 | Granjon et al 1997, 2002 |
| BIB_1701 | Rodentia | Muridae | Gerbillus amoenus | 20.460500 | -12.358300 | H6 | Dekeyser and Villiers 1956 |
| BIB_1700 | Rodentia | Muridae | Gerbillus amoenus | 20.831667 | -14.961024 | H4 | Granjon et al 1997 |
| BIB_1699 | Rodentia | Muridae | Gerbillus amoenus | 16.583000 | -9.583000 | L9 | Ndiaye et al 2016 |
| BIB_1698 | Rodentia | Muridae | Gerbillus amoenus | 19.740000 | -14.370000 | I4 | Peled et al 2016 |
| BIB_1697 | Rodentia | Muridae | Gerbillus amoenus | 19.740000 | -14.370000 | I4 | Poulet 1974; Granjon and Duplantier 2009 |
| BIB_1696 | Rodentia | Muridae | Gerbillus amoenus | 19.736000 | -14.367000 | I4 | Ndiaye et al 2016 |
| BIB_1695 | Rodentia | Muridae | Gerbillus amoenus | 19.733333 | -14.366667 | I4 | Diatta et al 2015 |
| 12115 | Rodentia | Muridae | Gerbillus amoenus | 20.880795 | -12.127885 | H7 |  |
| 12079 | Rodentia | Muridae | Gerbillus amoenus | 15.904015 | -11.936261 | M7 |  |
| 12078 | Rodentia | Muridae | Gerbillus amoenus | 15.904015 | -11.936261 | M7 |  |
| 12077 | Rodentia | Muridae | Gerbillus amoenus | 15.904015 | -11.936261 | M7 |  |
| 12063 | Rodentia | Muridae | Gerbillus amoenus | 16.608071 | -16.436528 | M2 | Boratyński et al 2017 |
| 10089 | Rodentia | Muridae | Gerbillus amoenus | 18.442821 | -11.387395 | J7 |  |
| 6546 | Rodentia | Muridae | Gerbillus amoenus | 20.265050 | -13.207592 | H6 | Boratyński et al 2017 |
| 6543 | Rodentia | Muridae | Gerbillus amoenus | 21.018148 | -11.924847 | H7 |  |
| 6536 | Rodentia | Muridae | Gerbillus amoenus | 21.018148 | -11.924847 | H7 | Boratyński et al 2017 |
| 6535 | Rodentia | Muridae | Gerbillus amoenus | 21.018148 | -11.924847 | H7 | Boratyński et al 2017 |
| 6521 | Rodentia | Muridae | Gerbillus amoenus | 21.519632 | -12.853367 | G6 | Boratyński et al 2017 |
| 6519 | Rodentia | Muridae | Gerbillus amoenus | 21.519632 | -12.853367 | G6 | Boratyński et al 2017 |
| BIB_1839 | Rodentia | Muridae | Gerbillus campestris | 18.783333 | -11.683333 | J7 | Diatta et al 2015 |
| BIB_1835 | Rodentia | Muridae | Gerbillus campestris | 20.000000 | -13.050000 | I6 | Diatta et al 2015 |
| BIB_1834 | Rodentia | Muridae | Gerbillus campestris | 17.983333 | -12.233333 | K7 | Diatta et al 2015 |
| BIB_1817 | Rodentia | Muridae | Gerbillus campestris | 20.266667 | -13.116667 | H6 | Diatta et al 2015 |
| BIB_1816 | Rodentia | Muridae | Gerbillus campestris | 20.527377 | -13.019975 | H6 | Granjon et al 2009 |
| 10117 | Rodentia | Muridae | Gerbillus campestris | 18.150508 | -12.065716 | K7 |  |
| 10116 | Rodentia | Muridae | Gerbillus campestris | 18.150508 | -12.065716 | K7 |  |
| 10115 | Rodentia | Muridae | Gerbillus campestris | 18.150508 | -12.065716 | K7 |  |
| 10114 | Rodentia | Muridae | Gerbillus campestris | 18.150508 | -12.065716 | K7 | Boratyński et al 2017 |
| 10108 | Rodentia | Muridae | Gerbillus campestris | 18.206234 | -11.730334 | K7 | Boratyński et al 2017 |
| 10107 | Rodentia | Muridae | Gerbillus campestris | 18.206040 | -11.730977 | K7 |  |
| 10106 | Rodentia | Muridae | Gerbillus campestris | 18.206040 | -11.730977 | K7 |  |
| 10105 | Rodentia | Muridae | Gerbillus campestris | 18.400353 | -11.814568 | J7 | Boratyński et al 2017 |
| 10104 | Rodentia | Muridae | Gerbillus campestris | 18.206040 | -11.730977 | K7 |  |
| 10103 | Rodentia | Muridae | Gerbillus campestris | 18.206040 | -11.730977 | K7 |  |
| 10095 | Rodentia | Muridae | Gerbillus campestris | 18.356822 | -11.816107 | J7 |  |
| 10091 | Rodentia | Muridae | Gerbillus campestris | 18.442821 | -11.387395 | J7 |  |
| 10090 | Rodentia | Muridae | Gerbillus campestris | 18.442821 | -11.387395 | J7 | Boratyński et al 2017 |
| 10086 | Rodentia | Muridae | Gerbillus campestris | 18.442821 | -11.387395 | J7 |  |
| 10085 | Rodentia | Muridae | Gerbillus campestris | 18.442821 | -11.387395 | J7 | Boratyński et al 2017 |
| 10071 | Rodentia | Muridae | Gerbillus campestris | 18.367468 | -9.048530 | J10 | Boratyński et al 2017 |
| 10059 | Rodentia | Muridae | Gerbillus campestris | 18.099577 | -8.010730 | K11 | Boratyński et al 2017 |
| 10054 | Rodentia | Muridae | Gerbillus campestris | 17.589160 | -7.445969 | K12 | Boratyński et al 2017 |
| 7988 | Rodentia | Muridae | Gerbillus campestris | 16.444294 | -11.778041 | M7 | Boratyński et al 2017 |
| 6569 | Rodentia | Muridae | Gerbillus campestris | 18.053485 | -11.942891 | K7 | Boratyński et al 2017 |
| 6564 | Rodentia | Muridae | Gerbillus campestris | 17.887298 | -12.110844 | K7 | Boratyński et al 2017 |
| 6545 | Rodentia | Muridae | Gerbillus campestris | 20.265050 | -13.207592 | H6 | Boratyński et al 2017 |
| 6544 | Rodentia | Muridae | Gerbillus campestris | 21.018148 | -11.924847 | H7 |  |
| 6540 | Rodentia | Muridae | Gerbillus campestris | 21.018148 | -11.924847 | H7 | Boratyński et al 2017 |
| 6538 | Rodentia | Muridae | Gerbillus campestris | 21.018148 | -11.924847 | H7 | Boratyński et al 2017 |
| 6532 | Rodentia | Muridae | Gerbillus campestris | 20.550798 | -12.676174 | H6 | Boratyński et al 2017 |
| 6525 | Rodentia | Muridae | Gerbillus campestris | 21.438097 | -12.980000 | G6 | Boratyński et al 2017 |
| 6524 | Rodentia | Muridae | Gerbillus campestris | 21.775207 | -12.879487 | G6 | Boratyński et al 2017 |
| 4901 | Rodentia | Muridae | Gerbillus campestris | 17.162042 | -12.128122 | L7 | Boratyński et al 2017 |
| 4454 | Rodentia | Muridae | Gerbillus campestris | 17.031593 | -16.272575 | L2 |  |
| BIB_2079 | Rodentia | Muridae | Gerbillus gerbillus | 22.116667 | -12.683333 | F6 | Diatta et al 2015 |
| BIB_2078 | Rodentia | Muridae | Gerbillus gerbillus | 22.133333 | -12.683333 | F6 | Ndiaye et al 2012 |
| BIB_2077 | Rodentia | Muridae | Gerbillus gerbillus | 18.419049 | -10.585766 | J8 | Peled et al 2016 |
| BIB_2076 | Rodentia | Muridae | Gerbillus gerbillus | 22.819879 | -12.734806 | F6 | Granjon et al 2009 |
| BIB_2075 | Rodentia | Muridae | Gerbillus gerbillus | 17.200000 | -16.066667 | L3 | Diatta et al 2015 |
| BIB_2069 | Rodentia | Muridae | Gerbillus gerbillus | 17.871812 | -9.309937 | K10 | Granjon et al 2009 |
| BIB_2068 | Rodentia | Muridae | Gerbillus gerbillus | 17.817089 | -11.004562 | K8 | Granjon et al 2009 |
| BIB_2067 | Rodentia | Muridae | Gerbillus gerbillus | 20.272200 | -13.120600 | H6 | Peled et al 2016 |
| BIB_2066 | Rodentia | Muridae | Gerbillus gerbillus | 20.974000 | -15.892000 | H3 | Klein et al 1975 |
| BIB_2011 | Rodentia | Muridae | Gerbillus gerbillus | 18.115564 | -16.015721 | K3 | Chevret and Dobigny 2005 |
| BIB_2010 | Rodentia | Muridae | Gerbillus gerbillus | 17.983333 | -12.233333 | K7 | Diatta et al 2015 |
| BIB_1892 | Rodentia | Muridae | Gerbillus gerbillus | 18.689493 | -16.127390 | J2 | Peled et al 2016 |
| BIB_1891 | Rodentia | Muridae | Gerbillus gerbillus | 18.671667 | -16.113333 | J3 | Ribas et al 2017 |
| BIB_1890 | Rodentia | Muridae | Gerbillus gerbillus | 18.650000 | -16.100000 | J3 | Diatta et al 2015 |
| BIB_1889 | Rodentia | Muridae | Gerbillus gerbillus | 16.566667 | -16.283333 | M2 | Diatta et al 2015 |
| BIB_1880 | Rodentia | Muridae | Gerbillus gerbillus | 16.303340 | -16.401357 | M2 | Granjon et al 2009 |
| BIB_1877 | Rodentia | Muridae | Gerbillus gerbillus | 20.891216 | -16.613172 | H2 | Granjon et al 2009 |
| BIB_1872 | Rodentia | Muridae | Gerbillus gerbillus | 20.460500 | -12.358300 | H6 | Dekeyser and Villiers 1956 |
| BIB_1857 | Rodentia | Muridae | Gerbillus gerbillus | 16.620000 | -14.290000 | L4 | Granjon et al 2009 |
| BIB_1856 | Rodentia | Muridae | Gerbillus gerbillus | 19.833333 | -15.916667 | I3 | Klein et al 1975 |
| BIB_1855 | Rodentia | Muridae | Gerbillus gerbillus | 20.083333 | -15.966667 | I3 | Klein et al 1975 |
| BIB_1854 | Rodentia | Muridae | Gerbillus gerbillus | 20.831667 | -14.961024 | H4 | Granjon et al 1997 |
| BIB_1848 | Rodentia | Muridae | Gerbillus gerbillus | 19.119974 | -16.226517 | J2 | Granjon et al 2009 |
| BIB_1847 | Rodentia | Muridae | Gerbillus gerbillus | 17.143650 | -16.164047 | L2 | Granjon et al 2009 |
| BIB_1841 | Rodentia | Muridae | Gerbillus gerbillus | 20.266667 | -13.116667 | H6 | Diatta et al 2015 |
| BIB_1840 | Rodentia | Muridae | Gerbillus gerbillus | 20.527377 | -13.019975 | H6 | Granjon et al 2009 |
| 12103 | Rodentia | Muridae | Gerbillus gerbillus | 19.071674 | -15.050074 | J4 |  |
| 12102 | Rodentia | Muridae | Gerbillus gerbillus | 19.071674 | -15.050074 | J4 | Boratyński et al 2017 |
| 12100 | Rodentia | Muridae | Gerbillus gerbillus | 19.114910 | -14.944218 | J4 |  |
| 12097 | Rodentia | Muridae | Gerbillus gerbillus | 19.114910 | -14.944218 | J4 |  |
| 12096 | Rodentia | Muridae | Gerbillus gerbillus | 19.114910 | -14.944218 | J4 |  |
| 12092 | Rodentia | Muridae | Gerbillus gerbillus | 18.880884 | -15.445370 | J3 | Boratyński et al 2017 |
| 10123 | Rodentia | Muridae | Gerbillus gerbillus | 20.730418 | -16.024828 | H3 | Boratyński et al 2017 |
| 10121 | Rodentia | Muridae | Gerbillus gerbillus | 20.730418 | -16.024828 | H3 |  |
| 10110 | Rodentia | Muridae | Gerbillus gerbillus | 18.206234 | -11.730334 | K7 |  |
| 10101 | Rodentia | Muridae | Gerbillus gerbillus | 18.206040 | -11.730977 | K7 |  |
| 10099 | Rodentia | Muridae | Gerbillus gerbillus | 18.206040 | -11.730977 | K7 |  |
| 10098 | Rodentia | Muridae | Gerbillus gerbillus | 18.206040 | -11.730977 | K7 |  |
| 10082 | Rodentia | Muridae | Gerbillus gerbillus | 18.447193 | -10.683335 | J8 |  |
| 10081 | Rodentia | Muridae | Gerbillus gerbillus | 18.447193 | -10.683335 | J8 |  |
| 10080 | Rodentia | Muridae | Gerbillus gerbillus | 18.447193 | -10.683335 | J8 |  |
| 10078 | Rodentia | Muridae | Gerbillus gerbillus | 18.447193 | -10.683335 | J8 |  |
| 10076 | Rodentia | Muridae | Gerbillus gerbillus | 18.447193 | -10.683335 | J8 |  |
| 10075 | Rodentia | Muridae | Gerbillus gerbillus | 18.578719 | -9.818611 | J9 |  |
| 10074 | Rodentia | Muridae | Gerbillus gerbillus | 18.578719 | -9.818611 | J9 |  |
| 10069 | Rodentia | Muridae | Gerbillus gerbillus | 18.367468 | -9.048530 | J10 | Boratyński et al 2017 |
| 10068 | Rodentia | Muridae | Gerbillus gerbillus | 18.367468 | -9.048530 | J10 |  |
| 10064 | Rodentia | Muridae | Gerbillus gerbillus | 18.383716 | -8.521678 | J10 |  |
| 10063 | Rodentia | Muridae | Gerbillus gerbillus | 18.383716 | -8.521678 | J10 |  |
| 10062 | Rodentia | Muridae | Gerbillus gerbillus | 18.383716 | -8.521678 | J10 |  |
| 10061 | Rodentia | Muridae | Gerbillus gerbillus | 18.380798 | -8.272197 | J11 | Boratyński et al 2017 |
| 10057 | Rodentia | Muridae | Gerbillus gerbillus | 18.099577 | -8.010730 | K11 | Boratyński et al 2017 |
| 10056 | Rodentia | Muridae | Gerbillus gerbillus | 18.099577 | -8.010730 | K11 |  |
| 10052 | Rodentia | Muridae | Gerbillus gerbillus | 16.484142 | -9.289591 | M10 |  |
| 10003 | Rodentia | Muridae | Gerbillus gerbillus | 20.130966 | -15.953221 | I3 |  |
| 10002 | Rodentia | Muridae | Gerbillus gerbillus | 20.844486 | -16.148816 | H2 | Boratyński et al 2017 |
| 7945 | Rodentia | Muridae | Gerbillus gerbillus | 20.099201 | -15.926960 | I3 | Boratyński et al 2017 |
| 7944 | Rodentia | Muridae | Gerbillus gerbillus | 20.375724 | -15.991165 | H3 | Boratyński et al 2017 |
| 6571 | Rodentia | Muridae | Gerbillus gerbillus | 20.863023 | -16.162677 | H2 | Boratyński et al 2017 |
| 6555 | Rodentia | Muridae | Gerbillus gerbillus | 19.740060 | -16.274618 | I2 | Boratyński et al 2017 |
| 6554 | Rodentia | Muridae | Gerbillus gerbillus | 19.740060 | -16.274618 | I2 | Boratyński et al 2017 |
| 6522 | Rodentia | Muridae | Gerbillus gerbillus | 21.519632 | -12.853367 | G6 |  |
| 6517 | Rodentia | Muridae | Gerbillus gerbillus | 21.197600 | -14.222085 | G4 | Boratyński et al 2017 |
| 6516 | Rodentia | Muridae | Gerbillus gerbillus | 21.197600 | -14.222085 | G4 | Boratyński et al 2017 |
| 6515 | Rodentia | Muridae | Gerbillus gerbillus | 21.197600 | -14.222085 | G4 | Boratyński et al 2017 |
| 6514 | Rodentia | Muridae | Gerbillus gerbillus | 21.242615 | -14.582155 | G4 | Boratyński et al 2017 |
| 5745 | Rodentia | Muridae | Gerbillus gerbillus | 21.280500 | -16.091700 | G2 | Boratyński et al 2017 |
| 5744 | Rodentia | Muridae | Gerbillus gerbillus | 21.280500 | -16.091700 | G2 | Boratyński et al 2017 |
| 5743 | Rodentia | Muridae | Gerbillus gerbillus | 21.280500 | -16.091700 | G2 | Boratyński et al 2017 |
| 5742 | Rodentia | Muridae | Gerbillus gerbillus | 21.280500 | -16.091700 | G2 |  |
| 5287 | Rodentia | Muridae | Gerbillus gerbillus | 20.679607 | -16.031008 | H3 |  |
| 5283 | Rodentia | Muridae | Gerbillus gerbillus | 20.471672 | -15.606627 | H3 |  |
| 5282 | Rodentia | Muridae | Gerbillus gerbillus | 20.471672 | -15.606627 | H3 |  |
| 5281 | Rodentia | Muridae | Gerbillus gerbillus | 20.471672 | -15.606627 | H3 | Boratyński et al 2017 |
| 5280 | Rodentia | Muridae | Gerbillus gerbillus | 20.471672 | -15.606627 | H3 |  |
| 5190 | Rodentia | Muridae | Gerbillus gerbillus | 19.851582 | -15.646828 | I3 | Boratyński et al 2017 |
| 5187 | Rodentia | Muridae | Gerbillus gerbillus | 20.611237 | -16.012710 | H3 | Boratyński et al 2017 |
| 5001 | Rodentia | Muridae | Gerbillus gerbillus | 18.919427 | -15.384957 | J3 | Boratyński et al 2017 |
| BIB_2082 | Rodentia | Muridae | Gerbillus henleyi | 18.783333 | -11.683333 | J7 | Diatta et al 2015 |
| BIB_2081 | Rodentia | Muridae | Gerbillus henleyi | 16.615280 | -11.402313 | L7 | Ndiaye et al 2014 |
| BIB_2080 | Rodentia | Muridae | Gerbillus henleyi | 19.740000 | -14.370000 | I4 | Ndiaye et al 2014 |
| 7860 | Rodentia | Muridae | Gerbillus henleyi | 16.763097 | -11.222535 | L8 | Boratyński et al 2017 |
| 5014 | Rodentia | Muridae | Gerbillus henleyi | 19.808792 | -14.288472 | I4 | Boratyński et al 2017 |
| 12073 | Rodentia | Muridae | Gerbillus nancillus | 15.957322 | -12.107513 | M7 |  |
| 12072 | Rodentia | Muridae | Gerbillus nancillus | 15.957322 | -12.107513 | M7 |  |
| 12062 | Rodentia | Muridae | Gerbillus nancillus | 16.608071 | -16.436528 | M2 |  |
| 4967 | Rodentia | Muridae | Gerbillus nancillus | 17.422828 | -13.435163 | L5 | Boratyński et al 2017 |
| BIB_2130 | Rodentia | Muridae | Gerbillus nigeriae | 17.984892 | -12.238730 | K7 | Granjon et al 2009 |
| BIB_2129 | Rodentia | Muridae | Gerbillus nigeriae | 16.643809 | -15.138664 | L4 | Granjon et al 2009 |
| BIB_2126 | Rodentia | Muridae | Gerbillus nigeriae | 18.115564 | -16.015721 | K3 | Granjon et al 2009 |
| BIB_2121 | Rodentia | Muridae | Gerbillus nigeriae | 19.712360 | -16.482097 | I2 | Ndiaye et al 2012 |
| BIB_2112 | Rodentia | Muridae | Gerbillus nigeriae | 16.303340 | -16.401357 | M2 | Granjon et al 2009 |
| BIB_2108 | Rodentia | Muridae | Gerbillus nigeriae | 16.607000 | -16.439323 | M2 | Granjon et al 2002 |
| BIB_2093 | Rodentia | Muridae | Gerbillus nigeriae | 16.620000 | -14.290000 | L4 | Granjon et al 2009 |
| 19826 | Rodentia | Muridae | Gerbillus nigeriae | 19.851327 | -16.357723 | I2 |  |
| 12111 | Rodentia | Muridae | Gerbillus nigeriae | 19.624224 | -13.006298 | I6 |  |
| 12061 | Rodentia | Muridae | Gerbillus nigeriae | 16.303468 | -16.407704 | M2 |  |
| 10045 | Rodentia | Muridae | Gerbillus nigeriae | 15.940344 | -9.456543 | M10 | Boratyński et al 2017 |
| 8009 | Rodentia | Muridae | Gerbillus nigeriae | 16.762937 | -11.221976 | L8 | Boratyński et al 2017 |
| 7984 | Rodentia | Muridae | Gerbillus nigeriae | 16.053281 | -11.669933 | M7 | Boratyński et al 2017 |
| 7979 | Rodentia | Muridae | Gerbillus nigeriae | 16.053281 | -11.669933 | M7 | Boratyński et al 2017 |
| 7978 | Rodentia | Muridae | Gerbillus nigeriae | 16.053281 | -11.669933 | M7 | Boratyński et al 2017 |
| 7962 | Rodentia | Muridae | Gerbillus nigeriae | 15.999440 | -11.872613 | M7 | Boratyński et al 2017 |
| 7961 | Rodentia | Muridae | Gerbillus nigeriae | 15.999440 | -11.872613 | M7 | Boratyński et al 2017 |
| 6553 | Rodentia | Muridae | Gerbillus nigeriae | 19.851327 | -16.357723 | I2 | Boratyński et al 2017 |
| 4970 | Rodentia | Muridae | Gerbillus nigeriae | 17.422828 | -13.435163 | L5 | Boratyński et al 2017 |
| 4582 | Rodentia | Muridae | Gerbillus nigeriae | 16.434780 | -14.036880 | M5 | Boratyński et al 2017 |
| 4581 | Rodentia | Muridae | Gerbillus nigeriae | 16.434780 | -14.036880 | M5 |  |
| BIB_2154 | Rodentia | Muridae | Gerbillus pyramidum | 20.403924 | -14.777287 | H4 | Granjon et al 2009 |
| BIB_2153 | Rodentia | Muridae | Gerbillus pyramidum | 19.751600 | -14.428100 | I4 | Jullien and Petter 1970 |
| BIB_2142 | Rodentia | Muridae | Gerbillus pyramidum | 19.740000 | -14.370000 | I4 | Poulet 1974 |
| BIB_2141 | Rodentia | Muridae | Gerbillus pyramidum | 19.733333 | -14.366667 | I4 | Diatta et al 2015 |
| BIB_2140 | Rodentia | Muridae | Gerbillus pyramidum | 20.266667 | -13.116667 | H6 | Diatta et al 2015 |
| BIB_2139 | Rodentia | Muridae | Gerbillus pyramidum | 20.527377 | -13.019975 | H6 | Granjon et al 2009 |
| 6542 | Rodentia | Muridae | Gerbillus pyramidum | 21.018148 | -11.924847 | H7 | Boratyński et al 2017 |
| 6541 | Rodentia | Muridae | Gerbillus pyramidum | 21.018148 | -11.924847 | H7 | Boratyński et al 2017 |
| 5013 | Rodentia | Muridae | Gerbillus pyramidum | 19.808792 | -14.288472 | I4 | Boratyński et al 2017 |
| BIB_3898 | Rodentia | Muridae | Gerbillus sp. | 18.783333 | -11.683333 | J7 | Diatta et al 2015 |
| BIB_3884 | Rodentia | Muridae | Gerbillus sp. | 18.716667 | -15.600000 | J3 | Diatta et al 2015 |
| BIB_3877 | Rodentia | Muridae | Gerbillus sp. | 17.383333 | -16.050000 | L3 | Diatta et al 2015 |
| BIB_3621 | Rodentia | Muridae | Gerbillus sp. | 17.983333 | -12.233333 | K7 | Diatta et al 2015 |
| BIB_3487 | Rodentia | Muridae | Gerbillus sp. | 22.983333 | -12.000000 | E7 | Diatta et al 2015 |
| BIB_2748 | Rodentia | Muridae | Gerbillus sp. | 16.540090 | -10.801490 | L8 | Nickel 2003 |
| BIB_2340 | Rodentia | Muridae | Gerbillus sp. | 19.733333 | -14.366667 | I4 | Diatta et al 2015 |
| BIB_2339 | Rodentia | Muridae | Gerbillus sp. | 20.266667 | -13.116667 | H6 | Diatta et al 2015 |
| 19833 | Rodentia | Muridae | Gerbillus sp. | 20.472437 | -16.050332 | H3 |  |
| 19829 | Rodentia | Muridae | Gerbillus sp. | 16.384200 | -16.482462 | M2 |  |
| 19828 | Rodentia | Muridae | Gerbillus sp. | 16.385520 | -16.481824 | M2 |  |
| 19827 | Rodentia | Muridae | Gerbillus sp. | 16.414962 | -16.466327 | M2 |  |
| 19824 | Rodentia | Muridae | Gerbillus sp. | 20.473720 | -16.040907 | H3 |  |
| 19823 | Rodentia | Muridae | Gerbillus sp. | 20.473680 | -16.040777 | H3 |  |
| 19822 | Rodentia | Muridae | Gerbillus sp. | 20.590115 | -16.117913 | H2 |  |
| 19821 | Rodentia | Muridae | Gerbillus sp. | 20.591287 | -16.116292 | H2 |  |
| 19820 | Rodentia | Muridae | Gerbillus sp. | 20.587382 | -16.126798 | H2 |  |
| 19018 | Rodentia | Muridae | Gerbillus sp. | 16.567450 | -11.533010 | L7 |  |
| 19009 | Rodentia | Muridae | Gerbillus sp. | 16.544140 | -10.750440 | L8 |  |
| 18989 | Rodentia | Muridae | Gerbillus sp. | 16.002553 | -11.871748 | M7 |  |
| 18971 | Rodentia | Muridae | Gerbillus sp. | 16.362143 | -16.489483 | M2 |  |
| 18870 | Rodentia | Muridae | Gerbillus sp. | 16.567450 | -11.533010 | L7 |  |
| 18819 | Rodentia | Muridae | Gerbillus sp. | 16.538033 | -10.741550 | L8 |  |
| 18818 | Rodentia | Muridae | Gerbillus sp. | 16.538033 | -10.741550 | L8 |  |
| 18574 | Rodentia | Muridae | Gerbillus sp. | 16.279653 | -16.515098 | M2 |  |
| 15828 | Rodentia | Muridae | Gerbillus sp. | 21.283977 | -15.446167 | G3 |  |
| 13915 | Rodentia | Muridae | Gerbillus sp. | 17.125720 | -15.983043 | L3 |  |
| 13858 | Rodentia | Muridae | Gerbillus sp. | 16.855623 | -12.255068 | L7 |  |
| 13842 | Rodentia | Muridae | Gerbillus sp. | 18.120092 | -11.963288 | K7 |  |
| 13838 | Rodentia | Muridae | Gerbillus sp. | 18.206040 | -11.730977 | K7 |  |
| 13837 | Rodentia | Muridae | Gerbillus sp. | 18.206040 | -11.730977 | K7 |  |
| 13836 | Rodentia | Muridae | Gerbillus sp. | 18.206040 | -11.730977 | K7 |  |
| 13835 | Rodentia | Muridae | Gerbillus sp. | 18.206040 | -11.730977 | K7 |  |
| 13751 | Rodentia | Muridae | Gerbillus sp. | 23.829695 | -10.585353 | D8 |  |
| 13750 | Rodentia | Muridae | Gerbillus sp. | 23.829695 | -10.585353 | D8 |  |
| 13749 | Rodentia | Muridae | Gerbillus sp. | 23.829695 | -10.585353 | D8 |  |
| 13748 | Rodentia | Muridae | Gerbillus sp. | 23.829695 | -10.585353 | D8 |  |
| 13747 | Rodentia | Muridae | Gerbillus sp. | 23.829695 | -10.585353 | D8 |  |
| 13746 | Rodentia | Muridae | Gerbillus sp. | 23.829695 | -10.585353 | D8 |  |
| 13745 | Rodentia | Muridae | Gerbillus sp. | 23.829695 | -10.585353 | D8 |  |
| 13744 | Rodentia | Muridae | Gerbillus sp. | 23.829695 | -10.585353 | D8 |  |
| 13723 | Rodentia | Muridae | Gerbillus sp. | 24.810632 | -10.536038 | C8 |  |
| 13704 | Rodentia | Muridae | Gerbillus sp. | 25.110560 | -11.520470 | C7 |  |
| 13688 | Rodentia | Muridae | Gerbillus sp. | 24.932358 | -11.466057 | C7 |  |
| 13687 | Rodentia | Muridae | Gerbillus sp. | 24.616738 | -11.394455 | D7 |  |
| 13682 | Rodentia | Muridae | Gerbillus sp. | 24.573660 | -11.386578 | D7 |  |
| 13666 | Rodentia | Muridae | Gerbillus sp. | 23.871750 | -11.226817 | D8 |  |
| 13622 | Rodentia | Muridae | Gerbillus sp. | 21.265030 | -15.114152 | G3 |  |
| 12392 | Rodentia | Muridae | Gerbillus sp. | 17.070297 | -12.207848 | L7 |  |
| 12317 | Rodentia | Muridae | Gerbillus sp. | 17.571290 | -12.178565 | K7 |  |
| 12316 | Rodentia | Muridae | Gerbillus sp. | 17.571290 | -12.178565 | K7 |  |
| 12112 | Rodentia | Muridae | Gerbillus sp. | 19.773785 | -13.032954 | I6 |  |
| 12098 | Rodentia | Muridae | Gerbillus sp. | 19.114910 | -14.944218 | J4 |  |
| 12094 | Rodentia | Muridae | Gerbillus sp. | 18.924168 | -15.374332 | J3 |  |
| 12093 | Rodentia | Muridae | Gerbillus sp. | 18.882735 | -15.442722 | J3 |  |
| 12067 | Rodentia | Muridae | Gerbillus sp. | 16.436544 | -16.461979 | M2 |  |
| 11417 | Rodentia | Muridae | Gerbillus sp. | 16.303097 | -16.402640 | M2 |  |
| 11416 | Rodentia | Muridae | Gerbillus sp. | 18.489588 | -14.643788 | J4 |  |
| 11404 | Rodentia | Muridae | Gerbillus sp. | 18.958312 | -13.348447 | J5 |  |
| 11362 | Rodentia | Muridae | Gerbillus sp. | 16.774732 | -11.672555 | L7 |  |
| 11329 | Rodentia | Muridae | Gerbillus sp. | 16.517918 | -9.881345 | L9 |  |
| 10845 | Rodentia | Muridae | Gerbillus sp. | 15.013457 | -12.476630 | N6 |  |
| 10120 | Rodentia | Muridae | Gerbillus sp. | 20.730418 | -16.024828 | H3 |  |
| 10112 | Rodentia | Muridae | Gerbillus sp. | 17.982002 | -11.944523 | K7 |  |
| 10050 | Rodentia | Muridae | Gerbillus sp. | 16.484142 | -9.289591 | M10 |  |
| 10049 | Rodentia | Muridae | Gerbillus sp. | 16.499776 | -9.564642 | M9 |  |
| 10048 | Rodentia | Muridae | Gerbillus sp. | 16.499776 | -9.564642 | M9 |  |
| 10047 | Rodentia | Muridae | Gerbillus sp. | 16.499776 | -9.564642 | M9 |  |
| 10046 | Rodentia | Muridae | Gerbillus sp. | 16.499776 | -9.564642 | M9 |  |
| 10044 | Rodentia | Muridae | Gerbillus sp. | 15.940344 | -9.456543 | M10 |  |
| 10016 | Rodentia | Muridae | Gerbillus sp. | 16.385851 | -11.376121 | M7 |  |
| 10013 | Rodentia | Muridae | Gerbillus sp. | 16.160563 | -10.944397 | M8 |  |
| 10012 | Rodentia | Muridae | Gerbillus sp. | 16.160563 | -10.944397 | M8 |  |
| 10011 | Rodentia | Muridae | Gerbillus sp. | 16.160563 | -10.944397 | M8 |  |
| 10009 | Rodentia | Muridae | Gerbillus sp. | 16.077534 | -11.018243 | M8 |  |
| 9998 | Rodentia | Muridae | Gerbillus sp. | 18.356815 | -11.816080 | J7 |  |
| 9992 | Rodentia | Muridae | Gerbillus sp. | 18.356815 | -11.816080 | J7 |  |
| 9990 | Rodentia | Muridae | Gerbillus sp. | 18.367478 | -9.048450 | J10 |  |
| 9985 | Rodentia | Muridae | Gerbillus sp. | 18.150443 | -12.065303 | K7 |  |
| 9983 | Rodentia | Muridae | Gerbillus sp. | 18.206040 | -11.730977 | K7 |  |
| 9982 | Rodentia | Muridae | Gerbillus sp. | 18.356815 | -11.816080 | J7 |  |
| 9981 | Rodentia | Muridae | Gerbillus sp. | 18.442815 | -11.387390 | J7 |  |
| 9979 | Rodentia | Muridae | Gerbillus sp. | 18.578522 | -9.818463 | J9 |  |
| 9978 | Rodentia | Muridae | Gerbillus sp. | 18.367478 | -9.048450 | J10 |  |
| 9977 | Rodentia | Muridae | Gerbillus sp. | 18.383822 | -8.521617 | J10 |  |
| 9972 | Rodentia | Muridae | Gerbillus sp. | 18.356815 | -11.816080 | J7 |  |
| 9967 | Rodentia | Muridae | Gerbillus sp. | 18.150443 | -12.065303 | K7 |  |
| 9966 | Rodentia | Muridae | Gerbillus sp. | 18.356815 | -11.816080 | J7 |  |
| 9957 | Rodentia | Muridae | Gerbillus sp. | 18.356815 | -11.816080 | J7 |  |
| 9955 | Rodentia | Muridae | Gerbillus sp. | 18.447222 | -10.683298 | J8 |  |
| 9953 | Rodentia | Muridae | Gerbillus sp. | 18.383822 | -8.521617 | J10 |  |
| 9944 | Rodentia | Muridae | Gerbillus sp. | 18.442815 | -11.387390 | J7 |  |
| 9937 | Rodentia | Muridae | Gerbillus sp. | 18.356815 | -11.816080 | J7 |  |
| 9934 | Rodentia | Muridae | Gerbillus sp. | 18.442815 | -11.387390 | J7 |  |
| 9932 | Rodentia | Muridae | Gerbillus sp. | 18.367478 | -9.048450 | J10 |  |
| 9928 | Rodentia | Muridae | Gerbillus sp. | 18.447222 | -10.683298 | J8 |  |
| 9926 | Rodentia | Muridae | Gerbillus sp. | 16.484132 | -9.289523 | M10 |  |
| 9635 | Rodentia | Muridae | Gerbillus sp. | 16.484132 | -9.289523 | M10 |  |
| 9317 | Rodentia | Muridae | Gerbillus sp. | 16.182943 | -10.950508 | M8 |  |
| 9244 | Rodentia | Muridae | Gerbillus sp. | 15.041525 | -12.303785 | N7 |  |
| 8019 | Rodentia | Muridae | Gerbillus sp. | 20.350461 | -15.989710 | H3 |  |
| 7076 | Rodentia | Muridae | Gerbillus sp. | 20.538690 | -12.231090 | H7 |  |
| 6512 | Rodentia | Muridae | Gerbillus sp. | 20.739577 | -16.415012 | H2 |  |
| 6511 | Rodentia | Muridae | Gerbillus sp. | 19.422068 | -16.297312 | I2 |  |
| 6508 | Rodentia | Muridae | Gerbillus sp. | 19.567590 | -16.383732 | I2 |  |
| 6504 | Rodentia | Muridae | Gerbillus sp. | 17.846344 | -12.078243 | K7 |  |
| 6503 | Rodentia | Muridae | Gerbillus sp. | 17.363940 | -13.572142 | L5 |  |
| 6500 | Rodentia | Muridae | Gerbillus sp. | 16.339388 | -12.505163 | M6 |  |
| 6494 | Rodentia | Muridae | Gerbillus sp. | 20.944458 | -16.549482 | H2 |  |
| 6492 | Rodentia | Muridae | Gerbillus sp. | 19.959787 | -16.084098 | I3 |  |
| 6488 | Rodentia | Muridae | Gerbillus sp. | 21.018148 | -11.924847 | H7 |  |
| 6486 | Rodentia | Muridae | Gerbillus sp. | 21.519632 | -12.853367 | G6 |  |
| 6476 | Rodentia | Muridae | Gerbillus sp. | 20.944458 | -16.549482 | H2 |  |
| 6423 | Rodentia | Muridae | Gerbillus sp. | 20.739577 | -16.415012 | H2 |  |
| 6422 | Rodentia | Muridae | Gerbillus sp. | 20.739577 | -16.415012 | H2 |  |
| 6420 | Rodentia | Muridae | Gerbillus sp. | 20.642713 | -16.258352 | H2 |  |
| 6418 | Rodentia | Muridae | Gerbillus sp. | 20.489627 | -16.226523 | H2 |  |
| 6402 | Rodentia | Muridae | Gerbillus sp. | 20.420480 | -16.276895 | H2 |  |
| 6401 | Rodentia | Muridae | Gerbillus sp. | 20.414198 | -16.168990 | H2 |  |
| 6400 | Rodentia | Muridae | Gerbillus sp. | 20.394535 | -16.124797 | H2 |  |
| 6385 | Rodentia | Muridae | Gerbillus sp. | 20.101025 | -16.165552 | I2 |  |
| 6373 | Rodentia | Muridae | Gerbillus sp. | 20.084585 | -16.131387 | I2 |  |
| 6371 | Rodentia | Muridae | Gerbillus sp. | 20.056738 | -16.099315 | I2 |  |
| 6370 | Rodentia | Muridae | Gerbillus sp. | 20.056738 | -16.099315 | I2 |  |
| 6366 | Rodentia | Muridae | Gerbillus sp. | 19.980887 | -16.101692 | I2 |  |
| 6358 | Rodentia | Muridae | Gerbillus sp. | 19.845550 | -16.201477 | I2 |  |
| 6355 | Rodentia | Muridae | Gerbillus sp. | 19.794213 | -16.210145 | I2 |  |
| 6351 | Rodentia | Muridae | Gerbillus sp. | 19.781952 | -16.188017 | I2 |  |
| 6335 | Rodentia | Muridae | Gerbillus sp. | 19.787547 | -16.151107 | I2 |  |
| 6316 | Rodentia | Muridae | Gerbillus sp. | 19.679942 | -16.290316 | I2 |  |
| 6305 | Rodentia | Muridae | Gerbillus sp. | 19.584293 | -16.351473 | I2 |  |
| 6294 | Rodentia | Muridae | Gerbillus sp. | 19.422068 | -16.297312 | I2 |  |
| 6260 | Rodentia | Muridae | Gerbillus sp. | 15.742177 | -13.240178 | M6 |  |
| 6147 | Rodentia | Muridae | Gerbillus sp. | 15.727673 | -12.472117 | M6 |  |
| 6128 | Rodentia | Muridae | Gerbillus sp. | 16.339388 | -12.505163 | M6 |  |
| 6070 | Rodentia | Muridae | Gerbillus sp. | 17.649985 | -11.395480 | K7 |  |
| 6055 | Rodentia | Muridae | Gerbillus sp. | 18.258250 | -11.513437 | K7 |  |
| 6054 | Rodentia | Muridae | Gerbillus sp. | 18.258250 | -11.513437 | K7 |  |
| 5761 | Rodentia | Muridae | Gerbillus sp. | 21.197600 | -14.222085 | G4 |  |
| 5314 | Rodentia | Muridae | Gerbillus sp. | 15.962793 | -12.681060 | M6 |  |
| 3636 | Rodentia | Muridae | Gerbillus sp. | 16.403148 | -9.559860 | M9 |  |
| 3519 | Rodentia | Muridae | Gerbillus sp. | 17.372698 | -11.915772 | L7 |  |
| 529 | Rodentia | Muridae | Gerbillus sp. | 21.290150 | -16.029133 | G3 |  |
| 525 | Rodentia | Muridae | Gerbillus sp. | 21.189650 | -13.950167 | G5 |  |
| 522 | Rodentia | Muridae | Gerbillus sp. | 20.747567 | -13.140867 | H6 |  |
| 508 | Rodentia | Muridae | Gerbillus sp. | 19.444867 | -14.748900 | I4 |  |
| 507 | Rodentia | Muridae | Gerbillus sp. | 19.444867 | -14.748900 | I4 |  |
| 497 | Rodentia | Muridae | Gerbillus sp. | 17.785250 | -16.041700 | K3 |  |
| BIB_4368 | Rodentia | Muridae | Gerbillus tarabuli | 22.116667 | -12.683333 | F6 | Diatta et al 2015 |
| BIB_4367 | Rodentia | Muridae | Gerbillus tarabuli | 17.203333 | -16.075556 | L3 | Ribas et al 2017 |
| BIB_4365 | Rodentia | Muridae | Gerbillus tarabuli | 17.200000 | -16.066667 | L3 | Diatta et al 2015 |
| BIB_4358 | Rodentia | Muridae | Gerbillus tarabuli | 17.984892 | -12.238730 | K7 | Granjon et al 2009 |
| BIB_4357 | Rodentia | Muridae | Gerbillus tarabuli | 16.643809 | -15.138664 | L4 | Granjon et al 2009 |
| BIB_4355 | Rodentia | Muridae | Gerbillus tarabuli | 20.000000 | -13.050000 | I6 | Diatta et al 2015 |
| BIB_4354 | Rodentia | Muridae | Gerbillus tarabuli | 17.383333 | -16.050000 | L3 | Diatta et al 2015 |
| BIB_4342 | Rodentia | Muridae | Gerbillus tarabuli | 18.115564 | -16.015721 | K3 | Granjon et al 2009 |
| BIB_4337 | Rodentia | Muridae | Gerbillus tarabuli | 21.068914 | -16.992276 | H2 | Granjon et al 2009 |
| BIB_4335 | Rodentia | Muridae | Gerbillus tarabuli | 17.983333 | -12.233333 | K7 | Diatta et al 2015 |
| BIB_4320 | Rodentia | Muridae | Gerbillus tarabuli | 18.650000 | -16.100000 | J3 | Diatta et al 2015 |
| BIB_4319 | Rodentia | Muridae | Gerbillus tarabuli | 20.403924 | -14.777287 | H4 | Granjon et al 2009 |
| BIB_4318 | Rodentia | Muridae | Gerbillus tarabuli | 16.300000 | -10.250000 | M9 | Granjon et al 2009 |
| BIB_4313 | Rodentia | Muridae | Gerbillus tarabuli | 16.303340 | -16.401357 | M2 | Granjon et al 2009 |
| BIB_4311 | Rodentia | Muridae | Gerbillus tarabuli | 16.607000 | -16.439323 | M2 | Granjon et al 2002 |
| BIB_4304 | Rodentia | Muridae | Gerbillus tarabuli | 16.620000 | -14.290000 | L4 | Granjon et al 2009 |
| BIB_4297 | Rodentia | Muridae | Gerbillus tarabuli | 16.583333 | -9.583333 | L9 | Ndiaye et al 2012 |
| BIB_4289 | Rodentia | Muridae | Gerbillus tarabuli | 19.733333 | -14.366667 | I4 | Diatta et al 2015 |
| BIB_4288 | Rodentia | Muridae | Gerbillus tarabuli | 20.266667 | -13.116667 | H6 | Diatta et al 2015 |
| BIB_4284 | Rodentia | Muridae | Gerbillus tarabuli | 20.527377 | -13.019975 | H6 | Granjon et al 2009 |
| 12101 | Rodentia | Muridae | Gerbillus tarabuli | 19.167552 | -14.973780 | J4 | Boratyński et al 2017 |
| 12091 | Rodentia | Muridae | Gerbillus tarabuli | 18.826371 | -15.492424 | J3 | Boratyński et al 2017 |
| 10077 | Rodentia | Muridae | Gerbillus tarabuli | 18.447193 | -10.683335 | J8 | Boratyński et al 2017 |
| 10051 | Rodentia | Muridae | Gerbillus tarabuli | 16.484142 | -9.289591 | M10 | Boratyński et al 2017 |
| 8020 | Rodentia | Muridae | Gerbillus tarabuli | 20.692514 | -16.039001 | H3 | Boratyński et al 2017 |
| 6560 | Rodentia | Muridae | Gerbillus tarabuli | 17.578499 | -12.881094 | K6 | Boratyński et al 2017 |
| 6559 | Rodentia | Muridae | Gerbillus tarabuli | 17.363940 | -13.572142 | L5 | Boratyński et al 2017 |
| 6557 | Rodentia | Muridae | Gerbillus tarabuli | 18.795105 | -16.138979 | J2 | Boratyński et al 2017 |
| 6539 | Rodentia | Muridae | Gerbillus tarabuli | 21.018148 | -11.924847 | H7 | Boratyński et al 2017 |
| 6537 | Rodentia | Muridae | Gerbillus tarabuli | 21.018148 | -11.924847 | H7 | Boratyński et al 2017 |
| 6523 | Rodentia | Muridae | Gerbillus tarabuli | 21.519632 | -12.853367 | G6 | Boratyński et al 2017 |
| 5747 | Rodentia | Muridae | Gerbillus tarabuli | 21.280500 | -16.091700 | G2 | Boratyński et al 2017 |
| 5285 | Rodentia | Muridae | Gerbillus tarabuli | 20.561312 | -16.013968 | H3 | Boratyński et al 2017 |
| 5151 | Rodentia | Muridae | Gerbillus tarabuli | 20.554890 | -16.155358 | H2 | Boratyński et al 2017 |
| 4972 | Rodentia | Muridae | Gerbillus tarabuli | 17.422828 | -13.435163 | L5 | Boratyński et al 2017 |
| 4971 | Rodentia | Muridae | Gerbillus tarabuli | 17.422828 | -13.435163 | L5 | Boratyński et al 2017 |
| 4969 | Rodentia | Muridae | Gerbillus tarabuli | 17.422828 | -13.435163 | L5 | Boratyński et al 2017 |
| 4756 | Rodentia | Muridae | Gerbillus tarabuli | 15.641847 | -12.109977 | N7 | Boratyński et al 2017 |
| 4583 | Rodentia | Muridae | Gerbillus tarabuli | 16.434780 | -14.036880 | M5 | Boratyński et al 2017 |
| 4455 | Rodentia | Muridae | Gerbillus tarabuli | 17.031593 | -16.272575 | L2 | Boratyński et al 2017 |
| BIB_4376 | Rodentia | Muridae | Mastomys erythroleucus | 15.933333 | -12.016667 | M7 | Diatta et al 2015 |
| BIB_4375 | Rodentia | Muridae | Mastomys erythroleucus | 16.620000 | -14.290000 | L4 | Granjon et al 2009 |
| BIB_4374 | Rodentia | Muridae | Mastomys erythroleucus | 15.500000 | -12.956000 | N6 | Granjon et al 2009 |
| BIB_4373 | Rodentia | Muridae | Mastomys erythroleucus | 14.890000 | -12.420000 | N6 | Granjon et al 2009 |
| BIB_4371 | Rodentia | Muridae | Mastomys erythroleucus | 15.736325 | -11.964514 | M7 | Granjon et al 2009 |
| BIB_4370 | Rodentia | Muridae | Mastomys erythroleucus | 16.303340 | -16.401357 | M2 | Granjon et al 2009 |
| BIB_4381 | Rodentia | Muridae | Mastomys huberti | 16.620000 | -14.290000 | L4 | Granjon et al 2009 |
| BIB_4380 | Rodentia | Muridae | Mastomys huberti | 16.303340 | -16.401357 | M2 | Granjon et al 2009 |
| 19908 | Rodentia | Muridae | Mastomys huberti | 16.337420 | -16.397962 | M2 |  |
| BIB_4461 | Rodentia | Muridae | Meriones crassus | 16.538033 | -10.741550 | L8 | Nickel 2003 |
| BIB_4460 | Rodentia | Muridae | Meriones crassus | 20.403924 | -14.777287 | H4 | Granjon et al 2009 |
| BIB_4459 | Rodentia | Muridae | Meriones crassus | 19.751600 | -14.428100 | I4 | Jullien and Petter 1970 |
| BIB_4431 | Rodentia | Muridae | Meriones crassus | 20.878187 | -16.716230 | H2 | Granjon et al 2009 |
| BIB_4426 | Rodentia | Muridae | Meriones crassus | 19.740000 | -14.370000 | I4 | Poulet 1974 |
| BIB_4462 | Rodentia | Muridae | Meriones libycus | 20.687000 | -16.674000 | H2 | Granjon et al 1997 |
| BIB_4500 | Rodentia | Muridae | Mus musculus | 16.179554 | -13.819628 | M5 | Granjon et al 2009 |
| BIB_4499 | Rodentia | Muridae | Mus musculus | 20.000000 | -13.050000 | I6 | Diatta et al 2015 |
| BIB_4492 | Rodentia | Muridae | Mus musculus | 18.115564 | -16.015721 | K3 | Granjon et al 1997, 2009 |
| BIB_4489 | Rodentia | Muridae | Mus musculus | 16.303340 | -16.401357 | M2 | Granjon et al 2009 |
| BIB_4479 | Rodentia | Muridae | Mus musculus | 16.222172 | -16.490439 | M2 | Granjon et al 1997 |
| BIB_4475 | Rodentia | Muridae | Mus musculus | 16.607000 | -16.439323 | M2 | Granjon et al 1997 |
| BIB_4469 | Rodentia | Muridae | Mus musculus | 20.536743 | -12.961179 | H6 | Granjon et al 2009 |
| BIB_4468 | Rodentia | Muridae | Mus musculus | 20.536743 | -12.961179 | H6 | Dekeyser and Villiers 1956; Le Berre 1990 |
| 6558 | Rodentia | Muridae | Mus musculus | 18.130414 | -15.936053 | K3 |  |
| BIB_4537 | Rodentia | Muridae | Pachyuromys duprasi | 23.810833 | -11.455000 | D7 | Padial and Ibáñez 2005 |
| BIB_4536 | Rodentia | Muridae | Pachyuromys duprasi | 20.403924 | -14.777287 | H4 | Granjon et al 2009 |
| BIB_4505 | Rodentia | Muridae | Pachyuromys duprasi | 19.740000 | -14.370000 | I4 | Poulet 1974 |
| BIB_4504 | Rodentia | Muridae | Pachyuromys duprasi | 19.733333 | -14.366667 | I4 | Diatta et al 2015 |
| 13676 | Rodentia | Muridae | Pachyuromys duprasi | 24.462253 | -11.351480 | D7 |  |
| 5184 | Rodentia | Muridae | Pachyuromys duprasi | 20.775360 | -16.381225 | H2 |  |
| BIB_4540 | Rodentia | Muridae | Praomys cf daltoni | 16.541500 | -10.747200 | L8 | Mikula et al 2020 |
| BIB_4539 | Rodentia | Muridae | Praomys cf daltoni | 16.541500 | -10.747200 | L8 | Mikula et al 2020 |
| BIB_4538 | Rodentia | Muridae | Praomys cf daltoni | 16.543000 | -10.746900 | L8 | Mikula et al 2020 |
| 16010 | Rodentia | Muridae | Praomys cf daltoni | 16.002553 | -11.871748 | M7 |  |
| 12739 | Rodentia | Muridae | Praomys cf daltoni | 16.923777 | -10.593842 | L8 | Mikula et al 2020 |
| 10008 | Rodentia | Muridae | Praomys cf daltoni | 15.898938 | -11.339962 | M8 | Mikula et al 2020 |
| 9360 | Rodentia | Muridae | Praomys cf daltoni | 16.489543 | -11.057988 | M8 | Mikula et al 2020 |
| BIB_4568 | Rodentia | Muridae | Psammomys obesus | 18.599848 | -16.079906 | J3 | Granjon et al 1997 |
| BIB_4567 | Rodentia | Muridae | Psammomys obesus | 20.083333 | -15.666667 | I3 | Klein et al 1975 |
| BIB_4566 | Rodentia | Muridae | Psammomys obesus | 19.583333 | -16.083333 | I3 | Klein et al 1975 |
| BIB_4565 | Rodentia | Muridae | Psammomys obesus | 18.189955 | -16.007304 | K3 | Granjon et al 1997 |
| BIB_4561 | Rodentia | Muridae | Psammomys obesus | 18.115564 | -16.015721 | K3 | Granjon et al 2009 |
| BIB_4558 | Rodentia | Muridae | Psammomys obesus | 21.068914 | -16.992276 | H2 | Granjon et al 2009; Aulagnier et al 2017 |
| BIB_4554 | Rodentia | Muridae | Psammomys obesus | 20.403924 | -14.777287 | H4 | Granjon et al 2009 |
| BIB_4553 | Rodentia | Muridae | Psammomys obesus | 19.666667 | -15.966667 | I3 | Klein et al 1975 |
| BIB_4549 | Rodentia | Muridae | Psammomys obesus | 19.379495 | -16.522164 | I2 | Klein et al 1975 |
| BIB_4548 | Rodentia | Muridae | Psammomys obesus | 20.500000 | -14.916667 | H4 | Klein et al 1975 |
| BIB_4547 | Rodentia | Muridae | Psammomys obesus | 19.666667 | -16.166667 | I2 | Klein et al 1975 |
| BIB_4573 | Rodentia | Muridae | Rattus rattus | 16.303340 | -16.401357 | M2 | National Research Council 1981 |
| BIB_4571 | Rodentia | Muridae | Rattus rattus | 21.068914 | -16.992276 | H2 | Aulagnier and Thévenot 1986; Le Berre 1990; Aulagnier et al 2017 |
| BIB_4686 | Rodentia | Muridae | Taterillus arenarius | 16.952341 | -16.245387 | L2 | Granjon et al 2009 |
| BIB_4636 | Rodentia | Muridae | Taterillus arenarius | 17.709852 | -12.408925 | K6 | Granjon et al 2009 |
| BIB_4620 | Rodentia | Muridae | Taterillus arenarius | 16.566667 | -16.283333 | M2 | Diatta et al 2015 |
| BIB_4609 | Rodentia | Muridae | Taterillus arenarius | 19.342856 | -14.614389 | I4 | Granjon et al 2009 |
| BIB_4595 | Rodentia | Muridae | Taterillus arenarius | 16.303340 | -16.401357 | M2 | Granjon et al 2009 |
| BIB_4594 | Rodentia | Muridae | Taterillus arenarius | 16.222172 | -16.490439 | M2 | Granjon et al 1997 |
| BIB_4687 | Rodentia | Muridae | Taterillus gracilis | 19.751600 | -14.428100 | I4 | Jullien and Petter 1970 |
| 12070 | Rodentia | Muridae | Taterillus gracilis | 15.480686 | -12.305768 | N7 |  |
| BIB_4709 | Rodentia | Muridae | Taterillus tranieri | 16.300000 | -10.250000 | M9 | Granjon et al 2009 |
| BIB_4708 | Rodentia | Muridae | Taterillus tranieri | 16.425376 | -9.630707 | M9 | Dobigny et al 2005 |
| BIB_4740 | Rodentia | Sciuridae | Euxerus erythropus | 16.858848 | -9.408776 | L10 | Kirsch-Jung and Khtour 2007 |
| BIB_4739 | Rodentia | Sciuridae | Euxerus erythropus | 18.370251 | -12.147031 | J7 | Granjon et al 2009 |
| BIB_4738 | Rodentia | Sciuridae | Euxerus erythropus | 16.568819 | -15.596202 | M3 | Granjon et al 2009 |
| BIB_4737 | Rodentia | Sciuridae | Euxerus erythropus | 16.179554 | -13.819628 | M5 | Granjon et al 2009 |
| BIB_4736 | Rodentia | Sciuridae | Euxerus erythropus | 15.066842 | -12.492383 | N6 | Granjon et al 2009 |
| BIB_4735 | Rodentia | Sciuridae | Euxerus erythropus | 17.249855 | -10.667613 | L8 | Kirsch-Jung and Khtour 2007 |
| BIB_4734 | Rodentia | Sciuridae | Euxerus erythropus | 17.383333 | -16.050000 | L3 | Diatta et al 2015 |
| BIB_4731 | Rodentia | Sciuridae | Euxerus erythropus | 18.115564 | -16.015721 | K3 | Granjon et al 2009 |
| BIB_4730 | Rodentia | Sciuridae | Euxerus erythropus | 15.470000 | -12.950000 | N6 | Dia 2004 |
| BIB_4729 | Rodentia | Sciuridae | Euxerus erythropus | 16.780000 | -14.920000 | L4 | Dia 2004; Gueye and Dia 2004 |
| BIB_4728 | Rodentia | Sciuridae | Euxerus erythropus | 16.635783 | -16.161282 | L2 | Granjon et al 1997 |
| BIB_4727 | Rodentia | Sciuridae | Euxerus erythropus | 16.566667 | -16.283333 | M2 | Diatta et al 2015 |
| BIB_4726 | Rodentia | Sciuridae | Euxerus erythropus | 16.300000 | -10.250000 | M9 | Granjon et al 2009 |
| BIB_4725 | Rodentia | Sciuridae | Euxerus erythropus | 16.911465 | -10.140778 | L9 | Kirsch-Jung and Khtour 2007 |
| BIB_4724 | Rodentia | Sciuridae | Euxerus erythropus | 16.830000 | -15.700000 | L3 | Dia 2004; Gueye and Dia 2004 |
| BIB_4723 | Rodentia | Sciuridae | Euxerus erythropus | 16.120000 | -13.690000 | M5 | Dia 2004 |
| BIB_4722 | Rodentia | Sciuridae | Euxerus erythropus | 16.303340 | -16.401357 | M2 | National Research Council 1981; Granjon and Duplantier 2009 |
| BIB_4720 | Rodentia | Sciuridae | Euxerus erythropus | 16.540090 | -10.801490 | L8 | Nickel 2003 |
| BIB_4719 | Rodentia | Sciuridae | Euxerus erythropus | 16.510000 | -14.190000 | M5 | Dia 2004; Gueye and Dia 2004 |
| BIB_4713 | Rodentia | Sciuridae | Euxerus erythropus | 15.300000 | -12.500000 | N6 | Dia 2004 |
| 19798 | Rodentia | Sciuridae | Euxerus erythropus | 16.855487 | -16.124725 | L3 |  |
| 19797 | Rodentia | Sciuridae | Euxerus erythropus | 16.747804 | -16.131748 | L3 |  |
| 19796 | Rodentia | Sciuridae | Euxerus erythropus | 16.431100 | -16.456910 | M2 |  |
| 19775 | Rodentia | Sciuridae | Euxerus erythropus | 17.875568 | -12.091653 | K7 |  |
| 18850 | Rodentia | Sciuridae | Euxerus erythropus | 16.614303 | -11.461455 | L7 |  |
| 18848 | Rodentia | Sciuridae | Euxerus erythropus | 17.457317 | -11.340643 | K8 |  |
| 18822 | Rodentia | Sciuridae | Euxerus erythropus | 17.170590 | -10.638370 | L8 |  |
| 18777 | Rodentia | Sciuridae | Euxerus erythropus | 16.540090 | -10.801490 | L8 |  |
| 18752 | Rodentia | Sciuridae | Euxerus erythropus | 15.244620 | -10.868018 | N8 |  |
| 18733 | Rodentia | Sciuridae | Euxerus erythropus | 15.218818 | -10.956388 | N8 |  |
| 18727 | Rodentia | Sciuridae | Euxerus erythropus | 15.425605 | -11.169957 | N8 |  |
| 18726 | Rodentia | Sciuridae | Euxerus erythropus | 15.484457 | -11.204493 | N8 |  |
| 18725 | Rodentia | Sciuridae | Euxerus erythropus | 15.506115 | -11.206818 | N8 |  |
| 18660 | Rodentia | Sciuridae | Euxerus erythropus | 15.579450 | -11.964358 | N7 |  |
| 18635 | Rodentia | Sciuridae | Euxerus erythropus | 14.769627 | -12.243743 | O7 |  |
| 18620 | Rodentia | Sciuridae | Euxerus erythropus | 15.019882 | -12.455640 | N6 |  |
| 18606 | Rodentia | Sciuridae | Euxerus erythropus | 15.008882 | -12.482050 | N6 |  |
| 18605 | Rodentia | Sciuridae | Euxerus erythropus | 15.017393 | -12.490970 | N6 |  |
| 18604 | Rodentia | Sciuridae | Euxerus erythropus | 15.068010 | -12.577292 | N6 |  |
| 18602 | Rodentia | Sciuridae | Euxerus erythropus | 15.299262 | -12.824748 | N6 |  |
| 18571 | Rodentia | Sciuridae | Euxerus erythropus | 16.396427 | -16.429422 | M2 |  |
| 16177 | Rodentia | Sciuridae | Euxerus erythropus | 16.876818 | -16.100315 | L3 |  |
| 16169 | Rodentia | Sciuridae | Euxerus erythropus | 16.356807 | -16.408240 | M2 |  |
| 16151 | Rodentia | Sciuridae | Euxerus erythropus | 16.385838 | -16.481308 | M2 |  |
| 16131 | Rodentia | Sciuridae | Euxerus erythropus | 16.191065 | -13.082995 | M6 |  |
| 16128 | Rodentia | Sciuridae | Euxerus erythropus | 15.878062 | -11.468575 | M7 |  |
| 16114 | Rodentia | Sciuridae | Euxerus erythropus | 15.672178 | -11.320520 | M8 |  |
| 16068 | Rodentia | Sciuridae | Euxerus erythropus | 15.520000 | -11.296000 | N8 |  |
| 16036 | Rodentia | Sciuridae | Euxerus erythropus | 15.615627 | -11.261103 | N8 |  |
| 15998 | Rodentia | Sciuridae | Euxerus erythropus | 16.002553 | -11.871748 | M7 |  |
| 15993 | Rodentia | Sciuridae | Euxerus erythropus | 16.082252 | -11.891343 | M7 |  |
| 12910 | Rodentia | Sciuridae | Euxerus erythropus | 16.540090 | -10.801490 | L8 |  |
| 12764 | Rodentia | Sciuridae | Euxerus erythropus | 17.127682 | -10.978313 | L8 |  |
| 12740 | Rodentia | Sciuridae | Euxerus erythropus | 16.930083 | -10.634597 | L8 |  |
| 12718 | Rodentia | Sciuridae | Euxerus erythropus | 16.903207 | -10.732113 | L8 |  |
| 12574 | Rodentia | Sciuridae | Euxerus erythropus | 16.111143 | -10.836280 | M8 |  |
| 12494 | Rodentia | Sciuridae | Euxerus erythropus | 15.905693 | -10.946443 | M8 |  |
| 12474 | Rodentia | Sciuridae | Euxerus erythropus | 15.680940 | -11.070077 | M8 |  |
| 12471 | Rodentia | Sciuridae | Euxerus erythropus | 15.825777 | -11.132642 | M8 |  |
| 12457 | Rodentia | Sciuridae | Euxerus erythropus | 15.841567 | -11.160180 | M8 |  |
| 12448 | Rodentia | Sciuridae | Euxerus erythropus | 15.827323 | -11.248785 | M8 |  |
| BIB_4744 | Soricomorpha | Soricidae | Crocidura fuscomurina | 16.607000 | -16.439323 | M2 | Granjon et al 1997, 2002 |
| BIB_4751 | Soricomorpha | Soricidae | Crocidura lusitania | 18.189955 | -16.007304 | K3 | Granjon et al 1997 |
| BIB_4747 | Soricomorpha | Soricidae | Crocidura lusitania | 16.607000 | -16.439323 | M2 | Granjon et al 1997 |
| BIB_4745 | Soricomorpha | Soricidae | Crocidura lusitania | 19.740000 | -14.370000 | I4 | Poulet 1974; Le Berre 1990 |
| 4708 | Soricomorpha | Soricidae | Crocidura olivieri | 15.148390 | -12.182239 | N7 |  |
| BIB_4760 | Soricomorpha | Soricidae | Crocidura sp. | 19.751600 | -14.428100 | I4 | Jullien and Petter 1970 |
| 11868 | Soricomorpha | Soricidae | Crocidura sp. | 15.934090 | -12.046318 | M7 |  |
| BIB_4769 | Soricomorpha | Soricidae | Crocidura viaria | 18.189955 | -16.007304 | K3 | Granjon et al 1997 |
| BIB_4768 | Soricomorpha | Soricidae | Crocidura viaria | 22.650000 | -12.540000 | F6 | Heim de Balsac 1948; Le Berre 1990 |
| BIB_4761 | Soricomorpha | Soricidae | Crocidura viaria | 25.220000 | -11.577000 | C7 | Heim de Balsac 1948; Le Berre 1990 |
| BIB_4775 | Tubulidentata | Orycteropodidae | Orycteropus afer | 14.775834 | -12.213947 | O7 | Mauritanie 2000 SARL 2001 |
| BIB_4774 | Tubulidentata | Orycteropodidae | Orycteropus afer | 16.710000 | -16.120000 | L3 | Dia 2004; Gueye and Dia 2004 |
| BIB_4773 | Tubulidentata | Orycteropodidae | Orycteropus afer | 16.780000 | -14.920000 | L4 | Dia 2004; Gueye and Dia 2004 |
| BIB_4772 | Tubulidentata | Orycteropodidae | Orycteropus afer | 15.300000 | -12.500000 | N6 | Dia 2004; Gueye and Dia 2004 |
