# Supplementary material for: Diversity, distribution and conservation of land mammals in Mauritania, North-West Africa
Source: PLoS One. 2022 Aug 1;17(8):e0269870. doi: 10.1371/journal.pone.0269870 (PMC9342785; doi:10.1371/journal.pone.0269870)
Supplement: S3 Text — Dataset of observations of land mammals in Mauritania available at GBIF, including the code of the observation (Code), Order, Family and Species name (Species), the Latitude and Longitude in decimal degrees (WGS84 projection), the code of the UTM 100 km grid cell size of the observation (UTM100; codes are available in S12 Fig). (DOCX) [file pone.0269870.s021.docx]

**S16 Text. Dataset of observations.** Dataset of observations of land mammals in Mauritania available at GBIF, including the code of the observation (Code), Order, Family and Species name (Species), the Latitude and Longitude in decimal degrees (WGS84 projection), the code of the UTM 100 km grid cell size of the observation (UTM100; codes are available in S13 Figure).

| GBIF Occurrence ID | Order | Family | Species | Latitude | Longitude | UTM100 |
| --- | --- | --- | --- | --- | --- | --- |
| http://n2t.net/ark:/65665/3747c5a0a-997c-4c2f-a445-fe2c4e5ce71d | Artiodactyla | Bovidae | Gazella dorcas | 16.550000 | -15.770000 | M3 |
| b6568feb-5763-4797-beed-c3caeab3b422 | Artiodactyla | Bovidae | Gazella dorcas | 20.163000 | -16.147000 | I2 |
| cb4c9e2b-9b50-4877-9b55-788e14633f77 | Artiodactyla | Bovidae | Gazella dorcas | 19.705000 | -16.390000 | I2 |
| 40952b6a-d3c1-406e-bddf-0764df459170 | Artiodactyla | Bovidae | Gazella dorcas | 19.705000 | -16.390000 | I2 |
| 7a46afa1-e970-458e-8fae-428a76f51730 | Artiodactyla | Bovidae | Gazella dorcas | 19.705000 | -16.390000 | I2 |
| 0b777cf9-fcfb-4226-afba-9f8c5f06604e | Artiodactyla | Bovidae | Gazella dorcas | 19.705000 | -16.390000 | I2 |
| e67e9563-a62f-431c-b2fc-73c15dfa16f8 | Artiodactyla | Bovidae | Gazella dorcas | 19.705000 | -16.390000 | I2 |
| e7ba6d47-b7e7-4115-99ab-14320e5ca6f7 | Artiodactyla | Bovidae | Gazella dorcas | 19.705000 | -16.390000 | I2 |
| bc320d07-fc01-450c-8107-5c97c857726b | Artiodactyla | Bovidae | Gazella dorcas | 19.705000 | -16.390000 | I2 |
| 5c917026-e7a2-484c-ac8e-72b9b37dd3b4 | Artiodactyla | Bovidae | Gazella dorcas | 19.705000 | -16.390000 | I2 |
| 69428eff-6a8f-44b2-b3d6-46dbf228e86b | Artiodactyla | Bovidae | Gazella dorcas | 19.705000 | -16.390000 | I2 |
| 2ad92cfb-30b0-4f5b-8347-deb2f759c087 | Artiodactyla | Bovidae | Gazella dorcas | 19.806500 | -16.370000 | I2 |
| f718479c-2f96-4b1a-861d-f915da5cb8da | Artiodactyla | Bovidae | Gazella dorcas | 19.806500 | -16.370000 | I2 |
| 6da3f5ed-ffe6-4d54-9597-dc3ead5c24ad | Artiodactyla | Bovidae | Gazella dorcas | 19.806500 | -16.370000 | I2 |
| 34b3be4c-a523-4713-9c81-d81e3082b80d | Artiodactyla | Bovidae | Gazella dorcas | 19.806500 | -16.370000 | I2 |
| c6597329-d8c5-4f50-899d-799b8fbd2626 | Artiodactyla | Bovidae | Gazella dorcas | 19.806500 | -16.370000 | I2 |
| 760f6c45-3ed3-44b2-bb75-4ea070adf11b | Artiodactyla | Bovidae | Gazella dorcas | 19.806500 | -16.370000 | I2 |
| 65e792f0-5086-4791-8900-78f6c707f9cf | Artiodactyla | Bovidae | Gazella dorcas | 19.806500 | -16.370000 | I2 |
| 9265663e-b401-4de0-8946-1efe8ce14aef | Artiodactyla | Bovidae | Gazella dorcas | 19.806500 | -16.370000 | I2 |
| 4b8cb5ff-f1b2-482a-9842-5c3d20b89291 | Artiodactyla | Bovidae | Gazella dorcas | 19.806500 | -16.370000 | I2 |
| 4bfedc80-a8dd-4790-827b-0f62d4acbeef | Artiodactyla | Bovidae | Gazella dorcas | 19.806500 | -16.370000 | I2 |
| 571e0a06-0203-48a1-b7d0-58f728ddde5d | Artiodactyla | Bovidae | Gazella dorcas | 19.806500 | -16.370000 | I2 |
| fabdf102-8ff0-4980-936e-589bdb5d09ef | Artiodactyla | Bovidae | Gazella dorcas | 19.806500 | -16.370000 | I2 |
| ae8dff18-0683-4ab9-b5d2-5bc81d12f442 | Artiodactyla | Bovidae | Gazella dorcas | 19.806500 | -16.370000 | I2 |
| 6678ca7b-8f47-44a8-b975-5b4b3038ab86 | Artiodactyla | Bovidae | Gazella dorcas | 19.806500 | -16.370000 | I2 |
| 9bc5098d-23aa-443f-af75-53b9d54b7136 | Artiodactyla | Bovidae | Gazella dorcas | 19.806500 | -16.370000 | I2 |
| aae6ae6f-0a14-4c96-ac4a-a81a00d84b96 | Artiodactyla | Bovidae | Gazella dorcas | 19.806500 | -16.370000 | I2 |
| c1bdfd89-c3e3-437a-8915-cd977a2f78bd | Artiodactyla | Bovidae | Gazella dorcas | 19.806500 | -16.370000 | I2 |
| 2586978c-bb93-48c9-a991-a9ae25b62dc9 | Artiodactyla | Bovidae | Gazella dorcas | 19.806500 | -16.370000 | I2 |
| 1ee8ce37-f6ca-4bd1-bd36-6f8c6f0cd1fd | Artiodactyla | Bovidae | Gazella dorcas | 19.806500 | -16.370000 | I2 |
| b82ae4a6-44f3-4f89-9b5b-0d6acbac94d2 | Artiodactyla | Bovidae | Gazella dorcas | 19.806500 | -16.370000 | I2 |
| 54b6a12b-aeab-4d7e-b4da-2c9f15161086 | Artiodactyla | Bovidae | Gazella dorcas | 19.806500 | -16.370000 | I2 |
| 242c8059-b9e4-451c-954f-f9761d5f07fd | Artiodactyla | Bovidae | Gazella dorcas | 19.806500 | -16.370000 | I2 |
| d405994a-1fb9-4f08-9107-42478dad8dfa | Artiodactyla | Bovidae | Gazella dorcas | 19.806500 | -16.370000 | I2 |
| 2adf9630-1ed2-4743-ab1d-9220677b8485 | Artiodactyla | Bovidae | Gazella dorcas | 19.806500 | -16.370000 | I2 |
| 89e63c5d-bb0c-45d3-a010-475b09ddd66e | Artiodactyla | Bovidae | Gazella dorcas | 19.806500 | -16.370000 | I2 |
| 963686bb-9ca7-4b49-a0e8-421bc9f90c5d | Artiodactyla | Bovidae | Gazella dorcas | 19.806500 | -16.370000 | I2 |
| baab2e13-98f5-4752-93d2-785f2858c223 | Artiodactyla | Bovidae | Gazella dorcas | 19.806500 | -16.370000 | I2 |
| 2d940853-25e9-4eb8-9b9b-98398aaec3ee | Artiodactyla | Bovidae | Gazella dorcas | 19.806500 | -16.370000 | I2 |
| 5ae24852-8c54-457b-823d-a171acc91f1c | Artiodactyla | Bovidae | Gazella dorcas | 19.806500 | -16.370000 | I2 |
| acceec9a-c8e5-418e-b13b-02129e8c90a4 | Artiodactyla | Bovidae | Gazella dorcas | 19.806500 | -16.370000 | I2 |
| fc140ae2-a90b-40f4-bbc0-a6569eebde3c | Artiodactyla | Bovidae | Gazella dorcas | 19.806500 | -16.370000 | I2 |
| dada9d7e-b8d2-46d3-bf22-a76cc69a7728 | Artiodactyla | Bovidae | Gazella dorcas | 19.806500 | -16.370000 | I2 |
| 1ce902f2-1959-460c-938c-2f4353bca5a8 | Artiodactyla | Bovidae | Gazella dorcas | 19.806500 | -16.370000 | I2 |
| 39618fe6-9f97-4a49-9100-f085a38617ee | Artiodactyla | Bovidae | Gazella dorcas | 21.500223 | -11.619505 | G7 |
| e20e3f18-fc37-4509-9298-b12d4c0f435c | Artiodactyla | Bovidae | Gazella dorcas | 20.700000 | -13.100000 | H6 |
| 0ad0be99-d2cb-46e0-b798-548497e7f023 | Artiodactyla | Bovidae | Gazella dorcas | 21.262928 | -14.549194 | G4 |
| 72320a3d-3f74-45ab-961e-02b1a338757b | Artiodactyla | Bovidae | Gazella dorcas | 21.663533 | -11.683610 | G7 |
| 7ddba889-1a2a-4b53-9ed2-c934f8f35628 | Artiodactyla | Bovidae | Gazella dorcas | 21.760138 | -11.327094 | G7 |
| 59015f19-8042-4863-b1f4-2f2e6f6701e0 | Artiodactyla | Bovidae | Gazella dorcas | 21.760138 | -11.327094 | G7 |
| 0d839582-d461-4f01-9e6f-955c6522db02 | Artiodactyla | Bovidae | Gazella dorcas | 19.068717 | -11.932567 | J7 |
| https://observation.org/observation/98178121 | Artiodactyla | Suidae | Phacochoerus africanus | 16.507709 | -16.026590 | M3 |
| http://n2t.net/ark:/65665/38fc3a8e4-419e-4ea1-a9ca-349aad3e33d7 | Artiodactyla | Suidae | Phacochoerus africanus | 15.933333 | -12.016667 | M7 |
| https://observation.org/observation/133730954 | Artiodactyla | Suidae | Phacochoerus africanus | 16.570936 | -16.318386 | M2 |
| http://n2t.net/ark:/65665/37ab78ac5-25ac-43d0-8a4d-a93d66d4f174 | Artiodactyla | Suidae | Phacochoerus africanus | 16.550000 | -15.766667 | M3 |
| http://n2t.net/ark:/65665/3fd297b9c-181b-43ca-8541-7f611a0cef44 | Artiodactyla | Suidae | Phacochoerus africanus | 16.550000 | -15.766667 | M3 |
| http://www.inaturalist.org/observations/4757018 | Artiodactyla | Suidae | Phacochoerus africanus | 16.310333 | -16.394000 | M2 |
| https://www.inaturalist.org/observations/28074273 | Artiodactyla | Suidae | Phacochoerus africanus | 16.285079 | -16.411679 | M2 |
| https://observation.org/observation/52977781 | Artiodactyla | Suidae | Phacochoerus africanus | 16.303357 | -16.401211 | M2 |
| https://observation.org/observation/185326682 | Artiodactyla | Suidae | Phacochoerus africanus | 16.416761 | -16.330278 | M2 |
| https://observation.org/observation/83101372 | Artiodactyla | Suidae | Phacochoerus africanus | 16.409654 | -16.402263 | M2 |
| https://observation.org/observation/81293104 | Artiodactyla | Suidae | Phacochoerus africanus | 16.408707 | -16.400099 | M2 |
| https://observation.org/observation/97340766 | Artiodactyla | Suidae | Phacochoerus africanus | 16.407963 | -16.334926 | M2 |
| https://observation.org/observation/81358279 | Artiodactyla | Suidae | Phacochoerus africanus | 16.407794 | -16.399315 | M2 |
| https://observation.org/observation/185071831 | Artiodactyla | Suidae | Phacochoerus africanus | 16.362961 | -16.341778 | M2 |
| https://observation.org/observation/185326671 | Artiodactyla | Suidae | Phacochoerus africanus | 16.362120 | -16.341837 | M2 |
| https://observation.org/observation/165553520 | Artiodactyla | Suidae | Phacochoerus africanus | 16.359270 | -16.342962 | M2 |
| https://www.inaturalist.org/observations/19658687 | Artiodactyla | Suidae | Phacochoerus africanus | 16.359240 | -16.344029 | M2 |
| https://observation.org/observation/97602962 | Artiodactyla | Suidae | Phacochoerus africanus | 16.349927 | -16.342451 | M2 |
| https://observation.org/observation/185071817 | Artiodactyla | Suidae | Phacochoerus africanus | 16.344162 | -16.347746 | M2 |
| https://observation.org/observation/147749338 | Artiodactyla | Suidae | Phacochoerus africanus | 16.339700 | -16.354300 | M2 |
| https://observation.org/observation/149684647 | Artiodactyla | Suidae | Phacochoerus africanus | 16.315856 | -16.375637 | M2 |
| https://observation.org/observation/149684369 | Artiodactyla | Suidae | Phacochoerus africanus | 16.312226 | -16.387138 | M2 |
| https://observation.org/observation/97340729 | Artiodactyla | Suidae | Phacochoerus africanus | 16.506655 | -16.292496 | M2 |
| 6934e81c-7866-4d4c-adc6-62277fadb00b | Artiodactyla | Suidae | Phacochoerus africanus | 16.409377 | -16.432418 | M2 |
| 779a24de-c23a-4f7a-a155-ec3d44858469 | Artiodactyla | Suidae | Phacochoerus africanus | 16.713367 | -16.381220 | L2 |
| ae319846-202a-4f5a-8dd2-207b3c6902b8 | Artiodactyla | Suidae | Phacochoerus africanus | 16.756568 | -16.374088 | L2 |
| 5531ebc1-448b-437d-89fa-bb1ae948f870 | Artiodactyla | Suidae | Phacochoerus africanus | 16.369898 | -16.341423 | M2 |
| 6ebcd2be-fa10-4da4-802b-e6f22dd06a97 | Artiodactyla | Suidae | Phacochoerus africanus | 16.848708 | -15.296972 | L3 |
| 36c54d34-5d21-421a-a98f-78444da85eab | Artiodactyla | Suidae | Phacochoerus africanus | 16.579845 | -16.257075 | M2 |
| 8352a10c-06d0-4847-875a-c0e03f5255d7 | Artiodactyla | Suidae | Phacochoerus africanus | 16.499093 | -16.056827 | M3 |
| 5a18432a-424e-406c-802b-f0fb333d8a8d | Artiodactyla | Suidae | Phacochoerus africanus | 16.378000 | -16.386700 | M2 |
| c96dd7a8-7687-4989-8010-c761f2d3948e | Artiodactyla | Suidae | Phacochoerus africanus | 16.376340 | -16.421818 | M2 |
| dccd111b-7693-4a9b-adfc-4ab95017f95a | Artiodactyla | Suidae | Phacochoerus africanus | 16.406883 | -16.452698 | M2 |
| 5982355f-2492-474b-989e-7d38a7bfb5e3 | Artiodactyla | Suidae | Phacochoerus africanus | 16.491833 | -16.031667 | M3 |
| 21ba85a9-f0fc-41c2-bff5-029af8cc6b8a | Artiodactyla | Suidae | Phacochoerus africanus | 16.523933 | -16.205583 | M2 |
| 35f1d034-bc31-4b79-9d52-e01b623cd210 | Artiodactyla | Suidae | Phacochoerus africanus | 16.523933 | -16.205583 | M2 |
| a121c111-62e4-4f63-bd69-d2edfe9bf559 | Artiodactyla | Suidae | Phacochoerus africanus | 16.340750 | -16.352900 | M2 |
| d352146f-2840-4989-a4ce-65c7d1ec9a38 | Artiodactyla | Suidae | Phacochoerus africanus | 17.451667 | -12.394850 | K6 |
| https://www.inaturalist.org/observations/37493293 | Carnivora | Canidae | Canis lupaster | 19.521072 | -15.923861 | I3 |
| http://n2t.net/ark:/65665/3795a40b6-7671-43d2-a310-8bb344c552d9 | Carnivora | Canidae | Canis lupaster | 17.266783 | -16.028417 | L3 |
| http://n2t.net/ark:/65665/3540db170-58d7-4273-a55e-c9631a232cf3 | Carnivora | Canidae | Canis lupaster | 15.933333 | -12.016667 | M7 |
| http://n2t.net/ark:/65665/3c098d4cf-0a76-4f2f-9a74-93de851b8c54 | Carnivora | Canidae | Canis lupaster | 16.620000 | -11.400000 | L7 |
| http://n2t.net/ark:/65665/388b85d2a-0c1d-4950-969d-a8ca6aa4103b | Carnivora | Canidae | Canis lupaster | 16.150000 | -13.500000 | M5 |
| https://observation.org/observation/98324164 | Carnivora | Canidae | Canis lupaster | 19.894434 | -16.295613 | I2 |
| https://observation.org/observation/165129510 | Carnivora | Canidae | Canis lupaster | 19.891781 | -16.288897 | I2 |
| https://observation.org/observation/97981535 | Carnivora | Canidae | Canis lupaster | 19.889815 | -16.296236 | I2 |
| https://observation.org/observation/185326833 | Carnivora | Canidae | Canis lupaster | 19.887411 | -16.299377 | I2 |
| https://observation.org/observation/114007605 | Carnivora | Canidae | Canis lupaster | 19.887187 | -16.299352 | I2 |
| https://observation.org/observation/97071911 | Carnivora | Canidae | Canis lupaster | 19.887166 | -16.298984 | I2 |
| https://observation.org/observation/149376932 | Carnivora | Canidae | Canis lupaster | 19.887124 | -16.298910 | I2 |
| https://observation.org/observation/147672356 | Carnivora | Canidae | Canis lupaster | 19.887100 | -16.299300 | I2 |
| https://observation.org/observation/133617544 | Carnivora | Canidae | Canis lupaster | 19.887000 | -16.299000 | I2 |
| https://observation.org/observation/134274455 | Carnivora | Canidae | Canis lupaster | 19.886763 | -16.298991 | I2 |
| https://observation.org/observation/147672326 | Carnivora | Canidae | Canis lupaster | 19.886700 | -16.299300 | I2 |
| https://observation.org/observation/185013889 | Carnivora | Canidae | Canis lupaster | 19.886562 | -16.299219 | I2 |
| https://observation.org/observation/185326910 | Carnivora | Canidae | Canis lupaster | 19.886501 | -16.299164 | I2 |
| https://observation.org/observation/145844209 | Carnivora | Canidae | Canis lupaster | 19.885201 | -16.295196 | I2 |
| https://observation.org/observation/134162066 | Carnivora | Canidae | Canis lupaster | 19.885201 | -16.295196 | I2 |
| https://observation.org/observation/165129539 | Carnivora | Canidae | Canis lupaster | 19.884201 | -16.295240 | I2 |
| https://observation.org/observation/164965036 | Carnivora | Canidae | Canis lupaster | 19.883429 | -16.293491 | I2 |
| https://observation.org/observation/98178109 | Carnivora | Canidae | Canis lupaster | 16.506509 | -16.292395 | M2 |
| http://n2t.net/ark:/65665/340fe0638-b478-4375-aa0f-c570062c0750 | Carnivora | Canidae | Canis lupaster | 16.650000 | -14.283333 | L4 |
| http://n2t.net/ark:/65665/381c70871-ee5d-4879-b8b5-dd97dc3e7bcc | Carnivora | Canidae | Canis lupaster | 16.650000 | -14.283333 | L4 |
| 9ae9c35f-eda1-4013-b970-59d18a522a39 | Carnivora | Canidae | Canis lupaster | 15.480707 | -12.305692 | N7 |
| 9e968bf2-3bf9-4c20-919b-af46cff1c0e8 | Carnivora | Canidae | Canis lupaster | 15.480707 | -12.305692 | N7 |
| a4e6a755-10f1-410d-9f6f-a114d141bf68 | Carnivora | Canidae | Canis lupaster | 17.067572 | -12.260290 | L7 |
| 078d78ef-b364-4184-a7b3-360f0782a108 | Carnivora | Canidae | Canis lupaster | 15.903972 | -11.936185 | M7 |
| a65a510c-36b4-4587-9451-074e1e36d2c5 | Carnivora | Canidae | Canis lupaster | 17.230262 | -13.828335 | L5 |
| 0ab46140-80ad-4d13-b5e4-be0717a2e8b5 | Carnivora | Canidae | Canis lupaster | 16.543543 | -9.956927 | L9 |
| 8cbec83a-2327-4974-8653-10154bcd4f25 | Carnivora | Canidae | Canis lupaster | 15.798355 | -10.805377 | M8 |
| eb19c556-d2a0-4960-b9dd-5cc69c7690ee | Carnivora | Canidae | Canis lupaster | 15.694747 | -11.237802 | M8 |
| 61a49ddc-35d6-4821-b9cb-6ba0cefab650 | Carnivora | Canidae | Canis lupaster | 15.013457 | -12.476630 | N6 |
| 3eee12e2-5f18-4cd9-bbb4-ef53d96768ec | Carnivora | Canidae | Canis lupaster | 15.946357 | -13.293712 | M6 |
| fbf31eed-004f-4c08-ac73-8aca2156f86f | Carnivora | Canidae | Canis lupaster | 18.206040 | -11.730977 | K7 |
| 8623759b-6210-42fd-826a-f9fdd4f38372 | Carnivora | Canidae | Canis lupaster | 16.489543 | -11.057988 | M8 |
| be20d917-b00b-4273-a527-2cc9164bac39 | Carnivora | Canidae | Canis lupaster | 18.400353 | -11.814568 | J7 |
| b27c1648-b856-43a8-b65d-d9fb2a28c69e | Carnivora | Canidae | Canis lupaster | 18.400353 | -11.814568 | J7 |
| dd7681d2-dab1-44dc-a40a-234d828239ea | Carnivora | Canidae | Canis lupaster | 16.186915 | -13.638600 | M5 |
| 55de2633-db41-4759-9e70-761a19a672b0 | Carnivora | Canidae | Canis lupaster | 16.275638 | -13.703690 | M5 |
| 671bafe7-4057-44c3-bf38-fd3d1af4dd30 | Carnivora | Canidae | Canis lupaster | 20.131033 | -15.953302 | I3 |
| 49618aec-0ed2-495d-a49c-452333a8ae61 | Carnivora | Canidae | Canis lupaster | 17.124468 | -12.076173 | L7 |
| 2b152a74-34af-4626-bd23-db46bf578368 | Carnivora | Canidae | Canis lupaster | 16.444350 | -11.778073 | M7 |
| 63cb6dd1-f900-4a61-8a55-46a87310298d | Carnivora | Canidae | Canis lupaster | 16.053245 | -11.669883 | M7 |
| 11fced33-3abd-47fe-8e5c-4857a29dbbb5 | Carnivora | Canidae | Canis lupaster | 16.338801 | -11.978097 | M7 |
| a19a7e5f-4dda-445e-a921-7c72b39bfee4 | Carnivora | Canidae | Canis lupaster | 15.957078 | -12.009859 | M7 |
| 10ca1d82-d4ad-4b05-906d-ccc943becf10 | Carnivora | Canidae | Canis lupaster | 15.591013 | -12.242632 | N7 |
| 4b2a5ed4-8f72-4bcc-86f7-b2163e616642 | Carnivora | Canidae | Canis lupaster | 15.591013 | -12.242632 | N7 |
| dbdef364-16d8-417a-89e1-32357d2147fb | Carnivora | Canidae | Canis lupaster | 15.591013 | -12.242632 | N7 |
| 27127190-0137-46b7-b86f-100efb977fbc | Carnivora | Canidae | Canis lupaster | 15.058525 | -12.406165 | N6 |
| 2ae45294-7f7c-4e21-99b3-8a2590a3dd11 | Carnivora | Canidae | Canis lupaster | 16.442508 | -16.316668 | M2 |
| df8bcccb-f3d5-4f0a-9a0f-1cd864321848 | Carnivora | Canidae | Canis lupaster | 18.258250 | -11.513437 | K7 |
| 0fb61770-4c11-4686-9eba-c30d3eef58e7 | Carnivora | Canidae | Canis lupaster | 18.053485 | -11.942891 | K7 |
| 7c54a26e-e5be-403d-b665-b9992c427928 | Carnivora | Canidae | Canis lupaster | 18.258250 | -11.513437 | K7 |
| da7cba34-b407-45f7-82e5-2b0481e7c360 | Carnivora | Canidae | Canis lupaster | 20.739577 | -16.415012 | H2 |
| 6846a6b1-f983-42f0-92d1-c20932a2ab44 | Carnivora | Canidae | Canis lupaster | 20.420480 | -16.276895 | H2 |
| 23292c00-8d66-4ce5-87d6-40d89f29d751 | Carnivora | Canidae | Canis lupaster | 20.420480 | -16.276895 | H2 |
| b3b54076-af68-456a-960f-3030d0b9da4c | Carnivora | Canidae | Canis lupaster | 20.233015 | -16.124765 | H2 |
| d60875e2-134b-489b-b13b-3e22416eb1dd | Carnivora | Canidae | Canis lupaster | 20.123383 | -16.126637 | I2 |
| 41d6560c-e718-4f5c-845f-42fed7bcbd92 | Carnivora | Canidae | Canis lupaster | 19.596660 | -16.378452 | I2 |
| 6fe95632-628e-438f-803f-f4c69542e755 | Carnivora | Canidae | Canis lupaster | 19.563600 | -16.396827 | I2 |
| bd8f4d49-5c99-4b3c-8d8d-3633ca3d39c8 | Carnivora | Canidae | Canis lupaster | 19.422068 | -16.297312 | I2 |
| a46a8b95-7173-47b4-ae6e-c79690af14e9 | Carnivora | Canidae | Canis lupaster | 18.258250 | -11.513437 | K7 |
| cdc1e2a9-b4f0-4ce5-96d8-173f59b7fc9b | Carnivora | Canidae | Canis lupaster | 18.292045 | -11.728962 | K7 |
| e35a39a7-ddfb-403c-9ec3-aac494efe353 | Carnivora | Canidae | Canis lupaster | 18.053485 | -11.942891 | K7 |
| a5b399ca-a36b-4330-baa6-0ecb04f802d1 | Carnivora | Canidae | Canis lupaster | 17.846344 | -12.078243 | K7 |
| 8933cdd0-441f-4fe8-b8cc-3ea9349a904e | Carnivora | Canidae | Canis lupaster | 17.424440 | -13.357260 | L5 |
| 00573130-7cd0-430e-a84a-2d0bf8ece2bc | Carnivora | Canidae | Canis lupaster | 18.259342 | -16.017840 | K3 |
| 885471cb-4a1c-408c-acfe-6f8e201151bb | Carnivora | Canidae | Canis lupaster | 18.795397 | -16.139140 | J2 |
| a37fe6a8-eae3-4fc1-af0f-bd02042ba0b1 | Carnivora | Canidae | Canis lupaster | 19.835467 | -16.353253 | I2 |
| fecf7b3e-e675-4e27-b2f0-2e71983ddcf6 | Carnivora | Canidae | Canis lupaster | 19.818659 | -16.360463 | I2 |
| f9c7e1a4-ccbd-42d0-befe-9121020be433 | Carnivora | Canidae | Canis lupaster | 19.794865 | -16.366492 | I2 |
| 8ceca921-09a9-4a92-8af6-399c09c8205a | Carnivora | Canidae | Canis lupaster | 22.580110 | -12.715732 | F6 |
| 6cecc63b-ca10-487c-b064-bbcfcd355d08 | Carnivora | Canidae | Canis lupaster | 20.700182 | -14.531232 | H4 |
| 19fadbba-2237-4707-802c-e627578d4a26 | Carnivora | Canidae | Canis lupaster | 20.149803 | -16.171913 | I2 |
| 935be208-02dc-4b0f-9d75-c8ca6c462d32 | Carnivora | Canidae | Canis lupaster | 19.972027 | -16.229890 | I2 |
| f852286f-f3e2-4578-a029-582581bce7b6 | Carnivora | Canidae | Canis lupaster | 18.444300 | -15.710500 | J3 |
| d4a69a87-b43c-42e2-9caa-cf7441d6d2d9 | Carnivora | Canidae | Canis lupaster | 18.374573 | -15.737555 | K3 |
| ec634497-f34b-4978-8a3b-95bfe32a15bb | Carnivora | Canidae | Canis lupaster | 17.188300 | -13.854100 | L5 |
| e0074e4c-88cb-402a-92e2-287ae4844da5 | Carnivora | Canidae | Canis lupaster | 17.422828 | -13.435163 | L5 |
| a7c49658-a75b-415b-9ea7-6052272f03fa | Carnivora | Canidae | Canis lupaster | 15.908323 | -11.921210 | M7 |
| 57f72c42-f083-4cfe-a1c7-6eac0c490c89 | Carnivora | Canidae | Canis lupaster | 15.957078 | -12.009859 | M7 |
| 8d1ce09b-fd7a-4e27-bb90-a969fc09cf08 | Carnivora | Canidae | Canis lupaster | 15.641847 | -12.109977 | N7 |
| 5dc4db2f-5273-4efb-ba6b-cd71eddc36cf | Carnivora | Canidae | Canis lupaster | 15.506540 | -11.762700 | N7 |
| 5931aa52-00b9-491a-9eca-82cc3ddaecae | Carnivora | Canidae | Canis lupaster | 15.335200 | -12.657417 | N6 |
| 5a6307b9-5349-464a-8449-5575e89937bb | Carnivora | Canidae | Canis lupaster | 16.257035 | -13.658015 | M5 |
| 5966174c-8e3d-448b-9b75-de8e30e07541 | Carnivora | Canidae | Canis lupaster | 16.499093 | -16.056827 | M3 |
| 8efba09c-7b49-43bf-a73b-d912c4f3e745 | Carnivora | Canidae | Canis lupaster | 18.350840 | -16.008290 | K3 |
| 7141f996-340d-4112-8509-95a7f0d2dff9 | Carnivora | Canidae | Canis lupaster | 19.558323 | -16.401927 | I2 |
| 7b548c29-04c6-4289-8dcb-e06d71ae7fe5 | Carnivora | Canidae | Canis lupaster | 19.467583 | -16.433225 | I2 |
| 950e7b83-28f9-420a-8afe-4f6250f4d2a9 | Carnivora | Canidae | Canis lupaster | 19.600348 | -16.431332 | I2 |
| 5b4fe38c-78d9-4e67-9bba-1a5cef406538 | Carnivora | Canidae | Canis lupaster | 19.427665 | -16.468322 | I2 |
| d92adf6c-d827-41d9-a973-b64122ae367c | Carnivora | Canidae | Canis lupaster | 19.400027 | -16.397685 | I2 |
| a33e1203-7c8c-450f-8ef6-faf49a8232cb | Carnivora | Canidae | Canis lupaster | 16.756482 | -11.997233 | L7 |
| 3236b2ac-d95b-419e-927a-5fea30034ec5 | Carnivora | Canidae | Canis lupaster | 17.240833 | -12.101667 | L7 |
| d13fd190-a52f-41dc-b3e1-c4c9ad24e313 | Carnivora | Canidae | Canis lupaster | 17.478648 | -12.161713 | K7 |
| 2059e0f3-522e-4a08-95df-254899fccead | Carnivora | Canidae | Canis lupaster | 17.811980 | -11.943953 | K7 |
| 98311fe4-7ce8-4140-b973-c9df1ba3d306 | Carnivora | Canidae | Canis lupaster | 17.762083 | -11.882833 | K7 |
| 4eb5f5ab-b13d-43bf-9e89-3152ee7cb03b | Carnivora | Canidae | Canis lupaster | 17.706072 | -11.826152 | K7 |
| 7d2492ad-4012-4a8e-a2fe-4aed217bff84 | Carnivora | Canidae | Canis lupaster | 17.929848 | -11.745102 | K7 |
| d3a0eb61-9071-447a-b477-ea4bab88913c | Carnivora | Canidae | Canis lupaster | 17.846344 | -12.078243 | K7 |
| 2502cade-09a6-421e-8605-639fe64b52a3 | Carnivora | Canidae | Canis lupaster | 17.887298 | -12.110844 | K7 |
| 22361f8c-f322-4f9d-a5c7-255b2b3f3af5 | Carnivora | Canidae | Canis lupaster | 17.415093 | -12.528163 | L6 |
| 37e923cd-ef91-49f6-b034-c25493dd42a0 | Carnivora | Canidae | Canis lupaster | 16.888725 | -12.184868 | L7 |
| 42d43ef7-5d6a-4463-b087-bdd97b734e73 | Carnivora | Canidae | Canis lupaster | 15.957078 | -12.009859 | M7 |
| 61bc3221-0622-4556-b6fc-6563b7c1b987 | Carnivora | Canidae | Canis lupaster | 15.932785 | -12.010887 | M7 |
| b111cb4b-11b7-4f83-955f-bb2de305153b | Carnivora | Canidae | Canis lupaster | 16.538033 | -10.741550 | L8 |
| a1216b6b-4746-4ca6-85cc-3cd9c8408640 | Carnivora | Canidae | Canis lupaster | 16.687579 | -10.191363 | L9 |
| 78062682-1ea6-4d94-afe5-46f8d35b7c69 | Carnivora | Canidae | Canis lupaster | 16.515562 | -10.452908 | M8 |
| a35e2962-54ee-49c5-b46e-584a1d78a974 | Carnivora | Canidae | Canis lupaster | 16.425255 | -9.562955 | M9 |
| ad3e93a5-a980-43c2-adc5-4ab31735db46 | Carnivora | Canidae | Canis lupaster | 16.428120 | -9.561713 | M9 |
| 116438a8-3303-4b4c-849c-905bed003d85 | Carnivora | Canidae | Canis lupaster | 17.551855 | -11.284110 | K8 |
| 4e006a00-a735-4c23-92c2-90e2667522b4 | Carnivora | Canidae | Canis lupaster | 20.580946 | -13.136361 | H6 |
| 29dfb113-7087-41a0-a17c-d1bae75701af | Carnivora | Canidae | Canis lupaster | 20.519268 | -13.132583 | H6 |
| a6588736-73b9-467f-8456-c51af8e65ebe | Carnivora | Canidae | Canis lupaster | 21.243300 | -13.282010 | G5 |
| f334b419-4abc-442e-8541-1dfc4ff6a452 | Carnivora | Canidae | Canis lupaster | 17.378717 | -12.342117 | L6 |
| 87a815c7-0ed2-4b42-8816-06c7c636dc00 | Carnivora | Canidae | Canis lupaster | 16.225517 | -13.384467 | M5 |
| 41c894d5-364f-4549-aa45-bf1be96a6409 | Carnivora | Canidae | Canis lupaster | 19.123567 | -16.294817 | J2 |
| 5e829c79-ad17-4ac0-bac4-9a9a4ab69c85 | Carnivora | Canidae | Canis lupaster | 18.860750 | -16.174450 | J2 |
| 60d8bebf-75ea-446e-a148-c1b493d75edc | Carnivora | Canidae | Canis lupaster | 18.559750 | -16.088333 | J3 |
| 0bc63ab6-ea14-4fde-a07e-259432a33300 | Carnivora | Canidae | Canis lupaster | 21.154933 | -16.983000 | G2 |
| 77e12aa7-e058-4474-b066-de097e43a0c8 | Carnivora | Canidae | Canis lupaster | 19.844800 | -14.246483 | I4 |
| 36d6d50f-7155-41d7-a8b8-bd16864b53e3 | Carnivora | Canidae | Canis lupaster | 17.785250 | -16.041700 | K3 |
| 5243f29c-b9e5-481d-ade9-483ee30466d0 | Carnivora | Canidae | Canis lupaster | 17.551683 | -16.028833 | K3 |
| b254dd79-9428-47e8-9ba8-59acced21666 | Carnivora | Canidae | Canis lupaster | 19.242583 | -16.452500 | J2 |
| 68249893-7651-453b-911e-bac805b5887b | Carnivora | Canidae | Canis lupaster | 19.206383 | -16.382717 | J2 |
| d270eeee-0c0b-4422-bbcc-d06f872b0d6c | Carnivora | Canidae | Canis lupaster | 18.979700 | -16.210317 | J2 |
| 8a3cf18f-29c7-42d9-99f8-a835ddf17330 | Carnivora | Canidae | Canis lupaster | 17.392517 | -13.452850 | L5 |
| 721a8009-49e9-4d97-a733-aa4df99a0ddb | Carnivora | Canidae | Canis lupaster | 17.451667 | -12.394850 | K6 |
| 752c11ae-8c3b-46b9-9ba4-e5f5fbe75af6 | Carnivora | Canidae | Canis lupaster | 16.489217 | -10.643133 | M8 |
| e5651fea-6479-4c23-8e27-c5072b2574af | Carnivora | Canidae | Canis lupaster | 17.434417 | -7.407617 | K12 |
| http://n2t.net/ark:/65665/39731e7b0-4334-46e0-b0f5-a4f67a4632ea | Carnivora | Canidae | Vulpes pallida | 16.150000 | -13.500000 | M5 |
| http://n2t.net/ark:/65665/3945075fc-50a6-4519-8c05-fe3f0227fe8c | Carnivora | Canidae | Vulpes pallida | 16.150000 | -13.500000 | M5 |
| http://n2t.net/ark:/65665/32554cccf-3ef9-4191-9402-8f6dd7f3e0aa | Carnivora | Canidae | Vulpes pallida | 16.650000 | -14.283333 | L4 |
| dae45502-dd3d-4199-910d-4c1fad10c105 | Carnivora | Canidae | Vulpes pallida | 15.013457 | -12.476630 | N6 |
| d0f7581a-e76a-4213-b6a4-6d9a28f8376e | Carnivora | Canidae | Vulpes pallida | 17.230262 | -13.828335 | L5 |
| d864cfaa-dc32-47bc-a646-63e70d057c2c | Carnivora | Canidae | Vulpes pallida | 16.624512 | -9.446593 | L10 |
| f79ac57f-61d7-41ad-a4c6-a6d324e15061 | Carnivora | Canidae | Vulpes pallida | 16.605465 | -15.291103 | L3 |
| 041d8611-0f30-44de-bb19-48d83dd98439 | Carnivora | Canidae | Vulpes pallida | 16.484132 | -9.289523 | M10 |
| d8263e1d-3df2-40df-94c3-3269bcd905fd | Carnivora | Canidae | Vulpes pallida | 18.447222 | -10.683298 | J8 |
| ed30fe79-34bb-4a30-88bf-33017a7decbc | Carnivora | Canidae | Vulpes pallida | 16.484132 | -9.289523 | M10 |
| b04b094f-94ee-4989-8c3f-c08d205ff41b | Carnivora | Canidae | Vulpes pallida | 16.275638 | -13.703690 | M5 |
| fbb975f6-8657-4028-8f31-f292189e5eaa | Carnivora | Canidae | Vulpes pallida | 18.356815 | -11.816080 | J7 |
| 111d3908-7189-4c09-83a6-2a4365156b6e | Carnivora | Canidae | Vulpes pallida | 18.447222 | -10.683298 | J8 |
| 9264e215-26e0-43c0-aef7-c58bdc7bdc77 | Carnivora | Canidae | Vulpes pallida | 18.447222 | -10.683298 | J8 |
| a0e025c4-1339-4dc0-9286-81d58f086858 | Carnivora | Canidae | Vulpes pallida | 16.479953 | -7.589865 | M11 |
| b78b4db2-94db-4f87-a994-a265d393d46c | Carnivora | Canidae | Vulpes pallida | 16.484132 | -9.289523 | M10 |
| 9e76a798-9cf3-466e-ae76-e81056343512 | Carnivora | Canidae | Vulpes pallida | 16.574737 | -9.593983 | L9 |
| 2f32cb97-d6f5-4adf-9e6f-af0b63f2dd70 | Carnivora | Canidae | Vulpes pallida | 15.969122 | -9.476628 | M9 |
| a6e56b5f-1288-4f6a-b1b7-b78d072c9556 | Carnivora | Canidae | Vulpes pallida | 16.581247 | -11.413648 | L7 |
| e133c4a4-146f-4dd8-a075-e1b1f331c43e | Carnivora | Canidae | Vulpes pallida | 16.197008 | -13.092545 | M6 |
| 8474a3bc-0f6f-4b93-b0ad-6a3e5252f7b7 | Carnivora | Canidae | Vulpes pallida | 16.223883 | -13.173627 | M6 |
| ce444057-2ee2-482a-be6b-e85d3221f134 | Carnivora | Canidae | Vulpes pallida | 16.181137 | -13.633772 | M5 |
| 1b141f93-105f-449f-95a8-6a4af564f547 | Carnivora | Canidae | Vulpes pallida | 16.583550 | -14.223820 | L5 |
| 0ce96afb-4336-4c88-827d-7124bf1e7d73 | Carnivora | Canidae | Vulpes pallida | 17.165265 | -14.125562 | L5 |
| c82b11c8-b2a7-4e46-bb3d-2b916bf09f3c | Carnivora | Canidae | Vulpes pallida | 16.303340 | -16.401357 | M2 |
| a122dbd4-95ce-497a-a559-78848c024991 | Carnivora | Canidae | Vulpes pallida | 17.029738 | -13.955475 | L5 |
| dddcd03e-a7cb-43b0-a2c0-831ae331a160 | Carnivora | Canidae | Vulpes pallida | 16.693473 | -11.270058 | L8 |
| 0432eb6c-a96c-4918-8908-f2667e40e3a4 | Carnivora | Canidae | Vulpes pallida | 16.155158 | -13.544458 | M5 |
| 06e59169-aa86-4af1-b62d-3941d4abdb07 | Carnivora | Canidae | Vulpes pallida | 16.685707 | -15.019587 | L4 |
| 92ebd2b2-e703-4013-a3f6-0ab1bbd34039 | Carnivora | Canidae | Vulpes pallida | 16.339388 | -12.505163 | M6 |
| 30a6ad8a-02ac-4339-b57d-d28b9594f4c3 | Carnivora | Canidae | Vulpes pallida | 15.742177 | -13.240178 | M6 |
| ac0f61f5-ceb6-45d1-952f-e5b85c2a11f6 | Carnivora | Canidae | Vulpes pallida | 15.494547 | -12.952418 | N6 |
| b943ba00-a300-4016-9450-0a3ff9b1bcaf | Carnivora | Canidae | Vulpes pallida | 15.490148 | -12.940880 | N6 |
| d8ee1d23-4ed0-4467-b0d0-2113c4a5503e | Carnivora | Canidae | Vulpes pallida | 15.055633 | -12.554327 | N6 |
| e95c9f57-ff49-4063-b569-da6569d492d4 | Carnivora | Canidae | Vulpes pallida | 17.177600 | -14.146600 | L5 |
| 830f369d-d902-411d-8827-e7c208539e9f | Carnivora | Canidae | Vulpes pallida | 17.177600 | -14.146600 | L5 |
| f3dfad33-86b2-4b2d-bab1-72f78fedeb4d | Carnivora | Canidae | Vulpes pallida | 17.150350 | -13.878500 | L5 |
| 88019276-5a7f-40af-b2e0-2cfda99dc5d1 | Carnivora | Canidae | Vulpes pallida | 17.343400 | -13.625315 | L5 |
| 10c76f80-209f-4255-abe3-c794004117c1 | Carnivora | Canidae | Vulpes pallida | 17.416823 | -13.436495 | L5 |
| 106d7791-7cb5-4ac5-b47e-6bccb0415571 | Carnivora | Canidae | Vulpes pallida | 16.326600 | -13.930200 | M5 |
| 52abbe78-51d9-4b58-bcae-5dfa4c4f584e | Carnivora | Canidae | Vulpes pallida | 17.126187 | -14.065450 | L5 |
| 04bd67ad-4e2a-4bc5-ac88-b7086bd64e7e | Carnivora | Canidae | Vulpes pallida | 17.152482 | -12.199115 | L7 |
| 2ac69cfd-931e-4fda-8369-b52159b4cd7f | Carnivora | Canidae | Vulpes pallida | 17.927245 | -12.136773 | K7 |
| 4819cbf4-50cd-4d4c-97db-4a3b6c293263 | Carnivora | Canidae | Vulpes pallida | 17.981575 | -12.189942 | K7 |
| 8077bd58-194b-426d-995f-1709b61c8be9 | Carnivora | Canidae | Vulpes pallida | 16.211545 | -11.419975 | M7 |
| 18eb3fb8-ee2b-4af9-87ee-7e34fb851141 | Carnivora | Canidae | Vulpes pallida | 16.423790 | -11.362582 | M8 |
| 89bef523-3f96-4248-ac18-ea35ef59793c | Carnivora | Canidae | Vulpes pallida | 16.487108 | -10.680413 | M8 |
| 912d289e-1dd7-4d6c-95ea-0ca5c4038116 | Carnivora | Canidae | Vulpes pallida | 16.389973 | -10.305747 | M9 |
| 0ee61109-99ce-4de2-9f7f-3733dd90180b | Carnivora | Canidae | Vulpes pallida | 16.866805 | -9.572862 | L9 |
| 2811f7c2-a778-4193-97d9-74ca43717fbf | Carnivora | Canidae | Vulpes pallida | 16.980457 | -10.117865 | L9 |
| 197e8131-f3bf-4cf9-a7f7-8a032297048d | Carnivora | Canidae | Vulpes pallida | 16.899167 | -11.870033 | L7 |
| d4c6fe18-ddac-4d76-9181-7c00fc7116ff | Carnivora | Canidae | Vulpes pallida | 16.224017 | -13.231067 | M6 |
| b64ed248-6c7c-4116-83f0-10b837692bb3 | Carnivora | Canidae | Vulpes pallida | 16.303150 | -13.882250 | M5 |
| c0a23ae5-5d9f-43f6-b8ec-d0c58105fb94 | Carnivora | Canidae | Vulpes pallida | 16.889500 | -14.982800 | L4 |
| ab751803-56d4-4910-94fd-9e1282639b76 | Carnivora | Canidae | Vulpes pallida | 16.933650 | -16.121267 | L3 |
| c2d7f8ab-8357-4dd7-bb16-71c379dd6351 | Carnivora | Canidae | Vulpes pallida | 17.056467 | -16.085400 | L3 |
| 83422942-351c-4dc3-8258-5ed18da01651 | Carnivora | Canidae | Vulpes pallida | 17.242067 | -16.106700 | L3 |
| abb679f0-37d6-4795-957f-6b6b1e95507f | Carnivora | Canidae | Vulpes pallida | 17.347933 | -12.432883 | L6 |
| 34bf6652-8ef3-45c5-9ba8-6a90836674f1 | Carnivora | Canidae | Vulpes pallida | 17.291133 | -12.355817 | L6 |
| 00a4e262-d15f-4b97-afc2-68e8e12844ab | Carnivora | Canidae | Vulpes pallida | 16.514817 | -10.779683 | M8 |
| f684acc4-0b77-49e6-9706-e8fa43896527 | Carnivora | Canidae | Vulpes pallida | 16.517833 | -10.812583 | M8 |
| 72ddb054-116f-4043-9310-25846979122f | Carnivora | Canidae | Vulpes pallida | 16.997000 | -11.142983 | L8 |
| 9f1b6d74-16e0-424c-a29b-cf5b2ab6e5ee | Carnivora | Canidae | Vulpes pallida | 17.217117 | -11.110433 | L8 |
| b3b50d91-f9b3-43a8-b047-4247db011606 | Carnivora | Canidae | Vulpes pallida | 17.252583 | -14.275150 | L4 |
| db2a6638-f158-4f76-8871-f54c193e4695 | Carnivora | Canidae | Vulpes pallida | 17.278717 | -13.769900 | L5 |
| e1bb35ba-db35-4511-85f7-525589284a8c | Carnivora | Canidae | Vulpes pallida | 17.278717 | -13.769900 | L5 |
| d2268009-4f8b-45cb-ba24-fcf00e8432ce | Carnivora | Canidae | Vulpes pallida | 16.441450 | -10.466417 | M8 |
| 909448d9-b1f7-47a9-9d3a-88768a7d6e41 | Carnivora | Canidae | Vulpes pallida | 16.735550 | -7.398300 | L12 |
| http://n2t.net/ark:/65665/3ad3000a9-c837-4cf3-869d-6902e9bc06c0 | Carnivora | Canidae | Vulpes rueppellii | 22.678530 | -12.707130 | F6 |
| http://n2t.net/ark:/65665/31e43bd33-d216-422e-a5a6-8bddee9e19ef | Carnivora | Canidae | Vulpes rueppellii | 22.678530 | -12.707130 | F6 |
| http://n2t.net/ark:/65665/3cf900016-e1b0-42ea-9873-dc1c05d70277 | Carnivora | Canidae | Vulpes rueppellii | 22.678530 | -12.707130 | F6 |
| http://n2t.net/ark:/65665/31819b72f-e4ae-4928-8352-a4b87815d114 | Carnivora | Canidae | Vulpes rueppellii | 22.678530 | -12.707130 | F6 |
| http://n2t.net/ark:/65665/3bb24314f-64c4-48fc-8d88-d43c358c1da4 | Carnivora | Canidae | Vulpes rueppellii | 22.678530 | -12.707130 | F6 |
| http://n2t.net/ark:/65665/3f916472b-688c-46b0-82f0-56386e37dc39 | Carnivora | Canidae | Vulpes rueppellii | 22.678530 | -12.707130 | F6 |
| http://n2t.net/ark:/65665/37a6f174d-6935-482a-bd86-1272b1fdc124 | Carnivora | Canidae | Vulpes rueppellii | 21.525000 | -12.860600 | G6 |
| http://n2t.net/ark:/65665/32a4cc6d8-eef7-4fca-ab4f-55bb28f78bb6 | Carnivora | Canidae | Vulpes rueppellii | 21.525000 | -12.860600 | G6 |
| https://www.inaturalist.org/observations/46121163 | Carnivora | Canidae | Vulpes rueppellii | 19.682766 | -14.645144 | I4 |
| 99a2c1ec-aff8-490d-b998-2c01b32572ee | Carnivora | Canidae | Vulpes rueppellii | 19.156327 | -14.969700 | J4 |
| 13f762a1-2970-4369-a1f4-26f31ce495b5 | Carnivora | Canidae | Vulpes rueppellii | 18.958312 | -13.348447 | J5 |
| 79e7ddc5-9b6b-47f5-af81-6875b0229181 | Carnivora | Canidae | Vulpes rueppellii | 18.958312 | -13.348447 | J5 |
| 9bc0072b-79b8-4f8f-b7e6-5c5c9037a003 | Carnivora | Canidae | Vulpes rueppellii | 18.958312 | -13.348447 | J5 |
| afc65508-a943-43c3-b2b4-ca3abef83614 | Carnivora | Canidae | Vulpes rueppellii | 18.958312 | -13.348447 | J5 |
| 109c95f8-9274-4a3d-9f90-6e9719cf877a | Carnivora | Canidae | Vulpes rueppellii | 18.695828 | -14.075357 | J5 |
| 06b9e19c-c2e2-4249-8bfb-949e68a7bac7 | Carnivora | Canidae | Vulpes rueppellii | 18.447222 | -10.683298 | J8 |
| 56d60c73-5cd3-4900-b6f8-82055d2a6b99 | Carnivora | Canidae | Vulpes rueppellii | 18.380343 | -8.272207 | J11 |
| 1be7aa74-226a-49f4-8e92-a6bd0120f7c2 | Carnivora | Canidae | Vulpes rueppellii | 18.099567 | -8.010722 | K11 |
| 2f868b0a-1183-40df-a7b2-6853b14b2137 | Carnivora | Canidae | Vulpes rueppellii | 18.206040 | -11.730977 | K7 |
| f5bc58a4-5f94-42c2-ba2e-4c4c59bcf783 | Carnivora | Canidae | Vulpes rueppellii | 18.447222 | -10.683298 | J8 |
| 4e1d8b53-3a3e-46da-95a1-7bb13dfc144b | Carnivora | Canidae | Vulpes rueppellii | 18.150443 | -12.065303 | K7 |
| 61fa1b88-7255-4229-9f1e-6438e9883b0c | Carnivora | Canidae | Vulpes rueppellii | 18.367478 | -9.048450 | J10 |
| b2330559-a4fe-4251-b211-d1427c369773 | Carnivora | Canidae | Vulpes rueppellii | 18.383822 | -8.521617 | J10 |
| e113ed0f-2c25-40d8-8938-e8219e8fdd22 | Carnivora | Canidae | Vulpes rueppellii | 18.206040 | -11.730977 | K7 |
| af1fb859-cb54-48b2-a4f5-b2029008f476 | Carnivora | Canidae | Vulpes rueppellii | 18.367478 | -9.048450 | J10 |
| 75ceef1b-d7f1-4fc3-9e3e-69c478a0cff4 | Carnivora | Canidae | Vulpes rueppellii | 18.367478 | -9.048450 | J10 |
| c6cfcb81-79a8-45fa-a958-642980426578 | Carnivora | Canidae | Vulpes rueppellii | 18.401312 | -8.573102 | J10 |
| 46611543-3bca-4130-a710-dc52c3a4cb45 | Carnivora | Canidae | Vulpes rueppellii | 18.380343 | -8.272207 | J11 |
| 4eb0e1b8-2518-40a8-b9fb-bf39fb1211af | Carnivora | Canidae | Vulpes rueppellii | 18.380343 | -8.272207 | J11 |
| 36db0322-7902-40db-8f5e-b921cf4a0dc1 | Carnivora | Canidae | Vulpes rueppellii | 17.472420 | -7.424292 | K12 |
| dcbf890a-9407-405c-a24f-3e1ce41c6ce3 | Carnivora | Canidae | Vulpes rueppellii | 16.426157 | -9.565820 | M9 |
| f2f87e87-fd6b-40b4-a439-033db2433b21 | Carnivora | Canidae | Vulpes rueppellii | 17.530940 | -12.753980 | K6 |
| 7e12b49b-bcb8-4a82-b69c-70dbb1b52b7c | Carnivora | Canidae | Vulpes rueppellii | 19.911548 | -15.926908 | I3 |
| b3e8e324-66a4-49bc-b2e9-1a7526e859db | Carnivora | Canidae | Vulpes rueppellii | 20.394535 | -16.124797 | H2 |
| b02bb04e-a7bb-4c9d-a2b6-72cf303111b8 | Carnivora | Canidae | Vulpes rueppellii | 17.840183 | -12.410738 | K6 |
| 469fb14d-584a-44c2-a2ca-2a6aca51535c | Carnivora | Canidae | Vulpes rueppellii | 20.323193 | -13.142101 | H6 |
| 5c7deca6-292f-4506-88b0-7a3d24b5b1fa | Carnivora | Canidae | Vulpes rueppellii | 20.323193 | -13.142101 | H6 |
| 91dc4193-2fb8-4498-ab85-96c4d65ccfdf | Carnivora | Canidae | Vulpes rueppellii | 20.577380 | -12.492950 | H6 |
| fa3d19aa-ab97-4293-b1c0-13b2bcadd228 | Carnivora | Canidae | Vulpes rueppellii | 20.539352 | -12.791000 | H6 |
| 95ad3a09-7c1c-49bc-b3a0-7cb5c7f7c0fc | Carnivora | Canidae | Vulpes rueppellii | 20.504367 | -12.845628 | H6 |
| 673e9173-47b3-4c41-a7a1-93e0684e78d6 | Carnivora | Canidae | Vulpes rueppellii | 21.519632 | -12.853367 | G6 |
| 361f823e-225d-487e-a2b7-e5829a31837d | Carnivora | Canidae | Vulpes rueppellii | 20.484580 | -15.612723 | H3 |
| d7a648e5-f1bc-4a7a-9549-66a53b99581e | Carnivora | Canidae | Vulpes rueppellii | 20.626211 | -16.245192 | H2 |
| 09ff5074-8fb8-4cb7-9d79-3bb4f7699adf | Carnivora | Canidae | Vulpes rueppellii | 17.444500 | -13.303700 | L5 |
| 8b84c0c0-8659-433b-ad14-019f66558de0 | Carnivora | Canidae | Vulpes rueppellii | 17.069876 | -12.688625 | L6 |
| 315c85f9-3ccc-455e-be0f-9fd22297ed71 | Carnivora | Canidae | Vulpes rueppellii | 20.101790 | -16.059905 | I3 |
| 47bbaabb-d9f8-4077-a08a-3c3e78b54d80 | Carnivora | Canidae | Vulpes rueppellii | 20.050058 | -16.058292 | I3 |
| b8bcafdd-c6e8-43de-a3df-e6c389167075 | Carnivora | Canidae | Vulpes rueppellii | 20.124443 | -16.259393 | I2 |
| 1682576f-d17c-4464-b4c1-5723105259bf | Carnivora | Canidae | Vulpes rueppellii | 17.333602 | -12.077818 | L7 |
| 24081988-4449-4639-87ee-a9162c10bcd1 | Carnivora | Canidae | Vulpes rueppellii | 18.665692 | -12.964818 | J6 |
| 4548226d-bbb0-4f02-bff4-a35d28e3a35c | Carnivora | Canidae | Vulpes rueppellii | 20.019590 | -13.052400 | I6 |
| 2865e0c6-50d1-489b-bdb1-7224eaf554eb | Carnivora | Canidae | Vulpes rueppellii | 19.282833 | -14.880233 | I4 |
| 359b7578-4154-4161-a6cc-333847979578 | Carnivora | Canidae | Vulpes rueppellii | 19.404533 | -14.781667 | I4 |
| 9e021389-8933-43b1-8cb7-2f541b23f027 | Carnivora | Canidae | Vulpes rueppellii | 19.538333 | -14.663333 | I4 |
| b1e4cd3b-da72-44e4-ab3a-e5bf91250019 | Carnivora | Canidae | Vulpes rueppellii | 20.512300 | -16.235433 | H2 |
| fde4102d-3b3b-4ac0-b53a-aeb23de848fd | Carnivora | Canidae | Vulpes rueppellii | 17.339167 | -14.388583 | L4 |
| 1a5bcb7d-c96c-4778-aa1e-ee661ab27509 | Carnivora | Canidae | Vulpes rueppellii | 17.039750 | -11.967483 | L7 |
| 1cb683bc-99da-491e-a9d2-d40c99b9c3a3 | Carnivora | Canidae | Vulpes rueppellii | 17.582900 | -7.455833 | K12 |
| https://observation.org/observation/97952857 | Carnivora | Canidae | Vulpes zerda | 16.609187 | -15.925373 | L3 |
| https://observation.org/observation/159546007 | Carnivora | Canidae | Vulpes zerda | 19.310387 | -15.542499 | I3 |
| 1dc304eb-6bae-4786-b879-2e0515ed1602 | Carnivora | Canidae | Vulpes zerda | 18.489588 | -14.643788 | J4 |
| db8f29ed-3654-491a-a919-54dbdc3e9de1 | Carnivora | Canidae | Vulpes zerda | 18.958312 | -13.348447 | J5 |
| e8a2d31d-0923-443e-9d0d-6a8ea1f4325b | Carnivora | Canidae | Vulpes zerda | 18.614890 | -14.312745 | J4 |
| dd0adc75-0bc1-4a59-aa0e-ccbaccca11e2 | Carnivora | Canidae | Vulpes zerda | 17.617150 | -7.461348 | K12 |
| 98c79580-ddf0-43ae-857f-2d9dadfe4859 | Carnivora | Canidae | Vulpes zerda | 18.578522 | -9.818463 | J9 |
| 1942074c-6112-4187-9a7e-dd1d58a734ac | Carnivora | Canidae | Vulpes zerda | 18.578522 | -9.818463 | J9 |
| 58effefc-e0ae-4792-b42b-0e674cbe03ff | Carnivora | Canidae | Vulpes zerda | 18.578522 | -9.818463 | J9 |
| bac7c015-49a6-450c-930b-f2cb7ccf6fae | Carnivora | Canidae | Vulpes zerda | 18.578522 | -9.818463 | J9 |
| 2976cf3a-c2c2-4c9f-8b2e-80c565b5e2ed | Carnivora | Canidae | Vulpes zerda | 18.575172 | -10.074283 | J9 |
| be13f088-30ad-4458-90a9-2ce0a00b178a | Carnivora | Canidae | Vulpes zerda | 18.472040 | -9.524915 | J9 |
| 6709fa82-c247-4352-8e4a-ff45cc366dda | Carnivora | Canidae | Vulpes zerda | 18.390480 | -8.554273 | J10 |
| 91a5db33-6214-470e-95a0-666da3a630f2 | Carnivora | Canidae | Vulpes zerda | 18.377455 | -8.500047 | J10 |
| 4611439b-8f87-42ca-bfcc-a306d98159f3 | Carnivora | Canidae | Vulpes zerda | 18.354675 | -8.458417 | J11 |
| 82239e51-bd9b-4cbf-bc4e-57d14508fae4 | Carnivora | Canidae | Vulpes zerda | 18.353218 | -8.454685 | J11 |
| ac681bdf-0182-484b-967c-31469e9852a2 | Carnivora | Canidae | Vulpes zerda | 18.353218 | -8.454685 | J11 |
| fe6f5d44-d320-4c78-adfc-890b8e079081 | Carnivora | Canidae | Vulpes zerda | 18.340480 | -8.447220 | J11 |
| 89886c7d-d12c-43dd-8c67-5a282319a6d1 | Carnivora | Canidae | Vulpes zerda | 18.350298 | -8.349573 | J11 |
| 3d35df84-df3f-4289-a3dd-d5422b6b1666 | Carnivora | Canidae | Vulpes zerda | 17.691123 | -7.488007 | K12 |
| 041fbfa9-87ac-423c-a3b4-0646b1b71830 | Carnivora | Canidae | Vulpes zerda | 17.589240 | -7.445895 | K12 |
| 34a87e24-9b6a-464b-8175-74afb01b0afc | Carnivora | Canidae | Vulpes zerda | 21.433160 | -11.758860 | G7 |
| 90c5e971-4446-4dd0-b55c-5df19aff6432 | Carnivora | Canidae | Vulpes zerda | 19.959787 | -16.084098 | I3 |
| d6de4b97-df04-4b99-871c-d5bab79dc09f | Carnivora | Canidae | Vulpes zerda | 19.567590 | -16.383732 | I2 |
| d2f3c672-346a-4667-b591-08924dbd84e5 | Carnivora | Canidae | Vulpes zerda | 19.422068 | -16.297312 | I2 |
| f041e5f9-55c6-4b4f-9f9e-469e28b7cc95 | Carnivora | Canidae | Vulpes zerda | 19.984442 | -13.945550 | I5 |
| f0ab4382-5af7-415d-8f40-b4e09200d236 | Carnivora | Canidae | Vulpes zerda | 21.372827 | -11.910393 | G7 |
| 79eea5b3-90a2-40ca-b992-7c2ab67155f2 | Carnivora | Canidae | Vulpes zerda | 20.501642 | -12.866253 | H6 |
| eccd9a52-6a5c-4d9e-bf9d-d7fa84712cfd | Carnivora | Canidae | Vulpes zerda | 21.931075 | -12.745382 | G6 |
| dd57136b-0a5b-4c86-b211-e760e1fd57f9 | Carnivora | Canidae | Vulpes zerda | 21.931075 | -12.745382 | G6 |
| 903ef0e1-3d20-4709-a669-d36d6d00fac6 | Carnivora | Canidae | Vulpes zerda | 22.064895 | -12.711007 | F6 |
| a25d45e9-f970-4bad-9f02-308d59c199a1 | Carnivora | Canidae | Vulpes zerda | 21.678860 | -12.827375 | G6 |
| 9a74822b-6b67-4e81-a1a1-c532a830c919 | Carnivora | Canidae | Vulpes zerda | 21.678860 | -12.827375 | G6 |
| 529355ab-9166-4ef0-b821-2f6cc61ca1c0 | Carnivora | Canidae | Vulpes zerda | 21.205183 | -14.182682 | G4 |
| 340e7023-d5e4-43b5-810b-48e31a004f9e | Carnivora | Canidae | Vulpes zerda | 21.197600 | -14.222085 | G4 |
| e64cdae5-07dc-4c2a-b169-499d86e20013 | Carnivora | Canidae | Vulpes zerda | 21.197600 | -14.222085 | G4 |
| 4e727ce0-574c-49db-8f55-48ffc0a83fb6 | Carnivora | Canidae | Vulpes zerda | 21.197600 | -14.222085 | G4 |
| 95beca2a-ad53-45ee-babb-e2f4672ec6a8 | Carnivora | Canidae | Vulpes zerda | 21.262528 | -14.580797 | G4 |
| d3139ffa-3a33-4fef-9a81-46a7355d7b53 | Carnivora | Canidae | Vulpes zerda | 21.246962 | -14.584672 | G4 |
| 771ea7dd-0fb3-43ed-9bde-3ec69ef632b9 | Carnivora | Canidae | Vulpes zerda | 21.242615 | -14.582155 | G4 |
| a82c014e-2976-4027-8901-4ccd174d7567 | Carnivora | Canidae | Vulpes zerda | 21.242615 | -14.582155 | G4 |
| 27f4b265-fa2d-43b3-ab19-cbaa8c2ba631 | Carnivora | Canidae | Vulpes zerda | 21.236745 | -14.553275 | G4 |
| bf9615af-ecec-499b-96a6-6facfaa437a4 | Carnivora | Canidae | Vulpes zerda | 19.906568 | -16.009275 | I3 |
| 8b5fbac8-f4cb-44eb-9084-3ecca1160418 | Carnivora | Canidae | Vulpes zerda | 19.712827 | -16.060380 | I3 |
| 570a783f-427d-47f1-b65c-c288ac3a6591 | Carnivora | Canidae | Vulpes zerda | 19.158875 | -15.871528 | J3 |
| 249eb550-222d-4d7c-a5c3-df59528375ab | Carnivora | Canidae | Vulpes zerda | 19.387145 | -15.445718 | I3 |
| f067332f-c39f-4c13-9e18-64d7faaab6f8 | Carnivora | Canidae | Vulpes zerda | 19.437492 | -15.379233 | I3 |
| 2b93a9e6-50e5-4795-8c84-cc1e9d677141 | Carnivora | Canidae | Vulpes zerda | 18.585867 | -12.454410 | J6 |
| ab69bc66-070c-4a4d-a154-cccbfa099c0f | Carnivora | Canidae | Vulpes zerda | 18.594265 | -12.505532 | J6 |
| 963a379e-0027-4dce-8397-88fdebdf8e89 | Carnivora | Canidae | Vulpes zerda | 18.567048 | -12.912138 | J6 |
| ba268db5-8e2c-4320-8d10-46f8961a1443 | Carnivora | Canidae | Vulpes zerda | 18.776025 | -12.991767 | J6 |
| cd6791ba-0f57-44ab-b4e8-8940c2441aa9 | Carnivora | Canidae | Vulpes zerda | 19.488942 | -13.063735 | I6 |
| d45479bc-3a85-4b1c-a064-fe70abd48edd | Carnivora | Canidae | Vulpes zerda | 20.257798 | -14.310427 | H4 |
| 60ca0ed8-cb73-4073-b7c7-751367bc280c | Carnivora | Canidae | Vulpes zerda | 19.771495 | -16.187817 | I2 |
| 5966cb9a-28b5-462f-80e9-d7f9478f2a55 | Carnivora | Canidae | Vulpes zerda | 19.042855 | -11.924210 | J7 |
| f3799f2a-cf78-4fd7-8aa5-9c58cdbe0496 | Carnivora | Canidae | Vulpes zerda | 21.183333 | -13.750000 | G5 |
| d7605f9f-ad45-486d-a088-05e3208ab319 | Carnivora | Canidae | Vulpes zerda | 18.452950 | -14.733633 | J4 |
| a588e58e-90a8-44b2-8492-e06ffa33d873 | Carnivora | Canidae | Vulpes zerda | 18.471833 | -14.691100 | J4 |
| 01c4f360-13a4-4de2-8d82-1c0966038ab3 | Carnivora | Canidae | Vulpes zerda | 19.101500 | -16.246200 | J2 |
| 040a6ec3-3675-4d90-9660-2f78202713c0 | Carnivora | Canidae | Vulpes zerda | 18.121433 | -8.050067 | K11 |
| 0dd41cb8-a000-450c-a5a8-6bacd832fd5b | Carnivora | Felidae | Caracal caracal | 15.944687 | -11.929082 | M7 |
| 2756b160-e310-4484-a26a-26bbb1914239 | Carnivora | Felidae | Felis margarita | 18.406092 | -9.352792 | J10 |
| b5ed7733-84fb-4cc6-9cb7-5ddb18cf481e | Carnivora | Felidae | Felis margarita | 20.754297 | -16.618765 | H2 |
| 1d6c3f20-6619-4be3-b1e1-24a44dd982e2 | Carnivora | Felidae | Felis margarita | 19.227675 | -16.131643 | J2 |
| http://n2t.net/ark:/65665/302a15155-ea72-464b-a41f-58385cb022d1 | Carnivora | Felidae | Felis silvestris lybica | 16.150000 | -13.500000 | M5 |
| http://n2t.net/ark:/65665/3da421cd4-0ec4-4197-937c-dc2fc4229004 | Carnivora | Felidae | Felis silvestris lybica | 16.650000 | -14.283333 | L4 |
| http://n2t.net/ark:/65665/35e4f93f1-90cf-43fa-9823-f9569d8704f5 | Carnivora | Felidae | Felis silvestris lybica | 16.650000 | -14.283333 | L4 |
| http://n2t.net/ark:/65665/312c8c449-d03d-4e6e-a7b8-74d8ff2a3cba | Carnivora | Felidae | Felis silvestris lybica | 17.030000 | -13.920000 | L5 |
| http://n2t.net/ark:/65665/30de2dbd4-134d-424b-b538-107fc241b316 | Carnivora | Felidae | Felis silvestris lybica | 17.030000 | -13.920000 | L5 |
| http://n2t.net/ark:/65665/3512867a5-0f16-499f-9645-20769dbbfbdc | Carnivora | Felidae | Felis silvestris lybica | 16.650000 | -13.920000 | L5 |
| ce7b3cc4-5973-4f58-affc-fb137dec1c5e | Carnivora | Felidae | Felis silvestris lybica | 16.665295 | -9.882608 | L9 |
| 95612f37-3d34-40b2-9c83-0c9c6fc837e5 | Carnivora | Felidae | Felis silvestris lybica | 15.551130 | -11.087958 | N8 |
| 71179e19-80de-4e7b-8211-acfbded9fa65 | Carnivora | Felidae | Felis silvestris lybica | 16.665295 | -9.882608 | L9 |
| 266bcc97-9c38-4e89-8137-497815935bc5 | Carnivora | Felidae | Felis silvestris lybica | 18.402697 | -14.832732 | J4 |
| af670bef-0035-499a-b0b1-c2482484ec85 | Carnivora | Felidae | Felis silvestris lybica | 18.489588 | -14.643788 | J4 |
| 2e61aeed-e5bd-4766-a378-fef298d93cc4 | Carnivora | Felidae | Felis silvestris lybica | 16.483018 | -10.652837 | M8 |
| 92c89fd9-87e3-4520-8f4e-e1053d180b40 | Carnivora | Felidae | Felis silvestris lybica | 16.484132 | -9.289523 | M10 |
| 447d3cc7-ecdf-4624-9204-a0840c46cd04 | Carnivora | Felidae | Felis silvestris lybica | 16.484132 | -9.289523 | M10 |
| 728d84fb-ee7d-4cd5-b344-9a63b347bf8b | Carnivora | Felidae | Felis silvestris lybica | 18.356815 | -11.816080 | J7 |
| 778354f8-67ac-4e89-b667-8f4c77dc1c62 | Carnivora | Felidae | Felis silvestris lybica | 16.484132 | -9.289523 | M10 |
| d2ab616a-9689-4172-8feb-e528c19a8e35 | Carnivora | Felidae | Felis silvestris lybica | 17.294777 | -7.046927 | L12 |
| 2eba1be4-4365-4d15-a6ab-79655cc9a030 | Carnivora | Felidae | Felis silvestris lybica | 16.416477 | -8.957903 | M10 |
| bdf9edc0-974b-4066-ae45-13e32faf9fa7 | Carnivora | Felidae | Felis silvestris lybica | 16.628275 | -16.231821 | L2 |
| d396a7f0-27c0-4c34-ab0d-9042c0c1d5bb | Carnivora | Felidae | Felis silvestris lybica | 16.444350 | -11.778073 | M7 |
| d0b6e548-c97c-4ac0-b075-cf648ad5ce73 | Carnivora | Felidae | Felis silvestris lybica | 16.684655 | -11.291553 | L8 |
| 9325b533-4889-4d8b-9373-ed6c6d74b550 | Carnivora | Felidae | Felis silvestris lybica | 19.563600 | -16.396827 | I2 |
| df7440b2-5131-4c69-a2b4-107f028fc55b | Carnivora | Felidae | Felis silvestris lybica | 17.212027 | -13.840818 | L5 |
| 6c49dadb-a0df-446d-b59b-128114a828ba | Carnivora | Felidae | Felis silvestris lybica | 17.128420 | -12.594278 | L6 |
| f98aecda-9799-4e78-900c-17a7a9501c72 | Carnivora | Felidae | Felis silvestris lybica | 17.702622 | -14.570367 | K4 |
| ec9e6bb5-99c0-43d9-a65c-ba370d85bce3 | Carnivora | Felidae | Felis silvestris lybica | 16.746288 | -11.622208 | L7 |
| 8ada8d1d-b439-4cb0-ac5f-52d184cfeb45 | Carnivora | Felidae | Felis silvestris lybica | 16.389037 | -10.303163 | M9 |
| 8651db28-9094-4c7c-b585-e62e65fa1816 | Carnivora | Felidae | Felis silvestris lybica | 16.468027 | -10.028762 | M9 |
| eb6ac746-9040-482e-8cc8-0b4cf9d56ff8 | Carnivora | Felidae | Felis silvestris lybica | 16.864255 | -9.583395 | L9 |
| ce431828-c778-4983-b3ac-1a7132c321eb | Carnivora | Felidae | Felis silvestris lybica | 16.900395 | -10.178290 | L9 |
| af5b38e8-5b23-46af-ba69-d602f58321a4 | Carnivora | Felidae | Felis silvestris lybica | 17.039750 | -11.967483 | L7 |
| 9b115186-e9ff-4dd4-acb6-6ad230963ad5 | Carnivora | Herpestidae | Herpestes sanguineus | 15.944687 | -11.929082 | M7 |
| e323c78a-ce38-4c0a-868c-d3a839b8838a | Carnivora | Herpestidae | Herpestes sanguineus | 16.530060 | -11.806630 | M7 |
| 089690b1-440b-4c0c-9cf1-b61146f326e0 | Carnivora | Herpestidae | Herpestes sanguineus | 17.157260 | -11.932307 | L7 |
| 6fce55fc-7c15-480d-921b-99382dd8da39 | Carnivora | Herpestidae | Ichneumia albicauda | 16.579150 | -10.704550 | L8 |
| 909c3535-8d04-4708-b076-d10eebd81c22 | Carnivora | Herpestidae | Ichneumia albicauda | 16.579150 | -10.704550 | L8 |
| http://coldb.mnhn.fr/catalognumber/mnhn/zm/2006-510 | Carnivora | Mustelidae | Ictonyx libyca | 17.834850 | -11.557833 | K7 |
| http://n2t.net/ark:/65665/321529db3-997d-438f-9a8b-023e5e260d64 | Carnivora | Mustelidae | Ictonyx libyca | 17.030000 | -13.920000 | L5 |
| http://n2t.net/ark:/65665/3335038f0-d731-48b0-aad7-75103e4d92af | Carnivora | Mustelidae | Ictonyx libyca | 17.030000 | -13.920000 | L5 |
| http://n2t.net/ark:/65665/3f93fd446-3861-4525-a948-b7f2ae10d4f5 | Carnivora | Mustelidae | Ictonyx libyca | 17.030000 | -13.920000 | L5 |
| http://n2t.net/ark:/65665/341264ba2-082b-43f3-acaa-195df5fffc1c | Carnivora | Mustelidae | Ictonyx libyca | 17.030000 | -13.920000 | L5 |
| http://n2t.net/ark:/65665/3cf2695ca-5b53-4a9f-9436-f6ff0b0a7a4c | Carnivora | Mustelidae | Ictonyx libyca | 17.030000 | -13.920000 | L5 |
| 86ac6869-99b6-4c77-b93f-2a1a876113cc | Carnivora | Mustelidae | Ictonyx striatus | 16.318093 | -11.376447 | M7 |
| http://coldb.mnhn.fr/catalognumber/mnhn/zm/mo-1995-3150 | Carnivora | Mustelidae | Mellivora capensis | 17.283000 | -16.100000 | L3 |
| 155ad4ba-e8f1-40cb-ba9a-812a1dcac746 | Carnivora | Mustelidae | Mellivora capensis | 15.259737 | -12.483460 | N6 |
| 96a4be3a-c2b0-42c6-9fea-4948c5bddc78 | Carnivora | Mustelidae | Mellivora capensis | 16.483018 | -10.652837 | M8 |
| 0e2b4757-6e8b-45c6-be7f-2f61bc2194d4 | Carnivora | Mustelidae | Mellivora capensis | 16.275638 | -13.703690 | M5 |
| 51bebd05-9baa-4648-970e-1e3faa36813c | Carnivora | Mustelidae | Mellivora capensis | 15.117910 | -11.847563 | N7 |
| b5a83292-e9ad-470d-884f-7e86a83749f1 | Carnivora | Mustelidae | Mellivora capensis | 17.115870 | -13.575195 | L5 |
| d891127b-6270-4eeb-8af8-61533a3ce6b4 | Carnivora | Mustelidae | Mellivora capensis | 16.811947 | -11.958225 | L7 |
| be07fca7-2b9c-4fb2-b5d7-f5a096b4b7eb | Carnivora | Mustelidae | Mellivora capensis | 16.756482 | -11.997233 | L7 |
| 6f856587-46ac-406d-b84a-a2c3ae4f26ce | Carnivora | Mustelidae | Mellivora capensis | 16.019367 | -12.769100 | M6 |
| 45b74363-79ab-444c-ab9d-4abe3538b83d | Carnivora | Mustelidae | Mellivora capensis | 17.400717 | -16.063667 | L3 |
| 97239c4a-4eed-49de-b706-17040b29f750 | Carnivora | Mustelidae | Mellivora capensis | 17.584733 | -14.740867 | K4 |
| 203ac408-8aa3-4580-8fe1-f593f6074977 | Carnivora | Mustelidae | Mellivora capensis | 17.029417 | -13.957367 | L5 |
| 7f8d78a3-5748-4223-9998-50045cf87a1e | Carnivora | Mustelidae | Mellivora capensis | 17.278717 | -13.769900 | L5 |
| 61383163-482e-4478-a4ac-4b152018de90 | Carnivora | Viverridae | Civettictis civetta | 16.579150 | -10.704550 | L8 |
| http://n2t.net/ark:/65665/3a12466c3-2b83-46a3-bce2-cdffc4ee69b2 | Carnivora | Viverridae | Genetta genetta | 17.030000 | -13.920000 | L5 |
| http://n2t.net/ark:/65665/3ddef3285-cfce-440f-89f6-61c01327d2dc | Carnivora | Viverridae | Genetta genetta | 17.030000 | -13.920000 | L5 |
| http://n2t.net/ark:/65665/3fa24e4f1-3bdc-4150-8b8d-41ad231cd2e5 | Carnivora | Viverridae | Genetta genetta | 17.030000 | -13.920000 | L5 |
| http://n2t.net/ark:/65665/3ceea1c35-2c73-4d95-8d1f-87c7db52fd16 | Carnivora | Viverridae | Genetta genetta | 17.030000 | -13.920000 | L5 |
| 1ab14556-28f2-4ccb-9251-627618913113 | Carnivora | Viverridae | Genetta genetta | 15.250138 | -12.406722 | N6 |
| a2d02946-6c84-4fd2-a15d-317d1d0b8076 | Carnivora | Viverridae | Genetta genetta | 15.551130 | -11.087958 | N8 |
| 82402600-8dbd-47f6-ac36-5def82ffcf7a | Carnivora | Viverridae | Genetta genetta | 16.328193 | -11.481203 | M7 |
| 3080d242-2e62-4121-abc9-7259471dba65 | Carnivora | Viverridae | Genetta genetta | 16.608507 | -15.329743 | L3 |
| d007c9c6-efd3-494c-b3aa-346816ad3e7a | Carnivora | Viverridae | Genetta genetta | 16.547120 | -15.594075 | M3 |
| af6f98de-e6f9-4939-9e6a-688f4af926fa | Carnivora | Viverridae | Genetta genetta | 16.548878 | -15.720755 | M3 |
| 03140659-12c9-4486-a4e1-6dbe8ece6501 | Carnivora | Viverridae | Genetta genetta | 17.316903 | -16.079762 | L3 |
| bc4483eb-5a8e-41e6-b9ee-b31fb7b37883 | Carnivora | Viverridae | Genetta genetta | 15.598700 | -13.111170 | N6 |
| fd7ac425-eed0-4e69-898f-45b9f141c5e1 | Carnivora | Viverridae | Genetta genetta | 17.069876 | -12.688625 | L6 |
| b82081dc-5e20-4bad-adfd-33cc5d63a2eb | Carnivora | Viverridae | Genetta genetta | 16.547455 | -12.009590 | L7 |
| 6369fbed-8ef5-4489-9c06-4923b37b9ec1 | Carnivora | Viverridae | Genetta genetta | 16.303300 | -13.883800 | M5 |
| 4e77053d-bddf-48b4-b2e1-e5fc3cc98490 | Carnivora | Viverridae | Genetta genetta | 17.193703 | -14.174535 | L5 |
| 1828305a-2c73-4f96-95b2-61864e4b721f | Carnivora | Viverridae | Genetta genetta | 17.069876 | -12.688625 | L6 |
| 113c80f6-a8a1-4355-b56b-393ac836b7ad | Carnivora | Viverridae | Genetta genetta | 17.173397 | -11.938015 | L7 |
| 048ba7a1-fdf3-4262-a906-fa70df365636 | Carnivora | Viverridae | Genetta genetta | 19.729943 | -15.971117 | I3 |
| c3668173-5701-4740-b66a-713c1c5a664f | Carnivora | Viverridae | Genetta genetta | 17.552835 | -12.953093 | K6 |
| d4ebb0c7-edb9-4d9c-8ce2-03df00eb9529 | Carnivora | Viverridae | Genetta genetta | 16.948433 | -16.113667 | L3 |
| 6d144a9b-ed84-431c-a5f6-13efe741fc9b | Carnivora | Viverridae | Genetta genetta | 16.506533 | -10.414783 | M8 |
|  | Chiroptera | Emballonuridae | Taphozous nudiventris | 16.661020 | -9.616550 | L9 |
|  | Chiroptera | Emballonuridae | Taphozous nudiventris | 16.661020 | -9.616550 | L9 |
| http://n2t.net/ark:/65665/3e7775bce-ca28-42b4-a362-b76b1dc9509a | Chiroptera | Emballonuridae | Taphozous perforatus | 16.650000 | -14.283333 | L4 |
| 873888271824FFC7CEECC617E1F46DDC.mc.BFF9336C1824FFC8CDC3C118E0236F61 | Chiroptera | Hipposideridae | Asellia tridens | 20.620276 | -14.220408 | H4 |
| http://coldb.mnhn.fr/catalognumber/mnhn/zm/2008-95 | Chiroptera | Hipposideridae | Asellia tridens | 20.252804 | -13.088188 | H6 |
| 873888271824FFC7CEECC617E1F46DDC.mc.BFF9336C1824FFC8CB06C0D2E7A06F1B | Chiroptera | Hipposideridae | Asellia tridens | 20.252804 | -13.088188 | H6 |
| 873888271824FFC7CEECC617E1F46DDC.mc.BFF9336C1824FFC8CA06C0C8E1766ECD | Chiroptera | Hipposideridae | Asellia tridens | 20.932200 | -11.622333 | H7 |
| http://coldb.mnhn.fr/catalognumber/mnhn/zm/mo-1993-4320 | Chiroptera | Hipposideridae | Asellia tridens | 20.536743 | -12.961179 | H6 |
| http://coldb.mnhn.fr/catalognumber/mnhn/zm/mo-1993-4319 | Chiroptera | Hipposideridae | Asellia tridens | 20.536743 | -12.961179 | H6 |
| http://n2t.net/ark:/65665/33e4d8b2a-1f30-44a9-88f2-ca419241f466 | Chiroptera | Hipposideridae | Asellia tridens | 17.030000 | -13.920000 | L5 |
| http://n2t.net/ark:/65665/3bd255c3c-20d0-42bc-acf0-4af3b93eceef | Chiroptera | Hipposideridae | Asellia tridens | 17.030000 | -13.920000 | L5 |
| http://n2t.net/ark:/65665/35aaad01f-fe0e-4f6f-bd6b-fde9b3301d04 | Chiroptera | Hipposideridae | Asellia tridens | 17.030000 | -13.920000 | L5 |
| http://n2t.net/ark:/65665/384596815-ac54-4598-b40a-da1d21dd91ae | Chiroptera | Hipposideridae | Asellia tridens | 17.030000 | -13.920000 | L5 |
| http://n2t.net/ark:/65665/31a797410-4ec7-48a5-8488-3fcad4d8c80f | Chiroptera | Hipposideridae | Asellia tridens | 17.030000 | -13.920000 | L5 |
| http://n2t.net/ark:/65665/3d18d4513-e845-4ca7-b056-c6ed006ffe44 | Chiroptera | Hipposideridae | Asellia tridens | 17.030000 | -13.920000 | L5 |
| http://n2t.net/ark:/65665/3bda8c164-be3a-4da2-899e-80220682fb3e | Chiroptera | Hipposideridae | Asellia tridens | 17.030000 | -13.920000 | L5 |
| http://n2t.net/ark:/65665/3d1776a66-668c-4395-bd08-410bc700e65a | Chiroptera | Hipposideridae | Asellia tridens | 17.030000 | -13.920000 | L5 |
| http://n2t.net/ark:/65665/32bc1b57a-36ad-4f6b-80da-a32b4792a1ec | Chiroptera | Hipposideridae | Asellia tridens | 17.030000 | -13.920000 | L5 |
| http://n2t.net/ark:/65665/31cd84b50-94d6-4af7-91cb-ddf7dbb85f2a | Chiroptera | Hipposideridae | Asellia tridens | 17.030000 | -13.920000 | L5 |
| http://n2t.net/ark:/65665/3bb9590f9-7cb6-4fce-9273-6e8cde0a37fe | Chiroptera | Hipposideridae | Asellia tridens | 17.030000 | -13.920000 | L5 |
| http://n2t.net/ark:/65665/3c21dae98-ec36-4c2b-be92-2985878502da | Chiroptera | Hipposideridae | Asellia tridens | 17.030000 | -13.920000 | L5 |
| http://n2t.net/ark:/65665/34d7b9f82-f4ff-48f1-8ca4-d4056063d384 | Chiroptera | Hipposideridae | Asellia tridens | 17.030000 | -13.920000 | L5 |
| http://n2t.net/ark:/65665/35212dc3f-a512-4381-b40a-2e61e928cb4a | Chiroptera | Hipposideridae | Asellia tridens | 17.030000 | -13.920000 | L5 |
| http://n2t.net/ark:/65665/37fb92465-473a-45c1-a2f1-170fda555664 | Chiroptera | Hipposideridae | Asellia tridens | 17.030000 | -13.920000 | L5 |
| http://n2t.net/ark:/65665/308fe03f5-f2fe-4eec-b8ac-3b2523c13a84 | Chiroptera | Hipposideridae | Asellia tridens | 17.030000 | -13.920000 | L5 |
| http://n2t.net/ark:/65665/3d7eeb013-e006-4a94-b72c-e4a151fe532e | Chiroptera | Hipposideridae | Asellia tridens | 17.030000 | -13.920000 | L5 |
| http://n2t.net/ark:/65665/3e338906d-ac76-43c1-9eff-09dbe422915b | Chiroptera | Hipposideridae | Asellia tridens | 17.030000 | -13.920000 | L5 |
| http://n2t.net/ark:/65665/398608b15-3cc6-43b6-985a-233127218cf9 | Chiroptera | Hipposideridae | Asellia tridens | 17.030000 | -13.920000 | L5 |
| http://n2t.net/ark:/65665/3631d373f-d460-44b9-b700-4cd8a4da6cfa | Chiroptera | Hipposideridae | Asellia tridens | 17.030000 | -13.920000 | L5 |
| http://n2t.net/ark:/65665/3bb39fd7a-c167-4cff-b3b0-0b538a9d3f06 | Chiroptera | Hipposideridae | Asellia tridens | 17.030000 | -13.920000 | L5 |
| http://n2t.net/ark:/65665/3fb9cd71b-1df2-44a9-ab69-48780d428065 | Chiroptera | Hipposideridae | Asellia tridens | 17.030000 | -13.920000 | L5 |
| http://n2t.net/ark:/65665/3db6b1c1d-b071-4784-8d4d-729fac965cac | Chiroptera | Hipposideridae | Asellia tridens | 17.030000 | -13.920000 | L5 |
| http://n2t.net/ark:/65665/3c530ac01-aa1c-4cb2-a7c4-6e5d9e63305b | Chiroptera | Hipposideridae | Asellia tridens | 17.030000 | -13.920000 | L5 |
| http://n2t.net/ark:/65665/3556876a5-3cd4-42f3-b9e7-a8899065f181 | Chiroptera | Hipposideridae | Asellia tridens | 17.030000 | -13.920000 | L5 |
| http://n2t.net/ark:/65665/344b0e1ca-7f62-4404-ba94-b9a1ab1004cf | Chiroptera | Hipposideridae | Asellia tridens | 17.030000 | -13.920000 | L5 |
| http://n2t.net/ark:/65665/3e158ee65-b313-4f3e-9eb3-75eb757a25d1 | Chiroptera | Hipposideridae | Asellia tridens | 17.030000 | -13.920000 | L5 |
| http://n2t.net/ark:/65665/3ed442824-33de-4946-9b8e-cb8e7bbddc37 | Chiroptera | Hipposideridae | Asellia tridens | 17.030000 | -13.920000 | L5 |
| http://n2t.net/ark:/65665/34ae3cc18-8a64-4d80-8a06-e059a8c59707 | Chiroptera | Hipposideridae | Asellia tridens | 17.030000 | -13.920000 | L5 |
| http://n2t.net/ark:/65665/3e48dd2b3-615e-4c5b-9538-e3b2d4039c5e | Chiroptera | Hipposideridae | Asellia tridens | 17.030000 | -13.920000 | L5 |
| http://n2t.net/ark:/65665/3a7264ea0-0370-4c96-bb30-e5135e0578ea | Chiroptera | Hipposideridae | Asellia tridens | 17.030000 | -13.920000 | L5 |
| http://n2t.net/ark:/65665/330c2b63a-25d8-4f40-974b-7e3d78a4bc36 | Chiroptera | Hipposideridae | Asellia tridens | 17.030000 | -13.920000 | L5 |
| http://n2t.net/ark:/65665/381e17cde-b265-40fd-9d72-f564287d7b2c | Chiroptera | Hipposideridae | Asellia tridens | 17.030000 | -13.920000 | L5 |
| http://n2t.net/ark:/65665/3a8156549-9114-4c8b-be6f-196caddc4e2a | Chiroptera | Hipposideridae | Asellia tridens | 17.030000 | -13.920000 | L5 |
| http://n2t.net/ark:/65665/352c62c84-0359-4c8b-9a58-8d8dc16fd3dc | Chiroptera | Hipposideridae | Asellia tridens | 17.030000 | -13.920000 | L5 |
| http://n2t.net/ark:/65665/3ef9d3c77-6c7d-4f91-8909-6f324b267d1b | Chiroptera | Hipposideridae | Asellia tridens | 17.030000 | -13.920000 | L5 |
| http://n2t.net/ark:/65665/358c89ca5-e0fe-4a0f-89de-1735b9ad6cd5 | Chiroptera | Hipposideridae | Asellia tridens | 17.030000 | -13.920000 | L5 |
| http://n2t.net/ark:/65665/307d09650-0a23-436f-ae1b-aa5498397253 | Chiroptera | Hipposideridae | Asellia tridens | 17.030000 | -13.920000 | L5 |
| http://n2t.net/ark:/65665/38a6865db-00a8-47b6-b1e8-2a7ebae63206 | Chiroptera | Hipposideridae | Asellia tridens | 17.030000 | -13.920000 | L5 |
| http://n2t.net/ark:/65665/37bc56272-c488-4fa7-8a03-994cfbe3ea21 | Chiroptera | Hipposideridae | Asellia tridens | 17.030000 | -13.920000 | L5 |
| http://n2t.net/ark:/65665/3f9d7bcf8-b9b3-4919-8cb2-007947231486 | Chiroptera | Hipposideridae | Asellia tridens | 17.030000 | -13.920000 | L5 |
| http://n2t.net/ark:/65665/3fa097bc1-a3a6-425f-aa99-d97143218932 | Chiroptera | Hipposideridae | Asellia tridens | 17.030000 | -13.920000 | L5 |
| 25150d54-aa1d-4fc9-b927-a41260ed2cc2 | Chiroptera | Hipposideridae | Asellia tridens | 15.878200 | -12.039233 | M7 |
| 85176764 | Chiroptera | Hipposideridae | Hipposideros cf. caffer | 20.252804 | -13.088188 | H6 |
| http://coldb.mnhn.fr/catalognumber/mnhn/zm/2008-96 | Chiroptera | Hipposideridae | Hipposideros cf. caffer | 20.252804 | -13.088188 | H6 |
| http://n2t.net/ark:/65665/3d0734c60-4eb2-478c-809c-4f8e76f63af6 | Chiroptera | Hipposideridae | Hipposideros cf. caffer | 16.550000 | -15.766667 | M3 |
| http://coldb.mnhn.fr/catalognumber/mnhn/zm/mo-1913-664f | Chiroptera | Hipposideridae | Hipposideros cf. caffer | 17.546667 | -14.694444 | K4 |
| http://coldb.mnhn.fr/catalognumber/mnhn/zm/mo-1913-664e | Chiroptera | Hipposideridae | Hipposideros cf. caffer | 17.546667 | -14.694444 | K4 |
| http://coldb.mnhn.fr/catalognumber/mnhn/zm/mo-1913-664d | Chiroptera | Hipposideridae | Hipposideros cf. caffer | 17.546667 | -14.694444 | K4 |
| http://coldb.mnhn.fr/catalognumber/mnhn/zm/mo-1913-664c | Chiroptera | Hipposideridae | Hipposideros cf. caffer | 17.546667 | -14.694444 | K4 |
| http://coldb.mnhn.fr/catalognumber/mnhn/zm/mo-1913-664b | Chiroptera | Hipposideridae | Hipposideros cf. caffer | 17.546667 | -14.694444 | K4 |
| http://coldb.mnhn.fr/catalognumber/mnhn/zm/mo-1913-664a | Chiroptera | Hipposideridae | Hipposideros cf. caffer | 17.546667 | -14.694444 | K4 |
| http://coldb.mnhn.fr/catalognumber/mnhn/zm/mo-1913-663e | Chiroptera | Hipposideridae | Hipposideros cf. caffer | 17.546667 | -14.694444 | K4 |
| http://coldb.mnhn.fr/catalognumber/mnhn/zm/mo-1913-663d | Chiroptera | Hipposideridae | Hipposideros cf. caffer | 17.546667 | -14.694444 | K4 |
| http://coldb.mnhn.fr/catalognumber/mnhn/zm/mo-1913-663c | Chiroptera | Hipposideridae | Hipposideros cf. caffer | 17.546667 | -14.694444 | K4 |
| http://coldb.mnhn.fr/catalognumber/mnhn/zm/mo-1913-663b | Chiroptera | Hipposideridae | Hipposideros cf. caffer | 17.546667 | -14.694444 | K4 |
| http://coldb.mnhn.fr/catalognumber/mnhn/zm/mo-1913-663a | Chiroptera | Hipposideridae | Hipposideros cf. caffer | 17.546667 | -14.694444 | K4 |
| http://n2t.net/ark:/65665/3f7632686-8e15-43cb-8712-9d86451409b8 | Chiroptera | Hipposideridae | Hipposideros tephrus | 16.550000 | -15.766667 | M3 |
| http://n2t.net/ark:/65665/35fa2ae6e-b4c8-44eb-967e-9259ba918d59 | Chiroptera | Hipposideridae | Hipposideros tephrus | 16.550000 | -15.766667 | M3 |
| http://n2t.net/ark:/65665/35d83238a-f61b-4d07-aca2-e5cab3b107ac | Chiroptera | Hipposideridae | Hipposideros tephrus | 16.550000 | -15.766667 | M3 |
| http://n2t.net/ark:/65665/3e0cbfb64-7571-4a2e-8262-e7323a78629c | Chiroptera | Hipposideridae | Hipposideros tephrus | 16.550000 | -15.766667 | M3 |
| http://n2t.net/ark:/65665/328f837f5-f3e6-4d46-9098-24c86f38a95d | Chiroptera | Molossidae | Mops condylurus | 16.516667 | -15.816667 | M3 |
| http://n2t.net/ark:/65665/3fe0d198b-7d6d-45bb-84bb-d6deeb2bc01e | Chiroptera | Molossidae | Mops condylurus | 16.516667 | -15.816667 | M3 |
| http://n2t.net/ark:/65665/372639d7a-5d51-41bf-a7cd-1674aedbadb4 | Chiroptera | Molossidae | Mops condylurus | 16.516667 | -15.816667 | M3 |
| http://n2t.net/ark:/65665/31a0d3bb8-7d8e-4282-af5d-2be59a62f387 | Chiroptera | Molossidae | Mops condylurus | 16.516667 | -15.816667 | M3 |
| http://n2t.net/ark:/65665/3b8c776a0-e22c-4d56-9849-c7d19f601103 | Chiroptera | Molossidae | Mops condylurus | 16.516667 | -15.816667 | M3 |
| http://n2t.net/ark:/65665/37965d835-bb96-433d-99b0-26ee9f8eca49 | Chiroptera | Molossidae | Mops condylurus | 16.516667 | -15.816667 | M3 |
| http://n2t.net/ark:/65665/35c23b5e4-7012-4958-a6d0-d40fb4ab5561 | Chiroptera | Molossidae | Mops condylurus | 16.516667 | -15.816667 | M3 |
| http://n2t.net/ark:/65665/370be4802-5571-4c27-96ac-132785352f53 | Chiroptera | Molossidae | Mops condylurus | 16.516667 | -15.816667 | M3 |
| http://n2t.net/ark:/65665/3099dffa9-c9de-4708-98f0-51cc9b5640a1 | Chiroptera | Molossidae | Mops condylurus | 16.516667 | -15.816667 | M3 |
| http://n2t.net/ark:/65665/304bfced3-bd52-4d04-8a69-4b7cd44274f8 | Chiroptera | Molossidae | Mops condylurus | 16.516667 | -15.816667 | M3 |
| http://n2t.net/ark:/65665/38bf52621-fcb5-41b1-95aa-a154c801b5f2 | Chiroptera | Molossidae | Mops condylurus | 16.516667 | -15.816667 | M3 |
| http://n2t.net/ark:/65665/3cc35bcaa-ef3a-4142-a5ed-86cedd030271 | Chiroptera | Molossidae | Mops condylurus | 16.516667 | -15.816667 | M3 |
| http://n2t.net/ark:/65665/3f077146b-b020-451b-af9c-86e71c879b38 | Chiroptera | Molossidae | Mops condylurus | 16.516667 | -15.816667 | M3 |
| http://n2t.net/ark:/65665/3e2432b67-beb5-4acd-9374-dc3a092b2a84 | Chiroptera | Molossidae | Mops condylurus | 16.516667 | -15.816667 | M3 |
| http://n2t.net/ark:/65665/32d5d3f46-d7e2-48c1-9605-782a5dd49efd | Chiroptera | Molossidae | Mops condylurus | 16.516667 | -15.816667 | M3 |
| http://n2t.net/ark:/65665/3c936b6d4-1630-453d-bfc7-f8c82a07f444 | Chiroptera | Molossidae | Mops condylurus | 16.516667 | -15.816667 | M3 |
| http://n2t.net/ark:/65665/3dda2ee99-045f-4745-91c7-6c48f2bbfc79 | Chiroptera | Molossidae | Mops condylurus | 16.516667 | -15.816667 | M3 |
| http://n2t.net/ark:/65665/392398579-5bc4-4b1e-a378-0599e4f837cc | Chiroptera | Molossidae | Mops condylurus | 16.516667 | -15.816667 | M3 |
| http://n2t.net/ark:/65665/35875511e-c86f-4865-a90b-ce8396b36945 | Chiroptera | Molossidae | Mops condylurus | 16.516667 | -15.816667 | M3 |
| http://n2t.net/ark:/65665/376c4a736-e140-43fb-9c2c-74ec744309da | Chiroptera | Molossidae | Mops condylurus | 16.516667 | -15.816667 | M3 |
| http://n2t.net/ark:/65665/36af40de3-b994-40c0-8060-039a14d8e723 | Chiroptera | Molossidae | Mops condylurus | 16.516667 | -15.816667 | M3 |
| http://n2t.net/ark:/65665/31126e71f-93be-44bf-a1e4-353a7b2839a9 | Chiroptera | Molossidae | Mops condylurus | 16.516667 | -15.816667 | M3 |
| http://n2t.net/ark:/65665/35c7fc31c-1641-4411-aba2-4ecdc0359a6f | Chiroptera | Molossidae | Mops condylurus | 16.516667 | -15.816667 | M3 |
| http://n2t.net/ark:/65665/392853c59-cdc5-45c9-a98d-0177f3bc971c | Chiroptera | Molossidae | Mops condylurus | 16.516667 | -15.816667 | M3 |
| http://n2t.net/ark:/65665/32040e5b7-d87e-4a81-a6ee-96fc697073c8 | Chiroptera | Molossidae | Mops condylurus | 16.516667 | -15.816667 | M3 |
| http://n2t.net/ark:/65665/34af1861a-0a5c-4dc4-b835-cb5fecb70481 | Chiroptera | Molossidae | Mops condylurus | 16.516667 | -15.816667 | M3 |
| http://n2t.net/ark:/65665/36313b092-be1f-46d4-9a84-76427d6aab18 | Chiroptera | Molossidae | Mops condylurus | 16.516667 | -15.816667 | M3 |
| http://n2t.net/ark:/65665/3bac54ba0-e749-4ad2-b613-c9f0e41131ae | Chiroptera | Molossidae | Mops condylurus | 16.516667 | -15.816667 | M3 |
| http://n2t.net/ark:/65665/3294e13a1-1d99-487c-9145-91526cf79895 | Chiroptera | Molossidae | Mops condylurus | 16.516667 | -15.816667 | M3 |
| http://n2t.net/ark:/65665/325fa4e9a-cded-4451-8f7a-126bb7631c12 | Chiroptera | Molossidae | Mops condylurus | 16.516667 | -15.816667 | M3 |
| http://n2t.net/ark:/65665/3ba54111a-fef2-4a49-ae06-2da847352ad2 | Chiroptera | Molossidae | Mops condylurus | 16.516667 | -15.816667 | M3 |
| http://n2t.net/ark:/65665/35f36a137-75e4-4003-b4c4-93d19b4267b0 | Chiroptera | Molossidae | Mops condylurus | 16.516667 | -15.816667 | M3 |
| http://n2t.net/ark:/65665/32dde763b-3956-40bb-b7d7-c9ed1797076e | Chiroptera | Molossidae | Mops condylurus | 16.516667 | -15.816667 | M3 |
| http://n2t.net/ark:/65665/352fde345-2a67-4d9a-aa11-10db09a58faf | Chiroptera | Molossidae | Mops condylurus | 16.516667 | -15.816667 | M3 |
| http://n2t.net/ark:/65665/3b148bad7-3b1f-4852-bedd-a37349b9c7d1 | Chiroptera | Molossidae | Mops condylurus | 16.516667 | -15.816667 | M3 |
| http://n2t.net/ark:/65665/3b0428124-842b-4bdf-bc78-d5c2d749d844 | Chiroptera | Molossidae | Mops condylurus | 16.516667 | -15.816667 | M3 |
| http://n2t.net/ark:/65665/3db2fa597-a2d5-4179-b3b1-c623f59ee46d | Chiroptera | Molossidae | Mops condylurus | 16.516667 | -15.816667 | M3 |
| http://n2t.net/ark:/65665/3b99db1d2-d23d-4cdb-9963-8828ff696876 | Chiroptera | Molossidae | Mops condylurus | 16.516667 | -15.816667 | M3 |
| http://n2t.net/ark:/65665/37a8e8dc9-39a6-4adf-93e0-1193e38f3523 | Chiroptera | Molossidae | Mops condylurus | 16.516667 | -15.816667 | M3 |
| http://n2t.net/ark:/65665/3c69158c7-ff9b-44f8-ad8f-7a45e0b758a1 | Chiroptera | Molossidae | Mops condylurus | 16.516667 | -15.816667 | M3 |
| http://n2t.net/ark:/65665/3b95546a4-8d12-4696-b37c-67a17bf58df7 | Chiroptera | Molossidae | Mops condylurus | 16.516667 | -15.816667 | M3 |
| http://n2t.net/ark:/65665/3453a4601-f5b0-46a5-96e9-f9e48fe77128 | Chiroptera | Molossidae | Mops condylurus | 16.516667 | -15.816667 | M3 |
| http://n2t.net/ark:/65665/30d0352b8-e26b-4f7c-86cc-0f3870163152 | Chiroptera | Molossidae | Mops condylurus | 16.516667 | -15.816667 | M3 |
| http://n2t.net/ark:/65665/325bcbbf4-ae5f-475d-b54b-03ece97d9f99 | Chiroptera | Molossidae | Mops condylurus | 16.516667 | -15.816667 | M3 |
| http://n2t.net/ark:/65665/3a575e3d8-c87c-429e-8217-1b56d296b878 | Chiroptera | Molossidae | Mops condylurus | 16.516667 | -15.816667 | M3 |
| http://n2t.net/ark:/65665/327840584-0dcf-4726-9349-2a9e1548be79 | Chiroptera | Molossidae | Mops condylurus | 16.516667 | -15.816667 | M3 |
| http://n2t.net/ark:/65665/32b5a2a7a-7318-46a7-bcc8-31ac3a59a5ee | Chiroptera | Molossidae | Mops condylurus | 16.516667 | -15.816667 | M3 |
| http://n2t.net/ark:/65665/355f36fe3-33c7-4662-917e-87276446f681 | Chiroptera | Molossidae | Mops condylurus | 16.516667 | -15.816667 | M3 |
| http://n2t.net/ark:/65665/37783b31d-2f3a-4239-8856-a956dcc29e2d | Chiroptera | Molossidae | Mops condylurus | 16.516667 | -15.816667 | M3 |
| http://n2t.net/ark:/65665/37f2b318e-0cd3-4427-83a3-02f9848d6c4b | Chiroptera | Molossidae | Mops condylurus | 16.516667 | -15.816667 | M3 |
| http://n2t.net/ark:/65665/3e9f8710d-b2f5-4c59-b775-b9f97d67ef57 | Chiroptera | Molossidae | Mops condylurus | 16.516667 | -15.816667 | M3 |
| http://n2t.net/ark:/65665/3f587f09b-fb99-424b-8861-29262bc0a31c | Chiroptera | Molossidae | Mops condylurus | 16.516667 | -15.816667 | M3 |
| http://n2t.net/ark:/65665/303242a6b-ff8a-495a-b61d-c0fc83de308d | Chiroptera | Molossidae | Mops condylurus | 16.516667 | -15.816667 | M3 |
| http://n2t.net/ark:/65665/3dd592b03-ca22-42b3-9c26-92c8cd3b1fcc | Chiroptera | Molossidae | Mops condylurus | 16.516667 | -15.816667 | M3 |
| http://n2t.net/ark:/65665/39e0a19ea-7923-429a-89d4-b5c27c7ae4e7 | Chiroptera | Molossidae | Mops condylurus | 16.516667 | -15.816667 | M3 |
| http://n2t.net/ark:/65665/324d91696-b06b-4130-9513-a3aca69e22a4 | Chiroptera | Molossidae | Mops condylurus | 16.516667 | -15.816667 | M3 |
| http://n2t.net/ark:/65665/324b36f02-5dee-4a76-b406-d87305c63956 | Chiroptera | Molossidae | Mops condylurus | 16.516667 | -15.816667 | M3 |
| http://n2t.net/ark:/65665/332124411-5d8a-4ce2-b207-0c8516b419a0 | Chiroptera | Molossidae | Mops condylurus | 16.516667 | -15.816667 | M3 |
| http://n2t.net/ark:/65665/3a4900f63-acec-496a-af61-7993cf77c0d4 | Chiroptera | Molossidae | Mops condylurus | 16.516667 | -15.816667 | M3 |
| http://n2t.net/ark:/65665/377d81a06-79e3-4208-9371-d13bbff6bd86 | Chiroptera | Molossidae | Mops condylurus | 16.516667 | -15.816667 | M3 |
| http://n2t.net/ark:/65665/307f88b00-87fd-4e07-995d-f1d3d3a553a6 | Chiroptera | Molossidae | Mops condylurus | 16.516667 | -15.816667 | M3 |
| http://n2t.net/ark:/65665/3d73407fd-5061-4847-a7e5-2c41e7451832 | Chiroptera | Molossidae | Mops condylurus | 16.516667 | -15.816667 | M3 |
| http://n2t.net/ark:/65665/3afee4223-364f-4c1f-b7b1-1e44ddec2977 | Chiroptera | Molossidae | Mops condylurus | 16.516667 | -15.816667 | M3 |
| http://n2t.net/ark:/65665/330448e4d-61c4-46b5-a3ca-351b226cfdce | Chiroptera | Molossidae | Mops condylurus | 16.516667 | -15.816667 | M3 |
| http://n2t.net/ark:/65665/30d8a9222-dfd1-48a0-a1af-bee4ebb80abb | Chiroptera | Molossidae | Mops condylurus | 16.516667 | -15.816667 | M3 |
| http://n2t.net/ark:/65665/3419e9fa9-13d6-4f61-8fe9-79dba4ed9955 | Chiroptera | Molossidae | Mops condylurus | 16.516667 | -15.816667 | M3 |
| http://n2t.net/ark:/65665/3a20d7f55-79d1-448f-8fba-37b67dd719f2 | Chiroptera | Molossidae | Mops condylurus | 16.516667 | -15.816667 | M3 |
| http://n2t.net/ark:/65665/3df33f44c-096f-4137-b9a4-91e71283648f | Chiroptera | Molossidae | Mops condylurus | 16.516667 | -15.816667 | M3 |
| http://n2t.net/ark:/65665/323b084d6-3fbb-4492-9365-a7b5f5baf0e5 | Chiroptera | Molossidae | Mops condylurus | 16.516667 | -15.816667 | M3 |
| http://n2t.net/ark:/65665/3ebc1e0a6-0cb3-4150-bb4a-068accc073b5 | Chiroptera | Molossidae | Mops condylurus | 16.516667 | -15.816667 | M3 |
| http://n2t.net/ark:/65665/3cd34c606-d706-4228-9ad4-0f3a72305c4b | Chiroptera | Molossidae | Mops condylurus | 16.516667 | -15.816667 | M3 |
| http://n2t.net/ark:/65665/3d234cc8d-675d-44cd-9720-f54c442e95c7 | Chiroptera | Molossidae | Mops condylurus | 16.516667 | -15.816667 | M3 |
| http://n2t.net/ark:/65665/310c41c11-561b-4594-a70e-658484fd4d0a | Chiroptera | Molossidae | Mops condylurus | 16.516667 | -15.816667 | M3 |
| http://n2t.net/ark:/65665/37245db85-56d3-482a-bcbc-759a8bf6bd2f | Chiroptera | Molossidae | Mops condylurus | 16.516667 | -15.816667 | M3 |
| http://n2t.net/ark:/65665/3d00f115c-aefc-4650-b487-11ed5eeec784 | Chiroptera | Molossidae | Mops condylurus | 16.516667 | -15.816667 | M3 |
| http://n2t.net/ark:/65665/3a79fb7e2-3d18-476f-80d8-ee305b764b92 | Chiroptera | Molossidae | Mops condylurus | 16.516667 | -15.816667 | M3 |
| http://n2t.net/ark:/65665/3e2534253-aa91-4197-a251-0a24dad9f2cb | Chiroptera | Molossidae | Mops condylurus | 16.516667 | -15.816667 | M3 |
| http://n2t.net/ark:/65665/33895e297-ba35-47cd-95a8-a7b06776051d | Chiroptera | Molossidae | Mops condylurus | 16.516667 | -15.816667 | M3 |
| http://n2t.net/ark:/65665/362028262-16be-48fc-90b0-a1c236e57e7a | Chiroptera | Molossidae | Mops condylurus | 16.516667 | -15.816667 | M3 |
| http://n2t.net/ark:/65665/3d86832ef-feff-449d-a511-7867b988fcf4 | Chiroptera | Molossidae | Mops condylurus | 16.516667 | -15.816667 | M3 |
| http://n2t.net/ark:/65665/39d60b5f8-7ee6-4b52-a749-dbd85911769f | Chiroptera | Molossidae | Mops condylurus | 16.516667 | -15.816667 | M3 |
| http://n2t.net/ark:/65665/373eb69c0-5b5c-4b1f-9182-e29cf68605b5 | Chiroptera | Molossidae | Mops condylurus | 16.516667 | -15.816667 | M3 |
| http://n2t.net/ark:/65665/31064697f-0f80-4459-b900-52049579a050 | Chiroptera | Molossidae | Mops condylurus | 16.516667 | -15.816667 | M3 |
| http://n2t.net/ark:/65665/37068c608-4947-4842-b29a-1bd2259e3238 | Chiroptera | Molossidae | Mops condylurus | 16.516667 | -15.816667 | M3 |
| http://n2t.net/ark:/65665/37c099796-d392-4749-bcef-e27c68deb931 | Chiroptera | Molossidae | Mops condylurus | 16.516667 | -15.816667 | M3 |
| http://n2t.net/ark:/65665/34563395d-1bf0-442f-b91c-5d6f7bf9fbc8 | Chiroptera | Molossidae | Mops condylurus | 16.516667 | -15.816667 | M3 |
| http://n2t.net/ark:/65665/3e8a60982-7063-4c69-9e7b-b571cceaa1ba | Chiroptera | Molossidae | Mops condylurus | 16.516667 | -15.816667 | M3 |
| http://n2t.net/ark:/65665/3538784ac-66b5-411c-87a3-84aecf8cd832 | Chiroptera | Molossidae | Mops condylurus | 16.516667 | -15.816667 | M3 |
| http://n2t.net/ark:/65665/3d0d2ba36-adc5-4945-ae3a-0ae984c55483 | Chiroptera | Molossidae | Mops condylurus | 16.516667 | -15.816667 | M3 |
| http://n2t.net/ark:/65665/3e7816f5c-9f4b-4400-b260-abebe6e0bc7f | Chiroptera | Molossidae | Mops condylurus | 16.516667 | -15.816667 | M3 |
| http://n2t.net/ark:/65665/3053b5bd0-6bc1-4248-94ed-c60d51d7c80d | Chiroptera | Molossidae | Mops condylurus | 16.516667 | -15.816667 | M3 |
| http://n2t.net/ark:/65665/3b445d4f5-147b-42f2-b23a-a7fb7b075933 | Chiroptera | Molossidae | Mops condylurus | 16.516667 | -15.816667 | M3 |
| http://n2t.net/ark:/65665/378c631d1-f1a7-4110-8c07-32d9ea8221ae | Chiroptera | Molossidae | Mops condylurus | 16.516667 | -15.816667 | M3 |
| http://n2t.net/ark:/65665/3667943c7-8443-4b39-b816-22039c830f37 | Chiroptera | Molossidae | Mops condylurus | 16.516667 | -15.816667 | M3 |
| http://n2t.net/ark:/65665/3ce2f7c22-5e03-41bd-aaab-ba2e8361cc52 | Chiroptera | Molossidae | Mops condylurus | 16.516667 | -15.816667 | M3 |
| http://n2t.net/ark:/65665/3c62f7e70-40b1-4f2e-b1e2-1969b9d5634f | Chiroptera | Molossidae | Mops condylurus | 16.516667 | -15.816667 | M3 |
| http://n2t.net/ark:/65665/304561042-e7dc-4497-8740-1419c434c846 | Chiroptera | Molossidae | Mops condylurus | 16.516667 | -15.816667 | M3 |
| http://n2t.net/ark:/65665/3b1204ae5-81c5-4774-8837-7c8efa08ad9b | Chiroptera | Molossidae | Mops condylurus | 16.516667 | -15.816667 | M3 |
| http://n2t.net/ark:/65665/30f71609e-0163-4b03-8270-508c68a9821c | Chiroptera | Molossidae | Mops condylurus | 16.516667 | -15.816667 | M3 |
| http://n2t.net/ark:/65665/3c2263691-b296-47db-803a-a6744d97a12d | Chiroptera | Molossidae | Mops condylurus | 16.516667 | -15.816667 | M3 |
| http://n2t.net/ark:/65665/3050176d8-0eb5-4795-8ccf-82a0f4112b57 | Chiroptera | Molossidae | Mops condylurus | 16.516667 | -15.816667 | M3 |
| http://n2t.net/ark:/65665/32d189460-1830-474e-83dd-dc798d7a0489 | Chiroptera | Molossidae | Mops condylurus | 16.516667 | -15.816667 | M3 |
| http://n2t.net/ark:/65665/342fcff14-c227-4330-916d-c609776d3809 | Chiroptera | Molossidae | Mops condylurus | 16.516667 | -15.816667 | M3 |
|  | Chiroptera | Molossidae | Mops condylurus | 16.512550 | -15.804920 | M3 |
| http://n2t.net/ark:/65665/344a0a994-21b1-4c57-be5f-3a68674108e9 | Chiroptera | Nycteridae | Nycteris hispida | 16.550000 | -15.766667 | M3 |
| http://n2t.net/ark:/65665/3fda9a6c3-7563-4e6e-ad44-d39d4f0b98e3 | Chiroptera | Nycteridae | Nycteris hispida | 16.550000 | -15.766667 | M3 |
| http://n2t.net/ark:/65665/32be277a0-5774-4f4b-b04c-ea0833dd5410 | Chiroptera | Nycteridae | Nycteris hispida | 16.550000 | -15.766667 | M3 |
| http://n2t.net/ark:/65665/3cc96c3d4-837c-45b2-95cc-8ac69666b645 | Chiroptera | Nycteridae | Nycteris hispida | 16.550000 | -15.766667 | M3 |
| http://n2t.net/ark:/65665/3bf41ee12-1a62-441e-be22-60585e457b3d | Chiroptera | Nycteridae | Nycteris hispida | 16.550000 | -15.766667 | M3 |
| http://n2t.net/ark:/65665/3d25fbca1-f179-4951-9c57-baf39c4678d3 | Chiroptera | Nycteridae | Nycteris hispida | 16.550000 | -15.766667 | M3 |
| http://n2t.net/ark:/65665/349ca3301-b76f-4e1e-ba05-89794696a836 | Chiroptera | Nycteridae | Nycteris hispida | 16.550000 | -15.766667 | M3 |
| http://n2t.net/ark:/65665/3a877eb4d-8d52-4cee-9b30-62982074e207 | Chiroptera | Nycteridae | Nycteris hispida | 16.550000 | -15.766667 | M3 |
| http://n2t.net/ark:/65665/331bc9107-08e9-462c-856b-51eda35320fc | Chiroptera | Nycteridae | Nycteris hispida | 16.550000 | -15.766667 | M3 |
| http://n2t.net/ark:/65665/30c1abcfc-3e79-4740-ac15-7f1ac46c8920 | Chiroptera | Nycteridae | Nycteris hispida | 16.550000 | -15.766667 | M3 |
| http://n2t.net/ark:/65665/3d9fa06c9-ccb5-416e-8a24-f755c76dac43 | Chiroptera | Nycteridae | Nycteris hispida | 16.550000 | -15.766667 | M3 |
| http://n2t.net/ark:/65665/329b216cf-e86a-4cfd-b7dd-eda889c71e0b | Chiroptera | Nycteridae | Nycteris hispida | 16.550000 | -15.766667 | M3 |
| http://n2t.net/ark:/65665/345a2a648-5719-4c67-a97a-6b07f2d6357a | Chiroptera | Nycteridae | Nycteris hispida | 16.550000 | -15.766667 | M3 |
| http://n2t.net/ark:/65665/32a255e31-86ac-4466-ac47-e3dd579c7199 | Chiroptera | Nycteridae | Nycteris hispida | 16.550000 | -15.766667 | M3 |
| http://n2t.net/ark:/65665/345482a43-176c-4a64-98e3-881285e708c5 | Chiroptera | Nycteridae | Nycteris hispida | 16.550000 | -15.766667 | M3 |
| http://n2t.net/ark:/65665/3dc08b486-e615-4825-bb8a-beaef405bfef | Chiroptera | Nycteridae | Nycteris hispida | 16.550000 | -15.766667 | M3 |
| http://n2t.net/ark:/65665/366998f44-9baf-4d6c-9463-f8abdea33fe9 | Chiroptera | Nycteridae | Nycteris hispida | 16.550000 | -15.766667 | M3 |
| http://n2t.net/ark:/65665/34ed844e3-0f0d-45a0-a9c3-86e35af9d038 | Chiroptera | Nycteridae | Nycteris hispida | 16.550000 | -15.766667 | M3 |
| http://n2t.net/ark:/65665/325559d15-7145-4c9d-ac66-6e2b59e3bd17 | Chiroptera | Nycteridae | Nycteris hispida | 16.550000 | -15.766667 | M3 |
| http://n2t.net/ark:/65665/3b5936194-078f-439e-8cc4-a064efffaddb | Chiroptera | Nycteridae | Nycteris hispida | 16.550000 | -15.766667 | M3 |
| http://n2t.net/ark:/65665/3c1842a2c-e990-430f-8f4b-0509db2fd36f | Chiroptera | Nycteridae | Nycteris hispida | 16.550000 | -15.766667 | M3 |
| http://n2t.net/ark:/65665/350091eec-a6e6-40e4-9ff0-e9fe5b303adc | Chiroptera | Nycteridae | Nycteris hispida | 16.550000 | -15.766667 | M3 |
| http://n2t.net/ark:/65665/3eb076f61-9423-4f35-9988-665096e5e458 | Chiroptera | Nycteridae | Nycteris hispida | 16.550000 | -15.766667 | M3 |
| http://n2t.net/ark:/65665/32fa08f01-5305-476e-8233-4e57fa0a2dba | Chiroptera | Nycteridae | Nycteris hispida | 16.550000 | -15.766667 | M3 |
| http://n2t.net/ark:/65665/3e9a36cf5-bffb-4716-883c-0a244b5ea240 | Chiroptera | Nycteridae | Nycteris hispida | 16.550000 | -15.766667 | M3 |
| http://n2t.net/ark:/65665/3e8111840-a261-4700-a9db-5dded63575e5 | Chiroptera | Nycteridae | Nycteris hispida | 16.550000 | -15.766667 | M3 |
| http://n2t.net/ark:/65665/337825d29-3c22-4634-bd0e-ed60d8a9b48e | Chiroptera | Nycteridae | Nycteris hispida | 16.550000 | -15.766667 | M3 |
| http://n2t.net/ark:/65665/39ad07b12-ee20-49df-970b-24d5368ace54 | Chiroptera | Nycteridae | Nycteris hispida | 16.550000 | -15.766667 | M3 |
| http://n2t.net/ark:/65665/349456a82-cbf2-418c-955c-8cf3bb900c74 | Chiroptera | Nycteridae | Nycteris hispida | 16.550000 | -15.766667 | M3 |
| http://n2t.net/ark:/65665/37603b260-36f7-42c4-81ed-161bb5b21cde | Chiroptera | Nycteridae | Nycteris hispida | 16.550000 | -15.766667 | M3 |
| http://n2t.net/ark:/65665/3392525ee-4261-4951-a085-1059315d9378 | Chiroptera | Nycteridae | Nycteris hispida | 16.550000 | -15.766667 | M3 |
| http://n2t.net/ark:/65665/365af8abf-1084-44a8-bd72-258f89b1ca4d | Chiroptera | Nycteridae | Nycteris hispida | 16.550000 | -15.766667 | M3 |
| http://n2t.net/ark:/65665/3cc472719-974a-46ce-b15c-2c1b385c78ed | Chiroptera | Nycteridae | Nycteris hispida | 16.550000 | -15.766667 | M3 |
| http://n2t.net/ark:/65665/39d120e0f-c7ab-4d2a-8526-4a80e02c731c | Chiroptera | Nycteridae | Nycteris hispida | 16.550000 | -15.766667 | M3 |
| http://n2t.net/ark:/65665/3792cd096-07c2-433d-b246-39dd7c22de51 | Chiroptera | Nycteridae | Nycteris hispida | 16.550000 | -15.766667 | M3 |
| http://n2t.net/ark:/65665/3d31192b3-4a20-46f5-a2aa-d61df45350d5 | Chiroptera | Nycteridae | Nycteris hispida | 16.550000 | -15.766667 | M3 |
| http://n2t.net/ark:/65665/3438ced2e-f9ec-4ec0-b008-d46c2b90f265 | Chiroptera | Nycteridae | Nycteris hispida | 16.550000 | -15.766667 | M3 |
| http://n2t.net/ark:/65665/3ccf96715-aaee-4f1e-864a-51cf7f577eae | Chiroptera | Nycteridae | Nycteris hispida | 16.550000 | -15.766667 | M3 |
| http://n2t.net/ark:/65665/372f59f3b-dd0c-4a9c-8e30-83d173897ba3 | Chiroptera | Nycteridae | Nycteris hispida | 16.550000 | -15.766667 | M3 |
| http://n2t.net/ark:/65665/3746cddce-7f97-4c77-a0ec-f0d9e645922f | Chiroptera | Nycteridae | Nycteris hispida | 16.550000 | -15.766667 | M3 |
| http://n2t.net/ark:/65665/30106a3ee-6792-4d37-b38a-b56cb2077bca | Chiroptera | Nycteridae | Nycteris hispida | 16.550000 | -15.766667 | M3 |
| http://n2t.net/ark:/65665/3b9c87fe1-54b0-46c7-a0cc-23418da189a3 | Chiroptera | Nycteridae | Nycteris hispida | 16.550000 | -15.766667 | M3 |
| http://n2t.net/ark:/65665/32434ff70-2ae9-4299-8c43-27d3e15d9464 | Chiroptera | Nycteridae | Nycteris hispida | 16.550000 | -15.766667 | M3 |
| http://n2t.net/ark:/65665/393e7d9d1-ad3e-4de0-a2cd-00af09fc8754 | Chiroptera | Nycteridae | Nycteris hispida | 16.550000 | -15.766667 | M3 |
| http://n2t.net/ark:/65665/3d63ca78f-1928-440b-ab7c-df2610d8e48d | Chiroptera | Nycteridae | Nycteris hispida | 16.550000 | -15.766667 | M3 |
| http://n2t.net/ark:/65665/3e7493246-8591-4bb7-9bd7-f939d80f03bd | Chiroptera | Nycteridae | Nycteris hispida | 16.550000 | -15.766667 | M3 |
| http://n2t.net/ark:/65665/3cc81a07a-a544-474d-8912-518ca27bfbba | Chiroptera | Nycteridae | Nycteris hispida | 16.550000 | -15.766667 | M3 |
| http://n2t.net/ark:/65665/33f2d085e-dc47-411e-8afc-2013673ecf7d | Chiroptera | Nycteridae | Nycteris hispida | 16.550000 | -15.766667 | M3 |
| http://n2t.net/ark:/65665/322a749c4-7478-4c84-8bcf-8f0049aa2243 | Chiroptera | Nycteridae | Nycteris hispida | 16.550000 | -15.766667 | M3 |
| http://n2t.net/ark:/65665/3bb4cdcee-6142-4ca8-969b-31da08b6a943 | Chiroptera | Nycteridae | Nycteris hispida | 16.550000 | -15.766667 | M3 |
| http://n2t.net/ark:/65665/34f3f07cf-9fa6-4108-b34f-df9d5974895a | Chiroptera | Nycteridae | Nycteris hispida | 16.550000 | -15.766667 | M3 |
| http://n2t.net/ark:/65665/3cdd313ed-bdcd-4e92-b719-d3580727b783 | Chiroptera | Nycteridae | Nycteris hispida | 16.550000 | -15.766667 | M3 |
| http://n2t.net/ark:/65665/349c89bc7-669b-4455-a05a-9273cfd5881c | Chiroptera | Nycteridae | Nycteris hispida | 16.550000 | -15.766667 | M3 |
| http://n2t.net/ark:/65665/3349339bf-0ae8-4b6b-80ac-0dbcba92958d | Chiroptera | Nycteridae | Nycteris hispida | 16.550000 | -15.766667 | M3 |
| http://n2t.net/ark:/65665/344cccd13-3e8d-4a0b-b615-e200f56bb5f0 | Chiroptera | Nycteridae | Nycteris hispida | 16.550000 | -15.766667 | M3 |
| http://n2t.net/ark:/65665/379a58ee4-565f-4df0-a3ce-320032abffe7 | Chiroptera | Nycteridae | Nycteris hispida | 16.550000 | -15.766667 | M3 |
| http://n2t.net/ark:/65665/34a53788e-5b9a-4869-b375-3afe4931aed0 | Chiroptera | Nycteridae | Nycteris hispida | 16.550000 | -15.766667 | M3 |
| http://n2t.net/ark:/65665/31eb6291a-697f-47a5-b903-1a0bc7900af2 | Chiroptera | Nycteridae | Nycteris hispida | 16.550000 | -15.766667 | M3 |
| http://n2t.net/ark:/65665/3a07aa54f-1e8f-4e99-abba-6ea58b5a2f03 | Chiroptera | Nycteridae | Nycteris hispida | 16.550000 | -15.766667 | M3 |
| http://n2t.net/ark:/65665/3efe52350-8fb0-449d-bd04-74a6fccb3f79 | Chiroptera | Nycteridae | Nycteris hispida | 16.550000 | -15.766667 | M3 |
| http://n2t.net/ark:/65665/35e1f9769-125c-4d9f-bcc7-1586c53a1cab | Chiroptera | Nycteridae | Nycteris hispida | 16.550000 | -15.766667 | M3 |
| http://n2t.net/ark:/65665/3a16e79e6-f0b1-48dc-b9bf-3354a1bfd502 | Chiroptera | Nycteridae | Nycteris hispida | 16.550000 | -15.766667 | M3 |
| http://n2t.net/ark:/65665/30edcf2d9-7304-4851-b6b8-d27866354f76 | Chiroptera | Nycteridae | Nycteris hispida | 16.550000 | -15.766667 | M3 |
| http://n2t.net/ark:/65665/383b92321-d955-494d-85b0-358ba759a7b9 | Chiroptera | Nycteridae | Nycteris hispida | 16.550000 | -15.766667 | M3 |
| http://n2t.net/ark:/65665/3d6e960ed-442c-422a-9ddb-2e791f6e78c2 | Chiroptera | Nycteridae | Nycteris hispida | 16.550000 | -15.766667 | M3 |
| http://n2t.net/ark:/65665/37ef80313-0329-46f4-9415-0b6edb185308 | Chiroptera | Nycteridae | Nycteris hispida | 16.550000 | -15.766667 | M3 |
| http://n2t.net/ark:/65665/3df42aa7f-b836-42fb-b52b-2188fa53d651 | Chiroptera | Nycteridae | Nycteris hispida | 16.550000 | -15.766667 | M3 |
| http://n2t.net/ark:/65665/3ad9284d9-c5d8-4c82-a17d-b544f443fca7 | Chiroptera | Nycteridae | Nycteris hispida | 16.550000 | -15.766667 | M3 |
| http://n2t.net/ark:/65665/31386f378-eec3-439b-8781-5ec608c80a14 | Chiroptera | Nycteridae | Nycteris hispida | 16.550000 | -15.766667 | M3 |
| http://n2t.net/ark:/65665/3472248da-8ec3-4aca-ab76-6f9ee9a2843e | Chiroptera | Nycteridae | Nycteris hispida | 16.550000 | -15.766667 | M3 |
| http://n2t.net/ark:/65665/3fab30259-8502-4a35-94d4-f0222006c5ea | Chiroptera | Nycteridae | Nycteris hispida | 16.550000 | -15.766667 | M3 |
| http://n2t.net/ark:/65665/30ff74f33-e7b7-4f53-9c1b-d30db387898d | Chiroptera | Nycteridae | Nycteris hispida | 16.550000 | -15.766667 | M3 |
| http://n2t.net/ark:/65665/3e267eb8b-deb1-4b6f-b3b3-28c7b0e7e0b3 | Chiroptera | Nycteridae | Nycteris hispida | 16.550000 | -15.766667 | M3 |
| http://n2t.net/ark:/65665/3fd9f24dd-23e9-4728-890d-da2ed131fd31 | Chiroptera | Nycteridae | Nycteris hispida | 16.550000 | -15.766667 | M3 |
| http://n2t.net/ark:/65665/31862d1da-e710-487c-8f24-c32b897609eb | Chiroptera | Nycteridae | Nycteris hispida | 16.550000 | -15.766667 | M3 |
| http://n2t.net/ark:/65665/3abc9c0ae-20c3-4ec7-80b8-423fce6cf19a | Chiroptera | Nycteridae | Nycteris hispida | 16.550000 | -15.766667 | M3 |
| http://n2t.net/ark:/65665/3e86fc044-2e05-445a-adc1-440e8f8cf572 | Chiroptera | Nycteridae | Nycteris hispida | 16.550000 | -15.766667 | M3 |
| http://n2t.net/ark:/65665/32e86f444-b240-463a-90c1-e87b4dd1f664 | Chiroptera | Nycteridae | Nycteris hispida | 16.550000 | -15.766667 | M3 |
| http://n2t.net/ark:/65665/37e23e8ae-3dcf-4f07-872a-c14a8a776fad | Chiroptera | Nycteridae | Nycteris hispida | 16.550000 | -15.766667 | M3 |
| http://n2t.net/ark:/65665/3b121ac6d-dd5b-4a7f-af1d-7d09a59ed1b0 | Chiroptera | Nycteridae | Nycteris hispida | 16.550000 | -15.766667 | M3 |
| http://n2t.net/ark:/65665/357e025ad-b16c-40c1-bad4-be8fdfa7d457 | Chiroptera | Nycteridae | Nycteris hispida | 16.550000 | -15.766667 | M3 |
| http://n2t.net/ark:/65665/301bc8cf2-9f71-4fd2-b9e9-0f8dd27d1d49 | Chiroptera | Nycteridae | Nycteris hispida | 16.550000 | -15.766667 | M3 |
| http://n2t.net/ark:/65665/34909dc93-6fe7-4705-840f-47c2bb7f6316 | Chiroptera | Nycteridae | Nycteris hispida | 16.550000 | -15.766667 | M3 |
| http://n2t.net/ark:/65665/39a3114f0-9ebc-4be9-94bf-f885b359e0d5 | Chiroptera | Nycteridae | Nycteris hispida | 16.550000 | -15.766667 | M3 |
| http://n2t.net/ark:/65665/3264d194f-3ad9-4575-8ed7-7ec29916b501 | Chiroptera | Nycteridae | Nycteris hispida | 16.550000 | -15.766667 | M3 |
| http://n2t.net/ark:/65665/3cb9e1250-9861-4f1b-981d-780a13960c0a | Chiroptera | Nycteridae | Nycteris hispida | 16.550000 | -15.766667 | M3 |
| http://n2t.net/ark:/65665/3ba4fffe5-c3ba-4aeb-b335-3145d5d3edb6 | Chiroptera | Nycteridae | Nycteris hispida | 16.550000 | -15.766667 | M3 |
| http://n2t.net/ark:/65665/3b20e8e3c-d5a6-4524-a066-ec6a570e3eb6 | Chiroptera | Nycteridae | Nycteris hispida | 16.550000 | -15.766667 | M3 |
| http://n2t.net/ark:/65665/30fd18d3b-9f91-4238-b4e5-9f12bdc70d60 | Chiroptera | Nycteridae | Nycteris hispida | 16.550000 | -15.766667 | M3 |
| http://n2t.net/ark:/65665/3950401e7-628c-4f47-bd5a-91668331eae0 | Chiroptera | Nycteridae | Nycteris hispida | 16.550000 | -15.766667 | M3 |
| http://n2t.net/ark:/65665/3aef31a7b-3431-4a2a-a8f5-118ec063de2c | Chiroptera | Nycteridae | Nycteris hispida | 16.550000 | -15.766667 | M3 |
| http://n2t.net/ark:/65665/3aa572c59-1578-464d-a6b4-1a4e3374fccb | Chiroptera | Nycteridae | Nycteris hispida | 16.550000 | -15.766667 | M3 |
| http://n2t.net/ark:/65665/320a39bbd-b4ab-4d47-a96b-08f64d69f6d9 | Chiroptera | Nycteridae | Nycteris hispida | 16.550000 | -15.766667 | M3 |
| http://n2t.net/ark:/65665/3a543f255-d7cd-4cf1-8248-9e08c310e444 | Chiroptera | Nycteridae | Nycteris hispida | 16.550000 | -15.766667 | M3 |
| http://n2t.net/ark:/65665/38134cf66-2200-470f-90be-b0a5ae4cee76 | Chiroptera | Nycteridae | Nycteris hispida | 16.550000 | -15.766667 | M3 |
| http://n2t.net/ark:/65665/35f43bdd7-62ff-4b97-b1c4-6b03e43ae435 | Chiroptera | Nycteridae | Nycteris hispida | 16.550000 | -15.766667 | M3 |
| http://n2t.net/ark:/65665/317f30b99-7fb2-4c07-a539-00ef06af6168 | Chiroptera | Nycteridae | Nycteris hispida | 16.550000 | -15.766667 | M3 |
| http://n2t.net/ark:/65665/362ad8fe0-3152-4457-9444-4edb4b019b02 | Chiroptera | Nycteridae | Nycteris hispida | 16.550000 | -15.766667 | M3 |
| http://n2t.net/ark:/65665/36091e7f9-8363-405e-a61c-700a4b38be66 | Chiroptera | Nycteridae | Nycteris hispida | 16.550000 | -15.766667 | M3 |
| http://n2t.net/ark:/65665/3615af026-3020-4261-a636-af98adec346a | Chiroptera | Nycteridae | Nycteris hispida | 16.550000 | -15.766667 | M3 |
| http://n2t.net/ark:/65665/33e3333bf-ec6d-439c-b535-e30834c1c789 | Chiroptera | Nycteridae | Nycteris hispida | 16.550000 | -15.766667 | M3 |
| http://n2t.net/ark:/65665/3dd9cf215-092e-4aee-98ba-0917797e0dd1 | Chiroptera | Nycteridae | Nycteris hispida | 16.550000 | -15.766667 | M3 |
| http://n2t.net/ark:/65665/31582bbf4-7dff-4677-968d-93e6aa2eee9a | Chiroptera | Nycteridae | Nycteris hispida | 16.550000 | -15.766667 | M3 |
| http://n2t.net/ark:/65665/3bf2d414b-7189-4c3f-bbbb-9d6826778460 | Chiroptera | Nycteridae | Nycteris hispida | 16.550000 | -15.766667 | M3 |
| http://n2t.net/ark:/65665/3e4cfdcd3-0566-4c4f-aba5-cb92431ca503 | Chiroptera | Nycteridae | Nycteris hispida | 16.550000 | -15.766667 | M3 |
| http://n2t.net/ark:/65665/3de7fc9ea-fdc0-4474-b3ef-57093deb47c7 | Chiroptera | Nycteridae | Nycteris hispida | 16.550000 | -15.766667 | M3 |
| http://n2t.net/ark:/65665/33df13bbb-6d95-4809-a4f8-14334f35c1f1 | Chiroptera | Nycteridae | Nycteris hispida | 16.550000 | -15.766667 | M3 |
| http://n2t.net/ark:/65665/3bf545017-e5c9-4743-86b0-621d2a9e2974 | Chiroptera | Nycteridae | Nycteris hispida | 16.550000 | -15.766667 | M3 |
| http://n2t.net/ark:/65665/3ac673bc0-39fe-4f1f-8740-eb4c3b6a81f6 | Chiroptera | Nycteridae | Nycteris hispida | 16.550000 | -15.766667 | M3 |
| http://n2t.net/ark:/65665/33f78bdc5-5015-439e-8609-cca9a19b7052 | Chiroptera | Nycteridae | Nycteris hispida | 16.550000 | -15.766667 | M3 |
| http://n2t.net/ark:/65665/39c9b49be-1f62-4b3c-9c89-a67670dfb01e | Chiroptera | Nycteridae | Nycteris hispida | 16.550000 | -15.766667 | M3 |
| http://n2t.net/ark:/65665/34d17568d-7790-4591-ad2a-ddc6fc9284c6 | Chiroptera | Nycteridae | Nycteris hispida | 16.550000 | -15.766667 | M3 |
| http://n2t.net/ark:/65665/39b2843e2-2595-4de2-9935-84339a45bae1 | Chiroptera | Nycteridae | Nycteris hispida | 16.550000 | -15.766667 | M3 |
| http://n2t.net/ark:/65665/313f3cf85-a540-4591-bc78-e4f8f125fea4 | Chiroptera | Nycteridae | Nycteris hispida | 16.550000 | -15.766667 | M3 |
| http://n2t.net/ark:/65665/366e165a3-d7de-485b-b181-f380b7cde4ff | Chiroptera | Nycteridae | Nycteris hispida | 16.550000 | -15.766667 | M3 |
| http://n2t.net/ark:/65665/3db16f0f9-9db4-4bfa-b58e-313c08ae571a | Chiroptera | Nycteridae | Nycteris hispida | 16.550000 | -15.766667 | M3 |
| http://n2t.net/ark:/65665/33274b2ec-7a90-4c94-932d-bab3c177bff8 | Chiroptera | Nycteridae | Nycteris hispida | 16.550000 | -15.766667 | M3 |
| http://n2t.net/ark:/65665/3637428e9-31e2-4695-958f-2f5d86d68452 | Chiroptera | Nycteridae | Nycteris hispida | 16.550000 | -15.766667 | M3 |
| http://n2t.net/ark:/65665/3ab671b4e-eef2-462b-9cc2-8d8f4757f547 | Chiroptera | Nycteridae | Nycteris hispida | 16.550000 | -15.766667 | M3 |
| http://n2t.net/ark:/65665/381716515-6a25-4888-8ae9-5cd5c4957e76 | Chiroptera | Nycteridae | Nycteris hispida | 16.550000 | -15.766667 | M3 |
| http://n2t.net/ark:/65665/31cf830b2-c438-454d-ab75-b47f24ce5f06 | Chiroptera | Nycteridae | Nycteris hispida | 16.550000 | -15.766667 | M3 |
| http://n2t.net/ark:/65665/30566afd2-c463-4fbb-a125-58b4e228907f | Chiroptera | Nycteridae | Nycteris hispida | 16.550000 | -15.766667 | M3 |
| http://n2t.net/ark:/65665/3c6307eba-4227-4a8e-8e20-9ad5e6036fbd | Chiroptera | Nycteridae | Nycteris hispida | 16.550000 | -15.766667 | M3 |
| http://n2t.net/ark:/65665/349465fdf-4710-430d-baff-151136986f65 | Chiroptera | Nycteridae | Nycteris hispida | 16.550000 | -15.766667 | M3 |
| http://n2t.net/ark:/65665/3c6b0c7cc-4afc-4cd0-b04b-895ac4f48d27 | Chiroptera | Nycteridae | Nycteris hispida | 16.550000 | -15.766667 | M3 |
| http://n2t.net/ark:/65665/39ace3920-edd6-45ca-b694-8d62103049b4 | Chiroptera | Nycteridae | Nycteris hispida | 16.550000 | -15.766667 | M3 |
| http://n2t.net/ark:/65665/3b3488d7b-ee63-450f-bbe4-4e11b1859fcc | Chiroptera | Nycteridae | Nycteris hispida | 16.550000 | -15.766667 | M3 |
| http://n2t.net/ark:/65665/3b67c5900-125f-4d57-ac98-7c18174dbbbb | Chiroptera | Nycteridae | Nycteris hispida | 16.550000 | -15.766667 | M3 |
| http://n2t.net/ark:/65665/345c7e3c6-88b4-4f55-bd5a-cb057e4b075e | Chiroptera | Nycteridae | Nycteris hispida | 16.550000 | -15.766667 | M3 |
| http://n2t.net/ark:/65665/30bee6b03-b5ad-41ea-9c65-31b2628f5e1c | Chiroptera | Nycteridae | Nycteris hispida | 16.550000 | -15.766667 | M3 |
| http://n2t.net/ark:/65665/32aaace60-0fb2-4209-bf15-22bb08e07595 | Chiroptera | Nycteridae | Nycteris hispida | 16.550000 | -15.766667 | M3 |
| http://n2t.net/ark:/65665/372228a62-0216-45b9-ae8e-fd24a88dc01e | Chiroptera | Nycteridae | Nycteris hispida | 16.550000 | -15.766667 | M3 |
| http://n2t.net/ark:/65665/339e02280-c29a-4250-8383-07a4d9a024b2 | Chiroptera | Nycteridae | Nycteris hispida | 16.550000 | -15.766667 | M3 |
| http://n2t.net/ark:/65665/3213d294c-f07e-43f5-9977-3f3bc2e3bb14 | Chiroptera | Nycteridae | Nycteris hispida | 16.550000 | -15.766667 | M3 |
| http://n2t.net/ark:/65665/3562ab505-d922-492d-acc3-b9381fe97769 | Chiroptera | Nycteridae | Nycteris hispida | 16.550000 | -15.766667 | M3 |
| http://n2t.net/ark:/65665/3fb08fd55-6677-4746-9712-295e9d72d814 | Chiroptera | Nycteridae | Nycteris hispida | 16.550000 | -15.766667 | M3 |
| http://n2t.net/ark:/65665/379ce664f-b83d-46dd-b482-e18c6ca34977 | Chiroptera | Nycteridae | Nycteris hispida | 16.550000 | -15.766667 | M3 |
| http://n2t.net/ark:/65665/358ed306d-9190-4514-8cd1-48ae50c1b0e8 | Chiroptera | Nycteridae | Nycteris hispida | 16.550000 | -15.766667 | M3 |
| http://n2t.net/ark:/65665/395411996-560b-41ad-a1b8-f3089c26438c | Chiroptera | Nycteridae | Nycteris hispida | 16.550000 | -15.766667 | M3 |
| http://n2t.net/ark:/65665/3f14be2fd-97a3-416d-b078-fe751eb82e9b | Chiroptera | Nycteridae | Nycteris hispida | 16.550000 | -15.766667 | M3 |
| http://n2t.net/ark:/65665/30996346c-b20c-4a7e-b85b-b4eec7ac783e | Chiroptera | Nycteridae | Nycteris hispida | 16.550000 | -15.766667 | M3 |
| http://n2t.net/ark:/65665/3d56a2acb-47f0-4645-95ed-dd709c1bf711 | Chiroptera | Nycteridae | Nycteris hispida | 16.550000 | -15.766667 | M3 |
| http://n2t.net/ark:/65665/3d652e676-5bb3-42bc-9186-e505a6b58bd8 | Chiroptera | Nycteridae | Nycteris hispida | 16.550000 | -15.766667 | M3 |
| http://n2t.net/ark:/65665/3c6a6c163-c090-458f-8882-ed75b4faa6b5 | Chiroptera | Nycteridae | Nycteris hispida | 16.550000 | -15.766667 | M3 |
| http://n2t.net/ark:/65665/38f33c1aa-408c-43b6-a307-c123cd729975 | Chiroptera | Nycteridae | Nycteris hispida | 16.550000 | -15.766667 | M3 |
| http://n2t.net/ark:/65665/3df9b348a-74eb-4575-b899-90f16ae9c002 | Chiroptera | Nycteridae | Nycteris hispida | 16.550000 | -15.766667 | M3 |
| http://n2t.net/ark:/65665/38dd453c3-c792-4965-85e6-7bcf277068d7 | Chiroptera | Nycteridae | Nycteris hispida | 16.550000 | -15.766667 | M3 |
| http://n2t.net/ark:/65665/376e62b0e-8691-4622-9e2a-8049b699aeaf | Chiroptera | Nycteridae | Nycteris hispida | 16.550000 | -15.766667 | M3 |
| http://n2t.net/ark:/65665/31d045f80-95d1-41ac-9c06-ecc2797f812f | Chiroptera | Nycteridae | Nycteris hispida | 16.550000 | -15.766667 | M3 |
| http://n2t.net/ark:/65665/35291eef3-03de-4fa2-b443-6f7367940f11 | Chiroptera | Nycteridae | Nycteris hispida | 16.550000 | -15.766667 | M3 |
| http://n2t.net/ark:/65665/374189328-cea8-4716-8e5e-afde5a0be8a7 | Chiroptera | Nycteridae | Nycteris hispida | 16.550000 | -15.766667 | M3 |
| http://n2t.net/ark:/65665/3d9c7f6d6-19f9-4e1c-b9e5-0630af746edd | Chiroptera | Nycteridae | Nycteris hispida | 16.550000 | -15.766667 | M3 |
| http://n2t.net/ark:/65665/3d27f7396-b1c9-4f91-b8db-8b8de9eca321 | Chiroptera | Nycteridae | Nycteris hispida | 16.550000 | -15.766667 | M3 |
| http://n2t.net/ark:/65665/376e2c0c1-5c72-40c6-80ff-5b755ca121c9 | Chiroptera | Nycteridae | Nycteris hispida | 16.550000 | -15.766667 | M3 |
| http://n2t.net/ark:/65665/3f4ad909a-a9f6-431e-8804-ab0072b5b559 | Chiroptera | Nycteridae | Nycteris hispida | 16.550000 | -15.766667 | M3 |
| http://n2t.net/ark:/65665/34c045259-eaf6-4228-8119-017af0f39cdf | Chiroptera | Nycteridae | Nycteris hispida | 16.550000 | -15.766667 | M3 |
| http://n2t.net/ark:/65665/3aacca444-7efd-4c34-8b95-b29d80f521fd | Chiroptera | Nycteridae | Nycteris hispida | 16.550000 | -15.766667 | M3 |
| http://n2t.net/ark:/65665/312d0bfd8-64aa-4942-9ff0-0add5a3c3683 | Chiroptera | Nycteridae | Nycteris hispida | 16.550000 | -15.766667 | M3 |
| http://n2t.net/ark:/65665/32187eb17-745b-4ebc-a0fc-c76914fe7339 | Chiroptera | Nycteridae | Nycteris hispida | 16.550000 | -15.766667 | M3 |
| http://n2t.net/ark:/65665/33e83c1a3-781e-41bd-b3f6-878c6d4f73a8 | Chiroptera | Nycteridae | Nycteris hispida | 16.550000 | -15.766667 | M3 |
| http://n2t.net/ark:/65665/371883ae4-8d9b-46b8-8a74-235dacffe3ec | Chiroptera | Nycteridae | Nycteris hispida | 16.550000 | -15.766667 | M3 |
| http://n2t.net/ark:/65665/3bea68672-8916-4063-b7b6-772c6fea4262 | Chiroptera | Nycteridae | Nycteris hispida | 16.550000 | -15.766667 | M3 |
| http://n2t.net/ark:/65665/3a52d7b96-e13f-4f72-a71b-7323e6690573 | Chiroptera | Nycteridae | Nycteris hispida | 16.550000 | -15.766667 | M3 |
| http://n2t.net/ark:/65665/301ff0a04-e7be-49d5-bf46-04c3ba33c02a | Chiroptera | Nycteridae | Nycteris hispida | 16.550000 | -15.766667 | M3 |
| http://n2t.net/ark:/65665/30dbac7dc-6db6-49c8-ba30-845300068021 | Chiroptera | Nycteridae | Nycteris hispida | 16.550000 | -15.766667 | M3 |
| http://n2t.net/ark:/65665/3bbbefd65-9e62-480f-8f64-5f06530df0b8 | Chiroptera | Nycteridae | Nycteris hispida | 16.550000 | -15.766667 | M3 |
| http://n2t.net/ark:/65665/3ffcc358e-5f92-4148-a724-a3a8f6faf3af | Chiroptera | Nycteridae | Nycteris hispida | 16.550000 | -15.766667 | M3 |
| http://n2t.net/ark:/65665/358d599f9-eff8-468b-b3d9-1be209d38835 | Chiroptera | Nycteridae | Nycteris hispida | 16.550000 | -15.766667 | M3 |
| http://n2t.net/ark:/65665/3c05e24ee-7f3e-47cb-b6d6-dd5e122d828e | Chiroptera | Nycteridae | Nycteris hispida | 16.550000 | -15.766667 | M3 |
| http://n2t.net/ark:/65665/363ba64db-a2f4-40a2-81f3-21a0262580ce | Chiroptera | Nycteridae | Nycteris hispida | 16.550000 | -15.766667 | M3 |
| http://n2t.net/ark:/65665/37eb1485f-f299-4402-a9ff-106e5d5125e7 | Chiroptera | Nycteridae | Nycteris hispida | 16.550000 | -15.766667 | M3 |
| http://n2t.net/ark:/65665/314a59ebc-be4d-4a55-9681-f99f182e3b7d | Chiroptera | Nycteridae | Nycteris hispida | 16.550000 | -15.766667 | M3 |
| http://n2t.net/ark:/65665/3ba30307d-1e00-42ea-bf68-ef226c121df1 | Chiroptera | Nycteridae | Nycteris hispida | 16.550000 | -15.766667 | M3 |
| http://n2t.net/ark:/65665/3c89c7aed-520f-425d-a42d-c012082af92e | Chiroptera | Nycteridae | Nycteris hispida | 16.550000 | -15.766667 | M3 |
| http://n2t.net/ark:/65665/36b450a7b-8020-467d-89db-7a8c8653d017 | Chiroptera | Nycteridae | Nycteris hispida | 16.550000 | -15.766667 | M3 |
| http://n2t.net/ark:/65665/3d5466d75-ffd7-4fc5-9ebc-7ac2d64b1f67 | Chiroptera | Nycteridae | Nycteris hispida | 16.550000 | -15.766667 | M3 |
| http://n2t.net/ark:/65665/3f9fb3ca5-c329-4df1-9dce-cf67baf5fa22 | Chiroptera | Nycteridae | Nycteris hispida | 16.550000 | -15.766667 | M3 |
|  | Chiroptera | Nycteridae | Nycteris macrotis | 16.512550 | -15.804920 | M3 |
|  | Chiroptera | Nycteridae | Nycteris macrotis | 16.512550 | -15.804920 | M3 |
|  | Chiroptera | Nycteridae | Nycteris thebaica | 16.512550 | -15.804920 | M3 |
|  | Chiroptera | Nycteridae | Nycteris thebaica | 16.550000 | -15.766667 | M3 |
|  | Chiroptera | Nycteridae | Nycteris thebaica | 16.550000 | -15.766667 | M3 |
| http://coldb.mnhn.fr/catalognumber/mnhn/zm/mo-1997-2108 | Chiroptera | Pteropodidae | Eidolon helvum | 18.093000 | -15.976000 | K3 |
| http://coldb.mnhn.fr/catalognumber/mnhn/zm/mo-1997-2107 | Chiroptera | Pteropodidae | Eidolon helvum | 18.093000 | -15.976000 | K3 |
| http://coldb.mnhn.fr/catalognumber/mnhn/zm/mo-1997-2106 | Chiroptera | Pteropodidae | Eidolon helvum | 18.093000 | -15.976000 | K3 |
| http://coldb.mnhn.fr/catalognumber/mnhn/zm/mo-1997-2105 | Chiroptera | Pteropodidae | Eidolon helvum | 18.093000 | -15.976000 | K3 |
| https://observation.org/observation/88164669 | Chiroptera | Pteropodidae | Eidolon helvum | 18.101568 | -15.989740 | K3 |
| https://observation.org/observation/88856623 | Chiroptera | Pteropodidae | Eidolon helvum | 18.096357 | -15.980129 | K3 |
| https://observation.org/observation/105931608 | Chiroptera | Pteropodidae | Eidolon helvum | 18.096242 | -15.979332 | K3 |
| https://observation.org/observation/146685626 | Chiroptera | Pteropodidae | Eidolon helvum | 18.093157 | -15.977390 | K3 |
| https://observation.org/observation/146346293 | Chiroptera | Pteropodidae | Eidolon helvum | 18.093157 | -15.977390 | K3 |
| https://observation.org/observation/145555695 | Chiroptera | Pteropodidae | Eidolon helvum | 18.093157 | -15.977390 | K3 |
| https://observation.org/observation/72505492 | Chiroptera | Pteropodidae | Eidolon helvum | 18.092528 | -15.982199 | K3 |
| https://observation.org/observation/126642961 | Chiroptera | Pteropodidae | Eidolon helvum | 18.091515 | -15.975652 | K3 |
| https://observation.org/observation/105943140 | Chiroptera | Pteropodidae | Eidolon helvum | 18.087199 | -15.992479 | K3 |
| https://observation.org/observation/105943139 | Chiroptera | Pteropodidae | Eidolon helvum | 18.086356 | -15.997981 | K3 |
| https://observation.org/observation/72291130 | Chiroptera | Pteropodidae | Eidolon helvum | 18.084343 | -15.988884 | K3 |
|  | Chiroptera | Rhinopomatidae | Rhinopoma cystops | 22.678530 | -12.707130 | F6 |
|  | Chiroptera | Rhinopomatidae | Rhinopoma cystops | 22.678530 | -12.707130 | F6 |
|  | Chiroptera | Rhinopomatidae | Rhinopoma cystops | 22.678530 | -12.707130 | F6 |
|  | Chiroptera | Rhinopomatidae | Rhinopoma cystops | 22.678530 | -12.707130 | F6 |
|  | Chiroptera | Rhinopomatidae | Rhinopoma cystops | 22.678530 | -12.707130 | F6 |
|  | Chiroptera | Rhinopomatidae | Rhinopoma cystops | 22.678530 | -12.707130 | F6 |
|  | Chiroptera | Rhinopomatidae | Rhinopoma cystops | 22.678530 | -12.707130 | F6 |
|  | Chiroptera | Rhinopomatidae | Rhinopoma cystops | 22.678530 | -12.707130 | F6 |
|  | Chiroptera | Rhinopomatidae | Rhinopoma cystops | 22.678530 | -12.707130 | F6 |
|  | Chiroptera | Rhinopomatidae | Rhinopoma cystops | 22.678530 | -12.707130 | F6 |
|  | Chiroptera | Rhinopomatidae | Rhinopoma cystops | 20.465160 | -13.002200 | H6 |
|  | Chiroptera | Rhinopomatidae | Rhinopoma cystops | 20.465160 | -13.002200 | H6 |
|  | Chiroptera | Rhinopomatidae | Rhinopoma cystops | 20.465160 | -13.002200 | H6 |
|  | Chiroptera | Rhinopomatidae | Rhinopoma cystops | 20.465160 | -13.002200 | H6 |
|  | Chiroptera | Rhinopomatidae | Rhinopoma cystops | 20.465160 | -13.002200 | H6 |
|  | Chiroptera | Rhinopomatidae | Rhinopoma cystops | 20.465160 | -13.002200 | H6 |
|  | Chiroptera | Rhinopomatidae | Rhinopoma cystops | 20.465160 | -13.002200 | H6 |
|  | Chiroptera | Rhinopomatidae | Rhinopoma cystops | 20.465160 | -13.002200 | H6 |
|  | Chiroptera | Rhinopomatidae | Rhinopoma cystops | 20.465160 | -13.002200 | H6 |
|  | Chiroptera | Rhinopomatidae | Rhinopoma cystops | 20.465160 | -13.002200 | H6 |
|  | Chiroptera | Rhinopomatidae | Rhinopoma cystops | 19.750000 | -14.383333 | I4 |
|  | Chiroptera | Rhinopomatidae | Rhinopoma cystops | 19.750000 | -14.383333 | I4 |
|  | Chiroptera | Rhinopomatidae | Rhinopoma cystops | 19.750000 | -14.383333 | I4 |
|  | Chiroptera | Rhinopomatidae | Rhinopoma cystops | 19.750000 | -14.383333 | I4 |
|  | Chiroptera | Rhinopomatidae | Rhinopoma cystops | 19.750000 | -14.383333 | I4 |
|  | Chiroptera | Rhinopomatidae | Rhinopoma cystops | 19.750000 | -14.383333 | I4 |
| http://coldb.mnhn.fr/catalognumber/mnhn/zm/mo-1995-1825 | Chiroptera | Rhinopomatidae | Rhinopoma hardwickii | 19.751600 | -14.428100 | I4 |
| http://n2t.net/ark:/65665/3fd9da650-8480-4936-81ce-445609553b8e | Chiroptera | Rhinopomatidae | Rhinopoma hardwickii | 19.750000 | -14.383333 | I4 |
| http://n2t.net/ark:/65665/3e3d110aa-3a94-4e31-b39c-4cae8f3f0f59 | Chiroptera | Rhinopomatidae | Rhinopoma hardwickii | 19.750000 | -14.383333 | I4 |
| http://n2t.net/ark:/65665/30908b383-b1cb-43ae-b2c7-83343a14ee6d | Chiroptera | Rhinopomatidae | Rhinopoma hardwickii | 19.750000 | -14.383333 | I4 |
| http://n2t.net/ark:/65665/3b884d45f-476f-41df-b64d-de11096596dc | Chiroptera | Rhinopomatidae | Rhinopoma hardwickii | 19.750000 | -14.383333 | I4 |
| http://n2t.net/ark:/65665/3f7198406-31ae-4658-9eb5-beeaf7cbdf5a | Chiroptera | Rhinopomatidae | Rhinopoma hardwickii | 19.750000 | -14.383333 | I4 |
| http://n2t.net/ark:/65665/3dba0d88a-5334-4f85-aa49-28c16a19f832 | Chiroptera | Rhinopomatidae | Rhinopoma hardwickii | 19.750000 | -14.383333 | I4 |
| http://n2t.net/ark:/65665/3e499dba1-ee5c-472e-a6e6-1c1579c010d9 | Chiroptera | Rhinopomatidae | Rhinopoma hardwickii | 19.750000 | -14.383333 | I4 |
| http://n2t.net/ark:/65665/3d80b8e87-1257-4f7a-adad-37ef5b82ccfc | Chiroptera | Rhinopomatidae | Rhinopoma hardwickii | 19.750000 | -14.383333 | I4 |
| http://n2t.net/ark:/65665/3d9f2a3f1-ee17-4c1f-88e8-6d17d70ff493 | Chiroptera | Rhinopomatidae | Rhinopoma hardwickii | 19.750000 | -14.383333 | I4 |
| http://n2t.net/ark:/65665/35b22f5ab-9dba-41a9-972f-10d13f968bf5 | Chiroptera | Rhinopomatidae | Rhinopoma hardwickii | 19.750000 | -14.383333 | I4 |
| http://n2t.net/ark:/65665/3bf8fccf8-f2ce-44c5-a0bd-2c644e8b6a2a | Chiroptera | Rhinopomatidae | Rhinopoma hardwickii | 19.750000 | -14.383333 | I4 |
| http://n2t.net/ark:/65665/3437e6fab-bb0f-401f-b70e-22772c8712cb | Chiroptera | Rhinopomatidae | Rhinopoma hardwickii | 19.750000 | -14.383333 | I4 |
| http://n2t.net/ark:/65665/391df1bc8-aaa8-484b-8b4d-277ccb837f97 | Chiroptera | Rhinopomatidae | Rhinopoma hardwickii | 19.750000 | -14.383333 | I4 |
| http://n2t.net/ark:/65665/35ae60f71-bf6b-4a6f-9bcf-d3917ad68c01 | Chiroptera | Rhinopomatidae | Rhinopoma hardwickii | 19.750000 | -14.383333 | I4 |
| http://n2t.net/ark:/65665/3c502ed53-cad8-4c23-84fb-b21130ad2b62 | Chiroptera | Rhinopomatidae | Rhinopoma hardwickii | 19.750000 | -14.383333 | I4 |
| http://n2t.net/ark:/65665/3c0e9424c-f76f-4348-aae3-294f1d342a92 | Chiroptera | Rhinopomatidae | Rhinopoma hardwickii | 19.750000 | -14.383333 | I4 |
| http://n2t.net/ark:/65665/359f8330a-df52-450a-b73c-ba41d35d835e | Chiroptera | Rhinopomatidae | Rhinopoma hardwickii | 19.750000 | -14.383333 | I4 |
| http://n2t.net/ark:/65665/31f21082e-62f9-4944-84ef-32f4390219e2 | Chiroptera | Rhinopomatidae | Rhinopoma hardwickii | 19.750000 | -14.383333 | I4 |
| http://n2t.net/ark:/65665/3b2113ba9-bc5c-41a7-88f7-1b9d84294aba | Chiroptera | Rhinopomatidae | Rhinopoma hardwickii | 19.750000 | -14.383333 | I4 |
| http://n2t.net/ark:/65665/3ed2f1b24-05e9-4bc3-8c0c-9a08765619b2 | Chiroptera | Rhinopomatidae | Rhinopoma hardwickii | 19.750000 | -14.383333 | I4 |
| http://n2t.net/ark:/65665/36ca6fc7c-2ee4-4b80-8da1-352fe16a03d4 | Chiroptera | Rhinopomatidae | Rhinopoma hardwickii | 19.750000 | -14.383333 | I4 |
| http://n2t.net/ark:/65665/39c1ada2d-2da9-43bc-9eaf-2cdd91b1ab55 | Chiroptera | Rhinopomatidae | Rhinopoma hardwickii | 19.750000 | -14.383333 | I4 |
| http://n2t.net/ark:/65665/361e5984c-571b-4d92-9d18-0d56f6c06fad | Chiroptera | Rhinopomatidae | Rhinopoma hardwickii | 19.750000 | -14.383333 | I4 |
| http://n2t.net/ark:/65665/3888b0177-c9fd-43de-b169-0442931504bd | Chiroptera | Rhinopomatidae | Rhinopoma hardwickii | 19.750000 | -14.383333 | I4 |
| http://n2t.net/ark:/65665/352026df2-ec2d-454c-8bb2-ad9cfd54f7d7 | Chiroptera | Rhinopomatidae | Rhinopoma hardwickii | 19.750000 | -14.383333 | I4 |
| http://n2t.net/ark:/65665/3aa02bc27-e8df-4626-b893-1ff42653c77a | Chiroptera | Rhinopomatidae | Rhinopoma hardwickii | 19.750000 | -14.383333 | I4 |
| http://n2t.net/ark:/65665/33cf84e14-4f5f-4231-8c23-ea711aee9032 | Chiroptera | Rhinopomatidae | Rhinopoma hardwickii | 19.750000 | -14.383333 | I4 |
| http://n2t.net/ark:/65665/337639e94-aa50-483a-b2e9-2425f8ba47fe | Chiroptera | Rhinopomatidae | Rhinopoma hardwickii | 19.750000 | -14.383333 | I4 |
| http://n2t.net/ark:/65665/3596828d2-a086-495e-904e-e265db50c19b | Chiroptera | Rhinopomatidae | Rhinopoma hardwickii | 19.750000 | -14.383333 | I4 |
| http://n2t.net/ark:/65665/3d122911c-61e4-4839-99cb-6597eeb531d8 | Chiroptera | Rhinopomatidae | Rhinopoma hardwickii | 19.750000 | -14.383333 | I4 |
| http://n2t.net/ark:/65665/315b01574-0aac-4548-89e7-56baa99cba1d | Chiroptera | Rhinopomatidae | Rhinopoma hardwickii | 19.750000 | -14.383333 | I4 |
| http://n2t.net/ark:/65665/350819e7a-7d6b-496e-ab7b-cabacb7a1a7e | Chiroptera | Rhinopomatidae | Rhinopoma hardwickii | 19.750000 | -14.383333 | I4 |
| http://n2t.net/ark:/65665/324b38978-ff86-46c8-a5be-6d7bb35397ac | Chiroptera | Rhinopomatidae | Rhinopoma hardwickii | 19.750000 | -14.383333 | I4 |
| http://n2t.net/ark:/65665/3d18afed0-92d3-4f23-b100-d2a9db088b13 | Chiroptera | Rhinopomatidae | Rhinopoma hardwickii | 19.750000 | -14.383333 | I4 |
| 9ebabbf1-425e-49d9-8d78-5088d3408cd0 | Chiroptera | Rhinopomatidae | Rhinopoma hardwickii | 17.881083 | -12.094000 | K7 |
| 975ce491-484b-4f04-8e23-7b5d71c5696e | Chiroptera | Rhinopomatidae | Rhinopoma hardwickii | 17.887298 | -12.110844 | K7 |
| 79ba24d2-7e3a-4a8e-bc9e-32694d7327ab | Chiroptera | Rhinopomatidae | Rhinopoma hardwickii | 17.887298 | -12.110844 | K7 |
| e455589c-7570-4fcf-9968-922b721e4563 | Chiroptera | Rhinopomatidae | Rhinopoma hardwickii | 16.864255 | -9.583395 | L9 |
| http://coldb.mnhn.fr/catalognumber/mnhn/zm/mo-1995-3142 | Chiroptera | Rhinopomatidae | Rhinopoma microphyllum | 17.873611 | -12.331667 | K6 |
| http://coldb.mnhn.fr/catalognumber/mnhn/zm/mo-1995-1824 | Chiroptera | Rhinopomatidae | Rhinopoma microphyllum | 19.751600 | -14.428100 | I4 |
| http://n2t.net/ark:/65665/340249b84-cc84-4072-8ab5-1745f9209349 | Chiroptera | Rhinopomatidae | Rhinopoma microphyllum | 19.750000 | -14.383333 | I4 |
| http://n2t.net/ark:/65665/3f7079577-0727-4b77-bbd8-8836081d0261 | Chiroptera | Rhinopomatidae | Rhinopoma microphyllum | 19.750000 | -14.383333 | I4 |
| http://n2t.net/ark:/65665/31683a18c-afb6-489d-90e9-786c8d4f8dd3 | Chiroptera | Rhinopomatidae | Rhinopoma microphyllum | 19.750000 | -14.383333 | I4 |
| http://n2t.net/ark:/65665/30d2ee9d1-c98e-40e6-a869-743fa278d2e7 | Chiroptera | Rhinopomatidae | Rhinopoma microphyllum | 19.750000 | -14.383333 | I4 |
| http://n2t.net/ark:/65665/34eed04d5-5cf4-4bad-ac14-7dfaaa6b9a5a | Chiroptera | Rhinopomatidae | Rhinopoma microphyllum | 19.750000 | -14.383333 | I4 |
| http://n2t.net/ark:/65665/3fb9bd574-cdf5-4c22-994e-311003571108 | Chiroptera | Rhinopomatidae | Rhinopoma microphyllum | 19.750000 | -14.383333 | I4 |
| http://n2t.net/ark:/65665/39be9df2e-7ce2-4e4b-a5ba-2865dcfe5299 | Chiroptera | Rhinopomatidae | Rhinopoma microphyllum | 19.750000 | -14.383333 | I4 |
| http://n2t.net/ark:/65665/386b57df4-4a03-40de-a2c2-c6ce73801b92 | Chiroptera | Rhinopomatidae | Rhinopoma microphyllum | 19.750000 | -14.383333 | I4 |
| http://n2t.net/ark:/65665/33ffbf4cd-d83f-4dbb-95b3-333dffcbfa8f | Chiroptera | Rhinopomatidae | Rhinopoma microphyllum | 19.750000 | -14.383333 | I4 |
| http://n2t.net/ark:/65665/372ccb374-9ac2-4f5f-8926-12f3c003675c | Chiroptera | Rhinopomatidae | Rhinopoma microphyllum | 19.750000 | -14.383333 | I4 |
| http://n2t.net/ark:/65665/3e541489d-f469-4ab7-8d9e-37d2c94abea0 | Chiroptera | Rhinopomatidae | Rhinopoma microphyllum | 19.750000 | -14.383333 | I4 |
| http://n2t.net/ark:/65665/3ec9c93e5-4baa-4489-8a59-5fc5ed6a9fc8 | Chiroptera | Rhinopomatidae | Rhinopoma microphyllum | 19.750000 | -14.383333 | I4 |
| http://n2t.net/ark:/65665/378682a61-79ba-47c0-8132-bde3529752aa | Chiroptera | Rhinopomatidae | Rhinopoma microphyllum | 19.750000 | -14.383333 | I4 |
| http://n2t.net/ark:/65665/3ba7f0b6f-eeee-42e5-be42-46f76b17d628 | Chiroptera | Rhinopomatidae | Rhinopoma microphyllum | 19.750000 | -14.383333 | I4 |
| http://n2t.net/ark:/65665/33dbb80c1-7d20-499e-b7c9-a61cca2e53ce | Chiroptera | Rhinopomatidae | Rhinopoma microphyllum | 19.750000 | -14.383333 | I4 |
| http://n2t.net/ark:/65665/30b4f48f0-46e5-45f4-8496-427af9420c72 | Chiroptera | Rhinopomatidae | Rhinopoma microphyllum | 19.750000 | -14.383333 | I4 |
| http://n2t.net/ark:/65665/357aedd55-eeb4-47cb-b4d0-943c0194620e | Chiroptera | Rhinopomatidae | Rhinopoma microphyllum | 19.750000 | -14.383333 | I4 |
| http://n2t.net/ark:/65665/357d6fbeb-b86b-49c8-a5f5-ce0862ed5b4e | Chiroptera | Rhinopomatidae | Rhinopoma microphyllum | 19.750000 | -14.383333 | I4 |
| http://n2t.net/ark:/65665/366ee7d62-d670-4b3a-aec0-d769140e0014 | Chiroptera | Rhinopomatidae | Rhinopoma microphyllum | 19.750000 | -14.383333 | I4 |
| http://n2t.net/ark:/65665/383b8a242-2293-4428-b33f-5746688cb860 | Chiroptera | Rhinopomatidae | Rhinopoma microphyllum | 19.750000 | -14.383333 | I4 |
| http://n2t.net/ark:/65665/334bff9ce-3c1e-4873-9bb1-010057519ed7 | Chiroptera | Rhinopomatidae | Rhinopoma microphyllum | 19.750000 | -14.383333 | I4 |
| http://n2t.net/ark:/65665/323eb3edb-1cc2-40c0-a80b-b725a77a77d0 | Chiroptera | Rhinopomatidae | Rhinopoma microphyllum | 19.750000 | -14.383333 | I4 |
| http://n2t.net/ark:/65665/3ba6b73ec-9cdf-49d1-93be-22ff33149453 | Chiroptera | Rhinopomatidae | Rhinopoma microphyllum | 19.750000 | -14.383333 | I4 |
| http://n2t.net/ark:/65665/3ff63340d-95fe-44e9-9a98-d331e6deabbd | Chiroptera | Rhinopomatidae | Rhinopoma microphyllum | 19.750000 | -14.383333 | I4 |
| http://n2t.net/ark:/65665/364e5b537-e59e-43d5-a51b-3620f157540c | Chiroptera | Rhinopomatidae | Rhinopoma microphyllum | 19.750000 | -14.383333 | I4 |
| http://n2t.net/ark:/65665/358b6f170-2664-4929-b68d-a13dbc2f0645 | Chiroptera | Rhinopomatidae | Rhinopoma microphyllum | 19.750000 | -14.383333 | I4 |
| http://n2t.net/ark:/65665/35c3cd1a2-7892-4690-9715-124be3f7f789 | Chiroptera | Rhinopomatidae | Rhinopoma microphyllum | 19.750000 | -14.383333 | I4 |
| http://n2t.net/ark:/65665/3a0cea6a8-471d-441b-8c95-344ebf6b169c | Chiroptera | Rhinopomatidae | Rhinopoma microphyllum | 19.750000 | -14.383333 | I4 |
| http://n2t.net/ark:/65665/3060b6c50-8c8d-4c13-b656-ee3d6ee722c6 | Chiroptera | Rhinopomatidae | Rhinopoma microphyllum | 19.750000 | -14.383333 | I4 |
| http://n2t.net/ark:/65665/3ce030c98-4cf7-41cd-9520-59c13468b0c6 | Chiroptera | Rhinopomatidae | Rhinopoma microphyllum | 19.750000 | -14.383333 | I4 |
| http://n2t.net/ark:/65665/32aa0c930-6b5c-4510-b6c9-5b7969888078 | Chiroptera | Rhinopomatidae | Rhinopoma microphyllum | 19.750000 | -14.383333 | I4 |
| http://n2t.net/ark:/65665/35907f067-fa9f-4706-b17e-df2348b8c9c1 | Chiroptera | Rhinopomatidae | Rhinopoma microphyllum | 19.750000 | -14.383333 | I4 |
| http://n2t.net/ark:/65665/38bd979b2-7dec-45d1-9e36-cc0c79db74c1 | Chiroptera | Rhinopomatidae | Rhinopoma microphyllum | 19.750000 | -14.383333 | I4 |
| http://n2t.net/ark:/65665/31f1c0dc6-256b-4662-91c7-64bb31fb8a75 | Chiroptera | Rhinopomatidae | Rhinopoma microphyllum | 19.750000 | -14.383333 | I4 |
| http://n2t.net/ark:/65665/365ee3ae4-388b-41c8-83cd-e663b3185072 | Chiroptera | Rhinopomatidae | Rhinopoma microphyllum | 19.750000 | -14.383333 | I4 |
| http://n2t.net/ark:/65665/3d4552a5b-4482-43db-9f1b-3bb40ac4df86 | Chiroptera | Rhinopomatidae | Rhinopoma microphyllum | 19.750000 | -14.383333 | I4 |
| http://n2t.net/ark:/65665/314315934-2c5f-4fda-b678-acc4fd7b4992 | Chiroptera | Rhinopomatidae | Rhinopoma microphyllum | 19.750000 | -14.383333 | I4 |
| http://n2t.net/ark:/65665/3d4be0109-397d-4b1b-aca5-79a475aa7a67 | Chiroptera | Rhinopomatidae | Rhinopoma microphyllum | 19.750000 | -14.383333 | I4 |
| http://n2t.net/ark:/65665/30d70e94b-bfc8-44c3-99f3-c6b9ef6f3f0f | Chiroptera | Rhinopomatidae | Rhinopoma microphyllum | 19.750000 | -14.383333 | I4 |
| http://n2t.net/ark:/65665/3fd8dda81-6298-4eab-ae22-27d5586f7eee | Chiroptera | Rhinopomatidae | Rhinopoma microphyllum | 19.750000 | -14.383333 | I4 |
| http://n2t.net/ark:/65665/3a78a8210-d56e-4e13-afd5-902ff2051a06 | Chiroptera | Rhinopomatidae | Rhinopoma microphyllum | 19.750000 | -14.383333 | I4 |
| http://n2t.net/ark:/65665/340be0ac4-722e-483e-87b6-efc3953bc1b3 | Chiroptera | Rhinopomatidae | Rhinopoma microphyllum | 19.750000 | -14.383333 | I4 |
| http://n2t.net/ark:/65665/3cfa1fba0-214a-4102-a61f-9bbd4a238d1d | Chiroptera | Rhinopomatidae | Rhinopoma microphyllum | 19.750000 | -14.383333 | I4 |
| http://n2t.net/ark:/65665/36a573f83-41f5-4982-b15a-f05afa54189d | Chiroptera | Rhinopomatidae | Rhinopoma microphyllum | 19.750000 | -14.383333 | I4 |
| 214463111 | Chiroptera | Vespertilionidae | Eptesicus floweri | 16.499483 | -7.715183 | L11 |
| 214463110 | Chiroptera | Vespertilionidae | Eptesicus floweri | 16.499483 | -7.715183 | L11 |
| http://n2t.net/ark:/65665/32351678e-beb0-4668-b01c-7e7abc418bb8 | Chiroptera | Vespertilionidae | Nycticeinops schlieffeni | 17.266783 | -16.028417 | L3 |
| http://n2t.net/ark:/65665/3414a1f56-dc7c-481d-a0b7-cbf2a117d312 | Chiroptera | Vespertilionidae | Nycticeinops schlieffeni | 17.266783 | -16.028417 | L3 |
| http://n2t.net/ark:/65665/39a32c382-a0da-45e3-bcef-70ca86ba2f03 | Chiroptera | Vespertilionidae | Nycticeinops schlieffeni | 17.266783 | -16.028417 | L3 |
| http://n2t.net/ark:/65665/32f225948-b230-4d05-9bd3-800a52600f4b | Chiroptera | Vespertilionidae | Nycticeinops schlieffeni | 17.266783 | -16.028417 | L3 |
| http://n2t.net/ark:/65665/3091cbad5-4b04-4510-b251-6525ec439f34 | Chiroptera | Vespertilionidae | Nycticeinops schlieffeni | 17.266783 | -16.028417 | L3 |
| http://n2t.net/ark:/65665/3add3b59b-f305-4ac0-8efd-2b37af4fe3c5 | Chiroptera | Vespertilionidae | Nycticeinops schlieffeni | 15.933333 | -12.016667 | M7 |
| http://n2t.net/ark:/65665/3744190b1-24ae-4d3f-8f92-bf3a98074168 | Chiroptera | Vespertilionidae | Nycticeinops schlieffeni | 15.933333 | -12.016667 | M7 |
| http://coldb.mnhn.fr/catalognumber/mnhn/zm/mo-1997-2104 | Chiroptera | Vespertilionidae | Nycticeinops schlieffeni | 16.222172 | -16.490439 | M2 |
| http://coldb.mnhn.fr/catalognumber/mnhn/zm/mo-1913-662za | Chiroptera | Vespertilionidae | Nycticeinops schlieffeni | 17.546667 | -14.694444 | K4 |
| http://coldb.mnhn.fr/catalognumber/mnhn/zm/mo-1913-662z | Chiroptera | Vespertilionidae | Nycticeinops schlieffeni | 17.546667 | -14.694444 | K4 |
| http://coldb.mnhn.fr/catalognumber/mnhn/zm/mo-1913-662y | Chiroptera | Vespertilionidae | Nycticeinops schlieffeni | 17.546667 | -14.694444 | K4 |
| http://coldb.mnhn.fr/catalognumber/mnhn/zm/mo-1913-662x | Chiroptera | Vespertilionidae | Nycticeinops schlieffeni | 17.546667 | -14.694444 | K4 |
| http://coldb.mnhn.fr/catalognumber/mnhn/zm/mo-1913-662w | Chiroptera | Vespertilionidae | Nycticeinops schlieffeni | 17.546667 | -14.694444 | K4 |
| http://coldb.mnhn.fr/catalognumber/mnhn/zm/mo-1913-662v | Chiroptera | Vespertilionidae | Nycticeinops schlieffeni | 17.546667 | -14.694444 | K4 |
| http://coldb.mnhn.fr/catalognumber/mnhn/zm/mo-1913-662u | Chiroptera | Vespertilionidae | Nycticeinops schlieffeni | 17.546667 | -14.694444 | K4 |
| http://coldb.mnhn.fr/catalognumber/mnhn/zm/mo-1913-662t | Chiroptera | Vespertilionidae | Nycticeinops schlieffeni | 17.546667 | -14.694444 | K4 |
| http://coldb.mnhn.fr/catalognumber/mnhn/zm/mo-1913-662s | Chiroptera | Vespertilionidae | Nycticeinops schlieffeni | 17.546667 | -14.694444 | K4 |
| http://coldb.mnhn.fr/catalognumber/mnhn/zm/mo-1913-662r | Chiroptera | Vespertilionidae | Nycticeinops schlieffeni | 17.546667 | -14.694444 | K4 |
| http://coldb.mnhn.fr/catalognumber/mnhn/zm/mo-1913-662q | Chiroptera | Vespertilionidae | Nycticeinops schlieffeni | 17.546667 | -14.694444 | K4 |
| http://coldb.mnhn.fr/catalognumber/mnhn/zm/mo-1913-662p | Chiroptera | Vespertilionidae | Nycticeinops schlieffeni | 17.546667 | -14.694444 | K4 |
| http://coldb.mnhn.fr/catalognumber/mnhn/zm/mo-1913-662o | Chiroptera | Vespertilionidae | Nycticeinops schlieffeni | 17.546667 | -14.694444 | K4 |
| http://coldb.mnhn.fr/catalognumber/mnhn/zm/mo-1913-662n | Chiroptera | Vespertilionidae | Nycticeinops schlieffeni | 17.546667 | -14.694444 | K4 |
| http://coldb.mnhn.fr/catalognumber/mnhn/zm/mo-1913-662m | Chiroptera | Vespertilionidae | Nycticeinops schlieffeni | 17.546667 | -14.694444 | K4 |
| http://coldb.mnhn.fr/catalognumber/mnhn/zm/mo-1913-662l | Chiroptera | Vespertilionidae | Nycticeinops schlieffeni | 17.546667 | -14.694444 | K4 |
| http://coldb.mnhn.fr/catalognumber/mnhn/zm/mo-1913-662k | Chiroptera | Vespertilionidae | Nycticeinops schlieffeni | 17.546667 | -14.694444 | K4 |
| http://coldb.mnhn.fr/catalognumber/mnhn/zm/mo-1913-662j | Chiroptera | Vespertilionidae | Nycticeinops schlieffeni | 17.546667 | -14.694444 | K4 |
| http://coldb.mnhn.fr/catalognumber/mnhn/zm/mo-1913-662i | Chiroptera | Vespertilionidae | Nycticeinops schlieffeni | 17.546667 | -14.694444 | K4 |
| http://coldb.mnhn.fr/catalognumber/mnhn/zm/mo-1913-662h | Chiroptera | Vespertilionidae | Nycticeinops schlieffeni | 17.546667 | -14.694444 | K4 |
| http://coldb.mnhn.fr/catalognumber/mnhn/zm/mo-1913-662g | Chiroptera | Vespertilionidae | Nycticeinops schlieffeni | 17.546667 | -14.694444 | K4 |
| http://coldb.mnhn.fr/catalognumber/mnhn/zm/mo-1913-662f | Chiroptera | Vespertilionidae | Nycticeinops schlieffeni | 17.546667 | -14.694444 | K4 |
| http://coldb.mnhn.fr/catalognumber/mnhn/zm/mo-1913-662e | Chiroptera | Vespertilionidae | Nycticeinops schlieffeni | 17.546667 | -14.694444 | K4 |
| http://coldb.mnhn.fr/catalognumber/mnhn/zm/mo-1913-662d | Chiroptera | Vespertilionidae | Nycticeinops schlieffeni | 17.546667 | -14.694444 | K4 |
| http://coldb.mnhn.fr/catalognumber/mnhn/zm/mo-1913-662c | Chiroptera | Vespertilionidae | Nycticeinops schlieffeni | 17.546667 | -14.694444 | K4 |
| http://coldb.mnhn.fr/catalognumber/mnhn/zm/mo-1913-662b | Chiroptera | Vespertilionidae | Nycticeinops schlieffeni | 17.546667 | -14.694444 | K4 |
| http://coldb.mnhn.fr/catalognumber/mnhn/zm/mo-1913-662a | Chiroptera | Vespertilionidae | Nycticeinops schlieffeni | 17.546667 | -14.694444 | K4 |
| http://n2t.net/ark:/65665/305d33360-795a-41db-98f8-b7f7fd7d091c | Chiroptera | Vespertilionidae | Nycticeinops schlieffeni | 16.650000 | -14.283333 | L4 |
| http://n2t.net/ark:/65665/31ca82ca2-af65-42c8-ac42-3b9105462524 | Chiroptera | Vespertilionidae | Nycticeinops schlieffeni | 17.030000 | -13.920000 | L5 |
| http://n2t.net/ark:/65665/320625510-caa1-42cc-9f13-0f743dd0f73d | Chiroptera | Vespertilionidae | Nycticeinops schlieffeni | 17.030000 | -13.920000 | L5 |
| http://n2t.net/ark:/65665/330b015c1-72cb-4a1f-9f21-888647d0fe0a | Chiroptera | Vespertilionidae | Nycticeinops schlieffeni | 17.030000 | -13.920000 | L5 |
| http://n2t.net/ark:/65665/37bbc7cf9-1a59-45fe-a880-9270d7e3cc18 | Chiroptera | Vespertilionidae | Nycticeinops schlieffeni | 17.030000 | -13.920000 | L5 |
| 214467518 | Chiroptera | Vespertilionidae | Pipistrellus rueppellii | 16.499483 | -7.715183 | L11 |
| http://n2t.net/ark:/65665/35bc48a72-9af7-4090-bf43-82926cfecc6e | Chiroptera | Vespertilionidae | Pipistrellus rueppellii | 16.550000 | -15.766667 | M3 |
| http://n2t.net/ark:/65665/3670a0024-4531-4f4d-8d34-e54133457f0a | Chiroptera | Vespertilionidae | Pipistrellus rueppellii | 16.550000 | -15.766667 | M3 |
| http://n2t.net/ark:/65665/3ae0f10e0-7300-4e12-9919-9729f17cab74 | Chiroptera | Vespertilionidae | Scotophilus leucogaster | 17.030000 | -13.920000 | L5 |
| http://n2t.net/ark:/65665/38dd0639a-c9ee-420e-acee-8d15b44bac67 | Chiroptera | Vespertilionidae | Scotophilus leucogaster | 17.030000 | -13.920000 | L5 |
| http://coldb.mnhn.fr/catalognumber/mnhn/zm/mo-1992-1490 | Erinaceomorpha | Erinaceidae | Atelerix albiventris | 16.150000 | -13.500000 | M5 |
| 1d2ed27c-7183-4d33-a99d-e10c15fe1ea9 | Erinaceomorpha | Erinaceidae | Atelerix albiventris | 16.992968 | -12.327717 | L6 |
| fc8bc1c9-886e-420b-9b2a-1c7bb546d19f | Erinaceomorpha | Erinaceidae | Atelerix albiventris | 16.261320 | -13.715453 | M5 |
| 7e7b6b9c-aa26-4160-b0f0-3097b9f99d1d | Erinaceomorpha | Erinaceidae | Atelerix albiventris | 16.674617 | -16.394827 | L2 |
| d4bc339c-15ca-4e71-aab8-9a47dace2e43 | Erinaceomorpha | Erinaceidae | Atelerix albiventris | 16.292778 | -13.815525 | M5 |
| e9f53fe6-6078-41fa-a438-c2bcc99c46f4 | Erinaceomorpha | Erinaceidae | Atelerix albiventris | 18.206040 | -11.730977 | K7 |
| e2a66c87-c5af-4e15-a6c8-b79bd81aa18c | Erinaceomorpha | Erinaceidae | Atelerix albiventris | 16.385822 | -11.376077 | M7 |
| cbd70a99-9148-4779-bc10-6007d586bf88 | Erinaceomorpha | Erinaceidae | Atelerix albiventris | 17.424440 | -13.357260 | L5 |
| 3432342e-1bb5-4140-ac67-183ca32badb4 | Erinaceomorpha | Erinaceidae | Atelerix albiventris | 20.095725 | -13.203550 | I6 |
| 68f81271-7f26-476d-ab65-3dc13c222a1a | Erinaceomorpha | Erinaceidae | Atelerix albiventris | 16.540298 | -10.865143 | L8 |
| 8e7cbb27-b532-4adf-8ccd-7d2b5a93bb49 | Erinaceomorpha | Erinaceidae | Atelerix albiventris | 18.528000 | -10.229033 | J9 |
| http://coldb.mnhn.fr/catalognumber/mnhn/zm/mo-1995-3140 | Erinaceomorpha | Erinaceidae | Paraechinus aethiopicus | 18.192000 | -16.032000 | K3 |
| http://coldb.mnhn.fr/catalognumber/mnhn/zm/mo-1995-3141 | Erinaceomorpha | Erinaceidae | Paraechinus aethiopicus | 18.115564 | -16.015721 | K3 |
| http://n2t.net/ark:/65665/3b96787e6-15c6-4a68-995b-c63b7e1c46b0 | Erinaceomorpha | Erinaceidae | Paraechinus aethiopicus | 16.620000 | -11.400000 | L7 |
| http://n2t.net/ark:/65665/340d17534-292a-4744-8dea-917d83de50cc | Erinaceomorpha | Erinaceidae | Paraechinus aethiopicus | 16.620000 | -11.400000 | L7 |
| http://n2t.net/ark:/65665/3daf03c79-8d98-4a46-9fcd-06c81f4886b2 | Erinaceomorpha | Erinaceidae | Paraechinus aethiopicus | 16.620000 | -11.400000 | L7 |
| http://coldb.mnhn.fr/catalognumber/mnhn/zm/mo-1986-244 | Erinaceomorpha | Erinaceidae | Paraechinus aethiopicus | 25.220000 | -11.577000 | C7 |
| http://n2t.net/ark:/65665/304dfd974-2fe4-41ce-b465-f22c2478b793 | Erinaceomorpha | Erinaceidae | Paraechinus aethiopicus | 20.520000 | -13.050000 | H6 |
| 1f45f45b-0618-44ac-bc8d-5a0452a2e5e5 | Erinaceomorpha | Erinaceidae | Paraechinus aethiopicus | 20.424783 | -15.993853 | H3 |
| e9df4409-b24a-4ec1-a453-fcc7e0ddbba8 | Erinaceomorpha | Erinaceidae | Paraechinus aethiopicus | 17.646233 | -14.814192 | K4 |
| 0ce742b3-2e7f-4bf5-a66d-ce402b2e6767 | Erinaceomorpha | Erinaceidae | Paraechinus aethiopicus | 20.920755 | -16.213315 | H2 |
| 3d88face-30c7-4080-81bc-d495961c4acc | Erinaceomorpha | Erinaceidae | Paraechinus aethiopicus | 17.612773 | -12.752475 | K6 |
| 8893e274-1693-46c2-aa4a-56b34a2c980c | Erinaceomorpha | Erinaceidae | Paraechinus aethiopicus | 16.489543 | -11.057988 | M8 |
| a17796f8-5da1-43d8-88f3-4e8e9e9941a5 | Erinaceomorpha | Erinaceidae | Paraechinus aethiopicus | 16.489543 | -11.057988 | M8 |
| 40bfbe32-7943-42b2-a974-39208a72cc9d | Erinaceomorpha | Erinaceidae | Paraechinus aethiopicus | 17.597535 | -16.020713 | K3 |
| 04d9ebbe-9e6b-4f07-bbd0-b576f8a4c97f | Erinaceomorpha | Erinaceidae | Paraechinus aethiopicus | 20.717740 | -11.882700 | H7 |
| 318d8b80-e314-4234-8492-44052ab4b337 | Erinaceomorpha | Erinaceidae | Paraechinus aethiopicus | 21.487200 | -11.336028 | G7 |
| e0a53429-abed-4a09-9ec3-a7ca8a5d9f58 | Erinaceomorpha | Erinaceidae | Paraechinus aethiopicus | 20.789622 | -16.458048 | H2 |
| b8e938aa-831f-465c-b95f-25530d3b0ddd | Erinaceomorpha | Erinaceidae | Paraechinus aethiopicus | 19.787547 | -16.151107 | I2 |
| 6377a883-d9c6-49f9-a1de-4e42db82b522 | Erinaceomorpha | Erinaceidae | Paraechinus aethiopicus | 19.567590 | -16.383732 | I2 |
| 4c037949-58a4-47b7-bce3-99abe18569de | Erinaceomorpha | Erinaceidae | Paraechinus aethiopicus | 18.982345 | -16.185970 | J2 |
| 41a42ec2-04ab-4941-b325-d1e47d2c62b0 | Erinaceomorpha | Erinaceidae | Paraechinus aethiopicus | 17.632735 | -11.460103 | K7 |
| ebc135fd-fac2-49df-9eeb-e1db907265cf | Erinaceomorpha | Erinaceidae | Paraechinus aethiopicus | 18.255107 | -11.759570 | K7 |
| b4571653-3b8e-4f16-ba6a-fe70421229e7 | Erinaceomorpha | Erinaceidae | Paraechinus aethiopicus | 19.993367 | -13.928983 | I5 |
| 90a96a1e-d019-4c73-9ca6-cbbc5d02a2c0 | Erinaceomorpha | Erinaceidae | Paraechinus aethiopicus | 20.127462 | -13.691313 | I5 |
| 73c00eba-6332-4d9a-abb2-8ca08f9f5f72 | Erinaceomorpha | Erinaceidae | Paraechinus aethiopicus | 20.269873 | -13.230292 | H5 |
| 6220de09-e39c-4196-b34c-f3407c589149 | Erinaceomorpha | Erinaceidae | Paraechinus aethiopicus | 20.269873 | -13.230292 | H5 |
| b61d20d5-4a51-4324-9498-f2b6e3099813 | Erinaceomorpha | Erinaceidae | Paraechinus aethiopicus | 20.577201 | -12.458330 | H6 |
| a2c30012-3dd6-4ea7-8054-f392f1e055b7 | Erinaceomorpha | Erinaceidae | Paraechinus aethiopicus | 22.064895 | -12.711007 | F6 |
| 50c94b4d-3c8c-408a-97d2-daeb14341aa9 | Erinaceomorpha | Erinaceidae | Paraechinus aethiopicus | 21.519632 | -12.853367 | G6 |
| fb43fb32-703e-4cfd-ab9f-608ae560f34b | Erinaceomorpha | Erinaceidae | Paraechinus aethiopicus | 20.413142 | -14.956897 | H4 |
| d97442a2-4c12-462d-9b62-3e854e1d0786 | Erinaceomorpha | Erinaceidae | Paraechinus aethiopicus | 17.172840 | -13.869968 | L5 |
| 681d4e50-5578-4215-8e24-7d8a0afdb56c | Erinaceomorpha | Erinaceidae | Paraechinus aethiopicus | 17.576838 | -12.885510 | K6 |
| 8a55c95b-96ed-45ba-b046-82b7c3c10801 | Erinaceomorpha | Erinaceidae | Paraechinus aethiopicus | 17.583075 | -12.866542 | K6 |
| 03a30c37-ef25-471e-b5cc-0692a5c1a636 | Erinaceomorpha | Erinaceidae | Paraechinus aethiopicus | 19.462235 | -16.070569 | I3 |
| aa78aaf1-f45f-4cc1-9ef3-63293c77b631 | Erinaceomorpha | Erinaceidae | Paraechinus aethiopicus | 17.837285 | -14.560327 | K4 |
| ba964f46-7986-4644-9c36-7a1396dfd968 | Erinaceomorpha | Erinaceidae | Paraechinus aethiopicus | 17.993012 | -12.247403 | K7 |
| 66d19d27-4a82-4732-9492-8b79aff9f821 | Erinaceomorpha | Erinaceidae | Paraechinus aethiopicus | 18.255785 | -11.785698 | K7 |
| 53e85d8c-52c2-4dd0-9e43-199e1023e214 | Erinaceomorpha | Erinaceidae | Paraechinus aethiopicus | 18.205859 | -11.972349 | K7 |
| 42e48d60-a67a-48ef-b018-d4d37a559122 | Erinaceomorpha | Erinaceidae | Paraechinus aethiopicus | 20.531408 | -13.026740 | H6 |
| e2531dfa-f410-40d9-8a54-8814906b8a7c | Erinaceomorpha | Erinaceidae | Paraechinus aethiopicus | 20.469647 | -16.234562 | H2 |
| 0a023ce2-f7d7-49ad-bf03-71794145b43b | Erinaceomorpha | Erinaceidae | Paraechinus aethiopicus | 16.382675 | -9.564807 | M9 |
| c5e28442-64d4-4a5c-be43-256c3656a746 | Erinaceomorpha | Erinaceidae | Paraechinus aethiopicus | 17.249855 | -10.667613 | L8 |
| d9a0cfc3-ca33-4848-9eed-03584b2dd41c | Erinaceomorpha | Erinaceidae | Paraechinus aethiopicus | 18.874512 | -11.817575 | J7 |
| f373d716-524b-4f24-85f9-37623289d1f3 | Erinaceomorpha | Erinaceidae | Paraechinus aethiopicus | 16.302783 | -13.879300 | M5 |
| 3bceabc3-0f4d-4e0e-b9a1-47ee551dedc2 | Erinaceomorpha | Erinaceidae | Paraechinus aethiopicus | 19.844800 | -14.246483 | I4 |
| 5579598a-0a1d-4360-965e-3fd1fb9c2da0 | Erinaceomorpha | Erinaceidae | Paraechinus aethiopicus | 19.643033 | -14.517133 | I4 |
| 3d3f75b0-822c-4a5f-98f7-16c370cca4ce | Erinaceomorpha | Erinaceidae | Paraechinus aethiopicus | 19.608867 | -14.570167 | I4 |
| 2b73fefb-f832-459d-82e7-7b39f58500ce | Erinaceomorpha | Erinaceidae | Paraechinus aethiopicus | 19.558300 | -14.632433 | I4 |
| http://coldb.mnhn.fr/catalognumber/mnhn/zm/mo-1937-1258 | Hyracoidea | Procaviidae | Procavia capensis | 18.552000 | -11.428000 | J7 |
| http://coldb.mnhn.fr/catalognumber/mnhn/zm/mo-1995-3152 | Hyracoidea | Procaviidae | Procavia capensis | 20.251732 | -13.086483 | H6 |
| http://n2t.net/ark:/65665/3e58fa541-0f28-4dc2-9556-5a32c82487a5 | Hyracoidea | Procaviidae | Procavia capensis | 15.933333 | -12.016667 | M7 |
| http://n2t.net/ark:/65665/3b7fdf944-4e5c-4a12-acdb-66f1d627e18d | Hyracoidea | Procaviidae | Procavia capensis | 15.933333 | -12.016667 | M7 |
| http://n2t.net/ark:/65665/3f88e046a-229d-487b-ba2c-a5e88a446077 | Hyracoidea | Procaviidae | Procavia capensis | 15.933333 | -12.016667 | M7 |
| http://n2t.net/ark:/65665/343d49320-4f82-4d92-bb45-d62eadb5a3a8 | Hyracoidea | Procaviidae | Procavia capensis | 15.933333 | -12.016667 | M7 |
| http://n2t.net/ark:/65665/3bc1d7a1c-503b-47a5-be07-0f8b01d835af | Hyracoidea | Procaviidae | Procavia capensis | 15.933333 | -12.016667 | M7 |
| http://n2t.net/ark:/65665/3f0c2aeed-08e8-4ae7-83f5-57b74d9ab870 | Hyracoidea | Procaviidae | Procavia capensis | 15.933333 | -12.016667 | M7 |
| http://n2t.net/ark:/65665/330fcd795-32c5-4294-ac5e-6d7364219720 | Hyracoidea | Procaviidae | Procavia capensis | 15.933333 | -12.016667 | M7 |
| http://n2t.net/ark:/65665/33f541344-07c1-4327-8607-31eb6a9dfcfc | Hyracoidea | Procaviidae | Procavia capensis | 15.933333 | -12.016667 | M7 |
| http://n2t.net/ark:/65665/3557736fa-e9f4-4893-8daa-3282220479b9 | Hyracoidea | Procaviidae | Procavia capensis | 15.933333 | -12.016667 | M7 |
| http://n2t.net/ark:/65665/34149eb05-c0ef-4110-a11f-7c63a853167c | Hyracoidea | Procaviidae | Procavia capensis | 15.933333 | -12.016667 | M7 |
| http://n2t.net/ark:/65665/3327a8987-12fa-42f6-bba0-3cc147c83ea5 | Hyracoidea | Procaviidae | Procavia capensis | 15.933333 | -12.016667 | M7 |
| http://n2t.net/ark:/65665/35f2409fb-16fb-40cc-a670-28cf3de720ec | Hyracoidea | Procaviidae | Procavia capensis | 15.933333 | -12.016667 | M7 |
| http://n2t.net/ark:/65665/323e58dd9-7f24-4d91-ad4c-c83bbef90e63 | Hyracoidea | Procaviidae | Procavia capensis | 15.933333 | -12.016667 | M7 |
| http://n2t.net/ark:/65665/37eb6ae74-3103-4cad-90fc-66e78a171b10 | Hyracoidea | Procaviidae | Procavia capensis | 15.933333 | -12.016667 | M7 |
| http://n2t.net/ark:/65665/30c5144a9-96f5-4bb6-b7bd-9c7019bf42b3 | Hyracoidea | Procaviidae | Procavia capensis | 15.933333 | -12.016667 | M7 |
| http://n2t.net/ark:/65665/341cc9f86-9e24-4e18-827f-2080720b193b | Hyracoidea | Procaviidae | Procavia capensis | 15.933333 | -12.016667 | M7 |
| http://n2t.net/ark:/65665/3e6b706bb-b755-42e2-810f-dec2c6c7151e | Hyracoidea | Procaviidae | Procavia capensis | 15.933333 | -12.016667 | M7 |
| http://n2t.net/ark:/65665/337d7e01b-4cea-4803-bc03-dde5dbcf6d9f | Hyracoidea | Procaviidae | Procavia capensis | 15.933333 | -12.016667 | M7 |
| http://n2t.net/ark:/65665/3922ced84-88f2-42a8-babd-4b8da97fcb76 | Hyracoidea | Procaviidae | Procavia capensis | 15.933333 | -12.016667 | M7 |
| http://n2t.net/ark:/65665/33cd12676-ee8a-4c8d-bb12-1e3bcc8e92d2 | Hyracoidea | Procaviidae | Procavia capensis | 15.933333 | -12.016667 | M7 |
| http://n2t.net/ark:/65665/31a6d4e35-9fd9-4199-80d7-4b9d0d506ad9 | Hyracoidea | Procaviidae | Procavia capensis | 15.933333 | -12.016667 | M7 |
| http://n2t.net/ark:/65665/3bf02d7d0-4cae-402c-baf2-e8ec536da0b6 | Hyracoidea | Procaviidae | Procavia capensis | 15.933333 | -12.016667 | M7 |
| http://n2t.net/ark:/65665/34513ab1e-c9f7-4f55-8480-da1a42498505 | Hyracoidea | Procaviidae | Procavia capensis | 15.933333 | -12.016667 | M7 |
| http://n2t.net/ark:/65665/352bdfb99-0381-4dbb-9445-2fafc5220924 | Hyracoidea | Procaviidae | Procavia capensis | 15.933333 | -12.016667 | M7 |
| https://observation.org/observation/97111874 | Hyracoidea | Procaviidae | Procavia capensis | 20.624815 | -13.129509 | H6 |
| http://coldb.mnhn.fr/catalognumber/mnhn/zm/mo-2001-1989 | Hyracoidea | Procaviidae | Procavia capensis | 19.606000 | -12.991000 | I6 |
| http://coldb.mnhn.fr/catalognumber/mnhn/zm/mo-2001-1990 | Hyracoidea | Procaviidae | Procavia capensis | 20.778198 | -11.871185 | H7 |
| http://coldb.mnhn.fr/catalognumber/mnhn/zm/mo-1992-1491 | Hyracoidea | Procaviidae | Procavia capensis | 20.536743 | -12.961179 | H6 |
| http://coldb.mnhn.fr/catalognumber/mnhn/zm/mo-1995-561 | Hyracoidea | Procaviidae | Procavia capensis | 20.536743 | -12.961179 | H6 |
| http://coldb.mnhn.fr/catalognumber/mnhn/zm/mo-1977-302 | Hyracoidea | Procaviidae | Procavia capensis | 20.536743 | -12.961179 | H6 |
| http://coldb.mnhn.fr/catalognumber/mnhn/zm/mo-1977-301 | Hyracoidea | Procaviidae | Procavia capensis | 20.536743 | -12.961179 | H6 |
| 302b5ee6-9f1e-4a77-b0ea-9db2d335106a | Hyracoidea | Procaviidae | Procavia capensis | 20.252804 | -13.088188 | H6 |
| 8427b1f3-ae42-40bf-8cc4-c2c60b277717 | Hyracoidea | Procaviidae | Procavia capensis | 16.641702 | -9.915257 | L9 |
| 48979bce-79bf-4db8-8563-5241fd280a18 | Hyracoidea | Procaviidae | Procavia capensis | 16.038447 | -10.500127 | M8 |
| b22b55a7-da2d-4ab3-9480-5b985f1e24fc | Hyracoidea | Procaviidae | Procavia capensis | 16.054682 | -10.537382 | M8 |
| 47a4a76d-b672-48df-a412-147dd04fcea9 | Hyracoidea | Procaviidae | Procavia capensis | 15.798355 | -10.805377 | M8 |
| b1c062c2-650a-4cad-bb7c-5df1eba9deb2 | Hyracoidea | Procaviidae | Procavia capensis | 18.400353 | -11.814568 | J7 |
| 9aaee7f6-352e-42f7-89dd-63c89dda35f5 | Hyracoidea | Procaviidae | Procavia capensis | 18.442815 | -11.387390 | J7 |
| e733d064-2a80-4170-9ddd-a059958c1728 | Hyracoidea | Procaviidae | Procavia capensis | 18.390480 | -8.554273 | J10 |
| ed780544-5dca-4d1f-b88c-d9dd64c2ea11 | Hyracoidea | Procaviidae | Procavia capensis | 18.190823 | -8.115063 | K11 |
| e78d6db1-8cf0-41b3-afed-4a6a1c3e8d83 | Hyracoidea | Procaviidae | Procavia capensis | 16.489543 | -11.057988 | M8 |
| 2755bd71-7841-454e-a650-486ca16ab618 | Hyracoidea | Procaviidae | Procavia capensis | 17.125240 | -12.094277 | L7 |
| beafeccf-419f-4adf-8f6d-0680972bc737 | Hyracoidea | Procaviidae | Procavia capensis | 16.002553 | -11.871748 | M7 |
| 34d943e9-f960-427b-85cf-d89207c1247d | Hyracoidea | Procaviidae | Procavia capensis | 16.002553 | -11.871748 | M7 |
| 2f65c127-e41c-4c4a-8e06-5d8217da84ce | Hyracoidea | Procaviidae | Procavia capensis | 20.933333 | -11.616667 | H7 |
| c4aecdc2-2801-4cc5-ba17-edab5458fc0a | Hyracoidea | Procaviidae | Procavia capensis | 16.579150 | -10.704550 | L8 |
| 6b0a090e-8ec3-4a62-a885-0d558aafcb23 | Hyracoidea | Procaviidae | Procavia capensis | 17.649985 | -11.395480 | K7 |
| 2b84d361-852b-41a0-84df-27a0bd034fd3 | Hyracoidea | Procaviidae | Procavia capensis | 17.863795 | -12.090195 | K7 |
| 15566324-2a25-404c-bca0-d4c6fba11a04 | Hyracoidea | Procaviidae | Procavia capensis | 17.846344 | -12.078243 | K7 |
| 996830ef-2e32-494f-99d4-e06879438c6c | Hyracoidea | Procaviidae | Procavia capensis | 20.504367 | -12.845628 | H6 |
| ecb51cb8-889e-4d0e-a23c-ea74d5fc5cce | Hyracoidea | Procaviidae | Procavia capensis | 17.152482 | -12.199115 | L7 |
| dd5ca66c-35af-4c44-835c-c36425bdb2c5 | Hyracoidea | Procaviidae | Procavia capensis | 15.957078 | -12.009859 | M7 |
| ab92eb16-7e9e-414d-8da6-562a46f81496 | Hyracoidea | Procaviidae | Procavia capensis | 17.737962 | -12.245253 | K7 |
| bc1e0c3b-2d58-4b6f-acd2-84a5fd9a34b7 | Hyracoidea | Procaviidae | Procavia capensis | 17.451667 | -12.394850 | K6 |
| 8406da41-8809-4096-84c9-1a5c9daf33ff | Hyracoidea | Procaviidae | Procavia capensis | 17.152482 | -12.199115 | L7 |
| 8423f5ac-17c2-4a34-a098-20db5b0b0f53 | Hyracoidea | Procaviidae | Procavia capensis | 17.088727 | -12.117248 | L7 |
| 7547e7f4-bdfd-4d6c-8dfd-4893150179fc | Hyracoidea | Procaviidae | Procavia capensis | 16.787060 | -11.920642 | L7 |
| 7bcb4c6b-a286-4cf0-96f9-07c0bb033a69 | Hyracoidea | Procaviidae | Procavia capensis | 17.232793 | -12.100005 | L7 |
| 7a45550c-44a7-453c-8006-0fb127b4b39e | Hyracoidea | Procaviidae | Procavia capensis | 17.240833 | -12.101667 | L7 |
| a5b00415-dbc7-443c-8e84-71f2e555f782 | Hyracoidea | Procaviidae | Procavia capensis | 17.571290 | -12.178565 | K7 |
| fe86fb9a-b0de-447b-b775-5729cb0adfa5 | Hyracoidea | Procaviidae | Procavia capensis | 18.386433 | -12.164971 | J7 |
| 96418e2e-504f-4c31-9356-ee5e78fd1e7d | Hyracoidea | Procaviidae | Procavia capensis | 17.846344 | -12.078243 | K7 |
| ef3597db-d523-4d49-bc5e-883286dde104 | Hyracoidea | Procaviidae | Procavia capensis | 17.662245 | -12.245037 | K7 |
| 50167837-8a15-4160-a401-e2ee084f1a7f | Hyracoidea | Procaviidae | Procavia capensis | 16.547455 | -12.009590 | L7 |
| c326549a-0812-4b86-9769-666539f42d94 | Hyracoidea | Procaviidae | Procavia capensis | 15.957078 | -12.009859 | M7 |
| c65d62f9-3179-4ea5-ad76-66948d84272a | Hyracoidea | Procaviidae | Procavia capensis | 15.932785 | -12.010887 | M7 |
| 24861fb0-21f9-4fe4-b4fc-cb8b632538a4 | Hyracoidea | Procaviidae | Procavia capensis | 16.579150 | -10.704550 | L8 |
| 532f32e4-203f-4fff-a8bc-88e38eb26a19 | Hyracoidea | Procaviidae | Procavia capensis | 16.687579 | -10.191363 | L9 |
| 1171b3fc-d7c0-4172-9f03-059ce88e901f | Hyracoidea | Procaviidae | Procavia capensis | 16.691103 | -9.716622 | L9 |
| b95c9cd7-dd5b-4c67-80e3-15d0db3fff68 | Hyracoidea | Procaviidae | Procavia capensis | 16.900395 | -10.178290 | L9 |
| 8cbc6d8f-fa51-444e-969c-b7a73bbd9d5b | Hyracoidea | Procaviidae | Procavia capensis | 17.649567 | -11.395433 | K7 |
| f9a387d7-ab33-42eb-812b-d5580ee09d01 | Hyracoidea | Procaviidae | Procavia capensis | 17.634802 | -11.414415 | K7 |
| b057e0aa-d9d8-4bf9-b4f8-1dd3302687ec | Hyracoidea | Procaviidae | Procavia capensis | 17.772547 | -11.538147 | K7 |
| f40d9a23-cb01-4337-b7da-f66571c5b324 | Hyracoidea | Procaviidae | Procavia capensis | 20.252804 | -13.088188 | H6 |
| 90d12af6-2a67-4185-bb24-81f02bd56dbc | Hyracoidea | Procaviidae | Procavia capensis | 16.538033 | -10.741550 | L8 |
| e0f44ba4-9677-4788-b815-8817c50e9f99 | Hyracoidea | Procaviidae | Procavia capensis | 20.323817 | -13.145967 | H6 |
| 62bb8e99-18df-4784-aaec-9f19d9dcdd0b | Hyracoidea | Procaviidae | Procavia capensis | 20.533017 | -12.817617 | H6 |
| 03f10e9e-6735-4058-9a69-5580cd163428 | Hyracoidea | Procaviidae | Procavia capensis | 17.649567 | -11.395433 | K7 |
| 8c5f71d6-45e8-43eb-8e03-5d13d404f813 | Hyracoidea | Procaviidae | Procavia capensis | 17.834850 | -11.557833 | K7 |
| 557ee74a-25bb-4fc6-847b-c01fb184cc8d | Hyracoidea | Procaviidae | Procavia capensis | 18.161183 | -11.574617 | K7 |
| 015e87e2-c696-427b-82ae-b2966914e1e0 | Hyracoidea | Procaviidae | Procavia capensis | 18.325933 | -11.539983 | J7 |
| 55412200-41b4-4c01-82be-8892a0fc55f6 | Hyracoidea | Procaviidae | Procavia capensis | 20.932200 | -11.622333 | H7 |
| 1470c6ff-d8f4-45f4-af3d-2270fc21eb60 | Hyracoidea | Procaviidae | Procavia capensis | 20.745217 | -13.134567 | H6 |
| de1d6f7e-576d-4b04-b4f4-ed23f166f4f7 | Hyracoidea | Procaviidae | Procavia capensis | 20.533017 | -12.817617 | H6 |
| 25f50782-37cb-4bc7-9131-1e5117a29823 | Hyracoidea | Procaviidae | Procavia capensis | 17.887298 | -12.110844 | K7 |
| 42dd6035-d084-49e9-8b40-c95fd13ebfd8 | Hyracoidea | Procaviidae | Procavia capensis | 17.451667 | -12.394850 | K6 |
| c5558213-1938-437e-af1d-4377bba0d1fe | Hyracoidea | Procaviidae | Procavia capensis | 17.279133 | -12.221450 | L7 |
| http://n2t.net/ark:/65665/3f80e7f16-1fe2-4123-bfab-c6c48786c4a1 | Lagomorpha | Leporidae | Lepus spp. | 17.266783 | -16.028417 | L3 |
| http://n2t.net/ark:/65665/3890873b3-bcd8-411f-9f49-85d8fbe36a6a | Lagomorpha | Leporidae | Lepus spp. | 17.266783 | -16.028417 | L3 |
| https://observation.org/observation/88883872 | Lagomorpha | Leporidae | Lepus spp. | 19.557000 | -15.997000 | I3 |
| http://n2t.net/ark:/65665/379108a8f-c128-43e6-a958-55f3fc4bb76b | Lagomorpha | Leporidae | Lepus spp. | 18.015000 | -15.910000 | K3 |
| http://n2t.net/ark:/65665/33046c072-6120-4979-93d3-01397e788da4 | Lagomorpha | Leporidae | Lepus spp. | 16.620000 | -11.400000 | L7 |
| http://n2t.net/ark:/65665/39f683872-7018-4200-aabe-7bbbde731c8a | Lagomorpha | Leporidae | Lepus spp. | 16.620000 | -11.400000 | L7 |
| http://n2t.net/ark:/65665/3dfbf28ae-a7cf-4a04-97da-82300cb806c3 | Lagomorpha | Leporidae | Lepus spp. | 16.550000 | -15.766667 | M3 |
| http://n2t.net/ark:/65665/3f884bc50-2200-468f-ab4d-6387f89aac37 | Lagomorpha | Leporidae | Lepus spp. | 16.550000 | -15.766667 | M3 |
| http://n2t.net/ark:/65665/33adf9944-4952-4ba2-9813-4d9ff78e105f | Lagomorpha | Leporidae | Lepus spp. | 16.650000 | -14.283333 | L4 |
| http://n2t.net/ark:/65665/3bb9a2bdf-b275-41ec-a0b3-3a01be1847f9 | Lagomorpha | Leporidae | Lepus spp. | 17.030000 | -13.920000 | L5 |
| http://n2t.net/ark:/65665/3744b0192-88a8-4a22-961f-991cb80e2dca | Lagomorpha | Leporidae | Lepus spp. | 19.750000 | -14.383333 | I4 |
| http://n2t.net/ark:/65665/340a4bf40-8579-4660-aa27-748243c2f787 | Lagomorpha | Leporidae | Lepus spp. | 19.750000 | -14.383333 | I4 |
| http://n2t.net/ark:/65665/36d30457b-17c2-4da6-b725-50862a104d99 | Lagomorpha | Leporidae | Lepus spp. | 19.750000 | -14.383333 | I4 |
| http://n2t.net/ark:/65665/3e999d781-16f3-44b3-b4ab-7e817b620d5c | Lagomorpha | Leporidae | Lepus spp. | 21.020000 | -13.150000 | H6 |
| ce94432d-e3c7-4555-a839-ebfc87718625 | Lagomorpha | Leporidae | Lepus spp. | 15.095670 | -12.243753 | N7 |
| 9e996d2e-25d6-4821-8cf9-7bce71bbedff | Lagomorpha | Leporidae | Lepus spp. | 16.275638 | -13.703690 | M5 |
| 9f6e9f48-0971-47d0-bd27-40879f136009 | Lagomorpha | Leporidae | Lepus spp. | 18.447222 | -10.683298 | J8 |
| 777635d0-c460-4b35-90b2-9b9a54b12002 | Lagomorpha | Leporidae | Lepus spp. | 18.206040 | -11.730977 | K7 |
| 618b3676-c055-4632-9434-8897608e6e30 | Lagomorpha | Leporidae | Lepus spp. | 18.356815 | -11.816080 | J7 |
| 1d518005-ea67-4969-a90e-db0aaec3b7f8 | Lagomorpha | Leporidae | Lepus spp. | 18.447222 | -10.683298 | J8 |
| 77e65922-d9c2-4f58-ba84-09e9f62bc839 | Lagomorpha | Leporidae | Lepus spp. | 20.991860 | -11.502300 | H7 |
| 0fa7d6d8-7a8f-4e23-a8be-dfe820399b30 | Lagomorpha | Leporidae | Lepus spp. | 20.180835 | -16.147485 | I2 |
| 47b759be-49aa-48cb-84b7-9a3218405f44 | Lagomorpha | Leporidae | Lepus spp. | 20.532340 | -12.767240 | H6 |
| c0a599b0-8b17-452a-9438-68f7daf8b0c0 | Lagomorpha | Leporidae | Lepus spp. | 22.702950 | -12.784913 | F6 |
| c61430c6-0732-4037-831a-08b8c86d0ea4 | Lagomorpha | Leporidae | Lepus spp. | 20.748160 | -16.076502 | H3 |
| 9de61b4d-ac01-413e-abd4-9dbee5fe7503 | Lagomorpha | Leporidae | Lepus spp. | 19.248218 | -15.633512 | J3 |
| e57eb560-6ae2-4d7f-82fc-faa3fa6c3e40 | Lagomorpha | Leporidae | Lepus spp. | 17.674501 | -14.861960 | K4 |
| 80012942-887f-4631-b842-21bd862a114b | Lagomorpha | Leporidae | Lepus spp. | 19.656430 | -16.188847 | I2 |
| 61ca7b24-facc-43a3-bdfa-0075f6dd3d4e | Lagomorpha | Leporidae | Lepus spp. | 17.462775 | -11.455713 | K7 |
| 8cb40a57-6c1e-483d-8268-c54d7d6e4884 | Lagomorpha | Leporidae | Lepus spp. | 21.008583 | -12.269820 | H6 |
| b8072803-3f65-4c41-9098-dee40918f742 | Lagomorpha | Leporidae | Lepus spp. | 20.473600 | -13.794660 | H5 |
| 763d1207-d1b4-4eae-b53c-65a145e9ce37 | Lagomorpha | Leporidae | Lepus spp. | 20.208614 | -14.273922 | H4 |
| a7e67fb5-f309-4b29-bf2a-0c0eaada5a9b | Lagomorpha | Leporidae | Lepus spp. | 19.743830 | -15.965797 | I3 |
| 196a32ed-22da-4e44-b33b-e47696159d45 | Lagomorpha | Leporidae | Lepus spp. | 16.864255 | -9.583395 | L9 |
| 990d2e86-958d-40bd-832f-edd00725e864 | Lagomorpha | Leporidae | Lepus spp. | 17.177762 | -10.538402 | L8 |
| cafb7d0c-282a-4581-b73e-6fc5a193a1a6 | Lagomorpha | Leporidae | Lepus spp. | 21.193660 | -14.123190 | G5 |
| dde57ee9-ba8f-453e-9681-7a6d84a80efe | Lagomorpha | Leporidae | Lepus spp. | 15.907400 | -11.956550 | M7 |
| 1f66d596-11ea-4110-8be3-57fd30018931 | Lagomorpha | Leporidae | Lepus spp. | 21.183333 | -13.750000 | G5 |
| 95120e61-e844-49b7-8017-d93c8412cb7c | Lagomorpha | Leporidae | Lepus spp. | 20.062450 | -13.808700 | I5 |
| b9d313bb-5468-45a1-896b-1344156b3114 | Lagomorpha | Leporidae | Lepus spp. | 17.918250 | -12.214600 | K7 |
| 54e40fa1-822b-4f73-b545-2eba08a34e82 | Lagomorpha | Leporidae | Lepus spp. | 16.386467 | -10.297083 | M9 |
| 8c4e9996-23fb-4160-a65f-334ae9f8e2b6 | Lagomorpha | Leporidae | Lepus spp. | 17.156883 | -7.272200 | L12 |
| e23a2da5-57ac-4c02-bad5-d16a8f03c8aa | Lagomorpha | Leporidae | Lepus spp. | 17.259217 | -7.067800 | L12 |
| daecfa10-361d-4c4e-8c81-95ae0724055a | Lagomorpha | Leporidae | Lepus spp. | 17.560050 | -7.457550 | K12 |
| 0cf14f8c-abf3-477f-8a1c-851301195f7d | Lagomorpha | Leporidae | Lepus spp. | 18.132617 | -8.071000 | K11 |
| b955410f-ac1b-4daa-94a8-20f54ded9fb3 | Lagomorpha | Leporidae | Lepus spp. | 18.358783 | -11.144200 | J8 |
| http://n2t.net/ark:/65665/35b30e7d3-44be-4b6b-8edc-4bbb848452d6 | Primates | Cercopithecidae | Chlorocebus sabaeus | 16.550000 | -15.766667 | M3 |
| 09c01345-1ebd-4247-bb2c-c9f5e8156d13 | Primates | Cercopithecidae | Chlorocebus sabaeus | 16.756482 | -11.997233 | L7 |
| https://observation.org/observation/114320840 | Primates | Cercopithecidae | Erythrocebus patas | 16.557510 | -15.886326 | M3 |
| https://observation.org/observation/114320773 | Primates | Cercopithecidae | Erythrocebus patas | 16.573969 | -15.946625 | M3 |
| https://observation.org/observation/97340822 | Primates | Cercopithecidae | Erythrocebus patas | 16.571238 | -15.945923 | M3 |
| http://n2t.net/ark:/65665/3a54cc0d1-fc1e-464f-960a-fe4e49f96dac | Primates | Cercopithecidae | Erythrocebus patas | 15.933333 | -12.016667 | M7 |
| http://n2t.net/ark:/65665/3d6b07aa5-9c66-45cc-b726-9a50f5380f76 | Primates | Cercopithecidae | Erythrocebus patas | 15.933333 | -12.016667 | M7 |
| http://n2t.net/ark:/65665/373d275b1-6260-424c-ba22-aabd42aab720 | Primates | Cercopithecidae | Erythrocebus patas | 15.933333 | -12.016667 | M7 |
| http://n2t.net/ark:/65665/3575c8607-c7b0-4de2-aacc-0ab9f5f8197d | Primates | Cercopithecidae | Erythrocebus patas | 15.933333 | -12.016667 | M7 |
| 7963d88e-abd4-4331-9b26-241d7f2e56c7 | Primates | Cercopithecidae | Erythrocebus patas | 16.283965 | -16.422123 | M2 |
| e8260344-8856-4f59-97d6-bfb5ee28c74c | Primates | Cercopithecidae | Erythrocebus patas | 14.832540 | -12.351375 | N7 |
| 78158502-5fcc-4266-b18f-0071e2356034 | Primates | Cercopithecidae | Erythrocebus patas | 15.888167 | -13.255645 | M6 |
| 64a1d5d8-01ed-4480-a7d3-7886efbcd96f | Primates | Cercopithecidae | Erythrocebus patas | 16.784772 | -15.324220 | L3 |
| 9b170232-e50d-44cb-bfd4-513f36159d00 | Primates | Cercopithecidae | Erythrocebus patas | 16.811952 | -15.313472 | L3 |
| c7ebea29-38e1-4b5f-83d8-608306168067 | Primates | Cercopithecidae | Erythrocebus patas | 16.282053 | -16.409228 | M2 |
| 3a603375-d59f-43e5-94a2-27ab81438416 | Primates | Cercopithecidae | Erythrocebus patas | 16.476298 | -11.070020 | M8 |
| a3423d08-9640-4882-bbcb-79d6abb82711 | Primates | Cercopithecidae | Erythrocebus patas | 15.052628 | -12.567617 | N6 |
| c24f9839-81fb-446e-b604-055360f5d8f5 | Primates | Cercopithecidae | Erythrocebus patas | 15.975558 | -11.871912 | M7 |
| 22647bcc-68dd-44fc-8ad1-ceb3143356f8 | Primates | Cercopithecidae | Erythrocebus patas | 15.052628 | -12.567617 | N6 |
| aa87e408-3102-4252-824b-c6ea237696e1 | Primates | Cercopithecidae | Erythrocebus patas | 15.052628 | -12.567617 | N6 |
| b41a33ef-c663-4a70-8302-edb781f7316b | Primates | Cercopithecidae | Erythrocebus patas | 15.052628 | -12.567617 | N6 |
| 23f768ca-06b6-441d-af3e-e79dbe7e0a01 | Primates | Cercopithecidae | Erythrocebus patas | 15.052628 | -12.567617 | N6 |
| 99bd4bfd-b23a-4b32-8b03-3fc549f06cfa | Primates | Cercopithecidae | Erythrocebus patas | 15.490503 | -12.942663 | N6 |
| 077c80f4-6355-4190-acf8-802b09d9ca00 | Primates | Cercopithecidae | Erythrocebus patas | 15.260320 | -12.835785 | N6 |
| 69f15ad2-2589-4f36-ad7d-45fef196e2e3 | Primates | Cercopithecidae | Erythrocebus patas | 17.887298 | -12.110844 | K7 |
| 1932c62b-c371-498b-9360-4bfb445f370d | Primates | Cercopithecidae | Erythrocebus patas | 16.888725 | -12.184868 | L7 |
| 0ce077c7-1176-4940-b628-c6891d3372bb | Primates | Cercopithecidae | Erythrocebus patas | 15.878200 | -12.039233 | M7 |
| b1d91f21-a901-47a5-8f00-1cc8cf3e3769 | Primates | Cercopithecidae | Erythrocebus patas | 15.901033 | -11.834100 | M7 |
| https://observation.org/observation/146309107 | Primates | Cercopithecidae | Papio papio | 17.887020 | -12.097993 | K7 |
| http://n2t.net/ark:/65665/33314de74-9d4a-4bb1-a69f-58df8461d7a8 | Primates | Cercopithecidae | Papio papio | 15.933333 | -12.016667 | M7 |
| http://n2t.net/ark:/65665/38ce5755a-749c-4e70-bd23-21e692ef9bec | Primates | Cercopithecidae | Papio papio | 15.933333 | -12.016667 | M7 |
| http://n2t.net/ark:/65665/388d3737d-7a31-40a0-842e-b939c651d63b | Primates | Cercopithecidae | Papio papio | 15.933333 | -12.016667 | M7 |
| 8335224f-dbd0-4e88-a0e0-fdfae5c287d7 | Primates | Cercopithecidae | Papio papio | 17.070297 | -12.207848 | L7 |
| 87252012-e53a-48fa-9790-1b2c2777500b | Primates | Cercopithecidae | Papio papio | 17.067572 | -12.260290 | L7 |
| 22dbd66f-81ae-4893-a512-3d37dd6b5618 | Primates | Cercopithecidae | Papio papio | 17.240833 | -12.101667 | L7 |
| debdfcc2-f911-442f-b73e-fecabac8e2bc | Primates | Cercopithecidae | Papio papio | 17.240833 | -12.101667 | L7 |
| e1225af4-43b6-4ff0-8f28-30b96b2139ee | Primates | Cercopithecidae | Papio papio | 15.944687 | -11.929082 | M7 |
| 4a5ab2e9-10cf-40ce-8525-69b2a91905e6 | Primates | Cercopithecidae | Papio papio | 16.543543 | -9.956927 | L9 |
| f6da56fc-1773-4850-bf27-d99ce060a2da | Primates | Cercopithecidae | Papio papio | 16.060007 | -10.426330 | M8 |
| 963bf3d5-400f-43cd-8a33-415afb8c677f | Primates | Cercopithecidae | Papio papio | 16.038447 | -10.500127 | M8 |
| 3a46d51b-8088-4cad-80d6-8fbc6e034ce6 | Primates | Cercopithecidae | Papio papio | 16.054682 | -10.537382 | M8 |
| d4346b54-6f3b-4f10-914b-c6966445cc5b | Primates | Cercopithecidae | Papio papio | 16.054682 | -10.537382 | M8 |
| 55d30ae0-51c9-4f4d-81aa-fff767d04bc6 | Primates | Cercopithecidae | Papio papio | 16.054682 | -10.537382 | M8 |
| ac166e0a-be2e-4b21-a808-522c27ace1c7 | Primates | Cercopithecidae | Papio papio | 15.855740 | -10.717803 | M8 |
| 65f24252-5b03-4c6c-8380-c38c044a5338 | Primates | Cercopithecidae | Papio papio | 15.806617 | -10.771530 | M8 |
| 6c4a176e-0c8e-4e3d-b3fb-745673d4452d | Primates | Cercopithecidae | Papio papio | 15.800107 | -10.775588 | M8 |
| 3b97c980-0993-4565-9c27-375a39961add | Primates | Cercopithecidae | Papio papio | 15.798355 | -10.805377 | M8 |
| 9fae1b02-a278-4899-8a27-29453dcf5aa6 | Primates | Cercopithecidae | Papio papio | 15.798355 | -10.805377 | M8 |
| 7b21a81e-06d9-4ea0-b28c-d08e2ee20b35 | Primates | Cercopithecidae | Papio papio | 15.798355 | -10.805377 | M8 |
| e73a4bbe-3681-4ecc-8db7-c7c5e550b9dd | Primates | Cercopithecidae | Papio papio | 15.798355 | -10.805377 | M8 |
| ab2d034c-9165-44c1-a6f8-be0b4ea9fad9 | Primates | Cercopithecidae | Papio papio | 15.674163 | -10.837277 | M8 |
| a402a266-75d5-435b-aa6f-5fd2c0da0713 | Primates | Cercopithecidae | Papio papio | 15.563663 | -10.982040 | N8 |
| 95fc6336-1679-480c-a73b-612735e9e1dd | Primates | Cercopithecidae | Papio papio | 15.563663 | -10.982040 | N8 |
| 23048a5a-ebeb-4002-9eca-b543f2ab9b92 | Primates | Cercopithecidae | Papio papio | 15.551130 | -11.087958 | N8 |
| 3dff2079-e1a3-4d0e-830a-de87d9803b81 | Primates | Cercopithecidae | Papio papio | 15.551130 | -11.087958 | N8 |
| 0bf66025-5131-4641-964b-acca3d08de3a | Primates | Cercopithecidae | Papio papio | 15.551130 | -11.087958 | N8 |
| b1b2f537-7d06-4a6e-be15-4d7b542b3410 | Primates | Cercopithecidae | Papio papio | 15.551130 | -11.087958 | N8 |
| 8dd2b749-cdc9-42ac-aac4-2148f3ca0500 | Primates | Cercopithecidae | Papio papio | 15.694747 | -11.237802 | M8 |
| 37d8fd7a-d150-4e89-a765-d4acb392b301 | Primates | Cercopithecidae | Papio papio | 15.694747 | -11.237802 | M8 |
| c44bb336-1ba4-4d9a-abe4-75635305f094 | Primates | Cercopithecidae | Papio papio | 15.694747 | -11.237802 | M8 |
| cd37bea6-2bee-43c9-ab35-42c294bcf18b | Primates | Cercopithecidae | Papio papio | 15.694747 | -11.237802 | M8 |
| 8587c653-073a-4bcb-ab89-fcdc6f337ea2 | Primates | Cercopithecidae | Papio papio | 15.694747 | -11.237802 | M8 |
| 70f469d0-e33f-4fd9-8500-d0a1cf3cac13 | Primates | Cercopithecidae | Papio papio | 15.694747 | -11.237802 | M8 |
| c29710fe-d03e-4b7f-8c60-09ff45c3625d | Primates | Cercopithecidae | Papio papio | 15.694747 | -11.237802 | M8 |
| 48d78781-e6d6-464f-b642-5f4ba16c8a2e | Primates | Cercopithecidae | Papio papio | 15.694747 | -11.237802 | M8 |
| 3dd69950-eda8-4a46-b21c-b0429f438d6f | Primates | Cercopithecidae | Papio papio | 15.694747 | -11.237802 | M8 |
| df6337b5-c0d2-4468-a652-361ff5cc853e | Primates | Cercopithecidae | Papio papio | 15.694747 | -11.237802 | M8 |
| 68e6307c-3d5c-45a1-8bdd-cf931c68b351 | Primates | Cercopithecidae | Papio papio | 15.694747 | -11.237802 | M8 |
| 0cb5882c-9a9e-448f-8149-657dfca8b81a | Primates | Cercopithecidae | Papio papio | 16.489543 | -11.057988 | M8 |
| 9c10fc13-def9-4442-972d-aa3a9a93ab32 | Primates | Cercopithecidae | Papio papio | 16.489543 | -11.057988 | M8 |
| b9874efe-1231-476b-b7c2-121ee1bf9e39 | Primates | Cercopithecidae | Papio papio | 16.489543 | -11.057988 | M8 |
| 22ee5df8-d57c-4e2c-a064-827fe303ef89 | Primates | Cercopithecidae | Papio papio | 16.489543 | -11.057988 | M8 |
| b1c27ee0-e3bd-4c14-9bb7-1397f54b9da3 | Primates | Cercopithecidae | Papio papio | 16.422665 | -11.007265 | M8 |
| 1b1f809c-7b9f-43ac-92a4-afb6620c4c0a | Primates | Cercopithecidae | Papio papio | 16.422665 | -11.007265 | M8 |
| 92be8511-54e4-401a-8cb1-255abac85b3e | Primates | Cercopithecidae | Papio papio | 16.422665 | -11.007265 | M8 |
| d2dab328-f576-40b4-8db0-41b70d02b455 | Primates | Cercopithecidae | Papio papio | 16.422665 | -11.007265 | M8 |
| 107a4691-e62d-4835-a689-ea7b281fed36 | Primates | Cercopithecidae | Papio papio | 16.422665 | -11.007265 | M8 |
| 9cf8fe1a-8c94-4a96-b14e-14ac68d3f3b6 | Primates | Cercopithecidae | Papio papio | 16.422665 | -11.007265 | M8 |
| 562b2130-41f0-4e6a-bcd3-95f56dfd9c06 | Primates | Cercopithecidae | Papio papio | 16.422665 | -11.007265 | M8 |
| 28645229-ffb4-4b5f-b6d6-e7473c7cc754 | Primates | Cercopithecidae | Papio papio | 16.422665 | -11.007265 | M8 |
| 9b8a3404-22c9-4d3b-9cc1-7264fe673bbb | Primates | Cercopithecidae | Papio papio | 16.422665 | -11.007265 | M8 |
| 72ecb8e2-963c-4510-a639-941e6237624e | Primates | Cercopithecidae | Papio papio | 16.422665 | -11.007265 | M8 |
| f6995f1c-fa72-4a4d-9d88-bb97ed44278f | Primates | Cercopithecidae | Papio papio | 16.422665 | -11.007265 | M8 |
| 8e458c47-818c-490c-a772-f92726b20069 | Primates | Cercopithecidae | Papio papio | 16.422665 | -11.007265 | M8 |
| 2ea0507b-206d-47d9-86e3-8d8ef2f3a07f | Primates | Cercopithecidae | Papio papio | 16.422665 | -11.007265 | M8 |
| 0d34680b-0378-4363-961a-1405fd222f33 | Primates | Cercopithecidae | Papio papio | 16.422665 | -11.007265 | M8 |
| 715a74ef-4635-4d39-8fdf-bce8127fc7d0 | Primates | Cercopithecidae | Papio papio | 16.422665 | -11.007265 | M8 |
| e8cfcade-bba3-4c26-a9cc-bd71bc91e179 | Primates | Cercopithecidae | Papio papio | 16.422665 | -11.007265 | M8 |
| f020886c-882d-4022-ae81-4b98415fe26a | Primates | Cercopithecidae | Papio papio | 16.254623 | -11.000642 | M8 |
| 5557d4e7-7bc2-4979-b288-8159f3a67dbd | Primates | Cercopithecidae | Papio papio | 16.254623 | -11.000642 | M8 |
| e9389e7e-8281-4dbc-bde8-06c42d2f7342 | Primates | Cercopithecidae | Papio papio | 16.618758 | -11.846003 | L7 |
| 44feb3df-48ba-4e1f-9ffa-31baff17b336 | Primates | Cercopithecidae | Papio papio | 16.530060 | -11.806630 | M7 |
| 8d4a3e73-acb1-4e82-8522-d2ce5b34596f | Primates | Cercopithecidae | Papio papio | 15.944687 | -11.929082 | M7 |
| 1fd71840-f57f-4912-b157-b9809e9dd3ed | Primates | Cercopithecidae | Papio papio | 15.975558 | -11.871912 | M7 |
| 29d41e91-2ae3-42d7-bb8e-41504dd50a51 | Primates | Cercopithecidae | Papio papio | 16.002553 | -11.871748 | M7 |
| 8609e885-3529-4724-8bed-d4600dd71c2c | Primates | Cercopithecidae | Papio papio | 16.002553 | -11.871748 | M7 |
| f23a507d-5dca-4c3b-8d49-575ad2c01e95 | Primates | Cercopithecidae | Papio papio | 16.002553 | -11.871748 | M7 |
| 9fba137b-508d-4042-953c-8f18d4f9615c | Primates | Cercopithecidae | Papio papio | 16.338801 | -11.978097 | M7 |
| 3bb4cf2f-718e-4cf7-85c9-e870ddb3f01f | Primates | Cercopithecidae | Papio papio | 16.338801 | -11.978097 | M7 |
| f6aec3b5-468f-46c3-b5a9-ee6758bea3dd | Primates | Cercopithecidae | Papio papio | 16.338801 | -11.978097 | M7 |
| 22271b9f-3c29-4251-b006-4c793b4a9c9b | Primates | Cercopithecidae | Papio papio | 16.338801 | -11.978097 | M7 |
| bbb53855-c9c5-425c-b711-289381834ffe | Primates | Cercopithecidae | Papio papio | 15.957078 | -12.009859 | M7 |
| 68d006a9-6a31-42db-a7ee-c33038fc200a | Primates | Cercopithecidae | Papio papio | 15.957078 | -12.009859 | M7 |
| 65931d05-c753-435a-873d-9f6db0b85415 | Primates | Cercopithecidae | Papio papio | 15.957078 | -12.009859 | M7 |
| f9a4ecdd-b654-4ac0-8f07-9d9a128702e4 | Primates | Cercopithecidae | Papio papio | 15.957078 | -12.009859 | M7 |
| 4beef0e4-d2c5-4e6f-ad49-c126ad083fa1 | Primates | Cercopithecidae | Papio papio | 15.957078 | -12.009859 | M7 |
| 15804817-3e0d-441e-86ce-53b57f897117 | Primates | Cercopithecidae | Papio papio | 15.058383 | -12.557562 | N6 |
| 66518b71-2e9a-49e9-a1ab-178b3c39caf8 | Primates | Cercopithecidae | Papio papio | 15.058383 | -12.557562 | N6 |
| 6ec7dc0c-4dae-4066-8f94-543b7388982b | Primates | Cercopithecidae | Papio papio | 15.058383 | -12.557562 | N6 |
| 8e6906ea-8c5c-4d24-870a-205a184a9a9b | Primates | Cercopithecidae | Papio papio | 15.058383 | -12.557562 | N6 |
| 21cb9b20-2b1e-4614-97de-9cfe342615ff | Primates | Cercopithecidae | Papio papio | 15.058383 | -12.557562 | N6 |
| 6db61450-3e65-409c-8874-c43886c9aa1e | Primates | Cercopithecidae | Papio papio | 15.058383 | -12.557562 | N6 |
| 5ef580f3-1cab-485c-ad2a-ccd141641af1 | Primates | Cercopithecidae | Papio papio | 15.058383 | -12.557562 | N6 |
| e29401fe-d796-46bd-8f7f-1d90abec69a6 | Primates | Cercopithecidae | Papio papio | 15.052628 | -12.567617 | N6 |
| 13af6e6e-c072-45c1-9974-3532ad6dfaa7 | Primates | Cercopithecidae | Papio papio | 15.052628 | -12.567617 | N6 |
| a89c38a4-c1e5-4131-bd51-3080e59c84e5 | Primates | Cercopithecidae | Papio papio | 15.264440 | -12.827020 | N6 |
| 8e5cb858-4eb2-43dd-90eb-f1c12084257a | Primates | Cercopithecidae | Papio papio | 15.055633 | -12.554327 | N6 |
| a7ba28e0-9552-4255-a0b5-6de56fda7241 | Primates | Cercopithecidae | Papio papio | 15.039893 | -12.533115 | N6 |
| 7bff4f70-b479-4446-b2bd-f493270095bf | Primates | Cercopithecidae | Papio papio | 16.579150 | -10.704550 | L8 |
| 33be09b9-b8cd-4c2c-afe3-e5b0667dc8c3 | Primates | Cercopithecidae | Papio papio | 17.649985 | -11.395480 | K7 |
| 33370c3d-e173-4d3d-953b-4ab6d62991f4 | Primates | Cercopithecidae | Papio papio | 17.863795 | -12.090195 | K7 |
| a45f1d71-2a19-4072-93f0-73b6e627cb93 | Primates | Cercopithecidae | Papio papio | 17.152482 | -12.199115 | L7 |
| ad967399-21b7-4ceb-bd77-48c40c0261ee | Primates | Cercopithecidae | Papio papio | 16.547455 | -12.009590 | L7 |
| 15e5afb6-3c7e-46ff-97ed-1d43ffe4db36 | Primates | Cercopithecidae | Papio papio | 15.957078 | -12.009859 | M7 |
| 20a1e20e-8073-4037-a661-01ef6f99dc22 | Primates | Cercopithecidae | Papio papio | 17.451667 | -12.394850 | K6 |
| 1f4045ab-4697-4050-b275-811063b60240 | Primates | Cercopithecidae | Papio papio | 17.370975 | -12.314068 | L7 |
| 84b46736-8882-4065-ac9b-38652b655ae8 | Primates | Cercopithecidae | Papio papio | 17.152482 | -12.199115 | L7 |
| 8bd9e72d-cc3a-496b-9902-ac148eace1c3 | Primates | Cercopithecidae | Papio papio | 17.088727 | -12.117248 | L7 |
| 8e65d968-76ae-4e2d-ad06-d6a52d78bc35 | Primates | Cercopithecidae | Papio papio | 17.070297 | -12.207848 | L7 |
| a8c848a3-4739-4679-b0f5-02c3b30409c9 | Primates | Cercopithecidae | Papio papio | 17.042720 | -12.160860 | L7 |
| ea634492-f829-459e-a30b-05e7a5f0ca0f | Primates | Cercopithecidae | Papio papio | 16.820822 | -11.951678 | L7 |
| 0597dbc1-70f9-4106-849a-c91c528a3945 | Primates | Cercopithecidae | Papio papio | 16.756482 | -11.997233 | L7 |
| 684764db-1212-4a30-80ba-bb2c7bda0457 | Primates | Cercopithecidae | Papio papio | 16.798392 | -11.954551 | L7 |
| f326497b-4c14-44d9-8b02-4ade9a03ae8b | Primates | Cercopithecidae | Papio papio | 17.240833 | -12.101667 | L7 |
| 99848def-eaac-4485-9ef1-b6f562bb0629 | Primates | Cercopithecidae | Papio papio | 18.031400 | -12.169190 | K7 |
| cacbeb93-e381-44de-a719-ad32a8dcd60f | Primates | Cercopithecidae | Papio papio | 17.451667 | -12.394850 | K6 |
| dda3cd4b-db7e-4b09-8133-958689b14c4b | Primates | Cercopithecidae | Papio papio | 17.401433 | -12.364150 | L6 |
| d2dd1cb9-be95-427b-b7a8-8c8df9be2898 | Primates | Cercopithecidae | Papio papio | 17.101413 | -12.256712 | L7 |
| bd854f52-1192-4b5f-b336-a500860818f2 | Primates | Cercopithecidae | Papio papio | 16.888725 | -12.184868 | L7 |
| 2fa803b9-282d-4f2b-80da-acf951ddf303 | Primates | Cercopithecidae | Papio papio | 16.547455 | -12.009590 | L7 |
| 33686326-d8b3-40d4-b61d-f4722bb8294d | Primates | Cercopithecidae | Papio papio | 15.957078 | -12.009859 | M7 |
| fe0c927c-0e46-4c3c-8bc7-1e871e2c4c5c | Primates | Cercopithecidae | Papio papio | 16.538033 | -10.741550 | L8 |
| 072366a0-a7ed-4d42-aaf9-3ab825d767f4 | Primates | Cercopithecidae | Papio papio | 16.691103 | -9.716622 | L9 |
| 7cce6105-c4cc-45ce-8c50-d57125208a90 | Primates | Cercopithecidae | Papio papio | 16.900395 | -10.178290 | L9 |
| b2597988-398c-486b-95bf-73a740cd9524 | Primates | Cercopithecidae | Papio papio | 15.883250 | -12.036367 | M7 |
| 170bb61f-ab45-43bf-a21b-8b6251f90804 | Primates | Cercopithecidae | Papio papio | 15.877000 | -12.038233 | M7 |
| 1f0f6d7c-a530-4dc7-bb0f-5006baadff2e | Primates | Cercopithecidae | Papio papio | 17.887298 | -12.110844 | K7 |
| http://coldb.mnhn.fr/catalognumber/mnhn/zm/mo-1986-27 | Rodentia | Ctenodactylidae | Felovia vae | 18.552000 | -11.428000 | J7 |
| http://coldb.mnhn.fr/catalognumber/mnhn/zm/mo-1986-26 | Rodentia | Ctenodactylidae | Felovia vae | 18.552000 | -11.428000 | J7 |
| http://n2t.net/ark:/65665/3471cf0b3-bb50-44c1-b0a9-422508761ef2 | Rodentia | Ctenodactylidae | Felovia vae | 15.933333 | -12.016667 | M7 |
| http://n2t.net/ark:/65665/363919bbf-08b5-4a5b-853b-cd94463c1d4b | Rodentia | Ctenodactylidae | Felovia vae | 15.933333 | -12.016667 | M7 |
| http://n2t.net/ark:/65665/3209ab8ba-4d3e-48c0-98e9-50266547945a | Rodentia | Ctenodactylidae | Felovia vae | 15.933333 | -12.016667 | M7 |
| http://n2t.net/ark:/65665/3746cf3f2-e77e-40a5-b0be-2ee086e5c016 | Rodentia | Ctenodactylidae | Felovia vae | 15.933333 | -12.016667 | M7 |
| http://n2t.net/ark:/65665/33c520d9b-3b6b-4061-9cd5-04b39914f988 | Rodentia | Ctenodactylidae | Felovia vae | 15.933333 | -12.016667 | M7 |
| http://n2t.net/ark:/65665/362cd27eb-a4e5-4960-ae85-395c3b2b9203 | Rodentia | Ctenodactylidae | Felovia vae | 15.933333 | -12.016667 | M7 |
| http://n2t.net/ark:/65665/319b2a133-c457-4723-a22a-052bd7455cef | Rodentia | Ctenodactylidae | Felovia vae | 15.933333 | -12.016667 | M7 |
| http://n2t.net/ark:/65665/3c0f2127f-6b0a-4a53-84fe-9964956be9da | Rodentia | Ctenodactylidae | Felovia vae | 15.933333 | -12.016667 | M7 |
| http://n2t.net/ark:/65665/3c99a6f93-4ab4-43b6-b072-789118cdb549 | Rodentia | Ctenodactylidae | Felovia vae | 15.933333 | -12.016667 | M7 |
| http://n2t.net/ark:/65665/3eb3245ab-afc8-4275-80cc-d70aaf8fe20f | Rodentia | Ctenodactylidae | Felovia vae | 15.933333 | -12.016667 | M7 |
| http://n2t.net/ark:/65665/39b98c022-11a9-4bf4-a823-6d6b43b594f6 | Rodentia | Ctenodactylidae | Felovia vae | 15.933333 | -12.016667 | M7 |
| http://n2t.net/ark:/65665/31e88e527-2118-4e9e-9cba-81032e17d351 | Rodentia | Ctenodactylidae | Felovia vae | 15.933333 | -12.016667 | M7 |
| http://n2t.net/ark:/65665/35ed38d24-d9b6-4da0-b6f8-cddb99899fb5 | Rodentia | Ctenodactylidae | Felovia vae | 15.933333 | -12.016667 | M7 |
| http://n2t.net/ark:/65665/37d631b93-4723-4c38-93f1-d00c4090cd1d | Rodentia | Ctenodactylidae | Felovia vae | 15.933333 | -12.016667 | M7 |
| http://coldb.mnhn.fr/catalognumber/mnhn/zm/mo-2001-798 | Rodentia | Ctenodactylidae | Felovia vae | 17.643794 | -12.226694 | K7 |
| http://coldb.mnhn.fr/catalognumber/mnhn/zm/mo-2001-773 | Rodentia | Ctenodactylidae | Felovia vae | 17.737962 | -12.245253 | K7 |
| http://coldb.mnhn.fr/catalognumber/mnhn/zm/mo-2001-772 | Rodentia | Ctenodactylidae | Felovia vae | 17.737962 | -12.245253 | K7 |
| http://n2t.net/ark:/65665/30d8a4db4-244b-4870-a39a-3aa52d8483d6 | Rodentia | Ctenodactylidae | Felovia vae | 20.550000 | -12.500000 | H6 |
| http://n2t.net/ark:/65665/35710d274-f9ac-417d-9e3c-10eba8242942 | Rodentia | Ctenodactylidae | Felovia vae | 20.550000 | -12.500000 | H6 |
| http://n2t.net/ark:/65665/3fcb5c2e0-ffc0-44c6-b404-f591759800a5 | Rodentia | Ctenodactylidae | Felovia vae | 20.550000 | -12.500000 | H6 |
| http://n2t.net/ark:/65665/3a6ce4196-32d0-4e10-a8f4-ecfab13009ad | Rodentia | Ctenodactylidae | Felovia vae | 20.550000 | -12.500000 | H6 |
| http://n2t.net/ark:/65665/317d325e9-6c66-45de-97ad-4cbf895041ae | Rodentia | Ctenodactylidae | Felovia vae | 20.550000 | -12.500000 | H6 |
| http://n2t.net/ark:/65665/3ad24b788-7695-4bbf-9b43-4a0ca1c0ed7c | Rodentia | Ctenodactylidae | Felovia vae | 20.550000 | -12.500000 | H6 |
| http://n2t.net/ark:/65665/310c3d06d-6468-4665-bb47-e05da78008e2 | Rodentia | Ctenodactylidae | Felovia vae | 20.550000 | -12.500000 | H6 |
| http://n2t.net/ark:/65665/39e3dca7e-2eab-4f5c-b27d-c518d423d2d8 | Rodentia | Ctenodactylidae | Felovia vae | 20.550000 | -12.500000 | H6 |
| http://n2t.net/ark:/65665/3f8b92da1-6d7a-41bc-8187-633773114880 | Rodentia | Ctenodactylidae | Felovia vae | 20.550000 | -12.500000 | H6 |
| http://n2t.net/ark:/65665/31066b391-ec2d-4711-b550-d40bbc7ca995 | Rodentia | Ctenodactylidae | Felovia vae | 21.525000 | -12.860600 | G6 |
| http://n2t.net/ark:/65665/33f3709b7-a87d-4ce9-b282-936a6b75f3f1 | Rodentia | Ctenodactylidae | Felovia vae | 21.525000 | -12.860600 | G6 |
| http://n2t.net/ark:/65665/3a1582fde-4485-4135-aa07-43f1e6019f89 | Rodentia | Ctenodactylidae | Felovia vae | 21.525000 | -12.860600 | G6 |
| http://n2t.net/ark:/65665/3cd8987bf-4263-4bf1-b03b-d8beef29dd73 | Rodentia | Ctenodactylidae | Felovia vae | 21.525000 | -12.860600 | G6 |
| http://n2t.net/ark:/65665/3c363915d-7834-43eb-9152-b048aba42b3e | Rodentia | Ctenodactylidae | Felovia vae | 21.525000 | -12.860600 | G6 |
| http://n2t.net/ark:/65665/370ae9812-968f-443a-aa31-c77abf44f650 | Rodentia | Ctenodactylidae | Felovia vae | 21.525000 | -12.860600 | G6 |
| http://n2t.net/ark:/65665/363348ebd-cd60-40fa-a33a-4a4c53146d41 | Rodentia | Ctenodactylidae | Felovia vae | 21.525000 | -12.860600 | G6 |
| http://n2t.net/ark:/65665/3f243a4d8-e0ae-4a4a-87b6-352433b3718f | Rodentia | Ctenodactylidae | Felovia vae | 21.525000 | -12.860600 | G6 |
| http://coldb.mnhn.fr/catalognumber/mnhn/zm/mo-1989-22 | Rodentia | Ctenodactylidae | Felovia vae | 20.536743 | -12.961179 | H6 |
| http://coldb.mnhn.fr/catalognumber/mnhn/zm/mo-1986-32 | Rodentia | Ctenodactylidae | Felovia vae | 20.536743 | -12.961179 | H6 |
| http://coldb.mnhn.fr/catalognumber/mnhn/zm/mo-1986-30 | Rodentia | Ctenodactylidae | Felovia vae | 20.536743 | -12.961179 | H6 |
| http://coldb.mnhn.fr/catalognumber/mnhn/zm/mo-1995-3157 | Rodentia | Ctenodactylidae | Felovia vae | 20.533017 | -12.817617 | H6 |
| 7756bae3-7250-42a0-a94a-c608a8c8169a | Rodentia | Ctenodactylidae | Felovia vae | 20.539789 | -12.775142 | H6 |
| a58fe008-cffe-440f-8ca3-06cddb56f5a7 | Rodentia | Ctenodactylidae | Felovia vae | 21.081347 | -11.968225 | G7 |
| 4dd3bb30-ba47-466b-adf8-1d835ff89305 | Rodentia | Ctenodactylidae | Felovia vae | 21.149875 | -11.963723 | G7 |
| 7245440b-f6b9-489b-8284-68c69880cfa4 | Rodentia | Ctenodactylidae | Felovia vae | 20.573301 | -12.538105 | H6 |
| ae87e0a8-96ca-477c-8899-3144b5a139b5 | Rodentia | Ctenodactylidae | Felovia vae | 20.573301 | -12.538105 | H6 |
| b32f94fe-0b10-4f42-b449-6f98abaad2a8 | Rodentia | Ctenodactylidae | Felovia vae | 20.880795 | -12.127885 | H7 |
| 41764507-fedc-4763-90c5-a06d06692237 | Rodentia | Ctenodactylidae | Felovia vae | 20.323135 | -13.144596 | H6 |
| fb22c887-ade2-4b12-8a74-e74d7d8b66a5 | Rodentia | Ctenodactylidae | Felovia vae | 19.623705 | -13.002998 | I6 |
| 41a06b91-1cd8-4c18-b504-60240b26192d | Rodentia | Ctenodactylidae | Felovia vae | 19.624224 | -13.006298 | I6 |
| b442eb76-6aba-479c-a2b5-2a838daf264a | Rodentia | Ctenodactylidae | Felovia vae | 19.997415 | -13.058335 | I6 |
| 79c198a4-9395-4dec-ac5e-23f89a512236 | Rodentia | Ctenodactylidae | Felovia vae | 20.249069 | -13.086801 | H6 |
| 03959f3c-5cdb-4fa9-a855-cb69dc7fe74d | Rodentia | Ctenodactylidae | Felovia vae | 20.272485 | -13.635308 | H5 |
| 72dd919d-15b7-4500-b306-7e75ec9bbcf9 | Rodentia | Ctenodactylidae | Felovia vae | 17.265836 | -12.299305 | L7 |
| 8a0f5d67-00bb-4f57-972b-e89ee0aaf002 | Rodentia | Ctenodactylidae | Felovia vae | 17.267485 | -12.297521 | L7 |
| 01fb39da-2eaf-468a-a4d7-28a8f15f56fe | Rodentia | Ctenodactylidae | Felovia vae | 17.065233 | -12.253716 | L7 |
| 8a7e9a86-926a-4a6c-b212-dae910b3f859 | Rodentia | Ctenodactylidae | Felovia vae | 17.066622 | -12.256892 | L7 |
| a2b8fe96-e13f-4139-ab1d-0c85dd708e78 | Rodentia | Ctenodactylidae | Felovia vae | 17.065275 | -12.257395 | L7 |
| 547491c9-5a80-4531-89df-f655f1184ef3 | Rodentia | Ctenodactylidae | Felovia vae | 16.979855 | -12.261583 | L7 |
| ab6c2aff-43c9-49ad-8a69-888d7d181bb8 | Rodentia | Ctenodactylidae | Felovia vae | 17.242377 | -12.102614 | L7 |
| d2eae27a-e416-4bc2-b576-a0f6edd27a87 | Rodentia | Ctenodactylidae | Felovia vae | 17.240186 | -12.102899 | L7 |
| 82f344ba-635e-486d-828b-494efd40359f | Rodentia | Ctenodactylidae | Felovia vae | 15.899397 | -11.834250 | M7 |
| d6930729-ce74-4b23-bf09-8e44c6dcde5a | Rodentia | Ctenodactylidae | Felovia vae | 15.903207 | -11.933737 | M7 |
| b13632b3-2135-48ea-bc64-7713693200db | Rodentia | Ctenodactylidae | Felovia vae | 15.900511 | -11.952344 | M7 |
| 955822f1-7104-4f73-99f6-b43ec7b6e753 | Rodentia | Ctenodactylidae | Felovia vae | 15.905381 | -11.923752 | M7 |
| 6c5aea26-1186-4141-990e-281f48ad7f3b | Rodentia | Ctenodactylidae | Felovia vae | 15.958895 | -12.106053 | M7 |
| f6e12ff0-7714-4cd7-8c36-b17232891f6c | Rodentia | Ctenodactylidae | Felovia vae | 16.483018 | -10.652837 | M8 |
| 4adb0b9a-9930-46b4-b870-e10646fdc357 | Rodentia | Ctenodactylidae | Felovia vae | 16.371455 | -10.245473 | M9 |
| dace80b4-fbbd-4f8c-aaa8-75ec586843b3 | Rodentia | Ctenodactylidae | Felovia vae | 16.687572 | -9.900575 | L9 |
| 8d0a81e1-4343-416a-a468-d62045c68ba4 | Rodentia | Ctenodactylidae | Felovia vae | 16.641702 | -9.915257 | L9 |
| 4cc51dda-7ee6-4ef6-83cd-67bf541563da | Rodentia | Ctenodactylidae | Felovia vae | 16.641702 | -9.915257 | L9 |
| cf22e0ec-b9b0-4210-a6ed-8f637dab677c | Rodentia | Ctenodactylidae | Felovia vae | 16.051477 | -10.539183 | M8 |
| 78c86cf4-a05d-4680-8a26-f08c7efad8fd | Rodentia | Ctenodactylidae | Felovia vae | 16.054682 | -10.537382 | M8 |
| 767843a1-fe34-4f90-851f-75b583965d4d | Rodentia | Ctenodactylidae | Felovia vae | 15.798355 | -10.805377 | M8 |
| 4977b888-df7e-4372-a3d6-f1102477e3bc | Rodentia | Ctenodactylidae | Felovia vae | 15.798355 | -10.805377 | M8 |
| 652a36ba-cec1-4849-bb8e-4af67e90f169 | Rodentia | Ctenodactylidae | Felovia vae | 15.563663 | -10.982040 | N8 |
| 0e8abae0-fa9f-40e7-ade3-6675cad7e07a | Rodentia | Ctenodactylidae | Felovia vae | 15.694747 | -11.237802 | M8 |
| 160e2a77-3ee5-4e20-b7ff-4704ad6aee00 | Rodentia | Ctenodactylidae | Felovia vae | 15.694747 | -11.237802 | M8 |
| 7ad5826d-f3b7-4a7b-91b1-2ddc6f79c64a | Rodentia | Ctenodactylidae | Felovia vae | 15.694747 | -11.237802 | M8 |
| 6254916a-d633-4c14-8e05-438f2cfe571c | Rodentia | Ctenodactylidae | Felovia vae | 18.150508 | -12.065716 | K7 |
| cb05876e-3b94-43aa-b8ef-0f16840fcfe2 | Rodentia | Ctenodactylidae | Felovia vae | 18.465202 | -11.735459 | J7 |
| f0e526dd-1ad3-42b9-875d-2179afc70022 | Rodentia | Ctenodactylidae | Felovia vae | 18.443365 | -11.383242 | J7 |
| 2f82b707-004a-4947-98e1-dc800466c803 | Rodentia | Ctenodactylidae | Felovia vae | 18.490216 | -11.380623 | J7 |
| 4462c53c-e182-4c84-a7cc-d42324a35433 | Rodentia | Ctenodactylidae | Felovia vae | 18.565729 | -11.189926 | J8 |
| f1222227-7e54-46b4-9d52-e9aa694dbcc9 | Rodentia | Ctenodactylidae | Felovia vae | 18.580834 | -9.814674 | J9 |
| 200186b2-2412-4945-bf56-aa620f509c84 | Rodentia | Ctenodactylidae | Felovia vae | 18.365060 | -9.050788 | J10 |
| 8aa16e1a-db8c-403f-bbfd-77db5108e0bf | Rodentia | Ctenodactylidae | Felovia vae | 18.390458 | -8.554212 | J10 |
| efcea466-8d4b-40e6-8176-63154900eb0e | Rodentia | Ctenodactylidae | Felovia vae | 16.490629 | -11.054444 | M8 |
| 96c46f2b-9050-4f54-864b-bc636c6a1fac | Rodentia | Ctenodactylidae | Felovia vae | 16.422594 | -11.007332 | M8 |
| c6d28ed9-a2eb-4e30-b0c6-1aa04c99e619 | Rodentia | Ctenodactylidae | Felovia vae | 18.150443 | -12.065303 | K7 |
| fd667066-4537-4e36-8b30-df42b75b06f3 | Rodentia | Ctenodactylidae | Felovia vae | 17.982002 | -11.944523 | K7 |
| a08bb515-9323-4123-b8de-ea374e7e1033 | Rodentia | Ctenodactylidae | Felovia vae | 18.206040 | -11.730977 | K7 |
| 836ab0b9-6524-4f32-88cf-a53be4d56da6 | Rodentia | Ctenodactylidae | Felovia vae | 18.400353 | -11.814568 | J7 |
| 0ec7a952-2d35-423b-88fd-9fbdad023217 | Rodentia | Ctenodactylidae | Felovia vae | 18.356815 | -11.816080 | J7 |
| a149857f-3506-496f-8281-4a02106868a6 | Rodentia | Ctenodactylidae | Felovia vae | 18.403052 | -11.793938 | J7 |
| e3380876-36ca-4e63-8e0b-218ad4d661cb | Rodentia | Ctenodactylidae | Felovia vae | 18.442815 | -11.387390 | J7 |
| 96ae5929-f653-4dcc-8408-ad5e07e3eb6a | Rodentia | Ctenodactylidae | Felovia vae | 18.563030 | -11.189008 | J8 |
| 499ef243-89d6-4978-8de8-d5d04f98ff74 | Rodentia | Ctenodactylidae | Felovia vae | 18.592830 | -10.026298 | J9 |
| a56cc244-65ab-4e86-9912-8da57885da98 | Rodentia | Ctenodactylidae | Felovia vae | 18.578522 | -9.818463 | J9 |
| 21df0215-57a0-4125-9611-c7d7ce8beb27 | Rodentia | Ctenodactylidae | Felovia vae | 18.406092 | -9.352792 | J10 |
| aec794ba-5717-4578-a9e0-6291aa395128 | Rodentia | Ctenodactylidae | Felovia vae | 18.347685 | -9.174887 | J10 |
| 4befe49d-d678-4d5a-8e96-ddc9d8b8f948 | Rodentia | Ctenodactylidae | Felovia vae | 18.367478 | -9.048450 | J10 |
| 46713fd1-24be-4f7f-8600-585637c4b7f3 | Rodentia | Ctenodactylidae | Felovia vae | 16.500160 | -9.564038 | M9 |
| f6d2d7a2-3bd5-4555-91a3-d290e419e0a6 | Rodentia | Ctenodactylidae | Felovia vae | 16.426157 | -9.565820 | M9 |
| a872ac83-9dc0-4108-980a-a473d5a54883 | Rodentia | Ctenodactylidae | Felovia vae | 16.489543 | -11.057988 | M8 |
| f96abce6-838a-4cf9-86f9-21842f56f77a | Rodentia | Ctenodactylidae | Felovia vae | 16.261892 | -11.053208 | M8 |
| 1c6b873a-d7f7-448b-8d4f-bf4364b48756 | Rodentia | Ctenodactylidae | Felovia vae | 17.132642 | -12.086563 | L7 |
| d0ad2d1d-e772-480c-a882-f440ed9e77f8 | Rodentia | Ctenodactylidae | Felovia vae | 17.127168 | -12.098365 | L7 |
| fe09cf29-538f-4d5c-a184-083f2743daeb | Rodentia | Ctenodactylidae | Felovia vae | 17.124779 | -12.096350 | L7 |
| 0a0afa9c-bfea-4872-b270-269f23d9def1 | Rodentia | Ctenodactylidae | Felovia vae | 17.126136 | -12.094662 | L7 |
| 7b14c8aa-ad4a-4945-b9ee-9295877e4af0 | Rodentia | Ctenodactylidae | Felovia vae | 17.133689 | -12.091085 | L7 |
| 899ec140-03fb-4e45-af47-2cf24af77d45 | Rodentia | Ctenodactylidae | Felovia vae | 17.131866 | -11.523173 | L7 |
| a2ed0e0b-b006-4e48-9b84-a9704baf957d | Rodentia | Ctenodactylidae | Felovia vae | 17.132375 | -11.522293 | L7 |
| c013ecad-80b4-4881-a2e8-a40bb0c988b6 | Rodentia | Ctenodactylidae | Felovia vae | 17.132401 | -11.522098 | L7 |
| acd4e34a-392d-4b7d-833a-889b05d3e284 | Rodentia | Ctenodactylidae | Felovia vae | 16.763786 | -11.267341 | L8 |
| 60f0272f-701c-42aa-8372-a47365faf7e1 | Rodentia | Ctenodactylidae | Felovia vae | 16.762403 | -11.222997 | L8 |
| 3d6c97fd-629a-4bc9-b26e-1bfae8e2cc67 | Rodentia | Ctenodactylidae | Felovia vae | 16.763011 | -11.222889 | L8 |
| 82abf0af-32ad-41e9-a293-a0f322c98f48 | Rodentia | Ctenodactylidae | Felovia vae | 16.777549 | -11.142154 | L8 |
| 62501976-9ed3-45d5-9c6c-451efb51bc90 | Rodentia | Ctenodactylidae | Felovia vae | 16.729615 | -11.126995 | L8 |
| 1a369a06-0ad7-4825-8136-6ec937084efd | Rodentia | Ctenodactylidae | Felovia vae | 16.728307 | -11.128073 | L8 |
| 5e909c42-b767-43c1-a6a0-d77fc107ed84 | Rodentia | Ctenodactylidae | Felovia vae | 16.727708 | -11.128915 | L8 |
| 5ef1719c-b31a-4e9c-a48f-76b14389d352 | Rodentia | Ctenodactylidae | Felovia vae | 16.729606 | -11.128882 | L8 |
| 891ca900-4149-4f79-a9f0-6a51a1811c3a | Rodentia | Ctenodactylidae | Felovia vae | 16.706244 | -11.206893 | L8 |
| 13ea1f5d-95ec-4835-82b2-08f3c85643af | Rodentia | Ctenodactylidae | Felovia vae | 16.706210 | -11.207678 | L8 |
| df3a0df1-ac37-4847-9e03-fe58818ae705 | Rodentia | Ctenodactylidae | Felovia vae | 16.684018 | -11.288953 | L8 |
| ebbd6c39-85a1-443c-8e14-5db812e8cdf3 | Rodentia | Ctenodactylidae | Felovia vae | 16.685026 | -11.290362 | L8 |
| 00c1aa04-86e1-498f-8798-ca175f71dee2 | Rodentia | Ctenodactylidae | Felovia vae | 16.684506 | -11.291142 | L8 |
| 1d6ea095-5c22-4048-b890-16384c218687 | Rodentia | Ctenodactylidae | Felovia vae | 16.620727 | -11.845384 | L7 |
| 904a053a-c24c-4b88-a38f-eaf56a08ed5c | Rodentia | Ctenodactylidae | Felovia vae | 16.619301 | -11.844630 | L7 |
| 749f98f0-41b5-4ddd-a78a-f86a20f11330 | Rodentia | Ctenodactylidae | Felovia vae | 16.528664 | -11.807104 | M7 |
| 9a1f18f8-aae9-432e-91cd-c1a1f85ee291 | Rodentia | Ctenodactylidae | Felovia vae | 16.530050 | -11.806838 | M7 |
| c58b7639-c388-4cfe-bf33-9e2548ce3d57 | Rodentia | Ctenodactylidae | Felovia vae | 16.565333 | -11.717181 | L7 |
| f29c8367-de13-427c-a5ba-2853e3bbd654 | Rodentia | Ctenodactylidae | Felovia vae | 16.563005 | -11.717466 | L7 |
| dfb691e9-1992-4c6e-9b56-52ad5733cf6f | Rodentia | Ctenodactylidae | Felovia vae | 16.446773 | -11.777352 | M7 |
| 88e77d6f-c90a-4f8d-aa65-246c91a3c58f | Rodentia | Ctenodactylidae | Felovia vae | 16.446329 | -11.780042 | M7 |
| 4d6c941e-9998-4319-ad7f-01a992deb067 | Rodentia | Ctenodactylidae | Felovia vae | 16.163265 | -11.749919 | M7 |
| b91e5a89-de8d-465d-9c6c-80afa18f52e2 | Rodentia | Ctenodactylidae | Felovia vae | 16.163580 | -11.749686 | M7 |
| 0f72d1f4-7f40-4e95-b7d8-6debbc75d13e | Rodentia | Ctenodactylidae | Felovia vae | 16.162581 | -11.748411 | M7 |
| 78acc982-3d74-410a-932a-c51ace9a2122 | Rodentia | Ctenodactylidae | Felovia vae | 16.049958 | -11.672609 | M7 |
| 61bb171c-74d4-436f-99a1-d6cb6f6694cf | Rodentia | Ctenodactylidae | Felovia vae | 16.049853 | -11.673102 | M7 |
| f56ef20b-9867-488b-9a03-69c58c43748a | Rodentia | Ctenodactylidae | Felovia vae | 16.050970 | -11.673324 | M7 |
| d5319275-dc11-46d9-89b8-c3e822373c80 | Rodentia | Ctenodactylidae | Felovia vae | 16.053725 | -11.671430 | M7 |
| 0c8bb017-e556-4377-bf28-5328563b6f8d | Rodentia | Ctenodactylidae | Felovia vae | 15.947265 | -11.931579 | M7 |
| d3a3cf1c-25fe-402d-8336-ebf0c72066dd | Rodentia | Ctenodactylidae | Felovia vae | 15.945109 | -11.929452 | M7 |
| 2953214a-64ba-418d-a989-9a502857cc77 | Rodentia | Ctenodactylidae | Felovia vae | 15.944030 | -11.928372 | M7 |
| bb0f5587-14ab-4646-a8ef-10e91b17688d | Rodentia | Ctenodactylidae | Felovia vae | 15.942668 | -11.929295 | M7 |
| 73e7a136-b483-4b92-acef-c881bb4cdf4b | Rodentia | Ctenodactylidae | Felovia vae | 15.942204 | -11.928270 | M7 |
| 00fe3196-4d27-49d0-83cf-bb4cd699325d | Rodentia | Ctenodactylidae | Felovia vae | 15.976762 | -11.871097 | M7 |
| 24947d5b-09de-43f2-95e3-5b55384b42a3 | Rodentia | Ctenodactylidae | Felovia vae | 15.975472 | -11.872637 | M7 |
| ff823dd6-d27b-4f2b-b02c-f45fc8eb4a30 | Rodentia | Ctenodactylidae | Felovia vae | 15.977727 | -11.873689 | M7 |
| b799bd35-58cd-433f-b0c9-3ea6128e7d18 | Rodentia | Ctenodactylidae | Felovia vae | 15.978284 | -11.873429 | M7 |
| bcf720bc-623e-4025-bac7-59eb10b5b85c | Rodentia | Ctenodactylidae | Felovia vae | 16.000872 | -11.872135 | M7 |
| 1c65d0b4-c518-4912-aa12-2a2deef43146 | Rodentia | Ctenodactylidae | Felovia vae | 15.997792 | -11.871048 | M7 |
| 0cb4a199-0c9f-4d43-b229-a2d8f5ed54e8 | Rodentia | Ctenodactylidae | Felovia vae | 16.002615 | -11.871674 | M7 |
| 56952d38-99d5-4ac1-a820-f31a6ea9c241 | Rodentia | Ctenodactylidae | Felovia vae | 16.000077 | -11.872309 | M7 |
| f58787f9-8b9a-47b3-a7ad-a10ecf547f42 | Rodentia | Ctenodactylidae | Felovia vae | 15.999080 | -11.872057 | M7 |
| 1d0bb2f3-4391-4be8-8444-5b67ecd37b9d | Rodentia | Ctenodactylidae | Felovia vae | 16.296318 | -12.004644 | M7 |
| fe535877-da53-46ee-9258-25084454cc22 | Rodentia | Ctenodactylidae | Felovia vae | 16.296344 | -12.003785 | M7 |
| 9cffcbb7-4137-4169-9f6a-f28edbb3db08 | Rodentia | Ctenodactylidae | Felovia vae | 16.332832 | -11.977050 | M7 |
| d521dc29-e6ee-4449-9f46-6ebc7fa873c4 | Rodentia | Ctenodactylidae | Felovia vae | 16.336243 | -11.976410 | M7 |
| 9c7a28cb-092c-4921-80a2-bb5cc7ed2a0a | Rodentia | Ctenodactylidae | Felovia vae | 16.336014 | -11.977018 | M7 |
| eebdbc80-f6c8-4d2c-b9ea-704d9abf71e0 | Rodentia | Ctenodactylidae | Felovia vae | 16.335484 | -11.978091 | M7 |
| 122a582b-a262-4469-82db-ec2ddc48d351 | Rodentia | Ctenodactylidae | Felovia vae | 16.334712 | -11.978495 | M7 |
| e2bda883-297e-409d-ac92-8881a2f1a9c7 | Rodentia | Ctenodactylidae | Felovia vae | 15.959458 | -12.006809 | M7 |
| 2a9a09e9-dfb5-4974-afdc-aaafdeb958fa | Rodentia | Ctenodactylidae | Felovia vae | 15.958979 | -12.008185 | M7 |
| e1c00c5b-3b01-4ba8-974d-fd6750808c40 | Rodentia | Ctenodactylidae | Felovia vae | 15.954452 | -12.009812 | M7 |
| 1eae014f-9410-4ba3-a015-af04f567491d | Rodentia | Ctenodactylidae | Felovia vae | 15.957151 | -12.010422 | M7 |
| c6d0db31-a38a-4751-96f4-2f927f5f10b3 | Rodentia | Ctenodactylidae | Felovia vae | 15.958278 | -12.010022 | M7 |
| 675d3a52-6e2b-4ce8-8057-40d9f5503f1d | Rodentia | Ctenodactylidae | Felovia vae | 17.135660 | -12.090422 | L7 |
| 456684f8-116b-4e77-b1a2-a4f9c218347b | Rodentia | Ctenodactylidae | Felovia vae | 17.125240 | -12.094277 | L7 |
| ba0c1bfe-ae39-4946-85ea-41b5084e6e20 | Rodentia | Ctenodactylidae | Felovia vae | 16.777603 | -11.143815 | L8 |
| 4b251809-91c3-4c79-94c0-7b34248a66db | Rodentia | Ctenodactylidae | Felovia vae | 16.729080 | -11.130938 | L8 |
| 47207f85-fabd-4c99-9a86-179d99202ca7 | Rodentia | Ctenodactylidae | Felovia vae | 16.684655 | -11.291553 | L8 |
| 7dc4974f-9ad8-4c07-9194-bd1105dd74ef | Rodentia | Ctenodactylidae | Felovia vae | 16.618758 | -11.846003 | L7 |
| 32565c65-0ca2-475e-9e8b-d390066b49d5 | Rodentia | Ctenodactylidae | Felovia vae | 16.530060 | -11.806630 | M7 |
| 78c78409-243e-4c1e-a1cc-080137881736 | Rodentia | Ctenodactylidae | Felovia vae | 15.944687 | -11.929082 | M7 |
| 3ab0660e-7774-4a2d-95f3-359098ef7efd | Rodentia | Ctenodactylidae | Felovia vae | 15.975558 | -11.871912 | M7 |
| c33e1031-936e-4e20-b24c-43b09acd9471 | Rodentia | Ctenodactylidae | Felovia vae | 16.002553 | -11.871748 | M7 |
| 3e1f0e11-4166-407d-993d-a006b95c720b | Rodentia | Ctenodactylidae | Felovia vae | 16.338801 | -11.978097 | M7 |
| 41826ddf-a12f-49e1-9373-27df1e23804f | Rodentia | Ctenodactylidae | Felovia vae | 18.053485 | -11.942891 | K7 |
| 4dcc6c4c-874b-4154-a780-2cfa8ce256e0 | Rodentia | Ctenodactylidae | Felovia vae | 17.881083 | -12.094000 | K7 |
| 9740eb9a-343e-4c0a-b21c-618b6cb53972 | Rodentia | Ctenodactylidae | Felovia vae | 17.887298 | -12.110844 | K7 |
| b9accea4-3804-4222-a288-10bed47c073d | Rodentia | Ctenodactylidae | Felovia vae | 17.887298 | -12.110844 | K7 |
| 536f7234-4c78-4675-b2b9-4689244029c3 | Rodentia | Ctenodactylidae | Felovia vae | 20.549787 | -12.690467 | H6 |
| b50c36c8-83d8-4f39-9863-93dfff67de05 | Rodentia | Ctenodactylidae | Felovia vae | 20.546783 | -12.689570 | H6 |
| ba05b01f-fd42-4f11-b2f7-26b08685eebe | Rodentia | Ctenodactylidae | Felovia vae | 17.649985 | -11.395480 | K7 |
| 61953a32-8e75-4648-ad8b-65d3357c0b78 | Rodentia | Ctenodactylidae | Felovia vae | 18.258250 | -11.513437 | K7 |
| 1a0cb5c7-612a-41b1-bb22-407115737bc2 | Rodentia | Ctenodactylidae | Felovia vae | 18.053485 | -11.942891 | K7 |
| 7a811837-b507-4fc2-b256-3e6e223dcde0 | Rodentia | Ctenodactylidae | Felovia vae | 17.863795 | -12.090195 | K7 |
| 6461a6a5-d38f-460c-8ba3-b0ddbfaae286 | Rodentia | Ctenodactylidae | Felovia vae | 20.554748 | -12.691650 | H6 |
| cd836275-a3e4-4278-a294-20bc06beb305 | Rodentia | Ctenodactylidae | Felovia vae | 21.526448 | -12.864365 | G6 |
| 8f12e08d-0857-4f3e-a820-df5a2fb6e801 | Rodentia | Ctenodactylidae | Felovia vae | 15.957078 | -12.009859 | M7 |
| 9602ba32-d432-4954-97bc-ae07dcd1a2e4 | Rodentia | Ctenodactylidae | Felovia vae | 15.957078 | -12.009859 | M7 |
| 0632b8ec-e822-442a-917a-19ea906eea15 | Rodentia | Ctenodactylidae | Felovia vae | 15.957078 | -12.009859 | M7 |
| 7c49255b-f5db-4c64-b31e-55bd53f18734 | Rodentia | Ctenodactylidae | Felovia vae | 15.957078 | -12.009859 | M7 |
| 003fe894-14be-418d-a749-cf9b5318866d | Rodentia | Ctenodactylidae | Felovia vae | 15.957078 | -12.009859 | M7 |
| 257166f4-39b4-4df4-9a9c-4df7a2c00b19 | Rodentia | Ctenodactylidae | Felovia vae | 17.152482 | -12.199115 | L7 |
| 34ac9900-535f-4b45-b44c-37440e937585 | Rodentia | Ctenodactylidae | Felovia vae | 17.067572 | -12.260290 | L7 |
| 4388c327-4bfb-43c7-9e5a-22cc094ea0d4 | Rodentia | Ctenodactylidae | Felovia vae | 16.547455 | -12.009590 | L7 |
| a68a64f3-88d5-478f-aa5c-3ab975aa4878 | Rodentia | Ctenodactylidae | Felovia vae | 16.547455 | -12.009590 | L7 |
| 7cc6b9e0-8a0a-4a32-a985-f12a07b95539 | Rodentia | Ctenodactylidae | Felovia vae | 15.908323 | -11.921210 | M7 |
| 06521236-9a32-432e-aa7c-a3012ffb6fad | Rodentia | Ctenodactylidae | Felovia vae | 16.292280 | -12.011320 | M7 |
| db8e0564-961a-439b-ac32-039be90aa78e | Rodentia | Ctenodactylidae | Felovia vae | 15.964750 | -11.915715 | M7 |
| ba1188ff-3440-4a6d-9d70-87f7733df456 | Rodentia | Ctenodactylidae | Felovia vae | 15.957078 | -12.009859 | M7 |
| a67b0a91-1720-440d-9728-b69b6e538ba1 | Rodentia | Ctenodactylidae | Felovia vae | 15.957078 | -12.009859 | M7 |
| 4f5969ba-cf0b-4db2-ad8b-a963fc2b4cd7 | Rodentia | Ctenodactylidae | Felovia vae | 17.737962 | -12.245253 | K7 |
| 5ce21c10-0e2e-4af9-b66f-d66b82e6abbc | Rodentia | Ctenodactylidae | Felovia vae | 17.370975 | -12.314068 | L7 |
| 2fa4ef82-9c82-4737-860f-6a70fa2f06e8 | Rodentia | Ctenodactylidae | Felovia vae | 17.152482 | -12.199115 | L7 |
| 94c7ec29-c24b-4e9d-bd6f-f0310bb28258 | Rodentia | Ctenodactylidae | Felovia vae | 17.088727 | -12.117248 | L7 |
| 3d1f5082-e618-4cf1-94cc-9b4ac12f83f9 | Rodentia | Ctenodactylidae | Felovia vae | 17.042720 | -12.160860 | L7 |
| 47bd8653-f5f9-4fd8-930d-eec6848309e6 | Rodentia | Ctenodactylidae | Felovia vae | 17.142555 | -11.885383 | L7 |
| f114d60e-29ba-4628-be8b-ce9d7b1714d8 | Rodentia | Ctenodactylidae | Felovia vae | 17.173397 | -11.938015 | L7 |
| bd47f581-514a-4313-a879-6a5c05bec6d3 | Rodentia | Ctenodactylidae | Felovia vae | 17.382798 | -11.720837 | L7 |
| b00c9c5d-c437-4fa3-a0c6-bf921840aab9 | Rodentia | Ctenodactylidae | Felovia vae | 17.372698 | -11.915772 | L7 |
| c4c41a3a-966f-4dcb-84d2-82ea05a5bac6 | Rodentia | Ctenodactylidae | Felovia vae | 17.333602 | -12.077818 | L7 |
| ebf543f0-4a7b-4f6d-86f4-40d48bbb2261 | Rodentia | Ctenodactylidae | Felovia vae | 17.275708 | -12.101020 | L7 |
| 02095d64-4bc9-4077-87a0-773bb93ea6b1 | Rodentia | Ctenodactylidae | Felovia vae | 17.232793 | -12.100005 | L7 |
| 464a1916-b8b8-4108-9aa3-9f8e08d5e502 | Rodentia | Ctenodactylidae | Felovia vae | 17.240833 | -12.101667 | L7 |
| ecbd583f-999a-4074-b4ac-1d92803f076d | Rodentia | Ctenodactylidae | Felovia vae | 17.361490 | -12.197167 | L7 |
| 62c64278-16ea-43b2-a35e-26cb8a1949f9 | Rodentia | Ctenodactylidae | Felovia vae | 17.432012 | -12.182428 | L7 |
| 9f6fb6f6-77b6-4715-b916-969c01ac5127 | Rodentia | Ctenodactylidae | Felovia vae | 17.478648 | -12.161713 | K7 |
| 059144d3-d861-41b1-92c8-e3bd1bd4f2df | Rodentia | Ctenodactylidae | Felovia vae | 17.571290 | -12.178565 | K7 |
| ed3bbe5e-7c40-4578-be48-bd9161fb60eb | Rodentia | Ctenodactylidae | Felovia vae | 17.863795 | -12.090195 | K7 |
| 94d660f7-7340-47a7-ab21-4073fa67d661 | Rodentia | Ctenodactylidae | Felovia vae | 18.186980 | -11.746348 | K7 |
| 72727c8c-e3a9-477e-9c27-33d0dd7ed6b0 | Rodentia | Ctenodactylidae | Felovia vae | 18.070578 | -12.333977 | K6 |
| 63753b0c-a695-4a6e-a925-6828691b3914 | Rodentia | Ctenodactylidae | Felovia vae | 18.386433 | -12.164971 | J7 |
| 9b967fab-6612-41c9-9480-8e27791665d7 | Rodentia | Ctenodactylidae | Felovia vae | 21.455093 | -11.308397 | G7 |
| fb30b816-5f41-43e5-a918-cdf941c9a1b6 | Rodentia | Ctenodactylidae | Felovia vae | 17.957738 | -12.319137 | K6 |
| 68e89925-a8ad-4e89-a2ae-3d3d08bfc617 | Rodentia | Ctenodactylidae | Felovia vae | 17.662245 | -12.245037 | K7 |
| 08abc52a-ca95-4a0f-9037-08463d3e8674 | Rodentia | Ctenodactylidae | Felovia vae | 17.737962 | -12.245253 | K7 |
| 3a483e9f-afab-46eb-ba0d-c6a5c91394d7 | Rodentia | Ctenodactylidae | Felovia vae | 17.821264 | -12.185762 | K7 |
| bbbd227b-802f-4fef-828d-260d9a97d4d4 | Rodentia | Ctenodactylidae | Felovia vae | 17.451667 | -12.394850 | K6 |
| 7722abdf-070c-4c7b-9b3d-06ef66e5e29f | Rodentia | Ctenodactylidae | Felovia vae | 17.101413 | -12.256712 | L7 |
| a916644a-1088-48be-8972-2a7adc07b845 | Rodentia | Ctenodactylidae | Felovia vae | 16.184838 | -12.012062 | M7 |
| 2918c957-809a-4f40-9ab7-e8989b6b80e0 | Rodentia | Ctenodactylidae | Felovia vae | 15.932785 | -12.010887 | M7 |
| 313a2301-258d-4372-a313-fb7ba9f8b522 | Rodentia | Ctenodactylidae | Felovia vae | 16.579150 | -10.704550 | L8 |
| 49e01ab9-ae01-44d6-b533-245fc933e321 | Rodentia | Ctenodactylidae | Felovia vae | 16.687579 | -10.191363 | L9 |
| 94cf1bea-c7e7-4d89-8647-d5ea5cb4a0e3 | Rodentia | Ctenodactylidae | Felovia vae | 16.900395 | -10.178290 | L9 |
| 6a02e711-ef91-4a57-b44b-8b1be22f3fb4 | Rodentia | Ctenodactylidae | Felovia vae | 17.649567 | -11.395433 | K7 |
| a2ca26ee-1b3e-4021-890d-5f85ad07238d | Rodentia | Ctenodactylidae | Felovia vae | 17.634802 | -11.414415 | K7 |
| ce564281-b583-488e-acbd-52808b9eb7a3 | Rodentia | Ctenodactylidae | Felovia vae | 18.260307 | -11.523105 | K7 |
| b7a890c9-5599-4f78-8e66-85111caae067 | Rodentia | Ctenodactylidae | Felovia vae | 15.883250 | -12.036367 | M7 |
| ba501d53-60b4-4da0-bdcf-a6d0d3f3511e | Rodentia | Ctenodactylidae | Felovia vae | 17.673433 | -11.484867 | K7 |
| 049097cf-e26f-4007-b952-f3a8641c6302 | Rodentia | Ctenodactylidae | Felovia vae | 18.481250 | -11.382950 | J7 |
| 4f09cc44-46e0-4037-80fa-f9f85918b2c3 | Rodentia | Ctenodactylidae | Felovia vae | 18.875833 | -11.818333 | J7 |
| e74c649a-1b62-4179-b77c-a561ab5a29cf | Rodentia | Ctenodactylidae | Felovia vae | 20.068800 | -12.411950 | I6 |
| JX885140 | Rodentia | Dipodidae | Jaculus cf. hirtipes | 20.250000 | -13.270000 | H5 |
| JX885139 | Rodentia | Dipodidae | Jaculus cf. hirtipes | 20.250000 | -13.270000 | H5 |
| JX885138 | Rodentia | Dipodidae | Jaculus cf. hirtipes | 19.740000 | -14.370000 | I4 |
| 47887600-174c-46ab-9e5c-be1224554b14 | Rodentia | Dipodidae | Jaculus cf. hirtipes | 19.632245 | -14.538267 | I4 |
| a12a81d7-a48d-439a-93bf-a48e24638023 | Rodentia | Dipodidae | Jaculus cf. hirtipes | 15.565445 | -12.326598 | N7 |
| 06b586f1-550a-4afe-a29b-21c9fb3c82dc | Rodentia | Dipodidae | Jaculus cf. hirtipes | 16.219730 | -13.259707 | M6 |
| 901e2f58-4b11-4ad4-8c51-8bfc8468481e | Rodentia | Dipodidae | Jaculus cf. hirtipes | 16.633494 | -15.195661 | L4 |
| 3e53a02d-6169-4492-a906-203c2ba83bf0 | Rodentia | Dipodidae | Jaculus cf. hirtipes | 19.650517 | -14.504366 | I4 |
| b3a05aa5-04a1-4406-a274-cb31f5ac8056 | Rodentia | Dipodidae | Jaculus cf. hirtipes | 20.254134 | -13.295546 | H5 |
| a2201401-2b53-42d0-8e36-6d164f00f26c | Rodentia | Dipodidae | Jaculus cf. hirtipes | 20.507744 | -12.831218 | H6 |
| 25f8c31f-b64e-4f86-848e-487e45afc814 | Rodentia | Dipodidae | Jaculus cf. hirtipes | 20.556652 | -12.571878 | H6 |
| 84f30855-efd5-4fbd-aff1-d0da524168d0 | Rodentia | Dipodidae | Jaculus cf. hirtipes | 21.352217 | -13.038678 | G6 |
| 3e81e026-980f-4ee2-a46c-068958c208a7 | Rodentia | Dipodidae | Jaculus cf. hirtipes | 20.015839 | -13.887178 | I5 |
| fd8204a6-e281-40e0-a99b-992ab01b4ad0 | Rodentia | Dipodidae | Jaculus cf. hirtipes | 20.724430 | -16.057100 | H3 |
| fe295247-e4d2-47c3-9b67-9a54c790142c | Rodentia | Dipodidae | Jaculus cf. hirtipes | 16.434780 | -14.036880 | M5 |
| fe50e786-190f-4974-a7b1-efe0ca2f75b5 | Rodentia | Dipodidae | Jaculus cf. hirtipes | 16.566010 | -14.198147 | M5 |
| 558886ad-8f9e-4b63-a72a-21f917d8378c | Rodentia | Dipodidae | Jaculus cf. hirtipes | 20.929055 | -16.221407 | H2 |
| addd2093-6afe-4006-85fd-62f66a2821ec | Rodentia | Dipodidae | Jaculus cf. hirtipes | 17.938317 | -12.267117 | K7 |
| JX885137 | Rodentia | Dipodidae | Jaculus jaculus | 17.950000 | -12.267000 | K7 |
| JX885136 | Rodentia | Dipodidae | Jaculus jaculus | 17.950000 | -12.267000 | K7 |
| JX885135 | Rodentia | Dipodidae | Jaculus jaculus | 17.950000 | -12.267000 | K7 |
| JX885142 | Rodentia | Dipodidae | Jaculus jaculus | 22.970000 | -12.000000 | E7 |
| JX885141 | Rodentia | Dipodidae | Jaculus jaculus | 22.970000 | -12.000000 | E7 |
| http://n2t.net/ark:/65665/38f9afa12-5d67-4bbe-b3af-78b964a4d74b | Rodentia | Dipodidae | Jaculus jaculus | 20.536743 | -12.961179 | H6 |
| 2728f8c2-52a0-495a-8ea1-db7f5b2e4e1f | Rodentia | Dipodidae | Jaculus jaculus | 20.116955 | -13.705953 | I5 |
| ec719c0c-480d-4134-91d7-d67b836a5baf | Rodentia | Dipodidae | Jaculus jaculus | 19.007396 | -15.195947 | J3 |
| 9db84cb3-861c-4873-a60b-0f5f8421f853 | Rodentia | Dipodidae | Jaculus jaculus | 17.181650 | -12.077424 | L7 |
| c38a1966-46c1-4832-9e2c-b81d91376985 | Rodentia | Dipodidae | Jaculus jaculus | 16.677626 | -16.408948 | L2 |
| d92bfd8e-b94d-4c91-9a9c-e895be675bfa | Rodentia | Dipodidae | Jaculus jaculus | 16.677626 | -16.408948 | L2 |
| 99e7a896-44c8-44d7-b310-7e39ec4baf8c | Rodentia | Dipodidae | Jaculus jaculus | 16.607238 | -16.441857 | M2 |
| 34e1c92d-c47f-4ca6-8d3f-f54e8e05fb0d | Rodentia | Dipodidae | Jaculus jaculus | 18.429193 | -14.801320 | J4 |
| 1de584f3-3a6b-40b0-ac7b-22ecfab83d53 | Rodentia | Dipodidae | Jaculus jaculus | 18.489588 | -14.643788 | J4 |
| 4715f0fb-1107-4161-94fb-c438862fbd61 | Rodentia | Dipodidae | Jaculus jaculus | 18.261320 | -14.980998 | K4 |
| ed9ec462-ff3c-4bc2-bf5b-6232780cd661 | Rodentia | Dipodidae | Jaculus jaculus | 18.196812 | -15.046870 | K4 |
| aacc3bf0-1a4d-453b-bf88-1b16770232e2 | Rodentia | Dipodidae | Jaculus jaculus | 16.067740 | -11.508705 | M7 |
| dced0ec4-19ef-4343-a801-fc54f8abf385 | Rodentia | Dipodidae | Jaculus jaculus | 17.993385 | -11.880048 | K7 |
| da694d23-066f-41eb-b55b-5a56af8d5df6 | Rodentia | Dipodidae | Jaculus jaculus | 18.356822 | -11.816107 | J7 |
| 5146d15f-19de-43b5-a40c-ddf07d0aacbd | Rodentia | Dipodidae | Jaculus jaculus | 18.356822 | -11.816107 | J7 |
| 444cd76b-23c5-41ca-b97f-0ba9b1163f61 | Rodentia | Dipodidae | Jaculus jaculus | 20.844486 | -16.148816 | H2 |
| 6bc7850d-8201-484a-8732-225a30f2b6d6 | Rodentia | Dipodidae | Jaculus jaculus | 18.108780 | -11.915528 | K7 |
| 18216dae-3ce6-47de-b7f9-e86c7216019a | Rodentia | Dipodidae | Jaculus jaculus | 18.558558 | -11.248490 | J8 |
| 61999a6c-e6b2-40da-a7cf-ad892cddee46 | Rodentia | Dipodidae | Jaculus jaculus | 18.382915 | -9.312508 | J10 |
| af4757fa-8187-4f2a-8717-b81138134b12 | Rodentia | Dipodidae | Jaculus jaculus | 20.092682 | -15.926868 | I3 |
| 5cf33490-863f-4ed1-8a2b-ab420a4aa3d1 | Rodentia | Dipodidae | Jaculus jaculus | 21.020971 | -16.304408 | H2 |
| cc4fe17e-5b91-42d6-904a-d694da8c14a3 | Rodentia | Dipodidae | Jaculus jaculus | 18.094020 | -12.131506 | K7 |
| 0847cfa8-c144-4a45-8dfc-9325c30a13fb | Rodentia | Dipodidae | Jaculus jaculus | 17.590710 | -12.847843 | K6 |
| bbd95919-f36d-4df6-84ca-1e3747e2cc4c | Rodentia | Dipodidae | Jaculus jaculus | 19.650517 | -14.504366 | I4 |
| 0961ef0c-33ea-45cd-b4fe-83b79e5e6712 | Rodentia | Dipodidae | Jaculus jaculus | 19.650517 | -14.504366 | I4 |
| 3050e4e1-a6e9-4eb4-b56f-74634329ea2c | Rodentia | Dipodidae | Jaculus jaculus | 19.650517 | -14.504366 | I4 |
| e97d0e5c-1d50-49fb-a8ea-c805166a0621 | Rodentia | Dipodidae | Jaculus jaculus | 20.252778 | -13.310647 | H5 |
| 69519a0a-de10-4526-9ce3-23cbf0247290 | Rodentia | Dipodidae | Jaculus jaculus | 21.438097 | -12.980000 | G6 |
| 0b2e0fe4-7318-4100-8453-90b156ca413b | Rodentia | Dipodidae | Jaculus jaculus | 19.959787 | -16.084098 | I3 |
| 509a8a5a-2cf9-4009-a400-52de089f1b7b | Rodentia | Dipodidae | Jaculus jaculus | 18.480463 | -16.022135 | J3 |
| 3ac09b31-c805-4fda-8028-a80f31a0565d | Rodentia | Dipodidae | Jaculus jaculus | 18.480463 | -16.022135 | J3 |
| 642fdb3d-9459-4c1f-9e9c-c51c070d51d9 | Rodentia | Dipodidae | Jaculus jaculus | 18.480463 | -16.022135 | J3 |
| ed241011-6b31-45ae-8144-ad22033a8d22 | Rodentia | Dipodidae | Jaculus jaculus | 18.236072 | -11.518997 | K7 |
| 1f173dea-ca38-4ba1-ac39-9dae5e008fb8 | Rodentia | Dipodidae | Jaculus jaculus | 20.613110 | -16.012605 | H3 |
| 6c9c1d5d-508a-44d1-8d0c-c8e6a0295500 | Rodentia | Dipodidae | Jaculus jaculus | 20.378803 | -15.991290 | H3 |
| 74dca11f-f73f-4eac-902f-7966b043c0f8 | Rodentia | Dipodidae | Jaculus jaculus | 18.900835 | -15.415980 | J3 |
| 210b9060-735c-46b4-bdb2-29236802d3ac | Rodentia | Dipodidae | Jaculus jaculus | 20.601737 | -16.012458 | H3 |
| 463926c9-10b0-4248-852f-3d9783cdf626 | Rodentia | Dipodidae | Jaculus jaculus | 20.996882 | -16.282512 | H2 |
| d6269c2b-386e-459e-b568-684513c73e67 | Rodentia | Dipodidae | Jaculus jaculus | 18.020882 | -12.049943 | K7 |
| 92b77eb7-67a1-4ae4-a655-ca9f35a8cbee | Rodentia | Dipodidae | Jaculus jaculus | 17.895172 | -11.716192 | K7 |
| 74ccd628-5050-4975-9ae9-8382707215c0 | Rodentia | Dipodidae | Jaculus jaculus | 20.377967 | -15.991150 | H3 |
| 079f8615-8239-42e3-b795-b65d2e167f54 | Rodentia | Dipodidae | Jaculus jaculus | 19.640667 | -14.521700 | I4 |
| 8677de24-e535-4485-b344-931aef18ee1d | Rodentia | Dipodidae | Jaculus jaculus | 19.438717 | -14.753883 | I4 |
| 5f100f73-ce4c-43ee-af60-f68e56c02426 | Rodentia | Dipodidae | Jaculus jaculus | 17.408217 | -16.062283 | L3 |
| 74492eb3-594c-4c39-b951-ef01b636e05e | Rodentia | Dipodidae | Jaculus jaculus | 17.392517 | -13.452850 | L5 |
| 4c6cc20a-caf8-4e4f-9ac5-079ec4f67309 | Rodentia | Dipodidae | Jaculus jaculus | 17.692550 | -12.571133 | K6 |
| f0624e56-45c0-471e-9fb0-ab34e45e89dd | Rodentia | Dipodidae | Jaculus jaculus | 17.938317 | -12.267117 | K7 |
| b44d5904-3ab7-470d-9a4b-9cb9bbfe7b37 | Rodentia | Dipodidae | Jaculus jaculus | 17.899250 | -12.333783 | K6 |
| 659338fd-3457-4224-940a-4c1756da4619 | Rodentia | Dipodidae | Jaculus jaculus | 17.195050 | -7.141233 | L12 |
| 0730b6b5-1dc3-4b7f-bace-de15d98a97e1 | Rodentia | Dipodidae | Jaculus jaculus | 17.225267 | -7.068600 | L12 |
| http://n2t.net/ark:/65665/3c4e9e907-cf91-4765-8578-bc4434a552df | Rodentia | Dipodidae | Jaculus sp. | 17.266783 | -16.028417 | L3 |
| http://n2t.net/ark:/65665/35aa27852-56f9-4361-9138-7b7656ad9f01 | Rodentia | Dipodidae | Jaculus sp. | 17.266783 | -16.028417 | L3 |
| http://n2t.net/ark:/65665/3cca8bed8-adc3-4404-b78a-a9853a2a7213 | Rodentia | Dipodidae | Jaculus sp. | 17.266783 | -16.028417 | L3 |
| http://n2t.net/ark:/65665/319508d98-5749-4bd7-a92b-903cf4d7919d | Rodentia | Dipodidae | Jaculus sp. | 17.266783 | -16.028417 | L3 |
| http://n2t.net/ark:/65665/3141150d7-cddc-48cc-a201-378f9f43fe31 | Rodentia | Dipodidae | Jaculus sp. | 17.266783 | -16.028417 | L3 |
| http://n2t.net/ark:/65665/3fc2b9f83-7a8a-4d5d-96e6-0cee69a6a8ec | Rodentia | Dipodidae | Jaculus sp. | 17.266783 | -16.028417 | L3 |
| http://coldb.mnhn.fr/catalognumber/mnhn/zm/mo-2001-765 | Rodentia | Dipodidae | Jaculus sp. | 18.599848 | -16.079906 | J3 |
| http://coldb.mnhn.fr/catalognumber/mnhn/zm/mo-2001-771 | Rodentia | Dipodidae | Jaculus sp. | 17.439796 | -16.062595 | L3 |
| http://coldb.mnhn.fr/catalognumber/mnhn/zm/mo-2001-755 | Rodentia | Dipodidae | Jaculus sp. | 17.750000 | -12.233000 | K7 |
| http://coldb.mnhn.fr/catalognumber/mnhn/zm/mo-2001-754 | Rodentia | Dipodidae | Jaculus sp. | 17.750000 | -12.233000 | K7 |
| http://coldb.mnhn.fr/catalognumber/mnhn/zm/mo-2001-753 | Rodentia | Dipodidae | Jaculus sp. | 17.750000 | -12.233000 | K7 |
| http://n2t.net/ark:/65665/387fcd5f9-a21e-4f6f-9335-1dd297d50692 | Rodentia | Dipodidae | Jaculus sp. | 18.015000 | -15.910000 | K3 |
| http://n2t.net/ark:/65665/38f92f17b-e31f-4bcf-8c32-15cf0624f25b | Rodentia | Dipodidae | Jaculus sp. | 18.015000 | -15.910000 | K3 |
| http://n2t.net/ark:/65665/34c1840b4-57f0-4b14-87b7-6ef3be3f0112 | Rodentia | Dipodidae | Jaculus sp. | 18.015000 | -15.910000 | K3 |
| http://n2t.net/ark:/65665/33ed5f675-15df-4637-9441-b11325acdf14 | Rodentia | Dipodidae | Jaculus sp. | 18.015000 | -15.910000 | K3 |
| http://n2t.net/ark:/65665/3423f800c-78b5-420a-b393-347777d7e969 | Rodentia | Dipodidae | Jaculus sp. | 18.015000 | -15.910000 | K3 |
| http://n2t.net/ark:/65665/31bed9972-eddc-4957-ae8d-ac8507afd78b | Rodentia | Dipodidae | Jaculus sp. | 18.015000 | -15.910000 | K3 |
| http://n2t.net/ark:/65665/32e74bc4e-e940-41aa-837d-0785dbb8f85f | Rodentia | Dipodidae | Jaculus sp. | 18.015000 | -15.910000 | K3 |
| http://n2t.net/ark:/65665/37ddd89ab-d4a4-4cb4-b5da-9a6584ec8374 | Rodentia | Dipodidae | Jaculus sp. | 18.015000 | -15.910000 | K3 |
| http://n2t.net/ark:/65665/3bcc076e7-399b-4f02-af62-37690926306e | Rodentia | Dipodidae | Jaculus sp. | 18.015000 | -15.910000 | K3 |
| http://n2t.net/ark:/65665/3fb953efc-f15c-4afd-a781-a61c776e1067 | Rodentia | Dipodidae | Jaculus sp. | 18.015000 | -15.910000 | K3 |
| http://n2t.net/ark:/65665/38c5068c3-ff07-4cd5-8856-515581dc99d1 | Rodentia | Dipodidae | Jaculus sp. | 18.015000 | -15.910000 | K3 |
| http://n2t.net/ark:/65665/3e7e7588f-f771-44a0-9c44-33d45f561723 | Rodentia | Dipodidae | Jaculus sp. | 18.015000 | -15.910000 | K3 |
| http://n2t.net/ark:/65665/358235e6f-5888-4fff-90ad-d37e3c976d83 | Rodentia | Dipodidae | Jaculus sp. | 18.015000 | -15.910000 | K3 |
| http://n2t.net/ark:/65665/33a4e3d8b-7e54-4c85-96c5-7167287c4633 | Rodentia | Dipodidae | Jaculus sp. | 18.015000 | -15.910000 | K3 |
| http://n2t.net/ark:/65665/35479430b-7def-4f9e-93d2-f64ba396ff23 | Rodentia | Dipodidae | Jaculus sp. | 18.015000 | -15.910000 | K3 |
| http://n2t.net/ark:/65665/3d5d82a9f-b5d2-4d91-8e56-ec626b91599f | Rodentia | Dipodidae | Jaculus sp. | 18.015000 | -15.910000 | K3 |
| http://n2t.net/ark:/65665/3bbb91c0c-1bce-4327-9503-b985dd0ab788 | Rodentia | Dipodidae | Jaculus sp. | 18.015000 | -15.910000 | K3 |
| http://n2t.net/ark:/65665/3ebf4c973-364e-40f9-93b8-0836e6dab529 | Rodentia | Dipodidae | Jaculus sp. | 18.015000 | -15.910000 | K3 |
| http://n2t.net/ark:/65665/37c1af708-0e60-4cec-9ea0-eb7c45f15572 | Rodentia | Dipodidae | Jaculus sp. | 18.015000 | -15.910000 | K3 |
| http://n2t.net/ark:/65665/3b2537b7d-52a5-43a4-ae80-8703fb4ace1f | Rodentia | Dipodidae | Jaculus sp. | 18.015000 | -15.910000 | K3 |
| http://n2t.net/ark:/65665/37b8dac71-9caa-41f5-ae8b-d2cbca56cdf6 | Rodentia | Dipodidae | Jaculus sp. | 18.015000 | -15.910000 | K3 |
| http://n2t.net/ark:/65665/3df846492-e842-4bf4-8046-7fbb7a258a01 | Rodentia | Dipodidae | Jaculus sp. | 18.015000 | -15.910000 | K3 |
| http://n2t.net/ark:/65665/338f0732e-bcdb-4ef8-85bd-2672fd11a9b1 | Rodentia | Dipodidae | Jaculus sp. | 18.015000 | -15.910000 | K3 |
| http://n2t.net/ark:/65665/332c29781-c1e4-4dd4-a6c5-ac6c4d0a10b9 | Rodentia | Dipodidae | Jaculus sp. | 18.015000 | -15.910000 | K3 |
| http://n2t.net/ark:/65665/363e255bd-c2e1-49de-84c6-319c224fa97a | Rodentia | Dipodidae | Jaculus sp. | 18.015000 | -15.910000 | K3 |
| http://n2t.net/ark:/65665/35f0924dc-f315-46d7-b98e-29fa1a93ca0a | Rodentia | Dipodidae | Jaculus sp. | 18.015000 | -15.910000 | K3 |
| http://n2t.net/ark:/65665/3118c23bc-6d14-4ce4-b516-8fae1d81588f | Rodentia | Dipodidae | Jaculus sp. | 18.015000 | -15.910000 | K3 |
| http://coldb.mnhn.fr/catalognumber/mnhn/zm/mo-2001-758 | Rodentia | Dipodidae | Jaculus sp. | 18.267000 | -15.883000 | K3 |
| http://n2t.net/ark:/65665/3daa714fe-0d5c-4763-9078-30f2b2e264a9 | Rodentia | Dipodidae | Jaculus sp. | 18.185000 | -16.011000 | K3 |
| http://n2t.net/ark:/65665/37cebe4b3-8c97-4feb-894c-5f99ab1a4501 | Rodentia | Dipodidae | Jaculus sp. | 18.185000 | -16.011000 | K3 |
| http://n2t.net/ark:/65665/3a87671a8-7382-4fd9-9f01-9db3e2afed87 | Rodentia | Dipodidae | Jaculus sp. | 18.185000 | -16.011000 | K3 |
| http://n2t.net/ark:/65665/3e0cfa4da-7b4a-4562-9cb6-b4bc6445f42b | Rodentia | Dipodidae | Jaculus sp. | 18.185000 | -16.011000 | K3 |
| http://n2t.net/ark:/65665/330843d4f-0850-4542-8583-c65d6f2843ea | Rodentia | Dipodidae | Jaculus sp. | 18.185000 | -16.011000 | K3 |
| http://coldb.mnhn.fr/catalognumber/mnhn/zm/mo-1995-570 | Rodentia | Dipodidae | Jaculus sp. | 19.831000 | -15.923000 | I3 |
| http://coldb.mnhn.fr/catalognumber/mnhn/zm/mo-1977-187 | Rodentia | Dipodidae | Jaculus sp. | 19.831000 | -15.923000 | I3 |
| http://coldb.mnhn.fr/catalognumber/mnhn/zm/mo-2001-756 | Rodentia | Dipodidae | Jaculus sp. | 19.883000 | -16.300000 | I2 |
| http://coldb.mnhn.fr/catalognumber/mnhn/zm/mo-2001-762 | Rodentia | Dipodidae | Jaculus sp. | 19.022000 | -15.225000 | J3 |
| http://coldb.mnhn.fr/catalognumber/mnhn/zm/mo-2001-761 | Rodentia | Dipodidae | Jaculus sp. | 19.022000 | -15.225000 | J3 |
| http://coldb.mnhn.fr/catalognumber/mnhn/zm/mo-2001-760 | Rodentia | Dipodidae | Jaculus sp. | 19.022000 | -15.225000 | J3 |
| http://coldb.mnhn.fr/catalognumber/mnhn/zm/mo-2001-759 | Rodentia | Dipodidae | Jaculus sp. | 19.022000 | -15.225000 | J3 |
| http://coldb.mnhn.fr/catalognumber/mnhn/zm/mo-2001-757 | Rodentia | Dipodidae | Jaculus sp. | 18.833000 | -15.467000 | J3 |
| http://coldb.mnhn.fr/catalognumber/mnhn/zm/mo-2001-763 | Rodentia | Dipodidae | Jaculus sp. | 16.617000 | -15.483000 | L3 |
| http://n2t.net/ark:/65665/300f5d696-1a72-4c52-936c-6aa3ac9e5472 | Rodentia | Dipodidae | Jaculus sp. | 22.678530 | -12.707130 | F6 |
| http://n2t.net/ark:/65665/3a881e370-ffd4-49bf-8c8e-1a053271be18 | Rodentia | Dipodidae | Jaculus sp. | 22.678530 | -12.707130 | F6 |
| http://n2t.net/ark:/65665/3b3a9bfde-059d-4afd-b2b2-0d6b295a58e1 | Rodentia | Dipodidae | Jaculus sp. | 22.678530 | -12.707130 | F6 |
| http://n2t.net/ark:/65665/3f711d97d-f4b5-456e-b5e2-2b149ae89108 | Rodentia | Dipodidae | Jaculus sp. | 22.678530 | -12.707130 | F6 |
| http://n2t.net/ark:/65665/369078192-e663-4a1f-be54-bf8c07c47964 | Rodentia | Dipodidae | Jaculus sp. | 22.678530 | -12.707130 | F6 |
| http://n2t.net/ark:/65665/3c7b0a123-0b69-4ac5-ac6f-cd0915e9399d | Rodentia | Dipodidae | Jaculus sp. | 22.678530 | -12.707130 | F6 |
| http://n2t.net/ark:/65665/39a0c4bcd-aab0-4fc5-979f-807f572e0536 | Rodentia | Dipodidae | Jaculus sp. | 22.678530 | -12.707130 | F6 |
| http://n2t.net/ark:/65665/3c5e5519d-9e79-44e1-9fa0-62795ba54c9e | Rodentia | Dipodidae | Jaculus sp. | 22.678530 | -12.707130 | F6 |
| http://n2t.net/ark:/65665/34a68636a-912c-4b58-8e0b-0fe8c55cc3f9 | Rodentia | Dipodidae | Jaculus sp. | 22.678530 | -12.707130 | F6 |
| http://n2t.net/ark:/65665/3f6abf0a0-b511-4def-9105-352893777880 | Rodentia | Dipodidae | Jaculus sp. | 22.678530 | -12.707130 | F6 |
| http://n2t.net/ark:/65665/3ff778b35-3f2c-4e22-9783-32313740c4d5 | Rodentia | Dipodidae | Jaculus sp. | 22.678530 | -12.707130 | F6 |
| http://n2t.net/ark:/65665/3dbf881bf-bd2e-478c-9f00-866d7ef2c0a2 | Rodentia | Dipodidae | Jaculus sp. | 22.678530 | -12.707130 | F6 |
| http://n2t.net/ark:/65665/3ea3b352c-131c-48d5-861d-5583eaa6fcdf | Rodentia | Dipodidae | Jaculus sp. | 22.678530 | -12.707130 | F6 |
| http://n2t.net/ark:/65665/33eb0de5f-0c74-4287-8d82-93b5e85baf3b | Rodentia | Dipodidae | Jaculus sp. | 22.678530 | -12.707130 | F6 |
| http://n2t.net/ark:/65665/353ea9be2-8479-41d4-b1c7-38c9ffddea55 | Rodentia | Dipodidae | Jaculus sp. | 22.678530 | -12.707130 | F6 |
| http://coldb.mnhn.fr/catalognumber/mnhn/zm/mo-2001-766 | Rodentia | Dipodidae | Jaculus sp. | 19.181000 | -16.340800 | J2 |
| http://n2t.net/ark:/65665/3bdf4fab3-9f8b-4f34-9c97-be77ecbfdf82 | Rodentia | Dipodidae | Jaculus sp. | 19.012000 | -15.207000 | J3 |
| http://n2t.net/ark:/65665/35dda9a99-f65a-4185-9a0f-1ca5717c1966 | Rodentia | Dipodidae | Jaculus sp. | 19.012000 | -15.207000 | J3 |
| http://n2t.net/ark:/65665/3c66f2846-88a0-4c77-8194-07fad8945791 | Rodentia | Dipodidae | Jaculus sp. | 19.012000 | -15.207000 | J3 |
| http://n2t.net/ark:/65665/3586f9965-d11a-4996-b625-a45c7ab9f1f1 | Rodentia | Dipodidae | Jaculus sp. | 19.012000 | -15.207000 | J3 |
| http://n2t.net/ark:/65665/3091d0456-489f-499e-802b-9c0afc0c9132 | Rodentia | Dipodidae | Jaculus sp. | 19.012000 | -15.207000 | J3 |
| http://n2t.net/ark:/65665/3705f4ca8-ec31-4bec-affd-197a3af49d3d | Rodentia | Dipodidae | Jaculus sp. | 19.012000 | -15.207000 | J3 |
| http://n2t.net/ark:/65665/3fcbf08dc-f7ec-4472-8ed6-c8efa33938bb | Rodentia | Dipodidae | Jaculus sp. | 19.012000 | -15.207000 | J3 |
| http://n2t.net/ark:/65665/33044cdcc-216a-4d07-bb41-38c45cfd91df | Rodentia | Dipodidae | Jaculus sp. | 19.012000 | -15.207000 | J3 |
| http://n2t.net/ark:/65665/3d7e54af3-9170-4ea7-ad4c-430e71f4b0e9 | Rodentia | Dipodidae | Jaculus sp. | 19.012000 | -15.207000 | J3 |
| http://n2t.net/ark:/65665/3e193ee17-b15d-405e-8e55-4376a00cac37 | Rodentia | Dipodidae | Jaculus sp. | 19.012000 | -15.207000 | J3 |
| http://n2t.net/ark:/65665/38a1ac167-3f30-4d6a-9f21-af3839812f51 | Rodentia | Dipodidae | Jaculus sp. | 19.012000 | -15.207000 | J3 |
| http://n2t.net/ark:/65665/34d773fd9-13ad-46ce-ad3b-07c98ddece42 | Rodentia | Dipodidae | Jaculus sp. | 19.012000 | -15.207000 | J3 |
| http://n2t.net/ark:/65665/3e6e83521-e162-4a41-9a85-20c2e8d00ba6 | Rodentia | Dipodidae | Jaculus sp. | 19.012000 | -15.207000 | J3 |
| http://coldb.mnhn.fr/catalognumber/mnhn/zm/mo-2001-40 | Rodentia | Dipodidae | Jaculus sp. | 16.728000 | -16.394000 | L2 |
| http://coldb.mnhn.fr/catalognumber/mnhn/zm/mo-2001-770 | Rodentia | Dipodidae | Jaculus sp. | 16.607000 | -16.439323 | M2 |
| http://n2t.net/ark:/65665/352f4a9e5-3e45-48cb-b299-ab2e59f286bc | Rodentia | Dipodidae | Jaculus sp. | 20.550000 | -12.500000 | H6 |
| http://n2t.net/ark:/65665/312a2356d-6c7f-4a5f-8758-2157c5ad4742 | Rodentia | Dipodidae | Jaculus sp. | 20.550000 | -12.500000 | H6 |
| http://n2t.net/ark:/65665/3eb86cafe-b6ec-47c7-9b2f-583bc41653ef | Rodentia | Dipodidae | Jaculus sp. | 20.550000 | -12.500000 | H6 |
| http://n2t.net/ark:/65665/3fff32ec3-b8ad-4f3e-9777-4005f8e72187 | Rodentia | Dipodidae | Jaculus sp. | 20.550000 | -12.500000 | H6 |
| http://n2t.net/ark:/65665/398a0a6a6-1aab-4f66-97ac-3479086f01ab | Rodentia | Dipodidae | Jaculus sp. | 20.550000 | -12.500000 | H6 |
| http://n2t.net/ark:/65665/35cb8f15e-d4df-4bcb-9b38-eda749867e23 | Rodentia | Dipodidae | Jaculus sp. | 20.550000 | -12.500000 | H6 |
| http://n2t.net/ark:/65665/32867888f-703a-4397-8593-a5f37bc6fa28 | Rodentia | Dipodidae | Jaculus sp. | 20.550000 | -12.500000 | H6 |
| http://n2t.net/ark:/65665/395966dcf-50ab-493e-947a-84de4c482c28 | Rodentia | Dipodidae | Jaculus sp. | 20.550000 | -12.500000 | H6 |
| http://n2t.net/ark:/65665/35f4f6fe5-f610-4824-a148-1f3561a74e1c | Rodentia | Dipodidae | Jaculus sp. | 20.550000 | -12.500000 | H6 |
| http://n2t.net/ark:/65665/31459d6df-cbdf-45fc-8be6-3fda00c50b9d | Rodentia | Dipodidae | Jaculus sp. | 21.525000 | -12.860600 | G6 |
| http://n2t.net/ark:/65665/3cc349730-d2fb-4757-b6f4-49ae397ab549 | Rodentia | Dipodidae | Jaculus sp. | 20.161000 | -13.649000 | H5 |
| http://n2t.net/ark:/65665/3d4144b62-e318-4c73-812f-5621052bcc72 | Rodentia | Dipodidae | Jaculus sp. | 20.161000 | -13.649000 | H5 |
| http://n2t.net/ark:/65665/33afd7c92-7bc4-40e6-8cfe-356fa84e3f57 | Rodentia | Dipodidae | Jaculus sp. | 20.536743 | -12.961179 | H6 |
| http://n2t.net/ark:/65665/378992d9d-523d-4e40-a586-604672e33d4c | Rodentia | Dipodidae | Jaculus sp. | 20.536743 | -12.961179 | H6 |
| http://n2t.net/ark:/65665/300e8b3b3-9df2-4adc-b332-a4364e49bac6 | Rodentia | Dipodidae | Jaculus sp. | 20.536743 | -12.961179 | H6 |
| http://n2t.net/ark:/65665/39a62182d-bffd-4b43-a130-9ed66ee97fd7 | Rodentia | Dipodidae | Jaculus sp. | 20.536743 | -12.961179 | H6 |
| http://n2t.net/ark:/65665/37dc5a3de-48b8-447a-b1ad-c2b5804292b7 | Rodentia | Dipodidae | Jaculus sp. | 20.536743 | -12.961179 | H6 |
| http://n2t.net/ark:/65665/34f09d3f0-2743-4d99-9f2e-80bf643fc37a | Rodentia | Dipodidae | Jaculus sp. | 20.536743 | -12.961179 | H6 |
| http://coldb.mnhn.fr/catalognumber/mnhn/zm/mo-2001-752 | Rodentia | Dipodidae | Jaculus sp. | 18.767000 | -15.317000 | J3 |
| http://coldb.mnhn.fr/catalognumber/mnhn/zm/mo-2001-751 | Rodentia | Dipodidae | Jaculus sp. | 18.767000 | -15.317000 | J3 |
| http://n2t.net/ark:/65665/38adef552-67ee-4d78-9383-04b3a0e1f5d3 | Rodentia | Dipodidae | Jaculus sp. | 17.030000 | -13.920000 | L5 |
| http://n2t.net/ark:/65665/3ce6a4486-1c79-44a1-bd7f-c9da5d1e12cd | Rodentia | Dipodidae | Jaculus sp. | 17.030000 | -13.920000 | L5 |
| http://n2t.net/ark:/65665/370185d5d-89fc-4bbf-81b0-7a7520069368 | Rodentia | Dipodidae | Jaculus sp. | 17.030000 | -13.920000 | L5 |
| http://n2t.net/ark:/65665/3350a4712-dd1d-40f7-a64f-8c468e969b85 | Rodentia | Dipodidae | Jaculus sp. | 17.030000 | -13.920000 | L5 |
| http://n2t.net/ark:/65665/38af75ef8-04b9-461a-ae47-788723198798 | Rodentia | Dipodidae | Jaculus sp. | 17.030000 | -13.920000 | L5 |
| http://n2t.net/ark:/65665/396d9da11-a432-49b1-9567-98b0c3dbc4d3 | Rodentia | Dipodidae | Jaculus sp. | 21.020000 | -13.150000 | H6 |
| http://n2t.net/ark:/65665/3098ef30f-3917-4d91-8c97-d8b78a844f68 | Rodentia | Dipodidae | Jaculus sp. | 21.020000 | -13.150000 | H6 |
| http://n2t.net/ark:/65665/36cce8261-1b26-4cce-9482-1003b10cc301 | Rodentia | Dipodidae | Jaculus sp. | 21.020000 | -13.150000 | H6 |
| http://n2t.net/ark:/65665/3a97bc1e3-c92e-4c0e-b5ea-b6ad0000286f | Rodentia | Dipodidae | Jaculus sp. | 21.020000 | -13.150000 | H6 |
| http://coldb.mnhn.fr/catalognumber/mnhn/zm/mo-2001-769 | Rodentia | Dipodidae | Jaculus sp. | 19.333333 | -16.283333 | I2 |
| http://coldb.mnhn.fr/catalognumber/mnhn/zm/mo-2001-764 | Rodentia | Dipodidae | Jaculus sp. | 17.053000 | -16.263000 | L2 |
| bd446645-f692-4026-bd46-69e96c776b02 | Rodentia | Dipodidae | Jaculus sp. | 20.172297 | -13.658762 | H5 |
| 4dd1c426-60c1-4dc3-b734-784292859e9c | Rodentia | Dipodidae | Jaculus sp. | 18.442815 | -11.387390 | J7 |
| a7b89eb2-105e-4604-a5c4-5545da30c418 | Rodentia | Dipodidae | Jaculus sp. | 18.383822 | -8.521617 | J10 |
| 562ba92f-4a51-4c4f-acad-73ae887a8e15 | Rodentia | Dipodidae | Jaculus sp. | 18.000483 | -11.883720 | K7 |
| b908adc3-e573-46dd-a66a-49675f7b9669 | Rodentia | Dipodidae | Jaculus sp. | 14.834637 | -12.332624 | N7 |
| fabe72b6-c51f-4893-93fb-6038fd6d0d7c | Rodentia | Dipodidae | Jaculus sp. | 20.536019 | -12.954155 | H6 |
| 7b4ec70a-08ad-45a1-bc3f-461b338b821f | Rodentia | Dipodidae | Jaculus sp. | 17.649985 | -11.395480 | K7 |
| dee4f62b-99a2-4a61-817b-35b79423a8ec | Rodentia | Dipodidae | Jaculus sp. | 20.944458 | -16.549482 | H2 |
| f558072b-ca7a-46a9-93d1-15e8b072e90f | Rodentia | Dipodidae | Jaculus sp. | 21.372827 | -11.910393 | G7 |
| 7a0bbd48-12bc-443d-9cca-e35ea28f44bc | Rodentia | Dipodidae | Jaculus sp. | 18.401588 | -12.380853 | J6 |
| 103e1bbc-7144-4367-b135-bb2120a4a713 | Rodentia | Dipodidae | Jaculus sp. | 17.267002 | -10.692772 | L8 |
| aa192145-8902-47b2-9d1d-f12b115e27aa | Rodentia | Dipodidae | Jaculus sp. | 16.834367 | -16.089950 | L3 |
| 03d96943-8972-4fad-9482-c8deac5d8884 | Rodentia | Dipodidae | Jaculus sp. | 17.939317 | -12.258333 | K7 |
| 84658351-fb69-4117-9cd2-d616ad79da7c | Rodentia | Dipodidae | Jaculus sp. | 16.896667 | -7.443333 | L12 |
| 6aa27c6c-9bc1-458a-b80c-50c595d4c507 | Rodentia | Dipodidae | Jaculus sp. | 17.532233 | -7.442383 | K12 |
| 968fe8ac-47d7-430f-8c58-df415e5a4dfd | Rodentia | Dipodidae | Jaculus sp. | 18.113717 | -8.033333 | K11 |
| 3203d3f3-a118-40f7-b11f-b4b0acaf20f2 | Rodentia | Dipodidae | Jaculus sp. | 18.378767 | -11.196033 | J8 |
| ccecb151-6931-4c58-bed2-ab24873e73c6 | Rodentia | Dipodidae | Jaculus sp. | 18.633533 | -11.592550 | J7 |
| https://observation.org/observation/97149012 | Rodentia | Hystricidae | Hystrix cristata | 20.251732 | -13.086483 | H6 |
| http://n2t.net/ark:/65665/312a2652b-dce0-4698-82bf-8203bb0137b2 | Rodentia | Hystricidae | Hystrix cristata | 16.550000 | -15.766667 | M3 |
| 3720f76e-350e-4e20-bd5d-6b0c5e30bc75 | Rodentia | Hystricidae | Hystrix cristata | 16.371455 | -10.245473 | M9 |
| 3cca270c-13da-4745-9d49-03902d94cb53 | Rodentia | Hystricidae | Hystrix cristata | 15.563663 | -10.982040 | N8 |
| ac10b8e8-6cd5-45c6-83bb-887012e07a7f | Rodentia | Hystricidae | Hystrix cristata | 15.946357 | -13.293712 | M6 |
| 3711f251-0055-4a46-92d4-412e42937dbf | Rodentia | Hystricidae | Hystrix cristata | 18.592830 | -10.026298 | J9 |
| db974873-d46d-4208-8aab-03a53d3fc580 | Rodentia | Hystricidae | Hystrix cristata | 18.390480 | -8.554273 | J10 |
| 9a8ba4a0-5a34-4a15-8f33-5d7929971973 | Rodentia | Hystricidae | Hystrix cristata | 16.489543 | -11.057988 | M8 |
| 4eea9e08-5b31-41ae-866c-98c4b3571257 | Rodentia | Hystricidae | Hystrix cristata | 16.113668 | -10.959342 | M8 |
| 54043379-8c71-4698-af2d-f04defe1bd56 | Rodentia | Hystricidae | Hystrix cristata | 16.053245 | -11.669883 | M7 |
| a2ea58ca-9ef2-48c7-a0e6-e4acfb26ad0f | Rodentia | Hystricidae | Hystrix cristata | 16.002553 | -11.871748 | M7 |
| d3bc61a7-006f-4528-aead-0537a755e9ca | Rodentia | Hystricidae | Hystrix cristata | 15.957078 | -12.009859 | M7 |
| a1ef229e-eb55-4503-b28e-747b7b2c2fad | Rodentia | Hystricidae | Hystrix cristata | 15.591013 | -12.242632 | N7 |
| 7fc8c6f7-2a05-4931-a3c0-af296f77d5e1 | Rodentia | Hystricidae | Hystrix cristata | 18.053485 | -11.942891 | K7 |
| 6a21a38f-d867-4fc2-b298-d9a0cbe901f2 | Rodentia | Hystricidae | Hystrix cristata | 17.142555 | -11.885383 | L7 |
| 6c07fdc1-e6de-4096-b0e9-4868d95a8b6c | Rodentia | Hystricidae | Hystrix cristata | 17.173397 | -11.938015 | L7 |
| 8362c7b1-606e-4496-9957-e12d6d53bbdf | Rodentia | Hystricidae | Hystrix cristata | 17.382798 | -11.720837 | L7 |
| 2fbe8102-607c-4697-a2b9-fabf148b8972 | Rodentia | Hystricidae | Hystrix cristata | 17.426530 | -12.010112 | L7 |
| 79614e4b-100a-4061-b56b-8b2ccf798dd8 | Rodentia | Hystricidae | Hystrix cristata | 18.186980 | -11.746348 | K7 |
| 7c66607d-a4ed-4e74-a5b5-d84945c3f467 | Rodentia | Hystricidae | Hystrix cristata | 18.070578 | -12.333977 | K6 |
| 491c26d8-c26e-4f03-943a-9f8db8627306 | Rodentia | Hystricidae | Hystrix cristata | 17.737962 | -12.245253 | K7 |
| 08158789-55ad-4943-8bf3-6c01df62a620 | Rodentia | Hystricidae | Hystrix cristata | 17.821264 | -12.185762 | K7 |
| 3339dcc5-e201-41ac-9123-ca8946678096 | Rodentia | Hystricidae | Hystrix cristata | 16.701802 | -10.183587 | L9 |
| 4464cb9b-55e5-42e9-b6a4-2c1b655f32b7 | Rodentia | Hystricidae | Hystrix cristata | 20.519268 | -13.132583 | H6 |
| 26a87f79-1f85-4c66-8010-cf5c07ccc4ef | Rodentia | Hystricidae | Hystrix cristata | 17.401433 | -12.364150 | L6 |
| f9143cc0-353f-4428-8d45-2a77b6011ad8 | Rodentia | Hystricidae | Hystrix cristata | 17.274233 | -12.227017 | L7 |
| ebda2b37-2fdf-4a30-b4eb-b62096b9823e | Rodentia | Hystricidae | Hystrix cristata | 17.279133 | -12.221450 | L7 |
| 278b13ed-a514-4966-ad30-9c9a32adf314 | Rodentia | Hystricidae | Hystrix cristata | 18.391017 | -8.553600 | J10 |
| http://coldb.mnhn.fr/catalognumber/mnhn/zm/mo-1991-327 | Rodentia | Muridae | Acomys airensis | 17.873611 | -12.331667 | K6 |
| http://coldb.mnhn.fr/catalognumber/mnhn/zm/mo-1955-765 | Rodentia | Muridae | Acomys airensis | 17.873611 | -12.331667 | K6 |
| http://coldb.mnhn.fr/catalognumber/mnhn/zm/mo-1955-764 | Rodentia | Muridae | Acomys airensis | 17.873611 | -12.331667 | K6 |
| http://coldb.mnhn.fr/catalognumber/mnhn/zm/mo-1955-763 | Rodentia | Muridae | Acomys airensis | 17.873611 | -12.331667 | K6 |
| http://coldb.mnhn.fr/catalognumber/mnhn/zm/mo-1955-762 | Rodentia | Muridae | Acomys airensis | 17.873611 | -12.331667 | K6 |
| http://coldb.mnhn.fr/catalognumber/mnhn/zm/mo-1955-761 | Rodentia | Muridae | Acomys airensis | 17.873611 | -12.331667 | K6 |
| http://coldb.mnhn.fr/catalognumber/mnhn/zm/mo-1955-760 | Rodentia | Muridae | Acomys airensis | 17.873611 | -12.331667 | K6 |
| http://n2t.net/ark:/65665/31f8d0996-acc9-4ca7-803f-de7e0c94527c | Rodentia | Muridae | Acomys airensis | 20.550000 | -12.500000 | H6 |
| http://n2t.net/ark:/65665/3c8a99df9-0533-4bf6-ba38-aa1241598216 | Rodentia | Muridae | Acomys airensis | 20.550000 | -12.500000 | H6 |
| http://n2t.net/ark:/65665/386b48fce-b5d7-4df5-b3f4-86f42a550d49 | Rodentia | Muridae | Acomys airensis | 20.550000 | -12.500000 | H6 |
| http://n2t.net/ark:/65665/33b96ff0d-b458-4354-8c54-550d56e7145c | Rodentia | Muridae | Acomys airensis | 20.550000 | -12.500000 | H6 |
| http://coldb.mnhn.fr/catalognumber/mnhn/zm/2004-138 | Rodentia | Muridae | Acomys airensis | 16.417000 | -9.633000 | M9 |
| http://coldb.mnhn.fr/catalognumber/mnhn/zm/2006-180 | Rodentia | Muridae | Acomys airensis | 16.680556 | -9.301944 | L10 |
| http://n2t.net/ark:/65665/3a6670167-8b32-46c8-b73d-d6bc84d93c24 | Rodentia | Muridae | Acomys airensis | 20.536743 | -12.961179 | H6 |
| NMR999000000865 | Rodentia | Muridae | Arvicanthis niloticus | 18.115564 | -16.015721 | K3 |
| NMR999000000864 | Rodentia | Muridae | Arvicanthis niloticus | 18.115564 | -16.015721 | K3 |
| RMCA:Rodentia:63783 | Rodentia | Muridae | Arvicanthis niloticus | 16.166700 | -13.416700 | M5 |
| RMCA:Rodentia:63555 | Rodentia | Muridae | Arvicanthis niloticus | 16.166700 | -13.416700 | M5 |
| RMCA:Rodentia:63554 | Rodentia | Muridae | Arvicanthis niloticus | 16.166700 | -13.416700 | M5 |
| RMCA:Rodentia:63553 | Rodentia | Muridae | Arvicanthis niloticus | 16.166700 | -13.416700 | M5 |
| RMCA:Rodentia:63552 | Rodentia | Muridae | Arvicanthis niloticus | 16.166700 | -13.416700 | M5 |
| RMCA:Rodentia:63551 | Rodentia | Muridae | Arvicanthis niloticus | 16.166700 | -13.416700 | M5 |
| RMCA:Rodentia:63550 | Rodentia | Muridae | Arvicanthis niloticus | 16.166700 | -13.416700 | M5 |
| RMCA:Rodentia:63549 | Rodentia | Muridae | Arvicanthis niloticus | 16.166700 | -13.416700 | M5 |
| RMCA:Rodentia:63548 | Rodentia | Muridae | Arvicanthis niloticus | 16.166700 | -13.416700 | M5 |
| RMCA:Rodentia:63547 | Rodentia | Muridae | Arvicanthis niloticus | 16.166700 | -13.416700 | M5 |
| RMCA:Rodentia:63546 | Rodentia | Muridae | Arvicanthis niloticus | 16.166700 | -13.416700 | M5 |
| RMCA:Rodentia:63545 | Rodentia | Muridae | Arvicanthis niloticus | 16.166700 | -13.416700 | M5 |
| RMCA:Rodentia:63544 | Rodentia | Muridae | Arvicanthis niloticus | 16.166700 | -13.416700 | M5 |
| RMCA:Rodentia:63543 | Rodentia | Muridae | Arvicanthis niloticus | 16.166700 | -13.416700 | M5 |
| RMCA:Rodentia:63542 | Rodentia | Muridae | Arvicanthis niloticus | 16.166700 | -13.416700 | M5 |
| RMCA:Rodentia:63541 | Rodentia | Muridae | Arvicanthis niloticus | 16.166700 | -13.416700 | M5 |
| RMCA:Rodentia:63540 | Rodentia | Muridae | Arvicanthis niloticus | 16.166700 | -13.416700 | M5 |
| http://n2t.net/ark:/65665/3f6f5ecec-ac2a-47aa-b97d-09a98acbcc31 | Rodentia | Muridae | Arvicanthis niloticus | 16.550000 | -15.766667 | M3 |
| http://n2t.net/ark:/65665/34a782503-423f-4642-8e69-e267a9878d61 | Rodentia | Muridae | Arvicanthis niloticus | 16.550000 | -15.766667 | M3 |
| http://n2t.net/ark:/65665/35b520a64-aaf5-4dfa-8473-ad34e11b89ad | Rodentia | Muridae | Arvicanthis niloticus | 16.550000 | -15.766667 | M3 |
| http://n2t.net/ark:/65665/38fce9b0e-af08-43ec-8b61-f2d3174751ee | Rodentia | Muridae | Arvicanthis niloticus | 16.550000 | -15.766667 | M3 |
| http://n2t.net/ark:/65665/314c1e87d-0994-432a-aad0-03c2f70ca78c | Rodentia | Muridae | Arvicanthis niloticus | 16.550000 | -15.766667 | M3 |
| http://n2t.net/ark:/65665/36267db63-8062-415c-ba60-094786872751 | Rodentia | Muridae | Arvicanthis niloticus | 16.550000 | -15.766667 | M3 |
| http://n2t.net/ark:/65665/3a4e7bcba-6069-4256-88c4-e70d7b8aad31 | Rodentia | Muridae | Arvicanthis niloticus | 16.550000 | -15.766667 | M3 |
| http://n2t.net/ark:/65665/37b5e7c65-da9b-4509-8385-61b69f44e171 | Rodentia | Muridae | Arvicanthis niloticus | 16.550000 | -15.766667 | M3 |
| http://n2t.net/ark:/65665/311ae38f3-99cc-4e1a-a189-7ef152fda7f7 | Rodentia | Muridae | Arvicanthis niloticus | 16.550000 | -15.766667 | M3 |
| http://n2t.net/ark:/65665/3cdf4154d-bb36-4c92-ba76-4000a9c2d50f | Rodentia | Muridae | Arvicanthis niloticus | 16.550000 | -15.766667 | M3 |
| http://n2t.net/ark:/65665/32caddbc5-671c-48bb-9f87-9b9f33bbcd61 | Rodentia | Muridae | Arvicanthis niloticus | 16.550000 | -15.766667 | M3 |
| http://n2t.net/ark:/65665/312762284-a60d-4d4e-87e7-fe937f55c3c6 | Rodentia | Muridae | Arvicanthis niloticus | 16.550000 | -15.766667 | M3 |
| http://n2t.net/ark:/65665/393b8a49c-775d-46ca-831e-23e6e57f514a | Rodentia | Muridae | Arvicanthis niloticus | 16.550000 | -15.766667 | M3 |
| http://n2t.net/ark:/65665/3a1f2f27f-92ae-4583-a756-447781af4f5e | Rodentia | Muridae | Arvicanthis niloticus | 16.550000 | -15.766667 | M3 |
| http://n2t.net/ark:/65665/3ae8e47fb-515e-4653-97d4-955f03904665 | Rodentia | Muridae | Arvicanthis niloticus | 16.550000 | -15.766667 | M3 |
| http://n2t.net/ark:/65665/3c5b1dad0-764a-48f3-bf9f-c9214a5d7a13 | Rodentia | Muridae | Arvicanthis niloticus | 16.550000 | -15.766667 | M3 |
| http://n2t.net/ark:/65665/3fa021e40-22a9-4f0d-b751-a6ff3c1f825f | Rodentia | Muridae | Arvicanthis niloticus | 16.550000 | -15.766667 | M3 |
| http://n2t.net/ark:/65665/3fc46c5f4-22c7-4608-a8bf-554a4c33f8f6 | Rodentia | Muridae | Arvicanthis niloticus | 16.550000 | -15.766667 | M3 |
| http://n2t.net/ark:/65665/33459a04d-2ca2-4d84-a1d2-0198ac2d540c | Rodentia | Muridae | Arvicanthis niloticus | 16.550000 | -15.766667 | M3 |
| http://n2t.net/ark:/65665/3b16be1d7-812a-4e1e-ba2a-0bbbfb1a759b | Rodentia | Muridae | Arvicanthis niloticus | 16.550000 | -15.766667 | M3 |
| http://n2t.net/ark:/65665/300dc8e6c-cecd-474c-81da-30c60d575600 | Rodentia | Muridae | Arvicanthis niloticus | 16.550000 | -15.766667 | M3 |
| http://n2t.net/ark:/65665/3af0b2f6a-dfe2-4198-ab2c-bd390529bcaa | Rodentia | Muridae | Arvicanthis niloticus | 16.550000 | -15.766667 | M3 |
| http://n2t.net/ark:/65665/303412dd5-ce78-4240-8035-70378297ec2b | Rodentia | Muridae | Arvicanthis niloticus | 16.550000 | -15.766667 | M3 |
| http://n2t.net/ark:/65665/3a8fd52a2-8d93-42ac-b521-9b2a7f9d1657 | Rodentia | Muridae | Arvicanthis niloticus | 16.550000 | -15.766667 | M3 |
| http://n2t.net/ark:/65665/3be116963-dcc1-4345-88e8-b373449a467a | Rodentia | Muridae | Arvicanthis niloticus | 16.550000 | -15.766667 | M3 |
| http://n2t.net/ark:/65665/33b5c16f7-24e1-4331-959e-cbbef0df4c79 | Rodentia | Muridae | Arvicanthis niloticus | 16.550000 | -15.766667 | M3 |
| http://n2t.net/ark:/65665/365a64886-898a-4f6f-aca6-b121c742b862 | Rodentia | Muridae | Arvicanthis niloticus | 16.550000 | -15.766667 | M3 |
| http://n2t.net/ark:/65665/36cc54b5e-3bf9-4bc7-b108-85bf1c329cca | Rodentia | Muridae | Arvicanthis niloticus | 16.550000 | -15.766667 | M3 |
| http://n2t.net/ark:/65665/3bd35cf97-0669-43ee-8393-b2a591ef9234 | Rodentia | Muridae | Arvicanthis niloticus | 16.550000 | -15.766667 | M3 |
| http://n2t.net/ark:/65665/32da7052f-d1e7-4921-9104-07928148ede4 | Rodentia | Muridae | Arvicanthis niloticus | 16.550000 | -15.766667 | M3 |
| http://n2t.net/ark:/65665/32556dec9-6391-464a-98aa-80e718b69440 | Rodentia | Muridae | Arvicanthis niloticus | 16.550000 | -15.766667 | M3 |
| http://n2t.net/ark:/65665/32aed80be-4830-4191-8577-6e90826fdea4 | Rodentia | Muridae | Arvicanthis niloticus | 16.550000 | -15.766667 | M3 |
| http://n2t.net/ark:/65665/3aab613a8-44e5-4064-a363-45d59953d585 | Rodentia | Muridae | Arvicanthis niloticus | 16.550000 | -15.766667 | M3 |
| http://n2t.net/ark:/65665/32e705027-d9a8-40ff-9774-fe808bb93435 | Rodentia | Muridae | Arvicanthis niloticus | 16.550000 | -15.766667 | M3 |
| http://coldb.mnhn.fr/catalognumber/mnhn/zm/mo-2001-38 | Rodentia | Muridae | Arvicanthis niloticus | 16.634000 | -15.483000 | L3 |
| http://coldb.mnhn.fr/catalognumber/mnhn/zm/mo-2001-37 | Rodentia | Muridae | Arvicanthis niloticus | 16.634000 | -15.483000 | L3 |
| http://coldb.mnhn.fr/catalognumber/mnhn/zm/mo-2001-39 | Rodentia | Muridae | Arvicanthis niloticus | 19.033000 | -16.231000 | J2 |
| http://coldb.mnhn.fr/catalognumber/mnhn/zm/mo-2001-35 | Rodentia | Muridae | Arvicanthis niloticus | 16.607000 | -16.439323 | M2 |
| http://coldb.mnhn.fr/catalognumber/mnhn/zm/mo-2001-33 | Rodentia | Muridae | Arvicanthis niloticus | 16.607000 | -16.439323 | M2 |
| http://coldb.mnhn.fr/catalognumber/mnhn/zm/mo-2001-34 | Rodentia | Muridae | Arvicanthis niloticus | 16.607000 | -16.439323 | M2 |
| http://coldb.mnhn.fr/catalognumber/mnhn/zm/mo-2001-32 | Rodentia | Muridae | Arvicanthis niloticus | 15.007120 | -12.254392 | N7 |
| http://coldb.mnhn.fr/catalognumber/mnhn/zm/mo-2001-31 | Rodentia | Muridae | Arvicanthis niloticus | 15.007120 | -12.254392 | N7 |
| http://coldb.mnhn.fr/catalognumber/mnhn/zm/mo-2001-30 | Rodentia | Muridae | Arvicanthis niloticus | 15.007120 | -12.254392 | N7 |
| http://n2t.net/ark:/65665/37658ef7b-3412-4210-9fed-67fc35fda392 | Rodentia | Muridae | Arvicanthis niloticus | 16.650000 | -14.283333 | L4 |
| http://n2t.net/ark:/65665/37a6ec5c2-28ee-4e9c-a55e-df2ec86c68e2 | Rodentia | Muridae | Arvicanthis niloticus | 16.650000 | -14.283333 | L4 |
| http://n2t.net/ark:/65665/3f899755f-27b2-4cd4-9503-0a10eeadfacd | Rodentia | Muridae | Arvicanthis niloticus | 16.650000 | -14.283333 | L4 |
| http://n2t.net/ark:/65665/31a78b249-817c-43f8-b391-845a495f3e63 | Rodentia | Muridae | Arvicanthis niloticus | 16.650000 | -14.283333 | L4 |
| http://n2t.net/ark:/65665/3355c186b-80c1-4549-9cc4-d29154e9d189 | Rodentia | Muridae | Arvicanthis niloticus | 16.650000 | -14.283333 | L4 |
| http://n2t.net/ark:/65665/344e64f61-507d-4c56-beb6-6cbaf62cae54 | Rodentia | Muridae | Arvicanthis niloticus | 16.650000 | -14.283333 | L4 |
| http://n2t.net/ark:/65665/3078f47eb-41de-4593-b11f-8a9b8a5b9c6d | Rodentia | Muridae | Arvicanthis niloticus | 16.650000 | -14.283333 | L4 |
| http://n2t.net/ark:/65665/324073973-99a1-4e6f-b975-364b465455c6 | Rodentia | Muridae | Arvicanthis niloticus | 16.650000 | -14.283333 | L4 |
| http://n2t.net/ark:/65665/3345130cb-9ac2-4265-9c7c-bcfc4abe9cb2 | Rodentia | Muridae | Arvicanthis niloticus | 16.650000 | -14.283333 | L4 |
| http://n2t.net/ark:/65665/30ba33bc7-7c81-438b-be78-83a06cd1b2e5 | Rodentia | Muridae | Arvicanthis niloticus | 16.650000 | -14.283333 | L4 |
| http://n2t.net/ark:/65665/3e437997d-b8aa-4616-ad52-fb2582d72e1b | Rodentia | Muridae | Arvicanthis niloticus | 16.650000 | -14.283333 | L4 |
| http://n2t.net/ark:/65665/305fc8881-290c-4255-bdc8-425492bc0c78 | Rodentia | Muridae | Arvicanthis niloticus | 16.650000 | -14.283333 | L4 |
| http://n2t.net/ark:/65665/3b57b72fd-00b1-42cc-91bf-870519576adf | Rodentia | Muridae | Arvicanthis niloticus | 16.650000 | -14.283333 | L4 |
| http://n2t.net/ark:/65665/3b607ed9d-8ac8-4a75-9c9e-54e53bb107eb | Rodentia | Muridae | Arvicanthis niloticus | 16.650000 | -14.283333 | L4 |
| http://n2t.net/ark:/65665/3eff7b527-1318-4e47-a446-7896ad4e920b | Rodentia | Muridae | Arvicanthis niloticus | 16.650000 | -14.283333 | L4 |
| http://n2t.net/ark:/65665/36c9a0e13-91b9-4ffd-af36-37435e5f2a69 | Rodentia | Muridae | Arvicanthis niloticus | 16.650000 | -14.283333 | L4 |
| http://n2t.net/ark:/65665/379c43551-61d3-4bc8-b613-a6d6a2b0f7f9 | Rodentia | Muridae | Arvicanthis niloticus | 16.650000 | -14.283333 | L4 |
| http://n2t.net/ark:/65665/3878be9dd-d2a3-4c64-9651-22a0acb3c4d2 | Rodentia | Muridae | Arvicanthis niloticus | 16.650000 | -14.283333 | L4 |
| http://n2t.net/ark:/65665/33165ec24-9fb4-4fda-953b-0b6a708c42f4 | Rodentia | Muridae | Arvicanthis niloticus | 16.650000 | -14.283333 | L4 |
| http://n2t.net/ark:/65665/38259a8e9-ca65-4bcb-95c3-c4a1069e49ad | Rodentia | Muridae | Arvicanthis niloticus | 16.650000 | -14.283333 | L4 |
| http://n2t.net/ark:/65665/33a367ec7-ecd9-4308-8639-76b426b38c1f | Rodentia | Muridae | Arvicanthis niloticus | 16.650000 | -14.283333 | L4 |
| http://n2t.net/ark:/65665/3e0b74ee7-6c16-4bc6-be3a-63a026de529c | Rodentia | Muridae | Arvicanthis niloticus | 16.650000 | -14.283333 | L4 |
| http://n2t.net/ark:/65665/336767f5f-0508-4e8e-823f-aba64adc07bf | Rodentia | Muridae | Arvicanthis niloticus | 16.650000 | -14.283333 | L4 |
| http://n2t.net/ark:/65665/3b3f74885-7619-44b0-81aa-c64674f416ce | Rodentia | Muridae | Arvicanthis niloticus | 16.650000 | -14.283333 | L4 |
| http://n2t.net/ark:/65665/39150690c-196c-4250-9a23-b99ba5f7e7e3 | Rodentia | Muridae | Arvicanthis niloticus | 16.650000 | -14.283333 | L4 |
| http://n2t.net/ark:/65665/3d2b66a02-3c0c-40f9-9de6-9d3a1ea159a2 | Rodentia | Muridae | Arvicanthis niloticus | 16.650000 | -14.283333 | L4 |
| http://n2t.net/ark:/65665/3fc1bba92-01ca-48d9-b7bf-fcd752fd6cb2 | Rodentia | Muridae | Arvicanthis niloticus | 16.650000 | -14.283333 | L4 |
| http://n2t.net/ark:/65665/394a3826c-bbe9-48b7-afc1-4767315f67cc | Rodentia | Muridae | Arvicanthis niloticus | 16.650000 | -14.283333 | L4 |
| http://n2t.net/ark:/65665/3d548cd7e-3667-4e1c-90f5-ed85c41310d6 | Rodentia | Muridae | Arvicanthis niloticus | 17.030000 | -13.920000 | L5 |
| http://n2t.net/ark:/65665/3f9947a70-1ca9-4669-9965-1c8a2d5e2c31 | Rodentia | Muridae | Arvicanthis niloticus | 17.030000 | -13.920000 | L5 |
| http://n2t.net/ark:/65665/37a602b5e-8163-4aca-8054-e5dd6359caf8 | Rodentia | Muridae | Arvicanthis niloticus | 17.030000 | -13.920000 | L5 |
| http://n2t.net/ark:/65665/3ffab61f2-e498-4dc6-87ac-26a11982d8a6 | Rodentia | Muridae | Arvicanthis niloticus | 17.030000 | -13.920000 | L5 |
| http://n2t.net/ark:/65665/35e45fb6d-be51-472a-b845-a55eff571862 | Rodentia | Muridae | Arvicanthis niloticus | 17.030000 | -13.920000 | L5 |
| http://n2t.net/ark:/65665/3591e34f8-8f39-4d8b-850e-50eff5a80eba | Rodentia | Muridae | Arvicanthis niloticus | 17.030000 | -13.920000 | L5 |
| http://n2t.net/ark:/65665/32b0426ad-26ae-4e87-8cc4-790fa3d878b8 | Rodentia | Muridae | Arvicanthis niloticus | 17.030000 | -13.920000 | L5 |
| http://coldb.mnhn.fr/catalognumber/mnhn/zm/mo-2001-750 | Rodentia | Muridae | Desmodilliscus braueri | 16.583000 | -15.417000 | M3 |
| http://n2t.net/ark:/65665/393be69b0-c40d-4bbf-90f1-faaef28f49fc | Rodentia | Muridae | Desmodilliscus braueri | 16.970000 | -13.930000 | L5 |
| http://n2t.net/ark:/65665/32a134b36-f5b6-4329-9d72-a030d2e02b6f | Rodentia | Muridae | Desmodilliscus braueri | 17.030000 | -13.920000 | L5 |
| http://n2t.net/ark:/65665/391944d22-c345-4f02-8db2-151460915dc9 | Rodentia | Muridae | Desmodilliscus braueri | 17.030000 | -13.920000 | L5 |
| http://n2t.net/ark:/65665/35dbaa7a6-d269-466e-b3ef-267e6f907886 | Rodentia | Muridae | Desmodilliscus braueri | 17.030000 | -13.920000 | L5 |
| http://n2t.net/ark:/65665/335f32b73-0d74-4ee9-b24c-f98f13cb034a | Rodentia | Muridae | Desmodilliscus braueri | 17.030000 | -13.920000 | L5 |
| http://n2t.net/ark:/65665/328cbb382-a6b1-4e61-9839-9362fd548cc3 | Rodentia | Muridae | Desmodilliscus braueri | 17.030000 | -13.920000 | L5 |
| http://n2t.net/ark:/65665/3679bd1ad-05b0-40ca-9df0-1319a71316b4 | Rodentia | Muridae | Desmodilliscus braueri | 17.030000 | -13.920000 | L5 |
| http://n2t.net/ark:/65665/3163937cf-410e-43a6-b23a-c4c71633ba03 | Rodentia | Muridae | Desmodilliscus braueri | 17.030000 | -13.920000 | L5 |
| http://n2t.net/ark:/65665/35f9e0f08-1a6a-4923-ad3e-eabf7f6ca288 | Rodentia | Muridae | Desmodilliscus braueri | 17.030000 | -13.920000 | L5 |
| http://n2t.net/ark:/65665/366ac4949-7667-4eaa-be51-895c764588f5 | Rodentia | Muridae | Desmodilliscus braueri | 17.030000 | -13.920000 | L5 |
| http://n2t.net/ark:/65665/3a1ce9f5d-eaf5-4651-ba71-2086440af1c5 | Rodentia | Muridae | Desmodilliscus braueri | 17.030000 | -13.920000 | L5 |
| http://n2t.net/ark:/65665/3ef805e1f-3317-48fd-95f7-74b3963b798a | Rodentia | Muridae | Desmodilliscus braueri | 17.030000 | -13.920000 | L5 |
| http://n2t.net/ark:/65665/39cbda826-69e5-4ccb-886b-653a90d786b4 | Rodentia | Muridae | Desmodilliscus braueri | 17.030000 | -13.920000 | L5 |
| http://n2t.net/ark:/65665/3cca39d41-fcfb-4dc9-99ba-64d75924115e | Rodentia | Muridae | Desmodilliscus braueri | 17.030000 | -13.920000 | L5 |
| http://n2t.net/ark:/65665/380922768-6835-46bf-938b-b2e53705b369 | Rodentia | Muridae | Desmodilliscus braueri | 17.030000 | -13.920000 | L5 |
| http://n2t.net/ark:/65665/3a7d9c823-0555-40fa-86f9-6b8105f19286 | Rodentia | Muridae | Desmodilliscus braueri | 17.030000 | -13.920000 | L5 |
| http://n2t.net/ark:/65665/3267711d0-59e9-4064-8732-2254c5ca8ed2 | Rodentia | Muridae | Desmodilliscus braueri | 17.030000 | -13.920000 | L5 |
| http://n2t.net/ark:/65665/360eb49bf-7be9-4c26-be1d-8d70b8994dae | Rodentia | Muridae | Desmodilliscus braueri | 17.030000 | -13.920000 | L5 |
| http://n2t.net/ark:/65665/386810734-9a0f-41b8-abb0-0e1f21204f14 | Rodentia | Muridae | Desmodilliscus braueri | 17.030000 | -13.920000 | L5 |
| http://n2t.net/ark:/65665/3e4931f04-e2bf-4206-bd75-dcf4ab99667f | Rodentia | Muridae | Desmodilliscus braueri | 17.030000 | -13.920000 | L5 |
| http://n2t.net/ark:/65665/35d0c26d8-167e-4f92-8899-7465514125c3 | Rodentia | Muridae | Desmodilliscus braueri | 17.030000 | -13.920000 | L5 |
| http://n2t.net/ark:/65665/366d4f44b-a6f5-4407-affd-4ae48f4f2cfc | Rodentia | Muridae | Desmodilliscus braueri | 17.030000 | -13.920000 | L5 |
| http://n2t.net/ark:/65665/33ab87957-f839-4435-b420-10085cb9b71c | Rodentia | Muridae | Desmodilliscus braueri | 17.030000 | -13.920000 | L5 |
| http://n2t.net/ark:/65665/3b9f9e04b-061f-40a1-8509-1524a8bf6827 | Rodentia | Muridae | Desmodilliscus braueri | 17.030000 | -13.920000 | L5 |
| http://n2t.net/ark:/65665/3896063b9-adaa-414e-922a-1cc1c3370a11 | Rodentia | Muridae | Desmodilliscus braueri | 17.030000 | -13.920000 | L5 |
| http://n2t.net/ark:/65665/31bb9f17c-6061-4a2f-919b-73ea72ad1654 | Rodentia | Muridae | Desmodilliscus braueri | 17.030000 | -13.920000 | L5 |
| http://n2t.net/ark:/65665/385250db1-dcb4-44ec-9eaf-7c958ae770da | Rodentia | Muridae | Desmodilliscus braueri | 17.030000 | -13.920000 | L5 |
| http://n2t.net/ark:/65665/3f2520ce7-d876-4b46-a71b-a048fae7db72 | Rodentia | Muridae | Desmodilliscus braueri | 17.030000 | -13.920000 | L5 |
| http://n2t.net/ark:/65665/3d8f6ee64-98b8-4d4e-8388-2360feb37d43 | Rodentia | Muridae | Desmodilliscus braueri | 17.030000 | -13.920000 | L5 |
| http://n2t.net/ark:/65665/384a88008-8df8-48c3-a6ae-fbed917f8b86 | Rodentia | Muridae | Desmodilliscus braueri | 17.030000 | -13.920000 | L5 |
| http://n2t.net/ark:/65665/358d270d0-902b-4def-9da7-b90028b718f2 | Rodentia | Muridae | Desmodilliscus braueri | 17.030000 | -13.920000 | L5 |
| http://n2t.net/ark:/65665/3aca4e3dc-b168-45fa-b59b-91c99092482e | Rodentia | Muridae | Desmodilliscus braueri | 17.030000 | -13.920000 | L5 |
| http://n2t.net/ark:/65665/3699a5a98-f399-45dc-8c8e-f54df6879114 | Rodentia | Muridae | Desmodilliscus braueri | 17.030000 | -13.920000 | L5 |
| http://n2t.net/ark:/65665/3bd42f4c2-9600-4c92-b3cc-80508b33e6f4 | Rodentia | Muridae | Desmodilliscus braueri | 17.030000 | -13.920000 | L5 |
| http://n2t.net/ark:/65665/37af6254d-ea50-4b06-95c9-a0056292878d | Rodentia | Muridae | Desmodilliscus braueri | 16.800000 | -13.880000 | L5 |
| http://coldb.mnhn.fr/catalognumber/mnhn/zm/mo-1964-402 | Rodentia | Muridae | Desmodilliscus braueri | 20.527377 | -13.019975 | H6 |
| 62dde8e9-b988-49fb-bfbc-bf0924168914 | Rodentia | Muridae | Desmodilliscus braueri | 15.957322 | -12.107513 | M7 |
| 254f7fa4-28b4-45ed-a294-d7689f205bcc | Rodentia | Muridae | Desmodilliscus braueri | 15.259813 | -12.483478 | N6 |
| 8fd25140-87a3-4fbb-b178-720897b689cb | Rodentia | Muridae | Desmodilliscus braueri | 15.470327 | -12.887454 | N6 |
| 3eb913de-dbc1-43c0-82bb-ec51413df744 | Rodentia | Muridae | Desmodilliscus braueri | 18.356822 | -11.816107 | J7 |
| 913cc677-0843-436c-9bbe-598d111a531e | Rodentia | Muridae | Desmodilliscus braueri | 15.053715 | -11.855454 | N7 |
| 2c0436fc-b8aa-4d69-97fa-4ff12a208168 | Rodentia | Muridae | Desmodilliscus braueri | 15.095737 | -12.243834 | N7 |
| 4dca09a2-28b9-4afc-bbc4-8668c1c7f080 | Rodentia | Muridae | Desmodilliscus braueri | 16.275675 | -13.703754 | M5 |
| 93607946-90c7-4881-9f17-50b1a58d19b0 | Rodentia | Muridae | Desmodilliscus braueri | 16.275675 | -13.703754 | M5 |
| 7fc73ce0-1de8-4d42-a07c-ac00a103989b | Rodentia | Muridae | Desmodilliscus braueri | 15.025799 | -12.183352 | N7 |
| 918de94f-944f-4d90-bbd3-8c8a71775989 | Rodentia | Muridae | Desmodilliscus braueri | 16.257035 | -13.658015 | M5 |
| 777ea2c2-03b1-4655-a4b5-8fb90709adfc | Rodentia | Muridae | Desmodilliscus braueri | 16.257035 | -13.658015 | M5 |
| c821acfe-b009-4d46-a5fb-86a90f9c76e4 | Rodentia | Muridae | Desmodilliscus braueri | 16.257035 | -13.658015 | M5 |
| http://coldb.mnhn.fr/catalognumber/mnhn/zm/mo-1997-1437 | Rodentia | Muridae | Gerbillus amoenus | 18.836000 | -16.133100 | J2 |
| http://coldb.mnhn.fr/catalognumber/mnhn/zm/mo-2001-1676 | Rodentia | Muridae | Gerbillus amoenus | 18.694000 | -16.102720 | J3 |
| http://coldb.mnhn.fr/catalognumber/mnhn/zm/mo-1997-1444 | Rodentia | Muridae | Gerbillus amoenus | 18.694000 | -16.102720 | J3 |
| http://coldb.mnhn.fr/catalognumber/mnhn/zm/mo-1997-1439 | Rodentia | Muridae | Gerbillus amoenus | 18.642000 | -16.106000 | J3 |
| http://coldb.mnhn.fr/catalognumber/mnhn/zm/mo-2001-364 | Rodentia | Muridae | Gerbillus amoenus | 17.636000 | -16.011100 | K3 |
| http://coldb.mnhn.fr/catalognumber/mnhn/zm/mo-1997-1436 | Rodentia | Muridae | Gerbillus amoenus | 17.636000 | -16.011100 | K3 |
| http://coldb.mnhn.fr/catalognumber/mnhn/zm/mo-2001-276 | Rodentia | Muridae | Gerbillus amoenus | 17.600000 | -16.018600 | K3 |
| http://coldb.mnhn.fr/catalognumber/mnhn/zm/mo-2001-271 | Rodentia | Muridae | Gerbillus amoenus | 17.600000 | -16.018600 | K3 |
| http://coldb.mnhn.fr/catalognumber/mnhn/zm/mo-2001-1674 | Rodentia | Muridae | Gerbillus amoenus | 17.600000 | -16.018600 | K3 |
| http://coldb.mnhn.fr/catalognumber/mnhn/zm/mo-1997-1447 | Rodentia | Muridae | Gerbillus amoenus | 17.517000 | -16.033300 | K3 |
| http://coldb.mnhn.fr/catalognumber/mnhn/zm/mo-1997-1446 | Rodentia | Muridae | Gerbillus amoenus | 17.517000 | -16.033300 | K3 |
| http://coldb.mnhn.fr/catalognumber/mnhn/zm/mo-1997-1445 | Rodentia | Muridae | Gerbillus amoenus | 17.517000 | -16.033300 | K3 |
| http://coldb.mnhn.fr/catalognumber/mnhn/zm/mo-2001-224 | Rodentia | Muridae | Gerbillus amoenus | 18.703000 | -16.030000 | J3 |
| http://coldb.mnhn.fr/catalognumber/mnhn/zm/mo-2001-223 | Rodentia | Muridae | Gerbillus amoenus | 18.703000 | -16.030000 | J3 |
| JQ753059 | Rodentia | Muridae | Gerbillus amoenus | 18.801000 | -16.141000 | J2 |
| http://coldb.mnhn.fr/catalognumber/mnhn/zm/mo-1997-1454 | Rodentia | Muridae | Gerbillus amoenus | 18.267000 | -16.016900 | K3 |
| http://coldb.mnhn.fr/catalognumber/mnhn/zm/mo-1997-1453 | Rodentia | Muridae | Gerbillus amoenus | 18.267000 | -16.016900 | K3 |
| http://coldb.mnhn.fr/catalognumber/mnhn/zm/mo-1997-1452 | Rodentia | Muridae | Gerbillus amoenus | 18.267000 | -16.016900 | K3 |
| http://coldb.mnhn.fr/catalognumber/mnhn/zm/mo-1998-1269 | Rodentia | Muridae | Gerbillus amoenus | 18.115564 | -16.015721 | K3 |
| http://coldb.mnhn.fr/catalognumber/mnhn/zm/mo-1988-303 | Rodentia | Muridae | Gerbillus amoenus | 18.115564 | -16.015721 | K3 |
| http://coldb.mnhn.fr/catalognumber/mnhn/zm/mo-1988-302 | Rodentia | Muridae | Gerbillus amoenus | 18.115564 | -16.015721 | K3 |
| http://coldb.mnhn.fr/catalognumber/mnhn/zm/mo-1983-818 | Rodentia | Muridae | Gerbillus amoenus | 18.115564 | -16.015721 | K3 |
| http://coldb.mnhn.fr/catalognumber/mnhn/zm/mo-2001-255bis | Rodentia | Muridae | Gerbillus amoenus | 16.558000 | -16.224000 | M2 |
| http://coldb.mnhn.fr/catalognumber/mnhn/zm/mo-2001-255 | Rodentia | Muridae | Gerbillus amoenus | 16.558000 | -16.224000 | M2 |
| http://coldb.mnhn.fr/catalognumber/mnhn/zm/mo-2001-253 | Rodentia | Muridae | Gerbillus amoenus | 16.558000 | -16.224000 | M2 |
| http://coldb.mnhn.fr/catalognumber/mnhn/zm/mo-2001-252 | Rodentia | Muridae | Gerbillus amoenus | 16.558000 | -16.224000 | M2 |
| http://coldb.mnhn.fr/catalognumber/mnhn/zm/mo-2001-251 | Rodentia | Muridae | Gerbillus amoenus | 16.558000 | -16.224000 | M2 |
| http://coldb.mnhn.fr/catalognumber/mnhn/zm/mo-1997-1438 | Rodentia | Muridae | Gerbillus amoenus | 18.333000 | -16.033000 | K3 |
| http://coldb.mnhn.fr/catalognumber/mnhn/zm/mo-1997-1428 | Rodentia | Muridae | Gerbillus amoenus | 20.050000 | -16.217000 | I2 |
| http://coldb.mnhn.fr/catalognumber/mnhn/zm/mo-1997-1442 | Rodentia | Muridae | Gerbillus amoenus | 19.083330 | -16.233300 | J2 |
| http://coldb.mnhn.fr/catalognumber/mnhn/zm/mo-2001-94 | Rodentia | Muridae | Gerbillus amoenus | 19.033000 | -16.231000 | J2 |
| http://coldb.mnhn.fr/catalognumber/mnhn/zm/mo-2001-739 | Rodentia | Muridae | Gerbillus amoenus | 16.386000 | -16.467000 | M2 |
| http://coldb.mnhn.fr/catalognumber/mnhn/zm/mo-2001-738 | Rodentia | Muridae | Gerbillus amoenus | 16.386000 | -16.467000 | M2 |
| http://coldb.mnhn.fr/catalognumber/mnhn/zm/mo-2001-737 | Rodentia | Muridae | Gerbillus amoenus | 16.386000 | -16.467000 | M2 |
| http://coldb.mnhn.fr/catalognumber/mnhn/zm/mo-2001-736 | Rodentia | Muridae | Gerbillus amoenus | 16.386000 | -16.467000 | M2 |
| http://coldb.mnhn.fr/catalognumber/mnhn/zm/mo-1997-1450 | Rodentia | Muridae | Gerbillus amoenus | 16.386000 | -16.467000 | M2 |
| http://coldb.mnhn.fr/catalognumber/mnhn/zm/mo-1997-1455 | Rodentia | Muridae | Gerbillus amoenus | 16.375000 | -16.466000 | M2 |
| http://coldb.mnhn.fr/catalognumber/mnhn/zm/mo-2001-735 | Rodentia | Muridae | Gerbillus amoenus | 16.372000 | -16.462000 | M2 |
| http://coldb.mnhn.fr/catalognumber/mnhn/zm/mo-2001-734 | Rodentia | Muridae | Gerbillus amoenus | 16.372000 | -16.462000 | M2 |
| http://coldb.mnhn.fr/catalognumber/mnhn/zm/mo-2001-733 | Rodentia | Muridae | Gerbillus amoenus | 16.372000 | -16.462000 | M2 |
| http://coldb.mnhn.fr/catalognumber/mnhn/zm/mo-1997-1451 | Rodentia | Muridae | Gerbillus amoenus | 16.372000 | -16.462000 | M2 |
| http://coldb.mnhn.fr/catalognumber/mnhn/zm/mo-2001-747 | Rodentia | Muridae | Gerbillus amoenus | 16.747000 | -16.383000 | L2 |
| http://coldb.mnhn.fr/catalognumber/mnhn/zm/mo-2001-746 | Rodentia | Muridae | Gerbillus amoenus | 16.747000 | -16.383000 | L2 |
| http://coldb.mnhn.fr/catalognumber/mnhn/zm/mo-2001-745 | Rodentia | Muridae | Gerbillus amoenus | 16.747000 | -16.383000 | L2 |
| http://coldb.mnhn.fr/catalognumber/mnhn/zm/mo-2001-241 | Rodentia | Muridae | Gerbillus amoenus | 16.747000 | -16.383000 | L2 |
| http://coldb.mnhn.fr/catalognumber/mnhn/zm/mo-2001-237 | Rodentia | Muridae | Gerbillus amoenus | 16.747000 | -16.383000 | L2 |
| http://coldb.mnhn.fr/catalognumber/mnhn/zm/mo-2001-236 | Rodentia | Muridae | Gerbillus amoenus | 16.747000 | -16.383000 | L2 |
| http://coldb.mnhn.fr/catalognumber/mnhn/zm/mo-2001-235 | Rodentia | Muridae | Gerbillus amoenus | 16.747000 | -16.383000 | L2 |
| http://coldb.mnhn.fr/catalognumber/mnhn/zm/mo-1997-1449 | Rodentia | Muridae | Gerbillus amoenus | 16.747000 | -16.383000 | L2 |
| http://coldb.mnhn.fr/catalognumber/mnhn/zm/mo-1997-1448 | Rodentia | Muridae | Gerbillus amoenus | 16.747000 | -16.383000 | L2 |
| http://coldb.mnhn.fr/catalognumber/mnhn/zm/mo-2001-93 | Rodentia | Muridae | Gerbillus amoenus | 16.747000 | -16.383000 | L2 |
| http://coldb.mnhn.fr/catalognumber/mnhn/zm/mo-1997-1443 | Rodentia | Muridae | Gerbillus amoenus | 16.747000 | -16.383000 | L2 |
| http://coldb.mnhn.fr/catalognumber/mnhn/zm/mo-1997-1441 | Rodentia | Muridae | Gerbillus amoenus | 16.747000 | -16.383000 | L2 |
| http://coldb.mnhn.fr/catalognumber/mnhn/zm/mo-1997-1440 | Rodentia | Muridae | Gerbillus amoenus | 16.747000 | -16.383000 | L2 |
| http://coldb.mnhn.fr/catalognumber/mnhn/zm/mo-1997-1435 | Rodentia | Muridae | Gerbillus amoenus | 16.747000 | -16.383000 | L2 |
| http://coldb.mnhn.fr/catalognumber/mnhn/zm/mo-1997-1434 | Rodentia | Muridae | Gerbillus amoenus | 16.747000 | -16.383000 | L2 |
| http://coldb.mnhn.fr/catalognumber/mnhn/zm/mo-1997-1433 | Rodentia | Muridae | Gerbillus amoenus | 16.747000 | -16.383000 | L2 |
| http://coldb.mnhn.fr/catalognumber/mnhn/zm/mo-1997-1432 | Rodentia | Muridae | Gerbillus amoenus | 16.747000 | -16.383000 | L2 |
| http://coldb.mnhn.fr/catalognumber/mnhn/zm/mo-1997-1431 | Rodentia | Muridae | Gerbillus amoenus | 16.747000 | -16.383000 | L2 |
| http://coldb.mnhn.fr/catalognumber/mnhn/zm/mo-1997-1430 | Rodentia | Muridae | Gerbillus amoenus | 16.747000 | -16.383000 | L2 |
| http://coldb.mnhn.fr/catalognumber/mnhn/zm/mo-1997-1429 | Rodentia | Muridae | Gerbillus amoenus | 16.747000 | -16.383000 | L2 |
| http://coldb.mnhn.fr/catalognumber/mnhn/zm/mo-2001-713 | Rodentia | Muridae | Gerbillus amoenus | 16.747000 | -16.387000 | L2 |
| http://coldb.mnhn.fr/catalognumber/mnhn/zm/mo-2001-748 | Rodentia | Muridae | Gerbillus amoenus | 16.733000 | -16.378000 | L2 |
| http://coldb.mnhn.fr/catalognumber/mnhn/zm/mo-2001-459 | Rodentia | Muridae | Gerbillus amoenus | 16.733000 | -16.378000 | L2 |
| http://coldb.mnhn.fr/catalognumber/mnhn/zm/mo-2001-432 | Rodentia | Muridae | Gerbillus amoenus | 16.733000 | -16.378000 | L2 |
| http://coldb.mnhn.fr/catalognumber/mnhn/zm/mo-2001-431 | Rodentia | Muridae | Gerbillus amoenus | 16.733000 | -16.378000 | L2 |
| http://coldb.mnhn.fr/catalognumber/mnhn/zm/mo-2001-427 | Rodentia | Muridae | Gerbillus amoenus | 16.733000 | -16.378000 | L2 |
| http://coldb.mnhn.fr/catalognumber/mnhn/zm/mo-2001-426 | Rodentia | Muridae | Gerbillus amoenus | 16.733000 | -16.378000 | L2 |
| http://coldb.mnhn.fr/catalognumber/mnhn/zm/mo-2001-425 | Rodentia | Muridae | Gerbillus amoenus | 16.733000 | -16.378000 | L2 |
| http://coldb.mnhn.fr/catalognumber/mnhn/zm/mo-2001-423 | Rodentia | Muridae | Gerbillus amoenus | 16.733000 | -16.378000 | L2 |
| http://coldb.mnhn.fr/catalognumber/mnhn/zm/mo-2001-417 | Rodentia | Muridae | Gerbillus amoenus | 16.733000 | -16.378000 | L2 |
| http://coldb.mnhn.fr/catalognumber/mnhn/zm/mo-2001-416 | Rodentia | Muridae | Gerbillus amoenus | 16.733000 | -16.378000 | L2 |
| http://coldb.mnhn.fr/catalognumber/mnhn/zm/mo-2001-415 | Rodentia | Muridae | Gerbillus amoenus | 16.733000 | -16.378000 | L2 |
| http://coldb.mnhn.fr/catalognumber/mnhn/zm/mo-2001-414 | Rodentia | Muridae | Gerbillus amoenus | 16.733000 | -16.378000 | L2 |
| http://coldb.mnhn.fr/catalognumber/mnhn/zm/mo-2001-413 | Rodentia | Muridae | Gerbillus amoenus | 16.733000 | -16.378000 | L2 |
| http://coldb.mnhn.fr/catalognumber/mnhn/zm/mo-2001-412 | Rodentia | Muridae | Gerbillus amoenus | 16.733000 | -16.378000 | L2 |
| http://coldb.mnhn.fr/catalognumber/mnhn/zm/mo-2001-411 | Rodentia | Muridae | Gerbillus amoenus | 16.733000 | -16.378000 | L2 |
| http://coldb.mnhn.fr/catalognumber/mnhn/zm/mo-2001-409 | Rodentia | Muridae | Gerbillus amoenus | 16.733000 | -16.378000 | L2 |
| http://coldb.mnhn.fr/catalognumber/mnhn/zm/mo-2001-408 | Rodentia | Muridae | Gerbillus amoenus | 16.733000 | -16.378000 | L2 |
| http://coldb.mnhn.fr/catalognumber/mnhn/zm/mo-2001-406 | Rodentia | Muridae | Gerbillus amoenus | 16.733000 | -16.378000 | L2 |
| http://coldb.mnhn.fr/catalognumber/mnhn/zm/mo-2001-405 | Rodentia | Muridae | Gerbillus amoenus | 16.733000 | -16.378000 | L2 |
| http://coldb.mnhn.fr/catalognumber/mnhn/zm/mo-2001-404 | Rodentia | Muridae | Gerbillus amoenus | 16.733000 | -16.378000 | L2 |
| http://coldb.mnhn.fr/catalognumber/mnhn/zm/mo-2001-1675 | Rodentia | Muridae | Gerbillus amoenus | 16.733000 | -16.378000 | L2 |
| http://coldb.mnhn.fr/catalognumber/mnhn/zm/mo-2001-221 | Rodentia | Muridae | Gerbillus amoenus | 16.714000 | -16.401000 | L2 |
| http://coldb.mnhn.fr/catalognumber/mnhn/zm/mo-2001-220 | Rodentia | Muridae | Gerbillus amoenus | 16.714000 | -16.401000 | L2 |
| http://coldb.mnhn.fr/catalognumber/mnhn/zm/mo-2001-219 | Rodentia | Muridae | Gerbillus amoenus | 16.714000 | -16.401000 | L2 |
| http://coldb.mnhn.fr/catalognumber/mnhn/zm/mo-2001-218 | Rodentia | Muridae | Gerbillus amoenus | 16.714000 | -16.401000 | L2 |
| http://coldb.mnhn.fr/catalognumber/mnhn/zm/mo-2001-729 | Rodentia | Muridae | Gerbillus amoenus | 16.661000 | -16.424000 | L2 |
| http://coldb.mnhn.fr/catalognumber/mnhn/zm/mo-2001-728 | Rodentia | Muridae | Gerbillus amoenus | 16.661000 | -16.424000 | L2 |
| http://coldb.mnhn.fr/catalognumber/mnhn/zm/mo-2001-727 | Rodentia | Muridae | Gerbillus amoenus | 16.661000 | -16.424000 | L2 |
| http://coldb.mnhn.fr/catalognumber/mnhn/zm/mo-2001-615 | Rodentia | Muridae | Gerbillus amoenus | 16.600000 | -16.433000 | M2 |
| http://coldb.mnhn.fr/catalognumber/mnhn/zm/mo-1969-243 | Rodentia | Muridae | Gerbillus amoenus | 17.210000 | -14.180000 | L5 |
| http://coldb.mnhn.fr/catalognumber/mnhn/zm/mo-1969-245 | Rodentia | Muridae | Gerbillus amoenus | 17.210000 | -14.180000 | L5 |
| http://coldb.mnhn.fr/catalognumber/mnhn/zm/mo-1969-246 | Rodentia | Muridae | Gerbillus amoenus | 17.210000 | -14.180000 | L5 |
| http://coldb.mnhn.fr/catalognumber/mnhn/zm/mo-1969-244 | Rodentia | Muridae | Gerbillus amoenus | 17.210000 | -14.180000 | L5 |
| http://coldb.mnhn.fr/catalognumber/mnhn/zm/mo-2001-232 | Rodentia | Muridae | Gerbillus amoenus | 17.053000 | -16.263000 | L2 |
| http://coldb.mnhn.fr/catalognumber/mnhn/zm/mo-2001-242 | Rodentia | Muridae | Gerbillus amoenus | 16.875000 | -16.336000 | L2 |
| http://coldb.mnhn.fr/catalognumber/mnhn/zm/mo-1992-63 | Rodentia | Muridae | Gerbillus amoenus | 18.759760 | -16.151000 | J2 |
| http://coldb.mnhn.fr/catalognumber/mnhn/zm/mo-1988-301 | Rodentia | Muridae | Gerbillus amoenus | 18.759760 | -16.151000 | J2 |
| http://n2t.net/ark:/65665/367a176cf-6145-4001-9fd4-d027d755a3a1 | Rodentia | Muridae | Gerbillus campestris | 15.933333 | -12.016667 | M7 |
| http://n2t.net/ark:/65665/3ac47cfac-071d-497d-9750-bcdbf2066748 | Rodentia | Muridae | Gerbillus campestris | 15.933333 | -12.016667 | M7 |
| http://n2t.net/ark:/65665/39e0a77a5-a3a9-4537-8592-19262a234076 | Rodentia | Muridae | Gerbillus campestris | 15.933333 | -12.016667 | M7 |
| http://coldb.mnhn.fr/catalognumber/mnhn/zm/mo-1995-567 | Rodentia | Muridae | Gerbillus campestris | 19.831000 | -15.923000 | I3 |
| http://coldb.mnhn.fr/catalognumber/mnhn/zm/mo-1992-617 | Rodentia | Muridae | Gerbillus campestris | 15.694713 | -9.482034 | M9 |
| http://n2t.net/ark:/65665/33be1377b-fb7d-4ba1-a72f-04e8bd88d904 | Rodentia | Muridae | Gerbillus campestris | 20.550000 | -12.500000 | H6 |
| http://n2t.net/ark:/65665/38ffc3a6b-2cf5-4837-8541-d5dddc38cd3a | Rodentia | Muridae | Gerbillus campestris | 20.550000 | -12.500000 | H6 |
| http://n2t.net/ark:/65665/3f568316a-565f-4e42-9204-584e04661004 | Rodentia | Muridae | Gerbillus campestris | 20.550000 | -12.500000 | H6 |
| http://n2t.net/ark:/65665/337ae8281-062c-4a4f-98c5-86cc48e2a79b | Rodentia | Muridae | Gerbillus campestris | 20.550000 | -12.500000 | H6 |
| http://n2t.net/ark:/65665/320beecd8-1e75-416a-a63f-9c92f5cda255 | Rodentia | Muridae | Gerbillus campestris | 20.550000 | -12.500000 | H6 |
| http://n2t.net/ark:/65665/3dfadc894-a688-4c11-bb48-b903233f73ed | Rodentia | Muridae | Gerbillus campestris | 20.550000 | -12.500000 | H6 |
| http://n2t.net/ark:/65665/34f628739-4be5-458d-b87e-7b8892653300 | Rodentia | Muridae | Gerbillus campestris | 20.550000 | -12.500000 | H6 |
| http://n2t.net/ark:/65665/37a474c48-c062-4468-a5ac-45ca69536b8e | Rodentia | Muridae | Gerbillus campestris | 20.550000 | -12.500000 | H6 |
| http://n2t.net/ark:/65665/3b0881690-d836-4334-b0d9-0da254e07490 | Rodentia | Muridae | Gerbillus campestris | 20.550000 | -12.500000 | H6 |
| http://n2t.net/ark:/65665/3b96041ba-53b5-4804-b700-d5981b65647a | Rodentia | Muridae | Gerbillus campestris | 20.550000 | -12.500000 | H6 |
| http://n2t.net/ark:/65665/3c0c21cde-5836-4ca5-9ef8-19bb95c47f94 | Rodentia | Muridae | Gerbillus campestris | 20.550000 | -12.500000 | H6 |
| http://n2t.net/ark:/65665/3a4c05ae6-4f98-4096-a0a5-89d0f0fe88a4 | Rodentia | Muridae | Gerbillus campestris | 20.550000 | -12.500000 | H6 |
| http://n2t.net/ark:/65665/35abc5ca5-59b5-43c6-b6e3-ef4ca7e1ae5f | Rodentia | Muridae | Gerbillus campestris | 20.550000 | -12.500000 | H6 |
| http://n2t.net/ark:/65665/3c6cb6178-3256-45f1-b817-dc50e8724afc | Rodentia | Muridae | Gerbillus campestris | 20.550000 | -12.500000 | H6 |
| http://coldb.mnhn.fr/catalognumber/mnhn/zm/mo-1997-1407 | Rodentia | Muridae | Gerbillus gerbillus | 18.450000 | -9.500000 | J9 |
| http://coldb.mnhn.fr/catalognumber/mnhn/zm/mo-1997-1406 | Rodentia | Muridae | Gerbillus gerbillus | 18.450000 | -9.500000 | J9 |
| http://coldb.mnhn.fr/catalognumber/mnhn/zm/mo-1997-1416 | Rodentia | Muridae | Gerbillus gerbillus | 17.633000 | -16.028600 | K3 |
| http://coldb.mnhn.fr/catalognumber/mnhn/zm/mo-1997-1420 | Rodentia | Muridae | Gerbillus gerbillus | 17.517000 | -16.033300 | K3 |
| http://coldb.mnhn.fr/catalognumber/mnhn/zm/mo-1997-1423 | Rodentia | Muridae | Gerbillus gerbillus | 20.165000 | -16.221000 | I2 |
| http://coldb.mnhn.fr/catalognumber/mnhn/zm/mo-2001-724 | Rodentia | Muridae | Gerbillus gerbillus | 18.267000 | -16.016900 | K3 |
| http://coldb.mnhn.fr/catalognumber/mnhn/zm/mo-2001-714 | Rodentia | Muridae | Gerbillus gerbillus | 18.267000 | -16.016900 | K3 |
| http://coldb.mnhn.fr/catalognumber/mnhn/zm/mo-2001-1680 | Rodentia | Muridae | Gerbillus gerbillus | 18.267000 | -16.016900 | K3 |
| http://coldb.mnhn.fr/catalognumber/mnhn/zm/mo-1997-1421 | Rodentia | Muridae | Gerbillus gerbillus | 18.267000 | -16.016900 | K3 |
| http://coldb.mnhn.fr/catalognumber/mnhn/zm/mo-1992-1383 | Rodentia | Muridae | Gerbillus gerbillus | 18.115564 | -16.015721 | K3 |
| http://coldb.mnhn.fr/catalognumber/mnhn/zm/mo-1992-1382 | Rodentia | Muridae | Gerbillus gerbillus | 18.115564 | -16.015721 | K3 |
| http://coldb.mnhn.fr/catalognumber/mnhn/zm/mo-1992-1381 | Rodentia | Muridae | Gerbillus gerbillus | 18.115564 | -16.015721 | K3 |
| http://coldb.mnhn.fr/catalognumber/mnhn/zm/mo-1992-1380 | Rodentia | Muridae | Gerbillus gerbillus | 18.115564 | -16.015721 | K3 |
| http://coldb.mnhn.fr/catalognumber/mnhn/zm/mo-1992-1379 | Rodentia | Muridae | Gerbillus gerbillus | 18.115564 | -16.015721 | K3 |
| http://coldb.mnhn.fr/catalognumber/mnhn/zm/mo-1992-1378 | Rodentia | Muridae | Gerbillus gerbillus | 18.115564 | -16.015721 | K3 |
| http://coldb.mnhn.fr/catalognumber/mnhn/zm/mo-1992-1377 | Rodentia | Muridae | Gerbillus gerbillus | 18.115564 | -16.015721 | K3 |
| http://coldb.mnhn.fr/catalognumber/mnhn/zm/mo-1992-1376 | Rodentia | Muridae | Gerbillus gerbillus | 18.115564 | -16.015721 | K3 |
| http://coldb.mnhn.fr/catalognumber/mnhn/zm/mo-1992-1375 | Rodentia | Muridae | Gerbillus gerbillus | 18.115564 | -16.015721 | K3 |
| http://coldb.mnhn.fr/catalognumber/mnhn/zm/mo-1992-1374 | Rodentia | Muridae | Gerbillus gerbillus | 18.115564 | -16.015721 | K3 |
| http://coldb.mnhn.fr/catalognumber/mnhn/zm/mo-1992-1373 | Rodentia | Muridae | Gerbillus gerbillus | 18.115564 | -16.015721 | K3 |
| http://coldb.mnhn.fr/catalognumber/mnhn/zm/mo-1992-1372 | Rodentia | Muridae | Gerbillus gerbillus | 18.115564 | -16.015721 | K3 |
| http://coldb.mnhn.fr/catalognumber/mnhn/zm/mo-1992-1371 | Rodentia | Muridae | Gerbillus gerbillus | 18.115564 | -16.015721 | K3 |
| http://coldb.mnhn.fr/catalognumber/mnhn/zm/mo-1992-1370 | Rodentia | Muridae | Gerbillus gerbillus | 18.115564 | -16.015721 | K3 |
| http://coldb.mnhn.fr/catalognumber/mnhn/zm/mo-1992-1369 | Rodentia | Muridae | Gerbillus gerbillus | 18.115564 | -16.015721 | K3 |
| http://coldb.mnhn.fr/catalognumber/mnhn/zm/mo-1992-1368 | Rodentia | Muridae | Gerbillus gerbillus | 18.115564 | -16.015721 | K3 |
| http://coldb.mnhn.fr/catalognumber/mnhn/zm/mo-1992-1367 | Rodentia | Muridae | Gerbillus gerbillus | 18.115564 | -16.015721 | K3 |
| http://coldb.mnhn.fr/catalognumber/mnhn/zm/mo-1992-1366 | Rodentia | Muridae | Gerbillus gerbillus | 18.115564 | -16.015721 | K3 |
| http://coldb.mnhn.fr/catalognumber/mnhn/zm/mo-1992-1365 | Rodentia | Muridae | Gerbillus gerbillus | 18.115564 | -16.015721 | K3 |
| http://coldb.mnhn.fr/catalognumber/mnhn/zm/mo-1992-1364 | Rodentia | Muridae | Gerbillus gerbillus | 18.115564 | -16.015721 | K3 |
| http://coldb.mnhn.fr/catalognumber/mnhn/zm/mo-1992-1363 | Rodentia | Muridae | Gerbillus gerbillus | 18.115564 | -16.015721 | K3 |
| http://coldb.mnhn.fr/catalognumber/mnhn/zm/mo-1992-1362 | Rodentia | Muridae | Gerbillus gerbillus | 18.115564 | -16.015721 | K3 |
| http://coldb.mnhn.fr/catalognumber/mnhn/zm/mo-1992-1361 | Rodentia | Muridae | Gerbillus gerbillus | 18.115564 | -16.015721 | K3 |
| http://coldb.mnhn.fr/catalognumber/mnhn/zm/mo-1992-1360 | Rodentia | Muridae | Gerbillus gerbillus | 18.115564 | -16.015721 | K3 |
| http://coldb.mnhn.fr/catalognumber/mnhn/zm/mo-1992-1359 | Rodentia | Muridae | Gerbillus gerbillus | 18.115564 | -16.015721 | K3 |
| http://coldb.mnhn.fr/catalognumber/mnhn/zm/mo-1992-1358 | Rodentia | Muridae | Gerbillus gerbillus | 18.115564 | -16.015721 | K3 |
| http://coldb.mnhn.fr/catalognumber/mnhn/zm/mo-1992-1357 | Rodentia | Muridae | Gerbillus gerbillus | 18.115564 | -16.015721 | K3 |
| http://coldb.mnhn.fr/catalognumber/mnhn/zm/mo-1992-1356 | Rodentia | Muridae | Gerbillus gerbillus | 18.115564 | -16.015721 | K3 |
| http://coldb.mnhn.fr/catalognumber/mnhn/zm/mo-1992-1355 | Rodentia | Muridae | Gerbillus gerbillus | 18.115564 | -16.015721 | K3 |
| http://coldb.mnhn.fr/catalognumber/mnhn/zm/mo-1992-1354 | Rodentia | Muridae | Gerbillus gerbillus | 18.115564 | -16.015721 | K3 |
| http://coldb.mnhn.fr/catalognumber/mnhn/zm/mo-1992-1353 | Rodentia | Muridae | Gerbillus gerbillus | 18.115564 | -16.015721 | K3 |
| http://coldb.mnhn.fr/catalognumber/mnhn/zm/mo-1992-1352 | Rodentia | Muridae | Gerbillus gerbillus | 18.115564 | -16.015721 | K3 |
| http://coldb.mnhn.fr/catalognumber/mnhn/zm/mo-1992-1351 | Rodentia | Muridae | Gerbillus gerbillus | 18.115564 | -16.015721 | K3 |
| http://coldb.mnhn.fr/catalognumber/mnhn/zm/mo-1992-1350 | Rodentia | Muridae | Gerbillus gerbillus | 18.115564 | -16.015721 | K3 |
| http://coldb.mnhn.fr/catalognumber/mnhn/zm/mo-1992-1349 | Rodentia | Muridae | Gerbillus gerbillus | 18.115564 | -16.015721 | K3 |
| http://coldb.mnhn.fr/catalognumber/mnhn/zm/mo-1992-1348 | Rodentia | Muridae | Gerbillus gerbillus | 18.115564 | -16.015721 | K3 |
| http://coldb.mnhn.fr/catalognumber/mnhn/zm/mo-1992-1347 | Rodentia | Muridae | Gerbillus gerbillus | 18.115564 | -16.015721 | K3 |
| http://coldb.mnhn.fr/catalognumber/mnhn/zm/mo-1992-1346 | Rodentia | Muridae | Gerbillus gerbillus | 18.115564 | -16.015721 | K3 |
| http://coldb.mnhn.fr/catalognumber/mnhn/zm/mo-1992-1345 | Rodentia | Muridae | Gerbillus gerbillus | 18.115564 | -16.015721 | K3 |
| http://coldb.mnhn.fr/catalognumber/mnhn/zm/mo-1992-1344 | Rodentia | Muridae | Gerbillus gerbillus | 18.115564 | -16.015721 | K3 |
| http://coldb.mnhn.fr/catalognumber/mnhn/zm/mo-1992-1343 | Rodentia | Muridae | Gerbillus gerbillus | 18.115564 | -16.015721 | K3 |
| http://coldb.mnhn.fr/catalognumber/mnhn/zm/mo-1992-1342 | Rodentia | Muridae | Gerbillus gerbillus | 18.115564 | -16.015721 | K3 |
| http://coldb.mnhn.fr/catalognumber/mnhn/zm/mo-1992-1341 | Rodentia | Muridae | Gerbillus gerbillus | 18.115564 | -16.015721 | K3 |
| http://coldb.mnhn.fr/catalognumber/mnhn/zm/mo-1992-1340 | Rodentia | Muridae | Gerbillus gerbillus | 18.115564 | -16.015721 | K3 |
| http://coldb.mnhn.fr/catalognumber/mnhn/zm/mo-1992-1339 | Rodentia | Muridae | Gerbillus gerbillus | 18.115564 | -16.015721 | K3 |
| http://coldb.mnhn.fr/catalognumber/mnhn/zm/mo-1992-1338 | Rodentia | Muridae | Gerbillus gerbillus | 18.115564 | -16.015721 | K3 |
| http://coldb.mnhn.fr/catalognumber/mnhn/zm/mo-1992-1337 | Rodentia | Muridae | Gerbillus gerbillus | 18.115564 | -16.015721 | K3 |
| http://coldb.mnhn.fr/catalognumber/mnhn/zm/mo-1992-1336 | Rodentia | Muridae | Gerbillus gerbillus | 18.115564 | -16.015721 | K3 |
| http://coldb.mnhn.fr/catalognumber/mnhn/zm/mo-1992-1335 | Rodentia | Muridae | Gerbillus gerbillus | 18.115564 | -16.015721 | K3 |
| http://coldb.mnhn.fr/catalognumber/mnhn/zm/mo-1911-1569 | Rodentia | Muridae | Gerbillus gerbillus | 18.115564 | -16.015721 | K3 |
| http://coldb.mnhn.fr/catalognumber/mnhn/zm/mo-1995-593 | Rodentia | Muridae | Gerbillus gerbillus | 19.831000 | -15.923000 | I3 |
| http://coldb.mnhn.fr/catalognumber/mnhn/zm/mo-1995-569 | Rodentia | Muridae | Gerbillus gerbillus | 19.831000 | -15.923000 | I3 |
| http://coldb.mnhn.fr/catalognumber/mnhn/zm/mo-1995-566 | Rodentia | Muridae | Gerbillus gerbillus | 19.831000 | -15.923000 | I3 |
| http://coldb.mnhn.fr/catalognumber/mnhn/zm/mo-1990-56 | Rodentia | Muridae | Gerbillus gerbillus | 19.831000 | -15.923000 | I3 |
| http://coldb.mnhn.fr/catalognumber/mnhn/zm/mo-1982-539 | Rodentia | Muridae | Gerbillus gerbillus | 19.831000 | -15.923000 | I3 |
| http://coldb.mnhn.fr/catalognumber/mnhn/zm/mo-1981-622 | Rodentia | Muridae | Gerbillus gerbillus | 19.831000 | -15.923000 | I3 |
| http://coldb.mnhn.fr/catalognumber/mnhn/zm/mo-1980-402 | Rodentia | Muridae | Gerbillus gerbillus | 19.831000 | -15.923000 | I3 |
| http://coldb.mnhn.fr/catalognumber/mnhn/zm/mo-1980-401 | Rodentia | Muridae | Gerbillus gerbillus | 19.831000 | -15.923000 | I3 |
| http://coldb.mnhn.fr/catalognumber/mnhn/zm/mo-1977-99 | Rodentia | Muridae | Gerbillus gerbillus | 19.831000 | -15.923000 | I3 |
| http://coldb.mnhn.fr/catalognumber/mnhn/zm/mo-1977-98 | Rodentia | Muridae | Gerbillus gerbillus | 19.831000 | -15.923000 | I3 |
| http://coldb.mnhn.fr/catalognumber/mnhn/zm/mo-1977-97 | Rodentia | Muridae | Gerbillus gerbillus | 19.831000 | -15.923000 | I3 |
| http://coldb.mnhn.fr/catalognumber/mnhn/zm/mo-1977-96 | Rodentia | Muridae | Gerbillus gerbillus | 19.831000 | -15.923000 | I3 |
| http://coldb.mnhn.fr/catalognumber/mnhn/zm/mo-1977-95 | Rodentia | Muridae | Gerbillus gerbillus | 19.831000 | -15.923000 | I3 |
| http://coldb.mnhn.fr/catalognumber/mnhn/zm/mo-1977-94 | Rodentia | Muridae | Gerbillus gerbillus | 19.831000 | -15.923000 | I3 |
| http://coldb.mnhn.fr/catalognumber/mnhn/zm/mo-1977-93 | Rodentia | Muridae | Gerbillus gerbillus | 19.831000 | -15.923000 | I3 |
| http://coldb.mnhn.fr/catalognumber/mnhn/zm/mo-1977-92 | Rodentia | Muridae | Gerbillus gerbillus | 19.831000 | -15.923000 | I3 |
| http://coldb.mnhn.fr/catalognumber/mnhn/zm/mo-1977-201 | Rodentia | Muridae | Gerbillus gerbillus | 19.831000 | -15.923000 | I3 |
| http://coldb.mnhn.fr/catalognumber/mnhn/zm/mo-1977-200 | Rodentia | Muridae | Gerbillus gerbillus | 19.831000 | -15.923000 | I3 |
| http://coldb.mnhn.fr/catalognumber/mnhn/zm/mo-1977-199 | Rodentia | Muridae | Gerbillus gerbillus | 19.831000 | -15.923000 | I3 |
| http://coldb.mnhn.fr/catalognumber/mnhn/zm/mo-1977-198 | Rodentia | Muridae | Gerbillus gerbillus | 19.831000 | -15.923000 | I3 |
| http://coldb.mnhn.fr/catalognumber/mnhn/zm/mo-1977-197 | Rodentia | Muridae | Gerbillus gerbillus | 19.831000 | -15.923000 | I3 |
| http://coldb.mnhn.fr/catalognumber/mnhn/zm/mo-1977-196 | Rodentia | Muridae | Gerbillus gerbillus | 19.831000 | -15.923000 | I3 |
| http://coldb.mnhn.fr/catalognumber/mnhn/zm/mo-1977-195 | Rodentia | Muridae | Gerbillus gerbillus | 19.831000 | -15.923000 | I3 |
| http://coldb.mnhn.fr/catalognumber/mnhn/zm/mo-1977-194 | Rodentia | Muridae | Gerbillus gerbillus | 19.831000 | -15.923000 | I3 |
| http://coldb.mnhn.fr/catalognumber/mnhn/zm/mo-1977-193 | Rodentia | Muridae | Gerbillus gerbillus | 19.831000 | -15.923000 | I3 |
| http://coldb.mnhn.fr/catalognumber/mnhn/zm/mo-1977-192 | Rodentia | Muridae | Gerbillus gerbillus | 19.831000 | -15.923000 | I3 |
| http://coldb.mnhn.fr/catalognumber/mnhn/zm/mo-1977-191 | Rodentia | Muridae | Gerbillus gerbillus | 19.831000 | -15.923000 | I3 |
| http://coldb.mnhn.fr/catalognumber/mnhn/zm/mo-1977-190 | Rodentia | Muridae | Gerbillus gerbillus | 19.831000 | -15.923000 | I3 |
| http://coldb.mnhn.fr/catalognumber/mnhn/zm/mo-1977-189 | Rodentia | Muridae | Gerbillus gerbillus | 19.831000 | -15.923000 | I3 |
| http://coldb.mnhn.fr/catalognumber/mnhn/zm/mo-1977-188 | Rodentia | Muridae | Gerbillus gerbillus | 19.831000 | -15.923000 | I3 |
| http://coldb.mnhn.fr/catalognumber/mnhn/zm/mo-1977-178 | Rodentia | Muridae | Gerbillus gerbillus | 19.831000 | -15.923000 | I3 |
| http://coldb.mnhn.fr/catalognumber/mnhn/zm/mo-1977-171 | Rodentia | Muridae | Gerbillus gerbillus | 19.831000 | -15.923000 | I3 |
| http://coldb.mnhn.fr/catalognumber/mnhn/zm/mo-1977-157 | Rodentia | Muridae | Gerbillus gerbillus | 19.831000 | -15.923000 | I3 |
| http://coldb.mnhn.fr/catalognumber/mnhn/zm/mo-1977-156 | Rodentia | Muridae | Gerbillus gerbillus | 19.831000 | -15.923000 | I3 |
| http://coldb.mnhn.fr/catalognumber/mnhn/zm/mo-1977-155 | Rodentia | Muridae | Gerbillus gerbillus | 19.831000 | -15.923000 | I3 |
| http://coldb.mnhn.fr/catalognumber/mnhn/zm/mo-1977-154 | Rodentia | Muridae | Gerbillus gerbillus | 19.831000 | -15.923000 | I3 |
| http://coldb.mnhn.fr/catalognumber/mnhn/zm/mo-1977-153 | Rodentia | Muridae | Gerbillus gerbillus | 19.831000 | -15.923000 | I3 |
| http://coldb.mnhn.fr/catalognumber/mnhn/zm/mo-1977-152 | Rodentia | Muridae | Gerbillus gerbillus | 19.831000 | -15.923000 | I3 |
| http://coldb.mnhn.fr/catalognumber/mnhn/zm/mo-1977-151 | Rodentia | Muridae | Gerbillus gerbillus | 19.831000 | -15.923000 | I3 |
| http://coldb.mnhn.fr/catalognumber/mnhn/zm/mo-1977-150 | Rodentia | Muridae | Gerbillus gerbillus | 19.831000 | -15.923000 | I3 |
| http://coldb.mnhn.fr/catalognumber/mnhn/zm/mo-1977-149 | Rodentia | Muridae | Gerbillus gerbillus | 19.831000 | -15.923000 | I3 |
| http://coldb.mnhn.fr/catalognumber/mnhn/zm/mo-1977-148 | Rodentia | Muridae | Gerbillus gerbillus | 19.831000 | -15.923000 | I3 |
| http://coldb.mnhn.fr/catalognumber/mnhn/zm/mo-1977-147 | Rodentia | Muridae | Gerbillus gerbillus | 19.831000 | -15.923000 | I3 |
| http://coldb.mnhn.fr/catalognumber/mnhn/zm/mo-1977-146 | Rodentia | Muridae | Gerbillus gerbillus | 19.831000 | -15.923000 | I3 |
| http://coldb.mnhn.fr/catalognumber/mnhn/zm/mo-1977-145 | Rodentia | Muridae | Gerbillus gerbillus | 19.831000 | -15.923000 | I3 |
| http://coldb.mnhn.fr/catalognumber/mnhn/zm/mo-1977-144 | Rodentia | Muridae | Gerbillus gerbillus | 19.831000 | -15.923000 | I3 |
| http://coldb.mnhn.fr/catalognumber/mnhn/zm/mo-1977-143 | Rodentia | Muridae | Gerbillus gerbillus | 19.831000 | -15.923000 | I3 |
| http://coldb.mnhn.fr/catalognumber/mnhn/zm/mo-1977-142 | Rodentia | Muridae | Gerbillus gerbillus | 19.831000 | -15.923000 | I3 |
| http://coldb.mnhn.fr/catalognumber/mnhn/zm/mo-1977-141 | Rodentia | Muridae | Gerbillus gerbillus | 19.831000 | -15.923000 | I3 |
| http://coldb.mnhn.fr/catalognumber/mnhn/zm/mo-1977-139 | Rodentia | Muridae | Gerbillus gerbillus | 19.831000 | -15.923000 | I3 |
| http://coldb.mnhn.fr/catalognumber/mnhn/zm/mo-1977-138 | Rodentia | Muridae | Gerbillus gerbillus | 19.831000 | -15.923000 | I3 |
| http://coldb.mnhn.fr/catalognumber/mnhn/zm/mo-1977-137 | Rodentia | Muridae | Gerbillus gerbillus | 19.831000 | -15.923000 | I3 |
| http://coldb.mnhn.fr/catalognumber/mnhn/zm/mo-1977-136 | Rodentia | Muridae | Gerbillus gerbillus | 19.831000 | -15.923000 | I3 |
| http://coldb.mnhn.fr/catalognumber/mnhn/zm/mo-1977-134 | Rodentia | Muridae | Gerbillus gerbillus | 19.831000 | -15.923000 | I3 |
| http://coldb.mnhn.fr/catalognumber/mnhn/zm/mo-1977-133 | Rodentia | Muridae | Gerbillus gerbillus | 19.831000 | -15.923000 | I3 |
| http://coldb.mnhn.fr/catalognumber/mnhn/zm/mo-1977-132 | Rodentia | Muridae | Gerbillus gerbillus | 19.831000 | -15.923000 | I3 |
| http://coldb.mnhn.fr/catalognumber/mnhn/zm/mo-1977-131 | Rodentia | Muridae | Gerbillus gerbillus | 19.831000 | -15.923000 | I3 |
| http://coldb.mnhn.fr/catalognumber/mnhn/zm/mo-1977-130 | Rodentia | Muridae | Gerbillus gerbillus | 19.831000 | -15.923000 | I3 |
| http://coldb.mnhn.fr/catalognumber/mnhn/zm/mo-1977-129 | Rodentia | Muridae | Gerbillus gerbillus | 19.831000 | -15.923000 | I3 |
| http://coldb.mnhn.fr/catalognumber/mnhn/zm/mo-1977-128 | Rodentia | Muridae | Gerbillus gerbillus | 19.831000 | -15.923000 | I3 |
| http://coldb.mnhn.fr/catalognumber/mnhn/zm/mo-1977-127 | Rodentia | Muridae | Gerbillus gerbillus | 19.831000 | -15.923000 | I3 |
| http://coldb.mnhn.fr/catalognumber/mnhn/zm/mo-1977-126 | Rodentia | Muridae | Gerbillus gerbillus | 19.831000 | -15.923000 | I3 |
| http://coldb.mnhn.fr/catalognumber/mnhn/zm/mo-1977-125 | Rodentia | Muridae | Gerbillus gerbillus | 19.831000 | -15.923000 | I3 |
| http://coldb.mnhn.fr/catalognumber/mnhn/zm/mo-1977-124 | Rodentia | Muridae | Gerbillus gerbillus | 19.831000 | -15.923000 | I3 |
| http://coldb.mnhn.fr/catalognumber/mnhn/zm/mo-1977-123 | Rodentia | Muridae | Gerbillus gerbillus | 19.831000 | -15.923000 | I3 |
| http://coldb.mnhn.fr/catalognumber/mnhn/zm/mo-1977-121 | Rodentia | Muridae | Gerbillus gerbillus | 19.831000 | -15.923000 | I3 |
| http://coldb.mnhn.fr/catalognumber/mnhn/zm/mo-1977-120 | Rodentia | Muridae | Gerbillus gerbillus | 19.831000 | -15.923000 | I3 |
| http://coldb.mnhn.fr/catalognumber/mnhn/zm/mo-1977-119 | Rodentia | Muridae | Gerbillus gerbillus | 19.831000 | -15.923000 | I3 |
| http://coldb.mnhn.fr/catalognumber/mnhn/zm/mo-1977-118 | Rodentia | Muridae | Gerbillus gerbillus | 19.831000 | -15.923000 | I3 |
| http://coldb.mnhn.fr/catalognumber/mnhn/zm/mo-1977-117 | Rodentia | Muridae | Gerbillus gerbillus | 19.831000 | -15.923000 | I3 |
| http://coldb.mnhn.fr/catalognumber/mnhn/zm/mo-1977-116 | Rodentia | Muridae | Gerbillus gerbillus | 19.831000 | -15.923000 | I3 |
| http://coldb.mnhn.fr/catalognumber/mnhn/zm/mo-1977-115 | Rodentia | Muridae | Gerbillus gerbillus | 19.831000 | -15.923000 | I3 |
| http://coldb.mnhn.fr/catalognumber/mnhn/zm/mo-1977-114 | Rodentia | Muridae | Gerbillus gerbillus | 19.831000 | -15.923000 | I3 |
| http://coldb.mnhn.fr/catalognumber/mnhn/zm/mo-1977-113 | Rodentia | Muridae | Gerbillus gerbillus | 19.831000 | -15.923000 | I3 |
| http://coldb.mnhn.fr/catalognumber/mnhn/zm/mo-1977-111 | Rodentia | Muridae | Gerbillus gerbillus | 19.831000 | -15.923000 | I3 |
| http://coldb.mnhn.fr/catalognumber/mnhn/zm/mo-1977-110 | Rodentia | Muridae | Gerbillus gerbillus | 19.831000 | -15.923000 | I3 |
| http://coldb.mnhn.fr/catalognumber/mnhn/zm/mo-1977-109 | Rodentia | Muridae | Gerbillus gerbillus | 19.831000 | -15.923000 | I3 |
| http://coldb.mnhn.fr/catalognumber/mnhn/zm/mo-1977-108 | Rodentia | Muridae | Gerbillus gerbillus | 19.831000 | -15.923000 | I3 |
| http://coldb.mnhn.fr/catalognumber/mnhn/zm/mo-1977-106 | Rodentia | Muridae | Gerbillus gerbillus | 19.831000 | -15.923000 | I3 |
| http://coldb.mnhn.fr/catalognumber/mnhn/zm/mo-1977-105 | Rodentia | Muridae | Gerbillus gerbillus | 19.831000 | -15.923000 | I3 |
| http://coldb.mnhn.fr/catalognumber/mnhn/zm/mo-1977-104 | Rodentia | Muridae | Gerbillus gerbillus | 19.831000 | -15.923000 | I3 |
| http://coldb.mnhn.fr/catalognumber/mnhn/zm/mo-1977-103 | Rodentia | Muridae | Gerbillus gerbillus | 19.831000 | -15.923000 | I3 |
| http://coldb.mnhn.fr/catalognumber/mnhn/zm/mo-1977-102 | Rodentia | Muridae | Gerbillus gerbillus | 19.831000 | -15.923000 | I3 |
| http://coldb.mnhn.fr/catalognumber/mnhn/zm/mo-1977-101 | Rodentia | Muridae | Gerbillus gerbillus | 19.831000 | -15.923000 | I3 |
| http://coldb.mnhn.fr/catalognumber/mnhn/zm/mo-1977-100 | Rodentia | Muridae | Gerbillus gerbillus | 19.831000 | -15.923000 | I3 |
| http://coldb.mnhn.fr/catalognumber/mnhn/zm/mo-1975-515 | Rodentia | Muridae | Gerbillus gerbillus | 19.831000 | -15.923000 | I3 |
| http://coldb.mnhn.fr/catalognumber/mnhn/zm/mo-1975-509 | Rodentia | Muridae | Gerbillus gerbillus | 19.831000 | -15.923000 | I3 |
| http://coldb.mnhn.fr/catalognumber/mnhn/zm/mo-1975-507 | Rodentia | Muridae | Gerbillus gerbillus | 19.831000 | -15.923000 | I3 |
| http://coldb.mnhn.fr/catalognumber/mnhn/zm/mo-1975-506 | Rodentia | Muridae | Gerbillus gerbillus | 19.831000 | -15.923000 | I3 |
| http://coldb.mnhn.fr/catalognumber/mnhn/zm/mo-1975-505 | Rodentia | Muridae | Gerbillus gerbillus | 19.831000 | -15.923000 | I3 |
| http://coldb.mnhn.fr/catalognumber/mnhn/zm/mo-1975-503 | Rodentia | Muridae | Gerbillus gerbillus | 19.831000 | -15.923000 | I3 |
| http://coldb.mnhn.fr/catalognumber/mnhn/zm/mo-1975-502 | Rodentia | Muridae | Gerbillus gerbillus | 19.831000 | -15.923000 | I3 |
| http://coldb.mnhn.fr/catalognumber/mnhn/zm/mo-1975-501 | Rodentia | Muridae | Gerbillus gerbillus | 19.831000 | -15.923000 | I3 |
| http://coldb.mnhn.fr/catalognumber/mnhn/zm/mo-1995-591 | Rodentia | Muridae | Gerbillus gerbillus | 19.831000 | -15.923000 | I3 |
| http://coldb.mnhn.fr/catalognumber/mnhn/zm/mo-1995-589 | Rodentia | Muridae | Gerbillus gerbillus | 19.831000 | -15.923000 | I3 |
| http://coldb.mnhn.fr/catalognumber/mnhn/zm/mo-1995-588 | Rodentia | Muridae | Gerbillus gerbillus | 19.831000 | -15.923000 | I3 |
| http://coldb.mnhn.fr/catalognumber/mnhn/zm/mo-1995-586 | Rodentia | Muridae | Gerbillus gerbillus | 19.831000 | -15.923000 | I3 |
| http://coldb.mnhn.fr/catalognumber/mnhn/zm/mo-1995-585 | Rodentia | Muridae | Gerbillus gerbillus | 19.831000 | -15.923000 | I3 |
| http://coldb.mnhn.fr/catalognumber/mnhn/zm/mo-1995-584 | Rodentia | Muridae | Gerbillus gerbillus | 19.831000 | -15.923000 | I3 |
| http://coldb.mnhn.fr/catalognumber/mnhn/zm/mo-1995-583 | Rodentia | Muridae | Gerbillus gerbillus | 19.831000 | -15.923000 | I3 |
| http://coldb.mnhn.fr/catalognumber/mnhn/zm/mo-1995-582 | Rodentia | Muridae | Gerbillus gerbillus | 19.831000 | -15.923000 | I3 |
| http://coldb.mnhn.fr/catalognumber/mnhn/zm/mo-1995-581 | Rodentia | Muridae | Gerbillus gerbillus | 19.831000 | -15.923000 | I3 |
| http://coldb.mnhn.fr/catalognumber/mnhn/zm/mo-1995-580 | Rodentia | Muridae | Gerbillus gerbillus | 19.831000 | -15.923000 | I3 |
| http://coldb.mnhn.fr/catalognumber/mnhn/zm/mo-1995-579 | Rodentia | Muridae | Gerbillus gerbillus | 19.831000 | -15.923000 | I3 |
| http://coldb.mnhn.fr/catalognumber/mnhn/zm/mo-1995-578 | Rodentia | Muridae | Gerbillus gerbillus | 19.831000 | -15.923000 | I3 |
| http://coldb.mnhn.fr/catalognumber/mnhn/zm/mo-1995-577 | Rodentia | Muridae | Gerbillus gerbillus | 19.831000 | -15.923000 | I3 |
| http://coldb.mnhn.fr/catalognumber/mnhn/zm/mo-1995-576 | Rodentia | Muridae | Gerbillus gerbillus | 19.831000 | -15.923000 | I3 |
| http://coldb.mnhn.fr/catalognumber/mnhn/zm/mo-1978-54 | Rodentia | Muridae | Gerbillus gerbillus | 19.831000 | -15.923000 | I3 |
| http://coldb.mnhn.fr/catalognumber/mnhn/zm/mo-1978-48 | Rodentia | Muridae | Gerbillus gerbillus | 19.831000 | -15.923000 | I3 |
| http://coldb.mnhn.fr/catalognumber/mnhn/zm/mo-1978-47 | Rodentia | Muridae | Gerbillus gerbillus | 19.831000 | -15.923000 | I3 |
| http://coldb.mnhn.fr/catalognumber/mnhn/zm/mo-1978-46 | Rodentia | Muridae | Gerbillus gerbillus | 19.831000 | -15.923000 | I3 |
| http://coldb.mnhn.fr/catalognumber/mnhn/zm/mo-1978-44 | Rodentia | Muridae | Gerbillus gerbillus | 19.831000 | -15.923000 | I3 |
| http://coldb.mnhn.fr/catalognumber/mnhn/zm/mo-1978-43 | Rodentia | Muridae | Gerbillus gerbillus | 19.831000 | -15.923000 | I3 |
| http://coldb.mnhn.fr/catalognumber/mnhn/zm/mo-1978-42 | Rodentia | Muridae | Gerbillus gerbillus | 19.831000 | -15.923000 | I3 |
| http://coldb.mnhn.fr/catalognumber/mnhn/zm/mo-1978-41 | Rodentia | Muridae | Gerbillus gerbillus | 19.831000 | -15.923000 | I3 |
| http://coldb.mnhn.fr/catalognumber/mnhn/zm/mo-1997-1426 | Rodentia | Muridae | Gerbillus gerbillus | 17.467000 | -13.150000 | K6 |
| http://coldb.mnhn.fr/catalognumber/mnhn/zm/mo-1997-1425 | Rodentia | Muridae | Gerbillus gerbillus | 17.467000 | -13.150000 | K6 |
| http://coldb.mnhn.fr/catalognumber/mnhn/zm/2004-141 | Rodentia | Muridae | Gerbillus gerbillus | 19.083330 | -16.233300 | J2 |
| http://coldb.mnhn.fr/catalognumber/mnhn/zm/mo-1997-1424 | Rodentia | Muridae | Gerbillus gerbillus | 19.083330 | -16.233300 | J2 |
| http://coldb.mnhn.fr/catalognumber/mnhn/zm/mo-1997-1418 | Rodentia | Muridae | Gerbillus gerbillus | 19.083330 | -16.233300 | J2 |
| http://coldb.mnhn.fr/catalognumber/mnhn/zm/mo-1997-1415 | Rodentia | Muridae | Gerbillus gerbillus | 19.033000 | -16.231000 | J2 |
| http://coldb.mnhn.fr/catalognumber/mnhn/zm/mo-1997-1405 | Rodentia | Muridae | Gerbillus gerbillus | 18.367000 | -10.983000 | J8 |
| http://coldb.mnhn.fr/catalognumber/mnhn/zm/mo-1997-1410 | Rodentia | Muridae | Gerbillus gerbillus | 18.650000 | -11.583000 | J7 |
| http://coldb.mnhn.fr/catalognumber/mnhn/zm/mo-1997-1409 | Rodentia | Muridae | Gerbillus gerbillus | 18.650000 | -11.583000 | J7 |
| http://coldb.mnhn.fr/catalognumber/mnhn/zm/mo-1997-1408 | Rodentia | Muridae | Gerbillus gerbillus | 18.650000 | -11.583000 | J7 |
| http://coldb.mnhn.fr/catalognumber/mnhn/zm/mo-2001-92 | Rodentia | Muridae | Gerbillus gerbillus | 16.375000 | -16.466000 | M2 |
| http://coldb.mnhn.fr/catalognumber/mnhn/zm/mo-2001-741 | Rodentia | Muridae | Gerbillus gerbillus | 16.372000 | -16.462000 | M2 |
| http://coldb.mnhn.fr/catalognumber/mnhn/zm/mo-2001-721 | Rodentia | Muridae | Gerbillus gerbillus | 16.607000 | -16.439323 | M2 |
| http://coldb.mnhn.fr/catalognumber/mnhn/zm/mo-2001-720 | Rodentia | Muridae | Gerbillus gerbillus | 16.607000 | -16.439323 | M2 |
| http://coldb.mnhn.fr/catalognumber/mnhn/zm/mo-2001-1681 | Rodentia | Muridae | Gerbillus gerbillus | 16.607000 | -16.439323 | M2 |
| http://coldb.mnhn.fr/catalognumber/mnhn/zm/mo-1997-1417 | Rodentia | Muridae | Gerbillus gerbillus | 16.607000 | -16.439323 | M2 |
| http://coldb.mnhn.fr/catalognumber/mnhn/zm/mo-1978-61 | Rodentia | Muridae | Gerbillus gerbillus | 20.099201 | -15.926960 | I3 |
| http://coldb.mnhn.fr/catalognumber/mnhn/zm/mo-1978-60 | Rodentia | Muridae | Gerbillus gerbillus | 20.099201 | -15.926960 | I3 |
| http://coldb.mnhn.fr/catalognumber/mnhn/zm/mo-1978-59 | Rodentia | Muridae | Gerbillus gerbillus | 20.099201 | -15.926960 | I3 |
| http://coldb.mnhn.fr/catalognumber/mnhn/zm/mo-1978-58 | Rodentia | Muridae | Gerbillus gerbillus | 20.099201 | -15.926960 | I3 |
| http://coldb.mnhn.fr/catalognumber/mnhn/zm/mo-1978-57 | Rodentia | Muridae | Gerbillus gerbillus | 20.099201 | -15.926960 | I3 |
| http://coldb.mnhn.fr/catalognumber/mnhn/zm/mo-1978-56 | Rodentia | Muridae | Gerbillus gerbillus | 20.099201 | -15.926960 | I3 |
| http://coldb.mnhn.fr/catalognumber/mnhn/zm/mo-1978-55 | Rodentia | Muridae | Gerbillus gerbillus | 20.099201 | -15.926960 | I3 |
| http://coldb.mnhn.fr/catalognumber/mnhn/zm/mo-1978-53 | Rodentia | Muridae | Gerbillus gerbillus | 20.099201 | -15.926960 | I3 |
| http://coldb.mnhn.fr/catalognumber/mnhn/zm/mo-1978-51 | Rodentia | Muridae | Gerbillus gerbillus | 20.099201 | -15.926960 | I3 |
| http://coldb.mnhn.fr/catalognumber/mnhn/zm/mo-1978-49 | Rodentia | Muridae | Gerbillus gerbillus | 20.099201 | -15.926960 | I3 |
| http://coldb.mnhn.fr/catalognumber/mnhn/zm/mo-1978-62 | Rodentia | Muridae | Gerbillus gerbillus | 20.099201 | -15.926960 | I3 |
| http://coldb.mnhn.fr/catalognumber/mnhn/zm/mo-1978-63 | Rodentia | Muridae | Gerbillus gerbillus | 20.099201 | -15.926960 | I3 |
| http://coldb.mnhn.fr/catalognumber/mnhn/zm/mo-1978-50 | Rodentia | Muridae | Gerbillus gerbillus | 20.099201 | -15.926960 | I3 |
| http://coldb.mnhn.fr/catalognumber/mnhn/zm/mo-1997-1419 | Rodentia | Muridae | Gerbillus gerbillus | 20.687000 | -16.674000 | H2 |
| http://coldb.mnhn.fr/catalognumber/mnhn/zm/mo-1997-1427 | Rodentia | Muridae | Gerbillus gerbillus | 18.767000 | -15.317000 | J3 |
| http://coldb.mnhn.fr/catalognumber/mnhn/zm/mo-1997-1411 | Rodentia | Muridae | Gerbillus gerbillus | 18.767000 | -15.317000 | J3 |
| http://coldb.mnhn.fr/catalognumber/mnhn/zm/mo-1997-1412 | Rodentia | Muridae | Gerbillus gerbillus | 18.467000 | -15.550000 | J3 |
| http://coldb.mnhn.fr/catalognumber/mnhn/zm/mo-1997-1414 | Rodentia | Muridae | Gerbillus gerbillus | 19.967000 | -13.350000 | I5 |
| http://coldb.mnhn.fr/catalognumber/mnhn/zm/mo-1997-1413 | Rodentia | Muridae | Gerbillus gerbillus | 19.967000 | -13.350000 | I5 |
| http://n2t.net/ark:/65665/33f17eb3c-7a75-464c-9d76-7e5b01fd451c | Rodentia | Muridae | Gerbillus gerbillus | 19.750000 | -14.380000 | I4 |
| http://n2t.net/ark:/65665/3f93453be-4f29-454f-ba07-4fbdd80d2fa2 | Rodentia | Muridae | Gerbillus gerbillus | 19.750000 | -14.380000 | I4 |
| http://n2t.net/ark:/65665/36dca52a3-d750-4f2e-bd93-492f239b6d45 | Rodentia | Muridae | Gerbillus gerbillus | 19.750000 | -14.380000 | I4 |
| http://n2t.net/ark:/65665/3129562e8-66ab-4ce7-8a92-747b79087d8d | Rodentia | Muridae | Gerbillus gerbillus | 19.750000 | -14.380000 | I4 |
| http://n2t.net/ark:/65665/327b69023-5258-48bd-a909-19812e707cce | Rodentia | Muridae | Gerbillus gerbillus | 19.750000 | -14.380000 | I4 |
| d2ab89e8-02a3-4ff2-9bee-dab37eed4109 | Rodentia | Muridae | Gerbillus gerbillus | 20.172297 | -13.658762 | H5 |
| 32082b24-91bc-413a-b084-d977eb18bfcc | Rodentia | Muridae | Gerbillus gerbillus | 19.071674 | -15.050074 | J4 |
| 160998b6-f918-4018-bde6-dd4c2489fc77 | Rodentia | Muridae | Gerbillus gerbillus | 19.114910 | -14.944218 | J4 |
| fe807086-4cc6-4033-a3ca-033f2d1ca85b | Rodentia | Muridae | Gerbillus gerbillus | 20.730418 | -16.024828 | H3 |
| 1db9c143-b901-48d5-a971-0f5001971e29 | Rodentia | Muridae | Gerbillus gerbillus | 18.206234 | -11.730334 | K7 |
| 2633e398-108a-4b72-b0f0-413bf91d5a6f | Rodentia | Muridae | Gerbillus gerbillus | 18.206040 | -11.730977 | K7 |
| 9e8fc806-50e5-4430-8818-a34fc3064955 | Rodentia | Muridae | Gerbillus gerbillus | 18.442821 | -11.387395 | J7 |
| 42aa4dbb-5674-42a1-995e-422086b81157 | Rodentia | Muridae | Gerbillus gerbillus | 18.447193 | -10.683335 | J8 |
| 9d593e73-0ba5-4cf5-b1a9-07512d859314 | Rodentia | Muridae | Gerbillus gerbillus | 18.578719 | -9.818611 | J9 |
| d19f8249-267a-4747-9d9f-7d1e38b21fbb | Rodentia | Muridae | Gerbillus gerbillus | 18.367468 | -9.048530 | J10 |
| 3751b6a9-78f7-4858-9581-1bffc6093f91 | Rodentia | Muridae | Gerbillus gerbillus | 18.383716 | -8.521678 | J10 |
| bc98387d-2096-4cd7-ba16-cc723d037db8 | Rodentia | Muridae | Gerbillus gerbillus | 18.099577 | -8.010730 | K11 |
| 62ec4d27-ad8b-4648-88cd-22ae82f9a123 | Rodentia | Muridae | Gerbillus gerbillus | 17.589160 | -7.445969 | K12 |
| 3f0353dd-1d8f-439a-9311-4ac3702ac1e4 | Rodentia | Muridae | Gerbillus gerbillus | 16.484142 | -9.289591 | M10 |
| 13452e08-12e5-476d-9c3c-5ded4513270c | Rodentia | Muridae | Gerbillus gerbillus | 19.740060 | -16.274618 | I2 |
| 215c6a39-39aa-44d7-8caa-b09df290946c | Rodentia | Muridae | Gerbillus gerbillus | 21.372827 | -11.910393 | G7 |
| 99a00edf-d66e-4b71-a443-5b2c7d8cc221 | Rodentia | Muridae | Gerbillus gerbillus | 21.519632 | -12.853367 | G6 |
| 4aef2703-26e8-4014-bf09-e2c67208a87c | Rodentia | Muridae | Gerbillus gerbillus | 21.197600 | -14.222085 | G4 |
| f9358d9a-4a88-4773-86e7-51f9edfb560a | Rodentia | Muridae | Gerbillus gerbillus | 21.280500 | -16.091700 | G2 |
| e73f85f3-a46e-4ac9-a9ad-5bb974a86e8e | Rodentia | Muridae | Gerbillus gerbillus | 18.557700 | -11.460600 | J7 |
| http://n2t.net/ark:/65665/397fabf7e-94c9-4878-892b-fb5b2fc83591 | Rodentia | Muridae | Gerbillus nancillus | 15.933333 | -12.016667 | M7 |
| http://n2t.net/ark:/65665/3880f91e9-3f3e-4901-9dda-6e0f9729365a | Rodentia | Muridae | Gerbillus nancillus | 18.068000 | -15.901000 | K3 |
| http://n2t.net/ark:/65665/378fa708e-4cef-4aa0-bc21-42d3a3adab49 | Rodentia | Muridae | Gerbillus nancillus | 17.409000 | -16.061000 | L3 |
| http://n2t.net/ark:/65665/37a361905-36c4-4cb0-b537-3215300bea09 | Rodentia | Muridae | Gerbillus nancillus | 16.150000 | -13.500000 | M5 |
| http://n2t.net/ark:/65665/3e2fafa7a-c055-4d57-b133-7e365627ae77 | Rodentia | Muridae | Gerbillus nancillus | 19.012000 | -15.207000 | J3 |
| http://n2t.net/ark:/65665/37bfd92c3-3347-4e93-b043-d57a7310976b | Rodentia | Muridae | Gerbillus nancillus | 17.030000 | -13.920000 | L5 |
| http://n2t.net/ark:/65665/32b15be1b-3362-4292-b168-0b2a495f4a93 | Rodentia | Muridae | Gerbillus nancillus | 17.030000 | -13.920000 | L5 |
| http://n2t.net/ark:/65665/39874242e-e1ac-490e-91fd-6173016e1373 | Rodentia | Muridae | Gerbillus nancillus | 17.030000 | -13.920000 | L5 |
| http://n2t.net/ark:/65665/366bc9a97-fc4f-4390-8a5d-3d0b285d53c2 | Rodentia | Muridae | Gerbillus nancillus | 17.030000 | -13.920000 | L5 |
| http://n2t.net/ark:/65665/3959071cc-efe0-4671-bb39-a7a834464ef0 | Rodentia | Muridae | Gerbillus nancillus | 17.030000 | -13.920000 | L5 |
| http://coldb.mnhn.fr/catalognumber/mnhn/zm/mo-1997-1461 | Rodentia | Muridae | Gerbillus nigeriae | 17.266783 | -16.028417 | L3 |
| http://coldb.mnhn.fr/catalognumber/mnhn/zm/mo-1997-1471 | Rodentia | Muridae | Gerbillus nigeriae | 19.705000 | -16.390000 | I2 |
| http://coldb.mnhn.fr/catalognumber/mnhn/zm/mo-1997-1470 | Rodentia | Muridae | Gerbillus nigeriae | 19.705000 | -16.390000 | I2 |
| http://coldb.mnhn.fr/catalognumber/mnhn/zm/mo-1997-1462 | Rodentia | Muridae | Gerbillus nigeriae | 19.705000 | -16.390000 | I2 |
| http://coldb.mnhn.fr/catalognumber/mnhn/zm/mo-1997-1463 | Rodentia | Muridae | Gerbillus nigeriae | 18.955000 | -16.126700 | J2 |
| http://coldb.mnhn.fr/catalognumber/mnhn/zm/mo-1997-1469 | Rodentia | Muridae | Gerbillus nigeriae | 18.694000 | -16.102720 | J3 |
| http://coldb.mnhn.fr/catalognumber/mnhn/zm/mo-1997-1472 | Rodentia | Muridae | Gerbillus nigeriae | 17.517000 | -16.033300 | K3 |
| http://coldb.mnhn.fr/catalognumber/mnhn/zm/mo-1997-1460 | Rodentia | Muridae | Gerbillus nigeriae | 17.486000 | -16.023100 | L3 |
| http://coldb.mnhn.fr/catalognumber/mnhn/zm/mo-1969-229 | Rodentia | Muridae | Gerbillus nigeriae | 16.520000 | -15.820000 | M3 |
| http://coldb.mnhn.fr/catalognumber/mnhn/zm/mo-1997-1466 | Rodentia | Muridae | Gerbillus nigeriae | 18.267000 | -16.016900 | K3 |
| http://coldb.mnhn.fr/catalognumber/mnhn/zm/mo-1997-1458 | Rodentia | Muridae | Gerbillus nigeriae | 17.950000 | -12.267000 | K7 |
| http://coldb.mnhn.fr/catalognumber/mnhn/zm/mo-1997-1457 | Rodentia | Muridae | Gerbillus nigeriae | 17.467000 | -13.150000 | K6 |
| http://coldb.mnhn.fr/catalognumber/mnhn/zm/mo-1997-1477 | Rodentia | Muridae | Gerbillus nigeriae | 19.712360 | -16.482097 | I2 |
| http://coldb.mnhn.fr/catalognumber/mnhn/zm/mo-1997-1464 | Rodentia | Muridae | Gerbillus nigeriae | 19.712360 | -16.482097 | I2 |
| http://coldb.mnhn.fr/catalognumber/mnhn/zm/mo-1992-1401 | Rodentia | Muridae | Gerbillus nigeriae | 20.050000 | -16.217000 | I2 |
| http://coldb.mnhn.fr/catalognumber/mnhn/zm/mo-1992-1400 | Rodentia | Muridae | Gerbillus nigeriae | 20.050000 | -16.217000 | I2 |
| http://coldb.mnhn.fr/catalognumber/mnhn/zm/mo-1992-1399 | Rodentia | Muridae | Gerbillus nigeriae | 20.050000 | -16.217000 | I2 |
| http://coldb.mnhn.fr/catalognumber/mnhn/zm/mo-1992-1398 | Rodentia | Muridae | Gerbillus nigeriae | 20.050000 | -16.217000 | I2 |
| http://coldb.mnhn.fr/catalognumber/mnhn/zm/mo-1992-1397 | Rodentia | Muridae | Gerbillus nigeriae | 20.050000 | -16.217000 | I2 |
| http://coldb.mnhn.fr/catalognumber/mnhn/zm/mo-1997-1459 | Rodentia | Muridae | Gerbillus nigeriae | 20.050000 | -16.217000 | I2 |
| http://coldb.mnhn.fr/catalognumber/mnhn/zm/mo-1997-1478 | Rodentia | Muridae | Gerbillus nigeriae | 19.083330 | -16.233300 | J2 |
| http://coldb.mnhn.fr/catalognumber/mnhn/zm/mo-1997-1465 | Rodentia | Muridae | Gerbillus nigeriae | 19.083330 | -16.233300 | J2 |
| http://coldb.mnhn.fr/catalognumber/mnhn/zm/mo-1997-1474 | Rodentia | Muridae | Gerbillus nigeriae | 16.386000 | -16.467000 | M2 |
| http://coldb.mnhn.fr/catalognumber/mnhn/zm/mo-1997-1476 | Rodentia | Muridae | Gerbillus nigeriae | 16.375000 | -16.466000 | M2 |
| http://coldb.mnhn.fr/catalognumber/mnhn/zm/2004-144 | Rodentia | Muridae | Gerbillus nigeriae | 16.372000 | -16.462000 | M2 |
| http://coldb.mnhn.fr/catalognumber/mnhn/zm/mo-1997-1473 | Rodentia | Muridae | Gerbillus nigeriae | 16.619440 | -16.436660 | L2 |
| http://coldb.mnhn.fr/catalognumber/mnhn/zm/mo-2001-79 | Rodentia | Muridae | Gerbillus nigeriae | 16.619440 | -16.436660 | L2 |
| http://coldb.mnhn.fr/catalognumber/mnhn/zm/mo-2001-78 | Rodentia | Muridae | Gerbillus nigeriae | 16.619440 | -16.436660 | L2 |
| http://coldb.mnhn.fr/catalognumber/mnhn/zm/mo-2001-77 | Rodentia | Muridae | Gerbillus nigeriae | 16.619440 | -16.436660 | L2 |
| http://coldb.mnhn.fr/catalognumber/mnhn/zm/mo-2001-76 | Rodentia | Muridae | Gerbillus nigeriae | 16.619440 | -16.436660 | L2 |
| http://coldb.mnhn.fr/catalognumber/mnhn/zm/mo-2001-75 | Rodentia | Muridae | Gerbillus nigeriae | 16.619440 | -16.436660 | L2 |
| http://coldb.mnhn.fr/catalognumber/mnhn/zm/mo-2001-74 | Rodentia | Muridae | Gerbillus nigeriae | 16.619440 | -16.436660 | L2 |
| http://coldb.mnhn.fr/catalognumber/mnhn/zm/mo-2001-73 | Rodentia | Muridae | Gerbillus nigeriae | 16.619440 | -16.436660 | L2 |
| http://coldb.mnhn.fr/catalognumber/mnhn/zm/mo-2001-71 | Rodentia | Muridae | Gerbillus nigeriae | 16.619440 | -16.436660 | L2 |
| http://coldb.mnhn.fr/catalognumber/mnhn/zm/mo-2001-70 | Rodentia | Muridae | Gerbillus nigeriae | 16.619440 | -16.436660 | L2 |
| http://coldb.mnhn.fr/catalognumber/mnhn/zm/mo-2001-69 | Rodentia | Muridae | Gerbillus nigeriae | 16.619440 | -16.436660 | L2 |
| http://coldb.mnhn.fr/catalognumber/mnhn/zm/mo-2001-68 | Rodentia | Muridae | Gerbillus nigeriae | 16.619440 | -16.436660 | L2 |
| http://coldb.mnhn.fr/catalognumber/mnhn/zm/mo-1997-1467 | Rodentia | Muridae | Gerbillus nigeriae | 16.619440 | -16.436660 | L2 |
| http://coldb.mnhn.fr/catalognumber/mnhn/zm/mo-1997-1468 | Rodentia | Muridae | Gerbillus nigeriae | 20.687000 | -16.674000 | H2 |
| http://coldb.mnhn.fr/catalognumber/mnhn/zm/mo-2001-396 | Rodentia | Muridae | Gerbillus pyramidum | 18.955000 | -16.126700 | J2 |
| http://coldb.mnhn.fr/catalognumber/mnhn/zm/mo-2001-393 | Rodentia | Muridae | Gerbillus pyramidum | 18.955000 | -16.126700 | J2 |
| http://coldb.mnhn.fr/catalognumber/mnhn/zm/mo-2001-392 | Rodentia | Muridae | Gerbillus pyramidum | 18.955000 | -16.126700 | J2 |
| http://coldb.mnhn.fr/catalognumber/mnhn/zm/mo-2001-712 | Rodentia | Muridae | Gerbillus pyramidum | 17.429000 | -16.066000 | L3 |
| http://coldb.mnhn.fr/catalognumber/mnhn/zm/mo-2001-711 | Rodentia | Muridae | Gerbillus pyramidum | 17.429000 | -16.066000 | L3 |
| http://coldb.mnhn.fr/catalognumber/mnhn/zm/mo-2001-710 | Rodentia | Muridae | Gerbillus pyramidum | 17.429000 | -16.066000 | L3 |
| http://coldb.mnhn.fr/catalognumber/mnhn/zm/mo-2001-709 | Rodentia | Muridae | Gerbillus pyramidum | 17.433000 | -14.093000 | L5 |
| http://coldb.mnhn.fr/catalognumber/mnhn/zm/mo-2001-708 | Rodentia | Muridae | Gerbillus pyramidum | 17.433000 | -14.093000 | L5 |
| http://coldb.mnhn.fr/catalognumber/mnhn/zm/mo-2001-707 | Rodentia | Muridae | Gerbillus pyramidum | 17.433000 | -14.093000 | L5 |
| http://coldb.mnhn.fr/catalognumber/mnhn/zm/mo-2001-697 | Rodentia | Muridae | Gerbillus pyramidum | 17.434000 | -16.065000 | L3 |
| http://coldb.mnhn.fr/catalognumber/mnhn/zm/mo-2001-696 | Rodentia | Muridae | Gerbillus pyramidum | 17.434000 | -16.065000 | L3 |
| http://coldb.mnhn.fr/catalognumber/mnhn/zm/mo-2001-695 | Rodentia | Muridae | Gerbillus pyramidum | 17.434000 | -16.065000 | L3 |
| http://coldb.mnhn.fr/catalognumber/mnhn/zm/mo-2001-694 | Rodentia | Muridae | Gerbillus pyramidum | 17.434000 | -16.065000 | L3 |
| http://coldb.mnhn.fr/catalognumber/mnhn/zm/mo-2001-706 | Rodentia | Muridae | Gerbillus pyramidum | 17.433000 | -16.072000 | L3 |
| http://coldb.mnhn.fr/catalognumber/mnhn/zm/mo-2001-705 | Rodentia | Muridae | Gerbillus pyramidum | 17.433000 | -16.072000 | L3 |
| http://coldb.mnhn.fr/catalognumber/mnhn/zm/mo-2001-704 | Rodentia | Muridae | Gerbillus pyramidum | 17.433000 | -16.072000 | L3 |
| http://coldb.mnhn.fr/catalognumber/mnhn/zm/mo-2001-703 | Rodentia | Muridae | Gerbillus pyramidum | 17.433000 | -16.072000 | L3 |
| http://coldb.mnhn.fr/catalognumber/mnhn/zm/mo-2001-702 | Rodentia | Muridae | Gerbillus pyramidum | 17.433000 | -16.072000 | L3 |
| http://coldb.mnhn.fr/catalognumber/mnhn/zm/mo-2001-701 | Rodentia | Muridae | Gerbillus pyramidum | 17.433000 | -16.072000 | L3 |
| http://coldb.mnhn.fr/catalognumber/mnhn/zm/mo-2001-700 | Rodentia | Muridae | Gerbillus pyramidum | 17.433000 | -16.072000 | L3 |
| http://coldb.mnhn.fr/catalognumber/mnhn/zm/mo-2001-699 | Rodentia | Muridae | Gerbillus pyramidum | 17.433000 | -16.072000 | L3 |
| http://coldb.mnhn.fr/catalognumber/mnhn/zm/mo-2001-698 | Rodentia | Muridae | Gerbillus pyramidum | 17.433000 | -16.072000 | L3 |
| http://coldb.mnhn.fr/catalognumber/mnhn/zm/mo-2001-693 | Rodentia | Muridae | Gerbillus pyramidum | 17.422000 | -16.064000 | L3 |
| http://coldb.mnhn.fr/catalognumber/mnhn/zm/mo-2001-692 | Rodentia | Muridae | Gerbillus pyramidum | 17.422000 | -16.064000 | L3 |
| http://coldb.mnhn.fr/catalognumber/mnhn/zm/mo-2001-691 | Rodentia | Muridae | Gerbillus pyramidum | 17.422000 | -16.064000 | L3 |
| http://coldb.mnhn.fr/catalognumber/mnhn/zm/mo-2001-690 | Rodentia | Muridae | Gerbillus pyramidum | 17.422000 | -16.064000 | L3 |
| http://coldb.mnhn.fr/catalognumber/mnhn/zm/mo-2001-689 | Rodentia | Muridae | Gerbillus pyramidum | 17.422000 | -16.064000 | L3 |
| http://coldb.mnhn.fr/catalognumber/mnhn/zm/mo-2001-688 | Rodentia | Muridae | Gerbillus pyramidum | 17.422000 | -16.064000 | L3 |
| http://coldb.mnhn.fr/catalognumber/mnhn/zm/mo-2001-687 | Rodentia | Muridae | Gerbillus pyramidum | 17.422000 | -16.064000 | L3 |
| http://coldb.mnhn.fr/catalognumber/mnhn/zm/mo-2001-686 | Rodentia | Muridae | Gerbillus pyramidum | 17.422000 | -16.064000 | L3 |
| JN652810 | Rodentia | Muridae | Gerbillus pyramidum | 20.272200 | -13.120600 | H6 |
| http://coldb.mnhn.fr/catalognumber/mnhn/zm/mo-2001-719 | Rodentia | Muridae | Gerbillus pyramidum | 18.267000 | -16.016900 | K3 |
| http://coldb.mnhn.fr/catalognumber/mnhn/zm/mo-2001-718 | Rodentia | Muridae | Gerbillus pyramidum | 18.267000 | -16.016900 | K3 |
| http://coldb.mnhn.fr/catalognumber/mnhn/zm/mo-2001-717 | Rodentia | Muridae | Gerbillus pyramidum | 18.267000 | -16.016900 | K3 |
| http://coldb.mnhn.fr/catalognumber/mnhn/zm/mo-2001-716 | Rodentia | Muridae | Gerbillus pyramidum | 18.267000 | -16.016900 | K3 |
| http://coldb.mnhn.fr/catalognumber/mnhn/zm/mo-1992-1396b | Rodentia | Muridae | Gerbillus pyramidum | 18.115564 | -16.015721 | K3 |
| http://coldb.mnhn.fr/catalognumber/mnhn/zm/mo-1992-1396a | Rodentia | Muridae | Gerbillus pyramidum | 18.115564 | -16.015721 | K3 |
| http://coldb.mnhn.fr/catalognumber/mnhn/zm/mo-1969-237 | Rodentia | Muridae | Gerbillus pyramidum | 19.831000 | -15.923000 | I3 |
| http://coldb.mnhn.fr/catalognumber/mnhn/zm/mo-2001-382 | Rodentia | Muridae | Gerbillus pyramidum | 18.616000 | -16.103300 | J3 |
| http://coldb.mnhn.fr/catalognumber/mnhn/zm/mo-2001-380 | Rodentia | Muridae | Gerbillus pyramidum | 18.616000 | -16.103300 | J3 |
| http://coldb.mnhn.fr/catalognumber/mnhn/zm/mo-2001-379 | Rodentia | Muridae | Gerbillus pyramidum | 18.616000 | -16.103300 | J3 |
| http://coldb.mnhn.fr/catalognumber/mnhn/zm/mo-2001-377 | Rodentia | Muridae | Gerbillus pyramidum | 18.616000 | -16.103300 | J3 |
| http://coldb.mnhn.fr/catalognumber/mnhn/zm/mo-2001-376 | Rodentia | Muridae | Gerbillus pyramidum | 18.616000 | -16.103300 | J3 |
| http://coldb.mnhn.fr/catalognumber/mnhn/zm/mo-2001-375 | Rodentia | Muridae | Gerbillus pyramidum | 18.616000 | -16.103300 | J3 |
| http://coldb.mnhn.fr/catalognumber/mnhn/zm/mo-2001-374 | Rodentia | Muridae | Gerbillus pyramidum | 18.616000 | -16.103300 | J3 |
| http://coldb.mnhn.fr/catalognumber/mnhn/zm/mo-2001-372 | Rodentia | Muridae | Gerbillus pyramidum | 18.616000 | -16.103300 | J3 |
| http://coldb.mnhn.fr/catalognumber/mnhn/zm/mo-2001-370 | Rodentia | Muridae | Gerbillus pyramidum | 18.616000 | -16.103300 | J3 |
| http://coldb.mnhn.fr/catalognumber/mnhn/zm/mo-2001-245 | Rodentia | Muridae | Gerbillus pyramidum | 16.634000 | -15.483000 | L3 |
| http://coldb.mnhn.fr/catalognumber/mnhn/zm/mo-2001-244 | Rodentia | Muridae | Gerbillus pyramidum | 16.634000 | -15.483000 | L3 |
| http://coldb.mnhn.fr/catalognumber/mnhn/zm/mo-2001-391 | Rodentia | Muridae | Gerbillus pyramidum | 19.100000 | -16.273000 | J2 |
| http://coldb.mnhn.fr/catalognumber/mnhn/zm/mo-2001-390 | Rodentia | Muridae | Gerbillus pyramidum | 19.100000 | -16.273000 | J2 |
| http://coldb.mnhn.fr/catalognumber/mnhn/zm/mo-2001-389 | Rodentia | Muridae | Gerbillus pyramidum | 19.100000 | -16.273000 | J2 |
| http://coldb.mnhn.fr/catalognumber/mnhn/zm/mo-2001-386 | Rodentia | Muridae | Gerbillus pyramidum | 19.100000 | -16.273000 | J2 |
| http://coldb.mnhn.fr/catalognumber/mnhn/zm/mo-2001-384 | Rodentia | Muridae | Gerbillus pyramidum | 19.100000 | -16.273000 | J2 |
| http://coldb.mnhn.fr/catalognumber/mnhn/zm/mo-2001-167 | Rodentia | Muridae | Gerbillus pyramidum | 18.767000 | -15.317000 | J3 |
| http://coldb.mnhn.fr/catalognumber/mnhn/zm/mo-2001-159 | Rodentia | Muridae | Gerbillus pyramidum | 18.767000 | -15.317000 | J3 |
| JN652811 | Rodentia | Muridae | Gerbillus pyramidum | 19.740000 | -14.370000 | I4 |
| http://n2t.net/ark:/65665/3470bc0c5-55bb-4a94-be39-dfe6c292a4e3 | Rodentia | Muridae | Gerbillus sp. | 17.266783 | -16.028417 | L3 |
| http://n2t.net/ark:/65665/3c1583d75-eac5-40b6-8164-5b208027e6d6 | Rodentia | Muridae | Gerbillus sp. | 17.266783 | -16.028417 | L3 |
| http://n2t.net/ark:/65665/31369bbdf-0fb1-4813-a957-456a64d06e05 | Rodentia | Muridae | Gerbillus sp. | 17.266783 | -16.028417 | L3 |
| http://n2t.net/ark:/65665/355b89c65-0bcc-489d-a4e0-7295a8a82812 | Rodentia | Muridae | Gerbillus sp. | 17.266783 | -16.028417 | L3 |
| http://n2t.net/ark:/65665/350825aeb-3ed9-44f3-a320-b63528ee874d | Rodentia | Muridae | Gerbillus sp. | 17.266783 | -16.028417 | L3 |
| http://n2t.net/ark:/65665/3b97bb2b8-3137-4ffe-bdec-5d3fc03cd4e6 | Rodentia | Muridae | Gerbillus sp. | 17.266783 | -16.028417 | L3 |
| http://n2t.net/ark:/65665/3b7bd174b-ecb2-437a-a979-16759914fa97 | Rodentia | Muridae | Gerbillus sp. | 17.266783 | -16.028417 | L3 |
| http://n2t.net/ark:/65665/30eb43fc7-1559-40ba-8d0c-9046a4f79ab6 | Rodentia | Muridae | Gerbillus sp. | 17.266783 | -16.028417 | L3 |
| http://n2t.net/ark:/65665/31ca7c1d8-721c-41a2-90d6-1bbeed2b012b | Rodentia | Muridae | Gerbillus sp. | 17.266783 | -16.028417 | L3 |
| http://n2t.net/ark:/65665/370fab79e-bba7-454b-a4df-22268ecaa517 | Rodentia | Muridae | Gerbillus sp. | 17.266783 | -16.028417 | L3 |
| http://n2t.net/ark:/65665/34308d5ae-b5dc-480a-a26f-52881c96aabb | Rodentia | Muridae | Gerbillus sp. | 17.266783 | -16.028417 | L3 |
| http://n2t.net/ark:/65665/3180e38dc-57d8-48b8-937f-1385aa182bbd | Rodentia | Muridae | Gerbillus sp. | 17.266783 | -16.028417 | L3 |
| http://n2t.net/ark:/65665/3a9454b45-8e98-4da6-81d1-895727fbd515 | Rodentia | Muridae | Gerbillus sp. | 17.266783 | -16.028417 | L3 |
| http://n2t.net/ark:/65665/3d9f37944-60f1-43dc-bf85-e5e1ad2f74dc | Rodentia | Muridae | Gerbillus sp. | 17.266783 | -16.028417 | L3 |
| http://n2t.net/ark:/65665/3884a88a4-8115-44cf-be12-40b694b6a320 | Rodentia | Muridae | Gerbillus sp. | 17.266783 | -16.028417 | L3 |
| http://n2t.net/ark:/65665/3af885bf5-f5cc-4ec8-96f1-1d6f5ab617bf | Rodentia | Muridae | Gerbillus sp. | 17.266783 | -16.028417 | L3 |
| http://n2t.net/ark:/65665/3b1ae03be-47c8-4aae-9f80-e105aad4cddb | Rodentia | Muridae | Gerbillus sp. | 17.266783 | -16.028417 | L3 |
| http://n2t.net/ark:/65665/358e42e35-92ed-4031-b875-d5dd05d719f2 | Rodentia | Muridae | Gerbillus sp. | 17.266783 | -16.028417 | L3 |
| http://n2t.net/ark:/65665/3fc709d5e-388b-446e-b9dc-b8d6df96221d | Rodentia | Muridae | Gerbillus sp. | 17.266783 | -16.028417 | L3 |
| http://n2t.net/ark:/65665/3f4ef0963-9a42-48ba-8918-af825fe17456 | Rodentia | Muridae | Gerbillus sp. | 17.266783 | -16.028417 | L3 |
| http://n2t.net/ark:/65665/39c80b0b9-46ad-41ab-8c55-9cda4e6ce481 | Rodentia | Muridae | Gerbillus sp. | 17.266783 | -16.028417 | L3 |
| http://n2t.net/ark:/65665/3999ae7c2-8988-48c6-b59b-e3595c7b1bfd | Rodentia | Muridae | Gerbillus sp. | 17.266783 | -16.028417 | L3 |
| http://n2t.net/ark:/65665/339f4d4d8-2ccd-4852-a628-23b3b27a644f | Rodentia | Muridae | Gerbillus sp. | 17.266783 | -16.028417 | L3 |
| http://n2t.net/ark:/65665/3efeb5d79-5522-4294-908d-3a4fce4766ae | Rodentia | Muridae | Gerbillus sp. | 17.266783 | -16.028417 | L3 |
| http://n2t.net/ark:/65665/351a9d8e3-c2a4-4c66-88af-f03f58c620f1 | Rodentia | Muridae | Gerbillus sp. | 17.266783 | -16.028417 | L3 |
| http://n2t.net/ark:/65665/33a23bb42-3bb1-4c18-ac47-f2d142b314fc | Rodentia | Muridae | Gerbillus sp. | 17.266783 | -16.028417 | L3 |
| http://n2t.net/ark:/65665/3336cc362-c04d-496a-9281-b4a1e52aafd5 | Rodentia | Muridae | Gerbillus sp. | 17.266783 | -16.028417 | L3 |
| http://n2t.net/ark:/65665/34b348f54-d959-45f1-9f82-44408d40ce8a | Rodentia | Muridae | Gerbillus sp. | 17.266783 | -16.028417 | L3 |
| http://n2t.net/ark:/65665/3db8f1314-9d56-43de-93fc-a1f230ab8f3a | Rodentia | Muridae | Gerbillus sp. | 17.266783 | -16.028417 | L3 |
| http://n2t.net/ark:/65665/3d538acf5-9a8a-46e2-9da7-53c71afc5013 | Rodentia | Muridae | Gerbillus sp. | 17.266783 | -16.028417 | L3 |
| http://n2t.net/ark:/65665/3902f25d1-4199-4900-bba3-37064e17148b | Rodentia | Muridae | Gerbillus sp. | 17.266783 | -16.028417 | L3 |
| http://n2t.net/ark:/65665/3f6458f11-15ca-4566-8b21-5f3350cfaca8 | Rodentia | Muridae | Gerbillus sp. | 17.266783 | -16.028417 | L3 |
| http://n2t.net/ark:/65665/3baa9ed37-070e-4939-8d20-5a5e927d7f8d | Rodentia | Muridae | Gerbillus sp. | 17.266783 | -16.028417 | L3 |
| http://n2t.net/ark:/65665/340a7f797-94ef-4500-8023-3e7db58c8971 | Rodentia | Muridae | Gerbillus sp. | 17.266783 | -16.028417 | L3 |
| http://n2t.net/ark:/65665/32685ce71-749c-4d47-90da-2931d87310a9 | Rodentia | Muridae | Gerbillus sp. | 17.266783 | -16.028417 | L3 |
| http://n2t.net/ark:/65665/3ce78355f-c2a4-4f65-8c4c-66de592c3c03 | Rodentia | Muridae | Gerbillus sp. | 17.266783 | -16.028417 | L3 |
| http://n2t.net/ark:/65665/3b5b9041c-1409-42c1-90f5-bf36a62264bd | Rodentia | Muridae | Gerbillus sp. | 17.266783 | -16.028417 | L3 |
| http://n2t.net/ark:/65665/3f7827c74-6239-4531-a5f0-e5627521058f | Rodentia | Muridae | Gerbillus sp. | 17.266783 | -16.028417 | L3 |
| http://n2t.net/ark:/65665/3f4519ebc-055d-4bda-a609-df587a3730ec | Rodentia | Muridae | Gerbillus sp. | 17.266783 | -16.028417 | L3 |
| http://n2t.net/ark:/65665/3d0fd67ab-d191-4023-bc40-9b3e0a158c4e | Rodentia | Muridae | Gerbillus sp. | 17.266783 | -16.028417 | L3 |
| http://n2t.net/ark:/65665/3f6aaaadf-eafb-423f-b590-03e28dfc7bf6 | Rodentia | Muridae | Gerbillus sp. | 17.266783 | -16.028417 | L3 |
| http://n2t.net/ark:/65665/373acb5d7-17b0-4c9f-8fad-b25689d634a8 | Rodentia | Muridae | Gerbillus sp. | 17.266783 | -16.028417 | L3 |
| http://n2t.net/ark:/65665/349531a9c-49eb-4958-ab44-bcdd4147747a | Rodentia | Muridae | Gerbillus sp. | 17.266783 | -16.028417 | L3 |
| http://n2t.net/ark:/65665/3d481d44c-d9d8-4aca-a310-1345d2febc43 | Rodentia | Muridae | Gerbillus sp. | 17.266783 | -16.028417 | L3 |
| http://n2t.net/ark:/65665/3e17868c7-f462-4476-a9ee-94384ab1241a | Rodentia | Muridae | Gerbillus sp. | 17.266783 | -16.028417 | L3 |
| http://n2t.net/ark:/65665/327e7f929-4363-408f-80a6-842a860c699c | Rodentia | Muridae | Gerbillus sp. | 17.266783 | -16.028417 | L3 |
| http://n2t.net/ark:/65665/33ff1d3b9-72bd-4285-8b3e-d47a38b25e9c | Rodentia | Muridae | Gerbillus sp. | 17.266783 | -16.028417 | L3 |
| http://n2t.net/ark:/65665/3f1c839d1-a4b9-4faf-ac2b-9b619209615f | Rodentia | Muridae | Gerbillus sp. | 17.266783 | -16.028417 | L3 |
| http://n2t.net/ark:/65665/3ae1a19f4-1011-4d1a-a8a3-0ca0cb010ecc | Rodentia | Muridae | Gerbillus sp. | 17.266783 | -16.028417 | L3 |
| http://n2t.net/ark:/65665/37f9166fe-2789-406e-9ae3-346c4a69f304 | Rodentia | Muridae | Gerbillus sp. | 17.266783 | -16.028417 | L3 |
| http://n2t.net/ark:/65665/3a277f172-4cba-4c50-a7cd-8fda84c2a50d | Rodentia | Muridae | Gerbillus sp. | 17.266783 | -16.028417 | L3 |
| http://n2t.net/ark:/65665/38da37cc9-c359-4e03-9441-cf1b4be9d740 | Rodentia | Muridae | Gerbillus sp. | 17.266783 | -16.028417 | L3 |
| http://n2t.net/ark:/65665/32d008d4c-98b1-4597-a423-464142579f05 | Rodentia | Muridae | Gerbillus sp. | 17.266783 | -16.028417 | L3 |
| http://n2t.net/ark:/65665/3ac64ce9d-97d1-4df4-9b45-89176c247333 | Rodentia | Muridae | Gerbillus sp. | 17.266783 | -16.028417 | L3 |
| http://n2t.net/ark:/65665/35ae8edec-686d-42a8-a24e-ed9c258f3e66 | Rodentia | Muridae | Gerbillus sp. | 17.266783 | -16.028417 | L3 |
| http://n2t.net/ark:/65665/3e0d8382e-a117-477b-b18f-84470d1dbfac | Rodentia | Muridae | Gerbillus sp. | 17.266783 | -16.028417 | L3 |
| http://n2t.net/ark:/65665/394e0c4bb-6e92-49cf-a22a-1c8ad1e96691 | Rodentia | Muridae | Gerbillus sp. | 17.266783 | -16.028417 | L3 |
| http://n2t.net/ark:/65665/3516e61ab-71bd-402f-ac43-042ef078b7d4 | Rodentia | Muridae | Gerbillus sp. | 17.266783 | -16.028417 | L3 |
| http://n2t.net/ark:/65665/355750c15-d2c9-4683-8d8c-c50d70b017b8 | Rodentia | Muridae | Gerbillus sp. | 17.266783 | -16.028417 | L3 |
| http://n2t.net/ark:/65665/3ef0a276a-870c-4803-bcee-18719e905508 | Rodentia | Muridae | Gerbillus sp. | 17.266783 | -16.028417 | L3 |
| http://n2t.net/ark:/65665/34e5ba5f2-a658-44a2-b230-b812c37fee58 | Rodentia | Muridae | Gerbillus sp. | 17.266783 | -16.028417 | L3 |
| http://n2t.net/ark:/65665/3a03adebc-d688-4dbb-a6e9-50c969a69b02 | Rodentia | Muridae | Gerbillus sp. | 17.266783 | -16.028417 | L3 |
| http://n2t.net/ark:/65665/3f1c0b6b0-5be7-46e2-810a-e051b76512ec | Rodentia | Muridae | Gerbillus sp. | 17.266783 | -16.028417 | L3 |
| http://n2t.net/ark:/65665/38a987923-d9b2-4912-8450-605b7f7f5f25 | Rodentia | Muridae | Gerbillus sp. | 17.266783 | -16.028417 | L3 |
| http://n2t.net/ark:/65665/33e312a30-d294-4ae6-a28d-c81be19dd10a | Rodentia | Muridae | Gerbillus sp. | 17.266783 | -16.028417 | L3 |
| http://n2t.net/ark:/65665/35f8a3c08-be86-4b94-bb88-eb8ad89ab233 | Rodentia | Muridae | Gerbillus sp. | 17.266783 | -16.028417 | L3 |
| http://n2t.net/ark:/65665/310f1b9b0-d8b6-4caa-9347-df250a2fde3c | Rodentia | Muridae | Gerbillus sp. | 17.266783 | -16.028417 | L3 |
| http://n2t.net/ark:/65665/357d41590-61b5-41ce-b3a2-d7d9083a28cb | Rodentia | Muridae | Gerbillus sp. | 17.266783 | -16.028417 | L3 |
| http://n2t.net/ark:/65665/3823637fa-e449-4b3d-aa8c-f5476c04663c | Rodentia | Muridae | Gerbillus sp. | 17.266783 | -16.028417 | L3 |
| http://n2t.net/ark:/65665/34ee29b69-a0e2-4d02-8e1c-ff1c7a984bb8 | Rodentia | Muridae | Gerbillus sp. | 17.266783 | -16.028417 | L3 |
| http://n2t.net/ark:/65665/363eb984c-3753-4462-98c3-9d06042a7bb8 | Rodentia | Muridae | Gerbillus sp. | 17.266783 | -16.028417 | L3 |
| http://n2t.net/ark:/65665/3d07afeef-6df9-4270-a749-66e587cf9e8d | Rodentia | Muridae | Gerbillus sp. | 17.266783 | -16.028417 | L3 |
| http://n2t.net/ark:/65665/30f9c54ae-6563-4a01-902d-33ec9f4b6468 | Rodentia | Muridae | Gerbillus sp. | 17.266783 | -16.028417 | L3 |
| http://n2t.net/ark:/65665/3087264fa-9eb6-4253-8663-1e3f6e579af6 | Rodentia | Muridae | Gerbillus sp. | 17.266783 | -16.028417 | L3 |
| http://n2t.net/ark:/65665/36475941c-ea93-436e-b329-c255410ed707 | Rodentia | Muridae | Gerbillus sp. | 17.266783 | -16.028417 | L3 |
| http://n2t.net/ark:/65665/35a6d2cc5-26c7-4443-8834-a2806e5b4aac | Rodentia | Muridae | Gerbillus sp. | 17.266783 | -16.028417 | L3 |
| http://n2t.net/ark:/65665/30e3b82c3-acae-4b25-9eea-d0d7fd3244fd | Rodentia | Muridae | Gerbillus sp. | 17.266783 | -16.028417 | L3 |
| http://n2t.net/ark:/65665/3901ca5c2-18d1-4220-91ee-b015ab15573e | Rodentia | Muridae | Gerbillus sp. | 17.266783 | -16.028417 | L3 |
| http://n2t.net/ark:/65665/38c0c0737-b6b5-43e1-bd5f-a5a8ec0a69e0 | Rodentia | Muridae | Gerbillus sp. | 17.266783 | -16.028417 | L3 |
| http://n2t.net/ark:/65665/3181200ce-f994-4630-b14c-54a1fb8344a6 | Rodentia | Muridae | Gerbillus sp. | 17.266783 | -16.028417 | L3 |
| http://n2t.net/ark:/65665/3723d449e-6cc4-4486-84b3-f505a872a7bb | Rodentia | Muridae | Gerbillus sp. | 17.266783 | -16.028417 | L3 |
| http://n2t.net/ark:/65665/3bbe8d5af-0ed6-4048-a7b7-7ee5b14bea6b | Rodentia | Muridae | Gerbillus sp. | 17.266783 | -16.028417 | L3 |
| http://n2t.net/ark:/65665/3d6ca0c1e-7d73-43ac-acb8-11fe9eb4fbe4 | Rodentia | Muridae | Gerbillus sp. | 17.266783 | -16.028417 | L3 |
| http://n2t.net/ark:/65665/3f84ea710-cd13-4a2a-b489-086b3cbd08ab | Rodentia | Muridae | Gerbillus sp. | 17.266783 | -16.028417 | L3 |
| http://n2t.net/ark:/65665/317698581-cd72-4b10-9de1-be1cc50a20cb | Rodentia | Muridae | Gerbillus sp. | 17.266783 | -16.028417 | L3 |
| http://n2t.net/ark:/65665/347f07e24-6fa9-4364-85ed-9076b8f0a9f5 | Rodentia | Muridae | Gerbillus sp. | 17.266783 | -16.028417 | L3 |
| http://n2t.net/ark:/65665/3f4aa52ab-efdc-47f8-b434-aef3fc4f1209 | Rodentia | Muridae | Gerbillus sp. | 17.266783 | -16.028417 | L3 |
| http://n2t.net/ark:/65665/3f5c7a23d-c5a2-4bef-877c-bbc307271cde | Rodentia | Muridae | Gerbillus sp. | 17.266783 | -16.028417 | L3 |
| http://n2t.net/ark:/65665/327e94a5f-ce6e-4188-98e1-9268ef34606d | Rodentia | Muridae | Gerbillus sp. | 17.266783 | -16.028417 | L3 |
| http://n2t.net/ark:/65665/30173d619-55b8-4876-8c87-0a024898a63e | Rodentia | Muridae | Gerbillus sp. | 17.266783 | -16.028417 | L3 |
| http://n2t.net/ark:/65665/3299f173c-907f-4bd7-8427-54ecd95dfc14 | Rodentia | Muridae | Gerbillus sp. | 17.266783 | -16.028417 | L3 |
| http://n2t.net/ark:/65665/38f491d12-db4a-4376-8d3d-853f486c3d1a | Rodentia | Muridae | Gerbillus sp. | 17.266783 | -16.028417 | L3 |
| http://n2t.net/ark:/65665/318c53cc9-92cd-48ba-9f87-6a3c88ca38d7 | Rodentia | Muridae | Gerbillus sp. | 17.266783 | -16.028417 | L3 |
| http://n2t.net/ark:/65665/3508d5cef-3e0a-4efc-a46b-577b0590cb8b | Rodentia | Muridae | Gerbillus sp. | 17.266783 | -16.028417 | L3 |
| http://n2t.net/ark:/65665/3e5bd3d9e-7e6c-4bab-a349-8b4aa8f0c0ee | Rodentia | Muridae | Gerbillus sp. | 17.266783 | -16.028417 | L3 |
| http://n2t.net/ark:/65665/335ca5bcd-4d3f-4e87-8fb6-77b99be45f70 | Rodentia | Muridae | Gerbillus sp. | 17.266783 | -16.028417 | L3 |
| http://n2t.net/ark:/65665/36096a825-1697-42ff-b1b3-e7e6814334e8 | Rodentia | Muridae | Gerbillus sp. | 17.266783 | -16.028417 | L3 |
| http://n2t.net/ark:/65665/333f3b29f-1e1a-4664-9e20-cc057e1f89ff | Rodentia | Muridae | Gerbillus sp. | 17.266783 | -16.028417 | L3 |
| http://n2t.net/ark:/65665/370841658-0f76-4fed-b16c-95d05007f500 | Rodentia | Muridae | Gerbillus sp. | 17.266783 | -16.028417 | L3 |
| http://n2t.net/ark:/65665/3b8ce5ef4-aaeb-4a9a-8382-535f89c1482d | Rodentia | Muridae | Gerbillus sp. | 17.266783 | -16.028417 | L3 |
| http://n2t.net/ark:/65665/36aa6bab6-dda1-453d-98a8-37ae2c6658f2 | Rodentia | Muridae | Gerbillus sp. | 17.266783 | -16.028417 | L3 |
| http://n2t.net/ark:/65665/3f4c74b8b-f487-4630-bcfb-65a9008dc4af | Rodentia | Muridae | Gerbillus sp. | 17.266783 | -16.028417 | L3 |
| http://n2t.net/ark:/65665/31f3db69b-05e4-41a5-814d-ca39691d81e8 | Rodentia | Muridae | Gerbillus sp. | 17.266783 | -16.028417 | L3 |
| http://n2t.net/ark:/65665/37d088f04-db1b-411d-8a0e-634373eb597f | Rodentia | Muridae | Gerbillus sp. | 17.266783 | -16.028417 | L3 |
| http://n2t.net/ark:/65665/3bc105e61-bcd7-459e-909c-80b4aa284cc0 | Rodentia | Muridae | Gerbillus sp. | 17.266783 | -16.028417 | L3 |
| http://n2t.net/ark:/65665/34d0b0308-c342-4240-b756-1f99f32e4037 | Rodentia | Muridae | Gerbillus sp. | 17.266783 | -16.028417 | L3 |
| http://n2t.net/ark:/65665/3b5659d95-c35d-43cf-b8fe-9e9bd7494d30 | Rodentia | Muridae | Gerbillus sp. | 17.266783 | -16.028417 | L3 |
| http://n2t.net/ark:/65665/383e319f7-57ea-433a-b626-38b21ffa5206 | Rodentia | Muridae | Gerbillus sp. | 17.266783 | -16.028417 | L3 |
| http://n2t.net/ark:/65665/3cb88041e-f629-4b72-89cc-c1f44623648e | Rodentia | Muridae | Gerbillus sp. | 17.266783 | -16.028417 | L3 |
| http://n2t.net/ark:/65665/3a1763a56-5a38-40da-b2b2-818f3da6fb94 | Rodentia | Muridae | Gerbillus sp. | 17.266783 | -16.028417 | L3 |
| http://n2t.net/ark:/65665/32906f142-eefb-4def-902f-b700ab01681b | Rodentia | Muridae | Gerbillus sp. | 17.266783 | -16.028417 | L3 |
| http://n2t.net/ark:/65665/377f09d85-ba74-4f92-aa57-ed6ecf54ea98 | Rodentia | Muridae | Gerbillus sp. | 17.266783 | -16.028417 | L3 |
| http://n2t.net/ark:/65665/345c5327e-32d0-46c9-94c0-d58b3f8735a1 | Rodentia | Muridae | Gerbillus sp. | 17.266783 | -16.028417 | L3 |
| http://n2t.net/ark:/65665/38487bb57-4d82-4c8e-8196-48c110ecc352 | Rodentia | Muridae | Gerbillus sp. | 17.266783 | -16.028417 | L3 |
| http://n2t.net/ark:/65665/3040cbab8-35f3-4abb-a685-63e16120a1e5 | Rodentia | Muridae | Gerbillus sp. | 17.266783 | -16.028417 | L3 |
| http://n2t.net/ark:/65665/3713408ba-4699-4855-b3af-5377f777b12c | Rodentia | Muridae | Gerbillus sp. | 17.266783 | -16.028417 | L3 |
| http://n2t.net/ark:/65665/3221cfc74-38c2-4285-a945-302acdd9cb1b | Rodentia | Muridae | Gerbillus sp. | 17.266783 | -16.028417 | L3 |
| http://n2t.net/ark:/65665/379158030-2514-47eb-9afc-04faf6f4f25e | Rodentia | Muridae | Gerbillus sp. | 17.266783 | -16.028417 | L3 |
| http://n2t.net/ark:/65665/334e977a0-bea2-49cf-8313-e3ec8fca553e | Rodentia | Muridae | Gerbillus sp. | 17.266783 | -16.028417 | L3 |
| http://n2t.net/ark:/65665/3ecdd36c4-7bfe-4ce8-8a7f-5f4b63aa8f45 | Rodentia | Muridae | Gerbillus sp. | 17.266783 | -16.028417 | L3 |
| http://n2t.net/ark:/65665/3f2b02aa4-caf1-42a6-a990-8b2e68eb5004 | Rodentia | Muridae | Gerbillus sp. | 17.266783 | -16.028417 | L3 |
| http://n2t.net/ark:/65665/386e2b2d9-0929-43a6-b1a7-847cfbfa7417 | Rodentia | Muridae | Gerbillus sp. | 17.266783 | -16.028417 | L3 |
| http://n2t.net/ark:/65665/3c38e6be0-8e9a-4c5e-878e-728cc8335aa4 | Rodentia | Muridae | Gerbillus sp. | 17.266783 | -16.028417 | L3 |
| http://n2t.net/ark:/65665/3339189aa-72fe-4c8c-94c1-83f4b94331b0 | Rodentia | Muridae | Gerbillus sp. | 17.266783 | -16.028417 | L3 |
| http://n2t.net/ark:/65665/35b9327bb-e493-491d-8ebe-d8b4a0d03eb2 | Rodentia | Muridae | Gerbillus sp. | 17.266783 | -16.028417 | L3 |
| http://n2t.net/ark:/65665/320e893dc-f35d-4b55-b50c-c162798f4d03 | Rodentia | Muridae | Gerbillus sp. | 17.266783 | -16.028417 | L3 |
| http://n2t.net/ark:/65665/337ecebf7-a634-4c40-879e-8817a4542ca1 | Rodentia | Muridae | Gerbillus sp. | 17.266783 | -16.028417 | L3 |
| http://n2t.net/ark:/65665/384c25a95-567f-4de5-b92e-22135c0a1de4 | Rodentia | Muridae | Gerbillus sp. | 17.266783 | -16.028417 | L3 |
| http://n2t.net/ark:/65665/30f2bdf7f-4602-4ac9-9c35-19d509407b76 | Rodentia | Muridae | Gerbillus sp. | 17.266783 | -16.028417 | L3 |
| http://n2t.net/ark:/65665/3d5010590-0b72-4e67-8cd4-5382bcb7f5ff | Rodentia | Muridae | Gerbillus sp. | 17.266783 | -16.028417 | L3 |
| http://n2t.net/ark:/65665/35f9f07e8-bb65-4748-9cb2-a249ade943af | Rodentia | Muridae | Gerbillus sp. | 17.266783 | -16.028417 | L3 |
| http://n2t.net/ark:/65665/3f03ddd50-6139-43f6-b37d-b997609ba2c2 | Rodentia | Muridae | Gerbillus sp. | 17.266783 | -16.028417 | L3 |
| http://n2t.net/ark:/65665/37c80323a-8df1-4549-91e5-8646f946d40c | Rodentia | Muridae | Gerbillus sp. | 17.266783 | -16.028417 | L3 |
| http://n2t.net/ark:/65665/3c733d37e-2c92-443a-90a9-8a2f7be51e78 | Rodentia | Muridae | Gerbillus sp. | 17.266783 | -16.028417 | L3 |
| http://n2t.net/ark:/65665/36a15e550-d423-4500-aa7c-06631983d2fe | Rodentia | Muridae | Gerbillus sp. | 17.266783 | -16.028417 | L3 |
| http://n2t.net/ark:/65665/32efecc21-8b54-4bbb-8f3b-93e247a7532d | Rodentia | Muridae | Gerbillus sp. | 17.266783 | -16.028417 | L3 |
| http://n2t.net/ark:/65665/342980473-0518-4937-a6e7-b61c0980f478 | Rodentia | Muridae | Gerbillus sp. | 17.266783 | -16.028417 | L3 |
| http://n2t.net/ark:/65665/335da914f-c762-4933-8fe6-d231672a2d4e | Rodentia | Muridae | Gerbillus sp. | 17.266783 | -16.028417 | L3 |
| http://n2t.net/ark:/65665/3cde6761a-f0eb-4972-88cc-c384c776f0aa | Rodentia | Muridae | Gerbillus sp. | 17.266783 | -16.028417 | L3 |
| http://n2t.net/ark:/65665/3d814b417-9bc2-4e7d-bfef-308b6e00838d | Rodentia | Muridae | Gerbillus sp. | 17.266783 | -16.028417 | L3 |
| http://n2t.net/ark:/65665/35d47998c-5554-4f2f-be79-3b8782daf3e8 | Rodentia | Muridae | Gerbillus sp. | 17.266783 | -16.028417 | L3 |
| http://n2t.net/ark:/65665/3269778e1-d67d-491b-81c8-762af6f66fc1 | Rodentia | Muridae | Gerbillus sp. | 17.266783 | -16.028417 | L3 |
| http://n2t.net/ark:/65665/38b6efcc2-83d3-4ffc-928f-c8add051281e | Rodentia | Muridae | Gerbillus sp. | 17.266783 | -16.028417 | L3 |
| http://n2t.net/ark:/65665/3879ef68d-6c64-4d1c-8021-cd9ce614fb20 | Rodentia | Muridae | Gerbillus sp. | 17.266783 | -16.028417 | L3 |
| http://n2t.net/ark:/65665/3fa28f744-dec2-45e3-919f-b43422e361a1 | Rodentia | Muridae | Gerbillus sp. | 17.266783 | -16.028417 | L3 |
| http://n2t.net/ark:/65665/358086f17-e0a5-46d8-9ef8-361e1fad40b9 | Rodentia | Muridae | Gerbillus sp. | 17.266783 | -16.028417 | L3 |
| http://n2t.net/ark:/65665/317e97194-c2c5-4ecc-a301-80e5ee30aa69 | Rodentia | Muridae | Gerbillus sp. | 17.266783 | -16.028417 | L3 |
| http://n2t.net/ark:/65665/370b2c8f7-779f-4e1a-92a0-1493b60d49f9 | Rodentia | Muridae | Gerbillus sp. | 17.266783 | -16.028417 | L3 |
| http://n2t.net/ark:/65665/38693dd30-16cc-45fc-94bf-59eba78c450e | Rodentia | Muridae | Gerbillus sp. | 17.266783 | -16.028417 | L3 |
| http://n2t.net/ark:/65665/3ad7216e1-ec09-4078-881e-05398ffc6060 | Rodentia | Muridae | Gerbillus sp. | 17.266783 | -16.028417 | L3 |
| http://n2t.net/ark:/65665/32ee2e20d-67f4-4112-8f64-2bc2ac4bc404 | Rodentia | Muridae | Gerbillus sp. | 17.266783 | -16.028417 | L3 |
| http://n2t.net/ark:/65665/3175249be-bab8-48db-aab9-7e477e0eb654 | Rodentia | Muridae | Gerbillus sp. | 17.266783 | -16.028417 | L3 |
| http://n2t.net/ark:/65665/3e0f4e9b2-676d-4132-b750-82dc36a7a206 | Rodentia | Muridae | Gerbillus sp. | 17.266783 | -16.028417 | L3 |
| http://n2t.net/ark:/65665/3d808c1b1-457b-4730-a4d2-e51f4f44b4ec | Rodentia | Muridae | Gerbillus sp. | 17.266783 | -16.028417 | L3 |
| http://n2t.net/ark:/65665/316a84f37-b894-447a-9db2-f5b1d6c6bf1b | Rodentia | Muridae | Gerbillus sp. | 17.266783 | -16.028417 | L3 |
| http://n2t.net/ark:/65665/32808ebf7-ef6e-4222-bf74-edd7d0cee801 | Rodentia | Muridae | Gerbillus sp. | 17.266783 | -16.028417 | L3 |
| http://n2t.net/ark:/65665/325353f69-ba0a-4566-bea2-e50143cffa1d | Rodentia | Muridae | Gerbillus sp. | 17.266783 | -16.028417 | L3 |
| http://n2t.net/ark:/65665/386ef1d33-e47c-433f-811f-2572377dc417 | Rodentia | Muridae | Gerbillus sp. | 17.266783 | -16.028417 | L3 |
| http://n2t.net/ark:/65665/36a1e2552-6bea-48ae-a479-a2f2f65885ac | Rodentia | Muridae | Gerbillus sp. | 17.266783 | -16.028417 | L3 |
| http://n2t.net/ark:/65665/3afc98e77-60eb-415f-b580-bdf0feb6ed8b | Rodentia | Muridae | Gerbillus sp. | 17.266783 | -16.028417 | L3 |
| http://n2t.net/ark:/65665/3ed002ffd-cee3-43ee-bf3a-a5e26691b947 | Rodentia | Muridae | Gerbillus sp. | 17.266783 | -16.028417 | L3 |
| http://n2t.net/ark:/65665/3897f85ea-a954-4890-b1da-0582ff084747 | Rodentia | Muridae | Gerbillus sp. | 17.266783 | -16.028417 | L3 |
| http://n2t.net/ark:/65665/3dbd26066-ac5f-41c7-8b0f-b38e72c4ff43 | Rodentia | Muridae | Gerbillus sp. | 17.266783 | -16.028417 | L3 |
| http://n2t.net/ark:/65665/3dd3fdccf-7ad9-48f0-818b-2de8067053cf | Rodentia | Muridae | Gerbillus sp. | 17.266783 | -16.028417 | L3 |
| http://n2t.net/ark:/65665/35295d2a8-0079-4686-9a7e-c24b33d1ceb3 | Rodentia | Muridae | Gerbillus sp. | 17.266783 | -16.028417 | L3 |
| http://n2t.net/ark:/65665/3e959a994-c310-44a7-8147-80aab84b98b1 | Rodentia | Muridae | Gerbillus sp. | 17.266783 | -16.028417 | L3 |
| http://n2t.net/ark:/65665/345ab8b59-602e-4c1b-a348-8d562c5e7080 | Rodentia | Muridae | Gerbillus sp. | 17.266783 | -16.028417 | L3 |
| http://n2t.net/ark:/65665/32443cf26-2835-4f75-92d3-eaec441f4d3d | Rodentia | Muridae | Gerbillus sp. | 17.266783 | -16.028417 | L3 |
| http://n2t.net/ark:/65665/3803268de-844c-4e27-8709-e91b63080607 | Rodentia | Muridae | Gerbillus sp. | 17.266783 | -16.028417 | L3 |
| http://n2t.net/ark:/65665/356031c44-baee-4608-b23e-e0132824b173 | Rodentia | Muridae | Gerbillus sp. | 17.266783 | -16.028417 | L3 |
| http://n2t.net/ark:/65665/3fd560f38-9da4-4fd9-ac47-96a14e89d44c | Rodentia | Muridae | Gerbillus sp. | 17.266783 | -16.028417 | L3 |
| http://n2t.net/ark:/65665/35c997dee-4523-404b-8e5f-b47af11750c7 | Rodentia | Muridae | Gerbillus sp. | 17.266783 | -16.028417 | L3 |
| http://n2t.net/ark:/65665/33424a78b-dcdf-4551-ae22-304a91134b86 | Rodentia | Muridae | Gerbillus sp. | 17.266783 | -16.028417 | L3 |
| http://n2t.net/ark:/65665/394591096-53bf-4160-b8af-71c60e30cb2a | Rodentia | Muridae | Gerbillus sp. | 17.266783 | -16.028417 | L3 |
| http://n2t.net/ark:/65665/323f5a2c7-56ff-4808-ad99-6f409771fe94 | Rodentia | Muridae | Gerbillus sp. | 17.266783 | -16.028417 | L3 |
| http://n2t.net/ark:/65665/3406d8a16-9a3e-46f7-a0f4-ccab0bd1da78 | Rodentia | Muridae | Gerbillus sp. | 17.266783 | -16.028417 | L3 |
| http://n2t.net/ark:/65665/330ec1750-5fc4-41a9-99d4-650d4aaf01b0 | Rodentia | Muridae | Gerbillus sp. | 17.266783 | -16.028417 | L3 |
| http://n2t.net/ark:/65665/37450e0e6-ea38-4ad2-bc9f-7a38274e31a6 | Rodentia | Muridae | Gerbillus sp. | 17.266783 | -16.028417 | L3 |
| http://n2t.net/ark:/65665/35e73307b-a5ea-45bc-bd1d-a023dc9cffd3 | Rodentia | Muridae | Gerbillus sp. | 17.266783 | -16.028417 | L3 |
| http://n2t.net/ark:/65665/359697d3d-ae7a-41a9-9218-9afbfde7738e | Rodentia | Muridae | Gerbillus sp. | 17.266783 | -16.028417 | L3 |
| http://n2t.net/ark:/65665/39099f381-14b5-40cc-b8c4-3ea97bf23ffb | Rodentia | Muridae | Gerbillus sp. | 17.266783 | -16.028417 | L3 |
| http://n2t.net/ark:/65665/3c6368186-7cdc-443d-9968-9466663d573f | Rodentia | Muridae | Gerbillus sp. | 17.266783 | -16.028417 | L3 |
| http://n2t.net/ark:/65665/335c2e3c0-6f4b-4dd6-b273-bca04ecdd2a3 | Rodentia | Muridae | Gerbillus sp. | 17.266783 | -16.028417 | L3 |
| http://n2t.net/ark:/65665/3487bbff4-d054-4ede-b8aa-a83a159054c0 | Rodentia | Muridae | Gerbillus sp. | 17.266783 | -16.028417 | L3 |
| http://n2t.net/ark:/65665/3968c043a-0f8b-4105-9689-8e9084953f4f | Rodentia | Muridae | Gerbillus sp. | 17.266783 | -16.028417 | L3 |
| http://n2t.net/ark:/65665/3174451fb-ed5d-4027-a0a5-87256e2b9bea | Rodentia | Muridae | Gerbillus sp. | 17.266783 | -16.028417 | L3 |
| http://n2t.net/ark:/65665/346f69b49-6b13-471a-88ce-1dc729b58a10 | Rodentia | Muridae | Gerbillus sp. | 17.266783 | -16.028417 | L3 |
| http://n2t.net/ark:/65665/3c40a37f6-541f-4cb7-bf90-7dd01bebf2a9 | Rodentia | Muridae | Gerbillus sp. | 17.266783 | -16.028417 | L3 |
| http://n2t.net/ark:/65665/3d2fefd90-5d41-487e-b7fb-bb126fa5c723 | Rodentia | Muridae | Gerbillus sp. | 17.266783 | -16.028417 | L3 |
| http://n2t.net/ark:/65665/35f6dea49-e196-4953-b341-4b54065b6ee7 | Rodentia | Muridae | Gerbillus sp. | 17.266783 | -16.028417 | L3 |
| http://n2t.net/ark:/65665/35ab9a455-59cb-4cf5-aa22-43e7f1724291 | Rodentia | Muridae | Gerbillus sp. | 17.266783 | -16.028417 | L3 |
| http://n2t.net/ark:/65665/30093d48f-1caa-4f30-b335-e2d1cf28563e | Rodentia | Muridae | Gerbillus sp. | 17.266783 | -16.028417 | L3 |
| http://n2t.net/ark:/65665/36f6d7813-02a2-4ab7-9622-909a526283b7 | Rodentia | Muridae | Gerbillus sp. | 17.266783 | -16.028417 | L3 |
| http://n2t.net/ark:/65665/30e1a29fb-8ac3-416d-858d-63711a416cd9 | Rodentia | Muridae | Gerbillus sp. | 17.266783 | -16.028417 | L3 |
| http://n2t.net/ark:/65665/3e071a3a2-c1d0-4fdc-84ff-b483437ef803 | Rodentia | Muridae | Gerbillus sp. | 17.266783 | -16.028417 | L3 |
| http://n2t.net/ark:/65665/3b93fb0a8-85e8-4a40-b498-c5a99fdc9e3d | Rodentia | Muridae | Gerbillus sp. | 17.266783 | -16.028417 | L3 |
| http://n2t.net/ark:/65665/3f7042ccd-be4d-4e92-bc36-c076866a566b | Rodentia | Muridae | Gerbillus sp. | 17.266783 | -16.028417 | L3 |
| http://n2t.net/ark:/65665/309d823c1-8996-44fd-a0fd-924ce8d125c7 | Rodentia | Muridae | Gerbillus sp. | 17.266783 | -16.028417 | L3 |
| http://n2t.net/ark:/65665/311014947-d118-4976-8573-89831dcf5619 | Rodentia | Muridae | Gerbillus sp. | 17.266783 | -16.028417 | L3 |
| http://n2t.net/ark:/65665/3acbc74bc-4afc-47e6-b1c8-616f5752d312 | Rodentia | Muridae | Gerbillus sp. | 17.266783 | -16.028417 | L3 |
| http://n2t.net/ark:/65665/38de8541d-8782-4ae5-8817-1ba7ed4205be | Rodentia | Muridae | Gerbillus sp. | 17.266783 | -16.028417 | L3 |
| http://n2t.net/ark:/65665/3777871c8-1348-4da6-a110-258a4fd7beab | Rodentia | Muridae | Gerbillus sp. | 17.266783 | -16.028417 | L3 |
| http://n2t.net/ark:/65665/3fef8c82f-dd0e-4e5a-890f-8b79ea04c8e1 | Rodentia | Muridae | Gerbillus sp. | 17.266783 | -16.028417 | L3 |
| http://n2t.net/ark:/65665/39fa4fca3-45cd-426a-8c7b-42ef02127e4c | Rodentia | Muridae | Gerbillus sp. | 17.266783 | -16.028417 | L3 |
| http://n2t.net/ark:/65665/3212a61b1-8f0d-451b-9fb7-0c27a7540dc0 | Rodentia | Muridae | Gerbillus sp. | 17.266783 | -16.028417 | L3 |
| http://n2t.net/ark:/65665/33cac987f-4b56-4c46-bbe1-35180b159429 | Rodentia | Muridae | Gerbillus sp. | 17.266783 | -16.028417 | L3 |
| http://n2t.net/ark:/65665/325315e52-4c40-4b27-acb3-9d9f57492198 | Rodentia | Muridae | Gerbillus sp. | 17.266783 | -16.028417 | L3 |
| http://n2t.net/ark:/65665/3ed45a5a7-adea-45d9-8410-7d09e2891343 | Rodentia | Muridae | Gerbillus sp. | 17.266783 | -16.028417 | L3 |
| http://n2t.net/ark:/65665/36fae9648-0f0c-44e4-920d-9e27dd9621ed | Rodentia | Muridae | Gerbillus sp. | 17.266783 | -16.028417 | L3 |
| http://n2t.net/ark:/65665/362a8e799-273f-493e-a5e7-8254decead39 | Rodentia | Muridae | Gerbillus sp. | 17.266783 | -16.028417 | L3 |
| http://n2t.net/ark:/65665/3d8200884-fc3d-4505-ab96-204a0928a982 | Rodentia | Muridae | Gerbillus sp. | 17.266783 | -16.028417 | L3 |
| http://n2t.net/ark:/65665/3b6bb3680-359d-4315-8815-ca7a01a08109 | Rodentia | Muridae | Gerbillus sp. | 17.266783 | -16.028417 | L3 |
| http://n2t.net/ark:/65665/3a820de1d-9e8b-49d1-8a79-9ec6ffc4776c | Rodentia | Muridae | Gerbillus sp. | 17.266783 | -16.028417 | L3 |
| http://n2t.net/ark:/65665/376131e0a-5276-4fea-bb32-79ef960571dd | Rodentia | Muridae | Gerbillus sp. | 17.266783 | -16.028417 | L3 |
| http://n2t.net/ark:/65665/3d2e60c1e-2a21-4985-aca8-b690c8ece35d | Rodentia | Muridae | Gerbillus sp. | 17.266783 | -16.028417 | L3 |
| http://n2t.net/ark:/65665/393b34582-a52f-4360-af0c-72ce6329d9c1 | Rodentia | Muridae | Gerbillus sp. | 17.266783 | -16.028417 | L3 |
| http://n2t.net/ark:/65665/33fd8ecfc-41c6-4fb5-a80e-28075bffcbfe | Rodentia | Muridae | Gerbillus sp. | 17.266783 | -16.028417 | L3 |
| http://n2t.net/ark:/65665/3a1a37b3d-b30b-4e8d-a4fe-976ac42ed31d | Rodentia | Muridae | Gerbillus sp. | 17.266783 | -16.028417 | L3 |
| http://n2t.net/ark:/65665/3dfcc95a8-3f49-43bc-b184-ab87c570ae94 | Rodentia | Muridae | Gerbillus sp. | 17.266783 | -16.028417 | L3 |
| http://n2t.net/ark:/65665/3722fbbf5-14f3-41db-be5c-9262624ba04c | Rodentia | Muridae | Gerbillus sp. | 17.266783 | -16.028417 | L3 |
| http://n2t.net/ark:/65665/3a957fc63-72ce-4e15-8222-46b604b179af | Rodentia | Muridae | Gerbillus sp. | 17.266783 | -16.028417 | L3 |
| http://n2t.net/ark:/65665/3ced24e50-8b6f-4863-9ffc-f5a7f93610b8 | Rodentia | Muridae | Gerbillus sp. | 17.266783 | -16.028417 | L3 |
| http://n2t.net/ark:/65665/3fa0c3cb2-42c9-4360-b46b-348a33511b34 | Rodentia | Muridae | Gerbillus sp. | 17.266783 | -16.028417 | L3 |
| http://n2t.net/ark:/65665/36584075d-0e5d-4e9c-a663-67fda7f186bb | Rodentia | Muridae | Gerbillus sp. | 17.266783 | -16.028417 | L3 |
| http://n2t.net/ark:/65665/3e5a41e67-c585-477e-b1f8-4a167eabc749 | Rodentia | Muridae | Gerbillus sp. | 17.266783 | -16.028417 | L3 |
| http://n2t.net/ark:/65665/32c9b3aa2-5a41-47fb-a6c8-19969a21d2ee | Rodentia | Muridae | Gerbillus sp. | 17.266783 | -16.028417 | L3 |
| http://n2t.net/ark:/65665/3f180327e-9bb1-4d4f-942f-da9350d2fa37 | Rodentia | Muridae | Gerbillus sp. | 17.266783 | -16.028417 | L3 |
| http://n2t.net/ark:/65665/326f76903-a643-4ce3-af6b-80cb0a9c714a | Rodentia | Muridae | Gerbillus sp. | 17.266783 | -16.028417 | L3 |
| http://n2t.net/ark:/65665/3ef1f1a1a-58f8-4648-8e1b-9e1bfe75cc35 | Rodentia | Muridae | Gerbillus sp. | 17.266783 | -16.028417 | L3 |
| http://n2t.net/ark:/65665/3c4da1417-a69f-46ea-85b1-417df6100c06 | Rodentia | Muridae | Gerbillus sp. | 17.266783 | -16.028417 | L3 |
| http://n2t.net/ark:/65665/35a427949-ccc4-452a-9dce-5ce34dc422f6 | Rodentia | Muridae | Gerbillus sp. | 17.266783 | -16.028417 | L3 |
| http://n2t.net/ark:/65665/3a2ab3cc1-7913-4650-a0d3-d5100e3e05c3 | Rodentia | Muridae | Gerbillus sp. | 17.266783 | -16.028417 | L3 |
| http://n2t.net/ark:/65665/3bc304c18-daf1-44ea-a3cc-d05c87174c65 | Rodentia | Muridae | Gerbillus sp. | 17.266783 | -16.028417 | L3 |
| http://n2t.net/ark:/65665/375c6a341-bab4-4bd7-94e6-6ef9d0f91a4f | Rodentia | Muridae | Gerbillus sp. | 17.266783 | -16.028417 | L3 |
| http://n2t.net/ark:/65665/3624222db-5577-4409-8eca-b1f305846053 | Rodentia | Muridae | Gerbillus sp. | 17.266783 | -16.028417 | L3 |
| http://n2t.net/ark:/65665/371073776-ba3c-4dc9-a199-6fc2e8930768 | Rodentia | Muridae | Gerbillus sp. | 17.266783 | -16.028417 | L3 |
| http://coldb.mnhn.fr/catalognumber/mnhn/zm/mo-2001-61 | Rodentia | Muridae | Gerbillus sp. | 17.266783 | -16.028417 | L3 |
| http://coldb.mnhn.fr/catalognumber/mnhn/zm/mo-2001-60 | Rodentia | Muridae | Gerbillus sp. | 17.266783 | -16.028417 | L3 |
| http://coldb.mnhn.fr/catalognumber/mnhn/zm/mo-2001-59 | Rodentia | Muridae | Gerbillus sp. | 17.266783 | -16.028417 | L3 |
| http://coldb.mnhn.fr/catalognumber/mnhn/zm/mo-2001-58 | Rodentia | Muridae | Gerbillus sp. | 17.266783 | -16.028417 | L3 |
| http://coldb.mnhn.fr/catalognumber/mnhn/zm/mo-2001-682 | Rodentia | Muridae | Gerbillus sp. | 19.705000 | -16.390000 | I2 |
| http://coldb.mnhn.fr/catalognumber/mnhn/zm/mo-2001-681 | Rodentia | Muridae | Gerbillus sp. | 19.705000 | -16.390000 | I2 |
| http://coldb.mnhn.fr/catalognumber/mnhn/zm/mo-2001-680 | Rodentia | Muridae | Gerbillus sp. | 19.705000 | -16.390000 | I2 |
| http://coldb.mnhn.fr/catalognumber/mnhn/zm/mo-2001-679 | Rodentia | Muridae | Gerbillus sp. | 19.705000 | -16.390000 | I2 |
| http://coldb.mnhn.fr/catalognumber/mnhn/zm/mo-2001-678 | Rodentia | Muridae | Gerbillus sp. | 19.705000 | -16.390000 | I2 |
| http://coldb.mnhn.fr/catalognumber/mnhn/zm/mo-2001-677 | Rodentia | Muridae | Gerbillus sp. | 19.705000 | -16.390000 | I2 |
| http://coldb.mnhn.fr/catalognumber/mnhn/zm/mo-2001-142 | Rodentia | Muridae | Gerbillus sp. | 18.450000 | -9.500000 | J9 |
| http://coldb.mnhn.fr/catalognumber/mnhn/zm/mo-2001-141 | Rodentia | Muridae | Gerbillus sp. | 18.450000 | -9.500000 | J9 |
| http://coldb.mnhn.fr/catalognumber/mnhn/zm/mo-2001-140 | Rodentia | Muridae | Gerbillus sp. | 18.450000 | -9.500000 | J9 |
| http://coldb.mnhn.fr/catalognumber/mnhn/zm/mo-2001-139 | Rodentia | Muridae | Gerbillus sp. | 18.450000 | -9.500000 | J9 |
| http://coldb.mnhn.fr/catalognumber/mnhn/zm/mo-2001-187 | Rodentia | Muridae | Gerbillus sp. | 19.933000 | -16.250000 | I2 |
| http://coldb.mnhn.fr/catalognumber/mnhn/zm/mo-2001-294 | Rodentia | Muridae | Gerbillus sp. | 19.536000 | -16.375000 | I2 |
| http://coldb.mnhn.fr/catalognumber/mnhn/zm/mo-2001-293 | Rodentia | Muridae | Gerbillus sp. | 19.536000 | -16.375000 | I2 |
| http://coldb.mnhn.fr/catalognumber/mnhn/zm/mo-2001-292 | Rodentia | Muridae | Gerbillus sp. | 19.536000 | -16.375000 | I2 |
| http://coldb.mnhn.fr/catalognumber/mnhn/zm/mo-2001-291 | Rodentia | Muridae | Gerbillus sp. | 19.536000 | -16.375000 | I2 |
| http://coldb.mnhn.fr/catalognumber/mnhn/zm/mo-2001-290 | Rodentia | Muridae | Gerbillus sp. | 19.536000 | -16.375000 | I2 |
| http://coldb.mnhn.fr/catalognumber/mnhn/zm/mo-2001-289 | Rodentia | Muridae | Gerbillus sp. | 19.536000 | -16.375000 | I2 |
| http://coldb.mnhn.fr/catalognumber/mnhn/zm/mo-2001-288 | Rodentia | Muridae | Gerbillus sp. | 18.836000 | -16.133100 | J2 |
| http://coldb.mnhn.fr/catalognumber/mnhn/zm/mo-2001-287 | Rodentia | Muridae | Gerbillus sp. | 18.836000 | -16.133100 | J2 |
| http://coldb.mnhn.fr/catalognumber/mnhn/zm/mo-2001-286 | Rodentia | Muridae | Gerbillus sp. | 18.836000 | -16.133100 | J2 |
| http://coldb.mnhn.fr/catalognumber/mnhn/zm/mo-2001-664 | Rodentia | Muridae | Gerbillus sp. | 18.778000 | -16.132500 | J2 |
| http://coldb.mnhn.fr/catalognumber/mnhn/zm/mo-2001-90 | Rodentia | Muridae | Gerbillus sp. | 18.778000 | -16.132500 | J2 |
| http://coldb.mnhn.fr/catalognumber/mnhn/zm/mo-2001-663 | Rodentia | Muridae | Gerbillus sp. | 18.767000 | -16.128600 | J2 |
| http://coldb.mnhn.fr/catalognumber/mnhn/zm/mo-2001-1677 | Rodentia | Muridae | Gerbillus sp. | 18.767000 | -16.128600 | J2 |
| http://coldb.mnhn.fr/catalognumber/mnhn/zm/mo-2001-671 | Rodentia | Muridae | Gerbillus sp. | 18.714000 | -16.131900 | J2 |
| http://coldb.mnhn.fr/catalognumber/mnhn/zm/mo-2001-89 | Rodentia | Muridae | Gerbillus sp. | 18.714000 | -16.131900 | J2 |
| http://coldb.mnhn.fr/catalognumber/mnhn/zm/mo-2001-180 | Rodentia | Muridae | Gerbillus sp. | 18.650000 | -16.113300 | J3 |
| http://coldb.mnhn.fr/catalognumber/mnhn/zm/mo-2001-179 | Rodentia | Muridae | Gerbillus sp. | 18.650000 | -16.113300 | J3 |
| http://coldb.mnhn.fr/catalognumber/mnhn/zm/mo-2001-178 | Rodentia | Muridae | Gerbillus sp. | 18.650000 | -16.113300 | J3 |
| http://coldb.mnhn.fr/catalognumber/mnhn/zm/mo-2001-177 | Rodentia | Muridae | Gerbillus sp. | 18.650000 | -16.113300 | J3 |
| http://coldb.mnhn.fr/catalognumber/mnhn/zm/mo-2001-176 | Rodentia | Muridae | Gerbillus sp. | 18.650000 | -16.113300 | J3 |
| http://coldb.mnhn.fr/catalognumber/mnhn/zm/mo-2001-175 | Rodentia | Muridae | Gerbillus sp. | 18.650000 | -16.113300 | J3 |
| http://coldb.mnhn.fr/catalognumber/mnhn/zm/mo-2001-174 | Rodentia | Muridae | Gerbillus sp. | 18.650000 | -16.113300 | J3 |
| http://coldb.mnhn.fr/catalognumber/mnhn/zm/mo-2001-622 | Rodentia | Muridae | Gerbillus sp. | 18.642000 | -16.106000 | J3 |
| http://coldb.mnhn.fr/catalognumber/mnhn/zm/mo-2001-621 | Rodentia | Muridae | Gerbillus sp. | 18.642000 | -16.106000 | J3 |
| http://coldb.mnhn.fr/catalognumber/mnhn/zm/mo-2001-620 | Rodentia | Muridae | Gerbillus sp. | 18.642000 | -16.106000 | J3 |
| http://coldb.mnhn.fr/catalognumber/mnhn/zm/mo-2001-619 | Rodentia | Muridae | Gerbillus sp. | 18.642000 | -16.106000 | J3 |
| http://coldb.mnhn.fr/catalognumber/mnhn/zm/mo-2001-618 | Rodentia | Muridae | Gerbillus sp. | 18.642000 | -16.106000 | J3 |
| http://coldb.mnhn.fr/catalognumber/mnhn/zm/mo-2001-608 | Rodentia | Muridae | Gerbillus sp. | 18.642000 | -16.106000 | J3 |
| http://coldb.mnhn.fr/catalognumber/mnhn/zm/mo-2001-403 | Rodentia | Muridae | Gerbillus sp. | 18.642000 | -16.106000 | J3 |
| http://coldb.mnhn.fr/catalognumber/mnhn/zm/mo-2001-402 | Rodentia | Muridae | Gerbillus sp. | 18.642000 | -16.106000 | J3 |
| http://coldb.mnhn.fr/catalognumber/mnhn/zm/mo-2001-401 | Rodentia | Muridae | Gerbillus sp. | 18.642000 | -16.106000 | J3 |
| http://coldb.mnhn.fr/catalognumber/mnhn/zm/mo-2001-400 | Rodentia | Muridae | Gerbillus sp. | 18.642000 | -16.106000 | J3 |
| http://coldb.mnhn.fr/catalognumber/mnhn/zm/mo-2001-399 | Rodentia | Muridae | Gerbillus sp. | 18.642000 | -16.106000 | J3 |
| http://coldb.mnhn.fr/catalognumber/mnhn/zm/mo-2001-398 | Rodentia | Muridae | Gerbillus sp. | 18.642000 | -16.106000 | J3 |
| http://coldb.mnhn.fr/catalognumber/mnhn/zm/mo-2001-397 | Rodentia | Muridae | Gerbillus sp. | 18.642000 | -16.106000 | J3 |
| http://coldb.mnhn.fr/catalognumber/mnhn/zm/mo-2001-395 | Rodentia | Muridae | Gerbillus sp. | 18.642000 | -16.106000 | J3 |
| http://coldb.mnhn.fr/catalognumber/mnhn/zm/mo-2001-394 | Rodentia | Muridae | Gerbillus sp. | 18.642000 | -16.106000 | J3 |
| http://coldb.mnhn.fr/catalognumber/mnhn/zm/mo-2001-64 | Rodentia | Muridae | Gerbillus sp. | 18.642000 | -16.106000 | J3 |
| http://coldb.mnhn.fr/catalognumber/mnhn/zm/mo-2001-63 | Rodentia | Muridae | Gerbillus sp. | 18.642000 | -16.106000 | J3 |
| http://coldb.mnhn.fr/catalognumber/mnhn/zm/mo-2001-62 | Rodentia | Muridae | Gerbillus sp. | 18.642000 | -16.106000 | J3 |
| http://coldb.mnhn.fr/catalognumber/mnhn/zm/mo-2001-50 | Rodentia | Muridae | Gerbillus sp. | 18.642000 | -16.106000 | J3 |
| http://coldb.mnhn.fr/catalognumber/mnhn/zm/mo-2001-173 | Rodentia | Muridae | Gerbillus sp. | 18.633000 | -16.113300 | J3 |
| http://coldb.mnhn.fr/catalognumber/mnhn/zm/mo-2001-172 | Rodentia | Muridae | Gerbillus sp. | 18.633000 | -16.113300 | J3 |
| http://coldb.mnhn.fr/catalognumber/mnhn/zm/mo-2001-171 | Rodentia | Muridae | Gerbillus sp. | 18.633000 | -16.113300 | J3 |
| http://coldb.mnhn.fr/catalognumber/mnhn/zm/mo-2001-1687 | Rodentia | Muridae | Gerbillus sp. | 18.625000 | -16.106000 | J3 |
| http://coldb.mnhn.fr/catalognumber/mnhn/zm/mo-2001-1686 | Rodentia | Muridae | Gerbillus sp. | 18.625000 | -16.106000 | J3 |
| http://coldb.mnhn.fr/catalognumber/mnhn/zm/mo-2001-1685 | Rodentia | Muridae | Gerbillus sp. | 18.625000 | -16.106000 | J3 |
| http://coldb.mnhn.fr/catalognumber/mnhn/zm/mo-2001-368 | Rodentia | Muridae | Gerbillus sp. | 17.636000 | -16.011100 | K3 |
| http://coldb.mnhn.fr/catalognumber/mnhn/zm/mo-2001-367 | Rodentia | Muridae | Gerbillus sp. | 17.636000 | -16.011100 | K3 |
| http://coldb.mnhn.fr/catalognumber/mnhn/zm/mo-2001-366 | Rodentia | Muridae | Gerbillus sp. | 17.636000 | -16.011100 | K3 |
| http://coldb.mnhn.fr/catalognumber/mnhn/zm/mo-2001-365 | Rodentia | Muridae | Gerbillus sp. | 17.636000 | -16.011100 | K3 |
| http://coldb.mnhn.fr/catalognumber/mnhn/zm/mo-2001-363 | Rodentia | Muridae | Gerbillus sp. | 17.636000 | -16.011100 | K3 |
| http://coldb.mnhn.fr/catalognumber/mnhn/zm/mo-2001-362 | Rodentia | Muridae | Gerbillus sp. | 17.636000 | -16.011100 | K3 |
| http://coldb.mnhn.fr/catalognumber/mnhn/zm/mo-2001-361 | Rodentia | Muridae | Gerbillus sp. | 17.636000 | -16.011100 | K3 |
| http://coldb.mnhn.fr/catalognumber/mnhn/zm/mo-2001-268 | Rodentia | Muridae | Gerbillus sp. | 17.636000 | -16.011100 | K3 |
| http://coldb.mnhn.fr/catalognumber/mnhn/zm/mo-2001-267 | Rodentia | Muridae | Gerbillus sp. | 17.636000 | -16.011100 | K3 |
| http://coldb.mnhn.fr/catalognumber/mnhn/zm/mo-2001-266 | Rodentia | Muridae | Gerbillus sp. | 17.636000 | -16.011100 | K3 |
| http://coldb.mnhn.fr/catalognumber/mnhn/zm/mo-2001-265 | Rodentia | Muridae | Gerbillus sp. | 17.636000 | -16.011100 | K3 |
| http://coldb.mnhn.fr/catalognumber/mnhn/zm/mo-2001-264 | Rodentia | Muridae | Gerbillus sp. | 17.636000 | -16.011100 | K3 |
| http://coldb.mnhn.fr/catalognumber/mnhn/zm/mo-2001-263 | Rodentia | Muridae | Gerbillus sp. | 17.636000 | -16.011100 | K3 |
| http://coldb.mnhn.fr/catalognumber/mnhn/zm/mo-2001-262 | Rodentia | Muridae | Gerbillus sp. | 17.636000 | -16.011100 | K3 |
| http://coldb.mnhn.fr/catalognumber/mnhn/zm/mo-2001-261 | Rodentia | Muridae | Gerbillus sp. | 17.636000 | -16.011100 | K3 |
| http://coldb.mnhn.fr/catalognumber/mnhn/zm/mo-2001-260 | Rodentia | Muridae | Gerbillus sp. | 17.636000 | -16.011100 | K3 |
| http://coldb.mnhn.fr/catalognumber/mnhn/zm/mo-2001-259 | Rodentia | Muridae | Gerbillus sp. | 17.636000 | -16.011100 | K3 |
| http://coldb.mnhn.fr/catalognumber/mnhn/zm/mo-2001-258 | Rodentia | Muridae | Gerbillus sp. | 17.636000 | -16.011100 | K3 |
| http://coldb.mnhn.fr/catalognumber/mnhn/zm/mo-2001-257 | Rodentia | Muridae | Gerbillus sp. | 17.636000 | -16.011100 | K3 |
| http://coldb.mnhn.fr/catalognumber/mnhn/zm/mo-2001-256 | Rodentia | Muridae | Gerbillus sp. | 17.636000 | -16.011100 | K3 |
| http://coldb.mnhn.fr/catalognumber/mnhn/zm/mo-2001-458 | Rodentia | Muridae | Gerbillus sp. | 17.633000 | -16.028600 | K3 |
| http://coldb.mnhn.fr/catalognumber/mnhn/zm/mo-2001-457 | Rodentia | Muridae | Gerbillus sp. | 17.633000 | -16.028600 | K3 |
| http://coldb.mnhn.fr/catalognumber/mnhn/zm/mo-2001-456 | Rodentia | Muridae | Gerbillus sp. | 17.633000 | -16.028600 | K3 |
| http://coldb.mnhn.fr/catalognumber/mnhn/zm/mo-2001-455 | Rodentia | Muridae | Gerbillus sp. | 17.633000 | -16.028600 | K3 |
| http://coldb.mnhn.fr/catalognumber/mnhn/zm/mo-2001-454 | Rodentia | Muridae | Gerbillus sp. | 17.633000 | -16.028600 | K3 |
| http://coldb.mnhn.fr/catalognumber/mnhn/zm/mo-2001-453 | Rodentia | Muridae | Gerbillus sp. | 17.633000 | -16.028600 | K3 |
| http://coldb.mnhn.fr/catalognumber/mnhn/zm/mo-2001-452 | Rodentia | Muridae | Gerbillus sp. | 17.633000 | -16.028600 | K3 |
| http://coldb.mnhn.fr/catalognumber/mnhn/zm/mo-2001-451 | Rodentia | Muridae | Gerbillus sp. | 17.633000 | -16.028600 | K3 |
| http://coldb.mnhn.fr/catalognumber/mnhn/zm/mo-2001-450 | Rodentia | Muridae | Gerbillus sp. | 17.633000 | -16.028600 | K3 |
| http://coldb.mnhn.fr/catalognumber/mnhn/zm/mo-2001-449 | Rodentia | Muridae | Gerbillus sp. | 17.633000 | -16.028600 | K3 |
| http://coldb.mnhn.fr/catalognumber/mnhn/zm/mo-2001-448 | Rodentia | Muridae | Gerbillus sp. | 17.633000 | -16.028600 | K3 |
| http://coldb.mnhn.fr/catalognumber/mnhn/zm/mo-2001-447 | Rodentia | Muridae | Gerbillus sp. | 17.633000 | -16.028600 | K3 |
| http://coldb.mnhn.fr/catalognumber/mnhn/zm/mo-2001-467 | Rodentia | Muridae | Gerbillus sp. | 17.631000 | -16.024200 | K3 |
| http://coldb.mnhn.fr/catalognumber/mnhn/zm/mo-2001-466 | Rodentia | Muridae | Gerbillus sp. | 17.631000 | -16.024200 | K3 |
| http://coldb.mnhn.fr/catalognumber/mnhn/zm/mo-2001-465 | Rodentia | Muridae | Gerbillus sp. | 17.631000 | -16.024200 | K3 |
| http://coldb.mnhn.fr/catalognumber/mnhn/zm/mo-2001-464 | Rodentia | Muridae | Gerbillus sp. | 17.631000 | -16.024200 | K3 |
| http://coldb.mnhn.fr/catalognumber/mnhn/zm/mo-2001-280 | Rodentia | Muridae | Gerbillus sp. | 17.600000 | -16.018600 | K3 |
| http://coldb.mnhn.fr/catalognumber/mnhn/zm/mo-2001-279 | Rodentia | Muridae | Gerbillus sp. | 17.600000 | -16.018600 | K3 |
| http://coldb.mnhn.fr/catalognumber/mnhn/zm/mo-2001-278 | Rodentia | Muridae | Gerbillus sp. | 17.600000 | -16.018600 | K3 |
| http://coldb.mnhn.fr/catalognumber/mnhn/zm/mo-2001-277 | Rodentia | Muridae | Gerbillus sp. | 17.600000 | -16.018600 | K3 |
| http://coldb.mnhn.fr/catalognumber/mnhn/zm/mo-2001-275 | Rodentia | Muridae | Gerbillus sp. | 17.600000 | -16.018600 | K3 |
| http://coldb.mnhn.fr/catalognumber/mnhn/zm/mo-2001-274 | Rodentia | Muridae | Gerbillus sp. | 17.600000 | -16.018600 | K3 |
| http://coldb.mnhn.fr/catalognumber/mnhn/zm/mo-2001-273 | Rodentia | Muridae | Gerbillus sp. | 17.600000 | -16.018600 | K3 |
| http://coldb.mnhn.fr/catalognumber/mnhn/zm/mo-2001-272 | Rodentia | Muridae | Gerbillus sp. | 17.600000 | -16.018600 | K3 |
| http://coldb.mnhn.fr/catalognumber/mnhn/zm/mo-2001-270 | Rodentia | Muridae | Gerbillus sp. | 17.600000 | -16.018600 | K3 |
| http://coldb.mnhn.fr/catalognumber/mnhn/zm/mo-2001-269 | Rodentia | Muridae | Gerbillus sp. | 17.600000 | -16.018600 | K3 |
| http://coldb.mnhn.fr/catalognumber/mnhn/zm/mo-1997-1628 | Rodentia | Muridae | Gerbillus sp. | 17.517000 | -16.033300 | K3 |
| http://coldb.mnhn.fr/catalognumber/mnhn/zm/mo-2001-1691 | Rodentia | Muridae | Gerbillus sp. | 17.494000 | -16.022500 | K3 |
| http://coldb.mnhn.fr/catalognumber/mnhn/zm/mo-2001-446 | Rodentia | Muridae | Gerbillus sp. | 17.486000 | -16.023100 | L3 |
| http://coldb.mnhn.fr/catalognumber/mnhn/zm/mo-2001-445 | Rodentia | Muridae | Gerbillus sp. | 17.486000 | -16.023100 | L3 |
| http://coldb.mnhn.fr/catalognumber/mnhn/zm/mo-2001-444 | Rodentia | Muridae | Gerbillus sp. | 17.486000 | -16.023100 | L3 |
| http://coldb.mnhn.fr/catalognumber/mnhn/zm/mo-2001-443 | Rodentia | Muridae | Gerbillus sp. | 17.486000 | -16.023100 | L3 |
| http://coldb.mnhn.fr/catalognumber/mnhn/zm/mo-2001-442 | Rodentia | Muridae | Gerbillus sp. | 17.486000 | -16.023100 | L3 |
| http://coldb.mnhn.fr/catalognumber/mnhn/zm/mo-2001-441 | Rodentia | Muridae | Gerbillus sp. | 17.486000 | -16.023100 | L3 |
| http://coldb.mnhn.fr/catalognumber/mnhn/zm/mo-2001-440 | Rodentia | Muridae | Gerbillus sp. | 17.486000 | -16.023100 | L3 |
| http://coldb.mnhn.fr/catalognumber/mnhn/zm/mo-2001-439 | Rodentia | Muridae | Gerbillus sp. | 17.486000 | -16.023100 | L3 |
| http://coldb.mnhn.fr/catalognumber/mnhn/zm/mo-2001-438 | Rodentia | Muridae | Gerbillus sp. | 17.486000 | -16.023100 | L3 |
| http://coldb.mnhn.fr/catalognumber/mnhn/zm/mo-2001-437 | Rodentia | Muridae | Gerbillus sp. | 17.486000 | -16.023100 | L3 |
| http://coldb.mnhn.fr/catalognumber/mnhn/zm/mo-2001-436 | Rodentia | Muridae | Gerbillus sp. | 17.486000 | -16.023100 | L3 |
| http://coldb.mnhn.fr/catalognumber/mnhn/zm/mo-1997-1624 | Rodentia | Muridae | Gerbillus sp. | 17.486000 | -16.023100 | L3 |
| http://coldb.mnhn.fr/catalognumber/mnhn/zm/mo-1997-1623 | Rodentia | Muridae | Gerbillus sp. | 17.486000 | -16.023100 | L3 |
| http://coldb.mnhn.fr/catalognumber/mnhn/zm/mo-2001-91 | Rodentia | Muridae | Gerbillus sp. | 17.434000 | -16.065000 | L3 |
| http://coldb.mnhn.fr/catalognumber/mnhn/zm/mo-2001-637 | Rodentia | Muridae | Gerbillus sp. | 17.417000 | -16.067000 | L3 |
| http://coldb.mnhn.fr/catalognumber/mnhn/zm/mo-2001-636 | Rodentia | Muridae | Gerbillus sp. | 17.417000 | -16.067000 | L3 |
| http://coldb.mnhn.fr/catalognumber/mnhn/zm/mo-2001-635 | Rodentia | Muridae | Gerbillus sp. | 17.417000 | -16.067000 | L3 |
| http://coldb.mnhn.fr/catalognumber/mnhn/zm/mo-2001-616 | Rodentia | Muridae | Gerbillus sp. | 17.417000 | -16.067000 | L3 |
| http://coldb.mnhn.fr/catalognumber/mnhn/zm/mo-2001-53 | Rodentia | Muridae | Gerbillus sp. | 17.417000 | -16.067000 | L3 |
| http://coldb.mnhn.fr/catalognumber/mnhn/zm/mo-2001-247 | Rodentia | Muridae | Gerbillus sp. | 20.165000 | -16.221000 | I2 |
| http://coldb.mnhn.fr/catalognumber/mnhn/zm/mo-2001-246 | Rodentia | Muridae | Gerbillus sp. | 20.165000 | -16.221000 | I2 |
| http://coldb.mnhn.fr/catalognumber/mnhn/zm/mo-2001-617 | Rodentia | Muridae | Gerbillus sp. | 17.428000 | -16.011900 | L3 |
| http://coldb.mnhn.fr/catalognumber/mnhn/zm/mo-2001-468 | Rodentia | Muridae | Gerbillus sp. | 17.428000 | -16.011900 | L3 |
| http://coldb.mnhn.fr/catalognumber/mnhn/zm/mo-2001-57 | Rodentia | Muridae | Gerbillus sp. | 17.428000 | -16.011900 | L3 |
| http://coldb.mnhn.fr/catalognumber/mnhn/zm/mo-2001-56 | Rodentia | Muridae | Gerbillus sp. | 17.428000 | -16.011900 | L3 |
| http://coldb.mnhn.fr/catalognumber/mnhn/zm/mo-2001-55 | Rodentia | Muridae | Gerbillus sp. | 17.428000 | -16.011900 | L3 |
| http://coldb.mnhn.fr/catalognumber/mnhn/zm/mo-2001-226 | Rodentia | Muridae | Gerbillus sp. | 18.703000 | -16.030000 | J3 |
| http://coldb.mnhn.fr/catalognumber/mnhn/zm/mo-2001-225 | Rodentia | Muridae | Gerbillus sp. | 18.703000 | -16.030000 | J3 |
| http://coldb.mnhn.fr/catalognumber/mnhn/zm/mo-2001-198 | Rodentia | Muridae | Gerbillus sp. | 20.124443 | -16.259393 | I2 |
| http://coldb.mnhn.fr/catalognumber/mnhn/zm/mo-2001-197 | Rodentia | Muridae | Gerbillus sp. | 20.124443 | -16.259393 | I2 |
| http://coldb.mnhn.fr/catalognumber/mnhn/zm/mo-2001-196 | Rodentia | Muridae | Gerbillus sp. | 20.124443 | -16.259393 | I2 |
| http://coldb.mnhn.fr/catalognumber/mnhn/zm/mo-2001-195 | Rodentia | Muridae | Gerbillus sp. | 20.124443 | -16.259393 | I2 |
| http://coldb.mnhn.fr/catalognumber/mnhn/zm/mo-2001-194 | Rodentia | Muridae | Gerbillus sp. | 20.124443 | -16.259393 | I2 |
| http://coldb.mnhn.fr/catalognumber/mnhn/zm/mo-2001-193 | Rodentia | Muridae | Gerbillus sp. | 20.124443 | -16.259393 | I2 |
| http://coldb.mnhn.fr/catalognumber/mnhn/zm/mo-2001-192 | Rodentia | Muridae | Gerbillus sp. | 20.124443 | -16.259393 | I2 |
| http://coldb.mnhn.fr/catalognumber/mnhn/zm/mo-2001-191 | Rodentia | Muridae | Gerbillus sp. | 20.124443 | -16.259393 | I2 |
| http://coldb.mnhn.fr/catalognumber/mnhn/zm/mo-2001-190 | Rodentia | Muridae | Gerbillus sp. | 20.124443 | -16.259393 | I2 |
| http://coldb.mnhn.fr/catalognumber/mnhn/zm/mo-2001-189 | Rodentia | Muridae | Gerbillus sp. | 20.124443 | -16.259393 | I2 |
| http://coldb.mnhn.fr/catalognumber/mnhn/zm/mo-2001-188 | Rodentia | Muridae | Gerbillus sp. | 20.124443 | -16.259393 | I2 |
| http://n2t.net/ark:/65665/341f5a10b-c91b-4276-b871-299917168f53 | Rodentia | Muridae | Gerbillus sp. | 15.933333 | -12.016667 | M7 |
| http://n2t.net/ark:/65665/34c5cf273-838b-40f3-9c84-dce11717570a | Rodentia | Muridae | Gerbillus sp. | 15.933333 | -12.016667 | M7 |
| http://n2t.net/ark:/65665/3da3526df-759a-406b-8abd-84ce93a3589b | Rodentia | Muridae | Gerbillus sp. | 15.933333 | -12.016667 | M7 |
| http://n2t.net/ark:/65665/3dd12979b-8b8d-4556-80f1-2ec1228197a8 | Rodentia | Muridae | Gerbillus sp. | 15.933333 | -12.016667 | M7 |
| http://n2t.net/ark:/65665/3218972d0-ea46-47b9-8b22-d7e11d85842f | Rodentia | Muridae | Gerbillus sp. | 15.933333 | -12.016667 | M7 |
| http://n2t.net/ark:/65665/3219b1885-c566-4874-a0a2-253f329539cc | Rodentia | Muridae | Gerbillus sp. | 15.933333 | -12.016667 | M7 |
| http://n2t.net/ark:/65665/3e51c02dc-b752-444d-a063-5e7c234c2194 | Rodentia | Muridae | Gerbillus sp. | 15.933333 | -12.016667 | M7 |
| http://n2t.net/ark:/65665/3cf183467-841b-4acb-8483-1f9d33141e17 | Rodentia | Muridae | Gerbillus sp. | 15.933333 | -12.016667 | M7 |
| http://n2t.net/ark:/65665/3eefc04ee-58e8-42bd-9862-5e9e249aa0a6 | Rodentia | Muridae | Gerbillus sp. | 15.933333 | -12.016667 | M7 |
| http://n2t.net/ark:/65665/3169e0117-43ae-4840-80a1-dd2fc85baaf0 | Rodentia | Muridae | Gerbillus sp. | 15.933333 | -12.016667 | M7 |
| http://n2t.net/ark:/65665/3c849f5e2-ff31-456d-b746-94ff53cd3251 | Rodentia | Muridae | Gerbillus sp. | 15.933333 | -12.016667 | M7 |
| http://n2t.net/ark:/65665/3575eac23-1458-4670-bbc1-feaee698b6a7 | Rodentia | Muridae | Gerbillus sp. | 15.933333 | -12.016667 | M7 |
| http://n2t.net/ark:/65665/3cb16bc08-6e3d-44ec-baee-ffa36cb4722d | Rodentia | Muridae | Gerbillus sp. | 15.933333 | -12.016667 | M7 |
| http://coldb.mnhn.fr/catalognumber/mnhn/zm/mo-2001-685 | Rodentia | Muridae | Gerbillus sp. | 17.961000 | -12.331000 | K6 |
| http://coldb.mnhn.fr/catalognumber/mnhn/zm/mo-2001-1679 | Rodentia | Muridae | Gerbillus sp. | 17.961000 | -12.331000 | K6 |
| http://coldb.mnhn.fr/catalognumber/mnhn/zm/mo-2001-1678 | Rodentia | Muridae | Gerbillus sp. | 17.961000 | -12.331000 | K6 |
| http://coldb.mnhn.fr/catalognumber/mnhn/zm/mo-2001-669 | Rodentia | Muridae | Gerbillus sp. | 19.297000 | -16.486000 | I2 |
| http://coldb.mnhn.fr/catalognumber/mnhn/zm/mo-2001-668 | Rodentia | Muridae | Gerbillus sp. | 19.297000 | -16.486000 | I2 |
| http://coldb.mnhn.fr/catalognumber/mnhn/zm/mo-2001-641 | Rodentia | Muridae | Gerbillus sp. | 19.364583 | -16.465478 | I2 |
| http://n2t.net/ark:/65665/38eced590-6212-4188-8c28-6359caae0d2a | Rodentia | Muridae | Gerbillus sp. | 18.068000 | -15.901000 | K3 |
| http://n2t.net/ark:/65665/3865d4df1-6c86-432f-a656-c22671a44eac | Rodentia | Muridae | Gerbillus sp. | 18.068000 | -15.901000 | K3 |
| http://n2t.net/ark:/65665/3e16b4943-edeb-4cbc-bc1f-c1d99d8b6f1f | Rodentia | Muridae | Gerbillus sp. | 18.068000 | -15.901000 | K3 |
| http://n2t.net/ark:/65665/38902e90d-9c60-4edb-befa-ed3e80487dc2 | Rodentia | Muridae | Gerbillus sp. | 18.068000 | -15.901000 | K3 |
| http://n2t.net/ark:/65665/3e0ca3dbe-9bba-4491-851c-ab1d9c2d588c | Rodentia | Muridae | Gerbillus sp. | 18.068000 | -15.901000 | K3 |
| http://n2t.net/ark:/65665/3cbea031c-97ca-4566-a71c-e5f52b7fd6dd | Rodentia | Muridae | Gerbillus sp. | 18.068000 | -15.901000 | K3 |
| http://n2t.net/ark:/65665/373a3c05e-7f9e-45c6-8919-e456d903b9fd | Rodentia | Muridae | Gerbillus sp. | 18.068000 | -15.901000 | K3 |
| http://n2t.net/ark:/65665/36a33d242-c758-4930-bf49-8a729d509b60 | Rodentia | Muridae | Gerbillus sp. | 18.068000 | -15.901000 | K3 |
| http://n2t.net/ark:/65665/38882e7cd-785c-41fb-9daf-934338e9d0aa | Rodentia | Muridae | Gerbillus sp. | 18.068000 | -15.901000 | K3 |
| http://n2t.net/ark:/65665/3099afedc-0339-440d-a340-2c9b1cfa96c8 | Rodentia | Muridae | Gerbillus sp. | 18.068000 | -15.901000 | K3 |
| http://n2t.net/ark:/65665/3430d06dc-1fb3-4c2b-b8fa-8c04ae4060ae | Rodentia | Muridae | Gerbillus sp. | 18.068000 | -15.901000 | K3 |
| http://n2t.net/ark:/65665/3ac204949-b853-4918-84e7-f7dbc4067302 | Rodentia | Muridae | Gerbillus sp. | 18.068000 | -15.901000 | K3 |
| http://n2t.net/ark:/65665/3120e4f15-99c4-4661-8474-c78e7bd8f140 | Rodentia | Muridae | Gerbillus sp. | 18.068000 | -15.901000 | K3 |
| http://n2t.net/ark:/65665/37ad03525-0971-43f5-91db-1bcfd0d5b6f1 | Rodentia | Muridae | Gerbillus sp. | 18.068000 | -15.901000 | K3 |
| http://n2t.net/ark:/65665/39ec1192c-9aa5-4cc6-8dbd-e8c264576259 | Rodentia | Muridae | Gerbillus sp. | 18.068000 | -15.901000 | K3 |
| http://n2t.net/ark:/65665/30242f6e9-c41f-4e8c-b5f5-773987fd88b0 | Rodentia | Muridae | Gerbillus sp. | 18.068000 | -15.901000 | K3 |
| http://n2t.net/ark:/65665/312213fa6-18da-4584-95d4-156eb4e03ba0 | Rodentia | Muridae | Gerbillus sp. | 18.068000 | -15.901000 | K3 |
| http://n2t.net/ark:/65665/3f02754b4-ba35-4dbc-819a-0671cc3b68f7 | Rodentia | Muridae | Gerbillus sp. | 18.068000 | -15.901000 | K3 |
| http://n2t.net/ark:/65665/38f0c6749-fef7-4f05-9c71-9964e0ca92ef | Rodentia | Muridae | Gerbillus sp. | 17.409000 | -16.061000 | L3 |
| http://n2t.net/ark:/65665/3b9c2f920-d66f-4976-9de4-33814420a0fd | Rodentia | Muridae | Gerbillus sp. | 18.103000 | -16.025000 | K3 |
| http://n2t.net/ark:/65665/338959e39-6958-43a5-a26d-910389944238 | Rodentia | Muridae | Gerbillus sp. | 18.103000 | -16.025000 | K3 |
| http://n2t.net/ark:/65665/3027c2983-debd-411d-9ee4-cd75e1363fe0 | Rodentia | Muridae | Gerbillus sp. | 18.103000 | -16.025000 | K3 |
| http://n2t.net/ark:/65665/303743a14-5477-4bc4-8e55-61a7c4a8d4dd | Rodentia | Muridae | Gerbillus sp. | 18.103000 | -16.025000 | K3 |
| http://n2t.net/ark:/65665/3dcd28c40-958b-4342-be72-e8d179b816fd | Rodentia | Muridae | Gerbillus sp. | 18.103000 | -16.025000 | K3 |
| http://n2t.net/ark:/65665/36b050536-9dda-4854-91a1-f915472c799d | Rodentia | Muridae | Gerbillus sp. | 18.103000 | -16.025000 | K3 |
| http://n2t.net/ark:/65665/36e7acd92-e2e2-499b-b702-c38047509556 | Rodentia | Muridae | Gerbillus sp. | 18.103000 | -16.025000 | K3 |
| http://n2t.net/ark:/65665/3a5c527e6-f6c3-4df9-b33a-cd18ee59627e | Rodentia | Muridae | Gerbillus sp. | 18.103000 | -16.025000 | K3 |
| http://n2t.net/ark:/65665/3ee2ae3ba-9bdb-4bf4-82b5-68f4c54cd7b0 | Rodentia | Muridae | Gerbillus sp. | 18.103000 | -16.025000 | K3 |
| http://n2t.net/ark:/65665/3337bb7f8-a236-481d-8d13-9f58f5d59d65 | Rodentia | Muridae | Gerbillus sp. | 18.103000 | -16.025000 | K3 |
| http://n2t.net/ark:/65665/3a6698f5a-d385-42f0-9a53-7e2db59a0063 | Rodentia | Muridae | Gerbillus sp. | 18.103000 | -16.025000 | K3 |
| http://n2t.net/ark:/65665/3bed3675f-2592-4ec6-804b-d03545bb9523 | Rodentia | Muridae | Gerbillus sp. | 18.103000 | -16.025000 | K3 |
| http://n2t.net/ark:/65665/3f07c0035-9bec-490b-9344-6b4c93185f02 | Rodentia | Muridae | Gerbillus sp. | 18.103000 | -16.025000 | K3 |
| http://n2t.net/ark:/65665/3c5109a77-2545-4e11-9c53-67fd1e0468d0 | Rodentia | Muridae | Gerbillus sp. | 18.103000 | -16.025000 | K3 |
| http://n2t.net/ark:/65665/364d8aff8-1616-484c-9dc6-f18f0e5ab817 | Rodentia | Muridae | Gerbillus sp. | 18.103000 | -16.025000 | K3 |
| http://n2t.net/ark:/65665/371c3c1b5-fbf7-436e-be18-7e92d709b016 | Rodentia | Muridae | Gerbillus sp. | 18.103000 | -16.025000 | K3 |
| http://n2t.net/ark:/65665/3b8c153b8-438e-4038-ad6f-b1d523a0693f | Rodentia | Muridae | Gerbillus sp. | 18.103000 | -16.025000 | K3 |
| http://n2t.net/ark:/65665/385d29d92-199d-42cf-8602-dc0f7fad1d81 | Rodentia | Muridae | Gerbillus sp. | 18.015000 | -15.910000 | K3 |
| http://n2t.net/ark:/65665/3fb644429-968c-4c5b-9bd7-c9ae8d1e8c85 | Rodentia | Muridae | Gerbillus sp. | 18.015000 | -15.910000 | K3 |
| http://n2t.net/ark:/65665/3d2dcb3b6-62f1-4a6a-9228-593930ef047f | Rodentia | Muridae | Gerbillus sp. | 18.015000 | -15.910000 | K3 |
| http://n2t.net/ark:/65665/36f147a64-c076-458d-8bea-8c8c0556a773 | Rodentia | Muridae | Gerbillus sp. | 18.015000 | -15.910000 | K3 |
| http://n2t.net/ark:/65665/36c3151f6-b491-4245-83f7-afe2419a41ef | Rodentia | Muridae | Gerbillus sp. | 18.015000 | -15.910000 | K3 |
| http://n2t.net/ark:/65665/3ae33ee83-ef3d-4ee3-8b93-c51c6f9d799e | Rodentia | Muridae | Gerbillus sp. | 18.015000 | -15.910000 | K3 |
| http://n2t.net/ark:/65665/324051a9b-8de3-47bc-a45b-872f1a8db217 | Rodentia | Muridae | Gerbillus sp. | 18.015000 | -15.910000 | K3 |
| http://n2t.net/ark:/65665/3d18dcd06-db2c-4624-b7f2-8eee208b7409 | Rodentia | Muridae | Gerbillus sp. | 18.015000 | -15.910000 | K3 |
| http://n2t.net/ark:/65665/37c1fafbd-f1a0-441f-9a09-5e0f5024abdd | Rodentia | Muridae | Gerbillus sp. | 18.015000 | -15.910000 | K3 |
| http://n2t.net/ark:/65665/3ba502462-6b26-45c6-ab1b-4b3417bc6439 | Rodentia | Muridae | Gerbillus sp. | 18.015000 | -15.910000 | K3 |
| http://n2t.net/ark:/65665/31e1fa448-10bd-4c5d-be98-69c5173baeae | Rodentia | Muridae | Gerbillus sp. | 18.015000 | -15.910000 | K3 |
| http://n2t.net/ark:/65665/31e0ba7f4-a5be-45a7-9431-460cc83cf385 | Rodentia | Muridae | Gerbillus sp. | 18.015000 | -15.910000 | K3 |
| http://n2t.net/ark:/65665/304dfe868-5570-4319-aff2-0496f0ae325a | Rodentia | Muridae | Gerbillus sp. | 18.015000 | -15.910000 | K3 |
| http://n2t.net/ark:/65665/3aeaacdff-2e0f-40b9-a3c5-bb5af74939c5 | Rodentia | Muridae | Gerbillus sp. | 18.015000 | -15.910000 | K3 |
| http://n2t.net/ark:/65665/3912b76f5-0515-4126-86ab-62952024edfe | Rodentia | Muridae | Gerbillus sp. | 18.015000 | -15.910000 | K3 |
| http://n2t.net/ark:/65665/3f926b91f-ee32-44b0-af27-b8f061893eba | Rodentia | Muridae | Gerbillus sp. | 18.015000 | -15.910000 | K3 |
| http://n2t.net/ark:/65665/30eff1f40-296a-431f-bb6a-96cd39503f62 | Rodentia | Muridae | Gerbillus sp. | 18.015000 | -15.910000 | K3 |
| http://n2t.net/ark:/65665/32a2ba030-0e5d-4296-9fa2-6b0b270bd9e1 | Rodentia | Muridae | Gerbillus sp. | 18.015000 | -15.910000 | K3 |
| http://n2t.net/ark:/65665/31f5937a7-3f3c-4902-bfee-7aecaa2ba59a | Rodentia | Muridae | Gerbillus sp. | 18.015000 | -15.910000 | K3 |
| http://n2t.net/ark:/65665/3736546d3-681e-4703-aeec-721a48d328f9 | Rodentia | Muridae | Gerbillus sp. | 18.015000 | -15.910000 | K3 |
| http://n2t.net/ark:/65665/31c076f44-8ff8-44df-a067-db9913264016 | Rodentia | Muridae | Gerbillus sp. | 18.015000 | -15.910000 | K3 |
| http://n2t.net/ark:/65665/3795d4ed8-74bc-488c-895f-bf21a4de7749 | Rodentia | Muridae | Gerbillus sp. | 18.015000 | -15.910000 | K3 |
| http://n2t.net/ark:/65665/30b28f40a-b6ad-4769-a92d-b579f577edb0 | Rodentia | Muridae | Gerbillus sp. | 18.015000 | -15.910000 | K3 |
| http://n2t.net/ark:/65665/34b62626d-8042-4ec4-90f6-c6d5acf21de5 | Rodentia | Muridae | Gerbillus sp. | 18.015000 | -15.910000 | K3 |
| http://n2t.net/ark:/65665/34652dd59-cf7f-46cc-be7d-0dc6dd2b274e | Rodentia | Muridae | Gerbillus sp. | 18.015000 | -15.910000 | K3 |
| http://n2t.net/ark:/65665/32b3d730a-de74-40f0-8314-b39a703130ec | Rodentia | Muridae | Gerbillus sp. | 18.015000 | -15.910000 | K3 |
| http://n2t.net/ark:/65665/36be15852-ff99-4790-861c-6922e3319bd9 | Rodentia | Muridae | Gerbillus sp. | 18.015000 | -15.910000 | K3 |
| http://n2t.net/ark:/65665/3de838fb6-c3e7-418f-a212-6f6fa922f996 | Rodentia | Muridae | Gerbillus sp. | 18.015000 | -15.910000 | K3 |
| http://n2t.net/ark:/65665/338ba5f2c-15a5-4e34-a330-40727861484b | Rodentia | Muridae | Gerbillus sp. | 18.015000 | -15.910000 | K3 |
| http://n2t.net/ark:/65665/3a151870f-6508-476a-b55d-cc152747ba10 | Rodentia | Muridae | Gerbillus sp. | 18.015000 | -15.910000 | K3 |
| http://n2t.net/ark:/65665/3d95e95a5-e168-4076-8e00-4fdda5a0a16c | Rodentia | Muridae | Gerbillus sp. | 18.015000 | -15.910000 | K3 |
| http://n2t.net/ark:/65665/3ea15f44d-787c-4424-862a-ffba7727339f | Rodentia | Muridae | Gerbillus sp. | 18.015000 | -15.910000 | K3 |
| http://n2t.net/ark:/65665/3663e0811-3931-452f-8279-11efd7628302 | Rodentia | Muridae | Gerbillus sp. | 18.015000 | -15.910000 | K3 |
| http://n2t.net/ark:/65665/3314e79b1-d281-4bae-80aa-e04afa6135b4 | Rodentia | Muridae | Gerbillus sp. | 18.015000 | -15.910000 | K3 |
| http://n2t.net/ark:/65665/32c529307-9b70-4259-8e2b-57a198714d5d | Rodentia | Muridae | Gerbillus sp. | 18.015000 | -15.910000 | K3 |
| http://n2t.net/ark:/65665/3d4bf54bd-02dd-400b-a745-9c9ac6d42421 | Rodentia | Muridae | Gerbillus sp. | 18.015000 | -15.910000 | K3 |
| http://n2t.net/ark:/65665/3b123f6ab-1c35-4f21-a9d4-93eb02a85953 | Rodentia | Muridae | Gerbillus sp. | 18.015000 | -15.910000 | K3 |
| http://n2t.net/ark:/65665/38766b809-15a8-407f-b64f-fe4871221b7d | Rodentia | Muridae | Gerbillus sp. | 18.015000 | -15.910000 | K3 |
| http://n2t.net/ark:/65665/31713cead-d88a-4145-b9ec-090422cbc28b | Rodentia | Muridae | Gerbillus sp. | 18.015000 | -15.910000 | K3 |
| http://n2t.net/ark:/65665/3d7b1222c-2ecc-4013-93ea-35473d68c24c | Rodentia | Muridae | Gerbillus sp. | 18.015000 | -15.910000 | K3 |
| http://n2t.net/ark:/65665/30cf2e54b-624d-410b-92f1-7af02a8c8396 | Rodentia | Muridae | Gerbillus sp. | 18.015000 | -15.910000 | K3 |
| http://n2t.net/ark:/65665/3fb9dc898-6010-40ba-8281-d4f822613c59 | Rodentia | Muridae | Gerbillus sp. | 18.015000 | -15.910000 | K3 |
| http://n2t.net/ark:/65665/35c296a8b-a755-4cb2-b888-37862b051ee2 | Rodentia | Muridae | Gerbillus sp. | 18.015000 | -15.910000 | K3 |
| http://n2t.net/ark:/65665/3b0f87aff-df24-484d-a00e-6c3eafd352a8 | Rodentia | Muridae | Gerbillus sp. | 18.015000 | -15.910000 | K3 |
| http://n2t.net/ark:/65665/3edbbd66e-93f9-4ed5-8b18-582def67d93d | Rodentia | Muridae | Gerbillus sp. | 18.015000 | -15.910000 | K3 |
| http://n2t.net/ark:/65665/3dd483e02-5041-4684-b70b-dacab87a6abb | Rodentia | Muridae | Gerbillus sp. | 18.015000 | -15.910000 | K3 |
| http://n2t.net/ark:/65665/3545fa4dc-6fa2-4772-860a-5c1895aded06 | Rodentia | Muridae | Gerbillus sp. | 18.015000 | -15.910000 | K3 |
| http://n2t.net/ark:/65665/39fd57d6f-1cc6-4c0f-ad2c-da2c52985e5e | Rodentia | Muridae | Gerbillus sp. | 18.015000 | -15.910000 | K3 |
| http://n2t.net/ark:/65665/3b9d95bd5-8f88-4369-bda2-a35f3bbf706f | Rodentia | Muridae | Gerbillus sp. | 18.015000 | -15.910000 | K3 |
| http://n2t.net/ark:/65665/37a7b3165-0454-4aab-8022-1d3afdfd7d7d | Rodentia | Muridae | Gerbillus sp. | 18.015000 | -15.910000 | K3 |
| http://n2t.net/ark:/65665/3daf51203-da23-4bc3-98c9-42e6a398798c | Rodentia | Muridae | Gerbillus sp. | 18.015000 | -15.910000 | K3 |
| http://n2t.net/ark:/65665/30949e2bd-9cde-481c-a732-50c2bfadb126 | Rodentia | Muridae | Gerbillus sp. | 18.015000 | -15.910000 | K3 |
| http://n2t.net/ark:/65665/36989b7d9-6276-4adf-9fce-09d4c37bd9bd | Rodentia | Muridae | Gerbillus sp. | 18.015000 | -15.910000 | K3 |
| http://n2t.net/ark:/65665/37188b090-65d1-4152-ae76-b8414e4d7a9a | Rodentia | Muridae | Gerbillus sp. | 18.015000 | -15.910000 | K3 |
| http://n2t.net/ark:/65665/3d02884ee-8f38-4569-86e9-e48ded51bf0b | Rodentia | Muridae | Gerbillus sp. | 18.015000 | -15.910000 | K3 |
| http://n2t.net/ark:/65665/301cb4b37-0299-4f26-bc17-ad61a4b67935 | Rodentia | Muridae | Gerbillus sp. | 18.015000 | -15.910000 | K3 |
| http://n2t.net/ark:/65665/3f54cc598-5fa1-40db-9562-41de92611673 | Rodentia | Muridae | Gerbillus sp. | 18.015000 | -15.910000 | K3 |
| http://n2t.net/ark:/65665/387612927-a009-4e4f-95c7-259a9228becc | Rodentia | Muridae | Gerbillus sp. | 18.015000 | -15.910000 | K3 |
| http://n2t.net/ark:/65665/376a6ec7f-86f0-465f-b39c-1eb181dac6b8 | Rodentia | Muridae | Gerbillus sp. | 18.015000 | -15.910000 | K3 |
| http://n2t.net/ark:/65665/36e02c8a5-63d7-4718-97f8-c158f464bd29 | Rodentia | Muridae | Gerbillus sp. | 18.015000 | -15.910000 | K3 |
| http://n2t.net/ark:/65665/3b9bed86c-d25f-45c9-8780-2a459e3f0117 | Rodentia | Muridae | Gerbillus sp. | 18.015000 | -15.910000 | K3 |
| http://n2t.net/ark:/65665/327d186ba-5cdf-4056-b30d-8271d8b7ead5 | Rodentia | Muridae | Gerbillus sp. | 18.015000 | -15.910000 | K3 |
| http://n2t.net/ark:/65665/3006148ad-bee2-4e00-a636-cdad1bb030cd | Rodentia | Muridae | Gerbillus sp. | 18.015000 | -15.910000 | K3 |
| http://n2t.net/ark:/65665/388480484-30aa-4735-bf2a-a5be863c24fa | Rodentia | Muridae | Gerbillus sp. | 18.015000 | -15.910000 | K3 |
| http://n2t.net/ark:/65665/386914007-5032-4750-87f1-1f9d3e5a907d | Rodentia | Muridae | Gerbillus sp. | 18.015000 | -15.910000 | K3 |
| http://n2t.net/ark:/65665/33c3654db-d685-422c-9a85-512089f6c2d4 | Rodentia | Muridae | Gerbillus sp. | 18.015000 | -15.910000 | K3 |
| http://n2t.net/ark:/65665/31f9809ce-160c-423e-8d46-35465610a50f | Rodentia | Muridae | Gerbillus sp. | 18.015000 | -15.910000 | K3 |
| http://n2t.net/ark:/65665/301ac7e11-b0cd-4298-8209-d02341870f10 | Rodentia | Muridae | Gerbillus sp. | 18.015000 | -15.910000 | K3 |
| http://n2t.net/ark:/65665/3b96896fa-efc3-4210-8565-149ebfc6dd3c | Rodentia | Muridae | Gerbillus sp. | 18.015000 | -15.910000 | K3 |
| http://n2t.net/ark:/65665/3cd800c5c-88aa-4bff-a818-16836634e835 | Rodentia | Muridae | Gerbillus sp. | 18.015000 | -15.910000 | K3 |
| http://n2t.net/ark:/65665/371849e44-6da7-4961-9267-1f973dc99527 | Rodentia | Muridae | Gerbillus sp. | 18.015000 | -15.910000 | K3 |
| http://n2t.net/ark:/65665/35f935108-2af2-4f9b-9694-ddfb613222ab | Rodentia | Muridae | Gerbillus sp. | 18.015000 | -15.910000 | K3 |
| http://n2t.net/ark:/65665/3c342164f-f775-4b10-9ce4-5ca64c6704ac | Rodentia | Muridae | Gerbillus sp. | 18.015000 | -15.910000 | K3 |
| http://n2t.net/ark:/65665/344d510f2-64c6-48a5-89d9-ff5899d20e37 | Rodentia | Muridae | Gerbillus sp. | 18.015000 | -15.910000 | K3 |
| http://n2t.net/ark:/65665/32bebd7de-faf3-4c93-9f58-238c6bbd2332 | Rodentia | Muridae | Gerbillus sp. | 18.015000 | -15.910000 | K3 |
| http://n2t.net/ark:/65665/3c3bee089-75cb-4f91-ab64-5bc58abe1152 | Rodentia | Muridae | Gerbillus sp. | 18.015000 | -15.910000 | K3 |
| http://n2t.net/ark:/65665/33ece854b-9a51-4345-8b86-5db137ac51ec | Rodentia | Muridae | Gerbillus sp. | 18.015000 | -15.910000 | K3 |
| http://n2t.net/ark:/65665/3945209e5-b3e8-4110-9d52-abf9f3e4ce71 | Rodentia | Muridae | Gerbillus sp. | 18.015000 | -15.910000 | K3 |
| http://n2t.net/ark:/65665/356c9163f-dd91-41af-9918-8795b8009780 | Rodentia | Muridae | Gerbillus sp. | 18.015000 | -15.910000 | K3 |
| http://n2t.net/ark:/65665/33801b750-13af-449c-a336-d5a92eb48810 | Rodentia | Muridae | Gerbillus sp. | 18.015000 | -15.910000 | K3 |
| http://n2t.net/ark:/65665/33efa9794-8627-4f70-9158-71c1090b6149 | Rodentia | Muridae | Gerbillus sp. | 18.015000 | -15.910000 | K3 |
| http://n2t.net/ark:/65665/305a3e39c-0ef4-4597-9a63-47309fcc4551 | Rodentia | Muridae | Gerbillus sp. | 18.015000 | -15.910000 | K3 |
| http://n2t.net/ark:/65665/344e55b17-f5f8-4a57-8e7a-26911005b672 | Rodentia | Muridae | Gerbillus sp. | 18.015000 | -15.910000 | K3 |
| http://n2t.net/ark:/65665/31ccec018-b23e-406a-be8f-fb65ee3eb49b | Rodentia | Muridae | Gerbillus sp. | 18.015000 | -15.910000 | K3 |
| http://n2t.net/ark:/65665/3987d09a7-ea41-4355-90f0-4967f4682d13 | Rodentia | Muridae | Gerbillus sp. | 18.015000 | -15.910000 | K3 |
| http://n2t.net/ark:/65665/3f65c76d0-7e1c-4dd7-9ddd-f02777f09f1d | Rodentia | Muridae | Gerbillus sp. | 18.015000 | -15.910000 | K3 |
| http://n2t.net/ark:/65665/3a698c9ab-b74a-4990-8bfd-2e37361a958e | Rodentia | Muridae | Gerbillus sp. | 18.015000 | -15.910000 | K3 |
| http://n2t.net/ark:/65665/386012ffc-8ffe-4c1c-9cfd-c80a6773f8b1 | Rodentia | Muridae | Gerbillus sp. | 18.015000 | -15.910000 | K3 |
| http://n2t.net/ark:/65665/35ae24f63-66f5-4954-9e9c-0f2c0f0cb9ad | Rodentia | Muridae | Gerbillus sp. | 18.015000 | -15.910000 | K3 |
| http://n2t.net/ark:/65665/31d0e35b8-f3db-420a-98bb-1c124bcc38fb | Rodentia | Muridae | Gerbillus sp. | 18.015000 | -15.910000 | K3 |
| http://n2t.net/ark:/65665/38f4ab4b3-571b-4117-a088-341ac808be77 | Rodentia | Muridae | Gerbillus sp. | 18.015000 | -15.910000 | K3 |
| http://n2t.net/ark:/65665/3a67afc07-d366-495c-a3e9-fd5641a7aeb5 | Rodentia | Muridae | Gerbillus sp. | 18.015000 | -15.910000 | K3 |
| http://n2t.net/ark:/65665/36694b9fb-8ceb-4bd0-ad6f-8a47219da0e4 | Rodentia | Muridae | Gerbillus sp. | 18.015000 | -15.910000 | K3 |
| http://n2t.net/ark:/65665/3bb96a29c-8127-4c3b-bb25-4b7e193d1e56 | Rodentia | Muridae | Gerbillus sp. | 18.015000 | -15.910000 | K3 |
| http://n2t.net/ark:/65665/3a7486777-b8b2-4280-a7e7-a91114d57e2f | Rodentia | Muridae | Gerbillus sp. | 18.015000 | -15.910000 | K3 |
| http://n2t.net/ark:/65665/3896520ca-ad59-484a-b550-975f7c6213f6 | Rodentia | Muridae | Gerbillus sp. | 18.015000 | -15.910000 | K3 |
| http://n2t.net/ark:/65665/3b6048e53-5574-4060-9d4b-70a7a5424ebe | Rodentia | Muridae | Gerbillus sp. | 18.015000 | -15.910000 | K3 |
| http://n2t.net/ark:/65665/3b78ab73e-82f6-4964-b588-7316a7c2b75d | Rodentia | Muridae | Gerbillus sp. | 18.015000 | -15.910000 | K3 |
| http://n2t.net/ark:/65665/3e65824a9-57dc-4ff1-9e74-d935538103fa | Rodentia | Muridae | Gerbillus sp. | 18.015000 | -15.910000 | K3 |
| http://n2t.net/ark:/65665/3254926ae-9040-46b9-a54c-6ed60ce51d77 | Rodentia | Muridae | Gerbillus sp. | 18.015000 | -15.910000 | K3 |
| http://n2t.net/ark:/65665/3f2fb5e9b-7163-4c50-a1bd-f142e8ffd902 | Rodentia | Muridae | Gerbillus sp. | 18.015000 | -15.910000 | K3 |
| http://n2t.net/ark:/65665/3fddca93a-5f31-4cd0-b55d-c6c4cff97e29 | Rodentia | Muridae | Gerbillus sp. | 18.015000 | -15.910000 | K3 |
| http://n2t.net/ark:/65665/3e626af3e-0190-4ebc-8bde-3e4c77a652b1 | Rodentia | Muridae | Gerbillus sp. | 18.015000 | -15.910000 | K3 |
| http://n2t.net/ark:/65665/36f4d901c-10b9-4cb0-b1f1-7d8d13889361 | Rodentia | Muridae | Gerbillus sp. | 18.015000 | -15.910000 | K3 |
| http://n2t.net/ark:/65665/3013debd1-ed8d-47c8-8885-fffe12a592cd | Rodentia | Muridae | Gerbillus sp. | 18.015000 | -15.910000 | K3 |
| http://n2t.net/ark:/65665/3ad507082-b02b-43dd-8a83-fe62531882d4 | Rodentia | Muridae | Gerbillus sp. | 18.015000 | -15.910000 | K3 |
| http://n2t.net/ark:/65665/399b2c3b6-f2e8-4540-baf2-905934d2e7bc | Rodentia | Muridae | Gerbillus sp. | 18.015000 | -15.910000 | K3 |
| http://n2t.net/ark:/65665/33dad291f-116e-4b51-8eea-eb090a969731 | Rodentia | Muridae | Gerbillus sp. | 18.015000 | -15.910000 | K3 |
| http://n2t.net/ark:/65665/34d0e11cd-0dcf-4b52-b331-ab1d20339b50 | Rodentia | Muridae | Gerbillus sp. | 18.015000 | -15.910000 | K3 |
| http://n2t.net/ark:/65665/3d6ac4a9f-9147-41d0-bcbf-e4e6dabbed5a | Rodentia | Muridae | Gerbillus sp. | 18.015000 | -15.910000 | K3 |
| http://n2t.net/ark:/65665/3b5020547-91a4-4dad-9bae-c307dd25958d | Rodentia | Muridae | Gerbillus sp. | 18.015000 | -15.910000 | K3 |
| http://n2t.net/ark:/65665/37466e914-144d-46ef-8bee-9e412b2b0c9f | Rodentia | Muridae | Gerbillus sp. | 18.015000 | -15.910000 | K3 |
| http://n2t.net/ark:/65665/352767e5d-f3fa-41c0-a66c-2f25a5c82771 | Rodentia | Muridae | Gerbillus sp. | 18.015000 | -15.910000 | K3 |
| http://n2t.net/ark:/65665/30884c9ab-3902-488d-8194-4efb07ec6425 | Rodentia | Muridae | Gerbillus sp. | 18.015000 | -15.910000 | K3 |
| http://n2t.net/ark:/65665/354c23f61-ebfe-4bad-b4eb-eea14d3dd629 | Rodentia | Muridae | Gerbillus sp. | 18.015000 | -15.910000 | K3 |
| http://n2t.net/ark:/65665/30e94f010-c18a-4d86-ae54-5212bf8b886b | Rodentia | Muridae | Gerbillus sp. | 18.015000 | -15.910000 | K3 |
| http://n2t.net/ark:/65665/3d7f798b6-ba7d-44ef-9ace-7b9e38b723b1 | Rodentia | Muridae | Gerbillus sp. | 18.015000 | -15.910000 | K3 |
| http://n2t.net/ark:/65665/38c9cf67b-d137-4a11-b07a-fe1dffe31b86 | Rodentia | Muridae | Gerbillus sp. | 18.015000 | -15.910000 | K3 |
| http://n2t.net/ark:/65665/304b1e770-2fcc-45e9-a4a1-3e78df6fc26f | Rodentia | Muridae | Gerbillus sp. | 18.015000 | -15.910000 | K3 |
| http://n2t.net/ark:/65665/332efeae2-b751-42b6-b002-cfa16963bb51 | Rodentia | Muridae | Gerbillus sp. | 18.015000 | -15.910000 | K3 |
| http://n2t.net/ark:/65665/3296048bd-a779-413a-bf2a-9b587422774c | Rodentia | Muridae | Gerbillus sp. | 18.015000 | -15.910000 | K3 |
| http://n2t.net/ark:/65665/306202f95-a156-4775-aa2f-6f6bba3bb106 | Rodentia | Muridae | Gerbillus sp. | 18.015000 | -15.910000 | K3 |
| http://n2t.net/ark:/65665/3f336adf5-0f82-4a47-b1fe-cd2d6573bc46 | Rodentia | Muridae | Gerbillus sp. | 18.015000 | -15.910000 | K3 |
| http://n2t.net/ark:/65665/382c72b86-881e-425a-aded-cf05205ddcf8 | Rodentia | Muridae | Gerbillus sp. | 18.015000 | -15.910000 | K3 |
| http://n2t.net/ark:/65665/351235408-0c65-4df3-81fa-078cbff45bbd | Rodentia | Muridae | Gerbillus sp. | 18.015000 | -15.910000 | K3 |
| http://n2t.net/ark:/65665/35964886a-0455-47d1-acfa-5c95b365b85c | Rodentia | Muridae | Gerbillus sp. | 18.015000 | -15.910000 | K3 |
| http://n2t.net/ark:/65665/324c5adea-de7c-48f1-ae9f-7e53dac661ba | Rodentia | Muridae | Gerbillus sp. | 18.015000 | -15.910000 | K3 |
| http://n2t.net/ark:/65665/33be076f8-eb40-471c-a01e-df3a4f07cf0b | Rodentia | Muridae | Gerbillus sp. | 18.015000 | -15.910000 | K3 |
| http://n2t.net/ark:/65665/38c5ea779-888f-4d14-9332-4ca0e7935bd5 | Rodentia | Muridae | Gerbillus sp. | 18.015000 | -15.910000 | K3 |
| http://n2t.net/ark:/65665/3baedca37-1183-4c03-bc0b-491bd64b59a5 | Rodentia | Muridae | Gerbillus sp. | 18.015000 | -15.910000 | K3 |
| http://n2t.net/ark:/65665/34e3a7d31-834d-4fcc-8a3a-a01836af8b2f | Rodentia | Muridae | Gerbillus sp. | 18.015000 | -15.910000 | K3 |
| http://n2t.net/ark:/65665/32825c054-1af6-4861-94f2-7b6ce0fd1f2f | Rodentia | Muridae | Gerbillus sp. | 18.015000 | -15.910000 | K3 |
| http://n2t.net/ark:/65665/351d5e83f-3288-441b-a695-1d48ba9d901c | Rodentia | Muridae | Gerbillus sp. | 18.015000 | -15.910000 | K3 |
| http://n2t.net/ark:/65665/30d585954-0b55-42bb-ba67-3ca69756bf62 | Rodentia | Muridae | Gerbillus sp. | 18.015000 | -15.910000 | K3 |
| http://n2t.net/ark:/65665/3ebe4299e-9726-483e-9038-a39d9708afaf | Rodentia | Muridae | Gerbillus sp. | 18.015000 | -15.910000 | K3 |
| http://n2t.net/ark:/65665/3abc2612c-f8d4-465c-9932-2b6c01240c01 | Rodentia | Muridae | Gerbillus sp. | 18.015000 | -15.910000 | K3 |
| http://n2t.net/ark:/65665/3b9673ab5-7340-4872-8cff-7118dba00a02 | Rodentia | Muridae | Gerbillus sp. | 18.015000 | -15.910000 | K3 |
| http://n2t.net/ark:/65665/37c256435-27ad-46b6-b4d7-94e658f6aca3 | Rodentia | Muridae | Gerbillus sp. | 18.015000 | -15.910000 | K3 |
| http://n2t.net/ark:/65665/325ae288a-ba40-45eb-a886-681b79cdc2f1 | Rodentia | Muridae | Gerbillus sp. | 18.015000 | -15.910000 | K3 |
| http://n2t.net/ark:/65665/38eb20880-0046-4fd3-a954-fe426c6b7409 | Rodentia | Muridae | Gerbillus sp. | 18.015000 | -15.910000 | K3 |
| http://n2t.net/ark:/65665/390bb48c8-eccd-4f85-b994-dda262ae153b | Rodentia | Muridae | Gerbillus sp. | 18.015000 | -15.910000 | K3 |
| http://n2t.net/ark:/65665/3aeb7383f-11fa-445c-9cfe-5e649212c7ba | Rodentia | Muridae | Gerbillus sp. | 18.015000 | -15.910000 | K3 |
| http://n2t.net/ark:/65665/3ac5cdd2c-0434-46fa-be3e-437497e19b63 | Rodentia | Muridae | Gerbillus sp. | 18.015000 | -15.910000 | K3 |
| http://n2t.net/ark:/65665/3a4a8a9c7-596d-4b58-8e0c-3927ba3f5872 | Rodentia | Muridae | Gerbillus sp. | 18.015000 | -15.910000 | K3 |
| http://n2t.net/ark:/65665/37d4768f2-1c42-4683-96e9-dad403996f33 | Rodentia | Muridae | Gerbillus sp. | 18.015000 | -15.910000 | K3 |
| http://n2t.net/ark:/65665/3a526e645-2bbf-4c82-a199-3bc061dddcbe | Rodentia | Muridae | Gerbillus sp. | 18.015000 | -15.910000 | K3 |
| http://n2t.net/ark:/65665/30c43f83a-101b-4fc4-b17a-a43e8e8d0860 | Rodentia | Muridae | Gerbillus sp. | 18.015000 | -15.910000 | K3 |
| http://n2t.net/ark:/65665/3cca6aed4-a53c-4e7f-b3ae-da80c69e0f63 | Rodentia | Muridae | Gerbillus sp. | 18.015000 | -15.910000 | K3 |
| http://n2t.net/ark:/65665/3c8d27592-88a9-48d0-a606-df180eb981c6 | Rodentia | Muridae | Gerbillus sp. | 18.015000 | -15.910000 | K3 |
| http://n2t.net/ark:/65665/3c7b0c4cd-be7d-44b0-ab4f-bf665b92927d | Rodentia | Muridae | Gerbillus sp. | 18.015000 | -15.910000 | K3 |
| http://n2t.net/ark:/65665/3552ef0da-7712-4a17-9e01-ced14c4e5723 | Rodentia | Muridae | Gerbillus sp. | 18.015000 | -15.910000 | K3 |
| http://n2t.net/ark:/65665/3bbc595b3-65e5-4184-9d80-05e96b533f32 | Rodentia | Muridae | Gerbillus sp. | 18.015000 | -15.910000 | K3 |
| http://n2t.net/ark:/65665/3b27a7ed1-ea12-44b0-9fd7-7f0b477b8976 | Rodentia | Muridae | Gerbillus sp. | 18.015000 | -15.910000 | K3 |
| http://n2t.net/ark:/65665/33886bb5c-e42a-44e4-862f-ecf7f2037dd0 | Rodentia | Muridae | Gerbillus sp. | 18.015000 | -15.910000 | K3 |
| http://n2t.net/ark:/65665/3d365f327-0ef4-4989-b163-c51e9625017b | Rodentia | Muridae | Gerbillus sp. | 18.015000 | -15.910000 | K3 |
| http://n2t.net/ark:/65665/37e385aec-5abe-4e8b-866c-78615759cdfa | Rodentia | Muridae | Gerbillus sp. | 18.015000 | -15.910000 | K3 |
| http://n2t.net/ark:/65665/331d84e04-20cf-469d-9e9a-d60504d735a3 | Rodentia | Muridae | Gerbillus sp. | 18.015000 | -15.910000 | K3 |
| http://n2t.net/ark:/65665/3bb955834-925e-4a4c-9dfb-ae5d9d18047a | Rodentia | Muridae | Gerbillus sp. | 18.015000 | -15.910000 | K3 |
| http://n2t.net/ark:/65665/36bbedd9a-318d-4bf3-9a4d-ec5ab28fca26 | Rodentia | Muridae | Gerbillus sp. | 18.015000 | -15.910000 | K3 |
| http://n2t.net/ark:/65665/369cc638e-06e6-4fcb-b84c-c55a36406278 | Rodentia | Muridae | Gerbillus sp. | 18.015000 | -15.910000 | K3 |
| http://n2t.net/ark:/65665/3bb77e2e0-4987-403c-8a6c-7c1cd7e5e712 | Rodentia | Muridae | Gerbillus sp. | 18.015000 | -15.910000 | K3 |
| http://n2t.net/ark:/65665/350625178-7f44-4627-85d6-6901c9cd432b | Rodentia | Muridae | Gerbillus sp. | 18.015000 | -15.910000 | K3 |
| http://n2t.net/ark:/65665/3ab63d1c8-809f-43aa-a67e-7b9a21b83313 | Rodentia | Muridae | Gerbillus sp. | 18.015000 | -15.910000 | K3 |
| http://n2t.net/ark:/65665/3851e37c9-d9fb-49fd-b012-07fc1e013ff5 | Rodentia | Muridae | Gerbillus sp. | 18.015000 | -15.910000 | K3 |
| http://n2t.net/ark:/65665/32962f7a3-a1e7-4aac-ba5d-44617c9ac730 | Rodentia | Muridae | Gerbillus sp. | 18.015000 | -15.910000 | K3 |
| http://n2t.net/ark:/65665/3b7fc3846-e3c9-438c-a986-2a99923cdf48 | Rodentia | Muridae | Gerbillus sp. | 18.015000 | -15.910000 | K3 |
| http://n2t.net/ark:/65665/371049927-9db5-4224-bc21-9611860e8e8d | Rodentia | Muridae | Gerbillus sp. | 18.015000 | -15.910000 | K3 |
| http://n2t.net/ark:/65665/38d93cf04-53a8-4d53-bce7-1cb85513dbe2 | Rodentia | Muridae | Gerbillus sp. | 18.015000 | -15.910000 | K3 |
| http://n2t.net/ark:/65665/3651a6468-97b0-4fda-af92-962a3d2aaf69 | Rodentia | Muridae | Gerbillus sp. | 18.015000 | -15.910000 | K3 |
| http://n2t.net/ark:/65665/3b23917d6-5790-430b-b691-7840cb60e6e7 | Rodentia | Muridae | Gerbillus sp. | 18.015000 | -15.910000 | K3 |
| http://n2t.net/ark:/65665/3b62f7ad6-9d8b-4fd1-a67e-0aa5cd932c18 | Rodentia | Muridae | Gerbillus sp. | 18.015000 | -15.910000 | K3 |
| http://n2t.net/ark:/65665/3d4375a18-0105-48f1-96b5-5a704e573abe | Rodentia | Muridae | Gerbillus sp. | 18.015000 | -15.910000 | K3 |
| http://n2t.net/ark:/65665/361acd2bd-db60-4bf0-abe7-8cca5adebb1c | Rodentia | Muridae | Gerbillus sp. | 18.015000 | -15.910000 | K3 |
| http://n2t.net/ark:/65665/338b52268-1b70-4dbc-b967-73bdbd7ad58a | Rodentia | Muridae | Gerbillus sp. | 18.015000 | -15.910000 | K3 |
| http://n2t.net/ark:/65665/352b5a653-e706-46a9-b5db-661db450bb45 | Rodentia | Muridae | Gerbillus sp. | 18.015000 | -15.910000 | K3 |
| http://n2t.net/ark:/65665/399494f5e-4a01-4c41-86ef-18c3dae8f16a | Rodentia | Muridae | Gerbillus sp. | 18.015000 | -15.910000 | K3 |
| http://n2t.net/ark:/65665/3e8fe078c-aa50-48c1-880f-ee0b1317f154 | Rodentia | Muridae | Gerbillus sp. | 18.015000 | -15.910000 | K3 |
| http://n2t.net/ark:/65665/33141f307-8eb4-4318-83fa-c4a8a3448d13 | Rodentia | Muridae | Gerbillus sp. | 18.015000 | -15.910000 | K3 |
| http://n2t.net/ark:/65665/3b8a8a3e9-e519-4ee3-a4da-0051022dae7b | Rodentia | Muridae | Gerbillus sp. | 18.015000 | -15.910000 | K3 |
| http://n2t.net/ark:/65665/345cce22e-6127-4fa0-986c-cac8a66f6c27 | Rodentia | Muridae | Gerbillus sp. | 18.015000 | -15.910000 | K3 |
| http://coldb.mnhn.fr/catalognumber/mnhn/zm/mo-1992-1495 | Rodentia | Muridae | Gerbillus sp. | 18.267000 | -16.016900 | K3 |
| http://n2t.net/ark:/65665/308ad4949-42c7-4737-96a7-abbe1b7bf030 | Rodentia | Muridae | Gerbillus sp. | 18.185000 | -16.011000 | K3 |
| http://n2t.net/ark:/65665/388c52e44-139d-4a6f-b367-7ce6f503ca0f | Rodentia | Muridae | Gerbillus sp. | 18.185000 | -16.011000 | K3 |
| http://n2t.net/ark:/65665/3e627294e-0e6c-4461-8dba-ab16820206ee | Rodentia | Muridae | Gerbillus sp. | 18.185000 | -16.011000 | K3 |
| http://n2t.net/ark:/65665/316e5e758-348a-47dc-a778-ee7d06056b65 | Rodentia | Muridae | Gerbillus sp. | 18.185000 | -16.011000 | K3 |
| http://n2t.net/ark:/65665/3b35188b6-9b4d-46a9-8eeb-4e39cdd30d78 | Rodentia | Muridae | Gerbillus sp. | 18.185000 | -16.011000 | K3 |
| http://n2t.net/ark:/65665/32be2d8d0-d077-4f91-96c6-9cd20bb0fa42 | Rodentia | Muridae | Gerbillus sp. | 18.185000 | -16.011000 | K3 |
| http://n2t.net/ark:/65665/38ef61f06-b232-4a38-a088-913fe977bd17 | Rodentia | Muridae | Gerbillus sp. | 18.185000 | -16.011000 | K3 |
| http://n2t.net/ark:/65665/3051895f9-83da-409a-abe4-3bcad1c2dfe4 | Rodentia | Muridae | Gerbillus sp. | 18.185000 | -16.011000 | K3 |
| http://n2t.net/ark:/65665/3105cc277-6049-4896-808a-f731a27f3377 | Rodentia | Muridae | Gerbillus sp. | 18.185000 | -16.011000 | K3 |
| http://n2t.net/ark:/65665/3d447e5be-4b06-4d5b-b1e8-ae13149bed80 | Rodentia | Muridae | Gerbillus sp. | 18.185000 | -16.011000 | K3 |
| http://n2t.net/ark:/65665/3b5c75ae5-33a8-4886-9696-9fd16fa98fb2 | Rodentia | Muridae | Gerbillus sp. | 18.185000 | -16.011000 | K3 |
| http://n2t.net/ark:/65665/3b0a0a509-b74b-4051-9ecf-859b5b08893e | Rodentia | Muridae | Gerbillus sp. | 18.185000 | -16.011000 | K3 |
| http://n2t.net/ark:/65665/37cbbc81c-12c6-4cd8-867a-0e4b928dcb23 | Rodentia | Muridae | Gerbillus sp. | 18.185000 | -16.011000 | K3 |
| http://n2t.net/ark:/65665/35236e9cd-cd75-49c1-9c62-010614ce2f73 | Rodentia | Muridae | Gerbillus sp. | 18.185000 | -16.011000 | K3 |
| http://n2t.net/ark:/65665/3252d2ce6-ef29-4933-932f-58ecdde4e836 | Rodentia | Muridae | Gerbillus sp. | 18.185000 | -16.011000 | K3 |
| http://n2t.net/ark:/65665/39c033c99-879b-4699-847c-a14662b12e32 | Rodentia | Muridae | Gerbillus sp. | 18.185000 | -16.011000 | K3 |
| http://n2t.net/ark:/65665/3a9b86b2a-f249-4e90-94e3-f004ee785c0d | Rodentia | Muridae | Gerbillus sp. | 18.185000 | -16.011000 | K3 |
| http://n2t.net/ark:/65665/35e6b8704-d28d-4d2f-b115-89603cb13369 | Rodentia | Muridae | Gerbillus sp. | 18.185000 | -16.011000 | K3 |
| http://n2t.net/ark:/65665/327bb4ded-513a-4103-b00d-17a5bbc1a9fd | Rodentia | Muridae | Gerbillus sp. | 18.185000 | -16.011000 | K3 |
| http://n2t.net/ark:/65665/383da18a3-07bb-4ef7-891a-e0e22f14519c | Rodentia | Muridae | Gerbillus sp. | 18.185000 | -16.011000 | K3 |
| http://n2t.net/ark:/65665/35f200804-d69e-4d97-a207-04ca13d9eb1f | Rodentia | Muridae | Gerbillus sp. | 18.185000 | -16.011000 | K3 |
| http://n2t.net/ark:/65665/3cd732578-737a-4959-b695-249e83020dcc | Rodentia | Muridae | Gerbillus sp. | 18.185000 | -16.011000 | K3 |
| http://coldb.mnhn.fr/catalognumber/mnhn/zm/mo-1992-1499 | Rodentia | Muridae | Gerbillus sp. | 18.115564 | -16.015721 | K3 |
| http://coldb.mnhn.fr/catalognumber/mnhn/zm/mo-1990-57 | Rodentia | Muridae | Gerbillus sp. | 18.115564 | -16.015721 | K3 |
| http://coldb.mnhn.fr/catalognumber/mnhn/zm/mo-2001-133 | Rodentia | Muridae | Gerbillus sp. | 18.350000 | -11.683000 | J7 |
| http://coldb.mnhn.fr/catalognumber/mnhn/zm/mo-2001-132 | Rodentia | Muridae | Gerbillus sp. | 18.350000 | -11.683000 | J7 |
| http://coldb.mnhn.fr/catalognumber/mnhn/zm/mo-2001-131 | Rodentia | Muridae | Gerbillus sp. | 18.350000 | -11.683000 | J7 |
| http://coldb.mnhn.fr/catalognumber/mnhn/zm/mo-2001-130 | Rodentia | Muridae | Gerbillus sp. | 18.350000 | -11.683000 | J7 |
| http://coldb.mnhn.fr/catalognumber/mnhn/zm/mo-2001-129 | Rodentia | Muridae | Gerbillus sp. | 18.350000 | -11.683000 | J7 |
| http://coldb.mnhn.fr/catalognumber/mnhn/zm/mo-2001-148 | Rodentia | Muridae | Gerbillus sp. | 18.250000 | -11.817000 | K7 |
| http://coldb.mnhn.fr/catalognumber/mnhn/zm/mo-2001-147 | Rodentia | Muridae | Gerbillus sp. | 18.250000 | -11.817000 | K7 |
| http://coldb.mnhn.fr/catalognumber/mnhn/zm/mo-2001-146 | Rodentia | Muridae | Gerbillus sp. | 18.250000 | -11.817000 | K7 |
| http://coldb.mnhn.fr/catalognumber/mnhn/zm/mo-2001-145 | Rodentia | Muridae | Gerbillus sp. | 18.250000 | -11.817000 | K7 |
| http://coldb.mnhn.fr/catalognumber/mnhn/zm/mo-2001-144 | Rodentia | Muridae | Gerbillus sp. | 18.250000 | -11.817000 | K7 |
| http://coldb.mnhn.fr/catalognumber/mnhn/zm/mo-2001-217 | Rodentia | Muridae | Gerbillus sp. | 16.248000 | -16.512000 | M2 |
| http://coldb.mnhn.fr/catalognumber/mnhn/zm/mo-2001-46 | Rodentia | Muridae | Gerbillus sp. | 16.242000 | -16.512000 | M2 |
| http://coldb.mnhn.fr/catalognumber/mnhn/zm/mo-2001-169 | Rodentia | Muridae | Gerbillus sp. | 17.950000 | -12.267000 | K7 |
| http://coldb.mnhn.fr/catalognumber/mnhn/zm/mo-2001-95 | Rodentia | Muridae | Gerbillus sp. | 17.950000 | -12.267000 | K7 |
| http://coldb.mnhn.fr/catalognumber/mnhn/zm/mo-1995-592 | Rodentia | Muridae | Gerbillus sp. | 19.831000 | -15.923000 | I3 |
| http://coldb.mnhn.fr/catalognumber/mnhn/zm/mo-1995-571 | Rodentia | Muridae | Gerbillus sp. | 19.831000 | -15.923000 | I3 |
| http://coldb.mnhn.fr/catalognumber/mnhn/zm/mo-1969-225 | Rodentia | Muridae | Gerbillus sp. | 19.831000 | -15.923000 | I3 |
| http://coldb.mnhn.fr/catalognumber/mnhn/zm/mo-1969-228 | Rodentia | Muridae | Gerbillus sp. | 19.831000 | -15.923000 | I3 |
| http://coldb.mnhn.fr/catalognumber/mnhn/zm/mo-1969-227 | Rodentia | Muridae | Gerbillus sp. | 19.831000 | -15.923000 | I3 |
[truncated: 253,203 more chars]
