# Supplementary material for: Diversity, distribution and conservation of land mammals in Mauritania, North-West Africa
Source: PLoS One. 2022 Aug 1;17(8):e0269870. doi: 10.1371/journal.pone.0269870 (PMC9342785; doi:10.1371/journal.pone.0269870)
Supplement: S4 Text — For each species (following the nomenclature of Mammal Species of the World; [1]) it is presented and discussed the details on the reported observation or range. For species with disagreements in taxonomy, the alternative name given by IUCN Red List is given between brackets. (DOCX) [file pone.0269870.s022.docx]

**S17 Text. Taxonomic list of land mammals with reported occurrence in Mauritania not considered.** For each species (following the nomenclature of Mammal Species of the World; [1]) it is presented and discussed the details on the reported observation or range. For species with disagreements in taxonomy, the alternative name given by IUCN Red List is given between brackets.

| Species | Details |
| --- | --- |
| Carnivora |  |
| Canidae |  |
| *Canis adustus* Sundevall, 1847 | IUCN range polygon reports occurrence along the southern border, from the Province of Trarza eastwards to Hodh Ech Chargui [2]. There are no vouchers deposited in museum collections neither confirmed records of the species in Mauritania. One observation available without photograph (OBS.98178109; [3]) reported as incorrect by the original source (<https://observation.org/observation/98178109>). Not considered by the Mammal Species of the World [1] as occurring in Mauritania. Possible overestimation of the IUCN range polygon for the species |
| Herpestidae |  |
| *Mungos mungo* (Gmelin, 1788) | Reported as possible to occur in the Diawling National Park based on questionnaires [4]. There are no vouchers deposited in museum collections neither confirmed records of the species in Mauritania. Closest known populations are from the Gambia River valley in Senegal and The Gambia [2]. Not considered by the Mammal Species of the World [1] neither by the IUCN Red List [2] as occurring in Mauritania |
| Mustelidae |  |
| *Hydrictis maculicollis* (Lichtenstein, 1835) | Reported as possible to occur along the Senegal River valley [5]. There are no vouchers deposited in museum collections neither confirmed records of the species in Mauritania. Closest known populations are from Guinea-Bissau [2]. Not considered by the Mammal Species of the World [1] neither by the IUCN Red List [2] as occurring in Mauritania |
| *Poecilogale albinucha* (Gray, 1864) | Reported as possible to occur in the Senegal River delta [5]. There are no vouchers deposited in museum collections neither confirmed records of the species in Mauritania. Closest known populations are from Southern Africa [2]. Not considered by the Mammal Species of the World [1] neither by the IUCN Red List [2] as occurring in Mauritania |
| Viverridae |  |
| *Genetta pardina* I. Geoffroy Saint-Hilaire, 1832 | IUCN range polygon reports occurrence along the southern border, from the Province of Trarza eastwards to Assaba [2]. There are no vouchers deposited in museum collections neither confirmed records of the species in Mauritania. Not considered by the Mammal Species of the World [1] as occurring in Mauritania. Possible overestimation of the IUCN range polygon for the species |
| *Genetta thierryi* Matschie, 1902 | IUCN range polygon reports occurrence in the Province of Guidimaka [2]. There are no vouchers deposited in museum collections neither confirmed records of the species in Mauritania. Not considered by the Mammal Species of the World [1] as occurring in Mauritania. Possible overestimation of the IUCN range polygon for the species |
| Chiroptera |  |
| Emballonuridae |  |
| *Taphozous mauritianus* É. Geoffroy, 1818 | Reported to occur in Mauritania by the IUCN Red List but the range polygon does not depict occurrence in the country [2]. There are no vouchers deposited in museum collections neither confirmed records of the species in Mauritania. Possible confusion with other country (e.g. Mauritius) where the species occurs. Not considered by the Mammal Species of the World [1] neither by the African Chiroptera Report [6] as occurring in Mauritania |
| Pteropodidae |  |
| *Epomophorus gambianus* (Ogilby, 1835) | Reported as possible to occur in the Senegal River delta based on questionnaires [4]. There are no vouchers deposited in museum collections neither confirmed records of the species in Mauritania. Closest known populations are from north-eastern Senegal and south-western Mali [2]. Not considered by the Mammal Species of the World [1], neither by the IUCN Red List [2], nor the African Chiroptera Report [6] as occurring in Mauritania |
| Vespertilionidae |  |
| *Scotophilus nigrita* (Schreber, 1774) | Reported as possible to occur in the Senegal River delta based on questionnaires [4]. There are no vouchers deposited in museum collections neither confirmed records of the species in Mauritania. Closest known populations are from north-western Senegal [2]. Not considered by the Mammal Species of the World [1], neither by the IUCN Red List [2], nor the African Chiroptera Report [6] as occurring in Mauritania |
| Lagomorpha |  |
| Leporidae |  |
| *Lepus capensis* Linnaeus, 1758  *Lepus microtis* (=*Lepus victoriae*) Heuglin, 1865 | Both *Lepus capensis* and *L. microtis* are reported to occur in Mauritania [1, 2], but the taxonomic status of African hares requires a thorough revision [7]. Additional morphological and genetic studies are needed to understand the status and range of both species, in Africa and Mauritania |
| Primates |  |
| Cercopithecidae |  |
| *Papio anubis* (Lesson, 1827) | IUCN range polygon reports occurrence along southern border, from the Province of Assaba eastwards to Hodh el Gharbi [2]. There are no vouchers deposited in museum collections neither confirmed records of the species in Mauritania. Closest known populations are in the Boucle du Baoulé, Mali [8]. Not considered by the Mammal Species of the World [1] as occurring in Mauritania. Possible overestimation of the IUCN range polygon for the species |
| Rodentia |  |
| Gliridae |  |
| *Graphiurus nagtglasii* Jentink, 1888 | Reported as possible to occur in the Senegal River delta based on questionnaires [4]. There are no vouchers deposited in museum collections neither confirmed records of the species in Mauritania. Closest known populations are from Sierra Leone [2]. Not considered by the Mammal Species of the World [1] neither by the IUCN Red List [2] as occurring in Mauritania |
| Muridae |  |
| *Acomys cahirinus* (É. Geoffroy, 1803) | IUCN range polygon reports occurrence in the northern regions, in the Province of Tiris-Zemmour [2]. There are no vouchers deposited in museum collections neither confirmed records of the species in Mauritania. Not considered by the Mammal Species of the World [1] as occurring in Mauritania. Possible overestimation of the IUCN range polygon for the species |
| *Dasymys incomtus* (Sundevall, 1847) | Reported as possible to occur in the Senegal River delta [5]. There are no vouchers deposited in museum collections neither confirmed records of the species in Mauritania. Closest known populations are from Southern Africa [2]. Not considered by the Mammal Species of the World [1] neither by the IUCN Red List [2] as occurring in Mauritania |
| *Gerbillus nanus* Blanford, 1875 | IUCN range polygon reports occurrence in almost all Mauritania [2]. Taxonomic assessments have divided African and Asian populations of *Gerbillus nanus*, with populations of *G. nanus* in Africa are referred as *G. amoenus*, while *G. nanus* is restricted to populations in Asia [9, 10] |
| Nesomyidae |  |
| *Cricetomys gambianus* Waterhouse, 1840 | Reported as possible to occur in the Senegal River delta based on questionnaires [4]. There are no vouchers deposited in museum collections neither confirmed records of the species in Mauritania. Closest known populations are from north-western Senegal [2]. Not considered by the Mammal Species of the World [1] neither by the IUCN Red List [2] as occurring in Mauritania |
| Thryonomyidae |  |
| *Thryonomys swinderianus* (Temminck, 1827) | Reported as possible to occur along the Senegal River valley [5]. There are no vouchers deposited in museum collections neither confirmed records of the species in Mauritania. Closest known populations are from the Gambia River valley in Senegal and The Gambia [2]. Not considered by the Mammal Species of the World [1] neither by the IUCN Red List [2] as occurring in Mauritania |
| Soricomorpha |  |
| Soricidae |  |
| *Crocidura tarfayensis* Vesmanis & Vesmanis, 1980 | IUCN range polygon reports occurrence in the north-western region, in the Province of Dakhlet-Nouâdhibou [2]. There are no vouchers deposited in museum collections neither confirmed records of the species in Mauritania. Closest known populations are from the Peninsula of Dakhla, Morocco [11]. Possible overestimation of the IUCN range polygon for the species |

[1] Wilson DE, Reeder DAE (editors). Mammal Species of the World. A Taxonomic and Geographic Reference (3rd ed). Johns Hopkins University Press. 2005; 2: 142 pp. [cited 2021 October 12]. Available from: http://www.press.jhu.edu.

[2] IUCN. The IUCN Red List of Threatened Species. Version 2021-2. 2021. [cited 2021 October 12]. Available from: https://www.iucnredlist.org.

[3] GBIF.org. GBIF Occurrence Download. [cited 2021 November 05]. Available from: https://doi.org/10.15468/dl.5n5czb.

[4] National Research Council. Environmental Degradation in Mauritania. National Academy Press, Washington; 1981.

[5] Hughes RH, Hughes JS, Bernacsek G. Mauritania. In: Bernacsek, G.M., Hughes, J.S., Hughes, R.H. (Eds.). A Directory of African Wetlands. IUCN, UNEP, WCMC, Gland; 1992.

[6] ACR (2020). African Chiroptera Report 2020. V. Van Cakenberghe and E.C.J. Seamark (Eds). AfricanBats NPC, Pretoria.

[7] Lado S, Alves PC, Islam MZ, Brito JC, Melo-Ferreira J. The evolutionary history of the Cape hare (*Lepus capensis* sensu lato): insights for systematics and biogeography. Heredity. 2019; 123: 634-646.

[8] Kopp GH, Ferreira da Silva MJ, Fischer J, Brito JC, Regnaut S, Roos C, Zinner D. The influence of social systems on patterns of mitochondrial DNA variation in Baboons. Int J Primatol. 2014; 35: 210-225.

[9] Ndiaye, A., Shanas, U., Chevret, P., & Granjon, L. (2013). Molecular variation and chromosomal stability within Gerbillus nanus (Rodentia, Gerbillinae): taxonomic and biogeographic implications. Mammalia, 77(1), 105-111

[10] Ndiaye, A., Tatard, C., Stanley, W., & Granjon, L. (2016). Taxonomic hypotheses regarding the genus Gerbillus (Rodentia, Muridae, Gerbillinae) based on molecular analyses of museum specimens. ZooKeys, (566), 145

[11] Aulagnier S, Cuzin F, Thevenot M. Mammifères Sauvages du Maroc. Peuplement, Répartition, Écologie. Société Française pour l'Étude et la Protection des Mammifères, Paris; 2017.
