# Supplementary material for: Diversity, distribution and conservation of land mammals in Mauritania, North-West Africa
Source: PLoS One. 2022 Aug 1;17(8):e0269870. doi: 10.1371/journal.pone.0269870 (PMC9342785; doi:10.1371/journal.pone.0269870)
Supplement: S5 Text — Details on the national conservation status assessment of land mammals species in Mauritania following the recommendations of IUCN [1, 2], including the global Red List status (GRL; [3]), the original national Red List status (NRL Original) and criteria, the final national Red List status (NRL Final) after considering if each species is endemic or nearly-endemic to Mauritania (Endemism), if the Mauritanian populations are a sink in relation to neighbouring populations (Sink), if Mauritanian populations can be rescued by neighbouring populations (Rescue) which implied a downgrading of the NRL Original (Graded). The extent of occurrence (EOO) and comments on calculation are also provided. (DOCX) [file pone.0269870.s023.docx]

**S20 Text –National Red List assessment.** Details on the national conservation status assessment of land mammals species in Mauritania following the recommendations of IUCN [1,2], including the global Red List status (GRL; [3]), the original national Red List status (NRL Original) and criteria, the final national Red List status (NRL Final) after considering if each species is endemic or nearly-endemic to Mauritania (Endemism), if the Mauritanian populations are a sink in relation to neighbouring populations (Sink), if Mauritanian populations can be rescued by neighbouring populations (Rescue) which implied a downgrading of the NRL Original (Graded). The extent of occurrence (EOO) and comments on calculation are also provided.

| Taxon | GRL | NRL Final | NRL Original | Criteria | Graded | Rescue | Sink | Endemism | EOO (km^2^) | Comment |
| --- | --- | --- | --- | --- | --- | --- | --- | --- | --- | --- |
| *Addax nasomaculatus* | CR | CR | CR | C2a(ii); D |  | no | no |  | unknown | Less than 3 observations available to calculate EOO |
| *Ammotragus lervia* | VU | EN | EN | B1a,b(i,iii,v); C2a(i); D |  | no | no |  | 2055 |  |
| *Damaliscus lunatus* | LC | RE | RE |  |  |  |  |  | - |  |
| *Eudorcas rufifrons* | VU | EN | EN | C2a(i) |  | no | no |  | 142472 |  |
| *Gazella dorcas* | VU | VU | VU | C2a(i) |  | no | no |  | 512845 |  |
| *Hippotragus equinus* | LC | RE | RE |  |  |  |  |  | - |  |
| *Kobus kob* | LC | RE | RE |  |  |  |  |  | - |  |
| *Nanger dama* | CR | RE | RE |  |  |  |  |  | - |  |
| *Oryx dammah* | EW | EW | EW |  |  |  |  |  | - |  |
| *Redunca redunca* | LC | RE | RE |  |  |  |  |  | - |  |
| *Taurotragus derbianus* | VU | RE | RE |  |  |  |  |  | - |  |
| *Tragelaphus scriptus* | LC | RE | RE |  |  |  |  |  | - |  |
| *Giraffa camelopardalis* | VU | EW | EW |  |  |  |  |  | - |  |
| *Hippopotamus amphibius* | VU | CR | CR | C2a(i); D |  | no | no |  | 255 | Calculated following the linear habitat of the Senegal River including a buffer of 500 m |
| *Phacochoerus africanus* | LC | LC | LC |  |  |  |  |  | 204862 |  |
| *Canis lupaster* | LC | LC | LC |  |  |  |  |  | 488468 |  |
| *Lycaon pictus* | EN | RE | RE |  |  |  |  |  | - |  |
| *Vulpes pallida* | LC | LC | LC |  |  |  |  |  | 198113 |  |
| *Vulpes rueppellii* | LC | LC | LC |  |  |  |  |  | 438435 |  |
| *Vulpes zerda* | LC | LC | LC |  |  |  |  |  | 499712 |  |
| *Acinonyx jubatus* | VU | RE | RE |  |  |  |  |  | - |  |
| *Caracal caracal* | LC | NT | VU | C2a(i) | down | yes | no |  | 93782 |  |
| *Felis margarita* | LC | LC | LC |  |  |  |  |  | 182922 |  |
| *Felis silvestris* | LC | LC | LC |  |  |  |  |  | 381476 |  |
| *Leptailurus serval* | LC | VU | EN | B1a,b(i,iii); C2a(i) | down | yes | no |  | 2936 |  |
| *Panthera leo* | VU | RE | RE |  |  |  |  |  | - |  |
| *Panthera pardus* | VU | CR | CR | C2a(i); D |  | no | unknown |  | unknown | Less than 3 observations available to calculate EOO |
| *Atilax paludinosus* | LC | DD | DD |  |  |  |  |  | 13410 | Calculated following the linear habitat of the Senegal River including a buffer of 20 km |
| *Herpestes ichneumon* | LC | LC | LC |  |  |  |  |  | 143674 |  |
| *Herpestes sanguineus* | LC | LC | LC |  |  |  |  |  | 152594 |  |
| *Ichneumia albicauda* | LC | LC | LC |  |  |  |  |  | 49540 |  |
| *Crocuta crocuta* | LC | VU | EN | C2a(i) | down | yes | no |  | 277783 |  |
| *Hyaena hyaena* | NT | NT | VU | C2a(i) | down | yes | no |  | 390833 |  |
| *Aonyx capensis* | NT | DD | DD |  |  |  |  |  | 13410 | Calculated following the linear habitat of the Senegal River including a buffer of 20 km |
| *Ictonyx libyca* | LC | LC | LC |  |  |  |  |  | 237602 |  |
| *Ictonyx striatus* | LC | DD | DD |  |  |  |  |  | 22588 |  |
| *Mellivora capensis* | LC | LC | LC |  |  |  |  |  | 408812 |  |
| *Civettictis civetta* | LC | LC | LC |  |  |  |  |  | 57205 |  |
| *Genetta genetta* | LC | LC | LC |  |  |  |  |  | 228501 |  |
| *Taphozous nudiventris* | LC | DD | DD |  |  |  |  |  | unknown | Less than 3 observations available to calculate EOO |
| *Taphozous perforatus* | LC | NT | NT | B1a |  | no |  |  | 13410 | Calculated following the linear habitat of the Senegal River including a buffer of 20 km |
| *Asellia tridens* | LC | LC | LC |  |  |  |  |  | 128277 |  |
| *Hipposideros cf. caffer* | NE | NT | NT | B1a |  | no |  |  | 16811 |  |
| *Hipposideros tephrus* | LC | LC | NT | B1a | down | yes |  |  | 7450 |  |
| *Mops condylurus* | LC | DD | DD |  |  |  |  |  | 13410 | Calculated following the linear habitat of the Senegal River including a buffer of 20 km |
| *Tadarida aegyptiaca* | LC | DD | DD |  |  |  |  |  | unknown | Less than 3 observations available to calculate EOO |
| *Nycteris gambiensis* | LC | DD | DD |  |  |  |  |  | unknown | Less than 3 observations available to calculate EOO |
| *Nycteris hispida* | LC | LC | NT | B1a | down | yes |  |  | 13410 | Calculated following the linear habitat of the Senegal River including a buffer of 20 km |
| *Nycteris macrotis* | LC | LC | NT | B1a | down | yes |  |  | 13410 | Calculated following the linear habitat of the Senegal River including a buffer of 20 km |
| *Nycteris thebaica* | LC | DD | DD |  |  |  |  |  | 13410 | Calculated following the linear habitat of the Senegal River including a buffer of 20 km |
| *Eidolon helvum* | NT | LC | LC |  |  |  |  |  | 72013 |  |
| *Rhinolophus fumigatus* | LC | DD | DD |  |  |  |  |  | unknown | Less than 3 observations available to calculate EOO |
| *Rhinolophus landeri* | LC | DD | DD |  |  |  |  |  | unknown | Less than 3 observations available to calculate EOO |
| *Rhinopoma cystops* | LC | LC | LC |  |  |  |  |  | 24071 |  |
| *Rhinopoma hardwickei* | LC | LC | LC |  |  |  |  |  | 155488 |  |
| *Rhinopoma microphyllum* | LC | LC | LC |  |  |  |  |  | 916 |  |
| *Eptesicus floweri* | LC | DD | DD |  |  |  |  |  | unknown | Less than 3 observations available to calculate EOO |
| *Nycticeinops schlieffeni* | LC | LC | LC |  |  |  |  |  | 210089 |  |
| *Pipistrellus rueppellii* | LC | LC | LC |  |  |  |  |  | 7210 |  |
| *Scotophilus leucogaster* | LC | DD | DD |  |  |  |  |  | unknown | Less than 3 observations available to calculate EOO |
| *Atelerix albiventris* | LC | LC | LC |  |  |  |  |  | 178621 |  |
| *Paraechinus aethiopicus* | LC | LC | LC |  |  |  |  |  | 415136 |  |
| *Procavia capensis* | LC | LC | LC |  |  |  |  |  | 192871 |  |
| *Lepus spp.* | NE | NA | NA |  |  |  |  |  | - |  |
| *Ceratotherium simum* | NT | RE | RE |  |  |  |  |  | - |  |
| *Chlorocebus sabaeus* | LC | NT | VU | C2a(i) | down | yes |  |  | 34325 |  |
| *Erythrocebus patas* | NT | LC | LC |  |  |  |  |  | 103600 |  |
| *Papio papio* | NT | VU | VU | C2a(i) |  | no |  |  | 61999 |  |
| *Galago senegalensis* | LC | DD | DD |  |  |  |  |  | unknown | Less than 3 observations available to calculate EOO |
| *Loxodonta africana* | EN | RE | RE |  |  |  |  |  | - |  |
| *Felovia vae* | LC | LC | LC |  |  |  |  | Nearly-endemic | 231701 |  |
| *Jaculus cf. hirtipes* | NE | LC | LC |  |  |  |  |  | 240866 |  |
| *Jaculus jaculus* | LC | LC | LC |  |  |  |  |  | 461651 |  |
| *Hystrix cristata* | LC | LC | LC |  |  |  |  |  | 275831 |  |
| *Acomys airensis* | LC | LC | LC |  |  |  |  |  | 391512 |  |
| *Arvicanthis niloticus* | LC | LC | LC |  |  |  |  |  | 185358 |  |
| *Desmodilliscus braueri* | LC | LC | LC |  |  |  |  |  | 232308 |  |
| *Gerbillus amoenus* | LC | LC | LC |  |  |  |  |  | 314996 |  |
| *Gerbillus campestris* | LC | LC | LC |  |  |  |  |  | 379965 |  |
| *Gerbillus gerbillus* | LC | LC | LC |  |  |  |  |  | 423274 |  |
| *Gerbillus henleyi* | LC | LC | LC |  |  |  |  |  | 36723 |  |
| *Gerbillus nancillus* | DD | LC | LC |  |  |  |  |  | 68332 |  |
| *Gerbillus nigeriae* | LC | LC | LC |  |  |  |  | Endemic | 213037 |  |
| *Gerbillus pyramidum* | LC | LC | LC |  |  |  |  |  | 113620 |  |
| *Gerbillus tarabuli* | LC | LC | LC |  |  |  |  |  | 415239 |  |
| *Mastomys erythroleucus* | LC | LC | LC |  |  |  |  |  | 15711 |  |
| *Mastomys huberti* | LC | LC | LC |  |  |  |  |  | 13410 | Calculated following the linear habitat of the Senegal River including a buffer of 20 km |
| *Meriones crassus* | LC | LC | LC |  |  |  |  |  | 150946 |  |
| *Meriones libycus* | LC | DD | DD |  |  |  |  |  | unknown | Less than 3 observations available to calculate EOO |
| *Mus haussa* | LC | LC | LC |  |  |  |  |  | 4215 |  |
| *Mus musculus* | LC | NA | NA |  |  |  |  |  | - |  |
| *Pachyuromys duprasi* | LC | LC | LC |  |  |  |  |  | 58489 |  |
| *Praomys cf. daltoni* | NE | VU | VU | B1a,b(iii) |  | no |  | Endemic | 5002 |  |
| *Psammomys obesus* | LC | LC | LC |  |  |  |  |  | 37571 |  |
| *Rattus rattus* | LC | NA | NA |  |  |  |  |  | - |  |
| *Taterillus arenarius* | LC | LC | LC |  |  |  |  |  | 104232 |  |
| *Taterillus gracilis* | LC | DD | DD |  |  |  |  |  | unknown | Less than 3 observations available to calculate EOO |
| *Taterillus pygargus* | LC | DD | DD |  |  |  |  |  | unknown | Less than 3 observations available to calculate EOO |
| *Taterillus tranieri* | LC | LC | NT | D2 | down | yes |  | Nearly-endemic | unknown | Less than 3 observations available to calculate EOO |
| *Euxerus erythropus* | LC | LC | LC |  |  |  |  |  | 265685 |  |
| *Crocidura cinderella* | LC | DD | DD |  |  |  |  |  | unknown | Less than 3 observations available to calculate EOO |
| *Crocidura fuscomurina* | LC | DD | DD |  |  |  |  |  | unknown | Less than 3 observations available to calculate EOO |
| *Crocidura lusitania* | LC | LC | LC |  |  |  |  |  | 38004 |  |
| *Crocidura nanilla* | LC | DD | DD |  |  |  |  |  | unknown | Less than 3 observations available to calculate EOO |
| *Crocidura olivieri* | LC | DD | DD |  |  |  |  |  | unknown | Less than 3 observations available to calculate EOO |
| *Crocidura viaria* | LC | LC | LC |  |  |  |  |  | 240532 |  |
| *Orycteropus afer* | LC | DD | DD |  |  |  |  |  | 13410 | Calculated following the linear habitat of the Senegal River including a buffer of 20 km |

[1] IUCN Standards and Petitions Committee. Guidelines for Using the IUCN Red List Categories and Criteria. Version 14. Gland, Switzerland and Cambridge, UK: IUCN; 2019. [cited from 2021 November 29]. Available from: http://www.iucnredlist.org/documents/RedListGuidelines.pdf.

[2] IUCN. Guidelines for application of IUCN Red List Criteria at regional and national levels. Version 4.0. Gland, Switzerland and Cambridge, UK: IUCN; 2012.

[3] IUCN. The IUCN Red List of Threatened Species. Version 2021-2. Gland, Switzerland and Cambridge, UK: IUCN; 2021. [cited 2021 October 12]. Available from: https://www.iucnredlist.org.
