# Supplementary material for: Diversity, distribution and conservation of land mammals in Mauritania, North-West Africa
Source: PLoS One. 2022 Aug 1;17(8):e0269870. doi: 10.1371/journal.pone.0269870 (PMC9342785; doi:10.1371/journal.pone.0269870)
Supplement: S6 Text — List of occupied habitats by land mammals in Mauritania following the IUCN standard habitat classification scheme [1]. (DOCX) [file pone.0269870.s024.docx]

**S21 Text –** **Habitats.** List of occupied habitats by land mammals in Mauritania following the IUCN standard habitat classification scheme [1].

|  | 2. Savanna | 3. Shrubland | 4. Grassland | | 5. Wetlands (inland) | | | | | | 6. Rocky Areas | 7. Caves & Subterranean Habitats (non-aquatic) | | 8. Desert | 13. Marine Coastal / Supratidal |  |
| --- | --- | --- | --- | --- | --- | --- | --- | --- | --- | --- | --- | --- | --- | --- | --- | --- |
|  | 2.1. - Dry | 3.5.– Subtropical / tropical dry | 4.5.– Subtropical / tropical dry | 4.6.– Subtropical / tropical seasonally wet / flooded | 5.1.– Permanent rivers / streams / creeks | 5.2.– Seasonal / intermittent / irregular rivers / streams / creeks | 5.4.– Bogs, marshes, swamps, fens, peatlands | 5.7.– Permanent freshwater marshes / pools (under 8 ha) | 5.8.– Seasonal / intermittent freshwater marshes / pools (under 8 ha) | 5.9. – Freshwater springs and oases | - | 7.1.– Caves | 7.2.– Other subterranean habitats | 8.1.– Hot | 13.3 Coastal Sand Dunes | **TOTAL** |
| *Addax nasomaculatus* |  |  |  |  |  |  |  |  |  |  |  |  |  | 1 |  | **1** |
| *Ammotragus lervia* |  |  |  |  |  |  |  |  |  |  | 1 |  |  | 1 |  | **2** |
| *Eudorcas rufifrons* | 1 | 1 | 1 | 1 |  |  |  |  |  |  |  |  |  |  | 1 | **5** |
| *Gazella dorcas* |  | 1 | 1 | 1 |  |  |  |  |  |  |  |  |  | 1 | 1 | **5** |
| *Hippopotamus amphibius* |  |  |  |  | 1 | 1 | 1 |  |  |  |  |  |  |  |  | **3** |
| *Phacochoerus africanus* | 1 | 1 | 1 | 1 | 1 | 1 | 1 | 1 | 1 | 1 |  |  |  |  |  | **10** |
| *Canis lupaster* | 1 | 1 | 1 | 1 | 1 | 1 | 1 | 1 | 1 | 1 | 1 |  |  | 1 | 1 | **13** |
| *Vulpes pallida* | 1 | 1 | 1 | 1 | 1 | 1 | 1 | 1 | 1 | 1 |  |  |  |  |  | **10** |
| *Vulpes rueppellii* |  |  | 1 | 1 | 1 | 1 | 1 | 1 | 1 | 1 | 1 |  |  | 1 |  | **10** |
| *Vulpes zerda* |  |  |  |  |  |  |  |  |  |  |  |  |  | 1 |  | **1** |
| *Caracal caracal* | 1 | 1 |  |  |  |  |  |  |  |  | 1 |  |  |  |  | **3** |
| *Felis margarita* |  |  |  |  |  |  |  |  |  |  |  |  |  | 1 |  | **1** |
| *Felis silvestris* | 1 | 1 | 1 | 1 | 1 | 1 | 1 | 1 | 1 | 1 | 1 |  |  |  | 1 | **12** |
| *Leptailurus serval* | 1 |  |  |  |  |  |  |  |  |  |  |  |  |  |  | **1** |
| *Panthera pardus* | 1 | 1 |  |  |  |  |  |  |  |  | 1 |  |  |  | 1 | **4** |
| *Atilax paludinosus* |  |  |  |  | 1 | 1 | 1 | 1 | 1 | 1 |  |  |  |  |  | **6** |
| *Herpestes ichneumon* | 1 | 1 | 1 | 1 | 1 | 1 | 1 | 1 | 1 | 1 | 1 |  |  |  |  | **11** |
| *Herpestes sanguineus* | 1 | 1 | 1 | 1 | 1 | 1 | 1 | 1 | 1 | 1 | 1 |  |  |  |  | **11** |
| *Ichneumia albicauda* | 1 | 1 | 1 | 1 | 1 | 1 | 1 | 1 | 1 | 1 | 1 |  |  |  |  | **11** |
| *Crocuta crocuta* | 1 | 1 | 1 | 1 |  | 1 | 1 |  |  |  | 1 |  |  |  |  | **7** |
| *Hyaena hyaena* | 1 | 1 | 1 |  |  |  |  |  |  |  | 1 |  |  | 1 | 1 | **6** |
| *Aonyx capensis* |  |  |  |  | 1 | 1 | 1 | 1 | 1 | 1 |  |  |  |  |  | **6** |
| *Ictonyx libyca* | 1 | 1 | 1 | 1 | 1 | 1 | 1 | 1 | 1 | 1 | 1 |  |  | 1 |  | **12** |
| *Ictonyx striatus* | 1 | 1 | 1 | 1 | 1 | 1 | 1 | 1 | 1 | 1 | 1 |  |  |  |  | **11** |
| *Mellivora capensis* | 1 | 1 | 1 | 1 | 1 | 1 | 1 | 1 | 1 | 1 | 1 |  |  | 1 |  | **12** |
| *Civettictis civetta* | 1 | 1 | 1 | 1 | 1 | 1 | 1 | 1 | 1 | 1 | 1 |  |  |  |  | **11** |
| *Genetta genetta* | 1 | 1 | 1 | 1 | 1 | 1 | 1 | 1 | 1 | 1 | 1 |  |  |  | 1 | **12** |
| *Taphozous nudiventris* |  |  |  |  |  |  |  |  |  |  | 1 |  |  |  |  | **1** |
| *Taphozous perforatus* | 1 | 1 | 1 | 1 |  |  |  |  |  |  |  | 1 | 1 |  |  | **6** |
| *Asellia tridens* |  |  |  |  |  |  |  |  |  |  | 1 | 1 | 1 | 1 |  | **4** |
| *Hipposideros cf. caffer* | 1 | 1 |  |  | 1 | 1 | 1 | 1 | 1 | 1 | 1 | 1 | 1 | 1 |  | **12** |
| *Hipposideros tephrus* | 1 | 1 |  |  | 1 | 1 | 1 | 1 | 1 | 1 | 1 | 1 | 1 | 1 |  | **12** |
| *Mops condylurus* | 1 |  |  |  |  |  |  |  |  |  |  | 1 | 1 |  |  | **3** |
| *Tadarida aegyptiaca* |  |  |  |  |  |  |  |  |  |  | 1 | 1 | 1 |  |  | **3** |
| *Nycteris hispida* | 1 | 1 | 1 | 1 | 1 | 1 | 1 | 1 | 1 | 1 |  | 1 | 1 |  |  | **12** |
| *Nycteris macrotis* | 1 | 1 | 1 | 1 | 1 | 1 | 1 | 1 | 1 | 1 |  | 1 | 1 |  |  | **12** |
| *Nycteris thebaica* | 1 | 1 | 1 | 1 | 1 | 1 | 1 | 1 | 1 | 1 |  | 1 | 1 |  |  | **12** |
| *Eidolon helvum* | 1 | 1 | 1 | 1 | 1 | 1 | 1 | 1 | 1 | 1 |  |  |  |  |  | **10** |
| *Rhinolophus fumigatus* | 1 |  |  |  |  |  |  |  |  |  |  | 1 | 1 |  |  | **3** |
| *Rhinolophus landeri* |  |  |  |  |  |  |  |  |  |  | 1 | 1 | 1 | 1 |  | **4** |
| *Rhinopoma cystops* |  |  |  |  |  |  |  |  |  |  | 1 | 1 | 1 | 1 |  | **4** |
| *Rhinopoma hardwickei* |  | 1 | 1 | 1 | 1 | 1 | 1 | 1 | 1 | 1 | 1 | 1 | 1 | 1 |  | **13** |
| *Rhinopoma microphyllum* |  | 1 | 1 | 1 | 1 | 1 | 1 | 1 | 1 | 1 | 1 | 1 | 1 | 1 |  | **13** |
| *Eptesicus floweri* |  |  |  |  | 1 | 1 | 1 | 1 | 1 |  |  | 1 | 1 |  |  | **7** |
| *Nycticeinops schlieffeni* | 1 | 1 | 1 | 1 | 1 | 1 | 1 | 1 | 1 | 1 | 1 | 1 | 1 | 1 |  | **14** |
| *Pipistrellus rueppellii* | 1 | 1 | 1 | 1 | 1 | 1 | 1 | 1 | 1 | 1 | 1 | 1 | 1 | 1 |  | **14** |
| *Scotophilus leucogaster* |  |  |  |  |  |  |  |  |  |  | 1 | 1 | 1 | 1 |  | **4** |
| *Atelerix albiventris* | 1 | 1 | 1 | 1 | 1 | 1 | 1 | 1 | 1 | 1 | 1 |  |  |  | 1 | **12** |
| *Paraechinus aethiopicus* |  | 1 | 1 | 1 | 1 | 1 | 1 | 1 | 1 | 1 | 1 |  |  | 1 | 1 | **12** |
| *Procavia capensis* |  |  |  |  |  |  |  |  |  |  | 1 |  |  |  |  | **1** |
| *Chlorocebus sabaeus* | 1 |  |  |  |  |  | 1 |  |  | 1 | 1 |  |  |  |  | **4** |
| *Erythrocebus patas* | 1 | 1 |  |  | 1 | 1 | 1 | 1 | 1 | 1 | 1 |  |  |  |  | **9** |
| *Papio papio* | 1 |  |  |  | 1 | 1 | 1 | 1 | 1 | 1 | 1 |  |  |  |  | **8** |
| *Galago senegalensis* | 1 |  |  |  |  |  |  |  |  |  |  |  |  |  |  | **1** |
| *Felovia vae* |  | 1 |  |  |  |  |  |  |  |  | 1 |  |  |  |  | **2** |
| *Jaculus cf. hirtipes* |  | 1 | 1 | 1 |  |  |  |  |  |  |  |  |  | 1 | 1 | **5** |
| *Jaculus jaculus* |  | 1 | 1 | 1 |  |  |  |  |  |  |  |  |  | 1 | 1 | **5** |
| *Hystrix cristata* | 1 | 1 | 1 | 1 | 1 | 1 | 1 | 1 | 1 | 1 | 1 |  |  |  |  | **11** |
| *Acomys airensis* |  | 1 | 1 | 1 |  |  |  |  |  |  | 1 |  |  | 1 |  | **5** |
| *Arvicanthis niloticus* | 1 | 1 | 1 | 1 | 1 | 1 | 1 | 1 | 1 | 1 |  |  |  | 1 | 1 | **12** |
| *Desmodilliscus braueri* | 1 | 1 | 1 | 1 | 1 | 1 | 1 | 1 | 1 | 1 | 1 |  |  | 1 |  | **12** |
| *Gerbillus amoenus* |  | 1 | 1 | 1 | 1 | 1 | 1 | 1 | 1 | 1 | 1 |  |  | 1 | 1 | **12** |
| *Gerbillus campestris* |  | 1 |  |  |  |  |  |  |  |  | 1 |  |  | 1 |  | **3** |
| *Gerbillus gerbillus* |  | 1 | 1 | 1 |  |  |  |  |  |  | 1 |  |  | 1 | 1 | **6** |
| *Gerbillus henleyi* |  | 1 |  |  |  |  |  |  |  |  | 1 |  |  | 1 |  | **3** |
| *Gerbillus nancillus* |  | 1 |  |  |  |  |  |  |  |  | 1 |  |  | 1 | 1 | **4** |
| *Gerbillus nigeriae* | 1 | 1 | 1 | 1 | 1 | 1 | 1 | 1 | 1 | 1 | 1 |  |  | 1 | 1 | **13** |
| *Gerbillus pyramidum* |  | 1 | 1 | 1 |  |  |  |  |  |  | 1 |  |  | 1 | 1 | **6** |
| *Gerbillus tarabuli* | 1 | 1 | 1 | 1 | 1 | 1 | 1 | 1 | 1 | 1 | 1 |  |  | 1 | 1 | **13** |
| *Mastomys erythroleucus* | 1 |  |  |  | 1 | 1 | 1 | 1 | 1 | 1 | 1 |  |  |  |  | **8** |
| *Mastomys huberti* | 1 |  |  |  | 1 | 1 | 1 | 1 | 1 | 1 |  |  |  |  | 1 | **8** |
| *Meriones crassus* |  | 1 |  |  |  |  |  |  |  |  | 1 |  |  | 1 |  | **3** |
| *Meriones libycus* |  |  |  |  |  |  |  |  |  |  |  |  |  | 1 | 1 | **2** |
| *Mus haussa* |  | 1 |  |  | 1 | 1 | 1 | 1 | 1 | 1 | 1 |  |  |  |  | **8** |
| *Pachyuromys duprasi* |  |  |  |  |  |  |  |  |  |  | 1 |  |  | 1 | 1 | **3** |
| *Praomys cf. daltoni* | 1 | 1 |  |  |  |  |  |  |  |  | 1 |  |  |  |  | **3** |
| *Psammomys obesus* |  |  |  |  |  |  |  |  |  |  |  |  |  | 1 | 1 | **2** |
| *Taterillus arenarius* | 1 | 1 | 1 | 1 | 1 | 1 | 1 | 1 | 1 | 1 |  |  |  | 1 | 1 | **12** |
| *Taterillus gracilis* | 1 | 1 |  |  |  |  |  |  |  |  | 1 |  |  |  |  | **3** |
| *Taterillus pygargus* |  | 1 | 1 | 1 |  |  |  |  |  |  | 1 |  |  |  |  | **4** |
| *Taterillus tranieri* |  | 1 | 1 | 1 |  |  |  |  |  |  | 1 |  |  |  |  | **4** |
| *Euxerus erythropus* | 1 | 1 | 1 | 1 | 1 | 1 | 1 | 1 | 1 | 1 | 1 |  |  |  | 1 | **12** |
| *Crocidura cinderella* |  |  |  |  |  |  |  |  |  |  |  |  |  | 1 | 1 | **2** |
| *Crocidura fuscomurina* |  | 1 |  |  |  |  |  |  |  |  |  |  |  |  | 1 | **2** |
| *Crocidura lusitania* |  | 1 |  |  |  |  |  |  |  |  |  |  |  | 1 | 1 | **3** |
| *Crocidura nanilla* |  |  | 1 | 1 | 1 | 1 | 1 | 1 | 1 | 1 |  |  |  |  |  | **8** |
| *Crocidura olivieri* | 1 |  |  |  |  |  |  |  |  |  | 1 |  |  |  |  | **2** |
| *Crocidura viaria* |  | 1 | 1 | 1 | 1 | 1 | 1 | 1 | 1 | 1 | 1 |  |  |  |  | **10** |
| *Orycteropus afer* | 1 | 1 | 1 | 1 | 1 | 1 | 1 | 1 | 1 | 1 |  |  |  |  |  | **10** |
|  |  |  |  |  |  |  |  |  |  |  |  |  |  |  |  |  |
| TOTAL | **47** | **59** | **46** | **45** | **45** | **46** | **47** | **44** | **44** | **44** | **57** | **18** | **18** | **41** | **27** |  |

[1] IUCN. Habitats Classification Scheme (Version 3.1). Gland, Switzerland and Cambridge, UK: IUCN; 2021. [cited on 2021 December 01]. Available from: https://www.iucnredlist.org/resources/habitat-classification-scheme
