# Supplementary material for: Diversity, distribution and conservation of land mammals in Mauritania, North-West Africa
Source: PLoS One. 2022 Aug 1;17(8):e0269870. doi: 10.1371/journal.pone.0269870 (PMC9342785; doi:10.1371/journal.pone.0269870)
Supplement: S7 Text — List of threats affecting land mammals categorised as threatened (CR, EN, VU) and Near-Threatened (NT) under the national Red List assessment following the IUCN standard threats classification scheme [1]. (DOCX) [file pone.0269870.s025.docx]

**S22Text –** **Threats.** List of threats affecting land mammals categorised as threatened (CR, EN, VU) and Near-Threatened (NT) under the national Red List assessment following the IUCN standard threats classification scheme [1].

|  | *Addax nasomaculatus* | *Ammotragus lervia* | *Eudorcas rufifrons* | *Gazella dorcas* | *Hippopotamus amphibius* | *Caracal caracal* | *Leptailurus serval* | *Panthera pardus* | *Crocuta crocuta* | *Hyaena hyaena* | *Taphozous perforatus* | *Hipposideros cf. caffer* | *Chlorocebus sabaeus* | *Papio papio* | *Praomys cf. daltoni* | TOTAL |
| --- | --- | --- | --- | --- | --- | --- | --- | --- | --- | --- | --- | --- | --- | --- | --- | --- |
| 1 Residential & commercial development |  |  |  |  |  |  |  |  |  |  |  |  |  |  |  |  |
| *1.1 Housing & urban areas* |  |  | 1 | 1 | 1 | 1 | 1 | 1 | 1 | 1 |  |  | 1 | 1 | 1 | **11** |
| *1.2 Commercial & industrial areas* |  |  | 1 | 1 |  |  | 1 |  | 1 | 1 | 1 | 1 | 1 |  |  | **8** |
|  |  |  |  |  |  |  |  |  |  |  |  |  |  |  |  |  |
| 2 Agriculture & aquaculture |  |  |  |  |  |  |  |  |  |  |  |  |  |  |  |  |
| *2.1 Annual & perennial non-timber crops* |  |  |  |  |  |  |  |  |  |  |  |  |  |  |  |  |
| 2.1.1 Shifting agriculture |  |  | 1 | 1 | 1 |  |  |  |  |  | 1 | 1 | 1 | 1 | 1 | **8** |
| 2.1.2 Small-holder farming |  |  | 1 | 1 | 1 | 1 | 1 | 1 | 1 | 1 | 1 | 1 | 1 | 1 | 1 | **13** |
| *2.3 Livestock farming & ranching* |  |  |  |  |  |  |  |  |  |  |  |  |  |  |  |  |
| 2.3.1 Nomadic grazing | 1 | 1 | 1 | 1 |  | 1 | 1 | 1 | 1 | 1 |  |  |  | 1 | 1 | **11** |
| 2.3.2 Small-holder grazing, ranching or farming |  |  | 1 | 1 | 1 | 1 | 1 | 1 | 1 | 1 |  |  | 1 | 1 | 1 | **11** |
|  |  |  |  |  |  |  |  |  |  |  |  |  |  |  |  |  |
| 3 Energy production & mining |  |  |  |  |  |  |  |  |  |  |  |  |  |  |  |  |
| *3.1 Oil & gas drilling* | 1 |  |  | 1 |  |  |  |  |  | 1 |  |  |  |  |  | **3** |
| *3.2 Mining & quarrying* | 1 | 1 |  | 1 |  |  |  |  |  | 1 |  |  |  |  |  | **4** |
|  |  |  |  |  |  |  |  |  |  |  |  |  |  |  |  |  |
| 4 Transportation & service corridors |  |  |  |  |  |  |  |  |  |  |  |  |  |  |  |  |
| *4.1 Roads & railroads* | 1 | 1 | 1 | 1 | 1 | 1 | 1 | 1 | 1 | 1 |  |  | 1 | 1 | 1 | **13** |
|  |  |  |  |  |  |  |  |  |  |  |  |  |  |  |  |  |
| 5 Biological resource use |  |  |  |  |  |  |  |  |  |  |  |  |  |  |  |  |
| *5.1 Hunting & collecting terrestrial animals* |  |  |  |  |  |  |  |  |  |  |  |  |  |  |  |  |
| 5.1.1 Intentional use (species being assessed is the target) | 1 | 1 | 1 | 1 |  |  |  |  |  |  |  |  |  |  |  | **4** |
| 5.1.3 Persecution/control |  |  |  |  | 1 | 1 | 1 | 1 | 1 | 1 |  |  | 1 | 1 |  | **8** |
| *5.3 Logging & wood harvesting* |  |  |  |  |  |  |  |  |  |  |  |  |  |  |  |  |
| 5.3.3 Unintentional effects: subsistence/small scale (species being assessed is not the target)[harvest] |  |  |  |  |  | 1 | 1 |  | 1 | 1 | 1 | 1 | 1 | 1 |  | **8** |
|  |  |  |  |  |  |  |  |  |  |  |  |  |  |  |  |  |
| 6 Human intrusions & disturbance |  |  |  |  |  |  |  |  |  |  |  |  |  |  |  |  |
| *6.1 Recreational activities* |  | 1 |  | 1 |  | 1 | 1 | 1 | 1 | 1 |  |  | 1 | 1 |  | **9** |
| *6.2 War, civil unrest & military exercises* | 1 |  | 1 | 1 |  |  |  | 1 |  |  |  |  |  |  |  | **4** |
| *6.3 Work & other activities* |  |  |  |  |  |  |  |  |  |  |  |  |  |  |  |  |
| 6.3.1 Prospecting activities | 1 |  |  | 1 |  |  |  |  |  |  |  |  |  |  |  | **2** |
|  |  |  |  |  |  |  |  |  |  |  |  |  |  |  |  |  |
| 7 Natural system modifications |  |  |  |  |  |  |  |  |  |  |  |  |  |  |  |  |
| *7.1 Fire & fire suppression* |  |  |  |  |  |  |  |  |  |  |  |  |  |  |  |  |
| 7.1.1 Increase in fire frequency/intensity |  |  | 1 |  |  |  | 1 | 1 | 1 | 1 | 1 | 1 | 1 | 1 | 1 | **10** |
| *7.2 Dams & water management/use* |  |  |  |  |  |  |  |  |  |  |  |  |  |  |  |  |
| 7.2.1 Abstraction of surface water (domestic use) |  |  | 1 |  | 1 | 1 | 1 | 1 | 1 | 1 | 1 | 1 | 1 | 1 | 1 | **12** |
| 7.2.3 Abstraction of surface water (agricultural use) |  |  | 1 |  | 1 | 1 | 1 | 1 | 1 | 1 | 1 | 1 | 1 | 1 | 1 | **12** |
| 7.2.5 Abstraction of ground water (domestic use) |  |  |  |  |  |  |  |  |  |  |  |  |  | 1 | 1 | **2** |
| 7.2.7 Abstraction of ground water (agricultural use) |  |  |  |  |  |  |  |  |  |  |  |  |  | 1 | 1 | **2** |
| 7.2.10 Large dams |  |  |  |  | 1 |  |  |  |  |  |  |  |  |  |  | **1** |
|  |  |  |  |  |  |  |  |  |  |  |  |  |  |  |  |  |
| 8 Invasive & other problematic species, genes & diseases |  |  |  |  |  |  |  |  |  |  |  |  |  |  |  |  |
| *8.1 Invasive non-native/alien species/diseases* |  |  |  |  |  |  |  |  |  |  |  |  |  |  |  |  |
| 8.1.2 Named Species (*Tamarix*) |  |  |  |  | 1 |  |  |  |  |  |  |  | 1 |  |  | **2** |
|  |  |  |  |  |  |  |  |  |  |  |  |  |  |  |  |  |
| 9 Pollution |  |  |  |  |  |  |  |  |  |  |  |  |  |  |  |  |
| *9.1 Domestic & urban waste water* |  |  |  |  |  |  |  |  |  |  |  |  |  |  |  |  |
| 9.1.2 Run-off |  |  |  |  | 1 |  |  |  |  |  |  |  |  |  |  | **1** |
| *9.2 Industrial & military effluents* |  |  |  |  |  |  |  |  |  |  |  |  |  |  |  |  |
| 9.2.2 Seepage from mining |  | 1 | 1 | 1 |  |  |  |  |  |  |  |  |  |  |  | **3** |
| *9.3 Agricultural & forestry effluents* |  |  |  |  |  |  |  |  |  |  |  |  |  |  |  |  |
| 9.3.1 Nutrient loads |  |  | 1 |  | 1 |  |  |  |  |  |  |  | 1 | 1 | 1 | **5** |
| 9.3.2 Soil erosion, sedimentation |  |  |  |  |  |  |  |  |  |  |  |  |  |  | 1 | **1** |
| 9.3.3 Herbicides & pesticides |  |  | 1 |  | 1 |  |  |  |  |  | 1 | 1 | 1 | 1 | 1 | **7** |
| *9.4 Garbage & solid waste* |  |  |  |  | 1 |  |  |  |  |  |  |  |  |  | 1 | **2** |
|  |  |  |  |  |  |  |  |  |  |  |  |  |  |  |  |  |
| 11 Climate change & severe weather |  |  |  |  |  |  |  |  |  |  |  |  |  |  |  |  |
| *11.1 Habitat shifting & alteration* | 1 | 1 | 1 | 1 | 1 | 1 | 1 | 1 | 1 | 1 | 1 | 1 | 1 | 1 | 1 | **15** |
| *11.2 Droughts* | 1 | 1 | 1 | 1 | 1 | 1 | 1 | 1 | 1 | 1 | 1 | 1 | 1 | 1 | 1 | **15** |
| *11.3 Temperature extremes* | 1 | 1 | 1 | 1 | 1 | 1 | 1 | 1 | 1 | 1 | 1 | 1 | 1 | 1 | 1 | **15** |
| *11.4 Storms & flooding* |  |  |  |  |  |  |  |  |  |  |  |  |  |  |  |  |
| 11.4.1 Dust Storms | 1 | 1 | 1 | 1 | 1 | 1 | 1 | 1 | 1 | 1 | 1 | 1 | 1 | 1 | 1 | **15** |
|  |  |  |  |  |  |  |  |  |  |  |  |  |  |  |  |  |
| TOTAL | **11** | **10** | **19** | **18** | **18** | **14** | **16** | **15** | **16** | **18** | **12** | **12** | **19** | **20** | **19** |  |

[1] IUCN. Threats Classification Scheme (Version 3.2). Gland, Switzerland and Cambridge, UK: IUCN; 2021. [cited on 2021 December 01]. Available from: https://www.iucnredlist.org/resources/threat-classification-scheme
